# Supplementary material for: Palladium-catalyzed Suzuki-Miyaura cross-couplings of stable glycal boronates for robust synthesis of C-1 glycals
Source: Nat Commun. 2024 Jun 19;15:5228. doi: 10.1038/s41467-024-49547-9 (PMC11187158; doi:10.1038/s41467-024-49547-9)
Supplement: Supplementary file 1 — Supplementary Information [file 41467_2024_49547_MOESM1_ESM.pdf]

SUPPLEMENTARY INFORMATION

Palladium-catalyzed Suzuki-Miyaura cross-couplings of stable glycal boronates for robust synthesis of C-1 glycals

Anrong Chen[1], Yang Han[1], Rongfeng Wu[2], Bo Yang[1], Lijuan Zhu[3*], and Feng Zhu[1*]

[1] Frontiers Science Center for Transformative Molecules (FSCTM), Center for Chemical Glycobiology, Shanghai Key Laboratory for Molecular Engineering of Chiral Drugs, School of Chemistry and Chemical Engineering, Zhangjiang Institute for Advanced Study, Shanghai Jiao Tong University, Shanghai, 200240, P. R. China

[2] Discovery Chemistry Unit, HitGen Inc., Chengdu, Sichuan, 610200, P. R. China

[3] Institute of Molecular Medicine, Renji Hospital, School of Medicine, Shanghai Jiao Tong University, 160 Pujian Road, Shanghai, 200127, P. R. China

*lijuanzhu@sjtu.edu.cn

*fzchem@sjtu.edu.cn

Table of Contents

1. Supplementary Methods 3

2. Supplementary Notes 4

2.1 Detailed experimental procedures 7

2.2 Detailed experimental procedures for preparation of start materials 7

2.3 Detailed experimental procedures for the palladium-catalyzed Suzuki-Miyaura cross-couplings 20

2.4 Detailed experimental procedures for the one pot two-step reaction 54

2.5 Detailed experimental procedures for on-DNA glycal-based Suzuki–Miyaura cross-couplings 59

2.6 Detailed experimental procedures for late-stage glycodiversifications and total synthesis 86

2.7 Copies of NMR spectra 88

3. Supplementary References 94

1. Supplementary Methods

Commercially available materials were used as received without further purification unless otherwise noted. All reactions were carried out under anhydrous N_2 in oven-dried glassware. $Pd(PPh_3)_2Cl_2$, K_3PO_4, Anhydrous DMF (99.5% purity) were purchased from Adamas. Automated column chromatography was performed on a SepaBean^{TM} machine T (SanTai Technologies, China) using Silicycle high-resolution SiO_2 cartridges unless otherwise noted. ^1H and ^{13}C NMR spectra were recorded on Bruker 400/500 MHz instruments and were reported as follows: chemical shift (δ), multiplicity (s = singlet, d = doublet, t = triplet, q =quartet, dd = doublet doublet, br = broad, m = multiplet), coupling constants (Hz), and integration. The residual solvent reference peaks were used from published literature. High-resolution mass spectra (HR-MS) were recorded on a Waters Micromass Q-Tof Premier mass spectrometer. Optical rotations were measured on an Anton Paar MCP550 automatic polarimeter using a 100 mm path-length cell at 589 nm. Thin layer chromatography was used to monitor reaction progress and analyze fractions from column chromatography.

2. Supplementary Notes

2.1 Detailed experimental procedures

General procedure for the palladium-catalyzed Suzuki-Miyaura cross-couplings

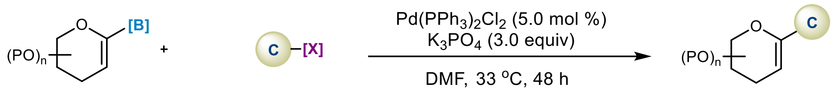

12 14 7

Procedure A: Glycal boronates **12** (1.20 equiv), electrophilic reagent **14** (1.00 equiv), $Pd(PPh_3)_2Cl_2$ (5.00 mol%) , K_3PO_4 (3.00 equiv) were added to a one-dram vial with a screw-top septum, and the vial was then evacuated and refilled with N_2 (3×). Anhydrous DMF (2.00 mL) were added, and the reaction mixture was stirred at 33 °C for 48 h, cooled to rt, and concentrated. The crude material was purified by column chromatography on SiO_2.

General procedure for the one pot two-step reactions

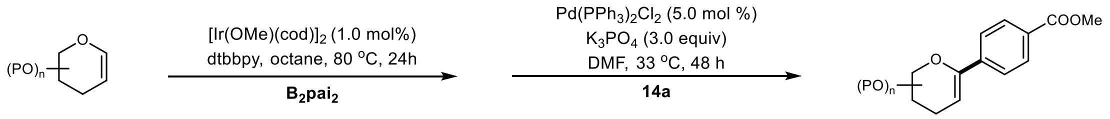

18 7e, 7f, 7h-7j, 7m, 7n, 15a

Procedure B: Glycals **18** (1.80 equiv), B_2Pai_2 (1.20 equiv), $[IrOMe(cod)]_2$ (2.40 mol%), dtbbpy (4.80 mol%) were added to a one-dram vial with a screw-top septum, and the vial was then evacuated and refilled with N_2 (3×). Anhydrous octane (1.00 mL) was added, and the reaction mixture was stirred at 80 °C for 24 h. After the reaction mixture was cooled to room temperature and concentrated under vacuum, the electrophilic reagent (1.00 equiv), $Pd(PPh_3)_2Cl_2$ (5.00 mol%), K_3PO_4 (3.00 equiv) were added. The vial was then evacuated and refilled with N_2 (3×). Anhydrous DMF (2.00 mL) were added, and the reaction mixture was stirred at 33 °C for 48 h, cooled to rt, and concentrated. The crude material was purified by column chromatography on SiO_2.

General procedure for the on-DNA glycal-based Suzuki–Miyaura couplings

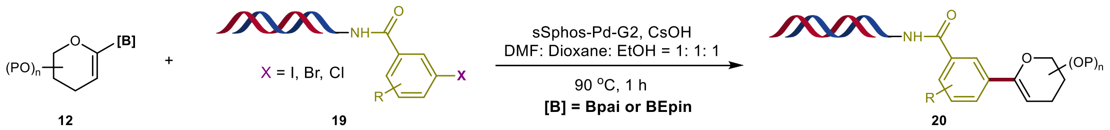

12 19 20

Procedure C: To each well of a 250 µL 96-well PCR microplate was added DNA (1.00 equiv, 10.0 nmol, 10.0 µL, 2.00 mM in H_2O), CsOH (100 equiv, 1.00 µmol, 2.00 µL, 0.50 M in H_2O), glycal boronates (250 equiv, 2.50 µmol, 16.70 µL, 0.15 M in DMF: Dioxane: EtOH = 1: 1: 1), and sSphos-Pd-G2 (2.00 equiv, 20.00 nmol, 1.00µL, 20.00 mM in DMF) sequentially. The solution was mixed with vortex for 30 seconds, then reacted at 90 °C for 1 hour. When time's up, adding DDTC (100 equiv, 1.00 µmol, 2.00

μL, 0.50 M in H_2O) to each well, the solution was mixed with vortex for 30 seconds, then reacted at 30 °C for 10 min. When time's up, centrifuging under 4 °C with 4000 rpm for 10 min. Remove precipitates and take supernatant for further ethanol precipitation, add 10% (v/v) 5 M NaCl solution and 3 times the volume of absolute ethanol to supernatant, cooled under -78°C for 2 hours. Centrifuge under 4 °C with 4000 rpm for 30 min. The precipitated material was isolated as a pellet by centrifugation and subsequent removal of the supernatant. 75% aq. ethanol was then added to the pellet and the mixture was centrifuged again. The supernatant again was discarded and the DNA pellet was dried under vacuum. The DNA pellet was redissolved in H_2O as 0.5 mM. Finally, 1-2 nmol DNA was taken for LC-MS detection.

General procedure for the preparation of start materials

General procedure for the preparation of disaccharides glycals:

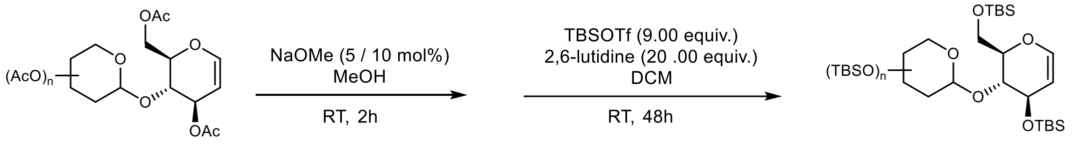

18k-s/18l-s **18k/18l**

Procedure D: Prepared according to a literature precedent with slight modification,[1] a solution of the fully acetylated protected disaccharide glycals **18k-s/18l-s** (1.00 equiv) in anhydrous MeOH at room temperature was treated with NaOMe (0.100 equiv). The reaction mixture was stirred for 2 h, concentrated, and azeotroped with toluene (2×). Subsequently, the crude underivatized disaccharides glycals were transferred to a dry flask under N_2. Dry methylene chloride was added, and the solution was stirred until most of the sugar dissolved. Dry 2,6-lutidine (20.0 equiv) was added, followed by *tert*-butyl-dimethyl triflate (9.00 equiv). Upon addition of the triflate, the previously undissolved sugar dissolved completely, and the reaction mixture became clear. The reaction mixture was stirred at room temperature for 48 h. The product was extracted with ether and washed with water. The aqueous layer was then extracted with ether. The product in the combined ether extracts was rotary evaporated, and the crude mixture was purified by chromatographic purification on SiO_2.

General procedure for the preparation of glycal boronates

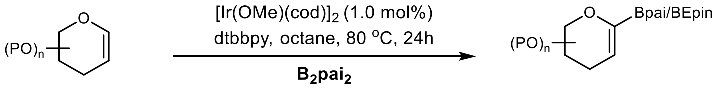

18 **12**

Procedure E: Prepared according to a literature precedent with slight modification.[2] To a Schlenk flask, $[Ir(OMe)(cod)]_2$ (1.00 mol%), dtbbpy (2.00 mol%), and either $B_2(pin)_2$ or $B_2(Epin)_2$ (1.00 equiv) were added, followed by purging with nitrogen. Subsequently, *n*-Octane and glycals (1.50 equiv) were added, and the mixture underwent stirring at 80 °C for 24 h. Following the completion of the reaction, the mixture was allowed to cool to room temperature and then concentrated under vacuum. The resulting crude material was purified by column chromatography on SiO_2 to afford the corresponding glycal boronates.

General procedure for the preparation of dipeptides and tripeptides

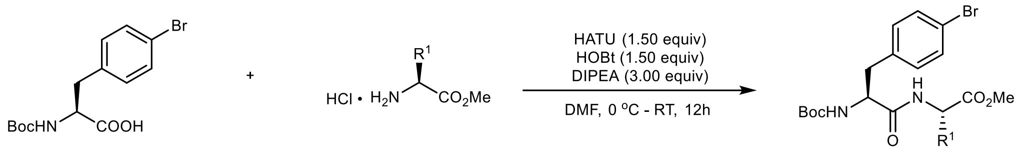

16s-1 **16s-2** **16-A**

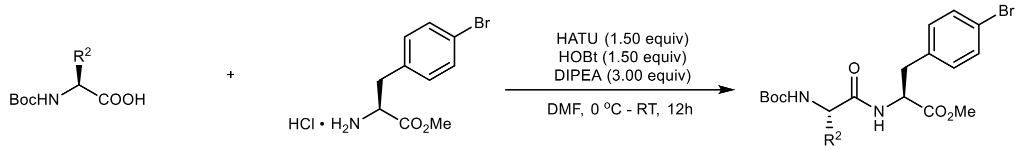

16s-3 **16s-4** **16-B**

Procedure F: Under N_2, a solution of carboxylic acid **16s-1/16s-3** (1.00 equiv) in DMF (0.2 M) was treated with HATU (1.50 equiv), and HOBt (1.50 equiv), followed by the addition of DIPEA (3.00 equiv). The reaction mixture was stirred at 0 °C for 0.5 hours, after which amino acid hydrochloride **16s-2**/**16s-4** (1.20 equiv) was added. After stirring at room temperature for another 12 hours, the crude material was quenched with water, extracted with EtOAc (3×), washed with 1.0 M HCl solution (2×), sat. aq. $NaHCO_3$ solution (2×), and brine (2×). The organic layers were then dried over Na_2SO_4, filtered, and concentrated under reduced pressure. The residue was purified by chromatographic purification on SiO_2.

General procedure for the preparation of dipeptides and tripeptides

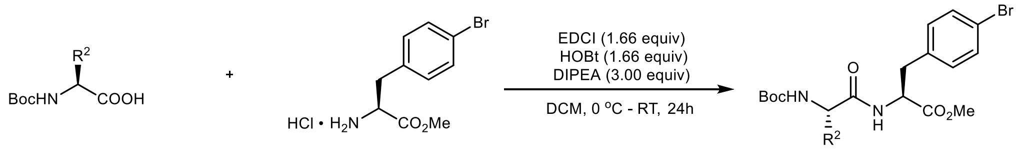

16s-3 **16s-4** **16-B**

Procedure G: In an ice-salt bath, a solution of carboxylic acid (1.00 equiv), amino acid hydrochloride (1.00 equiv), and HOBt (1.66 equiv) in dry DCM was added DIPEA (3.00 equiv). The reaction mixture was stirred at 0 °C, and a solution of EDCI (1.66 equiv) in dry DCM was added dropwise. The solution was then stirred at room temperature for 24 h. Upon completion of the reaction, a significant amount of solid formed. The mixture was filtered, and the solid was washed successively with saturated NH_4Cl solution, 1N HCl solution, and sat. aq. $NaHCO_3$ solution. The crude product obtained was recrystallized from CH_3OH to afford the desired compound.

2.2. Detailed experimental procedures for preparation of start materials

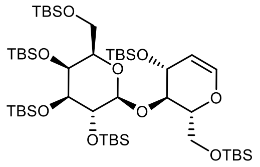

18k

(((2*S*,3*R*,4*S*,5*S*,6*R*)-2-(((2*R*,3*R*,4*R*)-4-((*tert*-Butyldimethylsilyl)oxy)-2-(((*tert*-butyldimethylsilyl)oxy)methyl)-3,4-dihydro-2*H*-pyran-3-yl)oxy)-6-(((*tert*-butyldimethylsilyl)oxy)methyl)tetrahydro-2*H*-pyran-3,4,5-triyl)tris(oxy))tris(*tert*-butyldimethylsilane) (18k). According to the general procedure D, a solution of the fully acetylated protected disaccharide glycals **18k-s**[3] (3.92 g, 7.00 mmol, 1.00 equiv) in anhydrous MeOH (30.0 mL) at room temperature was treated with NaOMe (126 mg, 0.70 mmol, 0.10 equiv). The reaction mixture was stirred for 2 h, concentrated, and azeotroped with toluene (2×). Subsequently, the crude underivatized disaccharides glycals were transferred to a dry flask under N_2. DDry DCM (100 mL) was added, and the solution was stirred until most of the sugar dissolved. Dry 2,6-lutidine (15.0 g, 140 mmol, 20.0 equiv) was added, followed by *tert*-butyl-dimethyl triflate (16.6 g, 63.0 mmol, 9.00 equiv). Upon addition of the triflate, the previously undissolved sugar dissolved completely, and the reaction mixture became clear. The reaction mixture was stirred at room temperature for 48 h. The product was extracted with ether and washed with water. The aqueous layer was then extracted with ether. The product in the combined ether extracts was rotary evaporated, and the crude mixture was purified by chromatographic purification on SiO_2 (Petroleum ether:Ether, 1:0-10:1) to afford **18k** (4.30 g, 62%) as a white foam: **^{1}H NMR** (400 MHz, $CDCl_3$) δ 6.28 (d, *J* = 6.0 Hz, 1H), 4.63 (dd, *J* = 6.1, 2.7 Hz, 1H), 4.51 (d, *J* = 7.3 Hz, 1H), 4.24 – 4.22 (m, 1H), 4.15 (dd, *J* = 11.6, 3.0 Hz, 1H), 4.08 (d, *J* = 1.8 Hz, 1H), 3.93 (dd, *J* = 8.4, 6.1 Hz, 1H), 3.87 (dd, *J* = 9.3, 7.3 Hz, 1H), 3.80 – 3.68 (m, 3H), 3.59 – 3.57 (m, 1H), 3.49 (dd, *J* = 9.3, 2.0 Hz, 1H), 3.26 – 3.23 (m, 1H), 0.93 – 0.92 (m, 18H), 0.89 – 0.89 (m, 27H), 0.87 (s, 9H), 0.15 (s, 3H), 0.13 – 0.12 (m, 9H), 0.09 – 0.08 (m, 12H), 0.06 (s, 3H), 0.04 (s, 3H), 0.02 (s, 3H), 0.01 (s, 3H); **^{13}C NMR** (101 MHz, $CDCl_3$) δ 143.8, 103.5, 102.6, 77.9, 75.5, 73.7, 72.1, 71.4, 68.3, 61.0, 60.2, 27.0, 26.7, 26.3, 26.2, 26.1, 25.9, 19.3, 18.8, 18.6, 18.5, 18.4, 18.2, -2.9, -3.3, -3.6, -3.7, -4.0, -4.2, -4.7, -5.0, -5.2(2), -5.3; **HRMS** (ESI) *m/z* calcd for $C_{48}H_{104}O_9Si_6Na$ $[M + Na]^+$ 1015.6194, found 1015.6210.

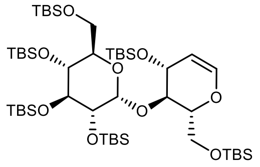

18l

(((2*R*,3*R*,4*S*,5*R*,6*R*)-2-(((2*R*,3*R*,4*R*)-4-((*tert*-Butyldimethylsilyl)oxy)-2-(((*tert*-butyldimethylsilyl)oxy)methyl)-3,4-dihydro-2*H*-pyran-3-yl)oxy)-6-(((*tert*-butyldimethylsilyl)oxy)methyl)tetrahydro-2*H*-pyran-3,4,5-triyl)tris(oxy))tris(*tert*-butyldimethylsilane) (18l). According to the general procedure D, a solution of the

full acetyl protected disaccharide glycals **18l-s**[3] (3.40 g, 6.00 mmol, 1.00 equiv) in anhydrous MeOH (30.0 mL) at room temperature was treated with NaOMe (54.0 mg, 0.30 mmol, 0.05 equiv). The reaction mixture was stirred for 2 h, concentrated, and azeotroped with toluene (2×). The crude underivatized disaccharides glycals was introduced in a dry flask under N_2. Dry DCM (100 mL) was added and the solution stirred until most of the sugar dissolved. Dry 2,6-lutidine (12.8 g, 120 mmol, 20.0 equiv) was added, followed by *tert*-butyl-dimethyl triflate (14.3 g, 54.0 mmol, 9.00 equiv). On addition of the triflate the undissolved sugar dissolved completely and the reaction mixture became clear. The reaction mixture was stirred at room temperature for 48 h. The product was extracted with ether and washed with water. The aqueous layer was then extracted with ether. The product in the combined ether extracts was rotary evaporated and the crude mixture was purified by chromatographic purification on SiO_2 (Petroleum ether:Ether, 1:0-10:1) to afford **18l** (3.10 g, 52%) as a light-colorless foam: **^{1}H NMR** (400 MHz, $CDCl_3$) δ 6.32 (d, *J* = 6.2 Hz, 1H), 4.95 (d, *J* = 3.1 Hz, 1H), 4.66 (dd, *J* = 6.2, 3.9 Hz, 1H), 4.18 – 4.13 (m, 1H), 4.02 (t, *J* = 4.0, 4.0 Hz, 1H), 3.96 – 3.87 (m, 3H), 3.84 – 3.79 (m, 4H), 3.76 – 3.73 (m, 2H), 0.89 – 0.87 (m, 54H), 0.10 – 0.03 (m, 36H); **^{13}C NMR** (126 MHz, $CDCl_3$) δ 143.6, 101.8, 95.1, 79.3, 76.8, 74.8, 73.4, 71.6, 71.5, 65.5, 63.0, 62.4, 26.2, 26.1, 26.0(2), 25.9, 18.7, 18.5, 18.4, 18.2, 18.1, 18.0, -3.7, -4.0, -4.3, -4.4, -4.6(2), -4.8, -5.0, -5.1(2), -5.2; **HRMS** (ESI) *m/z* calcd for $C_{48}H_{104}O_9Si_6Na$ $[M + Na]^+$ 1015.6194, found 1015.6210.

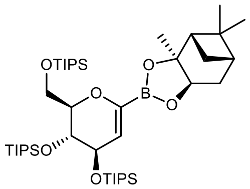

12b

(((2*R*,3*R*,4*R*)-2-(((Triisopropylsilyl)oxy)methyl)-6-((3a*S*,4*S*,6*S*,7a*R*)-3a,5,5-trimethylhexahydro-4,6-methanobenzo[*d*][1,3,2]dioxaborol-2-yl)-3,4-dihydro-2*H*-pyran-3,4-diyl)bis(oxy))bis(triisopropylsilane) (12b). According to the general procedure E,$[Ir(OMe)(cod)]_2$ (13.4 mg, 0.02 mmol, 1.00 mol%), dtbbpy (10.8 mg, 0.04 mmol, 2.00 mol%), and $B_2(pai)_2$ (716 mg, 2.00 mmol, 1.00 equiv) were added to a Schlenk flask, which was then flushed with nitrogen. *n*-Octane (8.00 mL) and 3,4,6-*tris*-*O*-(triisopropylsilyl)-D-glucal[4] (1.84 g, 3.00 mmol, 1.50 equiv) were added, and the mixture was stirred at 80 °C for 24 h. The reaction mixture was then cooled to rt andconcentrated under vaccum. The crude material was purified by column chromatography on SiO_2 (Petroleum ether:Ether, 1:0-30:1) to afford **12b** (1.10 g, 70%) as a colorless oil: **^{1}H NMR** (400 MHz, $CDCl_3$) δ 5.60 (dd, *J* = 5.3, 1.8 Hz, 1H), 4.35 – 4.28 (m, 2H), 4.20 – 4.18 (m, 1H), 3.98 – 3.92 (m, 3H), 2.37 – 2.30 (m, 1H), 2.23 – 2.17 (m, 1H), 2.11 – 2.08 (m, 1H), 1.94 – 1.88 (m, 2H), 1.41 (s, 3H), 1.28 (s, 3H), 1.16 (d, *J* = 11.0 Hz, 1H), 1.08 – 1.03 (m, 63H), 0.84 (s, 3H); **^{13}C NMR** (101 MHz, $CDCl_3$) δ 114.0,86.6, 79.7, 78.4, 69.8, 65.2, 61.8, 51.4, 39.6, 38.2, 35.5, 28.6, 27.2, 26.6, 24.2, 18.4, 18.3(3), 18.2, 12.7, 12.6, 12.2; **HRMS** (ESI) *m/z* calcd for $C_{43}H_{85}BO_6Si_3Na$ $[M + Na]^+$ 815.5639, found 815.5650.

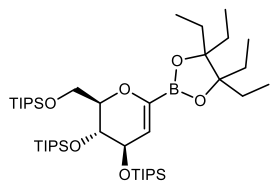

12c

(((2*R*,3*R*,4*R*)-6-(4,4,5,5-Tetraethyl-1,3,2-dioxaborolan-2-yl)-2-(((triisopropylsilyl)oxy)methyl)-3,4-dihydro-2*H*-pyran-3,4-diyl)bis(oxy))bis(triisopropylsilane) (12c). According to the general procedure E, $[Ir(OMe)(cod)]_2$ (13.4 mg, 0.02 mmol, 1.00 mol%), dtbbpy (10.8 mg, 0.04 mmol, 2.00 mol%), and $B_2(Epin)_2$ (732 mg, 2.00 mmol, 1.00 equiv) were added to a Schlenk flask, which was then flushed with nitrogen. *n*-Octane (8.00 mL) and 3,4,6-tris-*O*-(triisopropylsilyl)-D-glucal[4] (1.84 g, 3.00 mmol, 1.50 equiv) were added, and the mixture was stirred at 80 °C for 24 h. The reaction mixture cooled to rt and concentrated under vaccum. The crude material was purified by column chromatography on SiO_2 (Petroleum ether:Ether, 1:0-50:1) to afford **12c** (1.43g, 90%) as a colorless oil: **^{1}H NMR** (400 MHz, $CDCl_3$) δ 5.60 (dd, *J* = 5.3, 1.7 Hz, 1H), 4.30 – 4.26 (m, 1H), 4.12 – 4.11 (m, 1H), 3.98 – 3.93 (m, 2H), 3.85 (dd, *J* = 10.8, 5.2 Hz, 1H), 1.69 – 1.61 (m, 8H), 1.08 – 1.03 (m, 63H), 0.91 (t, *J* = 7.4, 7.4 Hz, 12H); **^{13}C NMR** (101 MHz, $CDCl_3$) δ 113.9, 89.0, 79.8, 69.9, 65.2, 62.0, 26.7, 26.4, 18.3(3), 18.2(2), 12.7, 12.6, 12.2, 9.1, 8.7; **HRMS** (ESI) *m/z* calcd for $C_{43}H_{89}BO_6Si_3Na$ $[M + Na]^+$ 819.5952, found 819.5961.

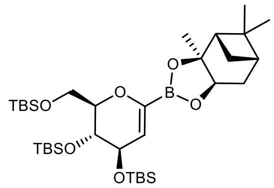

12e

(((2*R*,3*R*,4*R*)-2-(((*tert*-butyldimethylsilyl)oxy)methyl)-6-((3a*S*,4*S*,6*S*,7a*R*)-3a,5,5-trimethylhexahydro-4,6-methanobenzo[*d*][1,3,2]dioxaborol-2-yl)-3,4-dihydro-2*H*-pyran-3,4-diyl)bis(oxy))bis(*tert*-butyldimethylsilane) (12e). According to the general procedure E, $[Ir(OMe)(cod)]_2$ (26.8 mg, 0.04 mmol, 1.00 mol%), dtbbpy (21.6 mg, 0.08 mmol, 4.00 mol%), and $B_2(pai)_2$ (1.43 g, 4.00 mmol, 1.00 equiv) were added to a Schlenk flask, which was then flushed with nitrogen. *n*-Octane (16.0 mL) and 3,4,6-tris-*O*-(*tert*-butyldimethylsily)-D-glucal[4] (2.93 g, 1.00 mmol, 1.50 equiv) were added,and the mixture was stirred at 80 °C for 24 h. The reaction mixture cooled to rt and concentrated under vaccum. The crude material was purified by column chromatography on SiO_2 (Petroleum ether:Ether, 1:0-50:1) to afford **12e** (1.60 g, 60%) as a colorless oil: **^{1}H NMR** (400 MHz, $CDCl_3$) δ 5.48 – 5.46 (m, 1H), 4.34 (dd, *J* = 8.8, 1.8 Hz, 1H), 4.05 – 4.01 (m, 1H), 3.89 (dd, *J* = 3.3, 2.3 Hz, 2H), 3.83 (d, *J* = 5.9 Hz, 2H), 2.36 – 2.29 (m, 1H), 2.24 – 2.17 (m, 1H), 2.08 (t, *J* = 5.4, 5.4 Hz, 1H), 1.96 – 1.88 (m, 2H), 1.42 (s, 3H), 1.28 (s, 3H), 1.18 (d, *J* = 11.0 Hz, 1H), 0.90 – 0.86 (m, 27H), 0.84 (s, 3H), 0.09 – 0.08 (m, 12H), 0.04 (s, 3H), 0.02 (s, 3H); **^{13}C NMR** (101 MHz,

$CDCl_3$) δ 115.0, 86.7, 79.3, 78.5, 69.8, 66.8, 61.3, 51.3, 39.6, 38.2, 35.4, 28.6, 27.2, 26.6, 26.1(3), 24.2, 18.5, 18.3, 18.2, -4.1, -4.2(2), -4.5, -5.1; **HRMS** (ESI) *m/z* calcd for $C_{34}H_{67}BO_6Si_3Na$ $[M + Na]^+$ 689.4231, found 689.4249.

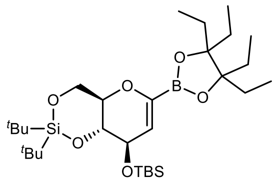

12f

(4a*R*,8*R*,8a*R*)-2,2-Di-*tert*-butyl-8-((*tert*-butyldimethylsilyl)oxy)-6-(4,4,5,5-tetraethyl-1,3,2-dioxaborolan-2-yl)-4,4a,8,8a-tetrahydropyrano[3,2-*d*][1,3,2]dioxasiline (12f). According to the general procedure E, $[Ir(OMe)(cod)]_2$ (6.70 mg, 0.01 mmol, 1.00 mol%), dtbbpy (5.40 mg, 0.02 mmol, 2.00 mol%), and $B_2(Epin)_2$ (366 mg, 1.00 mmol, 1.00 equiv) were added to a Schlenk flask, which was then flushed with nitrogen.*n*-Octane (4.00 mL) and 4,6-*O*-bis(*tert*-butylsilylidene)-3-*O*-(*tert*-butyldimethylsily)-D-glucal[5] (600 mg, 1.50 mmol, 1.50 equiv) were added, and the mixture was stirred at 80 °C for 24 h. The reaction mixture cooled to rt and concentrated under vaccum. The crude material was purified by column chromatography on SiO_2 (Petroleum ether:Ether, 1:0-30:1) to afford **12f** (460 mg, 79%) as a white foam: **^{1}H NMR** (500 MHz, Acetone-d_6) δ 5.35 (d, J = 2.1 Hz, 1H), 4.37 (dd, J = 7.2, 2.1 Hz, 1H), 4.16 (dd, J = 10.3, 5.0 Hz, 1H), 3.97 – 3.88 (m, 2H), 3.83 – 3.78 (m, 1H), 1.73 – 1.67 (m, 8H), 1.08 (s, 9H), 1.02 (s, 9H), 0.94 (s, 9H), 0.92 (s, 3H), 0.91 (s, 6H), 0.89 (s, 3H), 0.18 (s, 3H), 0.16 (s, 3H); **^{13}C NMR** (126 MHz, Acetone-d_6) δ 119.7, 89.9, 78.0, 73.5, 72.0, 66.8, 27.9, 27.4, 27.0, 26.9, 26.2, 23.3, 20.4, 18.7, 9.1, -4.0, -4.4; **HRMS** (ESI) *m/z* calcd for $C_{30}H_{60}BO_6Si_2$ $[M + H]^+$ 583.4016, found 583.4026.

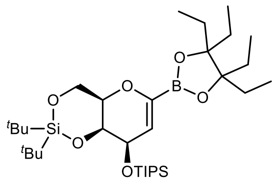

12g

(4a*R*,8*R*,8a*S*)-2,2-Di-*tert*-butyl-6-(4,4,5,5-tetraethyl-1,3,2-dioxaborolan-2-yl)-8-((triisopropylsilyl)oxy)-4,4a,8,8a-tetrahydropyrano[3,2-*d*][1,3,2]dioxasiline (12g). According to the general procedure E, $[Ir(OMe)(cod)]_2$ (13.4 mg, 0.02 mmol, 1.00 mol%), dtbbpy (10.8 mg, 0.04 mmol, 2.00 mol%), and $B_2(Epin)_2$ (732 mg, 2.00 mmol, 1.00 equiv) were added to a Schlenk flask, which was then flushed with nitrogen. *n*-Octane (8.00 mL) and 4,6-*O*-bis(*tert*-butylsilylidene)-3-*O*-triisopropylsilyl-D-galactal[4] (1.33 g, 3.00 mmol, 1.50 equiv) were added, and the mixture was stirred at 80 °C for 24 h. The reaction mixture cooled to rt and concentrated under vaccum. The crude material was purified by column chromatography on SiO_2 (Petroleum ether:Ether, 1:0-20:1) to afford **12g** (899 mg, 72%) as a syrup: **^{1}H NMR** (500 MHz, Acetone-d_6) δ 5.44 – 5.44 (m, 1H), 4.73 – 4.71 (m, 1H), 4.51 – 4.49 (m, 1H), 4.29 – 4.20 (m, 2H), 3.97 –

3.96 (m, 1H), 1.73 – 1.64 (m, 8H), 1.16 – 1.10 (m, 21H), 1.08 (s, 9H), 1.00 (s, 9H), 0.93 – 0.90 (m, 12H); **^{13}C NMR** (126 MHz, Acetone-d_6) δ 118.3, 89.5, 74.1, 70.1, 68.3, 67.2, 28.1, 27.8, 27.0, 26.7, 24.0, 21.5, 18.5(2), 13.2, 9.2, 8.9; **HRMS** (ESI) m/z calcd for $C_{33}H_{65}BO_6Si_2Na$ [M + Na]$^+$ 647.4305, found 647.4327.

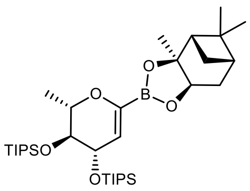

12h-1

(((2*S*,3*S*,4*S*)-2-Methyl-6-((3a*S*,4*S*,6*S*,7a*R*)-3a,5,5-trimethylhexahydro-4,6-methanobenzo[*d*][1,3,2]dioxaborol-2-yl)-3,4-dihydro-2*H*-pyran-3,4-diyl)bis(oxy))bis(triisopropylsilane) (12h-1). According to the general procedure E, $[Ir(OMe)(cod)]_2$ (26.8 mg, 0.04 mmol, 1.00 mol%), dtbbpy (21.6 mg, 0.08 mmol, 2.00 mol%), and $B_2(pai)_2$ (1.43 g, 4.00 mmol, 1.00 equiv) were added to a Schlenk flask, which was then flushed with nitrogen. *n*-Octane (16.0 mL) and 3,4-di-*O*-triisopropylsilyl-L-rhamnal[4] (2.65 g, 6.00 mmol, 1.50 equiv) were added, and the mixture was stirred at 80 °C for 24 h.The reaction mixture cooled to rt and concentrated under vaccum. The crude material was purified by column chromatography on SiO_2 (Petroleum ether:Ether, 1:0-50:1) toafford **12h-1** (1.75 g, 71%) as a colorless oil: **^{1}H NMR** (400 MHz, $CDCl_3$) δ 5.59 (dd, J = 5.0, 1.6 Hz, 1H), 4.37 – 4.27 (m, 2H), 4.02 – 3.99 (m, 1H), 3.92 – 3.90 (m, 1H), 2.36 – 2.29 (m, 1H), 2.22 – 2.15 (m, 1H), 2.10 (t, J = 5.5, 5.5 Hz, 1H), 1.96 – 1.88 (m, 2H), 1.44 (s, 3H), 1.35 (d, J = 7.1 Hz, 3H), 1.28 (s, 3H), 1.15 (d, 1H) 1.09 – 1.02 (m, 42H), 0.84 (s, 3H); **^{13}C NMR** (101 MHz, $CDCl_3$) δ 114.1, 86.7, 78.5, 74.1, 73.5, 66.1, 51.3, 39.6, 38.2, 35.4, 28.6, 27.2, 26.3, 24.2, 18.3(2), 18.2, 16.1, 12.8, 12.6; **HRMS** (ESI) *m/z* calcd for $C_{34}H_{66}BO_5Si_2$ [M + H]$^+$ 621.4536, found 621.4559.

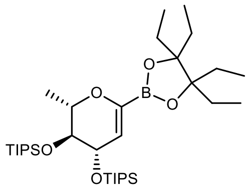

12h-2

(((2*S*,3*S*,4*S*)-2-Methyl-6-(4,4,5,5-tetraethyl-1,3,2-dioxaborolan-2-yl)-3,4-dihydro-2*H*-pyran-3,4-diyl)bis(oxy))bis(triisopropylsilane) (12h-2). According to the general procedure E, $[Ir(OMe)(cod)]_2$ (19.9 mg, 0.03 mmol, 1.00 mol%), dtbbpy (16.1 mg, 0.06 mmol, 2.00 mol%), and $B_2(Epin)_2$ (1.10 g, 3.00 mmol, 1.00 equiv) were added to a Schlenk flask, which was then flushed with nitrogen. *n*-Octane (12.0 mL) and 3,4-di-*O*-triisopropylsilyl-L-rhamnal[4] (1.99 g, 4.50 mmol, 1.50 equiv) were added, and the mixture was stirred at 80 °C for 24 h. The reaction mixture cooled to rt and concentrated under vaccum. The crude material was purified by column chromatography on SiO_2 (Petroleum ether:Ether, 1:0-50:1) to afford **12h-2** (1.40 g, 75%) as a colorless oil: **^{1}H**

NMR (500 MHz, Acetone-d_6) δ 5.63 (dd, J = 4.9, 1.6 Hz, 1H), 4.26 – 4.21 (m, 1H), 4.09 – 4.07 (m, 1H), 4.00 – 3.99 (m, 1H), 1.79 – 1.64 (m, 8H), 1.35 (d, J = 7.0 Hz, 3H), 1.16 – 1.06 (m, 42H), 0.95 – 0.91 (m, 12H); **^{13}C NMR** (126 MHz, Acetone-d_6) δ 114.3, 89.5, 74.4, 74.3, 67.3, 27.3, 26.9, 18.6(2), 18.5(2), 16.6, 13.4, 13.4, 9.3, 8.9; **HRMS** (ESI) *m/z* calcd for $C_{34}H_{69}BO_5Si_2Na$ [M + Na]$^+$ 647.4674, found 647.4684.

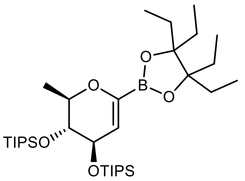

12i

(((2*R*,3*R*,4*R*)-2-Methyl-6-(4,4,5,5-tetraethyl-1,3,2-dioxaborolan-2-yl)-3,4-dihydro-2*H*-pyran-3,4-diyl)bis(oxy))bis(triisopropylsilane) (12i). According to the general procedure E, $[Ir(OMe)(cod)]_2$ (13.4 mg, 0.02 mmol, 1.00 mol%), dtbbpy (10.8 mg, 0.04 mmol, 2.00 mol%), and $B_2(Epin)_2$ (732 mg, 2.00 mmol, 1.00 equiv) were added to a Schlenk flask, which was then flushed with nitrogen. *n*-Octane (8.00 mL) and 3,4-di-*O*-(triisopropylsilyl)-6-deoxy-D-glucal[6] (1.32 g, 3.00 mmol, 1.50 equiv) were added, and the mixture was stirred at 80 °C for 24 h. The reaction mixture cooled to rt and concentrated under vaccum. The crude material was purified by column chromatography on SiO_2 (Petroleum ether:Ether, 1:0-30:1) to afford **12i** (792 mg, 63%) as a colorless oil: **^{1}H NMR** (400 MHz, Acetone-d_6) δ 5.63 (dd, J = 4.9, 1.7 Hz, 1H), 4.27 – 4.21 (m, 1H), 4.09 – 4.07 (m, 1H), 4.01 – 3.99 (m, 1H), 1.78 – 1.64 (m, 8H), 1.34 (d, J = 7.0 Hz, 3H), 1.17 – 1.06 (m, 42H), 0.95 – 0.90 (m, 12H); **^{13}C NMR** (101 MHz, Acetone-d_6) δ 114.4, 89.5, 74.4(2), 67.3, 27.3, 26.9, 18.6(2), 18.5(2), 16.6, 13.4, 13.4, 9.2, 8.9; **HRMS** (ESI) *m/z* calcd for $C_{34}H_{69}BO_5Si_2Na$ [M + Na]$^+$ 647.4674, found 647.4678.

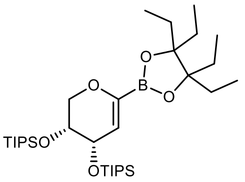

12j

(((3*R*,4*S*)-6-(4,4,5,5-Tetraethyl-1,3,2-dioxaborolan-2-yl)-3,4-dihydro-2*H*-pyran-3,4-diyl)bis(oxy))bis(triisopropylsilane) (12j). According to the general procedure E, $[Ir(OMe)(cod)]_2$ (13.4 mg, 0.02 mmol, 1.00 mol%), dtbbpy (10.8 mg, 0.04 mmol, 2.00 mol%), and $B_2(Epin)_2$ (732 mg, 2.00 mmol, 1.00 equiv) were added to a Schlenk flask,which was then flushed with nitrogen. *n*-Octane (8.00 mL) and 3,4,-di-*O*-(triisopropylsilyl)-D-arabinal[7] (1.28 g, 3.00 mmol, 1.50 equiv) were added, and the mixture was stirred at 80 °C for 24 h. The reaction mixture cooled to rt and concentrated under vaccum. The crude material was purified by column chromatography on SiO_2 (Petroleum ether:Ether, 1:0–50:1) to afford **12j** (894 mg, 85%) as a colorless oil: **^{1}H NMR** (500 MHz, Acetone-d_6) δ 5.64 (d, J = 5.5 Hz, 1H), 4.35 – 4.33 (m, 1H), 4.05 –

4.01 (m, 1H), 3.94 (t, *J* = 10.2, 10.2 Hz, 1H), 3.83 – 3.80 (m, 1H), 1.75 – 1.64 (m, 8H), 1.18 – 1.07 (m, 42H), 0.94 – 0.90 (m, 12H); **^{13}C NMR** (126 MHz, Acetone-*d*$_6$) δ 116.2, 89.7, 69.8, 65.4, 65.1, 27.2, 26.8, 18.7, 18.6, 18.5(2), 13.6, 13.2, 9.2, 8.9; **HRMS** (ESI) *m/z* calcd for $C_{33}H_{67}BO_5Si_2Na$ [M + Na]$^+$ 633.4512, found 633.4520.

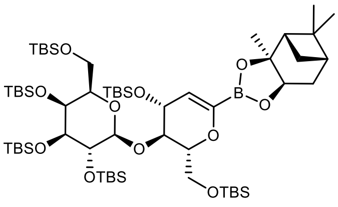

12k

(((2*S*,3*R*,4*S*,5*S*,6*R*)-2-(((2*R*,3*R*,4*R*)-4-((*tert*-Butyldimethylsilyl)oxy)-2-(((*tert*-butyldimethylsilyl)oxy)methyl)-6-((3a*S*,4*S*,6*S*,7a*R*)-3a,5,5-trimethylhexahydro-4,6-methanobenzo[*d*][1,3,2]dioxaborol-2-yl)-3,4-dihydro-2*H*-pyran-3-yl)oxy)-6-(((*tert*-butyldimethylsilyl)oxy)methyl)tetrahydro-2*H*-pyran-3,4,5-triyl)tris(oxy))tris(*tert*-butyldimethylsilane) (12k). According to the general procedure E, [Ir(OMe)(cod)]$_2$ (9.30 mg, 0.014 mmol, 2.00 mol%), dtbbpy (7.50 mg, 0.028 mmol, 4.00 mol%), and $B_2(Epin)_2$ (251 mg, 0.70 mmol, 1.00 equiv) were added to a Schlenk flask, which was then flushed with nitrogen. *n*-Octane (4.00 mL) and (((2*S*,3*R*,4*S*,5*S*,6*R*)-2-(((2*R*,3*R*,4*R*)-4-((*tert*-butyldimethylsilyl)oxy)-2-(((*tert*-butyldimethylsilyl)oxy)methyl)-3,4-dihydro-2*H*-pyran-3-yl)oxy)-6-(((*tert*-butyldimethylsilyl)oxy)methyl)tetrahydro-2*H*-pyran-3,4,5-triyl)tris(oxy))tris(*tert*-butyldimethylsilane) (1.04 g, 1.05 mmol, 1.50 equiv) were added, and the mixture was stirred at 80 °C for 24 h. The reaction mixture cooled to rt and concentrated under vaccum. The crude material was purified by column chromatography on SiO_2 (Petroleum ether:Ether, 1:0–30:1) to afford **12k** (605 mg, 74%) as a white foam: **^{1}H NMR** (400 MHz, Acetone-*d*$_6$) δ 5.36 (d, *J* = 2.8 Hz, 1H), 4.66 (d, *J* = 7.4 Hz, 1H), 4.40 (dd, *J* = 8.7, 1.8 Hz, 1H), 4.28 (dd, *J* = 6.1, 2.9 Hz, 1H), 4.22 – 4.18 (m, 2H), 3.97 – 3.76 (m, 6H), 3.64 (dd, *J* = 9.2, 2.1 Hz, 2H), 3.41 (dd, *J* = 9.6, 5.0 Hz, 1H), 2.42 – 2.34 (m, 1H), 2.25 – 2.19 (m, 1H), 2.04 – 2.02 (m, 1H), 1.94 – 1.89 (m, 1H), 1.86 – 1.81 (m, 1H), 1.39 (s, 3H), 1.30 (s, 3H), 0.99 – 0.92 (m, 54H), 0.88 (s, 3H), 0.23 (s, 3H), 0.21 (s, 3H), 0.19 (s, 3H), 0.17 – 0.13 (m, 18H), 0.09 (s, 9H); **^{13}C NMR** (101 MHz, Acetone-*d*$_6$) δ 117.7, 103.3, 87.0, 78.8, 78.1, 77.7, 76.1, 74.3, 72.8, 72.4, 68.7, 61.9, 61.1, 52.1, 40.3, 38.8, 36.0, 28.9, 27.4, 27.3, 27.2, 27.0, 26.7, 26.5(2), 26.2, 24.2, 19.8, 19.3, 19.0, 18.9, 18.8, 18.7, -2.5, -2.9, -3.1, -3.4, -3.6, -3.7, -3.9, -4.4, -4.6, -5.0, -5.1, -5.2; **HRMS** (ESI) m/z calcd for $C_{58}H_{119}BO_{11}Si_6Na$ [M + Na]$^+$ 1193.7353, found 1193.7344.

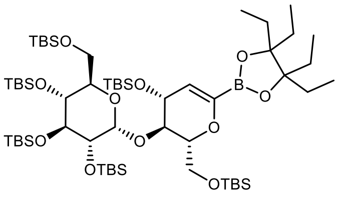

12l

(((2*R*,3*R*,4*S*,5*R*,6*R*)-2-(((2*R*,3*R*,4*R*)-4-((*tert*-Butyldimethylsilyl)oxy)-2-(((*tert*-butyldimethylsilyl)oxy)methyl)-6-(4,4,5,5-tetraethyl-1,3,2-dioxaborolan-2-yl)-3,4-dihydro-2*H*-pyran-3-yl)oxy)-6-(((*tert*-butyldimethylsilyl)oxy)methyl)tetrahydro-2*H*-pyran-3,4,5-triyl)tris(oxy))tris(*tert*-butyldimethylsilane) (12l). According to the general procedure E, [Ir(OMe)(cod)]$_2$ (9.30 mg, 0.014 mmol, 2.00 mol%), dtbbpy (7.50 mg, 0.028 mmol, 4.0 mol%), and B_2(Epin)$_2$ (251 mg, 0.70 mmol, 1.00 equiv) were added to a Schlenk flask, which was then flushed with nitrogen. *n*-Octane (4.00 mL) and (((2*R*,3*R*,4*S*,5*R*,6*R*)-2-(((2*R*,3*R*,4*R*)-4-((*tert*-butyldimethylsilyl)oxy)-2-(((*tert*-butyldimethylsilyl)oxy)methyl)-3,4-dihydro-2*H*-pyran-3-yl)oxy)-6-(((*tert*-butyldimethylsilyl)oxy)methyl)tetrahydro-2*H*-pyran-3,4,5-triyl)tris(oxy))tris(*tert*-butyldimethylsilane) (1.04 g, 1.05 mmol, 1.50 equiv) were added, and the mixture was stirred at 80 °C for 24 h. The reaction mixture cooled to rt and concentrated under vaccum. The crude material was purified by column chromatography on SiO_2 (Petroleum ether:Ether, 1:0–50:1) to afford **12l** (616 mg, 75%) as a syrup: **^{1}H NMR** (500 MHz, Acetone-*d*$_6$) δ 5.47 (dd, *J* = 3.9, 0.9 Hz, 1H), 5.08 (d, *J* = 3.5 Hz, 1H), 4.17 – 4.15 (m, 1H), 4.11 – 4.08 (m, 1H), 4.00 (d, *J* = 2.6 Hz, 2H), 3.95 – 3.90 (m, 5H), 3.88 – 3.86 (m, 2H), 1.76 – 1.67 (m, 8H), 0.95 – 0.90 (m, 66H), 0.18 – 0.08 (m, 36H); **^{13}C NMR** (126 MHz, Acetone-*d*$_6$) δ 115.8, 95.6, 89.6, 79.4, 77.7, 75.5, 73.7, 72.5, 72.2, 66.9, 63.7, 63.0, 27.0, 26.8, 26.5, 26.4(3), 26.3(2), 19.1, 19.0, 18.9, 18.6, 18.5, 9.2, 9.0, -3.5, -3.7, -4.1(2), -4.3(2), -4.5, -4.7, -4.8, -4.9, -5.0; **HRMS** (ESI) m/z calcd for $C_{58}H_{123}BO_{11}Si_6Na$ [M + Na]$^+$ 1197.7666, found 1197.7679.

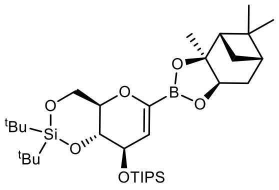

12m

(4a*R*,8*R*,8a*R*)-2,2-Di-*tert*-butyl-8-((triisopropylsilyl)oxy)-6-((3a*S*,4*S*,6*S*,7a*R*)-3a,5,5-trimethylhexahydro-4,6-methanobenzo[*d*][1,3,2]dioxaborol-2-yl)-4,4a,8,8a-tetrahydropyrano[3,2-*d*][1,3,2]dioxasiline (12m). According to the general procedure E,[Ir(OMe)(cod)]$_2$ (26.8 mg, 0.04 mmol, 1.00 mol%), dtbbpy (21.6 mg, 0.08 mmol, 2.00 mol%), and B_2(Epin)$_2$ (1.43 g, 4.00 mmol, 1.00 equiv) were added to a Schlenk flask, which was then flushed with nitrogen. *n*-Octane (16.00 mL) and 4,6-*O*-bis(*tert*-butylsilylidene)-3-*O*-triisopropylsilyl-D-glucal[8] (2.65 g, 6.00 mmol, 1.50 equiv) were added, and the mixture was stirred at 80 °C for 24 h. The reaction mixture cooled to rt andconcentrated under vaccum. The crude material was purified by column chromatography on SiO_2 (Petroleum ether:Ether, 1:0 – 30:1) to afford **12m** (2.08 g, 79%) as a white solid: **^{1}H NMR** (400 MHz, Acetone-*d*$_6$) δ 5.45 (d, *J* = 2.1 Hz, 1H), 4.52 (dd, *J* = 7.2, 2.1 Hz, 1H), 4.40 (dd, *J* = 8.7, 1.9 Hz, 1H), 4.16 (dd, *J* = 10.2, 5.0 Hz, 1H), 3.99 – 3.93 (m, 2H), 3.85 – 3.78 (m, 1H), 2.41 – 2.34 (m, 1H), 2.27 – 2.19 (m, 1H), 1.93 – 1.88 (m, 1H), 1.83 – 1.78 (m, 1H), 1.39 (s, 3H), 1.29 (s, 3H), 1.20 – 1.10 (m, 21H), 1.08 (s, 9H), 1.01 – 1.00 (m, 10H), 0.89 – 0.83 (m, 4H); **^{13}C NMR** (126 MHz, Acetone-*d*$_6$) δ 119.6, 87.3, 78.9, 78.4, 73.5, 72.2, 66.7, 52.0, 40.3, 38.8, 35.9, 28.8, 27.9,

27.4, 27.3, 27.0, 24.1, 23.3, 20.4, 18.6(2), 18.2, 13.3; **HRMS** (ESI) *m/z* calcd for $C_{33}H_{67}BO_5Si_2Na$ [M + Na]$^+$ 643.3992, found 643.3991

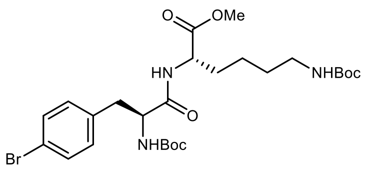

16e

Methyl N^2-((*S*)-3-(4-Bromophenyl)-2-((*tert*-butoxycarbonyl)amino)propanoyl)-N^6-(*tert*-butoxycarbonyl)-*L*-lysinate (16e). According to the general procedure F, under N_2, to a solution of (*S*)-3-(4-bromophenyl)-2-((*tert*-butoxycarbonyl)amino)propanoic acid (1.03 g, 3.00 mmol, 1.00 equiv) in DMF (15.0 mL) was added HATU (1.71 g, 4.50 mmol, 1.50 equiv), HOBT (608 mg, 4.50 mmol, 1.50 equiv), followed by the addition of DIPEA (1.16 g, 9.00 mmol, 3.00 equiv). After stirring at 0 °C for 0.5 h, the solution was added methyl N^6-(*tert*-butoxycarbonyl)-*L*-lysinate hydrochloride (1.07 g, 3.60 mmol, 1.20 equiv). After stirring at rt for another 12 hours, the crude material was quenched with water, extracted with EtOAc (3 × 30.0 mL), washed with 1.0 M HCl solution (2 × 60.0 mL), saturated $NaHCO_3$ solution (2 × 60.0 mL), and brine (2 × 60.0 mL). The organic layers were dried over Na_2SO_4, filtered, and concentrated under reduced pressure. The residue was purified by chromatographic purification on SiO_2 (Petroleum ether:Acetone, 2:1) to afford **16e** as a grayish-white powder (1.30 g, 74%): **^{1}H NMR** (400 MHz, $CDCl_3$) δ 7.37 (d, *J* = 8.2 Hz, 2H), 7.06 (d, *J* = 8.3 Hz, 2H), 6.70 (d, *J* = 7.7 Hz, 1H), 5.24 – 5.22 (m, 1H), 4.84 (s, 1H), 4.52 – 4.47 (m, 1H), 4.41 – 4.35 (m, 1H), 3.68 (s, 3H), 3.07 – 3.02 (m, 3H), 2.93 (dd, *J* = 13.9, 7.4 Hz, 1H), 1.82 – 1.72 (m, 1H), 1.66 – 1.57 (m, 1H), 1.46 – 1.41 (s, 11H), 1.37 (s, 9H), 1.27 – 1.20 (m, 3H); **^{13}C NMR** (101 MHz, $CDCl_3$) δ 172.3, 171.1, 156.1, 155.6, 135.8, 131.7, 131.2, 120.9, 80.4, 79.2, 55.5, 52.5, 52.0, 40.2, 38.7, 37.7, 32.0, 29.4, 28.5, 28.3, 22.4; **HRMS** (ESI) m/z calcd for $C_{26}H_{41}O_7N_3Br$ [M + H]+ 586.2122, found 586.2128.

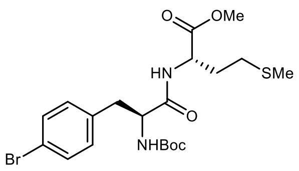

16f

Methyl ((*S*)-3-(4-Bromophenyl)-2-((*tert*-butoxycarbonyl)amino)propanoyl)-*L*-methioninate (16f). According to the general procedure F, under N_2, to a solution of (*S*)-3-(4-bromophenyl)-2-((*tert*-butoxycarbonyl)amino)propanoic acid (1.03 g, 3.00 mmol, 1.00 equiv) in DMF (15.0 mL) was added HATU (1.71 g, 4.50 mmol, 1.50 equiv), HOBT (608 mg, 4.50 mmol, 1.50 equiv), followed by the addition of DIPEA (1.16 g, 9.00 mmol, 3.00 equiv). After stirring at 0 °C for 0.5 h, the solution was added *L*-Methionine methyl ester hydrochloride (719 mg, 3.60 mmol, 1.20 equiv). After stirring at rt for another 12 hours, the crude material was quenched with water, extracted with

EtOAc (3 × 30.0 mL), washed with 1.0 M HCl solution (2 × 60.0 mL), saturated $NaHCO_3$ solution (2 × 60.0 mL), and brine (2 × 60.0 mL). The organic layers were dried over Na_2SO_4, filtered, and concentrated under reduced pressure. The residue was purified by chromatographic purification on SiO_2 (Petroleum ether:EtOAc:DCM, 1:0:0 – 6:2:0.5) to afford **16f** as a white powder (1.01 g, 69%): **^{1}H NMR** (400 MHz, $CDCl_3$) δ 7.42 – 7.40 (m, 2H), 7.08 – 7.06 (m, 2H), 6.60 (d, *J* = 7.7 Hz, 1H), 5.02 (d, *J* = 8.2 Hz, 1H), 4.65 – 4.60 (m, 1H), 4.35 – 4.30 (m, 1H), 3.72 (s, 3H), 3.01 (d, *J* = 6.8 Hz, 2H), 2.43 – 2.39 (m, 2H), 2.15 – 2.08 (m, 1H), 2.05 (s, 3H), 1.97 – 1.88 (m, 1H), 1.41 (s, 9H);**^{13}C NMR** (101 MHz, $CDCl_3$) δ 171.9, 170.9, 155.4, 135.6, 131.8, 131.2, 121.1, 80.6, 55.6, 52.7, 51.7, 37.6, 31.6, 29.8, 28.4, 15.5; **HRMS** (ESI) m/z calcd for $C_{20}H_{29}N_2O_5SBrNa$ [M + Na]+ 511.0873, found 511.0884.

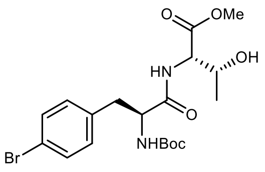

16g

Methyl ((*S*)-3-(4-bromophenyl)-2-((*tert*-butoxycarbonyl)amino)propanoyl)-*L*-threoninate (16g). According to the general procedure F, under N_2, to a solution of (*S*)-3-(4-bromophenyl)-2-((*tert*-butoxycarbonyl)amino)propanoic acid (1.03 g, 3.00 mmol, 1.00 equiv) in DMF (15.0 mL) was added HATU (1.71 g, 4.50 mmol, 1.50 equiv), HOBT (608 mg, 4.50 mmol, 1.50 equiv), followed by the addition of DIPEA (1.16 g, 9.00 mmol, 3.00 equiv). After stirring at 0 °C for 0.5 h, the solution was added *L*-threonine methyl ester hydrochloride (611 mg, 3.60 mmol, 1.20 equiv). After stirring at rt for another 12 hours, the crude material was quenched with water, extracted with EtOAc (3 × 30.0 mL), washed with 1.0 M HCl solution (2 × 60.0 mL), saturated $NaHCO_3$ solution (2 × 60.0 mL), and brine (2 × 60.0 mL). The organic layers were dried over Na_2SO_4, filtered, and concentrated under reduced pressure. The residue was purified by chromatographic purification on SiO_2 (Petroleum ether:Acetone, 1:0 – 2:1) to afford **16g** as a white foam (1.15 g, 83%): **^{1}H NMR** (500 MHz, $CDCl_3$) δ 7.41 – 7.38 (m, 2H), 7.10 – 7.08 (m, 2H), 6.85 (d, *J* = 8.9 Hz, 1H), 5.16 (d, *J* = 8.0 Hz, 1H), 4.56 (dd, *J* = 8.9, 2.7 Hz, 1H), 4.41 – 4.27 (m, 2H), 3.73 (s, 3H), 3.10 – 2.94 (m, 3H), 1.39 (s, 9H), 1.16 (d, *J* = 6.4 Hz, 3H); **^{13}C NMR** (126 MHz, $CDCl_3$) δ 171.7, 171.2, 155.7, 135.6, 131.7, 131.3, 121.0, 80.6, 68.3, 57.4, 55.7, 52.8, 37.6, 28.4, 19.9; **HRMS** (ESI) m/z calcd for $C_{19}H_{27}O_6N_2BrNa$ [M + Na]+ 481.0945, found 481.0946.

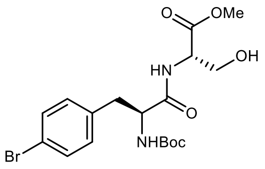

16h

Methyl ((*S*)-3-(4-bromophenyl)-2-((*tert*-butoxycarbonyl)amino)propanoyl)-*L*-

serinate (16h). According to the general procedure F, under N_2, to a solution of (*S*)-3-(4-bromophenyl)-2-((*tert*-butoxycarbonyl)amino)propanoic acid (1.03 g, 3.00 mmol, 1.00 equiv) in DMF (15.0 mL) was added HATU (1.71 g, 4.50 mmol, 1.50 equiv), HOBT (608 mg, 4.50 mmol, 1.50 equiv), followed by the addition of DIPEA (1.16 g, 9.00 mmol, 3.00 equiv). After stirring at 0 °C for 0.5 h, the solution was added *L*-Serine benzyl ester hydrochloride (834 mg, 3.60 mmol, 1.20 equiv). After stirring at rt for another 12 hours, the crude material was quenched with water, extracted with EtOAc (3 × 30.0 mL), washed with 1.0 M HCl solution (2 × 60.0 mL), saturated $NaHCO_3$ solution (2 × 60.0 mL), and brine (2 × 60.0 mL). The organic layers were dried over Na_2SO_4, filtered, and concentrated under reduced pressure. The residue was purified by chromatographic purification on SiO_2 (Petroleum ether:Acetone, 1:0 –1.5:1) to afford **16h** as alight-yellow powder (894 mg, 67%): **^{1}H NMR** (400 MHz, $CDCl_3$) δ 7.38 (dd, *J* = 8.3, 1.7 Hz, 2H), 7.28 – 7.26 (m, 1H), 7.08 (dd, *J* = 8.3, 1.7 Hz, 2H), 5.43 (d, *J* = 8.0 Hz, 1H), 4.63 – 4.60 (m, 1H), 4.47 – 4.42 (m, 1H), 3.92 – 3.84 (m, 2H), 3.73 – 3.66 (m, 4H), 3.08 (dd, *J* = 14.0, 5.9 Hz, 1H), 2.94 – 2.88 (m, 1H), 1.35 (s, 9H); **^{13}C NMR** (101 MHz, $CDCl_3$) δ 171.8, 170.7, 155.9, 135.7, 131.7, 131.2(2), 120.9, 80.6, 62.7, 55.6, 54.8, 52.8, 38.0, 28.3; **HRMS** (ESI) m/z calcd for $C_{18}H_{25}O_6N_2BrNa$ [M + Na]+ 467.0788, found 467.0799.

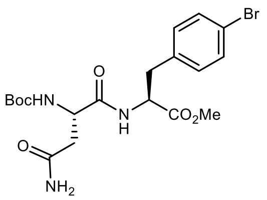

16i

Methyl (*S*)-2-((*S*)-4-amino-2-((*tert*-butoxycarbonyl)amino)-4-oxobutanamido)-3-(4-bromophenyl)propanoate (16i). According to the general procedure G, under ice-salt bath, to the solution of *L*-4-Bromophenylalanine methyl ester hydrochloride (1.76 g, 6.00 mmol, 1.00 equiv), N^2-[(*tert*-butoxy)carbonyl]-*L*-asparagine (1.40 g, 6.00 mmol, 1.00 equiv), and HOBT (1.35 g, 10.0 mmol, 1.66 equiv) in dry DCM (20.0 mL) was added DIPEA (3.00 mL, 18.0 mmol, 3.00 equiv). The reaction mixture was stirred at 0°C, added the solution of EDCI (1.91 g, 10.0 mmol, 1.66 equiv) in 20.0 mL of dry DCM dropwise. Then the solution was stirred at room temperature for 24 h. A lot of solid appeared, filtered, and the solid was washed with saturated NH_4Cl solution, 1 N HCl solution, and saturated $NaHCO_3$ solution, respectively. The crude product was twice recrystallized from CH_3OH to afford white powder **16i** (1.24 g, 44%): **^{1}H NMR** (400 MHz, DMSO-d_6) δ 8.23 (d, *J* = 7.4 Hz, 0.2H), 8.14 (d, *J* = 7.7 Hz, 0.8H), 7.45 – 7.43 (m, 2H), 7.26 – 7.15 (m, 3H), 6.93 – 6.87 (m, 1.8H), 6.43 (d, *J* = 7.7 Hz, 0.2H), 4.47 – 4.42 (m, 1H), 4.27 – 4.22 (m, 0.8H), 4.14 – 4.11 (m, 0.2H), 3.59 (s, 3H), 3.02 – 2.88 (m, 2H), 2.38 – 2.27 (m, 2H), 1.35 – 1.21 (m, 9H); **^{13}C NMR** (101 MHz, DMSO-d_6) δ 171.8, 171.6, 171.4, 155.1, 136.5, 131.5, 131.1, 119.8, 78.2, 53.3, 52.0, 51.2, 37.2, 35.8, 28.2; **HRMS** (ESI) m/z calcd for $C_{19}H_{26}N_3O_6BrNa$ [M + Na]+ 494.0897, found 494.0906

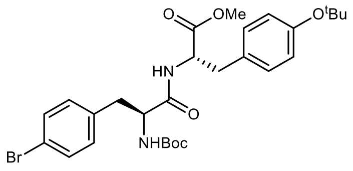

16j

Methyl (*S*)-2-((*S*)-3-(4-bromophenyl)-2-((*tert*-butoxycarbonyl)amino)propanamido)-3-(4-(*tert*-butoxy)phenyl)propanoate (16j). According to the general procedure F, under N_2, to a solution of (*S*)-3-(4-bromophenyl)-2-((*tert*-butoxycarbonyl)amino)propanoic acid (1.03 g, 3.00 mmol, 1.00 equiv) in DMF (15.0 mL) was added HATU (1.71 g, 4.50 mmol, 1.50 equiv), HOBT (608 mg, 4.50 mmol, 1.50 equiv), followed by the addition of DIPEA (1.16 g, 9.00 mmol, 3.00 equiv). After stirring at 0 °C for 0.5 h, the solution was added methyl *O*-*tert*-butyl-L-tyrosinate hydrochloride (1.03 g, 3.60 mmol, 1.20 equiv). After stirring at rt for another 12 hours, the crude material was quenched with water, extracted with EtOAc (3 × 30.0 mL), washed with 1.0 M HCl solution (2 × 60.0 mL), saturated $NaHCO_3$ solution (2 × 60.0 mL), and brine (2 × 60.0 mL). The organic layers were dried over Na_2SO_4, filtered, and concentrated under reduced pressure. The residue was purified by chromatographic purification on SiO_2 (Petroleum ether : EtOAc:DCM, 1:0:0 –3:1:0.4) to afford **16j** as a light-yellow solid (1.37 g, 79%): **^{1}H NMR** (500 MHz, $CDCl_3$) δ 7.40 – 7.38 (m, 2H), 7.05 (d, *J* = 8.1 Hz, 2H), 6.90 – 6.85 (m, 4H), 6.33 (d, *J* = 7.7 Hz, 1H), 4.97 – 4.96 (m, 1H), 4.74 – 4.70 (m, 1H), 4.32 – 4.26 (m, 1H), 3.64 (s, 3H), 3.03 – 2.95 (m, 4H), 1.40 (s, 9H), 1.31 (s, 9H); **^{13}C NMR** (126 MHz, $CDCl_3$) δ 171.5, 170.5, 155.3, 154.6, 135.6, 131.8, 131.2, 130.4, 129.7, 124.3, 121.0, 80.5, 78.5, 55.5, 53.5, 52.4, 37.8, 37.5, 28.9, 28.3; **HRMS** (ESI) *m/z* calcd for $C_{28}H_{37}BrO_6Na$ [M + Na]$^+$ 599.1727, found 599.1729.

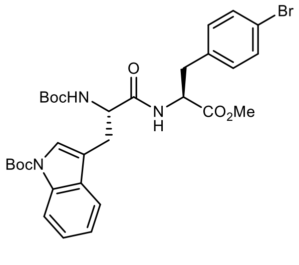

16k

***tert*-Butyl 3-((*S*)-3-(((*S*)-3-(4-bromophenyl)-1-methoxy-1-oxopropan-2-yl)amino)-2-((*tert*-butoxycarbonyl)amino)-3-oxopropyl)-1*H*-indole-1-carboxylate (16k).** According to the general procedure F, under N_2, to a solution of N^a,1-bis(*tert*-butoxycarbonyl)-*L*-tryptophan (1.00 g, 2.50 mmol, 1.00 equiv) in DMF (15.0 mL) was added HATU (1.42 g, 3.75 mmol, 1.50 equiv), HOBT (506 mg, 3.75 mmol, 1.50 equiv), followedby the addition of DIPEA (967 mg, 7.50 mmol, 3.00 equiv). After stirring at 0 °C for 0.5 h, the solution was added *L*-4-Bromophenylalanine methyl ester hydrochloride (885 mg, 3.00 mmol, 1.20 equiv). After stirring at rt for another 12 hours, the crude material was quenched with water, extracted with EtOAc (3 × 30.0 mL), washed with 1.0 M HCl solution (2 × 60.0 mL), saturated $NaHCO_3$ solution (2 × 60.0

mL), and brine (2 × 60.0 mL). The organic layers were dried over Na_2SO_4, filtered, and concentrated under reduced pressure. The residue was purified by chromatographic purification on SiO_2 (Petroleum ether:Acetone, 1:0 –5:1) to afford **16k** as a white powder (1.40 g, 87%): **^{1}H NMR** (400 MHz, Acetone-*d*$_6$) δ 8.13 (d, *J* = 8.2 Hz, 1H), 7.64 – 7.61 (m, 2H), 7.56 (s, 1H), 7.41 (d, *J* = 8.0 Hz, 2H), 7.33 – 7.29 (m, 1H), 7.25 – 7.21 (m, 1H), 7.13 (d, *J* = 8.1 Hz, 2H), 6.22 (d, *J* = 8.5 Hz, 1H), 4.78 – 4.73 (m, 1H), 4.56 – 4.51 (m, 1H), 3.68 (s, 3H), 3.21 (dd, *J* = 14.9, 5.1 Hz, 1H), 3.14 – 3.04 (m, 2H), 3.00 (dd, *J* = 13.8, 7.3 Hz, 1H), 1.66 (s, 9H), 1.36 (s, 9H); **^{13}C NMR** (101 MHz, Acetone-*d*$_6$) δ 172.1(2), 156.2, 150.2, 137.0, 136.2, 132.3, 132.1, 131.5, 125.0, 124.9, 123.2, 121.0, 120.0, 117.3, 115.8, 84.0, 79.4, 55.1, 54.1, 52.5, 37.5, 30.6, 28.5, 28.2; **HRMS** (ESI) m/z calcd for $C_{31}H_{38}N_3O_7BrNa$ [M + Na]+ 666.1785, found 666.1791.

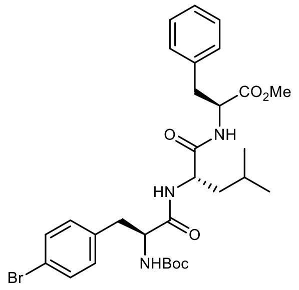

16l

Methyl ((*S*)-3-(4-bromophenyl)-2-((*tert*-butoxycarbonyl)amino)propanoyl)-*L*-leucyl-*L*-phenylalaninate (16l). According to the general procedure F, under N_2, to a solution of (*S*)-3-(4-bromophenyl)-2-((*tert*-butoxycarbonyl)amino)propanoic acid (1.03 g, 3.00 mmol, 1.00 equiv) in DMF (15.0 mL) was added HATU (1.71 g, 4.50 mmol, 1.50 equiv), HOBT (608 mg, 4.50 mmol, 1.50 equiv), followed by the addition of DIPEA (1.16 g, 9.00 mmol, 3.00 equiv). After stirring at 0 °C for 0.5 h, the solution was added methyl *L*-leucyl-*L*-phenylalaninate hydrochloride[9] (1.18 g, 3.60 mmol, 1.20 equiv). After stirring at rt for another 12 hours, the crude material was quenched with water, extracted with EtOAc (3 × 30.0 mL), washed with 1.0 M HCl solution (2 × 60.0 mL), saturated $NaHCO_3$ solution (2 × 60.0 mL), and brine (2 × 60.0 mL). The organic layers were dried over Na_2SO_4, filtered, and concentrated under reduced pressure. The residue was purified by chromatographic purification on SiO_2 (Petroleum ether:Acetone, 1:0 –2:1) to afford **16i** as a light-yellow powder (914 mg, 49%): **^{1}H NMR** (400 MHz, $CDCl_3$) δ 7.39 (d, *J* = 8.1 Hz, 2H), 7.31 – 7.22 (m, 4H), 7.11 – 7.05 (m, 3H), 6.51 (d, *J* = 8.4 Hz, 1H), 6.22 (d, *J* = 7.9 Hz, 1H), 4.95 (s, 1H), 4.85 – 4.80 (m, 1H), 4.31 (d, *J* = 7.4 Hz, 1H), 4.20 (dd, *J* = 8.4, 6.2 Hz, 1H), 3.71 (s, 3H), 3.15 – 2.95 (m, 4H), 1.82 – 1.79 (m, 1H), 1.41 (s, 9H), 1.36 – 1.28 (m, 1H), 1.07 – 0.96 (m, 1H), 0.85 – 0.81 (m, 6H); **^{13}C NMR** (101 MHz, $CDCl_3$) δ 171.7, 170.9, 170.3, 135.8, 131.9, 131.2, 129.4, 128.8, 127.4, 57.9, 53.3, 52.5, 38.0, 37.3, 28.4, 24.8, 15.3, 11.5; **HRMS** (ESI) m/z calcd for $C_{30}H_{40}N_3O_6BrNa$ [M + Na]+ 640.1993, found 640.2010

2.3. Detailed experimental procedures for the palladium-catalyzed Suzuki-Miyaura cross-couplings

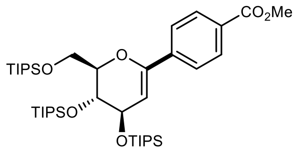

15a

Methyl 4-((2*R*,3*R*,4*R*)-3,4-bis((triisopropylsilyl)oxy)-2-(((triisopropylsilyl)oxy)methyl)-3,4-dihydro-2*H*-pyran-6-yl)benzoate (15a). According to the general procedue A, glycal boronates **12b** (95.0 mg, 0.12 mmol, 1.20 equiv), methyl 4-bromobenzoate (21.5mg, 0.10 mmol, 1.00 equiv), $Pd(PPh_3)_2Cl_2$ (3.50 mg, 0.005 mmol, 5.00 mol%), K_3PO_4 (63.6 mg, 0.30 mmol, 3.00 equiv) were added to a one-dram vial with a screw-top septum, and the vial was then evacuated and refilled with N_2 (3×). Anhydrous DMF (2.00 mL) were added, and the reaction mixture was stirred at 33 °C for 48 h, cooled to rt, and concentrated. The crude material was purified by column chromatography on SiO_2 (Petroleum ether:Ether, 1:0-50:1) to afford **15a** (69.5 mg, 93%) as a colorless oil: **^{1}H NMR** (500 MHz, Acetone-*d*$_6$) δ 8.00 (d, *J* = 8.3 Hz, 2H), 7.84 (d, *J* = 8.3 Hz, 2H), 5.71 (dd, *J* = 5.3, 1.5 Hz, 1H), 4.59 – 4.56 (m, 1H), 4.30 – 4.22 (m, 3H), 3.99 (dd, *J* = 11.4, 3.7 Hz, 1H), 3.88 (s, 3H), 1.19 – 1.04 (m, 63H); **^{13}C NMR** (126 MHz, Acetone-*d*$_6$) δ 166.9, 150.0, 141.1, 130.9, 130.0, 126.0, 99.5, 82.5, 70.8, 67.4, 62.7, 52.3, 18.6(2), 18.5(2), 18.4(2), 13.3, 13.2, 12.8; **HRMS** (ESI) *m/z* calcd for $C_{41}H_{76}O_6Si_3Na$ $[M + Na]^+$ 771.4842, found 771.4839.

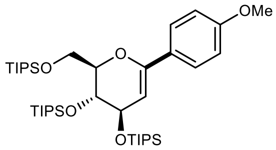

15b

(((2*R*,3*R*,4*R*)-6-(4-Methoxyphenyl)-2-(((triisopropylsilyl)oxy)methyl)-3,4-dihydro-2*H*-pyran-3,4-diyl)bis(oxy))bis(triisopropylsilane) (15b). According to the general procedure A, glycal boronates **12b** (95.0 mg, 0.12 mmol, 1.20 equiv), 1-bromo-4-methoxybenzene (18.7 mg, 0.10 mmol, 1.00 equiv), $Pd(PPh_3)_2Cl_2$ (3.50 mg, 0.005 mmol, 5.00 mol%), K_3PO_4 (63.6 mg, 0.30 mmol, 3.00 equiv) were added to a one-dram vial with a screw-top septum, and the vial was then evacuated and refilled with N_2 (3×). Anhydrous DMF (2.00 mL) were added, and the reaction mixture was stirred at 33 °C for 48 h, cooled to rt, and concentrated. The crude material was purified by column chromatography on SiO_2 (Petroleum ether: ether, 1:0 - 50:1) to afford **15b** (60.9 mg, 85%) as a colorless oil: **^{1}H NMR** (400 MHz, $CDCl_3$) δ 7.63 – 7.54 (m, 2H), 6.90 – 6.80 (m, 2H), 5.24 (dd, *J* = 5.4, 1.5 Hz, 1H), 4.47 – 4.43 (m, 1H), 4.19 – 4.09 (m, 3H), 3.90 (dd, *J* = 11.2, 4.2 Hz, 1H), 3.82 (s, 3H), 1.12 – 1.02 (m, 63H); **^{13}C NMR** (101 MHz, $CDCl_3$) δ 159.8, 150.1, 129.2, 126.8, 113.4, 95.4, 81.4, 70.3, 67.0, 62.1, 55.4, 18.4, 18.3(2), 18.2(2), 18.1, 12.7, 12.6, 12.2; **HRMS** (ESI) *m/z* calcd for $C_{40}H_{77}O_5Si_3Na$ [M

+ Na]$^+$ 721.5073, found 721.5085.

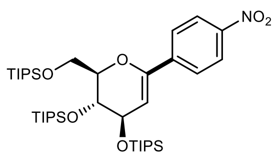

15c

(((2*R*,3*R*,4*R*)-6-(4-nitrophenyl)-2-(((triisopropylsilyl)oxy)methyl)-3,4-dihydro-2*H*-pyran-3,4-diyl)bis(oxy))bis(triisopropylsilane) (15c). According to the general procedure A, glycal boronates **12b** (95.0 mg, 0.12 mmol, 1.20 equiv), 1-bromo-4-nitrobenzene (20.2 mg, 0.10 mmol, 1.00 equiv), $Pd(PPh_3)_2Cl_2$ (3.50 mg, 0.005 mmol, 5.00 mol%), K_3PO_4 (63.6 mg, 0.30 mmol, 3.00 equiv) were added to a one-dram vial with a screw-top septum, and the vial was then evacuated and refilled with N_2 (3×). Anhydrous DMF (2.00 mL) were added, and the reaction mixture was stirred at 33 °C for 48 h, cooled to rt, and concentrated. The crude material was purified by column chromatography on SiO_2 (Petroleum ether:EtOAc, 1:0-50:1) to afford **15c** (71.3 mg, 97%) as a light-yellow oil: **^{1}H NMR** (400 MHz, Acetone-*d*$_6$) δ 8.28 – 8.22 (m, 2H), 8.03 – 7.96 (m, 2H), 5.85 (dd, *J* = 5.4, 1.6 Hz, 1H), 4.63 – 4.59 (m, 1H), 4.32 – 4.22 (m, 3H), 3.99 (dd, *J* = 11.4, 3.7 Hz, 1H), 1.16 – 1.03 (m, 63H); **^{13}C NMR** (101 MHz, Acetone-*d*$_6$) δ 149.0, 148.7, 142.8, 126.8, 124.2, 101.1, 82.6, 70.7, 67.2, 62.6, 18.6(2), 18.5(2), 18.4(3), 13.2, 13.1, 12.7; **HRMS** (ESI) *m/z* calcd for $C_{39}H_{73}NO_6Si_3Na$ [M + Na]$^+$ 758.4638, found 758.4641.

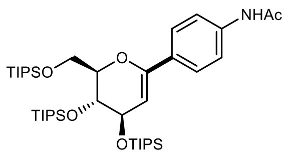

15d

***N*-(4-((2*R*,3*R*,4*R*)-3,4-bis((triisopropylsilyl)oxy)-2-(((triisopropylsilyl)oxy)methyl)-3,4-dihydro-2*H*-pyran-6-yl)phenyl)acetamide (15d)**. According to the general procedure A, glycal boronates **12b** (95.0 mg, 0.12 mmol, 1.20 equiv), 4'-bromoacetanilide (20.8 mg, 0.10 mmol, 1.00 equiv), $Pd(PPh_3)_2Cl_2$ (3.50 mg, 0.005 mmol, 5.00 mol%), K_3PO_4 (63.6 mg, 0.30 mmol, 3.00 equiv) were added to a one-dram vial with a screw-top septum, and the vial was then evacuated and refilled with N_2 (3×). Anhydrous DMF (2.00 mL) were added, and the reaction mixture was stirred at 33 °C for 48 h, cooled to rt, and concentrated. The crude material was purified by column chromatography on SiO_2 (Petroleum ether:EtOAc, 1:0-5:1) to afford **15d** (71.3 mg, 95%) as a white foam: **^{1}H NMR** (500 MHz, Acetone-*d*$_6$) δ 9.24 (s, 1H), 7.65 – 7.61 (m, 4H), 5.45 (dd, *J* = 5.4, 1.5 Hz, 1H), 4.53 – 4.50 (m, 1H), 4.28 – 4.26 (m, 1H), 4.24 – 4.20 (m, 2H), 3.99 (dd, *J* = 11.3, 3.8 Hz, 1H), 2.08 (s, 3H), 1.16 – 1.05 (m, 63H); **^{13}C NMR** (126 MHz, Acetone-*d*$_6$) δ 168.8, 150.7, 140.8, 131.6, 126.4, 119.2, 96.2, 82.2, 71.0, 67.7, 62.9, 24.3, 18.7, 18.6, 18.5(2), 18.4,(2) 13.3, 13.2, 12.7; **HRMS** (ESI) m/z calcd for $C_{41}H_{78}NO_5Si_3$ [M + H] $^+$ 748.5182, found 748.5189.

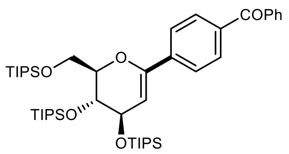

15e

(4-((2*R*,3*R*,4*R*)-3,4-Bis((triisopropylsilyl)oxy)-2-(((triisopropylsilyl)oxy)methyl)-3,4-dihydro-2*H*-pyran-6-yl)phenyl)(phenyl)methanone (15e). According to the general procedure A, glycal boronates **12b** (95.0 mg, 0.12 mmol, 1.20 equiv), 4-bromobenzophenone (26.1 mg, 0.10 mmol, 1.00 equiv), $Pd(PPh_3)_2Cl_2$ (3.50 mg, 0.005 mmol, 5.00 mol%), K_3PO_4 (63.6 mg, 0.30 mmol, 3.00 equiv) were added to a one-dram vial with a screw-top septum, and the vial was then evacuated and refilled with N_2 (3×). Anhydrous DMF (2.00 mL) were added, and the reaction mixture was stirred at 33 °C for 48 h, cooled to rt, and concentrated. The crude material was purified by column chromatography on SiO_2 (Petroleum ether:EtOAc, 1:0-30:1) to afford **15e** (73.4 mg, 92%) as a light-yellow oil: **^{1}H NMR** (400 MHz, $CDCl_3$) δ 7.82 – 7.74 (m, 6H), 7.61 – 7.57 (m, 1H), 7.50 – 7.47 (m, 2H), 5.51 (dd, *J* = 5.4, 1.5 Hz, 1H), 4.53 – 4.49 (m, 1H), 4.21 – 4.12 (m, 3H), 3.88 (dd, *J* = 11.3, 3.9 Hz, 1H), 1.11 – 1.01 (m, 63H); **^{13}C NMR** (101 MHz, $CDCl_3$) δ 196.5, 149.4, 140.2, 137.9, 137.1, 132.5, 130.2, 130.1, 128.4, 125.2, 98.9, 81.6, 70.1, 66.6, 62.0, 18.3(3), 18.2, 18.1(2), 12.6, 12.5, 12.1; **HRMS** (ESI) *m/z* calcd for $C_{46}H_{79}O_5Si_3$ $[M + H]^+$ 795.5230, found 795.5240.

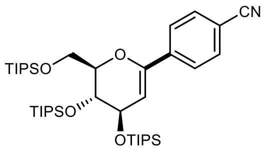

15f

4-((2*R*,3*R*,4*R*)-3,4-Bis((triisopropylsilyl)oxy)-2-(((triisopropylsilyl)oxy)methyl)-3,4-dihydro-2*H*-pyran-6-yl)benzonitrile (15f). According to the general procedure A, glycal boronates **12b** (95.0 mg, 0.12 mmol, 1.20 equiv), 4-bromobenzonitrile (18.2 mg, 0.10 mmol, 1.00 equiv), $Pd(PPh_3)_2Cl_2$ (3.50 mg, 0.005 mmol, 5.00 mol%), K_3PO_4 (63.6 mg, 0.30 mmol, 3.00 equiv) were added to a one-dram vial with a screw-top septum, and the vial was then evacuated and refilled with N_2 (3×). Anhydrous DMF (2.00 mL) were added, and the reaction mixture was stirred at 33 °C for 48 h, cooled to rt, and concentrated. The crude material was purified by column chromatography on SiO_2 (Petroleum ether:EtOAc, 1:0-30:1) to afford **15f** (54.1 mg, 76%) as a light-yellow oil: **^{1}H NMR** (400 MHz, $CDCl_3$) δ 7.73 (d, *J* = 8.4 Hz, 2H), 7.61 (d, *J* = 8.3 Hz, 2H), 5.48 (dd, *J* = 5.4, 1.5 Hz, 1H), 4.51 – 4.47 (m, 1H), 4.18 – 4.09 (m, 3H), 3.83 (dd, *J* = 11.4, 3.7 Hz, 1H), 1.11 – 0.99 (m, 63H); **^{13}C NMR** (101 MHz, $CDCl_3$) δ 148.6, 140.6, 132.0, 125.9, 119.2, 111.7, 99.7, 81.7, 69.9, 66.4, 61.8, 18.3, 18.2(3), 18.1(2), 12.6, 12.5, 12.1; **HRMS** (ESI) *m/z* calcd for $C_{40}H_{74}NO_4Si_3$ $[M + H]^+$ 716.4920, found 716.4926.

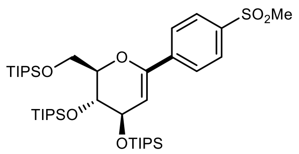

15g

(((2*R*,3*R*,4*R*)-6-(4-(Methylsulfonyl)phenyl)-2-(((triisopropylsilyl)oxy)methyl)-3,4-dihydro-2*H*-pyran-3,4-diyl)bis(oxy))bis(triisopropylsilane) (15g). According to the general procedure A, glycal boronates **12b** (95.0 mg, 0.12 mmol, 1.20 equiv), 4-bromophenyl methyl sulfone (23.5 mg, 0.10 mmol, 1.00 equiv), $Pd(PPh_3)_2Cl_2$ (3.50 mg, 0.005 mmol, 5.00 mol%), K_3PO_4 (63.6 mg, 0.30 mmol, 3.00 equiv) were added to a one-dram vial with a screw-top septum, and the vial was then evacuated and refilled with N_2 (3×). Anhydrous DMF (2.00 mL) were added, and the reaction mixture was stirred at 33 °C for 48 h, cooled to rt, and concentrated. The crude material was purified by column chromatography on SiO_2 (Petroleum ether:EtOAc, 1:0-10:1)to afford **15g** (65.0 mg, 85%) as a light-yellow oil: **^{1}H NMR** (400 MHz, $CDCl_3$) δ 7.95 – 7.87 (m, 2H), 7.82 (d, *J* = 8.6 Hz, 2H), 5.51 (dd, *J* = 5.4, 1.5 Hz, 1H), 4.52 – 4.48 (m, 1H), 4.19 – 4.10 (m, 3H), 3.84 (dd, *J* = 11.4, 3.7 Hz, 1H), 3.05 (s, 3H), 1.11 – 0.99 (m, 63H); **^{13}C NMR** (101 MHz, $CDCl_3$) δ 148.6, 141.6, 139.8, 127.3, 126.2, 99.8, 81.8, 70.0, 66.4, 61.8, 44.7, 18.3, 18.2(3), 18.1(2), 12.6, 12.5, 12.1; **HRMS** (ESI) *m/z* calcd for $C_{40}H_{77}O_6SSi_3$ $[M + H]^+$ 769.4743, found 769.4749.

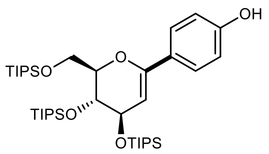

15h

4-((2*R*,3*R*,4*R*)-3,4-Bis((triisopropylsilyl)oxy)-2-(((triisopropylsilyl)oxy)methyl)-3,4-dihydro-2*H*-pyran-6-yl)phenol (15h). According to the general procedure A, glycal boronates **12b** (95.0 mg, 0.12 mmol, 1.20 equiv), 4-bromophbenol (17.3 mg, 0.10 mmol, 1.00 equiv), $Pd(PPh_3)_2Cl_2$ (3.50 mg, 0.005 mmol, 5.00 mol%), K_3PO_4 (63.6 mg, 0.30 mmol, 3.00 equiv) were added to a one-dram vial with a screw-top septum, and the vial was then evacuated and refilled with N_2 (3×). Anhydrous DMF (2.00 mL) were added, and the reaction mixture was stirred at 33 °C for 48 h, cooled to rt, and concentrated. The crude material was purified by column chromatography on SiO_2 (Petroleum ether:EtOAc, 1:0-10:1) to afford **15h** (28.6 mg, 41%) as a light-yellow oil: **^{1}H NMR** (400 MHz, Acetone-*d*$_6$) δ 8.50 (s, 1H), 7.61 – 7.47 (m, 2H), 6.81 (d, *J* = 8.7 Hz, 2H), 5.35 (dd, *J* = 5.4, 1.5 Hz, 1H), 4.52 – 4.48 (m, 1H), 4.27 – 4.16 (m, 3H), 3.99 (dd, *J* = 11.2, 3.9 Hz, 1H), 1.17 – 1.04 (m, 63H); **^{13}C NMR** (101 MHz, Acetone-*d*$_6$) δ 158.8, 151.1, 128.4, 127.5, 115.7, 115.6, 95.1, 82.2, 71.1, 67.9, 62.9, 18.7, 18.6, 18.5(2), 18.4(2), 18.2, 13.3, 13.2, 12.8; **HRMS** (ESI) *m/z* calcd for $C_{39}H_{75}O_5Si_3$ $[M + H]^+$ 707.4917, found 707.4920.

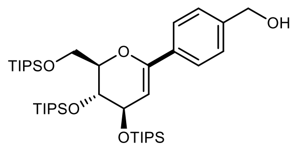

15i

(4-((2*R*,3*R*,4*R*)-3,4-Bis((triisopropylsilyl)oxy)-2-(((triisopropylsilyl)oxy)methyl)-3,4-dihydro-2*H*-pyran-6-yl)phenyl)methanol (15i). According to the general procedure A, glycal boronates **12b** (95.0 mg, 0.12 mmol, 1.20 equiv), 4-bromobenzyl alcohol (18.7 mg, 0.10 mmol, 1.00 equiv), $Pd(PPh_3)_2Cl_2$ (3.50 mg, 0.005 mmol, 5.00 mol%), K_3PO_4 (63.6 mg, 0.30 mmol, 3.00 equiv) were added to a one-dram vial with a screw-top septum, and the vial was then evacuated and refilled with N_2 (3×). Anhydrous DMF (2.00 mL) were added, and the reaction mixture was stirred at 33 °C for 48 h, cooled to rt, and concentrated. The crude material was purified by column chromatography on SiO_2 (Petroleum ether:EtOAc, 1:0-5:1) to afford **15i** (50.6 mg, 70%) as a colorless oil: **^{1}H NMR** (400 MHz, $CDCl_3$) δ 7.64 (d, *J* = 8.2 Hz, 2H), 7.33 (d, *J* = 8.1 Hz, 2H), 5.36 (dd, *J* = 5.3, 1.5 Hz, 1H), 4.69 (d, *J* = 3.6 Hz, 2H), 4.49 – 4.45 (m, 1H), 4.19 – 4.17 (m, 1H), 4.15 – 4.10 (m, 2H), 3.89 (dd, *J* = 11.2, 4.1 Hz, 1H), 1.69 (s, 1H), 1.10 – 1.01 (m, 63H); **^{13}C NMR** (101 MHz, $CDCl_3$) δ 150.0, 140.9, 135.9, 126.8, 125.7, 96.9, 81.4, 70.2, 66.8, 65.3, 62.1, 18.3(3), 18.2(2), 18.1(2), 12.7, 12.6, 12.1; **HRMS** (ESI) *m/z* calcd for $C_{40}H_{77}O_5Si_3$ $[M + H]^+$ 721.5073, found 721.5073.

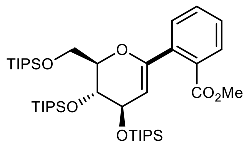

15j

Methyl 2-((2*R*,3*R*,4*R*)-3,4-bis((triisopropylsilyl)oxy)-2-(((triisopropylsilyl)oxy)methyl)-3,4-dihydro-2*H*-pyran-6-yl)benzoate (15j). According to the general procedure A, glycal boronates **12b** (95.0 mg, 0.12 mmol, 1.20 equiv), methyl 2-bromobenzoate (21.5 mg, 0.10 mmol, 1.00 equiv), $Pd(PPh_3)_2Cl_2$ (3.50 mg, 0.005 mmol, 5.00 mol%), K_3PO_4 (63.6 mg, 0.30 mmol, 3.00 equiv) were added to a one-dram vial with a screw-top septum, and the vial was then evacuated and refilled with N_2 (3×). Anhydrous DMF (2.00 mL) were added, and the reaction mixture was stirred at 33 °C for 48 h, cooled to rt, and concentrated. The crude material was purified by column chromatography on SiO_2 (Petroleum ether:Ether, 1:0-50:1) to afford **15j** (70.3 mg, 94%) as a colorless oil: **^{1}H NMR** (400 MHz, $CDCl_3$) δ 7.63 (dd, *J* = 7.6, 1.4 Hz, 1H), 7.48 (dd, *J* = 7.7, 1.5 Hz, 1H), 7.45 – 7.41 (m, 1H), 7.38 – 7.33 (m, 1H), 5.02 (dd, *J* = 5.3, 1.5 Hz, 1H), 4.39 – 4.35 (m, 1H), 4.27 – 4.25 (m, 1H), 4.23 – 4.21 (m, 1H), 4.14 (dd, *J* = 10.7, 6.4 Hz, 1H), 4.01 (dd, *J* = 10.7, 6.5 Hz, 1H), 3.83 (s, 3H), 1.12 – 1.03 (m, 63H); **^{13}C NMR** (101 MHz, $CDCl_3$) δ 169.2, 151.7, 136.7, 131.5, 130.8, 129.8, 129.1, 128.4, 99.4, 81.6, 69.6, 67.0, 61.7, 52.2, 18.4, 18.3(2), 18.2, 12.7, 12.6, 12.2; **HRMS** (ESI) *m/z* calcd for $C_{41}H_{76}O_6Si_3Na$ $[M + Na]^+$ 771.4842, found 771.4836.

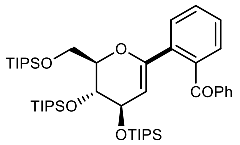

15k

(2-((2*R*,3*R*,4*R*)-3,4-Bis((triisopropylsilyl)oxy)-2-(((triisopropylsilyl)oxy)methyl)-3,4-dihydro-2*H*-pyran-6-yl)phenyl)(phenyl)methanone (15k). According to the general procedure A, glycal boronates **12b** (95.0 mg, 0.12 mmol, 1.20 equiv), 2-bromobenzophenone (26.1 mg, 0.10 mmol, 1.00 equiv), $Pd(PPh_3)_2Cl_2$ (3.50 mg, 0.005 mmol, 5.00 mol%), K_3PO_4 (63.6 mg, 0.30 mmol, 3.00 equiv) were added to a one-dram vial with a screw-top septum, and the vial was then evacuated and refilled with N_2 (3×). Anhydrous DMF (2.00 mL) were added, and the reaction mixture was stirred at 33 °C for 48 h, cooled to rt, and concentrated. The crude material was purified by column chromatography on SiO_2 (Petroleum ether:Ether, 1:0-50:1) to afford **15k** (73.9 mg, 93%) as a colorless oil: **^{1}H NMR** (500 MHz, Acetone-d_6) δ 7.78 – 7.73 (m, 3H), 7.63 – 7.59 (m, 1H), 7.54 – 7.45 (m, 4H), 7.27 (dd, J = 7.5, 1.4 Hz, 1H), 5.27 (dd, J = 5.3, 1.5 Hz, 1H), 4.27 – 4.26 (m, 1H), 4.18 – 4.16 (m, 1H), 4.14 – 4.10 (m, 1H), 3.99 (dd, J = 10.4, 7.9 Hz, 1H), 3.61 (dd, J = 10.4, 5.8 Hz, 1H), 1.09 – 1.02 (m, 63H); **^{13}C NMR** (126 MHz, Acetone-d_6) δ 197.2, 151.2, 139.9, 138.2, 135.7, 133.8, 130.4, 130.0, 129.3, 129.2, 128.7, 128.1, 101.1, 81.5, 70.2, 67.9, 61.2, 18.7, 18.6(2), 18.5, 18.4, 13.2(2), 12.6; **HRMS** (ESI) m/z calcd for $C_{46}H_{79}O_5Si_3$ $[M + H]^+$ 795.5230, found 795.5233.

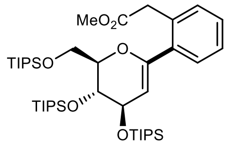

15l

Methyl 2-(2-((2*R*,3*R*,4*R*)-3,4-bis((triisopropylsilyl)oxy)-2-(((triisopropylsilyl)oxy)methyl)-3,4-dihydro-2*H*-pyran-6-yl)phenyl)acetate (15l). According to the general procedure A, glycal boronates **12b** (95.0 mg, 0.12 mmol, 1.20 equiv), methyl 2-(2-bromophenyl)acetate (22.9 mg, 0.10 mmol, 1.00 equiv), $Pd(PPh_3)_2Cl_2$ (3.50 mg, 0.005 mmol, 5.00 mol%), K_3PO_4 (63.6 mg, 0.30 mmol, 3.00 equiv) were added to a one-dram vial with a screw-top septum, and the vial was then evacuated and refilled with N_2 (3×). Anhydrous DMF (2.00 mL) were added, and the reaction mixture was stirred at 33 °C for 48 h, cooled to rt, and concentrated. The crude material was purified by column chromatography on SiO_2 (Petroleum ether:Ether, 1:0-50:1) to afford **15l** (67.8 mg, 89%) as a colorless oil: **^{1}H NMR** (500 MHz, Acetone-d_6) δ 7.38 – 7.25 (m, 4H), 5.05 (dd, J = 5.5, 1.6 Hz, 1H), 4.48 – 4.44 (m, 1H), 4.28 – 4.22 (m, 3H), 4.09 (dd, J = 11.1, 4.7 Hz, 1H), 3.90 (d, J = 16.3 Hz, 1H), 3.79 – 3.78 (m, 1H), 3.62 (s, 3H), 1.18 – 1.06 (m, 63H); **^{13}C NMR** (126 MHz, Acetone-d_6) δ 172.5, 152.9, 138.1, 134.0, 131.5, 129.9, 129.3, 127.5, 100.8, 82.5, 70.2, 67.2, 62.8, 51.9, 38.9, 18.6(2), 18.5(2), 18.4, 13.2(2), 12.7; **HRMS** (ESI) *m/z* calcd for $C_{42}H_{79}O_6Si_3$ $[M + H]^+$ 763.5179, found 763.5181.

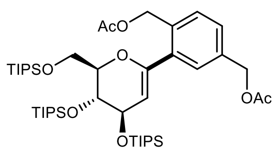

15m

(2-((2*R*,3*R*,4*R*)-3,4-Bis((triisopropylsilyl)oxy)-2-(((triisopropylsilyl)oxy)methyl)-3,4-dihydro-2*H*-pyran-6-yl)-1,4-phenylene)bis(methylene) diacetate (15m). According to the general procedure A, glycal boronates **12b** (95. 0 mg, 0.12 mmol, 1.20 equiv), (2-bromo-1,4-phenylene)bis(methylene) diacetate[10] (30.0 mg, 0.10 mmol, 1.00 equiv), $Pd(PPh_3)_2Cl_2$ (3.50 mg, 0.005 mmol, 5.00 mol%), K_3PO_4 (63.6 mg, 0.30 mmol, 3.00 equiv) were added to a one-dram vial with a screw-top septum, and the vial was then evacuated and refilled with N_2 (3×). Anhydrous DMF (2.00 mL) were added, and the reaction mixture was stirred at 33 °C for 48 h, cooled to rt, and concentrated. The crude material was purified by column chromatography on SiO_2 (Petroleum ether: EtOAc, 1:0-10:1) to afford **15m** (72.5 mg, 87%) as a colorless oil: **^{1}H NMR** (400 MHz, Acetone-d_6) δ 7.46 – 7.38 (m, 3H), 5.40 – 5.25 (m, 2H), 5.16 – 5.14 (m, 1H), 5.09 (s, 2H), 4.52 – 4.48 (m, 1H), 4.33 – 4.26 (m, 3H), 4.06 (dd, J = 11.3, 4.0 Hz, 1H), 2.06 - 2.05 (m, 6H), 1.20 – 1.05 (m, 63H); **^{13}C NMR** (101 MHz, Acetone-d_6) δ 170.7, 170.5, 151.8, 137.4, 137.1, 135.4, 129.5, 129.4, 129.1, 101.3, 82.7, 70.3, 67.2, 65.9, 64.1, 62.8, 20.8(2), 18.6(2), 18.5(2), 18.4, 13.2, 13.1, 12.7; **HRMS** (ESI) *m/z* calcd for $C_{45}H_{82}O_8Si_3Na$ $[M + Na]^+$ 857.5210, found 857.5216.

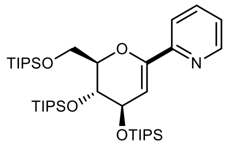

15n

2-((2*R*,3*R*,4*R*)-3,4-Bis((triisopropylsilyl)oxy)-2-(((triisopropylsilyl)oxy)methyl)-3,4-dihydro-2*H*-pyran-6-yl)pyridine (15n). According to the general procedure A, glycal boronates **12b** (95.0 mg, 0.12 mmol, 1.20 equiv), 2-bromopyridine (15.8 mg, 0.10 mmol, 1.00 equiv), $Pd(PPh_3)_2Cl_2$ (3.50 mg, 0.005 mmol, 5.00 mol%), K_3PO_4 (63.6 mg, 0.30 mmol, 3.00 equiv) were added to a one-dram vial with a screw-top septum, and the vial was then evacuated and refilled with N_2 (3×). Anhydrous DMF (2.00 mL) were added, and the reaction mixture was stirred at 33 °C for 48 h, cooled to rt, and concentrated. The crude material was purified by column chromatography on SiO_2 (Petroleum ether:EtOAc, 1:0-30:1) to afford **15n** (53.7 mg, 78%) as a light-yellow oil: **^{1}H NMR** (400 MHz, $CDCl_3$) δ 8.56 – 8.55 (m, 1H), 7.75 (d, J = 7.9 Hz, 1H), 7.68 – 7.64 (m, 1H), 7.19 – 7.16 (m, 1H), 6.13 (dd, J = 5.4, 1.6 Hz, 1H), 4.52 – 4.48 (m, 1H), 4.24 – 4.22 (m, 1H), 4.15 – 4.11 (m, 2H), 3.86 (dd, J = 11.3, 3.8 Hz, 1H), 1.10 – 1.00 (m, 63H); **^{13}C NMR** (101 MHz, $CDCl_3$) δ 153.3, 148.9, 148.6, 136.4, 123.0, 119.9, 99.1, 81.5, 70.5, 66.6, 61.9, 18.4, 18.3(2), 18.2, 18.1(2), 12.6, 12.5, 12.1; **HRMS** (ESI) *m/z* calcd for $C_{38}H_{73}NO_4Si_3Na$ $[M + Na]^+$ 714.4740, found 714.4734.

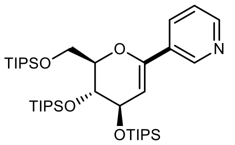

15o

3-((2*R*,3*R*,4*R*)-3,4-Bis((triisopropylsilyl)oxy)-2-(((triisopropylsilyl)oxy)methyl)-3,4-dihydro-2*H*-pyran-6-yl)pyridine (15o). According to the general procedure A, glycal boronates **12b** (95.0 mg, 0.12 mmol, 1.20 equiv), 3-bromopyridine (15.8 mg, 0.10 mmol, 1.00 equiv), $Pd(PPh_3)_2Cl_2$ (3.50 mg, 0.005 mmol, 5.00 mol%), K_3PO_4 (63.6 mg, 0.30 mmol, 3.00 equiv) were added to a one-dram vial with a screw-top septum, and the vial was then evacuated and refilled with N_2 (3×). Anhydrous DMF (2.00 mL) were added, and the reaction mixture was stirred at 33 °C for 48 h, cooled to rt, and concentrated. The crude material was purified by column chromatography on SiO_2 (Petroleum ether:EtOAc, 1:0-20:1) to afford **15o** (62.9 mg, 91%) as a colorless oil: **^{1}H NMR** (500 MHz, Acetone-*d*$_6$) δ 8.91 (d, *J* = 2.2 Hz, 1H), 8.53 (dd, *J* = 4.8, 1.7 Hz, 1H), 8.02 – 8.00 (m, 1H), 7.35 (dd, *J* = 7.9, 4.7 Hz, 1H), 5.65 (dd, *J* = 5.3, 1.6 Hz, 1H), 4.59 – 4.56 (m, 1H), 4.30 – 4.24 (m, 3H), 4.00 (dd, *J* = 11.4, 3.7 Hz, 1H), 1.21 – 1.05 (m, 63H); **^{13}C NMR** (126 MHz, Acetone-*d*$_6$) δ 150.4, 148.9, 147.5, 133.0, 132.3, 123.9, 98.7, 82.5, 70.8, 67.3, 62.7, 18.6, 18.6(2), 18.5, 18.4(3), 13.2(2), 12.7; **HRMS** (ESI) m/z calcd for $C_{38}H_{73}NO_4Si_3Na$ $[M + Na]^+$ 714.4740, found 714.4743.

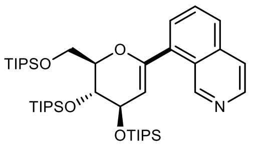

15p

8-((2*R*,3*R*,4*R*)-3,4-Bis((triisopropylsilyl)oxy)-2-(((triisopropylsilyl)oxy)methyl)-3,4-dihydro-2*H*-pyran-6-yl)isoquinoline (15p). According to the general procedure A, glycal boronates **12b** (95.0 mg, 0.12 mmol, 1.20 equiv), 8-bromoisoquinoline (20.8 mg, 0.10 mmol, 1.00 equiv), $Pd(PPh_3)_2Cl_2$ (3.50 mg, 0.005 mmol, 5.00 mol%), K_3PO_4 (63.6 mg, 0.30 mmol, 3.00 equiv) were added to a one-dram vial with a screw-top septum, and the vial was then evacuated and refilled with N_2 (3×). Anhydrous DMF (2.00 mL) were added, and the reaction mixture was stirred at 33 °C for 48 h, cooled to rt, and concentrated. The crude material was purified by column chromatography on SiO_2 (Petroleum ether:EtOAc, 1:0-10:1)to afford **15p** (64.7 mg, 87%) as a light-yellow oil: **^{1}H NMR** (400 MHz, $CDCl_3$) δ 9.72 (s, 1H), 8.54 (d, *J* = 5.7 Hz, 1H), 7.78 (dd, *J* = 7.8, 1.8 Hz, 1H), 7.66 – 7.59 (m, 3H), 5.21 (dd, *J* = 5.1, 2.0 Hz, 1H), 4.58 – 4.54 (m, 1H), 4.28 – 4.22 (m, 3H), 4.07 (dd, *J* = 11.1, 4.8 Hz, 1H), 1.16 – 1.09 (m, 42H), 1.07 – 1.01 (m, 21H); **^{13}C NMR** (101 MHz, $CDCl_3$) δ 151.6, 150.4, 143.0, 136.1, 136.0, 129.7, 127.6, 127.3, 126.6, 120.4, 102.1, 82.2, 69.4, 66.3, 62.1, 18.3(4), 18.1, 12.6, 12.5, 12.1; **HRMS** (ESI) *m/z* calcd for $C_{42}H_{76}NO_4SSi_3$ $[M + H]^+$ 742.5077, found 742.5082.

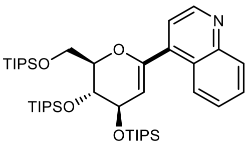

15q

4-((2*R*,3*R*,4*R*)-3,4-Bis((triisopropylsilyl)oxy)-2-(((triisopropylsilyl)oxy)methyl)-3,4-dihydro-2*H*-pyran-6-yl)quinoline (15q). According to the general procedure A, glycal boronates **12b** (95.0 mg, 0.12 mmol, 1.20 equiv), 4-bromoquinoline (20.8 mg, 0.10 mmol, 1.00 equiv), $Pd(PPh_3)_2Cl_2$ (3.50 mg, 0.005 mmol, 5.00 mol%), K_3PO_4 (63.6 mg, 0.30 mmol, 3.00 equiv) were added to a one-dram vial with a screw-top septum, and the vial was then evacuated and refilled with N_2 (3×). Anhydrous DMF (2.00 mL) were added, and the reaction mixture was stirred at 33 °C for 48 h, cooled to rt, and concentrated. The crude material was purified by column chromatography on SiO_2 (Petroleum ether:Ether, 1:0-50:1) to afford **15q** (71.6 mg, 96%) as a light-yellow oil: **^{1}H NMR** (400 MHz, $CDCl_3$) δ 8.89 (d, *J* = 4.4 Hz, 1H), 8.43 (dd, *J* = 8.6, 1.4 Hz, 1H), 8.10 (dd, *J* = 8.5, 1.2 Hz, 1H), 7.72 – 7.68 (m, 1H), 7.52 – 7.48 (m, 1H), 7.38 (d, *J* = 4.4 Hz, 1H), 5.23 (dd, *J* = 5.3, 1.8 Hz, 1H), 4.58 – 4.54 (m, 1H), 4.30 (dd, *J* = 11.5, 7.9 Hz, 1H), 4.24 – 4.21 (m, 2H), 4.01 (dd, *J* = 11.4, 4.0 Hz, 1H), 1.15 – 1.02 (m, 63H); **^{13}C NMR** (101 MHz, $CDCl_3$) δ 150.3, 150.1, 148.6, 143.4, 129.6, 129.4, 126.6(2), 126.4, 120.4, 102.3, 82.4, 69.6, 66.1, 62.1, 18.3(3), 18.1, 12.6, 12.5, 12.1; **HRMS** (ESI) *m/z* calcd for $C_{42}H_{76}NO_4Si_3$ $[M + H]^+$ 742.5077, found 742.5081.

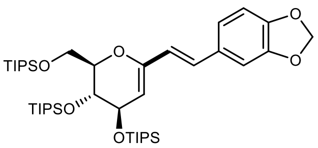

15r

(((2*R*,3*R*,4*R*)-6-((*E*)-2-(Benzo[*d*][1,3]dioxol-5-yl)vinyl)-2-(((triisopropylsilyl)oxy)methyl)-3,4-dihydro-2*H*-pyran-3,4-diyl)bis(oxy))bis(triisopropylsilane) (15r). According to the general procedure A, glycal boronates **12b** (95.0 mg, 0.12 mmol, 1.20 equiv), (*E*)-5-(2-bromovinyl)benzo[*d*][1,3]dioxole[11] (22.6 mg, 0.10 mmol, 1.00 equiv), $Pd(PPh_3)_2Cl_2$ (3.50 mg, 0.005 mmol, 5.00 mol%), K_3PO_4 (63.6 mg, 0.30 mmol, 3.00 equiv) were added to a one-dram vial with a screw-top septum, and the vial was then evacuated and refilled with N_2 (3×). Anhydrous DMF (2.00 mL) were added, and the reaction mixture was stirred at 33 °C for 48 h, cooled to rt, and concentrated. The crude material was purified by column chromatography on SiO_2 (Petroleum ether:Ether, 1:0 - 30:1) to afford **15r** (60.8 mg, 80%) as a colorless oil: **^{1}H NMR** (400 MHz, Acetone-d_6) δ 7.09 (d, *J* = 1.7 Hz, 1H), 6.97 – 6.91 (m, 2H), 6.81 (d, *J* = 8.0 Hz, 1H), 6.53 (d, *J* = 15.8 Hz, 1H), 6.00 (s, 2H), 5.10 – 5.08 (m, 1H), 4.48 – 4.44 (m, 1H), 4.21 (d, *J* = 2.4 Hz, 2H), 4.16 (dd, *J* = 11.2, 7.9 Hz, 1H), 3.95 (dd, *J* = 11.2, 3.7 Hz, 1H), 1.16 – 1.07 (m, 63H); **^{13}C NMR** (101 MHz, Acetone-d_6) δ 150.3, 149.2, 148.4, 132.3, 129.8, 123.5, 122.7, 109.1, 106.2, 102.3, 102.1, 82.0, 71.2, 67.7, 63.1, 18.6(2), 18.5(2), 18.4(2), 13.3, 13.2, 12.8; **HRMS** (ESI) m/z calcd for $C_{42}H_{77}O_6Si_3$ $[M + H]^+$ 761.5022, found 761.5036.

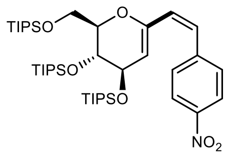

15s

(((2*R*,3*R*,4*R*)-6-((*Z*)-4-Nitrostyryl)-2-(((triisopropylsilyl)oxy)methyl)-3,4-dihydro-2*H*-pyran-3,4-diyl)bis(oxy))bis(triisopropylsilane) (15s). According to the general procedure A, glycal boronates **12b** (95.0 mg, 0.12 mmol, 1.20 equiv), (*Z*)-1-(2-bromovinyl)-4-nitrobenzene[12] (20.8 mg, 0.10 mmol, 1.00 equiv), $Pd(PPh_3)_2Cl_2$ (3.50 mg, 0.005 mmol, 5.00 mol%) , K_3PO_4 (63.6 mg, 0.30 mmol, 3.00 equiv) were added to a one-dram vial with a screw-top septum, and the vial was then evacuated and refilled with N_2 (3×). Anhydrous DMF (2.00 mL) were added, and the reaction mixture was stirred at 33 °C for 48 h, cooled to rt, and concentrated. The crude material was purified by column chromatography on SiO_2 (Petroleum ether:Ether, 1:0-50:1) to afford **15q** (71.6 mg, 96%) as a light-yellow oil: **^{1}H NMR** (500 MHz, Acetone-*d*$_6$) δ 8.23 – 8.20 (m, 2H), 7.78 – 7.75 (m, 2H), 7.12 (d, *J* = 15.8 Hz, 1H), 6.97 (d, *J* = 15.9 Hz, 1H), 5.32 (dd, *J* = 5.1, 2.0 Hz, 1H), 4.52 – 4.49 (m, 1H), 4.25 – 4.22 (m, 2H), 4.18 (dd, *J* = 11.4, 8.0 Hz, 1H), 3.97 (dd, *J* = 11.3, 3.7 Hz, 1H), 1.15 – 1.07 (m, 63H); **^{13}C NMR** (126 MHz, Acetone-*d*$_6$) δ 149.7, 147.8, 144.5, 129.8, 128.2, 127.7, 124.8, 105.8, 82.2, 71.0, 67.5, 62.9, 18.6(2), 18.5(2), 18.4(2), 13.3, 13.2, 12.8; **HRMS** (ESI) m/z calcd for $C_{41}H_{75}NO_6Si_3Na$ $[M + Na]^+$ 784.4794, found 784.4801.

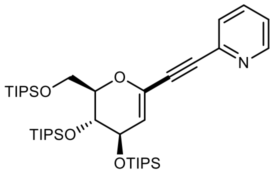

15t

2-(((2*R*,3*R*,4*R*)-3,4-bis((triisopropylsilyl)oxy)-2-(((triisopropylsilyl)oxy)methyl)-3,4-dihydro-2*H*-pyran-6-yl)ethynyl)pyridine (15t). According to the general procedure A, glycal boronates **12b** (95.0 mg, 0.12 mmol, 1.20 equiv), 2-(bromoethynyl)pyridine[13] (18.1 mg, 0.10 mmol, 1.00 equiv), $Pd(PPh_3)_2Cl_2$ (3.50 mg, 0.005 mmol, 5.00 mol%), K_3PO_4 (63.6 mg, 0.30 mmol, 3.00 equiv) were added to a one-dram vial with a screw-top septum, and the vial was then evacuated and refilled with N_2 (3×). Anhydrous DMF (2.00 mL) were added, and the reaction mixture was stirred at 33 °C for 48 h, cooled to rt, and concentrated. The crude material was purified by column chromatography on SiO_2 (Petroleum ether:EtOAc, 1:0-10:1) to afford **15t** (46.1 mg, 64%) as a light-yellow oil: **^{1}H NMR** (400 MHz, Acetone-*d*$_6$) δ 8.68 – 8.51 (m, 1H), 7.85 – 7.80 (m, 1H), 7.55 (d, *J* = 7.8 Hz, 1H), 7.39 (dd, *J* = 7.7, 4.9 Hz, 1H), 5.52 (dd, *J* = 5.4, 1.6 Hz, 1H), 4.44 – 4.40 (m, 1H), 4.24 (d, *J* = 2.1 Hz, 1H), 4.18 – 4.13 (m, 2H), 3.99 (dd, *J* = 11.4, 4.1 Hz, 1H), 1.15 – 1.09 (m, 63H); **^{13}C NMR** (101 MHz, Acetone-*d*$_6$) δ 151.1, 143.2, 137.3, 136.9, 128.2, 124.4, 109.1, 87.5, 84.6, 82.6, 70.3, 66.7, 62.6, 18.6, 18.5(2), 18.4(2), 13.2, 13.1, 12.8; **HRMS** (ESI) *m/z* calcd for

$C_{40}H_{74}NO_4Si_3$ [M + H]$^+$ 716.4920, found 716.4924.

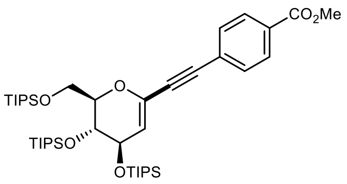

15u

Methyl 4-(((2*R*,3*R*,4*R*)-3,4-bis((triisopropylsilyl)oxy)-2-(((triisopropylsilyl)oxy)methyl)-3,4-dihydro-2*H*-pyran-6-yl)ethynyl)benzoate (15u). According to the general procedure A, glycal boronates **12b** (95.0 mg, 0.12 mmol, 1.20 equiv), methyl 4-(bromoethynyl)benzoate[13] (23.8 mg, 0.10 mmol, 1.00 equiv), $Pd(PPh_3)_2Cl_2$ (3.50 mg, 0.005 mmol, 5.00 mol%), K_3PO_4 (63.6 mg, 0.30 mmol, 3.00 equiv) were added to a one-dram vial with a screw-top septum, and the vial was then evacuated and refilled with N_2 (3×). Anhydrous DMF (2.00 mL) were added, and the reaction mixture was stirred at 33 °C for 48 h, cooled to rt, and concentrated. The crude material was purified by column chromatography on SiO_2 (Petroleum ether: EtOAc, 1:0 - 50:1) to afford **15u** (60.2 mg, 78%) as a yellow oil: **^{1}H NMR** (400 MHz, Acetone-*d*6) δ 8.03 (d, *J* = 8.0 Hz, 2H), 7.62 (d, *J* = 8.0 Hz, 2H), 5.50 (d, *J* = 5.3 Hz, 1H), 4.43 – 4.40 (m, 1H), 4.23 – 4.14 (m, 3H), 3.98 (dd, *J* = 11.4, 3.8 Hz, 1H), 3.90 (s, 3H), 1.11 (dd, *J* = 6.8, 4.2 Hz, 63H); **^{13}C NMR** (101 MHz, Acetone-*d*6) δ 166.5, 136.9, 132.5, 131.2, 130.3, 127.5, 108.8, 88.6, 87.0, 82.6, 70.4, 66.7, 62.7, 52.6, 18.6, 18.5(3), 18.4, 13.2, 13.1, 12.8; **HRMS** (ESI) *m/z* calcd for $C_{43}H_{77}O_6Si_3$ [M + H]$^+$ 773.5022, found 773.5025.

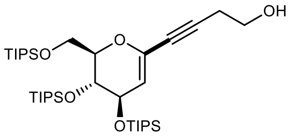

15v

4-((2*R*,3*R*,4*R*)-3,4-Bis((triisopropylsilyl)oxy)-2-(((triisopropylsilyl)oxy)methyl)-3,4-dihydro-2*H*-pyran-6-yl)but-3-yn-1-ol (15v). According to the general procedure A, glycal boronates **12b** (95.0 mg, 0.12 mmol, 1.20 equiv), 4-bromobut-3-yn-1-ol[14] (14.8 mg, 0.10 mmol, 1.00 equiv), $Pd(PPh_3)_2Cl_2$ (3.50 mg, 0.005 mmol, 5.00 mol%), K_3PO_4 (63.6 mg, 0.30 mmol, 3.00 equiv) were added to a one-dram vial with a screw-top septum, and the vial was then evacuated and refilled with N_2 (3×). Anhydrous DMF (2.00 mL) were added, and the reaction mixture was stirred at 33 °C for 48 h, cooled to rt, and concentrated. The crude material was purified by column chromatography on SiO_2 (Petroleum ether:EtOAc, 1:0 - 10:1) to afford **15v** (29.4 mg, 43%) as a light-yellow oil: **^{1}H NMR** (400 MHz, Acetone-*d*6) δ 5.18 (dd, *J* = 5.4, 1.6 Hz, 1H), 4.34 – 4.30 (m, 1H), 4.18 – 4.16 (m, 1H), 4.10 – 4.04 (m, 2H), 3.97 – 3.93 (m, 2H), 3.66 (d, *J* = 6.6 Hz, 2H), 2.51 (t, *J* = 7.0, 7.0 Hz, 2H), 1.12 – 1.08 (m, 63H); **^{13}C NMR** (101 MHz, Acetone-*d*6) δ 137.5, 106.2, 86.8, 82.2, 78.0, 70.4, 66.9, 62.7, 61.1, 61.0, 23.9, 18.6, 18.5(3), 18.4, 13.2, 13.1, 12.8; **HRMS** (ESI) *m/z* calcd for $C_{37}H_{75}O_5Si_3Na$ [M + Na]$^+$

705.4736, found 705.4744.

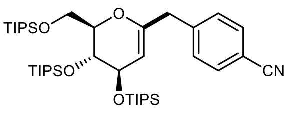

15w

4-(((2*R*,3*R*,4*R*)-3,4-Bis((triisopropylsilyl)oxy)-2-(((triisopropylsilyl)oxy)methyl)-3,4-dihydro-2*H*-pyran-6-yl)methyl)benzonitrile (15w). According to the general procedure A, glycal boronates **12b** (95.0 mg, 0.12 mmol, 1.20 equiv), α-bromo-*p*-tolunitrile (19.6 mg, 0.10 mmol, 1.00 equiv), $Pd(PPh_3)_2Cl_2$ (3.50 mg, 0.005 mmol, 5.00 mol%), K_3PO_4 (63.6 mg, 0.30 mmol, 3.00 equiv) were added to a one-dram vial with a screw-top septum, and the vial was then evacuated and refilled with N_2 (3×). Anhydrous DMF (2.00 mL) were added, and the reaction mixture was stirred at 33 °C for 48 h, cooled to rt, and concentrated. The crude material was purified by column chromatography on SiO_2 (Petroleum ether:EtOAc, 1:0-50:1) to afford **15q** (50.4 mg, 69%) as a light-yellow oil: **^{1}H NMR** (400 MHz, Acetone-d_6) δ 7.68 – 7.64 (m, 2H), 7.54 – 7.49 (m, 2H), 4.94 (dd, *J* = 5.4, 1.5 Hz, 1H), 4.31 – 4.27 (m, 1H), 4.14 – 4.09 (m, 2H), 4.01 (dd, *J* = 11.2, 7.6 Hz, 1H), 3.91 (dd, *J* = 11.2, 4.0 Hz, 1H), 3.51 (d, *J* = 1.5 Hz, 2H), 1.11 – 1.05 (m, 63H);**^{13}C NMR** (101 MHz, Acetone-d_6) δ 152.3, 145.3, 132.7, 130.6, 119.5, 110.9, 98.8, 82.4, 70.4, 67.2, 63.1, 41.3, 18.6(2), 18.5, 18.4(3), 13.1(2), 12.7; **HRMS** (ESI) *m/z* calcd for $C_{41}H_{75}NO_4Si_3Na$ $[M + Na]^+$ 752.4896, found 752.4888.

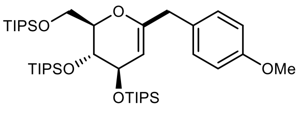

15x

(((2*R*,3*R*,4*R*)-6-(4-Methoxybenzyl)-2-(((triisopropylsilyl)oxy)methyl)-3,4-dihydro-2*H*-pyran-3,4-diyl)bis(oxy))bis(triisopropylsilane) (15x). According to the general procedure A, glycal boronates **12b** (95.0 mg, 0.12 mmol, 1.20 equiv), 4-methoxybenzyl bromide (20.1 mg, 0.10 mmol, 1.00 equiv), $Pd(PPh_3)_2Cl_2$ (3.50 mg, 0.005 mmol, 5.00 mol%), K_3PO_4 (63.6 mg, 0.30 mmol, 3.00 equiv) were added to a one-dram vial with a screw-top septum, and the vial was then evacuated and refilled with N_2 (3×). Anhydrous DMF (2.00 mL) were added, and the reaction mixture was stirred at 33 °C for 48 h, cooled to rt, and concentrated. The crude material was purified by column chromatography on SiO_2 (Petroleum ether:ether, 1:0-50:1) to afford **15q** (71.6 mg, 96%) as a light-yellow oil: **^{1}H NMR** (500 MHz, Acetone-d_6) δ 7.19 – 7.17 (m, 2H), 6.82 – 6.79 (m, 2H), 4.74 (dd, *J* = 5.3, 1.5 Hz, 1H), 4.29 – 4.26 (m, 1H), 4.15 – 4.13 (m, 1H), 4.09 – 4.07 (m, 1H), 4.00 (dd, *J* = 11.1, 7.3 Hz, 1H), 3.93 (dd, *J* = 11.1, 4.3 Hz, 1H), 3.75 (s, 3H), 3.31 (s, 2H), 1.14 – 1.02 (m, 63H); **^{13}C NMR** (126 MHz, Acetone-d_6) δ 159.2, 154.1, 131.0, 130.6, 114.3, 97.8, 82.1, 70.6, 67.5, 63.2, 55.4, 40.4, 18.6(2), 18.5(2), 18.4, 13.2(2), 12.8; **HRMS** (ESI) *m/z* calcd for $C_{41}H_{78}O_5Si_3Na$ $[M + Na]^+$ 757.5055, found 757.5057.

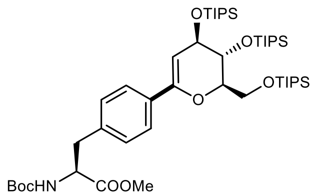

17a

Methyl (*S*)-3-(4-((2*R*,3*R*,4*R*)-3,4-bis((triisopropylsilyl)oxy)-2-(((triisopropylsilyl)oxy)methyl)-3,4-dihydro-2*H*-pyran-6-yl)phenyl)-2-((*tert*-butoxycarbonyl)amino)propanoate (17a). According to the general procedure A, glycal boronates **12b** (95.0 mg, 0.12 mmol, 1.20 equiv), methyl (*S*)-3-(4-bromophenyl)-2-((*tert*-butoxycarbonyl)amino)propanoate (35.7 mg, 0.10 mmol, 1.00 equiv), $Pd(PPh_3)_2Cl_2$ (3.50 mg, 0.005 mmol, 5.00 mol%), K_3PO_4 (63.6 mg, 0.30 mmol, 3.00 equiv) were added to a one-dram vial with a screw-top septum, and the vial was then evacuated and refilled with N_2 (3×). Anhydrous DMF (2.00 mL) were added, and the reaction mixture was stirred at 33 °C for 48 h, cooled to rt, and concentrated. The crude material was purified by column chromatography on SiO_2 (Petroleum ether:Ether, 1:0 -50:1) to afford **17a** (83.3 mg, 94%) as a light-yellow oil: **^{1}H NMR** (400 MHz, $CDCl_3$) δ 7.56 (d, *J* = 8.1 Hz, 2H), 7.07 (d, *J* = 7.9 Hz, 2H), 5.32 (dd, *J* = 5.3, 1.5 Hz, 1H), 4.97 (d, *J* = 8.3 Hz, 1H), 4.61 – 4.56 (m, 1H), 4.47 – 4.43 (m, 1H), 4.18 – 4.07 (m, 3H), 3.89 (dd, *J* = 11.2, 4.2 Hz, 1H), 3.70 (s, 3H), 3.14 – 3.03 (m, 2H), 1.42 (s, 9H), 1.10 – 1.00 (m, 63H); **^{13}C NMR** (101 MHz, $CDCl_3$) δ 172.5, 155.3, 150.1, 136.1, 135.3, 129.1, 125.7, 96.8, 81.4, 80.1, 70.2, 66.8, 62.1, 54.5, 52.3, 38.1, 28.4, 18.3(3), 18.2, 18.1(2), 12.7, 12.6, 12.1; **HRMS** (ESI) *m/z* calcd for $C_{48}H_{90}NO_8Si_3$ $[M + H]^+$ 892.5969, found 892.5974.

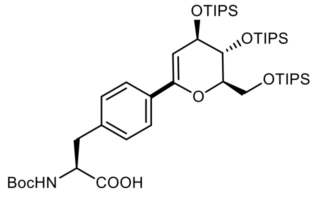

17b

(*S*)-3-(4-((2*R*,3*R*,4*R*)-3,4-Bis((triisopropylsilyl)oxy)-2-(((triisopropylsilyl)oxy)methyl)-3,4-dihydro-2*H*-pyran-6-yl)phenyl)-2-((*tert*-butoxycarbonyl)amino)propanoic acid (17b). According to the general procedure A, glycal boronates **12b** (95.0 mg, 0.12 mmol, 1.20 equiv), (*S*)-3-(4-bromophenyl)-2-((*tert*-butoxycarbonyl)amino)propanoic acid (34.4 mg, 0.10 mmol, 1.00 equiv), $Pd(PPh_3)_2Cl_2$ (3.50 mg, 0.005 mmol, 5.00 mol%), K_3PO_4 (63.6 mg, 0.30 mmol, 3.00 equiv) were added to a one-dram vial with a screw-top septum, and the vial was then evacuated and refilled with N_2 (3×). Anhydrous DMF (2.00 mL) were added, and the reaction mixture was stirred at 33 °C for 48 h, cooled to rt, and concentrated. The crude material was purified by column chromatography on SiO_2 (Petroleum ether:EtOAc, 1:0-2:1) to afford **17b** (71.6 mg, 96%) as a light-yellow oil: **^{1}H NMR** (400 MHz, Acetone-d_6) δ 7.63 (d, *J* = 8.0 Hz, 2H), 7.28 (d, *J* = 8.0 Hz, 2H), 6.08 (d, *J* = 8.5 Hz,

1H), 5.50 (dd, J = 5.3, 1.5 Hz, 1H), 4.54 – 4.50 (m, 1H), 4.46 – 4.40 (m, 1H), 4.28 – 4.19 (m, 3H), 4.00 (dd, J = 11.2, 3.9 Hz, 1H), 3.22 (dd, J = 13.9, 5.0 Hz, 1H), 3.05 – 2.91 (m, 2H), 1.35 (s, 9H), 1.18 – 1.05 (m, 63H); **^{13}C NMR** (101 MHz, Acetone-d_6) δ 173.5, 156.2, 151.0, 139.0, 135.3, 129.9, 126.0, 97.0, 82.2, 79.2, 71.0, 67.7, 62.8, 55.6, 37.8, 28.5, 18.6(2), 18.5(2), 18.4(2), 13.3, 13.2, 12.8; **HRMS** (ESI) *m/z* calcd for $C_{47}H_{88}NO_8Si_3$ $[M + H]^+$ 878.5812, found 878.5842.

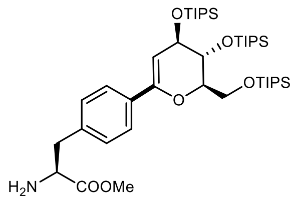

17c

Methyl (*S*)-2-amino-3-(4-((2*R*,3*R*,4*R*)-3,4-bis((triisopropylsilyl)oxy)-2-(((triisopropylsilyl)oxy)methyl)-3,4-dihydro-2*H*-pyran-6-yl)phenyl)propanoate (17c). According to the general procedure A, glycal boronates **12b** (95.0 mg, 0.12 mmol, 1.20 equiv), methyl (2*S*)-2-amino-3-(4-bromophenyl)propanoate hydrochloride (29.4 mg, 0.10 mmol, 1.00 equiv), $Pd(PPh_3)_2Cl_2$ (3.50 mg, 0.005 mmol, 5.00 mol%), K_3PO_4 (84.8 mg, 0.40 mmol, 4.00 equiv) were added to a one-dram vial with a screw-top septum, and the vial was then evacuated and refilled with N_2 (3×). Anhydrous DMF (2.00 mL) were added, and the reaction mixture was stirred at 33 °C for 48 h, cooled to rt, and concentrated. The crude material was purified by column chromatography on SiO_2 (Petroleum ether:EtOAc, 1:0-3:1) to afford **17c** (45.9 mg, 58%) as a light-yellow oil: **^{1}H NMR** (500 MHz, Acetone-d_6) δ 7.60 (d, J = 8.3 Hz, 2H), 7.19 – 7.18 (m, 2H), 5.49 (dd, J = 5.3, 1.6 Hz, 1H), 4.54 – 4.51 (m, 1H), 4.35 – 4.19 (m, 4H), 3.97 (dd, J = 11.3, 3.6 Hz, 1H), 3.62 (s, 3H), 3.21 (dd, J = 13.3, 5.3 Hz, 1H), 2.90 (dd, J = 13.4, 8.3 Hz, 1H), 1.90 – 1.87 (m, 1H), 1.57 – 1.51 (m, H),1.16 – 1.04 (m, 63H); **^{13}C NMR** (126 MHz, Acetone-d_6) δ 172.6, 150.9, 139.9, 134.9, 130.2, 125.8, 96.8, 82.3, 71.0, 67.7, 65.9, 62.8, 51.9, 39.7, 18.6(2), 18.5, 18.4(3), 13.3, 13.2, 12.7; **HRMS** (ESI) *m/z* calcd for $C_{43}H_{82}NO_6Si_3$ $[M + H]^+$ 792.5444, found 792.5451.

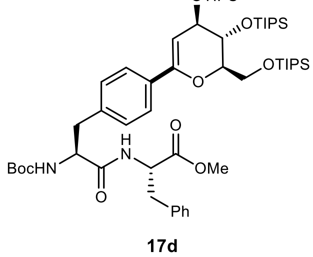

17d

Methyl ((*S*)-3-(4-((2*R*,3*R*,4*R*)-3,4-bis((triisopropylsilyl)oxy)-2-(((triisopropylsilyl)oxy)methyl)-3,4-dihydro-2*H*-pyran-6-yl)phenyl)-2-((*tert*-butoxycarbonyl)amino)propanoyl)-*L*-phenylalaninate (17d). According to the general procedure A, glycal boronates **12b** (95.0 mg, 0.12 mmol, 1.20 equiv), methyl ((*S*)-3-(4-bromophenyl)-2-((*tert*-butoxycarbonyl)amino)propanoyl)-*L*-phenylalaninate[15] (50.4 mg, 0.10 mmol, 1.00 equiv), $Pd(PPh_3)_2Cl_2$ (3.50 mg, 0.005

mmol, 5.00 mol%), K_3PO_4 (63.6 mg, 0.30 mmol, 3.00 equiv) were added to a one-dram vial with a screw-top septum, and the vial was then evacuated and refilled with N_2 (3×). Anhydrous DMF (2.00 mL) were added, and the reaction mixture was stirred at 33 °C for 48 h, cooled to rt, and concentrated. The crude material was purified by column chromatography on SiO_2 (Petroleum ether:EtOAc, 1:0-4:1) to afford **17d** (82.5 mg, 79%) as a colorless oil: **^{1}H NMR** (400 MHz, Acetone-*d*$_6$) δ 7.65 – 7.57 (m, 2H), 7.47 (d, *J* = 7.9 Hz, 1H), 7.30 – 7.19 (m, 7H), 6.04 (d, *J* = 8.5 Hz, 1H), 5.48 (dd, *J* = 5.3, 1.5 Hz, 1H), 4.77 – 4.71 (m, 1H), 4.54 – 4.50 (m, 1H), 4.42 – 4.37 (m, 1H), 4.29 – 4.24 (m, 2H), 4.21 (dd, *J* = 11.2, 7.9 Hz, 1H), 4.00 (dd, *J* = 11.2, 4.0 Hz, 1H), 3.65 (s, 3H), 3.16 – 3.11 (m, 2H), 3.04 (dd, *J* = 13.8, 7.3 Hz, 1H), 2.90 (dd, *J* = 14.0, 9.0 Hz, 1H), 1.33 (s, 9H), 1.16 – 1.06 (m, 63H); **^{13}C NMR** (101 MHz, Acetone-*d*$_6$) δ 172.4, 172.0, 156.2, 151.1, 139.2, 137.7, 135.1, 130.2, 130.0, 129.2, 127.5, 126.0, 96.9, 82.3, 79.4, 71.0, 67.7, 62.9, 56.5, 54.4, 52.3, 38.4, 28.5, 18.7, 18.6, 18.5(2), 18.4(2), 13.3, 13.2, 12.8; **HRMS** (ESI) *m/z* calcd for $C_{57}H_{99}N_2O_9Si_3$ $[M + H]^+$ 1039.6653, found 1039.6654.

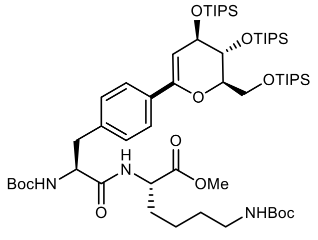

17e

Methyl *N*^{2}-((*S*)-3-(4-((2*R*,3*R*,4*R*)-3,4-bis((triisopropylsilyl)oxy)-2-(((triisopropylsilyl)oxy)methyl)-3,4-dihydro-2*H*-pyran-6-yl)phenyl)-2-((*tert*-butoxycarbonyl)amino)propanoyl)-*N*^{6}-(*tert*-butoxycarbonyl)-*L*-lysinate (17e). According to the general procedure A, glycal boronates **12b** (95.0 mg, 0.12 mmol, 1.20 equiv), methyl *N*^{2}-((*S*)-3-(4-bromophenyl)-2-((*tert*-butoxycarbonyl)amino)propanoyl)-*N*^{6}-(*tert*-butoxycarbonyl)-*L*-lysinate **16e** (58.5 mg, 0.10 mmol, 1.00 equiv), $Pd(PPh_3)_2Cl_2$ (3.50 mg, 0.005 mmol, 5.00 mol%), K_3PO_4 (63.6 mg, 0.30 mmol, 3.00 equiv) were added to a one-dram vial with a screw-top septum, and the vial was then evacuated and refilled with N_2 (3×). Anhydrous DMF (2.00 mL) were added, and the reaction mixture was stirred at 33 °C for 48 h, cooled to rt, and concentrated. The crude material was purified by column chromatography on SiO_2 (Petroleum ether:EtOAc, 1:0-4:1) to afford **17e** (108 mg, 91%) as a colorless oil: **^{1}H NMR** (500 MHz, Acetone-*d*$_6$) δ 7.61 (d, *J* = 8.2 Hz, 2H), 7.52 (d, *J* = 8.0 Hz, 1H), 7.26 (d, *J* = 7.9 Hz, 2H), 6.07 (d, *J* = 8.5 Hz, 1H), 5.91 (d, *J* = 6.0 Hz, 1H), 5.48 (dd, *J* = 5.4, 1.5 Hz, 1H), 4.54 – 4.46 (m, 2H), 4.43 – 4.39 (m, 1H), 4.29 – 4.24 (m, 2H), 4.21 (dd, *J* = 11.2, 7.9 Hz, 1H), 4.01 (dd, *J* = 11.3, 4.0 Hz, 1H), 3.68 (s, 3H), 3.20 (dd, *J* = 14.0, 5.1 Hz, 1H), 3.05 (q, *J* = 6.7, 6.6, 6.6 Hz, 2H), 2.94 (dd, *J* = 14.0, 8.9 Hz, 1H), 1.86 – 1.80 (m, 1H), 1.73 – 1.66 (m, 1H), 1.51 – 1.46 (m, 2H), 1.43 – 1.39 (m, 11H), 1.34 (s, 9H), 1.18 – 1.05 (m, 63H); **^{13}C NMR** (126 MHz, Acetone-*d*$_6$) δ 173.2, 172.2, 156.7, 156.2, 151.1, 139.2, 135.1, 130.0, 125.9, 96.9, 82.2, 79.3, 78.3, 71.0, 67.7, 62.9, 56.4, 52.9, 52.3, 40.8, 38.4, 32.3, 28.7, 28.5, 23.4, 18.7, 18.6, 18.5(2), 18.4(2), 13.3, 13.2, 12.8; **HRMS** (ESI) *m/z* calcd for $C_{59}H_{109}N_3O_{11}Si_3Na$ $[M + Na]^+$ 1142.7262, found 1142.7260.

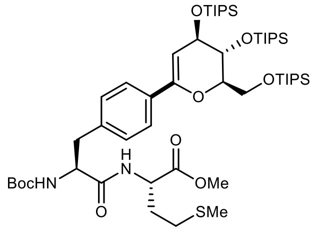

17f

Methyl ((*S*)-3-(4-((2*R*,3*R*,4*R*)-3,4-bis((triisopropylsilyl)oxy)-2-(((triisopropylsilyl)oxy)methyl)-3,4-dihydro-2*H*-pyran-6-yl)phenyl)-2-((*tert*-butoxycarbonyl)amino)propanoyl)-*L*-methioninate (17f). According to the general procedure A, glycal boronates **12b** (95.0 mg, 0.12 mmol, 1.20 equiv), methyl ((*S*)-3-(4-bromophenyl)-2-((*tert*-butoxycarbonyl)amino)propanoyl)-L-methioninate **16f** (48.80 mg, 0.10 mmol, 1.00 equiv), $Pd(PPh_3)_2Cl_2$ (35.00 mg, 0.05 mmol, 50.0 mol%), K_3PO_4 (63.60 mg, 0.30 mmol, 3.00 equiv) were added to a one-dram vial with a screw-top septum, and the vial was then evacuated and refilled with N_2 (3×). Anhydrous DMF (2.00 mL) were added, and the reaction mixture was stirred at 33 °C for 48 h, cooled to rt, and concentrated. The crude material was purified by column chromatography on SiO_2 (Petroleum ether:EtOAc, 1:0-3:1) to afford **17f** (58.5 mg, 57%) as a light-yellow foam: **^{1}H NMR** (400 MHz, Acetone-*d*$_6$) δ 7.63 – 7.56 (m, 3H), 7.26 (d, *J* = 7.9 Hz, 2H), 6.14 – 6.06 (m, 1H), 5.49 (dd, *J* = 5.4, 1.5 Hz, 1H), 4.67 – 4.58 (m, 1H), 4.54 – 4.50 (m, 1H), 4.42 – 4.36 (m, 1H), 4.29 – 4.18 (m, 3H), 4.01 (dd, *J* = 11.3, 3.9 Hz, 1H), 3.69 (s, 3H), 3.20 – 3.13 (m, 1H), 2.96 (dd, *J* = 13.8, 8.7 Hz, 1H), 2.58 – 2.42 (m, 2H), 2.06 (s, 3H), 2.00 – 1.92 (m, 1H), 1.34 (s, 9H), 1.16 – 1.06 (m, 63H); **^{13}C NMR** (101 MHz, Acetone-*d*$_6$) δ 172.8, 172.2, 156.2, 151.0, 139.2, 135.1, 130.1, 126.0(2), 96.9, 82.2, 79.3, 71.0, 67.7, 62.8, 56.5, 52.5, 52.4, 52.1, 38.2, 32.2, 28.5, 18.6(2), 18.5(2), 18.4(2), 15.2, 13.2(2), 12.7; **HRMS** (ESI) *m/z* calcd for $C_{53}H_{99}N_2O_9Si_3S$ [M + H]$^+$ 1023.6374, found 1023.6362.

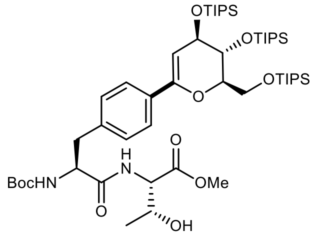

17g

Methyl ((*S*)-3-(4-((2*R*,3*R*,4*R*)-3,4-bis((triisopropylsilyl)oxy)-2-(((triisopropylsilyl)oxy)methyl)-3,4-dihydro-2*H*-pyran-6-yl)phenyl)-2-((*tert*-butoxycarbonyl)amino)propanoyl)-*L*-allothreoninate (17g). According to the general procedure A, glycal boronates **12b** (95.0 mg, 0.12 mmol, 1.20 equiv), methyl ((*S*)-3-(4-bromophenyl)-2-((*tert*-butoxycarbonyl)amino)propanoyl)-*L*-threoninate **16g** (45.8 mg, 0.10 mmol, 1.00 equiv), $Pd(PPh_3)_2Cl_2$ (35.00 mg, 0.050 mmol, 50.0 mol%), K_3PO_4 (63.60 mg, 0.30 mmol, 3.00 equiv) were added to a one-dram vial with a screw-top septum, and the vial was then evacuated and refilled with N_2 (3×). Anhydrous DMF

(2.00 mL) were added, and the reaction mixture was stirred at 33 °C for 48 h, cooled to rt, and concentrated. The crude material was purified by column chromatography on SiO_2 (Petroleum ether:EtOAc, 1:0-1.5:1) to afford **17g** (52.0 mg, 52%) as a light-yellow oil: **^{1}H NMR** (400 MHz, Acetone-d_6) δ 7.61 (d, *J* = 8.0 Hz, 2H), 7.40 (d, *J* = 9.1 Hz, 1H), 7.30 (d, *J* = 7.9 Hz, 2H), 6.23 (d, *J* = 8.6 Hz, 1H), 5.50 – 5.48 (m, 1H), 4.54 – 4.47 (m, 3H), 4.34 – 4.15 (m, 5H), 4.00 (dd, *J* = 11.2, 4.0 Hz, 1H), 3.68 (s, 3H), 3.26 (dd, *J* = 14.0, 4.8 Hz, 1H), 2.98 (dd, *J* = 14.0, 9.3 Hz, 1H), 1.34 (s, 9H), 1.20 – 1.06 (m, 66H); **^{13}C NMR** (101 MHz, Acetone-d_6) δ 172.6, 171.8, 156.2, 151.1, 139.4, 135.1, 130.1, 125.9, 96.9, 82.2, 79.4, 71.0, 68.1, 67.7, 62.8, 58.5, 56.6, 52.3, 38.1, 28.5, 18.6(2), 18.5(2), 18.4(2), 13.2(2), 12.7; **HRMS** (ESI) *m/z* calcd for $C_{52}H_{97}N_2O_{10}Si_3$ $[M + H]^+$ 993.6430 found 993.6430.

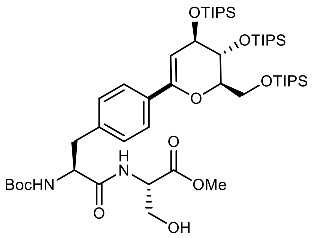

17h

Methyl ((*S*)-3-(4-((2*R*,3*R*,4*R*)-3,4-bis((triisopropylsilyl)oxy)-2-(((triisopropylsilyl)oxy)methyl)-3,4-dihydro-2*H*-pyran-6-yl)phenyl)-2-((*tert*-butoxycarbonyl)amino)propanoyl)-*L*-serinate (17h). According to the general procedure A, glycal boronates **12b** (95.0 mg, 0.12 mmol, 1.20 equiv), methyl ((*S*)-3-(4-bromophenyl)-2-((*tert*-butoxycarbonyl)amino)propanoyl)-*L*-serinate **16h** (44.4 mg, 0.10 mmol, 1.00 equiv), $Pd(PPh_3)_2Cl_2$ (35.0 mg, 0.050 mmol, 50.0 mol%), K_3PO_4 (63.6 mg, 0.30 mmol, 3.00 equiv) were added to a one-dram vial with a screw-top septum, and the vial was then evacuated and refilled with N_2 (3×). Anhydrous DMF (2.00 mL) were added, and the reaction mixture was stirred at 33 °C for 48 h, cooled to rt, and concentrated. The crude material was purified by column chromatography on SiO_2 (Petroleum ether:EtOAc, 1:0-1.5:1) to afford **17h** (52.4 mg, 53%) as a light-yellow foam: **^{1}H NMR** (500 MHz, Acetone-d_6) δ 7.61 (d, *J* = 8.0 Hz, 3H), 7.28 (d, *J* = 7.9 Hz, 2H), 6.13 (d, *J* = 8.5 Hz, 1H), 5.48 (dd, *J* = 5.3, 1.5 Hz, 1H), 4.59 – 4.56 (m, 1H), 4.54 – 4.46 (m, 2H), 4.28 – 4.24 (m, 2H), 4.22 – 4.19 (m, 1H), 4.00 (dd, *J* = 11.3, 4.0 Hz, 1H), 3.94 – 3.90 (m, 1H), 3.83 – 3.79 (m, 1H), 3.69 (s, 3H), 3.23 (dd, *J* = 14.0, 4.8 Hz, 1H), 2.98 – 2.92 (m, 1H), 1.33 (s, 9H), 1.30 – 1.27 (m, 1H), 1.18 – 1.06 (m, 63H); **^{13}C NMR** (126 MHz, Acetone-d_6) δ 172.2, 171.5, 156.3, 151.1, 139.3, 135.1, 130.1, 125.9, 96.9, 82.2, 79.4, 71.0, 67.7, 63.0, 62.9, 56.4, 55.6(2), 52.4, 38.4, 28.5, 18.6(2), 18.5(2), 18.4(2), 13.3, 13.2, 12.8; **HRMS** (ESI) *m/z* calcd for $C_{51}H_{95}N_2O_{10}Si_3$ $[M + H]^+$ 979.6289, found 979.6292.

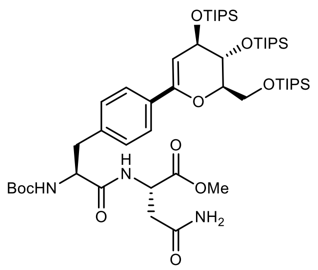

17i

Methyl ((*S*)-3-(4-((2*R*,3*R*,4*R*)-3,4-bis((triisopropylsilyl)oxy)-2-(((triisopropylsilyl)oxy)methyl)-3,4-dihydro-2*H*-pyran-6-yl)phenyl)-2-((*tert*-butoxycarbonyl)amino)propanoyl)-*L*-asparaginate (17i). According to the general procedure A, glycal boronates **12b** (95.0 mg, 0.12 mmol, 1.20 equiv), methyl ((*S*)-3-(4-bromophenyl)-2-((*tert*-butoxycarbonyl)amino)propanoyl)-*L*-asparaginate **16i** (47.1 mg, 0.10 mmol, 1.00 equiv), $Pd(PPh_3)_2Cl_2$ (35.0 mg, 0.050 mmol, 50.0 mol%), K_3PO_4 (63.6 mg, 0.30 mmol, 3.00 equiv) were added to a one-dram vial with a screw-top septum, and the vial was then evacuated and refilled with N_2 (3×). Anhydrous DMF (2.00 mL) were added, and the reaction mixture was stirred at 33 °C for 48 h, cooled to rt, and concentrated. The crude material was purified by column chromatography on SiO_2 (Petroleum ether:EtOAc, 1:0-1.5:1) to afford **17i** (46.5 mg, 46%) as a light-yellow oil: **^{1}H NMR** (500 MHz, Acetone-d_6) δ 7.66 – 7.60 (m, 3H), 7.23 (d, *J* = 8.0 Hz, 2H), 7.01 (s, 1H), 6.43 – 6.39 (m, 2H), 5.49 (dd, *J* = 5.3, 1.6 Hz, 1H), 4.69 (q, *J* = 6.6, 6.6, 6.5 Hz, 1H), 4.54 – 4.51 (m, 1H), 4.46 – 4.41 (m, 1H), 4.29 – 4.26 (m, 2H), 4.19 (dd, *J* = 11.2, 7.8 Hz, 1H), 4.02 (dd, *J* = 11.2, 4.2 Hz, 1H), 3.65 (s, 3H), 3.14 – 3.04 (m, 2H), 2.73 (dd, *J* = 15.8, 5.9 Hz, 1H), 2.63 (dd, *J* = 15.7, 6.0 Hz, 1H), 1.40 (s, 9H), 1.16 – 1.05 (m, 63H); **^{13}C NMR** (126 MHz, Acetone-d_6) δ 173.5, 172.2, 172.0, 156.3, 150.9, 138.1, 135.4, 130.0, 126.1, 97.0, 82.2, 79.7, 70.9, 67.7, 62.8, 54.5, 52.3, 52.1, 37.9, 37.6, 28.6, 18.7, 18.6, 18.5(2), 18.4(2), 13.2(2), 12.8; **HRMS** (ESI) m/z calcd for $C_{52}H_{95}N_3O_{10}Si_3Na$ $[M + Na]^+$ 1028.6217, found 1028.6198.

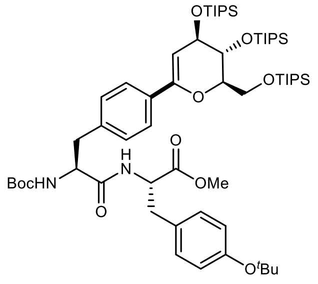

17j

Methyl (*S*)-2-((*S*)-3-(4-((2*R*,3*R*,4*R*)-3,4-bis((triisopropylsilyl)oxy)-2-(((triisopropylsilyl)oxy)methyl)-3,4-dihydro-2*H*-pyran-6-yl)phenyl)-2-((*tert*-butoxycarbonyl)amino)propanamido)-3-(4-(*tert*-butoxy)phenyl)propanoate (17j). According to the general procedure A, glycal boronates **12b** (95.0 mg, 0.12 mmol, 1.20 equiv), methyl (*S*)-2-((*S*)-3-(4-bromophenyl)-2-((*tert*-butoxycarbonyl)amino)propanamido)-3-(4-(*tert*-butoxy)phenyl)propanoate **16j** (57.6 mg, 0.10 mmol, 1.00 equiv), $Pd(PPh_3)_2Cl_2$ (3.50 mg, 0.005 mmol, 5.00 mol%), K_3PO_4

(63.6 mg, 0.30 mmol, 3.00 equiv) were added to a one-dram vial with a screw-top septum, and the vial was then evacuated and refilled with N_2 (3×). Anhydrous DMF (2.00 mL) were added, and the reaction mixture was stirred at 33 °C for 48 h, cooled to rt, and concentrated. The crude material was purified by column chromatography on SiO_2 (Petroleum ether:EtOAc, 1:0-5:1) to afford **17j** (48.2 mg, 43%) as a light-yellow oil: **^{1}H NMR** (400 MHz, Acetone-*d*$_6$) δ 7.60 (d, *J* = 7.9 Hz, 2H), 7.50 (d, *J* = 7.9 Hz, 1H), 7.23 (d, *J* = 8.0 Hz, 2H), 7.13 (d, *J* = 8.1 Hz, 2H), 6.90 (d, *J* = 8.1 Hz, 2H), 6.07 (d, *J* = 8.4 Hz, 1H), 5.48 (d, *J* = 5.2 Hz, 1H), 4.71 (q, *J* = 7.0, 7.0, 7.0 Hz, 1H), 4.53 – 4.50 (m, 1H), 4.41 – 4.36 (m, 1H), 4.28 – 4.18 (m, 3H), 4.00 (dd, *J* = 11.2, 3.9 Hz, 1H), 3.64 (s, 3H), 3.16 – 3.06 (m, 2H), 2.99 (dd, *J* = 13.8, 7.4 Hz, 1H), 1.33 (s, 9H), 1.29 (s, 9H), 1.15 – 1.05 (m, 63H); **^{13}C NMR** (126 MHz, Acetone-*d*$_6$) δ 172.5, 172.0, 156.1, 155.3, 151.0, 139.2, 135.1, 132.3, 130.6, 130.0, 125.9, 124.7, 96.9, 82.2, 79.3, 78.4, 71.0, 67.7, 62.9, 56.5, 54.5, 52.3, 38.4, 37.8, 29.1, 28.5, 18.6(2), 18.5(2), 18.4(2), 13.3, 13.2, 12.8; **HRMS** (ESI) m/z calcd for $C_{61}H_{107}N_2O_{10}Si_3$ $[M + H]^+$ 1111.7228, found 1111.7202.

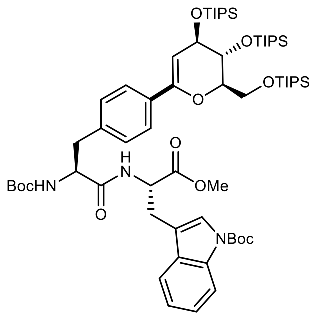

17k

***tert*-Butyl 3-((*S*)-2-((*S*)-3-(4-((2*R*,3*R*,4*R*)-3,4-bis((triisopropylsilyl)oxy)-2-(((triisopropylsilyl)oxy)methyl)-3,4-dihydro-2*H*-pyran-6-yl)phenyl)-2-((*tert*-butoxycarbonyl)amino)propanamido)-3-methoxy-3-oxopropyl)-1*H*-indole-1-carboxylate (17k).** According to the general procedure A, glycal boronates **12b** (95.0 mg, 0.12 mmol, 1.20 equiv), tert-butyl 3-((*S*)-2-((*S*)-3-(4-bromophenyl)-2-((*tert*-butoxycarbonyl)amino)propanamido)-3-methoxy-3-oxopropyl)-1*H*-indole-1-carboxylate **16k** (64.3 mg, 0.10 mmol, 1.00 equiv), $Pd(PPh_3)_2Cl_2$ (3.50 mg, 0.005 mmol, 5.00 mol%), K_3PO_4 (63.6 mg, 0.30 mmol, 3.00 equiv) were added to a one-dram vial with a screw-top septum, and the vial was then evacuated and refilled with N_2 (3×). Anhydrous DMF (2.00 mL) were added, and the reaction mixture was stirred at 33 °C for 48 h, cooled to rt, and concentrated. The crude material was purified by column chromatography on SiO_2 (Petroleum ether:EtOAc, 1:0-5:1) to afford **17k** (77.4 mg, 66%) as a colorless oil: **^{1}H NMR** (400 MHz, Acetone-*d*$_6$) δ 8.13 (d, *J* = 8.2 Hz, 1H), 7.64 (d, *J* = 7.7 Hz, 1H), 7.60 – 7.56 (m, 4H), 7.33 – 7.29 (m, 1H), 7.25 – 7.21 (m, 1H), 7.17 (d, *J* = 8.0 Hz, 2H), 6.18 (d, *J* = 8.5 Hz, 1H), 5.48 (dd, *J* = 5.3, 1.6 Hz, 1H), 4.77 – 4.72 (m, 1H), 4.54 – 4.48 (m, 2H), 4.28 – 4.25 (m, 2H), 4.18 (dd, *J* = 11.1, 7.8 Hz, 1H), 4.01 (dd, *J* = 11.2, 4.1 Hz, 1H), 3.67 (s, 3H), 3.22 (dd, *J* = 14.7, 5.2 Hz, 1H), 3.13 (dd, *J* = 13.9, 5.6 Hz, 1H), 3.08 – 3.02 (m, 2H), 1.66 (s, 9H), 1.36 (s, 9H), 1.15 – 1.04

(m, 63H); **^{13}C NMR** (101 MHz, Acetone-d_6) δ 172.2, 172.0, 156.2, 150.9, 150.3, 138.2, 136.3, 135.3, 131.7, 130.0, 126.0, 125.1, 125.0, 123.2, 120.0, 117.4, 115.8, 97.0, 84.0, 82.2, 79.4, 70.9, 67.7, 62.8, 55.5, 55.3, 54.4, 52.4, 37.9, 32.0, 28.6, 28.3, 18.7, 18.6, 18.5(2), 18.4, 13.2(2), 12.7; **HRMS** (ESI) m/z calcd for $C_{64}H_{107}N_3O_{11}Si_3Na$ [M + Na]$^+$ 1200.7106, found 1200.7101.

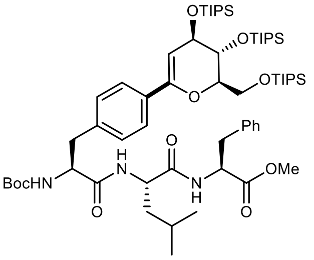

17l

Methyl ((*S*)-3-(4-((2*R*,3*R*,4*R*)-3,4-bis((triisopropylsilyl)oxy)-2-(((triisopropylsilyl)oxy)methyl)-3,4-dihydro-2*H*-pyran-6-yl)phenyl)-2-((*tert*-butoxycarbonyl)amino)propanoyl)-*L*-leucyl-*L*-phenylalaninate (17l). According to the general procedure A, glycal boronates **12b** (95.0 mg, 0.12 mmol, 1.20 equiv), methyl ((*S*)-3-(4-bromophenyl)-2-((*tert*-butoxycarbonyl)amino)propanoyl)-*L*-leucyl-*L*-phenylalaninate **16l** (61.7 mg, 0.10 mmol, 1.00 equiv), $Pd(PPh_3)_2Cl_2$ (3.50 mg, 0.005 mmol, 5.00 mol%), K_3PO_4 (63.6 mg, 0.30 mmol, 3.00 equiv) were added to a one-dram vial with a screw-top septum, and the vial was then evacuated and refilled with N_2 (3×). Anhydrous DMF (2.00 mL) were added, and the reaction mixture was stirred at 33 °C for 48 h, cooled to rt, and concentrated. The crude material was purified by column chromatography on SiO_2 (Petroleum ether:EtOAc, 1:0-3:1) to afford **17l** (98.2 mg, 85%) as a colorless oil: **^{1}H NMR** (400 MHz, Acetone-d_6) δ 7.63 – 7.58 (m, 3H), 7.32 (d, *J* = 9.0 Hz, 1H), 7.29 – 7.17 (m, 7H), 6.23 (d, *J* = 8.4 Hz, 1H), 5.46 (dd, *J* = 5.3, 1.6 Hz, 1H), 4.78 – 4.72 (m, 1H), 4.54 – 4.50 (m, 1H), 4.45 – 4.39 (m, 2H), 4.28 – 4.25 (m, 2H), 4.20 (dd, *J* = 11.2, 7.8 Hz, 1H), 4.01 (dd, *J* = 11.2, 4.1 Hz, 1H), 3.65 (s, 3H), 3.19 – 3.12 (m, 2H), 3.04 (dd, *J* = 13.9, 7.9 Hz, 1H), 2.94 (dd, *J* = 14.2, 9.5 Hz, 1H), 1.86 – 1.79 (m, 1H), 1.50 – 1.44 (m, 1H), 1.34 (s, 9H), 1.17 – 1.06 (m, 64H), 0.89 (d, *J* = 6.8 Hz, 3H), 0.83 (t, *J* = 7.3, 7.3 Hz, 3H); **^{13}C NMR** (101 MHz, Acetone-d_6) δ 172.4, 172.1, 171.7, 156.3, 151.1, 139.4, 137.8, 135.1, 130.1, 130.0, 129.2, 127.5, 125.9, 96.9, 82.2, 79.4, 71.0, 67.7, 62.9, 57.9, 56.5, 54.4, 52.2, 38.4, 38.2, 28.5, 25.1, 18.7, 18.6, 18.5(2), 18.4(2), 15.7, 13.3, 13.2, 12.8, 11.6; **HRMS** (ESI) *m/z* calcd for $C_{43}H_{82}NO_6Si_3$ [M + H$^+$ 1174.7313, found 1174.7313.

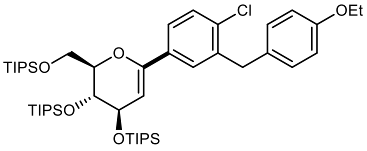

17m

(((2*R*,3*R*,4*R*)-6-(4-Chloro-3-(4-ethoxybenzyl)phenyl)-2-(((triisopropylsilyl)oxy)methyl)-3,4-dihydro-2*H*-pyran-3,4-

diyl)bis(oxy))bis(triisopropylsilane)(17m). According to the general procedure A, glycal boronates **12b** (95.0 mg, 0.12 mmol, 1.20 equiv), 4-bromo-1-chloro-2-[(4-ethoxyphenyl)methyl]benzene (32.5 mg, 0.10 mmol, 1.00 equiv), $Pd(PPh_3)_2Cl_2$ (3.50 mg, 0.005 mmol, 5.00 mol%), K_3PO_4 (63.6 mg, 0.30 mmol, 3.00 equiv) were added to a one-dram vial with a screw-top septum, and the vial was then evacuated and refilled with N_2 (3×). Anhydrous DMF (2.00 mL) were added, and the reaction mixture was stirred at 33 °C for 48 h, cooled to rt, and concentrated. The crude material was purified by column chromatography on SiO_2 (Petroleum ether:ether, 1:0-50:1)to afford **17m** (75.0 mg, 87%) as a colorless oil: **^{1}H NMR** (400 MHz, Acetone-*d*$_6$) δ 7.58 – 7.54 (m, 2H), 7.39 (d, *J* = 8.3 Hz, 1H), 7.12 (d, *J* = 8.3 Hz, 2H), 6.84 (d, *J* = 8.5 Hz, 2H), 5.48 (dd, *J* = 5.1, 1.8 Hz, 1H), 4.53 – 4.50 (m, 1H), 4.25 – 4.17 (m, 3H), 4.04 – 3.93 (m, 5H), 1.35 (t, *J* = 7.0, 7.0 Hz, 3H), 1.13 – 1.02 (m, 63H); **^{13}C NMR** (101 MHz, Acetone) δ 158.5, 150.0, 139.8, 135.8, 134.6, 131.9, 130.6, 129.9, 128.6, 125.3, 115.2, 97.9, 82.4, 70.8, 67.5, 63.8, 62.8, 38.8, 18.6(2), 18.5(3), 18.4(2), 18.3, 15.2, 13.3, 13.2, 12.7; **HRMS** (ESI) *m/z* calcd for $C_{48}H_{84}O_5Si_3Cl$ $[M + H]^+$ 859.5310, found 859.5326.

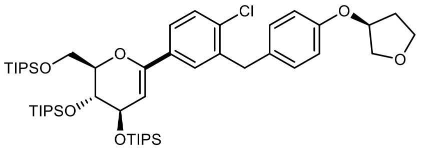

17n

(((2*R*,3*R*,4*R*)-6-(4-Chloro-3-(4-(((*S*)-tetrahydrofuran-3-yl)oxy)benzyl)phenyl)-2-(((triisopropylsilyl)oxy)methyl)-3,4-dihydro-2*H*-pyran-3,4-diyl)bis(oxy))bis(triisopropylsilane)(17n). According to the general procedure A, glycal boronates **12b** (95.0 mg, 0.12 mmol, 1.20 equiv), (3*S*)-3-[4-[(5-bromo-2-chlorophenyl)methyl]phenoxy]tetrahydro-furan (36.7 mg, 0.10 mmol, 1.00 equiv), $Pd(PPh_3)_2Cl_2$ (3.50 mg, 0.005 mmol, 5.00 mol%), K_3PO_4 (63.6 mg, 0.30 mmol, 3.00 equiv) were added to a one-dram vial with a screw-top septum, and the vial was then evacuated and refilled with N_2 (3×). Anhydrous DMF (2.00 mL) were added, and the reaction mixture was stirred at 33 °C for 48 h, cooled to rt, and concentrated. The crude material was purified by column chromatography on SiO_2 (Petroleum ether:ether, 1:0-50:1)to afford **17n** (73.0 mg, 81%) as a colorless oil: **^{1}H NMR** (400 MHz, $CDCl_3$) δ 7.47 (d, *J* = 2.1 Hz, 1H), 7.43 (dd, *J* = 8.3, 2.2 Hz, 1H), 7.30 (d, *J* = 8.3 Hz, 1H), 7.10 (d, *J* = 8.6 Hz, 2H), 6.77 (d, *J* = 8.6 Hz, 2H), 5.27 (dd, *J* = 5.4, 1.4 Hz, 1H), 4.90 – 4.87 (m, 1H), 4.46 – 4.42 (m, 1H), 4.15 – 4.08 (m, 3H), 4.03 – 3.81 (m, 7H), 2.20 – 2.12 (m, 2H), 1.06 – 0.99 (m, 63H); **^{13}C NMR** (101 MHz, $CDCl_3$) δ 155.9, 149.4, 138.4, 135.1, 134.1, 132.0, 130.0, 129.3, 128.0, 124.7, 115.4, 97.2, 81.5, 73.3, 70.1, 67.3, 66.7, 62.0, 38.6, 33.2, 18.3(2), 18.2(2), 18.1(2), 17.8, 12.7, 12.5, 12.1; **HRMS** (ESI) *m/z* calcd for $C_{50}H_{86}O_6Si_3Cl$ $[M + H]^+$ 901.5415, found 901.5421.

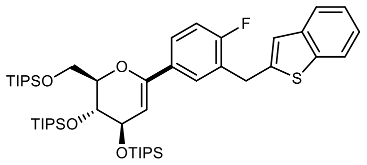

17o

(((2*R*,3*R*,4*R*)-6-(3-(Benzo[*b*]thiophen-2-ylmethyl)-4-fluorophenyl)-2-(((triisopropylsilyl)oxy)methyl)-3,4-dihydro-2*H*-pyran-3,4-diyl)bis(oxy))bis(triisopropylsilane)(17o). According to the general procedure A, glycal boronates **12b** (95.0 mg, 0.12 mmol, 1.20 equiv), 2-[(5-bromo-2-fluorophenyl)methyl]-benzo[*b*]thiophene (32.1 mg, 0.10 mmol, 1.00 equiv), $Pd(PPh_3)_2Cl_2$ (3.50 mg, 0.005 mmol, 5.00 mol%), K_3PO_4 (63.6 mg, 0.30 mmol, 3.00 equiv) were added to a one-dram vial with a screw-top septum, and the vial was then evacuated and refilled with N_2 (3×). Anhydrous DMF (2.00 mL) were added, and the reaction mixture was stirred at 33 °C for 48 h, cooled to rt, and concentrated. The crude material was purified by column chromatography on SiO_2 (Petroleum ether:Ether, 1:0-50:1)to afford **17o** (80.3 mg, 94%) as a light-yellow oil: **^{1}H NMR** (400 MHz, $CDCl_3$) δ 7.75 (d, *J* = 7.7 Hz, 1H), 7.66 (dd, *J* = 7.7, 1.3 Hz, 1H), 7.37 (d, *J* = 9.6 Hz, 2H), 7.32 – 7.20 (m, 3H), 7.03 (s, 1H), 5.36 (dd, *J* = 5.4, 1.5 Hz, 1H), 4.49 – 4.45 (m, 1H), 4.25 (s, 2H), 4.19 – 4.16 (m, 1H), 4.14 – 4.09 (m, 2H), 3.89 (dd, *J* = 11.2, 4.1 Hz, 1H), 1.10 – 1.02 (m, 63H); **^{13}C NMR** (101 MHz, $CDCl_3$) δ 160.7 (d, $J_{C\text{-}F}$ = 245.8 Hz), 149.1 (d, $J_{C\text{-}F}$ = 2.4 Hz), 143.6, 140.2, 139.9, 137.4 (d, $J_{C\text{-}F}$ = 8.1 Hz), 130.4(d, $J_{C\text{-}F}$ = 4.4 Hz), 126.5, 126.3, 124.3, 123.8, 123.1, 122.3, 122.0, 121.2 (d, $J_{C\text{-}F}$ = 3.2 Hz), 112.7(d, $J_{C\text{-}F}$ = 24.0 Hz), 97.5, 81.5, 70.1, 66.7, 62.0, 29.9(2), 18.3(3), 18.2, 18.1(2), 17.8, 12.6, 12.5, 12.1; **HRMS** (ESI) *m/z* calcd for $C_{48}H_{80}FO_4SSi_3$ $[M + H]^+$ 855.5064, found 855.5070.

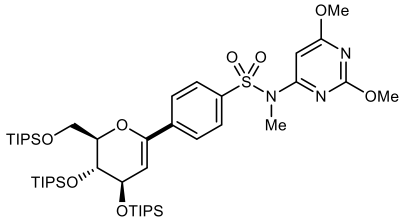

17p

4-((2*R*,3*R*,4*R*)-3,4-Bis((triisopropylsilyl)oxy)-2-(((triisopropylsilyl)oxy)methyl)-3,4-dihydro-2*H*-pyran-6-yl)-*N*-(2,6-dimethoxypyrimidin-4-yl)-*N*-methylbenzenesulfonamide (17p). According to the general procedure A, glycal boronates **12b** (95.0 mg, 0.12 mmol, 1.20 equiv), 4-bromo-*N*-(2,6-dimethoxypyrimidin-4-yl)-*N*-methylbenzenesulfonamide[16] (38.7 mg, 0.10 mmol, 1.00 equiv), $Pd(PPh_3)_2Cl_2$ (3.50 mg, 0.005 mmol, 5.00 mol%), K_3PO_4 (63.6 mg, 0.30 mmol, 3.00 equiv) were added to a one-dram vial with a screw-top septum, and the vial was then evacuated and refilled with N_2 (3×). Anhydrous DMF (2.00 mL) were added, and the reaction mixture was stirred at 33 °C for 48 h, cooled to rt, and concentrated. The crude material was purified by column chromatography on SiO_2 (Petroleum ether: EtOAc, 1:0-10:1) to afford **17p** (89.1 mg, 97%) as a colorless oil: **^{1}H NMR** (400 MHz, Acetone-d_6) δ 7.93 – 7.87 (m, 4H), 6.56 (s, 1H), 5.76 (dd, *J* = 5.3, 1.6 Hz, 1H), 4.59 –

4.55 (m, 1H), 4.29 – 4.19 (m, 3H), 3.96 (dd, *J* = 11.4, 3.6 Hz, 1H), 3.89 (s, 3H), 3.79 (s, 3H), 3.48 (s, 3H), 1.12 – 1.00 (m, 63H); **^{13}C NMR** (101 MHz, Acetone-d_6) δ 173.4, 165.3, 162.4, 149.2, 141.5, 139.3, 128.0, 126.6, 100.4, 89.9, 82.5, 70.7, 67.3, 62.6, 54.9, 54.3, 34.9, 18.6(2), 18.5, 18.4(2), 18.3, 13.2, 13.1, 12.7; **HRMS** (ESI) m/z calcd for $C_{46}H_{84}N_3O_8Si_3S$ $[M + H]^+$ 922.5281, found 922.5308.

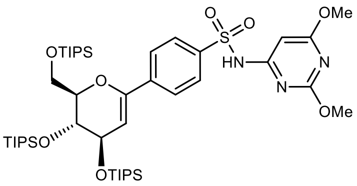

17q

4-((2*R*,3*R*,4*R*)-3,4-Bis((triisopropylsilyl)oxy)-2-(((triisopropylsilyl)oxy)methyl)-3,4-dihydro-2*H*-pyran-6-yl)-*N*-(2,6-dimethoxypyrimidin-4-yl)benzenesulfonamide (17q). According to the general procedure A, glycal boronates **12b** (95.0 mg, 0.12 mmol, 1.20 equiv), 4-bromo-*N*-(2,6-dimethoxypyrimidin-4-yl)benzenesulfonamide[17] (37.3 mg, 0.10 mmol, 1.00 equiv), $Pd(PPh_3)_2Cl_2$ (7.00 mg, 0.010 mmol, 10.0 mol%), K_3PO_4 (63.6 mg, 0.30 mmol, 3.00 equiv) were added to a one-dram vial with a screw-top septum, and the vial was then evacuated and refilled with N_2 (3×). Anhydrous DMF (2.00 mL) were added, and the reaction mixture was stirred at 33 °C for 48 h, cooled to rt, and concentrated. The crude material was purified by column chromatography on SiO_2 (Petroleum ether:EtOAc, 1:0-6:1) to afford **17q** (68.2 mg, 75%) as a light-yellow oil: **^{1}H NMR** (500 MHz, Acetone-d_6) δ 10.07 (s, 1H), 8.04 – 8.02 (m, 2H), 7.94 – 7.91 (m, 2H), 6.15 (s, 1H), 5.75 (dd, *J* = 5.3, 1.6 Hz, 1H), 4.58 – 4.55 (m, 1H), 4.29 – 4.25 (m, 2H), 4.22 (dd, *J* = 11.4, 8.3 Hz, 1H), 3.96 (dd, *J* = 11.4, 3.6 Hz, 1H), 3.85 (s, 3H), 3.79 (s, 3H), 1.16 – 0.99 (m, 63H); **^{13}C NMR** (126 MHz, Acetone-d_6) δ 173.4, 165.9, 160.7, 149.3, 141.4, 140.7, 128.4, 126.5, 100.3, 85.9, 82.5, 70.8, 67.3, 62.6, 55.0, 54.2, 18.6(2), 18.5, 18.4(2), 18.3, 13.2, 13.1, 12.7; **HRMS** (ESI) m/z calcd for $C_{45}H_{82}N_3O_8SSi_3$ $[M + H]^+$ 908.5125, found 908.5138.

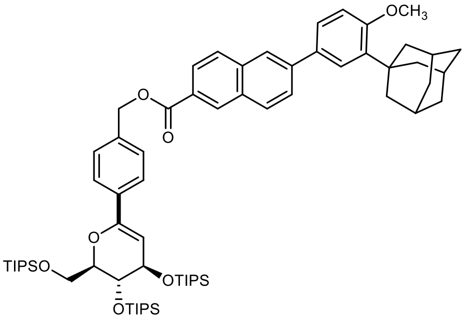

17r

4-((2*R*,3*R*,4*R*)-3,4-Bis((triisopropylsilyl)oxy)-2-(((triisopropylsilyl)oxy)methyl)-3,4-dihydro-2*H*-pyran-6-yl)benzyl 6-(3-((3*r*,5*r*,7*r*)-adamantan-1-yl)-4-methoxyphenyl)-2-naphthoate (17r). According to the general procedure A, glycal boronates **12b** (95.0 mg, 0.12 mmol, 1.20 equiv), 4-bromobenzyl 6-(3-((3*s*)-adamantan-1-yl)-4-methoxyphenyl)-2-naphthoate[18] (58.0 mg, 0.10 mmol, 1.00 equiv),

$Pd(PPh_3)_2Cl_2$ (3.50 mg, 0.005 mmol, 5.00 mol%), K_3PO_4 (63.6 mg, 0.30 mmol, 3.00 equiv) were added to a one-dram vial with a screw-top septum, and the vial was then evacuated and refilled with N_2 (3×). Anhydrous DMF (2.00 mL) were added, and the reaction mixture was stirred at 33 °C for 48 h, cooled to rt, and concentrated. The crude material was purified by column chromatography on SiO_2 (Petroleum ether:EtOAc, 1:0-20:1) to afford **17r** (89.5 mg, 81%) as a colorless oil: **^{1}H NMR** (400 MHz, $CDCl_3$) δ 8.65 (d, *J* = 1.6 Hz, 1H), 8.11 (dd, *J* = 8.6, 1.7 Hz, 1H), 8.02 – 7.98 (m, 2H), 7.92 (d, *J* = 8.6 Hz, 1H), 7.80 (dd, *J* = 8.6, 1.8 Hz, 1H), 7.71 (d, *J* = 8.2 Hz, 2H), 7.62 (d, *J* = 2.3 Hz, 1H), 7.55 (dd, *J* = 8.4, 2.3 Hz, 1H), 7.48 (d, *J* = 8.1 Hz, 2H), 7.00 (d, *J* = 8.4 Hz, 1H), 5.44 (s, 2H), 5.41 (dd, *J* = 5.4, 1.5 Hz, 1H), 4.51 – 4.47 (m, 1H), 4.22 – 4.11 (m, 3H), 3.95 – 3.91 (m, 4H), 2.20 – 2.19 (m, 6H), 2.13 – 2.11 (m, 3H), 1.85 – 1.78 (m, 6H), 1.12 – 1.03 (m, 63H); **^{13}C NMR** (101 MHz, $CDCl_3$) δ 166.8, 159.0, 150.0, 141.5, 139.1, 136.5, 136.1(2), 132.7, 131.4, 131.1, 129.9, 128.4, 128.1, 127.0, 126.6, 126.1, 125.9, 125.8(2), 124.9, 112.2, 97.2, 81.5, 70.1, 66.8, 62.1, 55.3, 40.7, 37.3(2), 29.2, 18.4, 18.3(2), 18.2(2), 18.1, 12.7, 12.6, 12.1; **HRMS** (ESI) *m/z* calcd for $C_{68}H_{103}O_7Si_3$ $[M + H]^+$ 1115.7006, found 1115.7000.

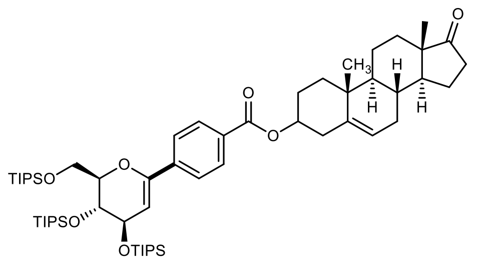

17s

(8*R*,9*S*,10*R*,13*S*,14*S*)-10,13-Dimethyl-17-oxo-2,3,4,7,8,9,10,11,12,13,14,15,16,17-tetradecahydro-1*H*-cyclopenta[a]phenanthren-3-yl 4-((2*R*,3*R*,4*R*)-3,4-bis((triisopropylsilyl)oxy)-2-(((triisopropylsilyl)oxy)methyl)-3,4-dihydro-2*H*-pyran-6-yl)benzoate (17s). According to the general procedure A, glycal boronates **12b** (95.0 mg, 0.12 mmol, 1.20 equiv), (8*R*,9*S*,10*R*,13*S*,14*S*)-10,13-dimethyl-17-oxo-2,3,4,7,8,9,10,11,12,13,14,15,16,17-tetradecahydro-1*H*-cyclopenta[*a*]phenanthren-3-yl 4-bromobenzoate[16] (47.0 mg, 0.10 mmol, 1.00 equiv), $Pd(PPh_3)_2Cl_2$ (3.50 mg, 0.005 mmol, 5.00 mol%), K_3PO_4 (63.6 mg, 0.30 mmol, 3.00 equiv) were added to a one-dram vial with a screw-top septum, and the vial was then evacuated and refilled with N_2 (3×). Anhydrous DMF (2.00 mL) were added, and the reaction mixture was stirred at 33 °C for 48 h, cooled to rt, and concentrated. The crude material was purified by column chromatography on SiO_2 (Petroleum ether:ether, 1:0-50:1) to afford **17s** (92.5 mg, 92%) as a colorless oil: **^{1}H NMR** (400 MHz, $CDCl_3$) δ 7.99 (d, *J* = 8.3 Hz, 2H), 7.69 (d, *J* = 8.2 Hz, 2H), 5.47 – 5.45 (m, 2H), 4.90 – 4.82 (m, 1H), 4.50 – 4.46 (m, 1H), 4.19 – 4.11 (m, 3H), 3.85 (dd, *J* = 11.3, 3.7 Hz, 1H), 2.52 – 2.43 (m, 3H), 2.16 – 1.84 (m, 6H), 1.80 – 1.67 (m, 4H), 1.58 – 1.46 (m, 2H), 1.35 – 1.18 (m, 4H), 1.11 – 1.00 (m, 66H), 0.90 (s, 3H); **^{13}C NMR** (101 MHz, $CDCl_3$) δ 166.0, 149.4, 140.5, 140.1, 130.3, 129.4, 125.2, 122.1, 98.7, 81.6, 74.4, 70.1, 66.6, 62.0, 51.8, 50.3, 47.7, 38.3, 37.1, 36.9, 36.0, 31.6, 31.5, 30.9, 27.9, 22.0, 20.5, 19.6, 18.3(2), 18.2(2), 18.1(2), 13.7, 12.6, 12.5, 12.1;

HRMS (ESI) *m/z* calcd for $C_{59}H_{101}O_7Si_3$ $[M + H]^+$ 1005.6850, found 1005.6851.

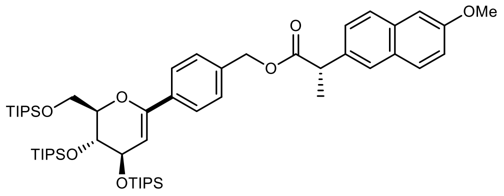

17t

4-((2*R*,3*R*,4*R*)-3,4-Bis((triisopropylsilyl)oxy)-2-(((triisopropylsilyl)oxy)methyl)-3,4-dihydro-2*H*-pyran-6-yl)benzyl (*S*)-2-(6-methoxynaphthalen-2-yl)propanoate (17t). According to the general procedure A, glycal boronates **12b** (95.0 mg, 0.12 mmol, 1.20 equiv), 4-bromobenzyl (*S*)-2-(6-methoxynaphthalen-2-yl)propanoate[16] (39.8 mg, 0.10 mmol, 1.00 equiv), $Pd(PPh_3)_2Cl_2$ (3.50 mg, 0.005 mmol, 5.00 mol%), K_3PO_4 (63.6 mg, 0.30 mmol, 3.00 equiv) were added to a one-dram vial with a screw-top septum, and the vial was then evacuated and refilled with N_2 (3×). Anhydrous DMF (2.00 mL) were added, and the reaction mixture was stirred at 33 °C for 48 h, cooled to rt, and concentrated. The crude material was purified by column chromatography on SiO_2 (Petroleum ether:EtOAc, 1:0-20:1) to afford **17t** (84.5 mg, 91%) as a colorless oil: **^{1}H NMR** (400 MHz, Acetone-*d*$_6$) δ 7.77 – 7.70 (m, 3H), 7.64 – 7.62 (m, 2H), 7.45 – 7.42 (m, 1H), 7.26 (dd, *J* = 5.5, 2.7 Hz, 3H), 7.14 (dd, *J* = 9.0, 2.6 Hz, 1H), 5.53 – 5.50 (m, 1H), 5.13 (d, *J* = 1.7 Hz, 2H), 4.55 – 4.51 (m, 1H), 4.28 – 4.19 (m, 3H), 4.01 – 3.95 (m, 2H), 3.90 (s, 3H), 1.54 (d, *J* = 7.1 Hz, 3H), 1.16 – 1.03 (m, 63H); **^{13}C NMR** (126 MHz, Acetone-*d*$_6$) δ 174.6, 158.7, 150.6, 137.8, 136.9, 136.5, 134.8, 130.0, 129.9, 128.4(2), 127.9, 127.1, 126.7(2), 126.0, 119.8, 106.4, 97.5, 82.3, 70.9, 67.6, 66.4, 62.8, 55.6, 45.9, 19.0, 18.7, 18.6, 18.5(2), 18.4(2), 13.3, 13.2, 12.7; **HRMS** (ESI) m/z calcd for $C_{54}H_{89}O_7Si_3$ $[M + H]^+$ 933.5911, found 933.5908.

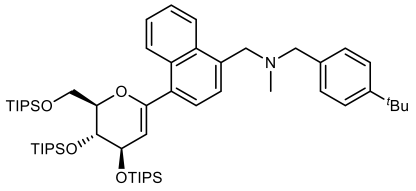

17u

1-(4-((2*R*,3*R*,4*R*)-3,4-Bis((triisopropylsilyl)oxy)-2-(((triisopropylsilyl)oxy)methyl)-3,4-dihydro-2*H*-pyran-6-yl)naphthalen-1-yl)-*N*-(4-(*tert*-butyl)benzyl)-*N*-methylmethanamine (17u). According to the general procedure A, glycal boronates **12b** (95.0 mg, 0.12 mmol, 1.20 equiv), 1-(4-bromonaphthalen-1-yl)-*N*-(4-(*tert*-butyl)benzyl)-*N*-methylmethanamine (39.5 mg, 0.10 mmol, 1.00 equiv), $Pd(PPh_3)_2Cl_2$ (3.50 mg, 0.005 mmol, 5.00 mol%), K_3PO_4 (63.6 mg, 0.30 mmol, 3.00 equiv) were added to a one-dram vial with a screw-top septum, and the vial was then evacuated and refilled with N_2 (3×). Anhydrous DMF (2.00 mL) were added, and the reaction mixture was stirred at 33 °C for 48 h, cooled to rt, and concentrated. The crude material was purified by column chromatography on SiO_2 (Petroleum ether:EtOAc, 1:0-10:1) to

afford **17u** (85.4 mg, 92%) as a light-yellow oil: **^{1}H NMR** (500 MHz, Acetone-d_6) δ 8.46 (dd, *J* = 8.4, 1.4 Hz, 1H), 8.34 (dd, *J* = 8.5, 1.4 Hz, 1H), 7.52 – 7.44 (m, 4H), 7.35 – 7.33 (m, 2H), 7.28 – 7.26 (m, 2H), 5.19 (dd, *J* = 5.1, 2.0 Hz, 1H), 4.58 – 4.55 (m, 1H), 4.40 – 4.32 (m, 3H), 4.15 (dd, *J* = 11.3, 4.0 Hz, 1H), 3.97 – 3.91 (m, 2H), 3.56 (s, 2H), 2.15 (s, 3H), 1.29 (s, 9H), 1.25 – 1.10 (m, 45H), 1.07 – 1.05 (m, 18H); **^{13}C NMR** (126 MHz, Acetone-d_6) δ 153.6, 150.4, 137.3, 137.0, 136.1, 133.4, 132.9, 129.5, 127.7, 127.4, 126.4, 126.3(2), 126.0, 125.8, 101.2, 82.9, 70.5, 67.3, 63.2, 62.4, 61.2, 42.4, 34.9, 31.7, 18.7(2), 18.6(2), 18.5, 13.2(2), 12.8; **HRMS** (ESI) m/z calcd for $C_{56}H_{96}NO_4Si_3$ [M + H]$^+$ 930.6642, found 930.6644.

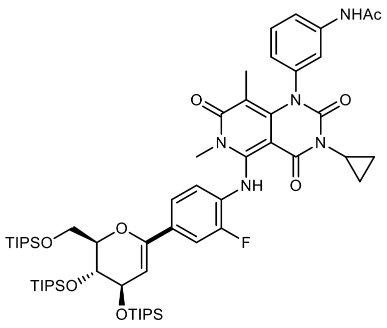

17v

***N*-(3-(5-((4-((2*R*,3*R*,4*R*)-3,4-Bis((triisopropylsilyl)oxy)-2-(((triisopropylsilyl)oxy)methyl)-3,4-dihydro-2*H*-pyran-6-yl)-2-fluorophenyl)amino)-3-cyclopropyl-6,8-dimethyl-2,4,7-trioxo-3,4,6,7-tetrahydropyrido[4,3-*d*]pyrimidin-1(2*H*)-yl)phenyl)acetamide (17v).** According to the general procedure A, glycal boronates **12b** (95.0 mg, 0.12 mmol, 1.20 equiv), *N*-[3-[3-cyclopropyl-5-[(2-fluoro-4-iodophenyl)amino]-3,4,6,7-tetrahydro-6,8-dimethyl-2,4,7-trioxopyrido[4,3-*d*]pyrimidin-1(2*H*)-yl]phenyl]acetamide (61.5 mg, 0.10 mmol, 1.00 equiv), $Pd(PPh_3)_2Cl_2$ (3.50 mg, 0.005 mmol, 5.00 mol%), K_3PO_4 (63.6 mg, 0.30 mmol, 3.00 equiv) were added to a one-dram vial with a screw-top septum, and the vial was then evacuated and refilled with N_2 (3×). Anhydrous DMF (2.00 mL) were added, and the reaction mixture was stirred at 33 °C for 48 h, cooled to rt, and concentrated. The crude material was purified by column chromatography on SiO_2 (Petroleum ether:EtOAc, 1:0-1:1) to afford **17v** (87.6 mg, 80%) as a white solid: **^{1}H NMR** (400 MHz, $CDCl_3$) δ 11.38 (s, 1H), 7.89 (s, 1H), 7.74 (d, *J* = 2.4 Hz, 1H), 7.46 – 7.38 (m, 2H), 7.26 – 7.25 (m, 2H), 6.98 – 6.90 (m, 2H), 5.36 (dd, *J* = 5.4, 1.4 Hz, 1H), 4.49 – 4.45 (m, 1H), 4.18 – 4.10 (m, 3H), 3.84 (dd, *J* = 11.4, 3.7 Hz, 1H), 3.19 (s, 3H), 2.77 – 2.72 (m, 1H), 2.10 (s, 3H), 1.41 (s, 3H), 1.12 – 1.00 (m, 65H), 0.82 – 0.78 (m, 2H); **^{13}C NMR** (101 MHz, $CDCl_3$) δ 168.7, 164.9, 164.1, 154.2, 152.6, 152.1, 148.3, 145.1, 140.4, 139.1, 135.5, 129.2, 127.5, 124.1, 123.6, 121.6, 120.6, 119.1, 113.7, 113.4, 103.3, 97.9, 89.6, 81.7, 70.1, 66.6, 61.9, 34.8, 25.4, 24.6, 18.3, 18.2(3), 18.1(2), 17.8, 13.5, 12.6, 12.5, 12.1, 8.6; **HRMS** (ESI) *m/z* calcd for $C_{59}H_{93}N_5O_8FSi_3$ [M + H]$^+$ 1102.6310, found 1102.6293.

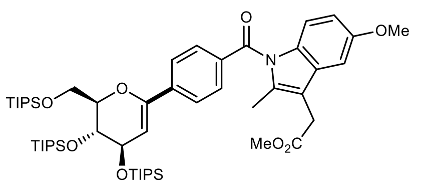

17w

Methyl 2-(1-(4-((2*R*,3*R*,4*R*)-3,4-bis((triisopropylsilyl)oxy)-2-(((triisopropylsilyl)oxy)methyl)-3,4-dihydro-2*H*-pyran-6-yl)benzoyl)-5-methoxy-2-methyl-1*H*-indol-3-yl)acetate (17w). According to the general procedure A, glycal boronates **12b** (95.0 mg, 0.12 mmol, 1.20 equiv), methyl 2-(1-(4-bromobenzoyl)-5-methoxy-2-methyl-1*H*-indol-3-yl)acetate[16] (41.5 mg, 0.10 mmol, 1.00 equiv), $Pd(PPh_3)_2Cl_2$ (3.50 mg, 0.005 mmol, 5.00 mol%), K_3PO_4 (63.6 mg, 0.30 mmol, 3.00 equiv) were added to a one-dram vial with a screw-top septum, and the vial was then evacuated and refilled with N_2 (3×). Anhydrous DMF (2.00 mL) were added, and the reaction mixture was stirred at 33 °C for 48 h, cooled to rt, and concentrated. The crude material was purified by column chromatography on SiO_2 (Petroleum ether:EtOAc, 1:0-10:1) to afford **17w** (69.8 mg, 73%) as a light-yellow oil: **^{1}H NMR** (500 MHz, Acetone-d_6) δ 7.92 – 7.90 (m, 2H), 7.72 – 7.70 (m, 2H), 7.07 (d, *J* = 2.5 Hz, 1H), 6.96 (d, *J* = 9.0 Hz, 1H), 6.66 (dd, *J* = 9.0, 2.6 Hz, 1H), 5.78 (dd, *J* = 5.4, 1.5 Hz, 1H), 4.63 – 4.60 (m, 1H), 4.33 – 4.27 (m, 3H), 4.00 (dd, *J* = 11.4, 3.5 Hz, 1H), 3.81 (s, 3H), 3.75 (s, 2H), 3.66 (s, 3H), 2.33 (s, 3H), 1.22 – 1.04 (m, 63H); **^{13}C NMR** (126 MHz, Acetone-d_6) δ 171.7, 169.5, 156.9, 149.8, 140.9, 136.6, 136.5, 131.8, 131.6, 130.3, 126.2, 115.5, 113.4, 112.2, 102.1, 99.7, 82.5, 70.9, 67.4, 62.7, 55.8, 52.1, 18.7, 18.6, 18.5(2), 18.4(2), 13.5, 13.3, 13.2, 12.8; **HRMS** (ESI) *m/z* calcd for $C_{53}H_{88}NO_8Si_3$ $[M + H]^+$ 950.5812, found 950.5819.

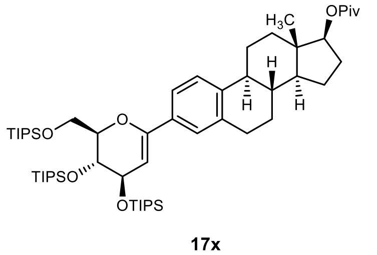

17x

(8*R*,9*S*,13*S*,14*S*,17*S*)-3-((2*R*,3*R*,4*R*)-3,4-Bis((triisopropylsilyl)oxy)-2-(((triisopropylsilyl)oxy)methyl)-3,4-dihydro-2*H*-pyran-6-yl)-13-methyl-7,8,9,11,12,13,14,15,16,17-decahydro-6*H*-cyclopenta[*a*]phenanthren-17-yl pivalate (17x). According to the general procedure A, glycal boronates **12b** (79.2 mg, 0.10 mmol, 1.00 equiv), (8*R*,9*S*,13*S*,14*S*,17*S*)-3-bromo-13-methyl-7,8,9,11,12,13,14,15,16,17-decahydro-6*H*-cyclopenta[*a*]phenanthren-17-yl pivalate[19] (73.2 mg, 0.15 mmol, 1.50 equiv), $Pd(PPh_3)_2Cl_2$ (3.50 mg, 0.005 mmol, 5.00 mol%), K_3PO_4 (63.6 mg, 0.30 mmol, 3.00 equiv) were added to a one-dram vial with a screw-top septum, and the vial was then evacuated and refilled with N_2 (3×). Anhydrous DMF (2.00 mL) were added, and the reaction mixture was stirred at 33 °C for 48 h, cooled to rt, and concentrated. The crude material was purified by column chromatography on

SiO_2 (Petroleum ether:ether, 1:0-50:1) to afford **17x** (85.8 mg, 90%) as a white foam: **^{1}H NMR** (400 MHz, Acetone-*d*$_6$) δ 7.45 – 7.42 (m, 2H), 7.26 (d, *J* = 8.1 Hz, 1H), 5.45 (dd, *J* = 5.3, 1.5 Hz, 1H), 4.65 (dd, *J* = 9.2, 7.3 Hz, 1H), 4.53 – 4.49 (m, 1H), 4.29 – 4.20 (m, 3H), 3.99 (dd, *J* = 11.2, 3.8 Hz, 1H), 2.93 – 2.84 (m, 2H), 2.38 – 2.14 (m, 3H), 1.96 – 1.85 (m, 2H), 1.81 – 1.74 (m, 1H), 1.54 – 1.29 (m, 7H), 1.20 (s, 9H), 1.16 – 1.07 (m, 63H), 0.90 (s, 3H); **^{13}C NMR** (101 MHz, Acetone-*d*$_6$) δ 178.1, 151.2, 141.5, 137.0, 134.2, 126.7, 125.9, 123.4, 96.5, 82.8, 82.2, 71.1, 67.8, 62.9, 50.7, 45.2, 43.8, 39.4, 39.2, 37.8, 28.3, 28.0, 27.5, 26.8, 23.9, 18.7, 18.6(2), 18.5(3), 13.3, 13.2, 12.8, 12.5; **HRMS** (ESI) m/z calcd for $C_{56}H_{101}O_6Si_3$ [M + H]$^+$ 953.6900, found 953.6894.

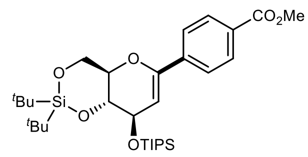

7a

Methyl 4-((4a*R*,8*R*,8a*R*)-2,2-di-*tert*-butyl-8-((triisopropylsilyl)oxy)-4,4a,8,8a-tetrahydropyrano[3,2-*d*][1,3,2]dioxasilin-6-yl)benzoate (7a). According to the general procedure A, glycal boronates **12m** (78mg, 0.12 mmol, 1.20 equiv), Methyl 4-bromobenzoate (21.5 mg, 0.10 mmol, 1.00 equiv), $Pd(PPh_3)_2Cl_2$ (3.50 mg, 0.005 mmol, 5.00 mol%), K_3PO_4 (63.6 mg, 0.30 mmol, 3.00 equiv) were added to a one-dram vial with a screw-top septum, and the vial was then evacuated and refilled with N_2 (3×). Anhydrous DMF (2.00 mL) were added, and the reaction mixture was stirred at 33 °C for 48 h, cooled to rt, and concentrated. The crude material was purified by column chromatography on SiO_2 (Petroleum ether:EtOAc, 1:0-50:1) to afford **7a** (53.6 mg, 93%) as a light-yellow oil: **^{1}H NMR** (400 MHz, Acetone-*d*$_6$) δ 8.01 – 7.98 (m, 2H), 7.72 – 7.68 (m, 2H), 5.50 (d, *J* = 2.5 Hz, 1H), 4.71 – 4.68 (m, 1H), 4.40 – 4.33 (m, 1H), 4.17 – 4.07 (m, 3H), 3.88 (s, 3H), 1.24 – 1.15 (m, 21H), 1.11 (s, 9H), 1.04 (s, 9H); **^{13}C NMR** (101 MHz, Acetone) δ 166.8, 150.7, 139.1, 131.1, 130.2, 125.7, 104.2, 78.5, 73.8, 72.6, 66.6, 52.4, 27.9, 27.4, 23.3, 20.4, 18.6(2), 13.2; **HRMS** (ESI) m/z calcd for $C_{31}H_{53}O_6Si_2$ [M + H]$^+$ 577.3375, found 577.3379.

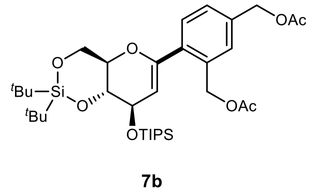

7b

(4-((4a*R*,8*R*,8a*R*)-2,2-Di-*tert*-butyl-8-((triisopropylsilyl)oxy)-4,4a,8,8a-tetrahydropyrano[3,2-*d*][1,3,2]dioxasilin-6-yl)-1,3-phenylene)bis(methylene) diacetate (7b). According to the general procedure A, glycal boronates **12m** (1.57 g, 2.40 mmol, 1.20 equiv), (2-bromo-1,4-phenylene)bis(methylene) diacetate (600 mg, 2.00 mmol, 1.00 equiv), $Pd(PPh_3)_2Cl_2$ (70.2 mg, 0.10 mmol, 5.00 mol%), K_3PO_4 (1.27 g, 6.00 mmol, 3.00 equiv) were added to a one-dram vial with a screw-top septum, and the vial was then evacuated and refilled with N_2 (3×). Anhydrous DMF (25.0 mL) were

added, and the reaction mixture was stirred at 33 °C for 48 h, cooled to rt, and concentrated. The crude material was purified by column chromatography on SiO_2 (Petroleum ether:EtOAc, 1:0-10:1) to afford **7b** (1.25 g, 91%) as a white foam: **^{1}H NMR** (400 MHz, Acetone-d_6) δ 7.44 – 7.38 (m, 3H), 5.19 (s, 2H), 5.10 (s, 2H), 4.97 (d, J = 2.2 Hz, 1H), 4.69 – 4.67 (m, 1H), 4.25 – 4.22 (m, 1H), 4.14 – 4.04 (m, 3H), 2.05 – 2.04 (m, 6H), 1.17 (q, J = 5.8, 5.3, 5.3 Hz, 21H), 1.11 (s, 9H), 1.05 (s, 9H); **^{13}C NMR** (101 MHz, Acetone-d_6) δ 170.7, 170.6, 152.7, 137.5, 135.6, 135.2, 130.3, 129.4, 129.3, 106.1, 78.5, 74.0, 72.6, 66.5, 65.7, 64.5, 27.9, 27.4, 23.3, 20.8(2), 20.4, 18.6(2), 13.2; **HRMS** (ESI) m/z calcd for $C_{35}H_{58}O_8Si_2Na$ $[M + Na]^+$ 685.3562, found 685.3565.

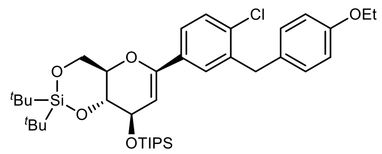

7c

(4a*R*,8*R*,8a*R*)-2,2-Di-*tert*-butyl-6-(4-chloro-3-(4-ethoxybenzyl)phenyl)-8-((triisopropylsilyl)oxy)-4,4a,8,8a-tetrahydropyrano[3,2-*d*][1,3,2]dioxasiline (17c). According to the general procedure A, glycal boronates **12m** (78.0 mg, 0.12 mmol, 1.20 equiv), 4-bromo-1-chloro-2-[(4-ethoxyphenyl)methyl]benzene (32.6 mg, 0.10 mmol, 1.00 equiv), $Pd(PPh_3)_2Cl_2$ (3.50 mg, 0.005 mmol, 5.0 mol%), K_3PO_4 (63.6 mg, 0.30 mmol, 3.00 equiv) were added to a one-dram vial with a screw-top septum, and the vial was then evacuated and refilled with N_2 (3×). Anhydrous DMF (2.00 mL) were added, and the reaction mixture was stirred at 33 °C for 48 h, cooled to rt, and concentrated. The crude material was purified by column chromatography on SiO_2 (Petroleum ether:Ether, 1:0-100:1) to afford **7c** (63.1 mg, 92%) as a colorless oil: **^{1}H NMR** (400 MHz, Acetone-d_6) δ 7.44 – 7.38 (m, 3H), 7.13 – 7.09 (m, 2H), 6.87 – 6.83 (m, 2H), 5.24 (d, J = 2.4 Hz, 1H), 4.65 – 4.59 (m, 1H), 4.31 – 4.28 (m, 1H), 4.11 – 3.98 (m, 7H), 1.35 (t, J = 7.0, 7.0 Hz, 3H), 1.19 – 1.12 (m, 21H), 1.09 (s, 9H), 1.02 (s, 9H); **^{13}C NMR** (101 MHz, Acetone-d_6) δ 158.6, 150.7, 140.1, 134.8, 133.9, 131.7, 130.7, 130.1, 128.1, 125.1, 115.2, 102.4, 78.5, 73.7, 72.5, 66.6, 63.8, 38.7, 27.8, 27.3, 23.3, 20.4, 18.6(2), 15.1, 13.2; **HRMS** (ESI) m/z calcd for $C_{38}H_{60}O_5Si_2Cl$ $[M + H]^+$ 687.3662, found 687.3668.

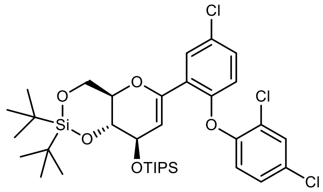

7d

(4a*R*,8*R*,8a*R*)-2,2-Di-*tert*-butyl-6-(5-chloro-2-(2,4-dichlorophenoxy)phenyl)-8-((triisopropylsilyl)oxy)-4,4a,8,8a-tetrahydropyrano[3,2-*d*][1,3,2]dioxasiline (7d). According to the general procedure A, glycal boronates **12m** (620 mg, 1.00 mmol, 1.00 equiv), 5-chloro-2-(2,4-dichlorophenoxy)phenyl trifluoromethanesulfonate[20] (630 mg, 1.50 mmol, 1.50 equiv), $Pd(PPh_3)_2Cl_2$ (70.0 mg, 0.10 mmol, 10.0 mol%), K_3PO_4 (636 mg, 3.00 mmol, 3.00 equiv) were added to a one-dram vial with a screw-top septum,

and the vial was then evacuated and refilled with N_2 (3×). Anhydrous DMF (20.0 mL) were added, and the reaction mixture was stirred at 33 °C for 48 h, cooled to rt, and concentrated. The crude material was purified by column chromatography on SiO_2 (Petroleum ether) to afford **7d** (610 mg, 86%) as a colorless oil: **^{1}H NMR** (500 MHz, Acetone-*d*$_6$) δ 7.62 – 7.61 (m, 2H), 7.40 – 7.35 (m, 2H), 6.96 (d, *J* = 8.8 Hz, 2H), 5.54 (d, *J* = 2.3 Hz, 1H), 4.57 (dd, *J* = 6.8, 2.3 Hz, 1H), 4.21 (dd, *J* = 10.1, 4.8 Hz, 1H), 4.06 – 3.93 (m, 3H), 1.14 – 1.06 (m, 30H), 1.02 (s, 9H); **^{13}C NMR** (126 MHz, Acetone-*d*$_6$) δ 152.6, 152.2, 146.9, 131.0, 130.7, 129.9, 129.6(2), 129.3, 128.4, 126.1, 121.9, 121.3, 108.4, 78.1, 73.9, 72.6, 66.5, 27.9, 27.4, 23.3, 20.4, 18.6, 18.5, 13.2; **HRMS** (ESI) m/z calcd for $C_{35}H_{52}Cl_3O_5Si_2$ [M + H]$^+$ 713.2413, found 713.2402.

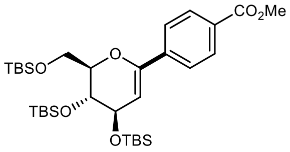

7e

Methyl 4-((2*R*,3*R*,4*R*)-3,4-bis((*tert*-butyldimethylsilyl)oxy)-2-(((*tert*-butyldimethylsilyl)oxy)methyl)-3,4-dihydro-2*H*-pyran-6-yl)benzoate (7e). According to the general procedure A, glycal boronates **12e** (78.0 mg, 0.12 mmol, 1.20 equiv), Methyl 4-bromobenzoate (21.5 mg, 0.10 mmol, 1.00 equiv), $Pd(PPh_3)_2Cl_2$ (3.50 mg, 0.005 mmol, 5.00 mol%), K_3PO_4 (63.6 mg, 0.30 mmol, 3.00 equiv) were added to a one-dram vial with a screw-top septum, and the vial was then evacuated and refilled with N_2 (3×). Anhydrous DMF (2.00 mL) were added, and the reaction mixture was stirred at 33 °C for 48 h, cooled to rt, and concentrated. The crude material was purified by column chromatography on SiO_2 (Petroleum ether:Ether, 1:0-50:1) to afford **7e** (55.7 mg, 90%) as a colorless oil: **^{1}H NMR** (400 MHz, $CDCl_3$) δ 8.00 (d, *J* = 8.5 Hz, 2H), 7.68 (d, *J* = 8.5 Hz, 2H), 5.38 (d, *J* = 4.3 Hz, 1H), 4.22 – 4.18 (m, 1H), 4.16 – 4.13 (m, 1H), 4.00 (dd, *J* = 11.3, 7.2 Hz, 1H), 3.91 (s, 3H), 3.88 (t, *J* = 4.1, 4.1 Hz, 1H), 3.83 (dd, *J* = 11.4, 3.6 Hz, 1H), 0.91 (s, 9H), 0.89 (s, 18H), 0.14 – 0.12 (m, 12H), 0.03 (s, 3H), 0.00 (s, 3H);**^{13}C NMR** (101 MHz, $CDCl_3$) δ 167.0, 149.7, 139.9, 129.9, 129.5, 125.2, 100.1, 80.8, 70.3, 68.7, 61.6, 52.2, 26.1, 26.0(2), 18.5, 18.2, -3.9, -4.0, -4.1, -4.5, -5.1, -5.2; **HRMS** (ESI) *m/z* calcd for $C_{32}H_{58}O_6Si_3Na$ [M + Na]$^+$ 645.3433, found 645.3434.

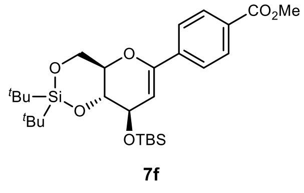

7f

Methyl 4-((4a*R*,8*R*,8a*R*)-2,2-di-*tert*-butyl-8-((*tert*-butyldimethylsilyl)oxy)-4,4a,8,8a-tetrahydropyrano[3,2-*d*][1,3,2]dioxasilin-6-yl)benzoate (7f). According to the general procedure A, glycal boronates **12f** (69.8 mg, 0.12 mmol, 1.20 equiv), Methyl 4-bromobenzoate (21.5 mg, 0.10 mmol, 1.00 equiv), $Pd(PPh_3)_2Cl_2$ (3.50 mg, 0.005 mmol, 5.00 mol%), K_3PO_4 (63.6 mg, 0.30 mmol, 3.00 equiv) were added to a one-dram vial with a screw-top septum, and the vial was then evacuated and refilled with N_2 (3×).

Anhydrous DMF (2.00 mL) were added, and the reaction mixture was stirred at 33 °C for 48 h, cooled to rt, and concentrated. The crude material was purified by column chromatography on SiO_2 (Petroleum ether:Ether, 1:0-50:1) to afford **7f** (47.4 mg, 89%) as a colorless oil: **^{1}H NMR** (400 MHz, Acetone-*d*$_6$) δ 8.00 – 7.96 (m, 2H), 7.73 – 7.70 (m, 2H), 5.46 (d, *J* = 2.5 Hz, 1H), 4.55 (dd, *J* = 6.9, 2.5 Hz, 1H), 4.40 – 4.33 (m, 1H), 4.16 – 4.02 (m, 3H), 3.88 (s, 3H), 1.11 (s, 9H), 1.05 (s, 8H), 0.97 (s, 9H), 0.22 (s, 3H), 0.21 (s, 3H); **^{13}C NMR** (101 MHz, Acetone-*d*$_6$) δ 166.8, 150.6, 139.2, 131.0, 130.2, 125.7, 104.3, 78.0, 73.8, 72.4, 66.7, 52.4, 27.9, 27.4, 26.3, 23.3, 20.4, 18.8, -4.0, -4.3; **HRMS** (ESI) *m/z* calcd for $C_{28}H_{47}O_6Si_2$ [M + H]$^+$ 535.2906, found 535.2911.

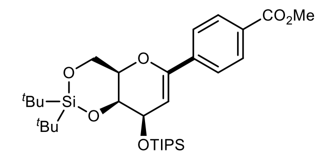

7g

Methyl 4-((4a*R*,8*R*,8a*S*)-2,2-di-*tert*-butyl-8-((triisopropylsilyl)oxy)-4,4a,8,8a-tetrahydropyrano[3,2-*d*][1,3,2]dioxasilin-6-yl)benzoate (7g). According to the general procedure A, glycal boronates **12g** (74.9 mg, 0.12 mmol, 1.20 equiv), Methyl 4-bromobenzoate (21.5 mg, 0.10 mmol, 1.00 equiv), $Pd(PPh_3)_2Cl_2$ (3.50 mg, 0.005 mmol, 5.00 mol%), K_3PO_4 (63.6 mg, 0.30 mmol, 3.00 equiv) were added to a one-dram vial with a screw-top septum, and the vial was then evacuated and refilled with N_2 (3×). Anhydrous DMF (2.00 mL) were added, and the reaction mixture was stirred at 33 °C for 48 h, cooled to rt, and concentrated. The crude material was purified by column chromatography on SiO_2 (Petroleum ether:Ether, 1:0-30:1) to afford **7g** (52.9 mg, 92%) as a white foam: **^{1}H NMR** (500 MHz, Acetone-*d*$_6$) δ 7.99 – 7.97 (m, 2H), 7.79 – 7.77 (m, 2H), 5.51 (t, *J* = 1.9, 1.9 Hz, 1H), 4.94 (dd, *J* = 4.7, 2.0 Hz, 1H), 4.64 – 4.62 (m, 1H), 4.46 – 4.40 (m, 2H), 4.30 (d, *J* = 1.8 Hz, 1H), 3.88 (s, 3H), 1.23 – 1.14 (m, 21H), 1.10 (s, 9H), 0.89 (s, 9H); **^{13}C NMR** (126 MHz, Acetone-*d*$_6$) δ 166.9, 150.2, 140.0, 130.5, 130.0, 125.4, 103.0, 75.2, 69.8, 68.2, 67.9, 52.3, 28.2, 27.5, 24.0, 21.2, 18.6, 18.5, 13.2; **HRMS** (ESI) m/z calcd for $C_{31}H_{52}O_6Si_2Na$ [M + Na]$^+$ 599.3195, found 599.3200.

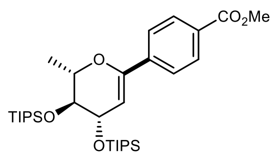

7h

Methyl 4-((2*S*,3*S*,4*S*)-2-methyl-3,4-bis((triisopropylsilyl)oxy)-3,4-dihydro-2*H*-pyran-6-yl)benzoate (7h). According to the general procedure A, glycal boronates **12b** (74.9 mg, 0.12 mmol, 1.20 equiv), methyl 4-bromobenzoate (21.5 mg, 0.10 mmol, 1.00 equiv), $Pd(PPh_3)_2Cl_2$ (3.50 mg, 0.005 mmol, 5.00 mol%), K_3PO_4 (63.6 mg, 0.30 mmol, 3.00 equiv) were added to a one-dram vial with a screw-top septum, and the vial was then evacuated and refilled with N_2 (3×). Anhydrous DMF (2.00 mL) were added, and the reaction mixture was stirred at 33 °C for 48 h, cooled to rt, and concentrated. The crude material was purified by column chromatography on SiO_2 (Petroleum ether: ether,

1:0 - 50:1) to afford **7h** (52.6 mg, 91%) as a colorless oil: **^{1}H NMR** (400 MHz, $CDCl_3$) δ 8.00 (d, *J* = 8.2 Hz, 2H), 7.66 (d, *J* = 8.3 Hz, 2H), 5.47 (d, *J* = 5.1 Hz, 1H), 4.53 – 4.48 (m, 1H), 4.25 – 4.22 (m, 1H), 4.01 – 3.99 (m, 1H), 3.91 (s, 3H), 1.44 (d, *J* = 7.0 Hz, 3H), 1.06 (dd, *J* = 14.2, 4.8 Hz, 42H); **^{13}C NMR** (101 MHz, $CDCl_3$) δ 167.1, 148.9, 140.9, 129.7, 129.6, 125.2, 99.1, 75.5, 73.2, 67.4, 52.2, 18.3(3), 18.2, 16.1, 12.7, 12.6; **HRMS** (ESI) *m/z* calcd for $C_{32}H_{57}O_5Si_2$ $[M + H]^+$ 577.3739, found 577.3749.

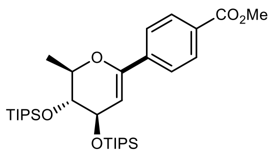

7i

Methyl 4-((2*R*,3*R*,4*R*)-2-methyl-3,4-bis((triisopropylsilyl)oxy)-3,4-dihydro-2*H*-pyran-6-yl)benzoate (7i). According to the general procedure A, glycal boronates **12i** (74.9 mg, 0.12 mmol, 1.20 equiv), methyl 4-bromobenzoate (21.5 mg, 0.10 mmol, 1.00 equiv), $Pd(PPh_3)_2Cl_2$ (3.50 mg, 0.005 mmol, 5.00 mol%), K_3PO_4 (63.6 mg, 0.30 mmol, 3.00 equiv) were added to a one-dram vial with a screw-top septum, and the vial was then evacuated and refilled with N_2 (3×). Anhydrous DMF (2.00 mL) were added, and the reaction mixture was stirred at 33 °C for 48 h, cooled to rt, and concentrated. The crude material was purified by column chromatography on SiO_2 (Petroleum ether:Ether, 1:0-50:1) to afford **7i** (51.8 mg, 90%) as a colorless oil: **^{1}H NMR** (500 MHz, Acetone-*d*$_6$) δ 8.01 – 7.99 (m, 2H), 7.80 – 7.78 (m, 2H), 5.69 (dd, *J* = 5.2, 1.5 Hz, 1H), 4.63 – 4.58 (m, 1H), 4.35 – 4.33 (m, 1H), 4.14 – 4.12 (m, 1H), 3.88 (s, 3H), 1.49 (d, *J* = 7.0 Hz, 3H), 1.19 – 1.07 (m, 42H); **^{13}C NMR** (126 MHz, Acetone-d6) δ 166.9, 149.5, 141.4, 130.8, 130.1, 125.9, 99.5, 76.1, 73.9, 68.2, 52.3, 18.6, 18.5(2), 18.4, 16.3, 13.3, 13.2; **HRMS** (ESI) *m/z* calcd for $C_{32}H_{57}O_5Si_2$ $[M + H]^+$ 577.3739, found 577.3748.

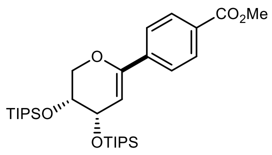

7j

Methyl 4-((3*R*,4*S*)-3,4-bis((triisopropylsilyl)oxy)-3,4-dihydro-2*H*-pyran-6-yl)benzoate (7j). According to the general procedure A, glycal boronates **12j** (63.1 mg, 0.12 mmol, 1.20 equiv), methyl 4-bromobenzoate (21.5 mg, 0.10 mmol, 1.00 equiv), $Pd(PPh_3)_2Cl_2$ (3.50 mg, 0.005 mmol, 5.0 mol%), K_3PO_4 (63.6 mg, 0.30 mmol, 3.00 equiv) were added to a one-dram vial with a screw-top septum, and the vial was then evacuated and refilled with N_2 (3×). Anhydrous DMF (2.00 mL) were added, and the reaction mixture was stirred at 33 °C for 48 h, cooled to rt, and concentrated. The crude material was purified by column chromatography on SiO_2 (Petroleum ether:Ether, 1:0-50:1) to afford **7j** (50.0 mg, 89%) as a colorless oil: **^{1}H NMR** (500 MHz, Acetone-*d*$_6$) δ 8.00 – 7.98 (m, 2H), 7.76 – 7.74 (m, 2H), 5.73 (d, *J* = 5.8 Hz, 1H), 4.59 – 4.57 (m, 1H), 4.30 – 4.26 (m, 1H), 4.22 – 4.19 (m, 1H), 4.13 – 4.10 (m, 1H), 3.88 (s, 3H), 1.20 – 1.09 (m, 42H); **^{13}C NMR** (126 MHz, Acetone-*d*$_6$) δ 166.8, 152.2, 140.1, 131.0, 130.2,

125.8, 101.3, 69.5, 66.1, 65.9, 52.4, 18.7, 18.6, 18.5(2), 13.7, 13.2; **HRMS** (ESI) *m/z* calcd for $C_{31}H_{54}O_5Si_2Na$ [M + Na] $^+$ 585.3402, found 585.3414.

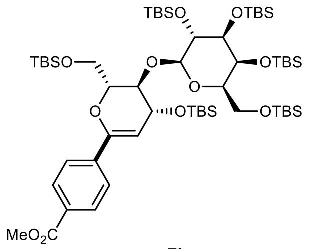

7k

Methyl 4-((2*R*,3*R*,4*R*)-4-((*tert*-butyldimethylsilyl)oxy)-2-(((*tert*-butyldimethylsilyl)oxy)methyl)-3-(((2*S*,3*R*,4*S*,5*S*,6*R*)-3,4,5-tris((*tert*-butyldimethylsilyl)oxy)-6-(((*tert*-butyldimethylsilyl)oxy)methyl)tetrahydro-2*H*-pyran-2-yl)oxy)-3,4-dihydro-2*H*-pyran-6-yl)benzoate (7k). According to the general procedure A, glycal boronates **12k** (140 mg, 0.12 mmol, 1.20 equiv), methyl 4-bromobenzoate (21.5 mg, 0.10 mmol, 1.00 equiv), $Pd(PPh_3)_2Cl_2$ (3.50 mg, 0.005 mmol, 5.00 mol%), K_3PO_4 (63.60 mg, 0.30 mmol, 3.00 equiv) were added to a one-dram vial with a screw-top septum, and the vial was then evacuated and refilled with N_2 (3×). Anhydrous DMF (2.00 mL) were added, and the reaction mixture was stirred at 33 °C for 48 h, cooled to rt, and concentrated. The crude material was purified by column chromatography on SiO_2 (Petroleum ether:EtOAc, 1:0-50:1) to afford **7k** (107 mg, 95%) as a white foam: **^{1}H NMR** (400 MHz, Acetone-d_6) δ 8.00 – 7.98 (m, 2H), 7.79 – 7.77 (m, 2H), 5.51 (d, *J* = 3.5 Hz, 1H), 4.70 (d, *J* = 7.3 Hz, 1H), 4.47 (dd, *J* = 5.5, 3.5 Hz, 1H), 4.29 (dd, *J* = 11.6, 4.2 Hz, 1H), 4.23 – 4.20 (m, 2H), 4.06 (dd, *J* = 7.4, 5.5 Hz, 1H), 4.02 (dd, *J* = 11.6, 2.6 Hz, 1H), 3.96 (dd, *J* = 9.3, 7.3 Hz, 1H), 3.88 (s, 3H), 3.83 – 3.79 (m, 1H), 3.69 – 3.66 (m, 2H), 3.47 (dd, *J* = 9.5, 5.1 Hz, 1H), 0.99 (d, *J* = 3.0 Hz, 18H), 0.95 – 0.94 (m, 27H), 0.92 (s, 9H), 0.23 – 0.18 (m, 18H), 0.14 – 0.12 (m, 9H), 0.09 (s, 9H); **^{13}C NMR** (101 MHz, Acetone-d_6) δ 166.8, 150.7, 140.0, 130.8, 130.0, 125.7, 103.3, 102.5, 79.1, 77.6, 76.1, 74.6, 72.9, 72.3, 69.0, 61.7, 61.2, 52.3, 27.3, 27.1, 26.7, 26.5, 26.4, 26.2, 19.7, 19.3, 18.9, 18.8, 18.7(2), -2.5, -3.1(2), -3.4, -3.7, -3.9, -4.4, -4.8, -5.0, -5.1(2); **HRMS** (ESI) m/z calcd for $C_{56}H_{110}O_{11}Si_6Na$ [M + Na]$^+$ 1149.6556, found 1149.6544.

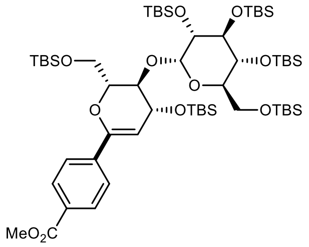

7l

Methyl 4-((2*R*,3*R*,4*R*)-4-((*tert*-butyldimethylsilyl)oxy)-2-(((*tert*-butyldimethylsilyl)oxy)methyl)-3-(((2*R*,3*R*,4*S*,5*R*,6*R*)-3,4,5-tris((*tert*-butyldimethylsilyl)oxy)-6-(((*tert*-butyldimethylsilyl)oxy)methyl)tetrahydro-2*H*-pyran-2-yl)oxy)-3,4-dihydro-2*H*-pyran-6-yl)benzoate (7l). According to the general

procedure A, glycal boronates **12l** (141 mg, 0.12 mmol, 1.20 equiv), methyl 4-bromobenzoate (21.5 mg, 0.10 mmol, 1.00 equiv), $Pd(PPh_3)_2Cl_2$ (3.50 mg, 0.005 mmol, 5.00 mol%), K_3PO_4 (63.6 mg, 0.30 mmol, 3.00 equiv) were added to a one-dram vial with a screw-top septum, and the vial was then evacuated and refilled with N_2 (3×). Anhydrous DMF (2.00 mL) were added, and the reaction mixture was stirred at 33 °C for 48 h, cooled to rt, and concentrated. The crude material was purified by column chromatography on SiO_2 (Petroleum ether:Ether, 1:0-50:1) to afford **7l** (101 mg, 90%) as a colorless oil: **^{1}H NMR** (500 MHz, Acetone-d_6) δ 8.01 – 7.98 (m, 2H), 7.83 – 7.81 (m, 2H), 5.62 (dd, *J* = 4.4, 1.0 Hz, 1H), 5.12 (d, *J* = 3.5 Hz, 1H), 4.52 – 4.49 (m, 1H), 4.37 – 4.35 (m, 1H), 4.13 (dd, *J* = 11.4, 9.1 Hz, 1H), 4.07 – 4.04 (m, 2H), 3.99 – 3.84 (m, 9H), 0.95 – 0.91 (m, 45H), 0.87 (s, 9H), 0.20 – 0.13 (m, 21H), 0.07 – 0.04 (m, 12H); **^{13}C NMR** (126 MHz, Acetone-d_6) δ 166.8, 150.4, 140.6, 130.9, 130.0, 126.0, 100.8, 95.9, 80.7, 77.6, 75.6, 74.1, 72.6, 72.2, 66.9, 63.3, 63.0, 52.3, 26.4(2), 26.3(3), 19.0(2), 18.8, 18.6, 18.5, -3.5, -3.8, -4.1, -4.3(2), -4.5, -4.9, -5.0, -5.1; **HRMS** (ESI) m/z calcd for $C_{56}H_{110}O_{11}Si_6Na$ $[M + Na]^+$ 1149.6556, found 1149.6559.

2.4. Detailed experimental procedures for the one pot two-step reaction

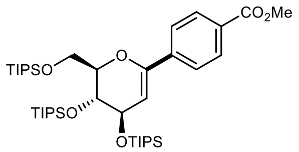

15a

Methyl 4-((2*R*,3*R*,4*R*)-3,4-bis((triisopropylsilyl)oxy)-2-(((triisopropylsilyl)oxy)methyl)-3,4-dihydro-2*H*-pyran-6-yl)benzoate (15a). According to the general procedure B, 3,4,6-tris-O-(triisopropylsilyl)-D-glucal (111 mg, 0.18 mmol, 1.80 equiv), B_2Pai_2 (43.0 mg, 0.12 mmol, 1.20 equiv), $[IrOMe(cod)]_2$ (1.60 mg, 0.0024 mmol, 2.40 mol%), dtbbpy (1.30 mg, 0.0048 mmol, 4.80 mol%) were added to a one-dram vial with a screw-top septum, and the vial was then evacuated and refilled with N_2 (3×). Anhydrous octane (1.00 mL) was added, and the reaction mixture was stirred at 80 °C for 24 h. The reaction mixture was cooled to rt and concentrated under vaccum, then methyl 4-bromobenzoate (21.5 mg, 0.10 mmol, 1.00 equiv), $Pd(PPh_3)_2Cl_2$ (7.00 mg, 0.01 mmol, 10.0 mol%), and K_3PO_4 (63.6 mg, 0.30 mmol, 3.00 equiv) were added. After being evacuated and refilled with N_2 (3×), anhydrous DMF (2.00 mL) were added to the reaction mixture. The resulting mixture was stirred at 33 °C for 48 h, then cooled to rt and concentrated, yielding compound **15a** in an 88% NMR yield.

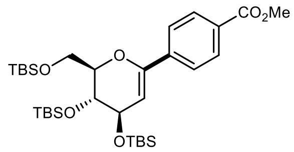

7e

Methyl 4-((2*R*,3*R*,4*R*)-3,4-bis((*tert*-butyldimethylsilyl)oxy)-2-(((*tert*-butyldimethylsilyl)oxy)methyl)-3,4-dihydro-2*H*-pyran-6-yl)benzoate (7e)

According to the general procedure B, 3,4,6-tri-*O*-(*tert*-butyldimethylsilyl)-D-glucal (88.0 mg, 0.18 mmol, 1.80 equiv), B_2Pai_2 (43.0 mg, 0.12 mmol, 1.20 equiv), $[IrOMe(cod)]_2$ (1.60 mg, 0.0024 mmol, 2.40 mol%), dtbbpy (1.30 mg, 0.0048 mmol, 4.80 mol%) were added to a one-dram vial with a screw-top septum, and the vial was then evacuated and refilled with N_2 (3×). Anhydrous octane (1.00 mL) was added, and the reaction mixture was stirred at 80 °C for 24 h. The reaction mixture cooled to rt and concentrated under vaccum, then methyl 4-bromobenzoate (21.5 mg, 0.10 mmol, 1.00 equiv), $Pd(PPh_3)_2Cl_2$ (7.00 mg, 0.01 mmol, 10.0 mol%) , K_3PO_4 (63.6 mg, 0.30 mmol, 3.00 equiv) were added. After being evacuated and refilled with N_2 (3×), anhydrous DMF (2.00 mL) were added to the reaction mixture. The resulting mixture was stirred at 33 °C for 48 h, then cooled to rt and concentrated. The crude material was purified by column chromatography on SiO_2 (Petroleum ether:Ether, 1:0-50:1) to afford **7e** (56.4 mg, 91%) as a colorless oil.

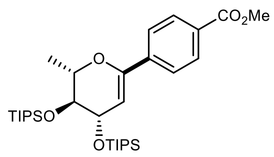

7h

Methyl 4-((2*S*,3*S*,4*S*)-2-methyl-3,4-bis((triisopropylsilyl)oxy)-3,4-dihydro-2*H*-pyran-6-yl)benzoate (7h). According to the general procedure B, 3,4-di-*O*-triisopropylsilyl-L-rhamnal (79.6 mg, 0.18 mmol, 1.80 equiv), B_2Pai_2 (43.0 mg, 0.12 mmol, 1.20 equiv), $[IrOMe(cod)]_2$ (1.60 mg, 0.0024 mmol, 2.40 mol%), dtbbpy (1.30 mg, 0.0048 mmol, 4.80 mol%) were added to a one-dram vial with a screw-top septum, and the vial was then evacuated and refilled with N_2 (3×). Anhydrous octane (1.00 mL) was added, and the reaction mixture was stirred at 80 °C for 24 h. The reaction mixture cooled to rt and concentrated under vaccum, then 4-bromobenzoate (21.5 mg, 0.10 mmol, 1.00 equiv), $Pd(PPh_3)_2Cl_2$ (7.00 mg, 0.01 mmol, 10.0 mol%) , K_3PO_4 (63.6 mg, 0.30 mmol, 3.00 equiv) were added. After being evacuated and refilled with N_2 (3×), anhydrous DMF (2.00 mL) were added to the reaction mixture. The resulting mixture was stirred at 33 °C for 48 h, then cooled to rt and concentrated. The crude material was purified by column chromatography on SiO_2 (Petroleum ether:Ether, 1:0-50:1) to afford **7h** (48.5 mg, 84%) as a colorless oil.

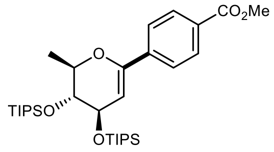

7i

Methyl 4-((2*R*,3*R*,4*R*)-2-methyl-3,4-bis((triisopropylsilyl)oxy)-3,4-dihydro-2*H*-pyran-6-yl)benzoate (7i). According to the general procedure B, 3,4-di-*O*-triisopropylsilyl-6-deoxy-D-glucal (79.6 mg, 0.18 mmol, 1.80 equiv), B_2Pai_2 (43.0 mg, 0.12 mmol, 1.20 equiv), $[IrOMe(cod)]_2$ (1.60 mg, 0.0024 mmol, 2.40 mol%), dtbbpy (1.30 mg, 0.0048 mmol, 4.80 mol%) were added to a one-dram vial with a screw-top septum, and the vial was then evacuated and refilled with N_2 (3x). Anhydrous octane (1.00 mL) was added, and the reaction mixture was stirred at 80 °C for 24 h. The reaction mixture cooled to rt and concentrated under vaccum, then 4-bromobenzoate (21.5 mg, 0.10 mmol, 1.00 equiv), $Pd(PPh_3)_2Cl_2$ (7.00 mg, 0.01 mmol, 10.0 mol%) , K_3PO_4 (63.6 mg, 0.30 mmol, 3.00 equiv) were added. After being evacuated and refilled with N_2 (3×), anhydrous DMF (2.00 mL) were added to the reaction mixture. The resulting mixture was stirred at 33 °C for 48 h, then cooled to rt and concentrated. The crude material was purified by column chromatography on SiO_2 (Petroleum ether:Ether, 1:0-50:1) to afford **7i** (52.9 mg, 92%) as a colorless oil: **^{1}H NMR** (500 MHz, Acetone-*d*$_6$) δ 8.01 – 7.99 (m, 2H), 7.80 – 7.78 (m, 2H), 5.69 (dd, *J* = 5.2, 1.5 Hz, 1H), 4.63 – 4.58 (m, 1H), 4.35 – 4.33 (m, 1H), 4.14 – 4.12 (m, 1H), 3.88 (s, 3H), 1.49 (d, *J* = 7.0 Hz, 3H), 1.19 – 1.07 (m, 42H); **^{13}C NMR** (126 MHz, Acetone-d6) δ 166.9, 149.5, 141.4, 130.8, 130.1, 125.9, 99.5, 76.1, 73.9, 68.2, 52.3, 18.6, 18.5(2), 18.4, 16.3, 13.3, 13.2; **HRMS** (ESI) *m/z* calcd for $C_{32}H_{57}O_5Si_2$ $[M + H]^+$ 577.3739, found 577.3748.

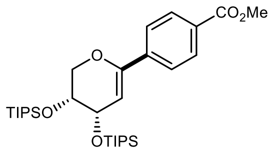

7j

Methyl 4-((3*R*,4*S*)-3,4-bis((triisopropylsilyl)oxy)-3,4-dihydro-2*H*-pyran-6-yl)benzoate (7j). According to the general procedure B, 3,4-di-*O*-triisopropylsilyl-6-deoxy-L-arabinal (77.0 mg, 0.18 mmol, 1.80 equiv), B_2Pai_2 (43.0 mg, 0.12 mmol, 1.20 equiv), $[IrOMe(cod)]_2$ (1.60 mg, 0.0024 mmol, 2.40 mol%), dtbbpy (1.30 mg, 0.0048 mmol, 4.80 mol%) were added to a one-dram vial with a screw-top septum, and the vial was then evacuated and refilled with N_2 (3×). Anhydrous octane (1.00 mL) was added, and the reaction mixture was stirred at 80 °C for 24 h. The reaction mixture cooled to rt and concentrated under vaccum, then 4-bromobenzoate (21.5 mg, 0.10 mmol, 1.00 equiv), $Pd(PPh_3)_2Cl_2$ (7.00 mg, 0.01 mmol, 10.0 mol%) , K_3PO_4 (63.60 mg, 0.30 mmol, 3.00 equiv) were added. After being evacuated and refilled with N_2 (3×), anhydrous DMF (2.00 mL) were added to the reaction mixture. The resulting mixture was stirred at 33 °C for 48 h, then cooled to rt and concentrated. The crude material was purified by column chromatography on SiO_2 (Petroleum ether:Ether, 1:0-50:1) to afford **7j** (48.3 mg, 84%) as a colorless oil.

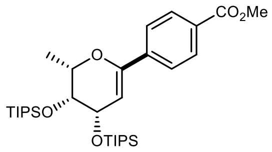

7m

Methyl 4-((2*S*,3*R*,4*S*)-2-methyl-3,4-bis((triisopropylsilyl)oxy)-3,4-dihydro-2*H*-pyran-6-yl)benzoate (7m). According to the general procedure B, 3,4-di-*O*-triisopropylsilyl-L- Fucal (79.6 mg, 0.18 mmol, 1.80 equiv), B_2Pai_2 (43.0 mg, 0.12 mmol, 1.20 equiv), $[IrOMe(cod)]_2$ (1.60 mg, 0.0024 mmol, 2.40 mol%), dtbbpy (1.30 mg, 0.0048 mmol, 4.80 mol%) were added to a one-dram vial with a screw-top septum, and the vial was then evacuated and refilled with N_2 (3×). Anhydrous octane (1.00 mL) was added, and the reaction mixture was stirred at 80 °C for 24 h. The reaction mixture cooled to rt and concentrated under vaccum, then 4-bromobenzoate (21.5 mg, 0.10 mmol, 1.00 equiv), $Pd(PPh_3)_2Cl_2$ (7.00 mg, 0.01 mmol, 10.0 mol%) , K_3PO_4 (63.6 mg, 0.30 mmol, 3.00 equiv) were added, evacuated and refilled with N_2 (3×). After being evacuated and refilled with N_2 (3×), anhydrous DMF (2.00 mL) were added to the reaction mixture. The resulting mixture was stirred at 33 °C for 48 h, then cooled to rt and concentrated. The crude material was purified by column chromatography on SiO_2 (Petroleum ether:Ether, 1:0-50:1) to afford **7m** (52.9 mg, 92%) as a colorless oil: **^{1}H NMR** (400 MHz, Acetone-*d*$_6$) δ 8.03 – 7.96 (m, 2H), 7.77 – 7.70 (m, 2H), 5.57 – 5.43 (m, 1H), 4.76 (t, *J* = 3.8, 3.8 Hz, 1H), 4.48 – 4.43 (m, 1H), 4.24 (t, *J* = 3.2, 3.2 Hz, 1H), 3.88 (s, 3H), 1.52 (d, *J* = 6.6 Hz, 3H), 1.20 – 1.11 (m, 42H); **^{13}C NMR** (101 MHz, Acetone-*d*$_6$) δ 166.9, 150.3, 140.7, 130.7, 130.1, 125.7, 101.9, 75.1, 71.4, 52.3, 18.8,

18.7(2), 14.0, 13.5, 13.4; **HRMS** (ESI) *m/z* calcd for $C_{32}H_{57}O_5Si_2$ [M + H]$^+$ 577.3739, found 577.3748.

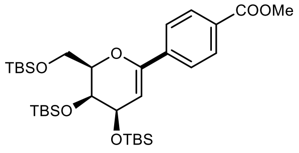

7n

Methyl 4-((2*R*,3*S*,4*R*)-3,4-bis((*tert*-butyldimethylsilyl)oxy)-2-(((*tert*-butyldimethylsilyl)oxy)methyl)-3,4-dihydro-2*H*-pyran-6-yl)benzoate (7n). According to the general procedure B, 3,4,6-tris-*O*-(*tert*-butyldimethylsilyl)-D-galactal (87.9 mg, 0.18 mmol, 1.80 equiv), B_2Pai_2 (43.0 mg, 0.12 mmol, 1.20 equiv), $[IrOMe(cod)]_2$ (1.60 mg, 0.0024 mmol, 2.40 mol%), dtbbpy (1.30 mg, 0.0048 mmol, 4.80 mol%) were added to a one-dram vial with a screw-top septum, and the vial was then evacuated and refilled with N_2 (3×). Anhydrous octane (1.00 mL) was added, and the reaction mixture was stirred at 80 °C for 24 h. The reaction mixture cooled to rt and concentrated under vaccum, then 4-bromobenzoate (21.5 mg, 0.10 mmol, 1.00 equiv), $Pd(PPh_3)_2Cl_2$ (7.00 mg, 0.01 mmol, 10.0 mol%) , K_3PO_4 (63.6 mg, 0.30 mmol, 3.00 equiv) were added. After being evacuated and refilled with N_2 (3×), anhydrous DMF (2.00 mL) were added to the reaction mixture. The resulting mixture was stirred at 33 °C for 48 h, then cooled to rt and concentrated. The crude material was purified by column chromatography on SiO_2 (Petroleum ether:ether, 1:0-50:1) to afford **7n** (56.0 mg, 90%) as a colorless oil: **^{1}H NMR** (500 MHz, Acetone-*d*$_6$) δ 8.00 – 7.97 (m, 2H), 7.78 – 7.75 (m, 2H), 5.50 (d, *J* = 4.0 Hz, 1H), 4.53 (s, 1H), 4.30 – 4.27 (m, 1H), 4.24 (t, *J* = 3.6, 3.6 Hz, 1H), 4.14 (dd, *J* = 11.2, 7.8 Hz, 1H), 3.99 (dd, *J* = 11.2, 4.0 Hz, 1H), 3.88 (s, 3H), 0.96 (s, 9H), 0.93 (d, *J* = 2.5 Hz, 18H), 0.20 (s, 3H), 0.18 (s, 9H), 0.07 (s, 6H); **^{13}C NMR** (126 MHz, Acetone-*d*$_6$) δ 166.8, 150.0, 140.5, 130.8, 130.1, 125.8, 102.3, 80.4, 68.9, 67.2, 61.8, 52.3, 26.5, 26.4, 26.3, 18.9, 18.8, -3.7, -4.1, -4.4, -4.6, -5.0, -5.1; **HRMS** (ESI) *m/z* calcd for $C_{32}H_{59}O_6Si_3$ [M + H]$^+$ 623.3614, found 623.3613.

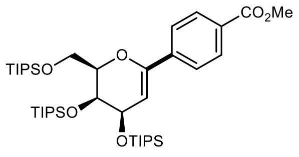

7o

Methyl 4-((2*R*,3*S*,4*R*)-3,4-bis((triisopropylsilyl)oxy)-2-(((triisopropylsilyl)oxy)methyl)-3,4-dihydro-2*H*-pyran-6-yl)benzoate (7o). According to the general procedure B, 3,4,6-tris-*O*-(triisopropylsilyl)-D-galactal (111 mg, 0.18 mmol, 1.80 equiv), B_2Pai_2 (43.0 mg, 0.12 mmol, 1.20 equiv), $[IrOMe(cod)]_2$ (1.60 mg, 0.0024 mmol, 2.40 mol%), dtbbpy (1.30 mg, 0.0048 mmol, 4.80 mol%) were added to a one-dram vial with a screw-top septum, and the vial was then evacuated and refilled with N_2 (3×). Anhydrous octane (1.00 mL) was added, and the reaction mixture was stirred at 80 °C for 24 h. The reaction mixture cooled to rt and concentrated under vaccum, then 4-bromobenzoate (21.5 mg, 0.10 mmol, 1.00 equiv), $Pd(PPh_3)_2Cl_2$ (7.00

mg, 0.01 mmol, 10.0 mol%) , K_3PO_4 (63.6 mg, 0.30 mmol, 3.00 equiv) were added. After being evacuated and refilled with N_2 (3×), anhydrous DMF (2.00 mL) were added to the reaction mixture. The resulting mixture was stirred at 33 °C for 48 h, then cooled to rt and concentrated. The crude material was purified by column chromatography on SiO_2 (Petroleum ether:Ether, 1:0-50:1) to afford **7o** (67.3 mg, 90%) as a colorless oil: **^{1}H NMR** (500 MHz, Acetone-d_6) δ 7.99 – 7.97 (m, 2H), 7.80 (d, J = 8.2 Hz, 2H), 5.67 – 5.66 (m, 1H), 4.58 – 4.22 (m, 5H), 3.88 (s, 3H), 1.27 – 1.02 (m, 63H); **^{13}C NMR** (126 MHz, Acetone-d_6) δ 166.8, 150.0, 140.4, 131.0, 130.0, 126.0, 101.8, 81.6, 70.6, 62.0, 52.3, 18.8, 18.7(2), 18.6, 18.4(2), 13.5, 12.8. **HRMS** (ESI) *m/z* calcd for $C_{41}H_{77}O_6Si_3$ $[M + H]^+$ 749.5022, found 749.5034.

2.5. Detailed experimental procedures for on-DNA glycal-based Suzuki–Miyaura cross-couplings

Materials and Equipment:

The chemically modified DNA oligonucleotide headpiece (5'-/5Phos/GAGTCA/iSp9/iUniAmM/iSp9/TGACTCCC-3', Figure S1) was synthesized at HitGen Inc.

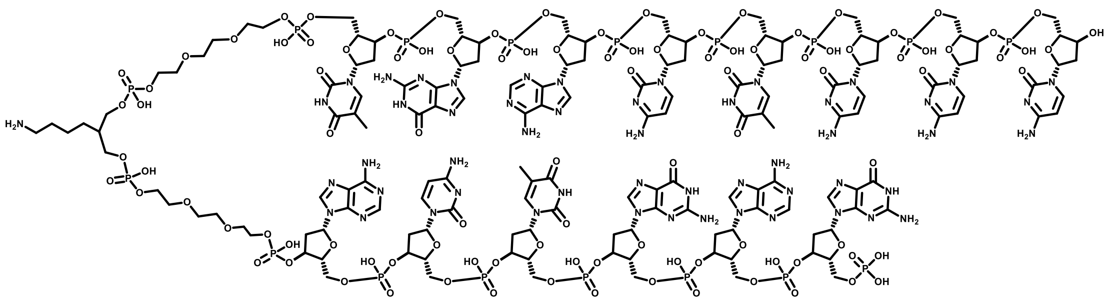

SupplementaryFigure 1. Headpiece (5'-/5Phos/GAGTCA/iSp9/iUniAmM/iSp9/TGACTCCC-3'), MW = 4937.2

General procedure for the analysis of oligonucleotide compositions

1. DNA was characterized on UPLC-MS system equipped with PDA and QDa mass detector (Waters, MA, USA). The UPLC system was set as following. Column: Acquity UPLC Oligonucleotide BEH C18 Column, 130A, 1.7 μm, 2.1 mm × 50 mm, and maintained at 40 °C. Mobile phases: 0.75 % HFIP/0.0375 % DIPEA/10 μM EDTA in HPLC grade water (A) and 0.75 % HFIP/0.0375 % DIPEA/10 μM EDTA in 80/20 HPLC grade methanol/water (B). Eluting gradient: from 24 % to 44 % of B in 1.2 minutes, flow rate 0.3 ml/min. Absorption was detected at 260 nm. Electrospray ionization (ESI) probe temperature was 600 °C, source temperature was 120 °C and ESI capillary was 0.8 kV. Mass detector (QDa) was operated at negative full scan mode in the range of 500-1200 (m/z). Data was analyzed by ProMass HR 2.0 (Novatia, Pennsylvania, USA) and MassLynx4.1 (Waters, MA, USA).

2. DNA was characterized on UPLC-MS system equipped with PDA and QDa mass detector (Waters, MA, USA). The UPLC system was set as following. Column: Acquity UPLC AdvanceBio Oligonucleotides C18 Column, 100A, 2.7 μm, 2.1 mm × 50 mm, and maintained at 40 °C. Mobile phases: 0.75 % HFIP/0.0375 % DIPEA/10 μM EDTA in HPLC grade water (A) and 0.75 % HFIP/0.0375 % DIPEA/10 μM EDTA in 80/20 HPLC grade methanol/water (B). Eluting gradient: from 24 % to 44 % of B in 1 minutes, flow rate 0.4 ml/min. The total analysis time was 2.5 min. Absorption was detected at 260 nm. Electrospray ionization (ESI) probe temperature was 600 °C, source temperature was 120 °C and ESI capillary was 0.8 kV. Mass detector (QDa) was operated at negative full scan mode in the range of 630-1200 (m/z). Data was analyzed by ProMass HR 2.0 (Novatia, Pennsylvania, USA) and MassLynx4.1 (Waters, MA,

USA).

General method for conversion calculation of DNA tagged material through LCMS

The conversions for DNA-encoded chemistries were determined by intensity calculation in TIC trace.[21,22] DNA recovery rate of the reaction was assumed as 100%.

$$\text{Conversion}(\%) = \frac{\text{Total detected intensity of } \textbf{target material}}{\text{Total detected intensity of } \textbf{DNA material}} \qquad \text{equation 1}$$

Example for yield calculation of 20h (5729.0 Da) determined by LCMS:
20h yield (%) =1.09E9/(2.17E7+6.49E7+9.96E6+1.02E8+1.09E9) = 84.6%

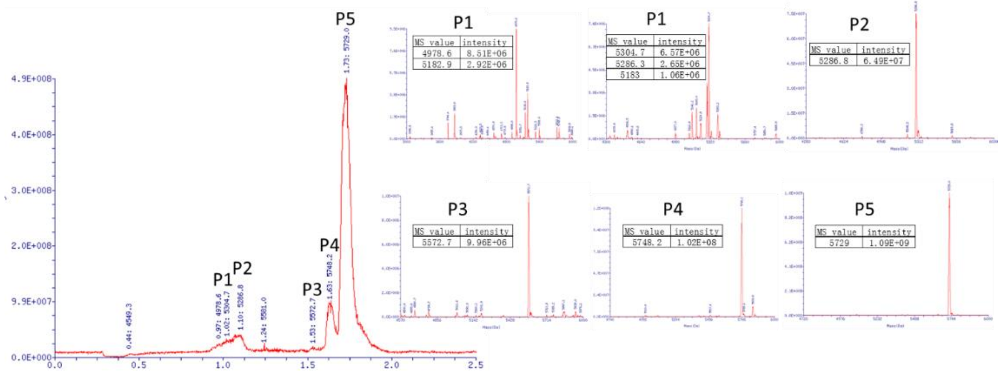

Supplementary Figure 2. Example for yield calculation of 20h (5729.0 Da) determined by LCMS

General procedures for on-DNA substrate preparation

Elaboration of HP to HP-AOP-NH_2 for Substrate Preparation. All substrates were prepared on HP that had been further elaborated by a long amino-terminating linker. This elaborated HP, HP-AOP-NH_2, was prepared through amidation of 1-(9H-fluoren-9-yl)-3-oxo-2, 7, 10, 13, 16-pentaoxa-4-azanonadecan-19-oic acid through the general acylation procedure and Fmoc deprotection.

The preparation of Fmoc protected HP

In a 15 mL Falcon tube, HP (2 µmol) was dissolved in sodium borate buffer (250 mM, pH 9.4, 2 mL). The stock solutions of 1-(9H-fluoren-9-yl)-3-oxo-2,7,10,13,16-pentaoxa-4-azanonadecan-19-oic acid (10 equiv, 100 µL, 200 mM in DMA), HATU (10 equiv, 50 µL, 400 mM in DMA), and DIPEA (10 equiv, 50 µL, 400 mM in DMA) were first chilled at 0 °C for 5 minutes and then mixed. The mixed reagents were further chilled at 4 °C for 5 minutes and finally added to HP solution in sodium borate buffer. The reaction was proceeded at room temperature for 30 minutes. The product was obtained by ethanol precipitation as described above. The DNA pellet was re-dissolved in 2 mL dd-H_2O and used directly without further purification (98 % yield). Calculated MS 5406.7, found 5407.2, observed m/z (product) =900.2 $[M-6H]^{6-}$

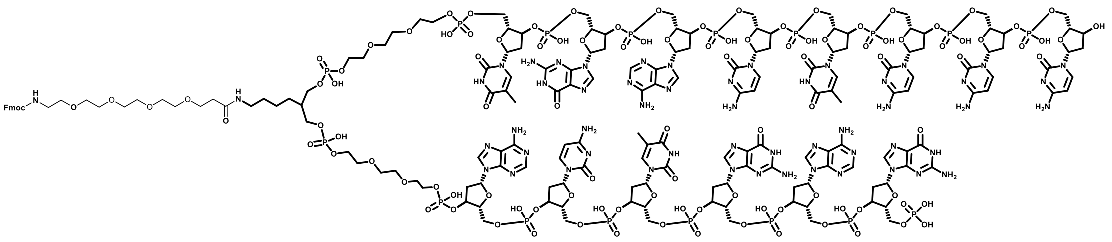

Supplementary Figure 3. Fmoc protected HP (MW = 5406.7)

The preparation of HP-AOP-NH2

In a 15 mL Falcon tube (Corning), 200 μL piperidine was added to a solution of Fmoc protected HP (2 μmoL in 2 mL dd-H_2O, Figure S2). The reaction was proceeded at room temperature for 30 minutes. The product was obtained by ethanol precipitation as described above (95 % yield). Calculated MS 5184.4, found 5184.8, observed m/z (product) = 647.1 $[M-8H]^{8-}$

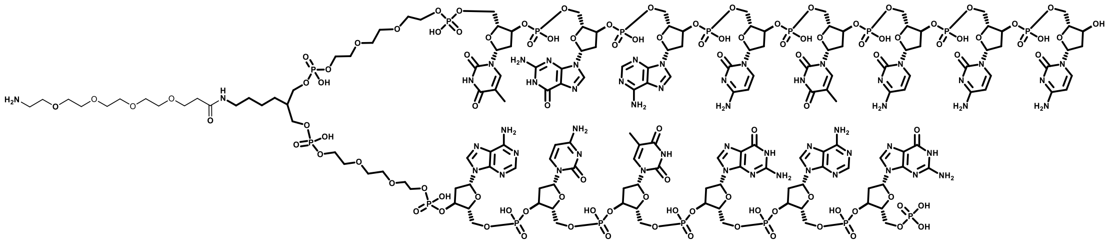

(Abbreviated as: 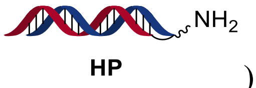

)

Supplementary Figure 4. HP-AOP-NH2 (MW = 5184.4)

on-DNA Substrates were obtained by treating headpiece and small molecules with the following protocols:

Amide formation (amines on DNA): HP-AOP-NH2 was dissolved in sodium borate buffer (250 mM, pH 9.4) to make 1 mM solution. Acid aryl-halide (200 mM in DMA, 50 equiv), HATU (200 mM in DMA, 50 equiv), and DIPEA (200 mM in DMA, 50 equiv) were firstly chilled at 4 °C for 5 minutes, and then mixed together. The mixture was chilled at 4 °C for 5 minutes, then transferred to HP-AOP-NH2 solution. The reaction was allowed to proceed at room temperature for 2 h.

General information of instruments for experiments

Reaction vessels: PCR MICROPLATE (Axygen 96 Well Half Skirt Microplate, Clear Single Notch, 300 μL, PCR-96M2-HS-C).

Sources of experimental reagents: All the reagents came from Enamine and Aladdin.

Experimental procedures for on-DNA chemistry

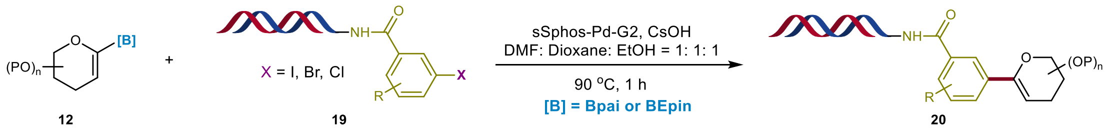

To each well of a 250 μL 96-well PCR microplate was added DNA (1.00 equiv., 10.0

nmol, 10.0 µL, 2.00 mM in H_2O), CsOH (100 equiv., 1.00 µmol, 2.00 µL, 0.50 M in H_2O), glycal boronates (250 equiv., 2.50 µmol, 16.70 µL, 0.15 M in DMF: Dioxane: EtOH = 1: 1: 1), and sSphos-Pd-G2 (2.00 equiv., 20.00 nmol, 1.00µL, 20.00 mM in DMF) sequentially. The solution was mixed with vortex for 30 seconds, then reacted at 90°C for 1 hour. When time's up, adding DDTC (Sodium diethyldithiocarbamate) (100 equiv., 1.00 µmol, 2.00 µL, 0.50 M in H_2O) to each well, the solution was mixed with vortex for 30 seconds, then reacted at 80°C for 10 min. When time's up, centrifuging under 4°C with 4000 rpm for 10 min. Remove precipitates and take supernatant for further ethanol precipitation, add 10% (v/v) 5 M NaCl solution and 3 times the volume of absolute ethanol to supernatant, cooled under -78°C for 2 hrs. Centrifuge under 4°C with 4000 rpm for 30 min. The precipitated material was isolated as a pellet by centrifugation and subsequent removal of the supernatant. 75% aq. ethanol was then added to the pellet and the mixture was centrifuged again. The supernatant again was discarded and the DNA pellet was dried under vacuum. The DNA pellet was redissolved in H_2O as 0.5 mM. Then take 1-2 nmol DNA for LC-MS detection.

Supplementary Table 1. On-DNA glycal-based Suzuki–Miyaura coupling of (hetero)aryl halides[a]

Substrate	Product Structure	X/B	Expected MW	Observed MW	Conversion
**20a**	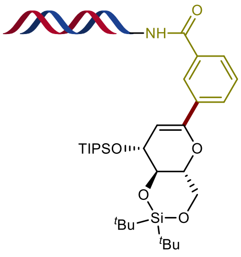	**20a-1**<br>X = Br<br>B = Bpai	5729.3	5728.8	92%
		**20a-2**<br>X = Cl<br>B = Bpai	5729.3	5728.4	92%
**20b**	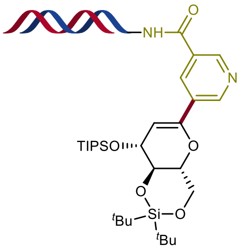	X = Br<br>B = Bpai	5730.3	5729.0	88%
**20c**	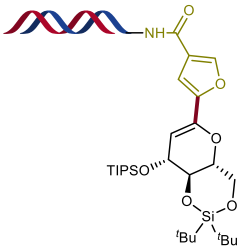	X = Br<br>B = Bpai	5719.3	5718.9	68%

**20d**	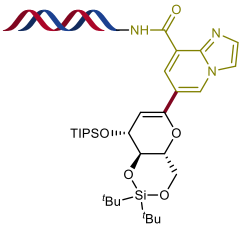	X = Br<br>B = Bpai	5769.3	5768.6	80%
**20e**	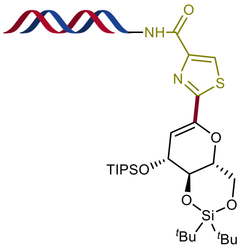	X = Br<br>B = Bpai	5736.3	5735.1	52%
**20f**	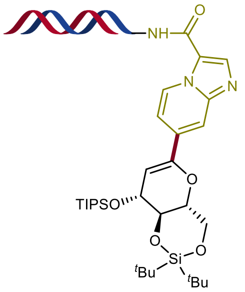	X = Br<br>B = Bpai	5769.3	5768.3	77%
**20g**	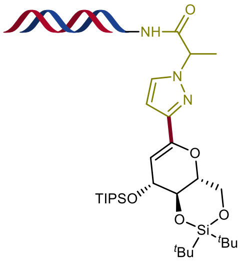	X = Br<br>B = Bpai	5747.3	5746.4	79%
**20h**	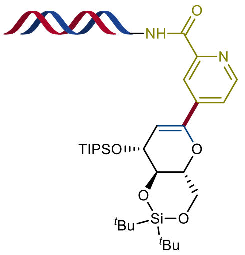	X = I<br>B = Bpai	5730.3	5729.0	85%
**20i**	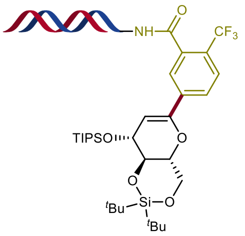	X = Cl<br>B = Bpai	5797.3	5797.0	93%
**20j**	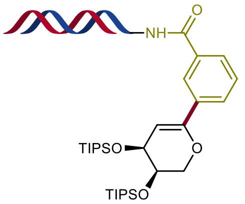	X = Br<br>B = BEpin	5715.3	5714.6	66%

**20k**	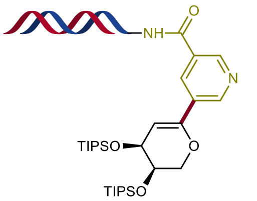	X = Br<br>B = BEpin	5716.3	5715.5	80%
**20l**	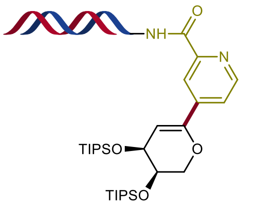	X = I<br>B = BEpin	5716.3	5714.8	71%
**20m**	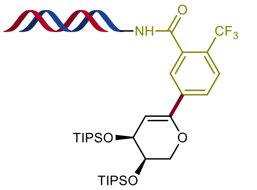	X = Cl<br>B = BEpin	5783.3	5782.4	76%
**20n**	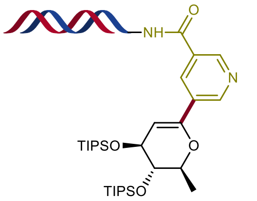	X = Br<br>B = Bpai	5730.3	5729.1	69%
**20o**	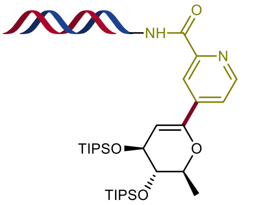	X = I<br>B = Bpai	5730.3	5729.6	68%
**20p**	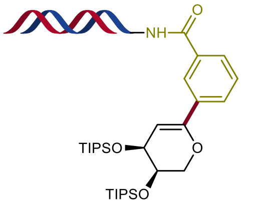	X = Br<br>B = BEpin	5729.3	5728.8	65%
**20q**	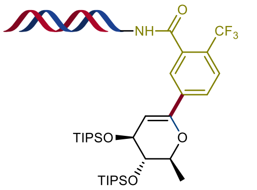	X = Cl<br>B = Bpai	5797.3	5797.0	73%
**20r**	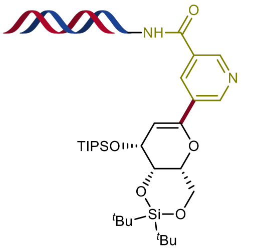	X = Br<br>B = BEpin	5730.3	5729.0	72%

**20s**	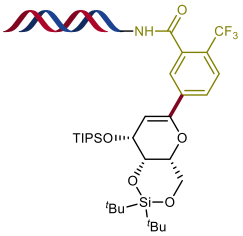	X = Cl<br>B = BEpin	5797.3	5796.6	71%

[a] Unless otherwise noted, all reactions were carried with **DNA 19** (1.00 equiv., 10.0 nmol, 10.0 µL, 2.00 mM in H_2O), CsOH (100 equiv., 1.00 µmol, 2.00 µL, 0.50 M in H_2O), **glycal boronates 12** (250 equiv., 2.50 µmol, 16.70 µL, 0.15 M in DMF: Dioxane: EtOH = 1: 1: 1), and sSphos-Pd-G2 (2.00 equiv., 20.00 nmol, 1.00µL, 20.00 mM in DMF) under 90°C for 1h.

Deconvoluted mass spectra of on-DNA substrates

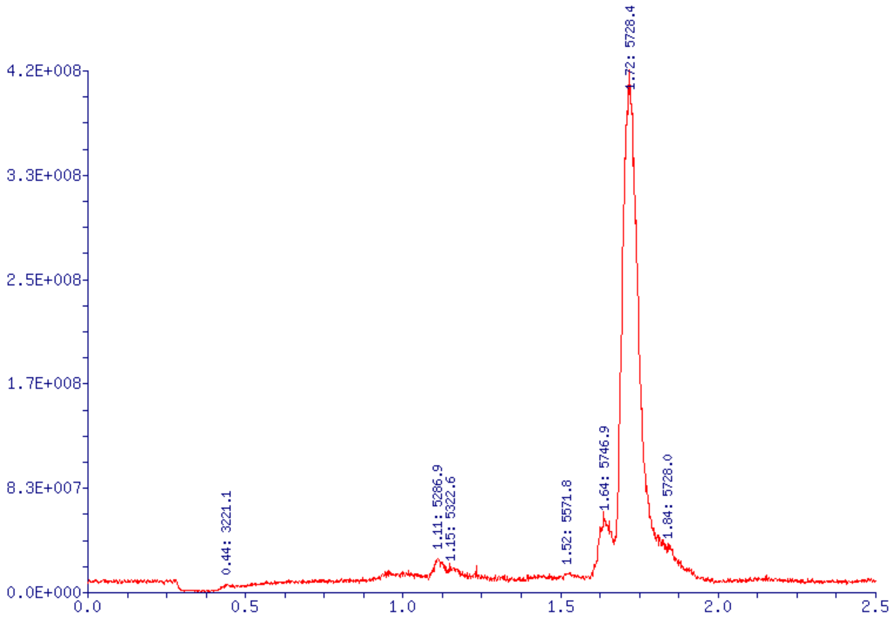

Supplementary Figure 5. LC-MS spectrum of **20a-1,** expected Mass: 5729.3; observed Mass: 5728.4

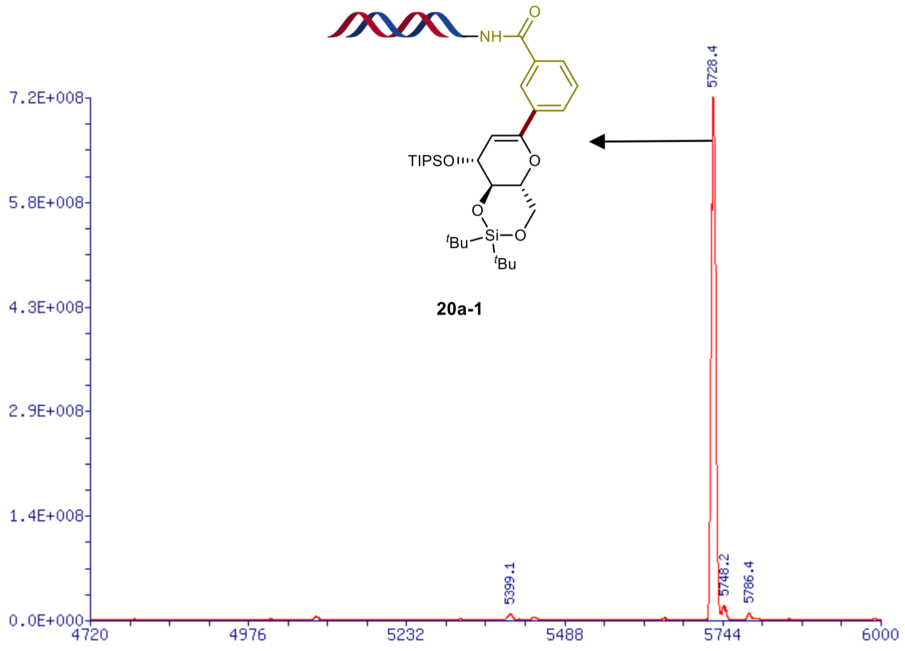

Supplementary Figure 6. Deconvoluted mass spectrum of **20a-1,** expected Mass: 5729.3; observed Mass: 5728.4

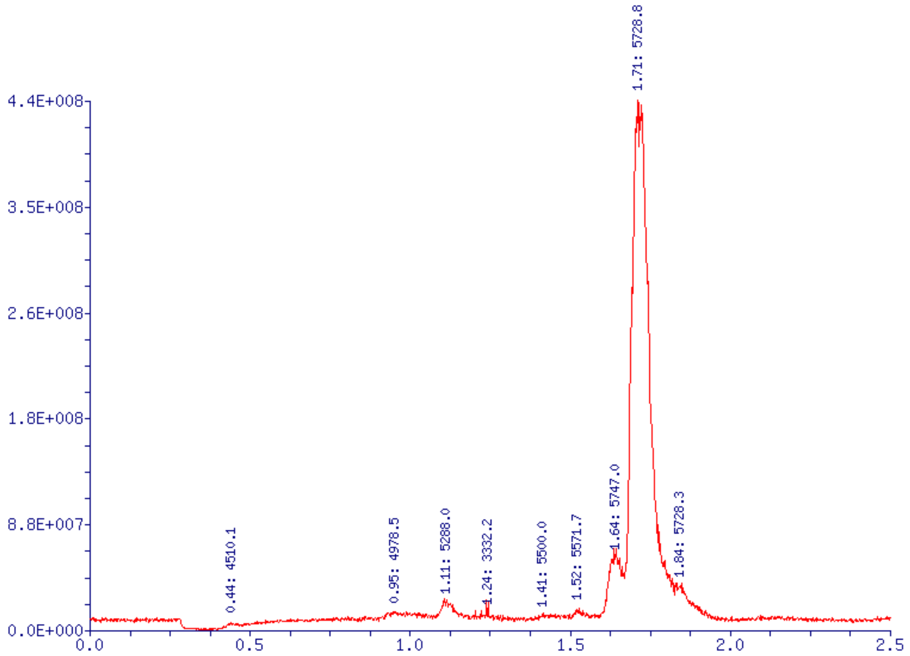

Supplementary Figure 7. LC-MS spectrum of **20a-2,** expected Mass: 5729.3; observed Mass: 5728.8

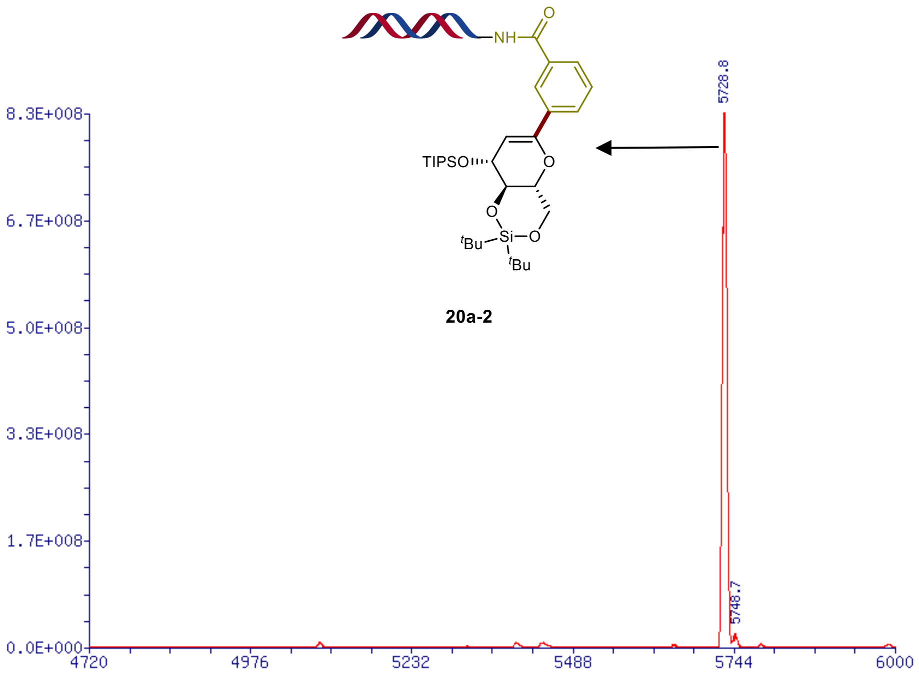

Supplementary Figure 8. Deconvoluted mass spectrum of **20a-2,** expected Mass: 5729.3; observed Mass: 5728.8

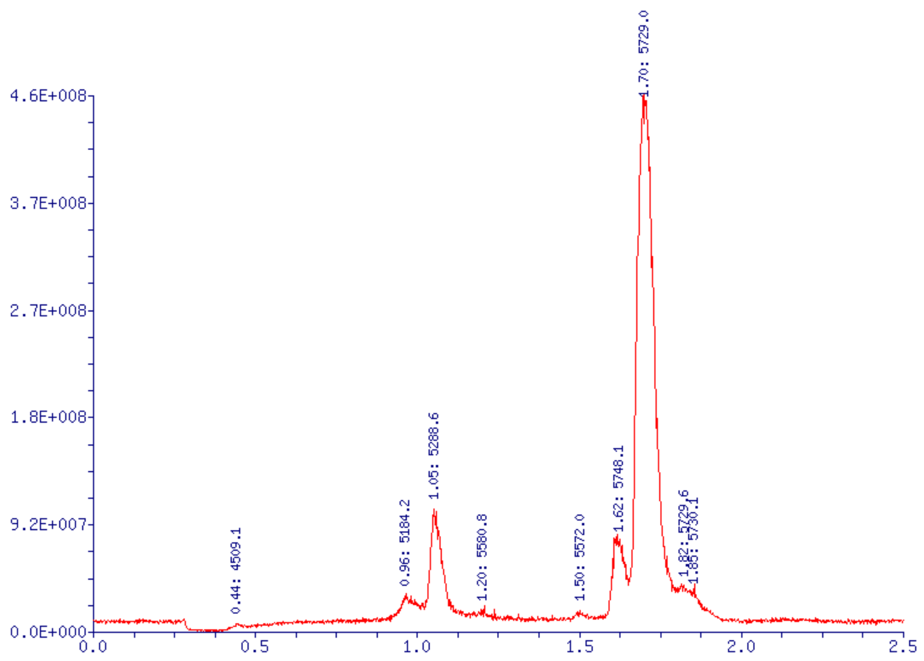

Supplementary Figure 9. LC-MS spectrum of **20b,** expected Mass: 5730.3; observed Mass: 5729.0

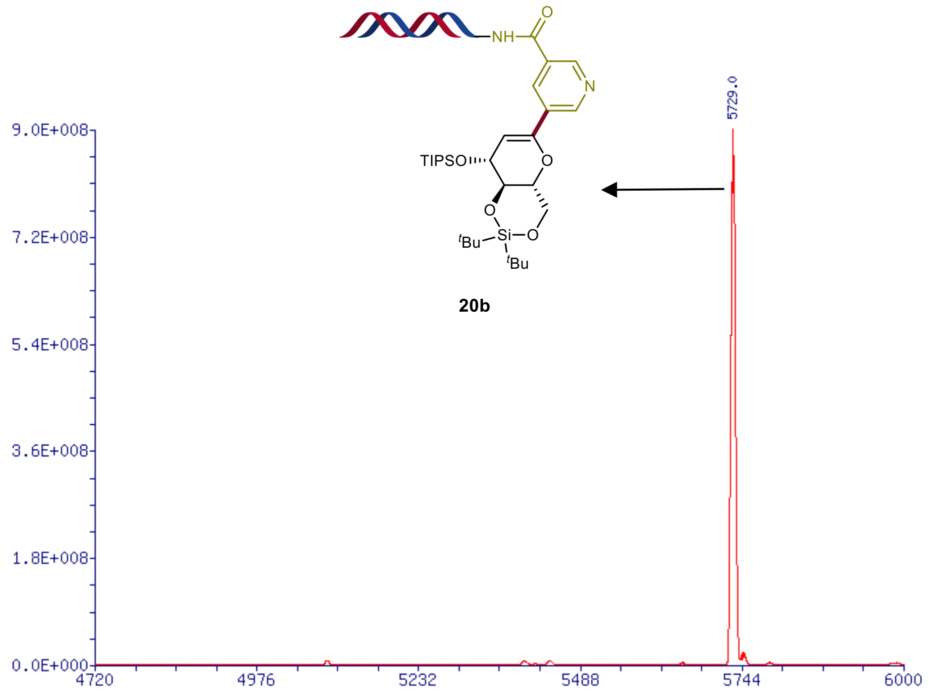

Supplementary Figure 10. Deconvoluted mass spectrum of **20b,** expected Mass: 5730.3; observed Mass: 5729.0

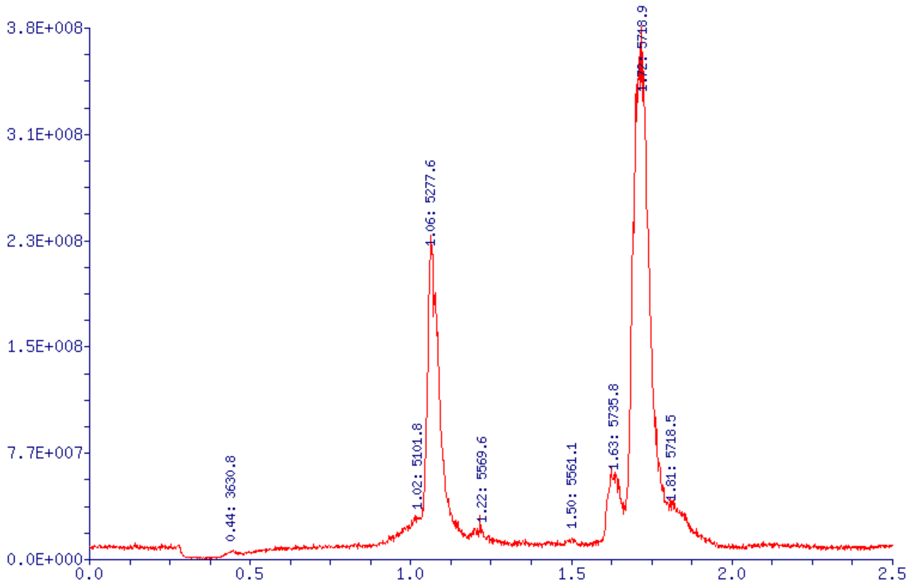

Supplementary Figure 11. LC-MS spectrum of **20c,** expected Mass: 5719.3; observed Mass: 5718.9

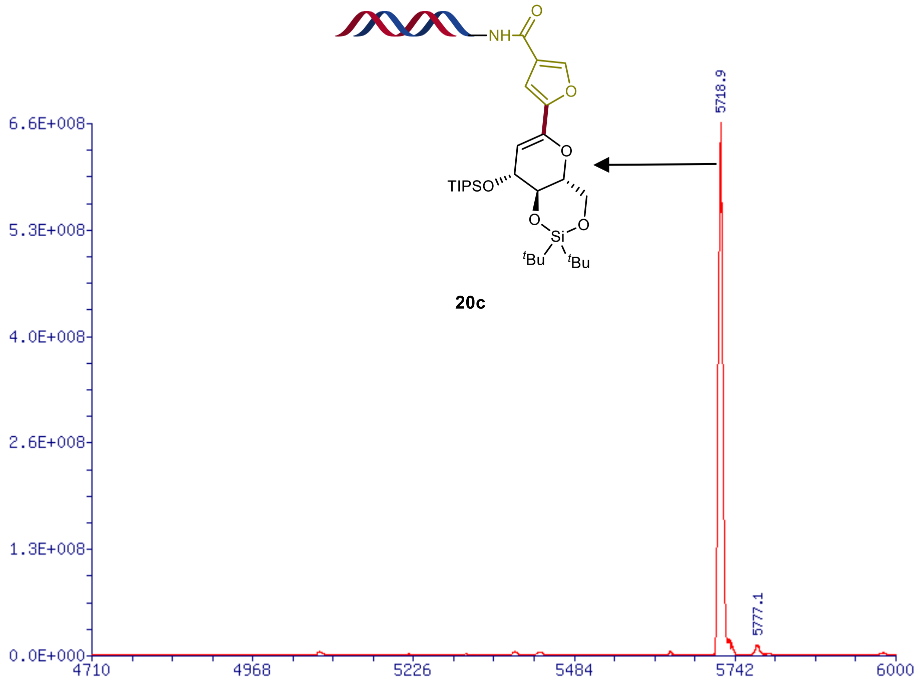

Supplementary Figure 12. Deconvoluted mass spectrum of **20c,** expected Mass: 5719.3; observed Mass: 5718.9

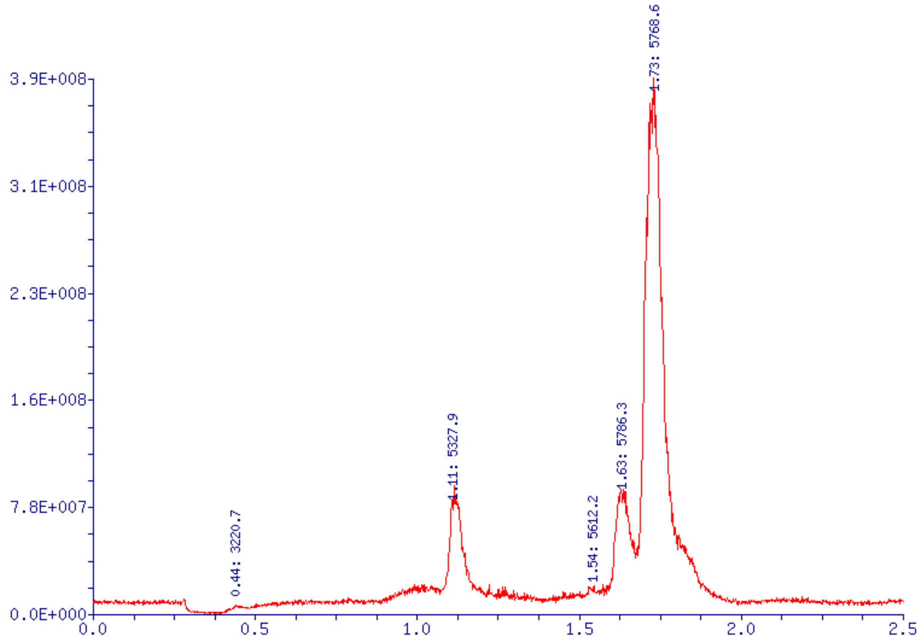

Supplementary Figure 13. LC-MS spectrum of **20d,** expected Mass: 5769.3; observed Mass: 5768.6

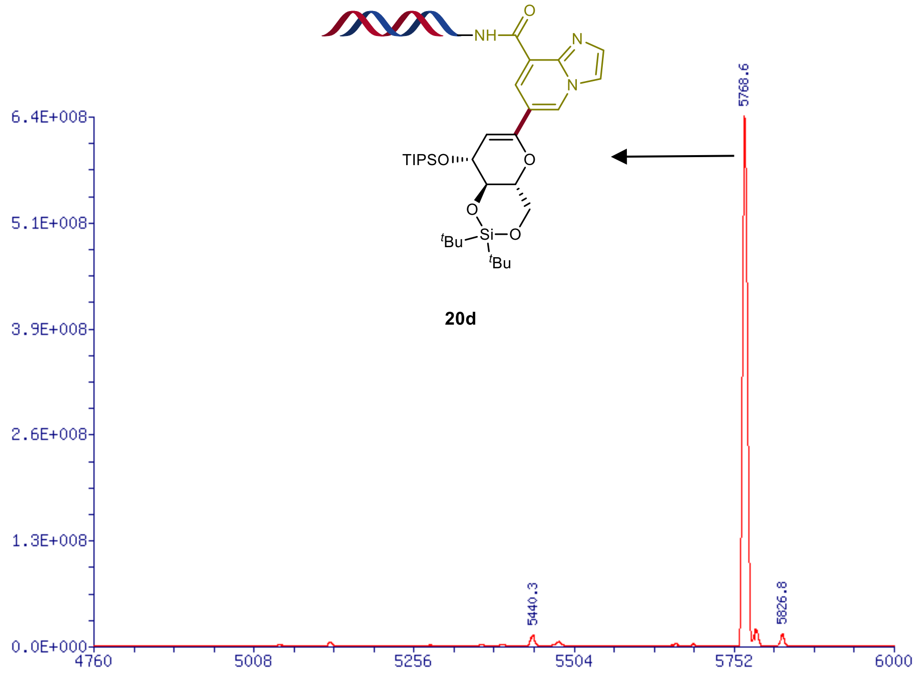

Supplementary Figure 14. Deconvoluted mass spectrum of **20d,** expected Mass: 5769.3; observed Mass: 5768.6

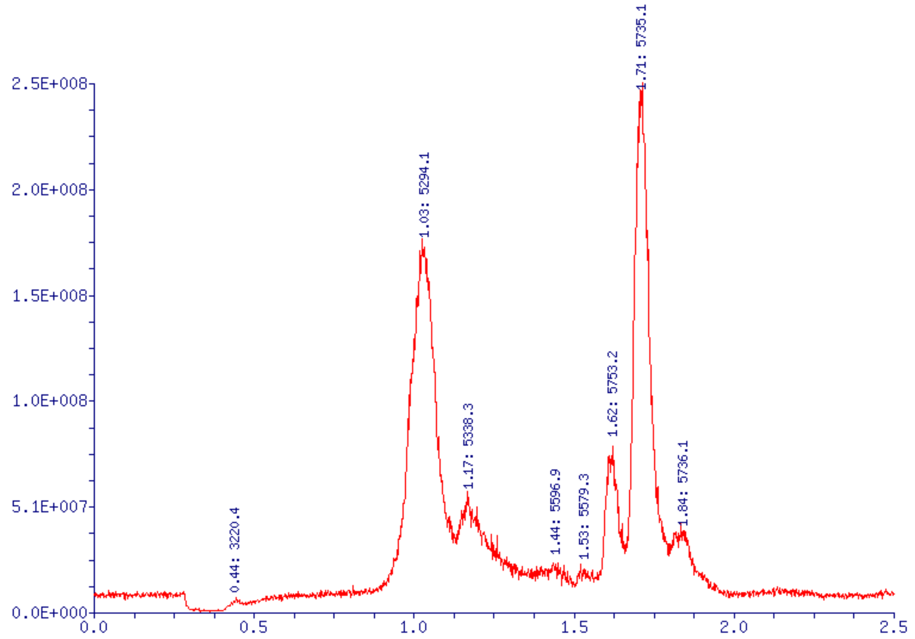

Supplementary Figure 15. LC-MS spectrum of **20e,** expected Mass: 5736.3; observed Mass: 5735.1

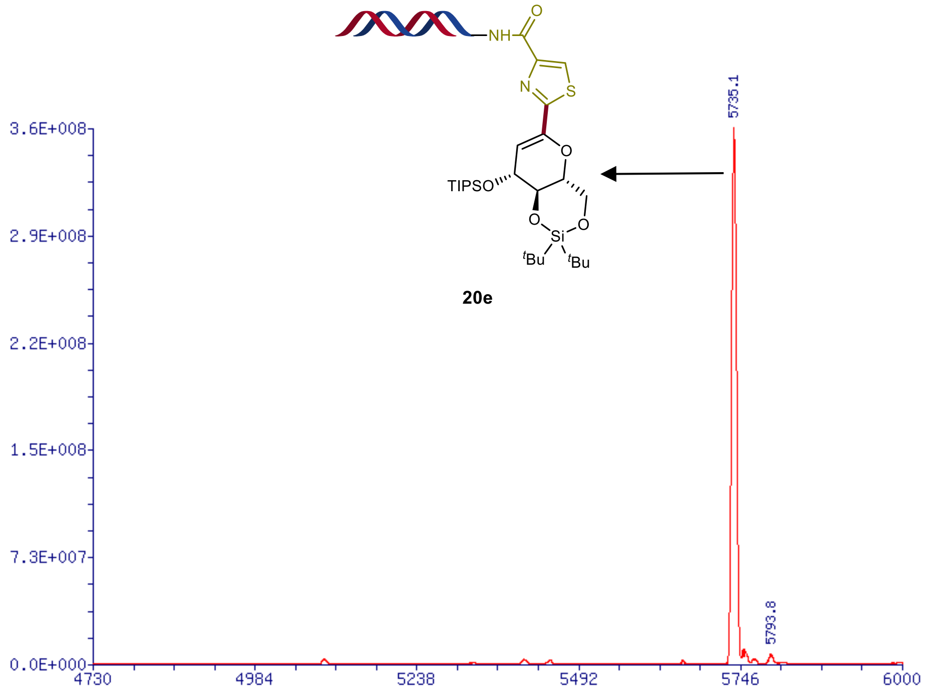

Supplementary Figure 16. Deconvoluted mass spectrum of **20e,** expected Mass: 5736.3; observed Mass: 5735.1

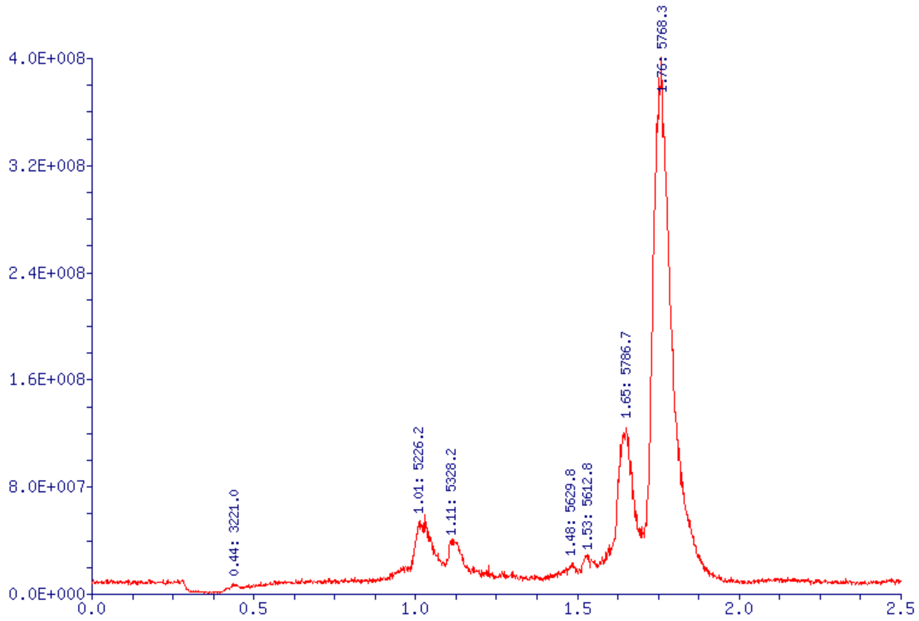

Supplementary Figure 17. LC-MS spectrum of **20f,** expected Mass: 5769.3; observed Mass: 5768.3

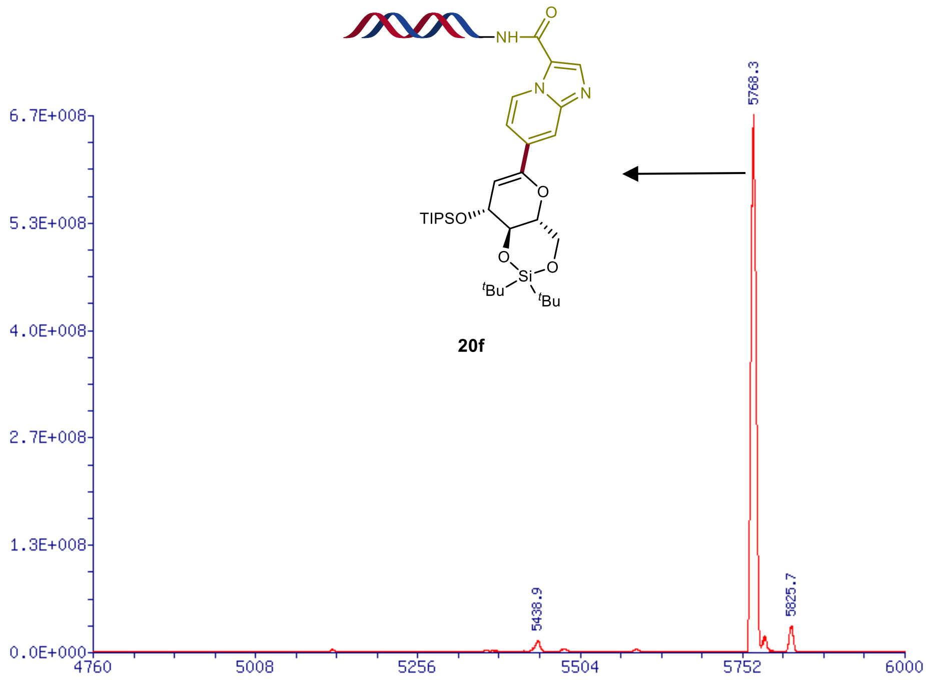

Supplementary Figure 18. Deconvoluted mass spectrum of **20f,** expected Mass: 5769.3; observed Mass: 5768.3

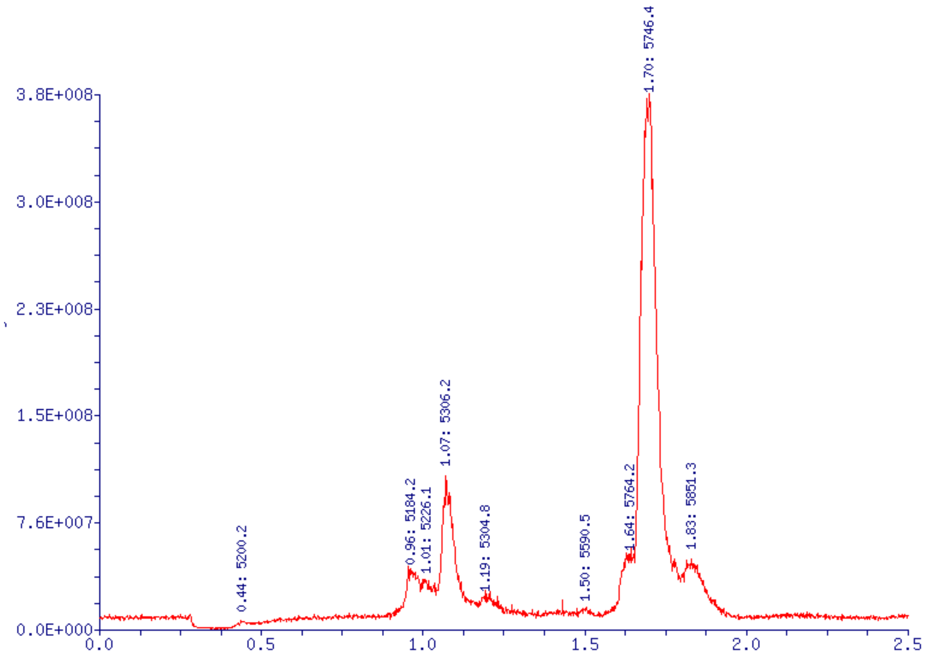

Supplementary Figure 19. LC-MS spectrum of **20g,** expected Mass: 5747.3; observed Mass: 5746.4

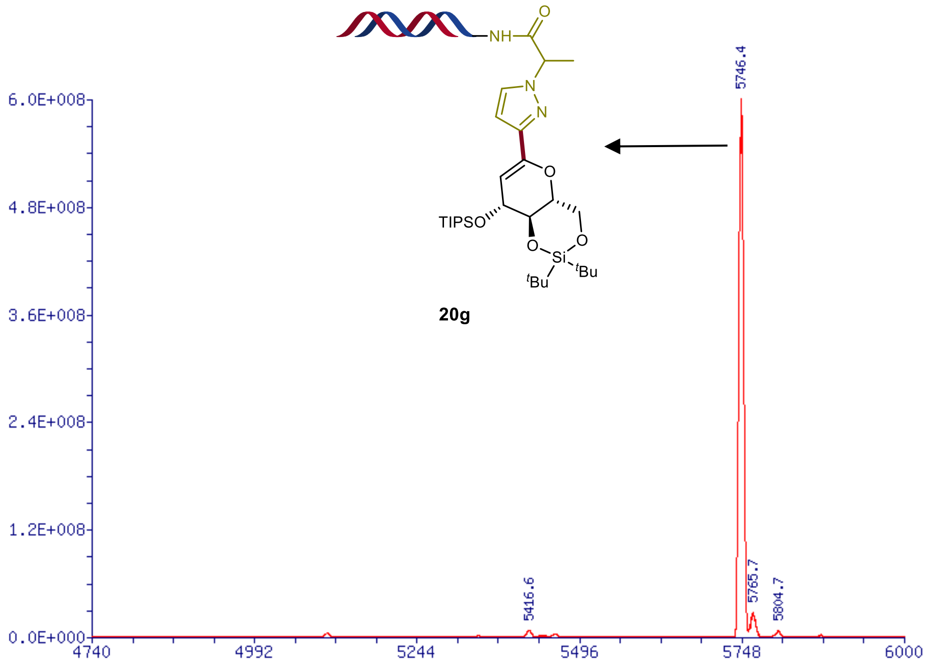

Supplementary Figure 20. Deconvoluted mass spectrum of **20g,** expected Mass: 5747.3; observed Mass: 5746.4

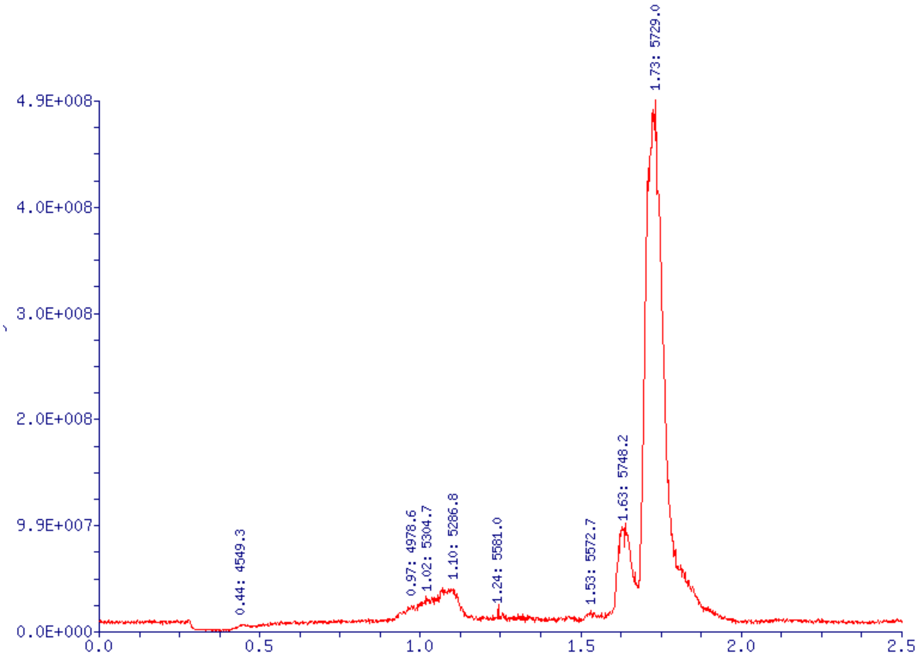

Supplementary Figure 21. LC-MS spectrum of **20h,** expected Mass: 5730.3; observed Mass: 5729.0

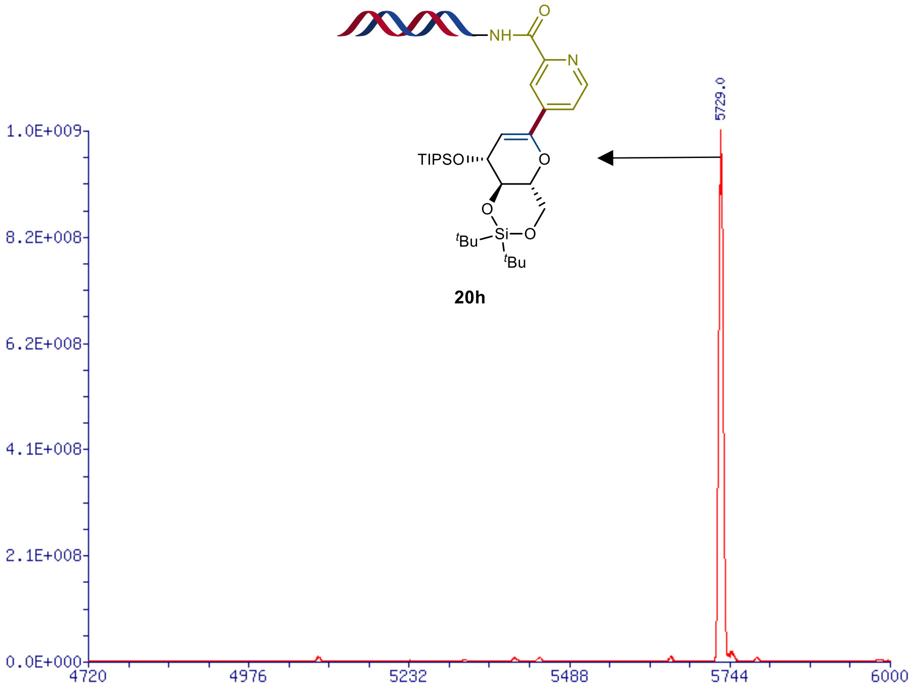

Supplementary Figure 22. Deconvoluted mass spectrum of **20h,** expected Mass: 5730.3; observed Mass: 5729.0

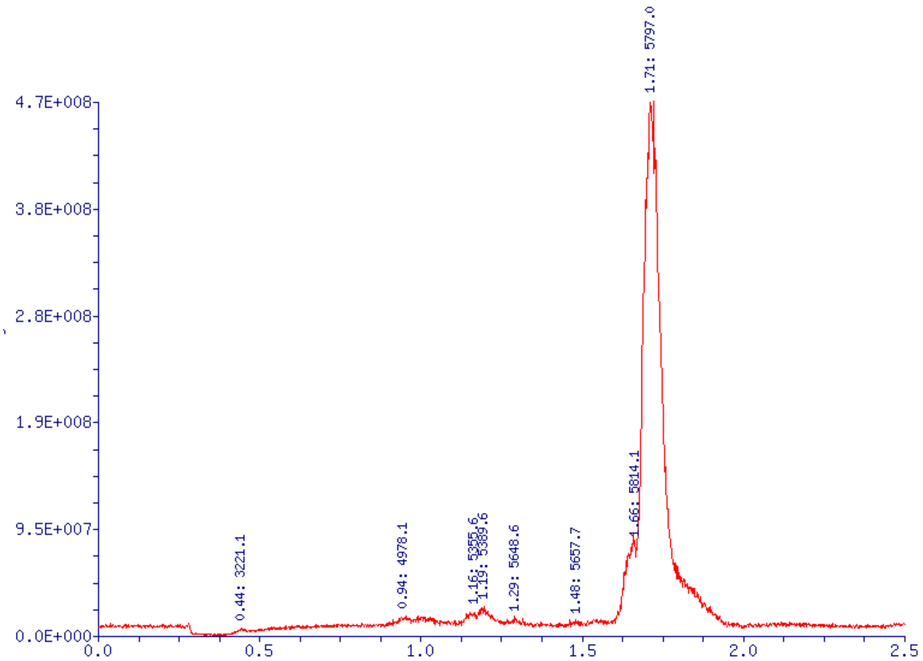

Supplementary Figure 23. LC-MS spectrum of **20i,** expected Mass: 5797.3; observed Mass: 5797.0

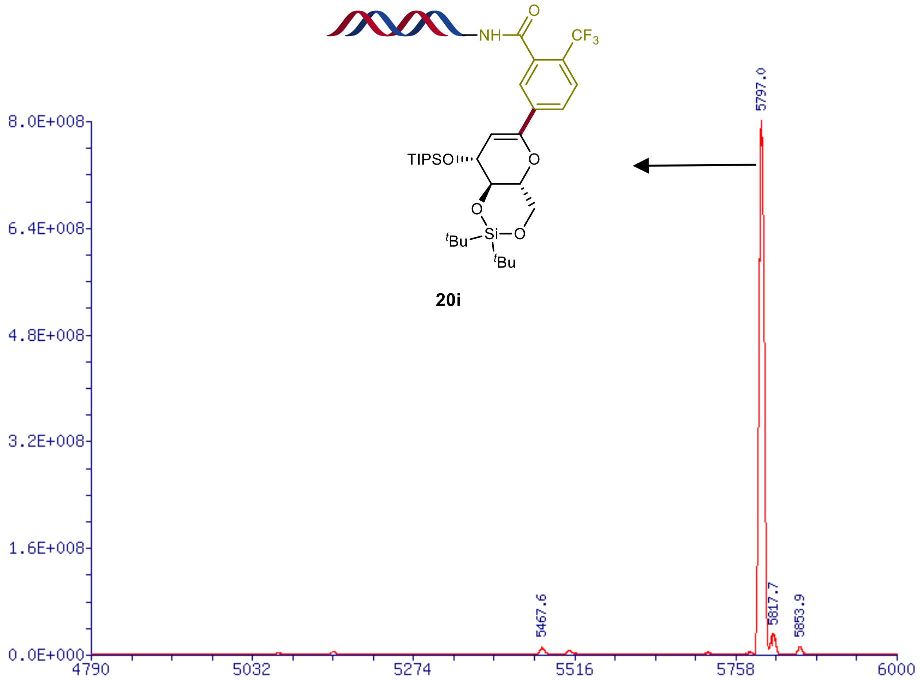

Supplementary Figure 24. Deconvoluted mass spectrum of **20i,** expected Mass: 5797.3; observed Mass: 5797.0

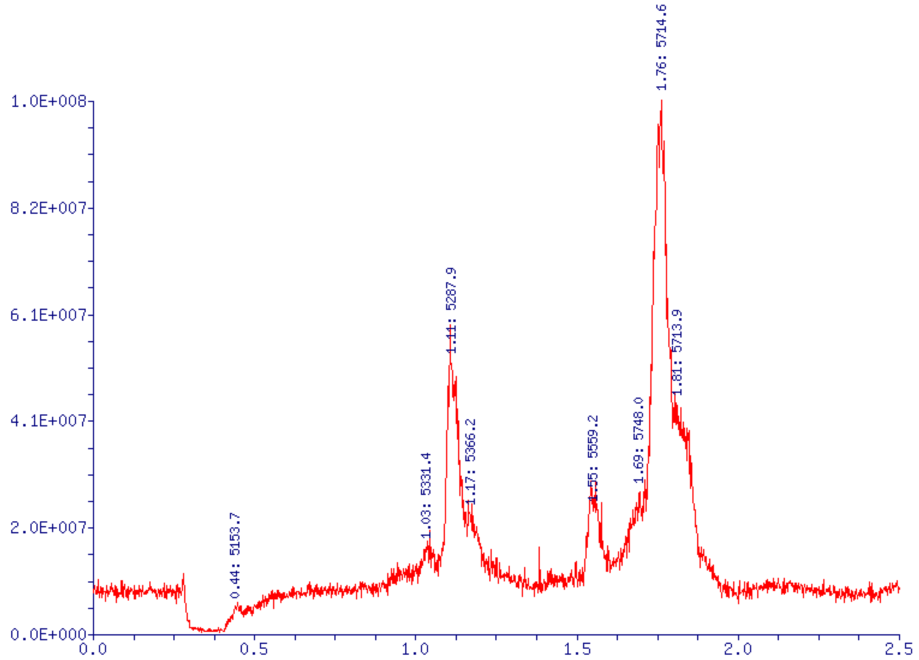

Supplementary Figure 25. LC-MS spectrum of **20j,** expected Mass: 5715.3; observed Mass: 5714.6

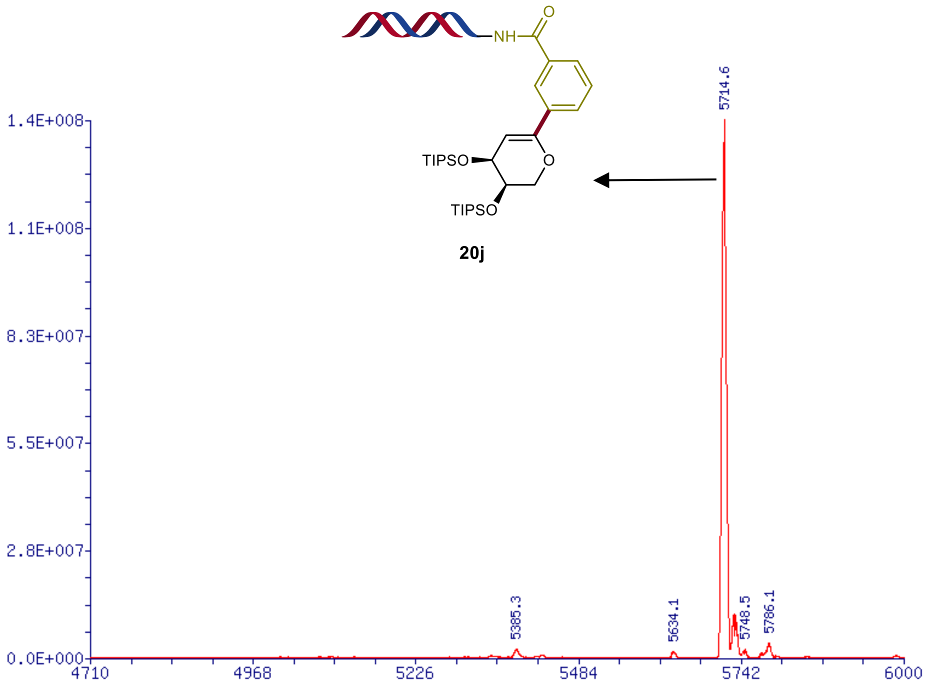

Supplementary Figure 26. Deconvoluted mass spectrum of **20j,** expected Mass: 5715.3; observed Mass: 5714.6

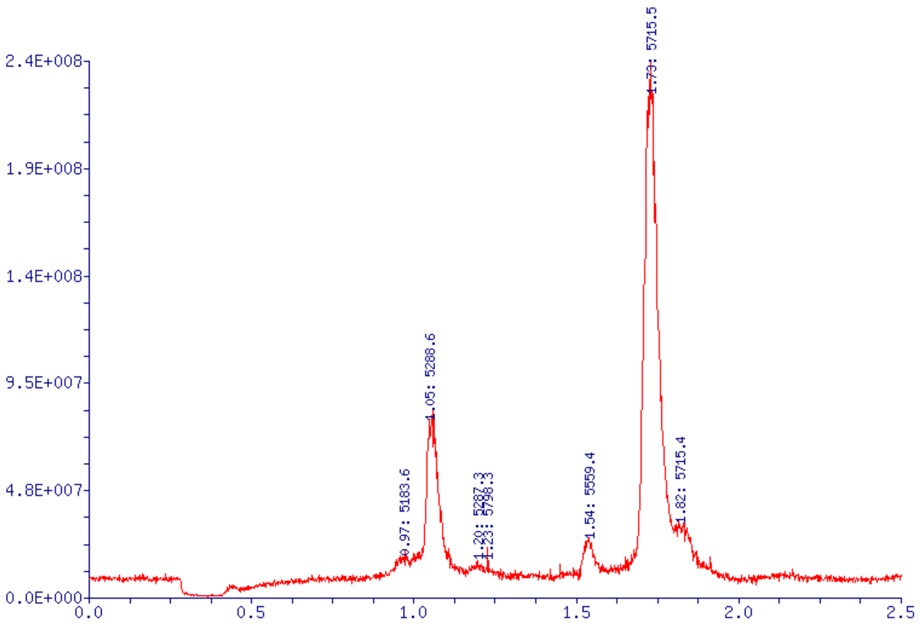

Supplementary Figure 27. LC-MS spectrum of **20k,** expected Mass: 5716.3; observed Mass: 5715.5

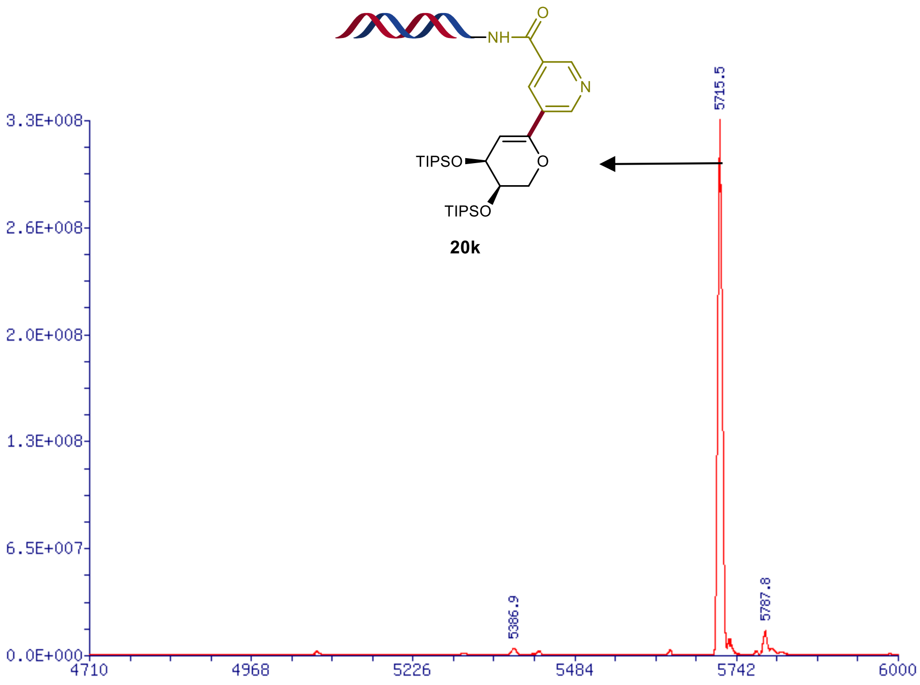

Supplementary Figure 28. Deconvoluted mass spectrum of **20k,** expected Mass: 5716.3; observed Mass: 5715.5

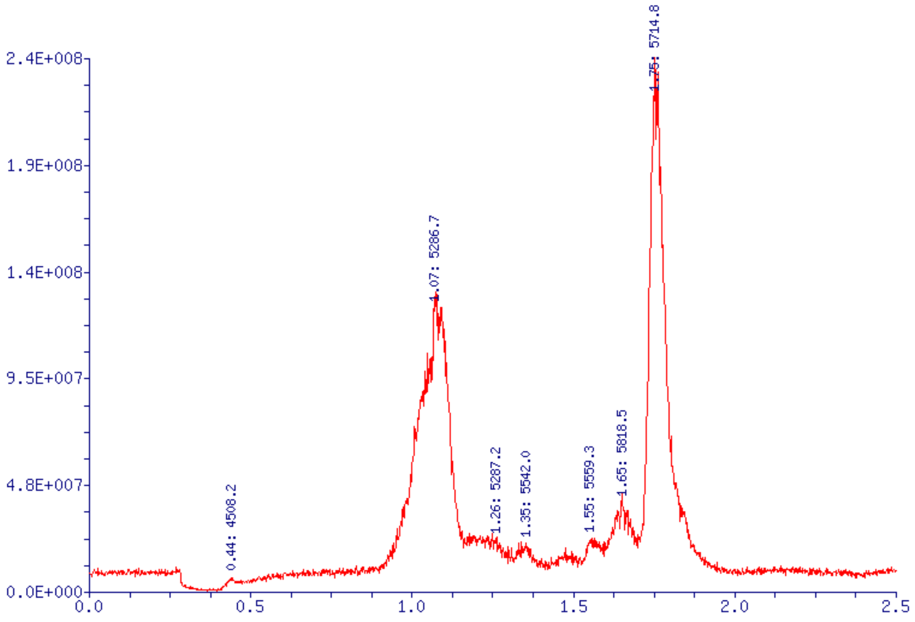

Supplementary Figure 29. LC-MS spectrum of **20l,** expected Mass: 5716.3; observed Mass: 5714.8

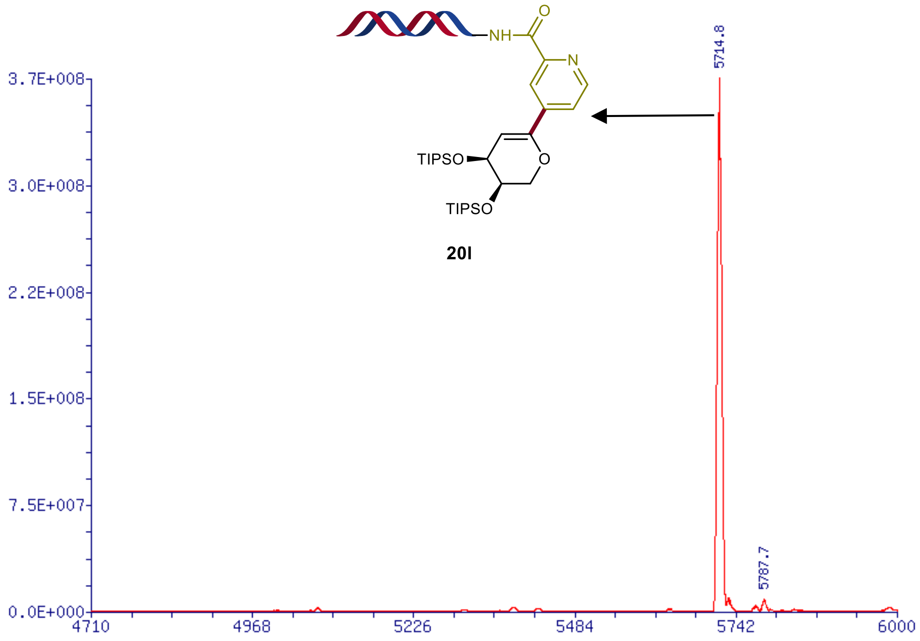

Supplementary Figure 30. Deconvoluted mass spectrum of **20l,** expected Mass: 5716.3; observed Mass: 5714.8

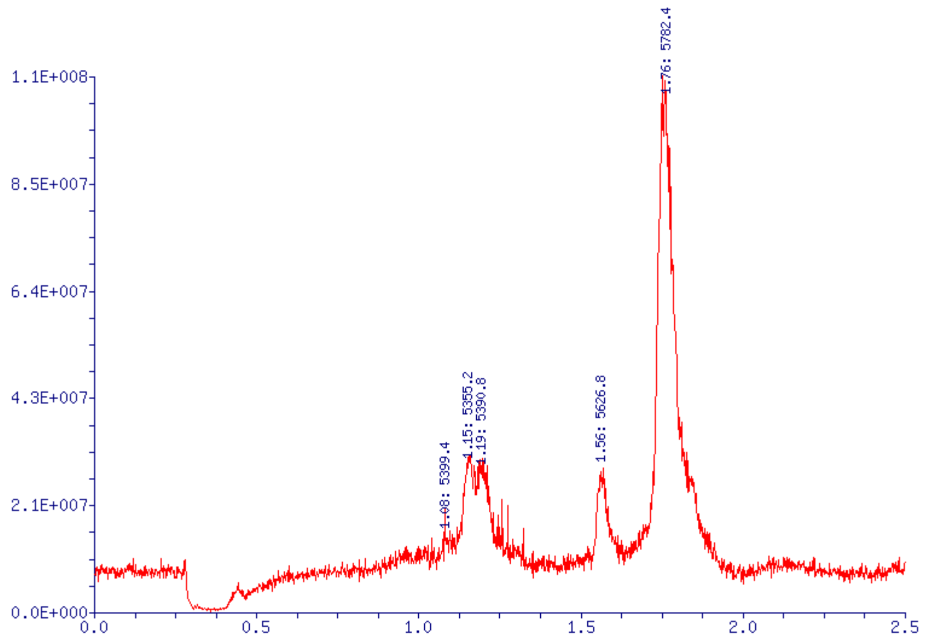

Supplementary Figure 31. LC-MS spectrum of **20m,** expected Mass: 5783.3; observed Mass: 5782.4

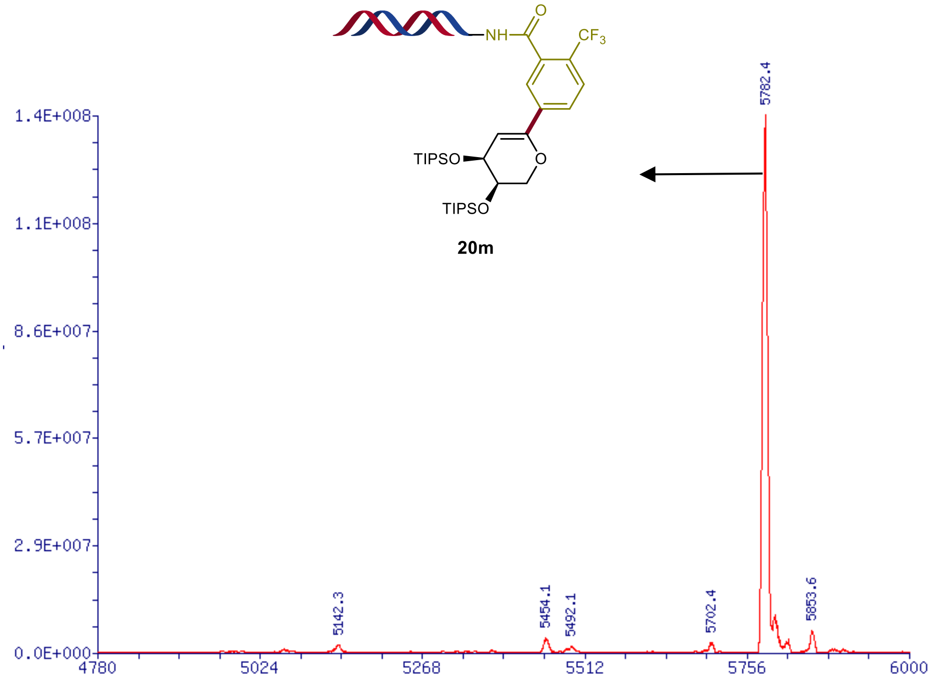

Supplementary Figure 32. Deconvoluted mass spectrum of **20m,** expected Mass: 5783.3; observed Mass: 5782.4

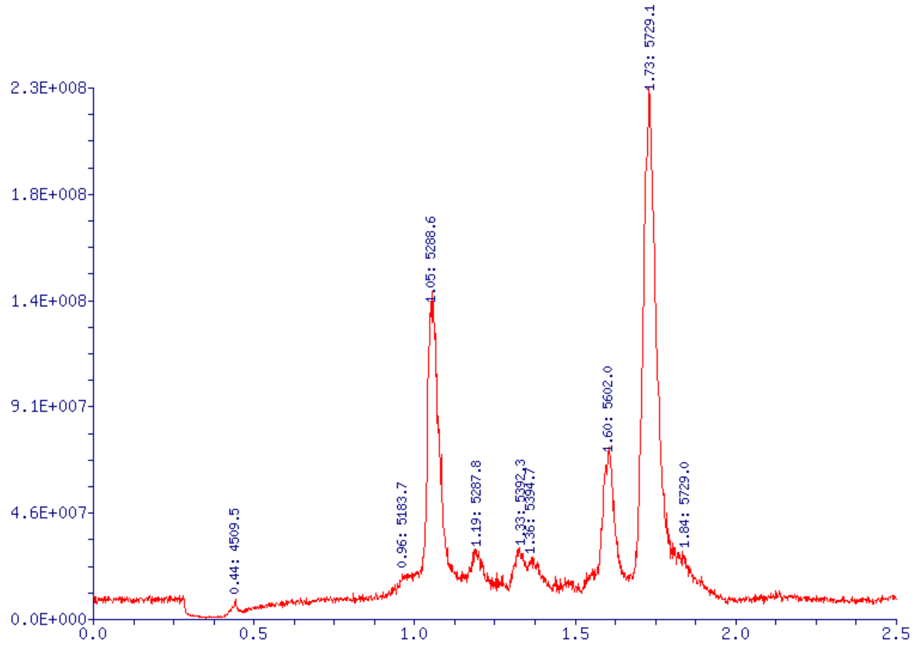

Supplementary Figure 33. LC-MS spectrum of **20n,** expected Mass: 5730.3; observed Mass: 5729.1

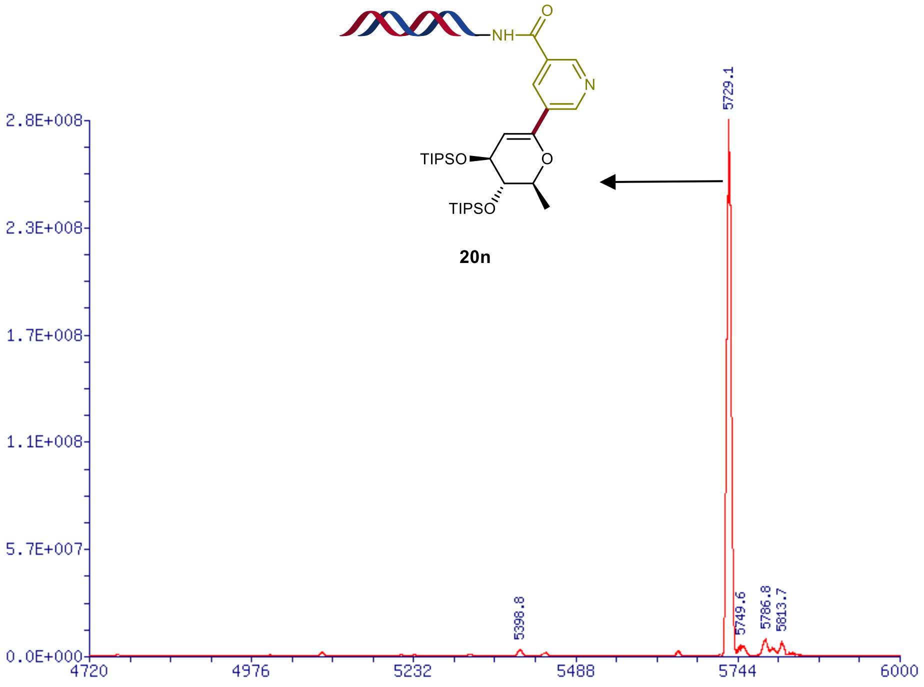

Supplementary Figure 34. Deconvoluted mass spectrum of **20n,** expected Mass: 5730.3; observed Mass: 5729.1

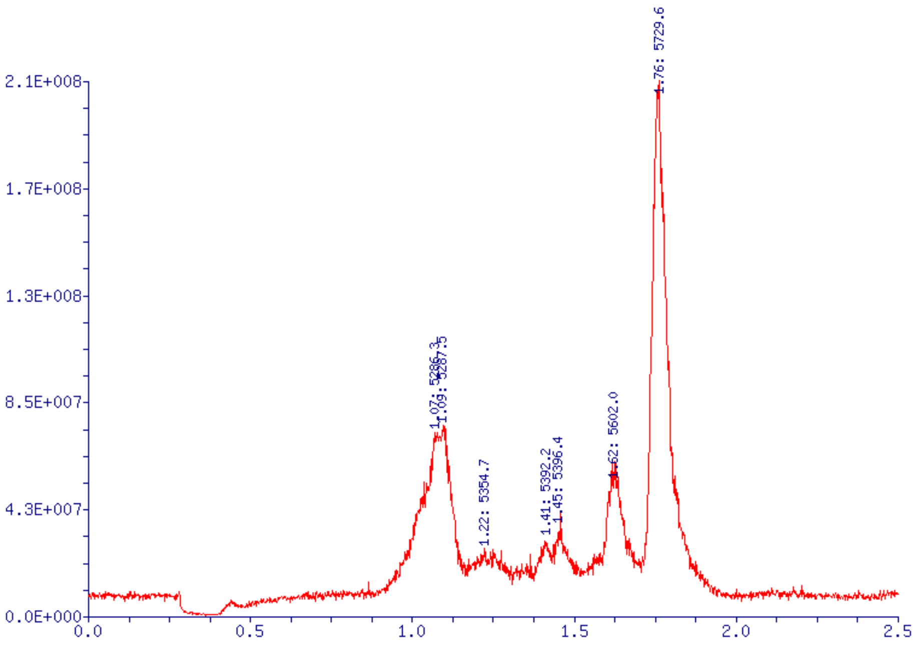

Supplementary Figure 35. LC-MS spectrum of **20o,** expected Mass: 5730.3; observed Mass: 5729.6

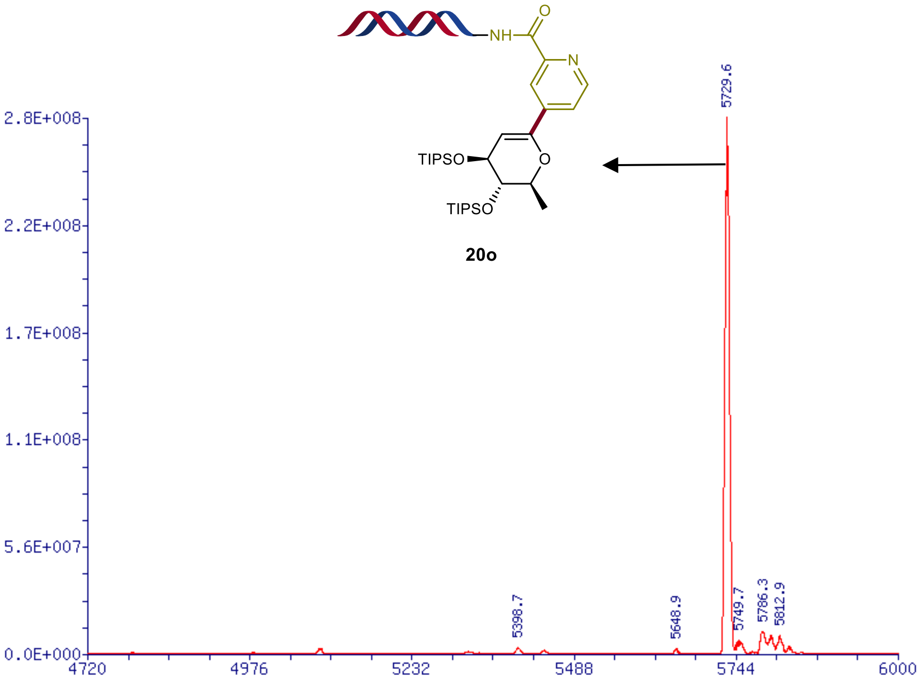

Supplementary Figure 36. Deconvoluted mass spectrum of **20o,** expected Mass: 5730.3; observed Mass: 5729.6

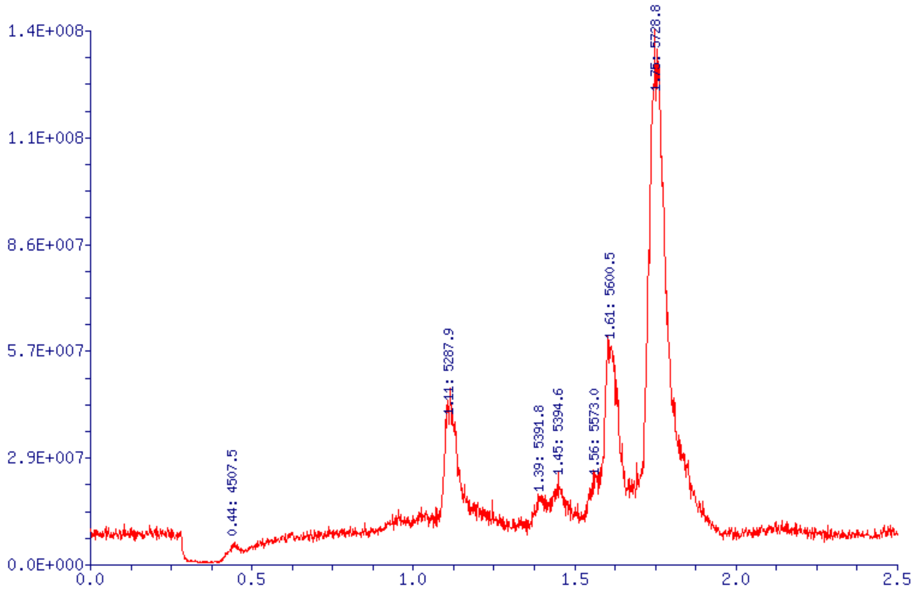

Supplementary Figure 37. LC-MS spectrum of **20p,** expected Mass: 5729.3; observed Mass: 5728.8

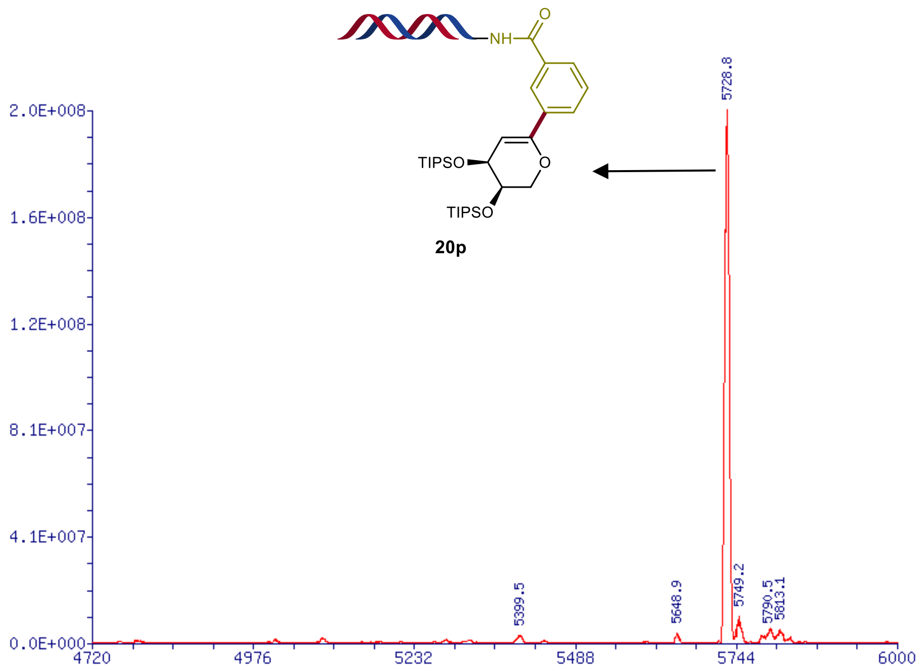

Supplementary Figure 38. Deconvoluted mass spectrum of **20p,** expected Mass: 5729.3; observed Mass: 5728.8

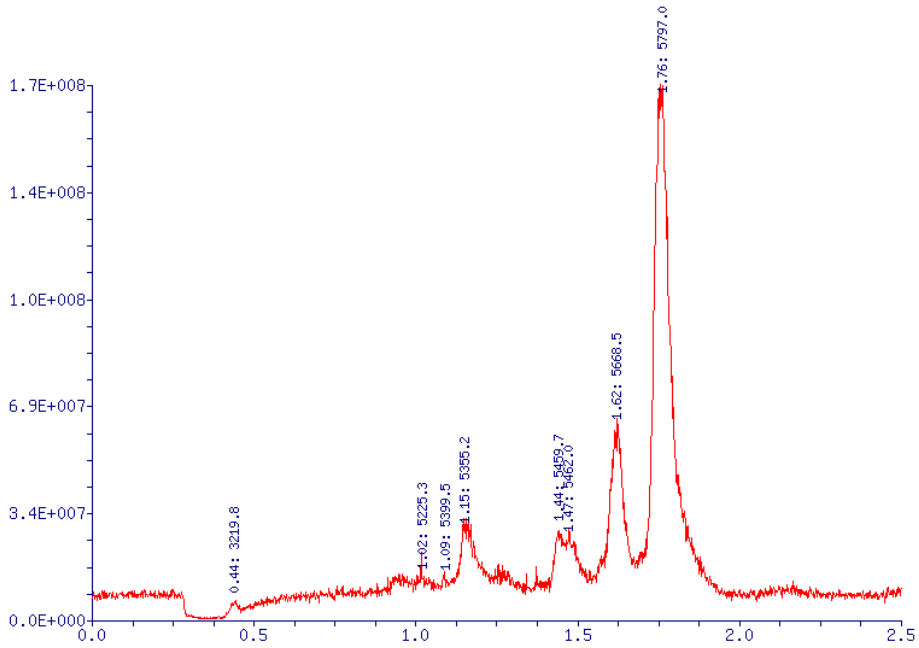

Supplementary Figure 39. LC-MS spectrum of **20q,** expected Mass: 5797.3; observed Mass: 5797.0

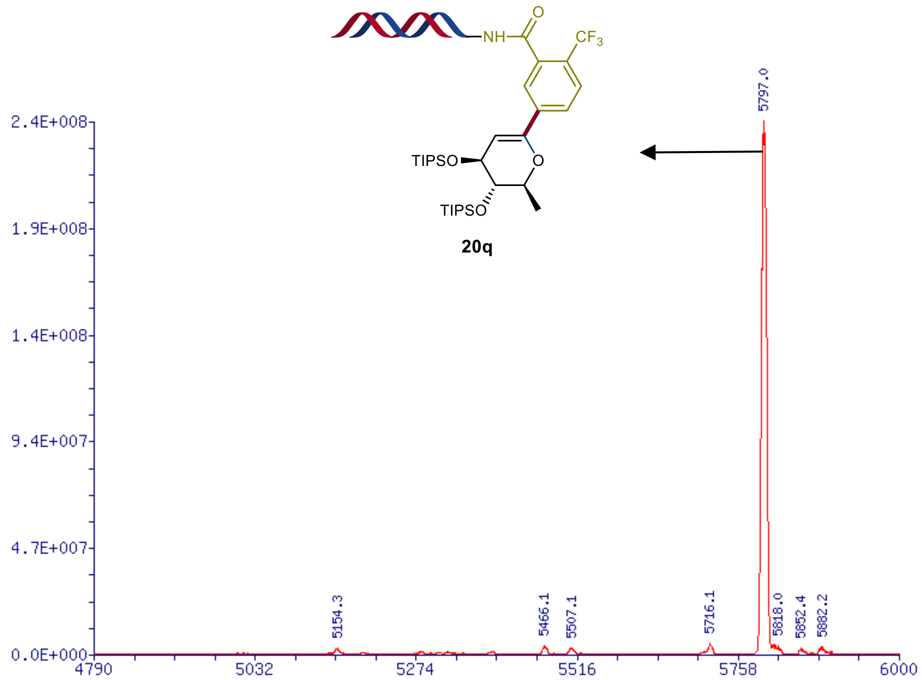

Supplementary Figure 40. Deconvoluted mass spectrum of **20q,** expected Mass: 5797.3; observed Mass: 5797.0

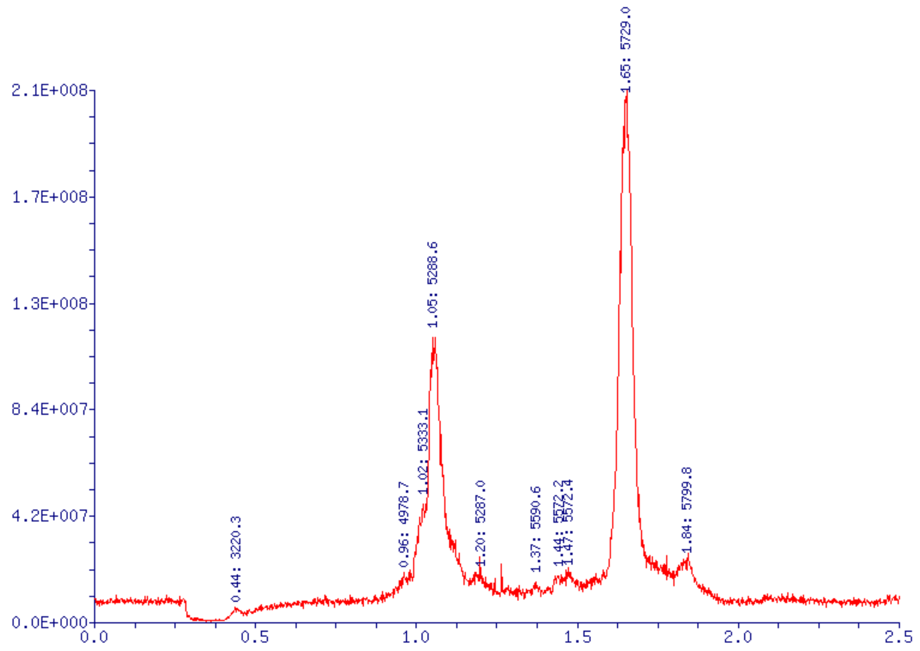

Supplementary Figure 41. LC-MS spectrum of **20r,** expected Mass: 5730.3; observed Mass: 5729.0

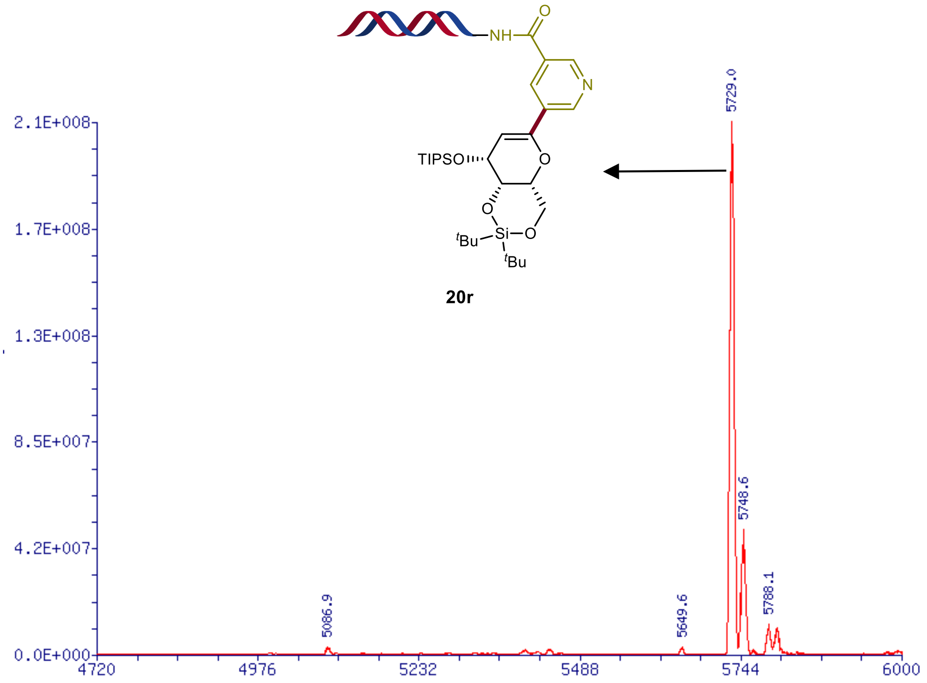

Supplementary Figure 42. Deconvoluted mass spectrum of **20r,** expected Mass: 5730.3; observed Mass: 5729.0

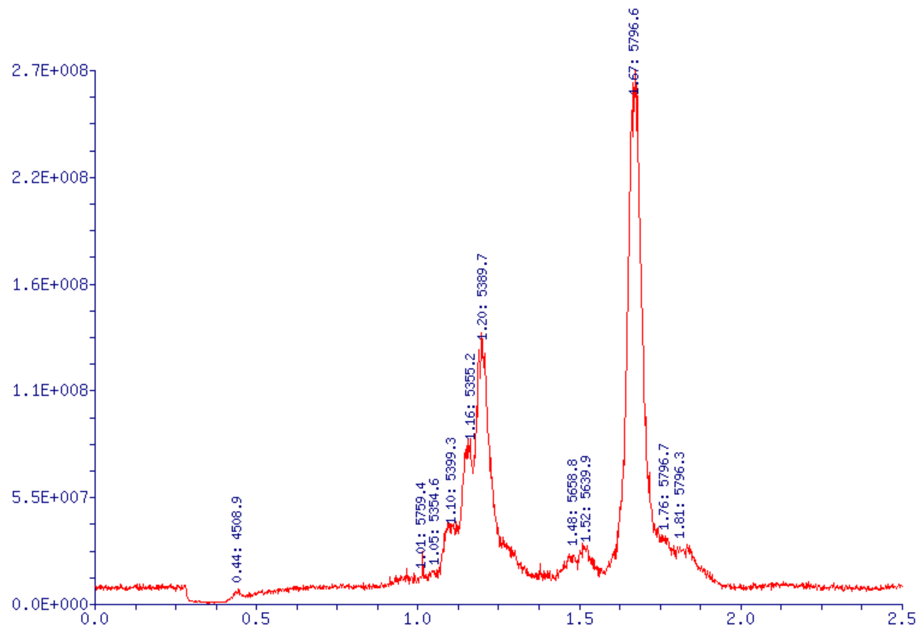

Supplementary Figure 43. LC-MS spectrum of **20s,** expected Mass: 5797.3; observed Mass: 5796.6

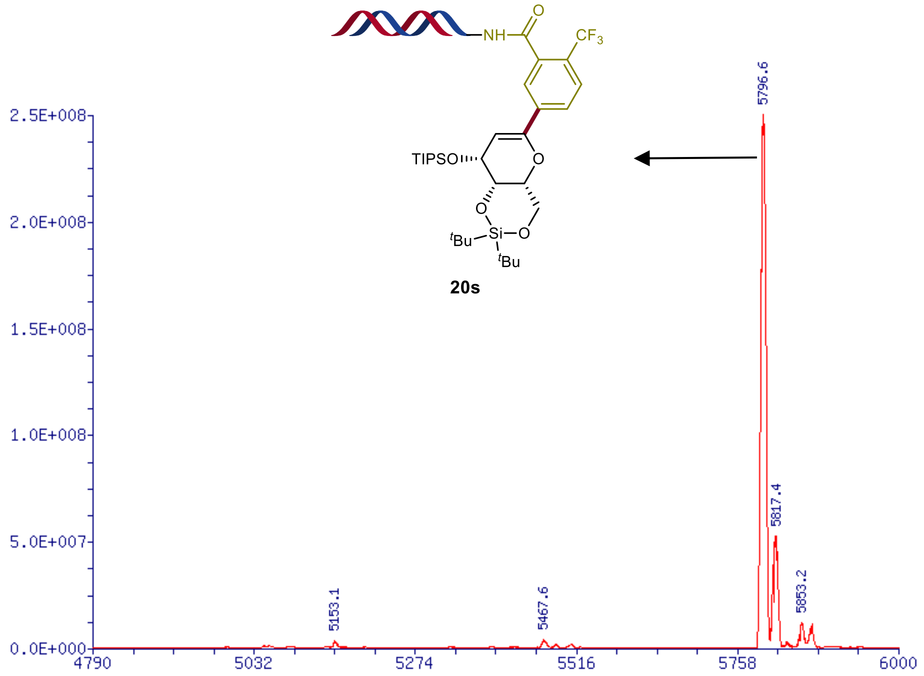

Supplementary Figure 44. Deconvoluted mass spectrum of **20s,** expected Mass: 5797.3; observed Mass: 5796.6

2.6. Detailed experimental procedures for late-stage glycodiversifications and total synthesis

Synthesis of Dapagliflozin

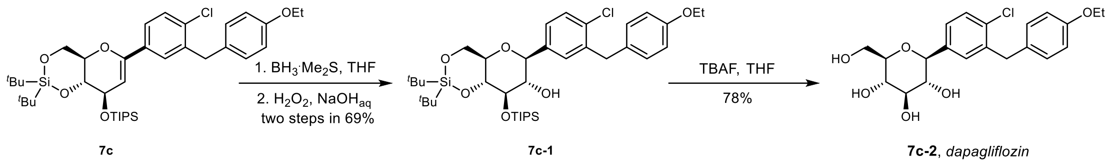

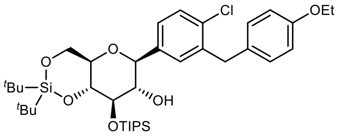

7c-1

(4a*R*,6*S*,7*S*,8*R*,8a*R*)-2,2-Di-*tert*-butyl-6-(4-chloro-3-(4-ethoxybenzyl)phenyl)-8-((triisopropylsilyl)oxy)hexahydropyrano[3,2-*d*][1,3,2]dioxasilin-7-ol (7c-1). *C*1-Aryl glucal **7c** (290 mg, 0.40 mmol, 1.00 equiv) was dissolved in THF (20.0 mL), and $BH_3 \cdot Me_2S$ (0.80 mL, 8.00 mmol, 20.0 equiv, 10.0 M in Me_2S) was added dropwise at 0 °C. The reaction mixture was stirred at room temperature for 24 h. Then, 30% NaOH/ 30% H_2O_2 (1:1, 8.00 mL) was added, and the reaction mixture was stirred at rt for an additional 24 h. The mixture was diluted with Et_2O (60.0 mL), and the organic layer was partitioned and washed successively saturated NH_4Cl solution (40.0 mL), H_2O (40.0 mL), brine (40.0 mL), dried over Na_2SO_4, and concentrated under reduced pressure. Column chromatography of the residue on SiO_2 (Petroleum ether:EtOAc, 1:0-20:1) to afford **7c-1** (195 mg, 69%) as a white foam: **^{1}H NMR** (500 MHz, $CDCl_3$) δ 7.38 (d, *J* = 8.1 Hz, 1H), 7.21 (d, *J* = 9.6 Hz, 2H), 7.10 (d, *J* = 8.2 Hz, 2H), 6.83 (d, *J* = 8.1 Hz, 2H), 4.20 – 4.16 (m, 2H), 4.09 (d, *J* = 15.3 Hz, 1H), 4.03 – 3.98 (m, 3H), 3.92 – 3.81 (m, 3H), 3.56 – 3.52 (m, 1H), 3.45 (t, *J* = 8.8, 8.8 Hz, 1H), 2.03 (s, 1H), 1.41 (t, *J* = 6.9, 6.9 Hz, 3H), 1.28 – 1.20 (m, 3H), 1.15 – 1.12 (m, 18H), 1.09 (s, 9H), 1.03 (s, 9H); **^{13}C NMR** (126 MHz, $CDCl_3$) δ 157.5, 139.2, 137.5, 131.3, 130.4, 130.0, 129.8, 126.4, 114.6, 81.5, 79.9, 78.1, 76.6, 75.4, 66.7, 63.5, 38.5, 27.6, 27.1, 22.9, 20.1, 18.6, 18.5, 15.0, 13.1; **HRMS** (ESI) m/z calcd for $C_{38}H_{61}O_6Si_2ClNa$ $[M + Na]^+$ 727.3587, found 727.3595.

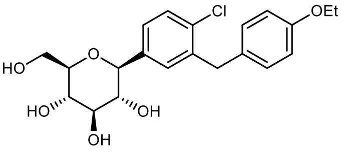

7c-2

(2*S*,3*R*,4*R*,5*S*,6*R*)-2-(4-Chloro-3-(4-ethoxybenzyl)phenyl)-6-(hydroxymethyl)tetrahydro-2*H*-pyran-3,4,5-triol (7c-2). To a solution of compound

7c-1 (141 mg, 0.20 mmol, 1.00 equiv) in THF (8.00 mL), a solution of TBAF in THF (1.00 M, 0.80 mL) was added at 0 °C, and this reaction mixture was stirred at 45 °C for 5 h. The resulting mixture was concentrated under reduced pressure, and column chromatography of the residue on silica gel (DCM/MeOH, 1:0-10:1) to afford **7c-2,** including some TBAF. Subsequent conventional formylation, column chromatography purification, and deformylation using a catalytic amount of sodium methoxide afford pure **7c-2** (63.3 mg, 78%) as a white solid: ^{1}**H NMR** (500 MHz, Methanol-d_4) δ 7.31 – 7.28 (m, 2H), 7.24 (dd, *J* = 8.2, 2.2 Hz, 1H), 7.06 – 7.04 (m, 2H), 6.76 – 6.74 (m, 2H), 4.06 – 3.91 (m, 5H), 3.83 (dd, *J* = 11.9, 2.0 Hz, 1H), 3.66 (dd, *J* = 12.0, 5.3 Hz, 1H), 3.44 – 3.32 (m, 3H), 3.28 – 3.26 (m, 1H), 1.31 (t, *J* = 7.0, 7.0 Hz, 3H); ^{13}**C NMR** (126 MHz, Methanol-d4) δ 158.8, 140.0, 139.9, 134.4, 132.8, 131.9, 130.8, 130.1, 128.2, 115.4, 82.8, 82.1, 79.7, 76.4, 71.8, 64.4, 63.0, 39.2, 15.2; Characterization data matched the literature report.[23]

Glycodiversification of drugs

Synthesis of glucosyl triclosan

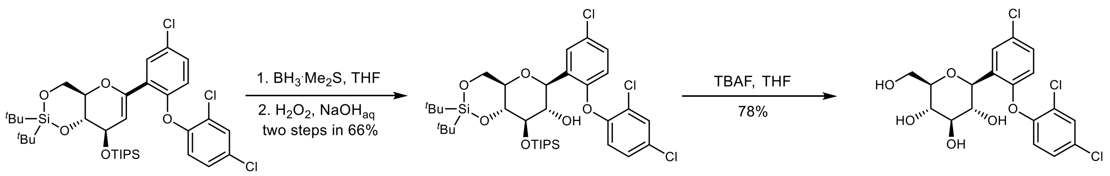

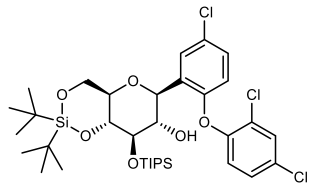

7d-1

(4a*R*,6*S*,7*S*,8*R*,8a*R*)-2,2-Di-*tert*-butyl-6-(5-chloro-2-(2,4-dichlorophenoxy)phenyl)-8-((triisopropylsilyl)oxy)hexahydropyrano[3,2-*d*][1,3,2]dioxasilin-7-ol (7d-1). *C*-Aryl glucal **7d** (285 mg, 0.40 mmol, 1.00 equiv) was dissolved in THF (20.0 mL), and BH_3•Me_2S (0.80 mL, 8.00 mmol, 20.0 equiv, 10.0 M in Me_2S) was added dropwise at 0 °C. The reaction mixture was stirred at room temperature for 24 h. Then, 30% NaOH/ 30% H_2O_2 (1:1, 8.00 mL) was added, and the reaction mixture was stirred at rt for an additional 24 h. The mixture was diluted with Et_2O (60.0 mL), and the organic layer was partitioned and washed successively saturated NH_4Cl solution (40.0 mL), H_2O (40.0 mL), brine (40.0 mL), dried over Na_2SO_4, and concentrated under reduced pressure. Column chromatography of the residue on SiO_2 (Petroleum ether:EtOAc, 1:0-20:1) to afford **7c-1** (192 mg, 66%) as a colorless oil: ^{1}**H NMR** (500 MHz, $CDCl_3$) δ 7.47 (dd, *J* = 4.7, 2.6 Hz, 2H), 7.22 (dd, *J* = 8.7, 2.6 Hz, 1H), 7.17 (dd, *J* = 8.8, 2.5 Hz, 1H), 6.89 (d, *J* = 8.8 Hz, 1H), 6.70 (d, *J* = 8.7 Hz, 1H), 4.71 (d, *J* = 9.7 Hz, 1H), 4.06 (dd, *J* = 10.2, 4.9 Hz, 1H), 3.87 – 3.80 (m, 2H), 3.75 (t, *J* = 10.2, 10.2 Hz, 1H), 3.67 (t, *J* = 8.7, 8.7 Hz, 1H), 3.53 – 3.48 (m, 1H),

2.11 (s, 1H), 1.26 – 1.20 (m, 3H), 1.13 – 1.11 (m, 18H), 1.07 (s, 9H), 1.00 (s, 9H); **^{13}C NMR** (126 MHz, $CDCl_3$) δ 153.1, 151.8, 131.5, 130.5, 129.9, 129.6, 129.4, 129.0, 128.2, 126.2, 121.1, 119.5, 80.3, 78.1, 76.2, 75.5(2), 66.5, 27.6, 27.1, 22.9, 20.1, 18.6, 18.5, 13.2; **HRMS** (ESI) m/z calcd for $C_{35}H_{53}Cl_3O_6Si_2Na$ [M + Na]$^+$ 753.2338, found 753.2330.

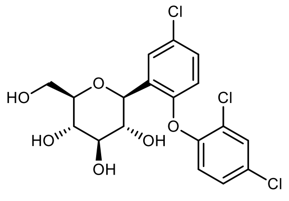

7d-2

(2*S*,3*R*,4*R*,5*S*,6*R*)-2-(5-Chloro-2-(2,4-dichlorophenoxy)phenyl)-6-(hydroxymethyl)tetrahydro-2*H*-pyran-3,4,5-triol (7d-2). To a solution of compound **7d-1** (132 mg, 0.18 mmol, 1.00 equiv) in THF (8.00 mL), a solution of TBAF in THF (1.00 M, 0.72 mL) was added at 0 °C, and this reaction mixture was stirred at 45 °C for 5 h. The resulting mixture was concentrated under reduced pressure, and column chromatography of the residue on SiO_2 (DCM/MeOH 1:0–10:1) to afford **7d-2** included some TBAF. Subsequent conventional formylation, column chromatography purification, and deformylation using catalytic amount of sodium methoxide afforded pure **7d-2** (61.3 mg, 78%) as a white foam: **^{1}H NMR** (500 MHz, Methanol-d_4) δ 7.61 (d, J = 2.6 Hz, 1H), 7.54 (d, J = 2.5 Hz, 1H), 7.29 – 7.24 (m, 2H), 6.99 (d, J = 8.8 Hz, 1H), 6.76 (d, J = 8.7 Hz, 1H), 4.63 (d, J = 9.5 Hz, 1H), 3.79 (dd, J = 12.0, 2.2 Hz, 1H), 3.62 – 3.54 (m, 2H), 3.47 (t, J = 8.7, 8.7 Hz, 1H), 3.42 (t, J = 9.1, 9.1 Hz, 1H), 3.35 – 3.32 (m, 1H); **^{13}C NMR** (126 MHz, Methanol-d_4) δ 154.7, 153.4, 133.9, 131.1, 130.5, 130.3(2), 130.1, 129.4, 127.0, 122.4, 120.7, 82.4, 79.7, 76.8, 75.8, 71.8, 63.0; **HRMS** (ESI) m/z calcd for $C_{18}H_{17}Cl_3O_6Na$ [M + Na]$^+$ 456.9983, found 456.9987.

Synthesis of 2-Deoxy-glucosyl 17*β*-estradiol

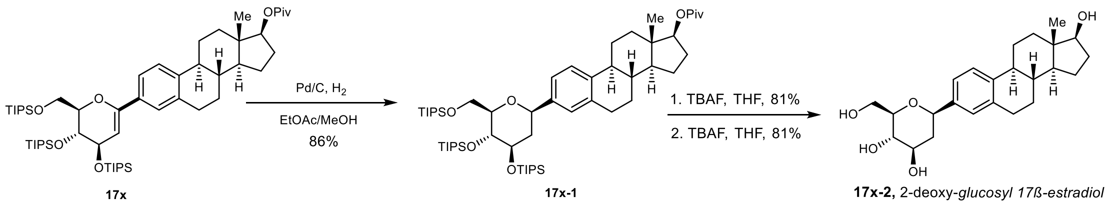

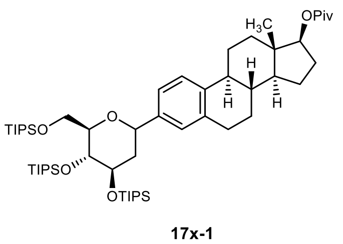

17x-1

**(8*R*,9*S*,13*S*,14*S*,17*S*)-3-((4*R*,5*R*,6*R*)-4,5-Bis((triisopropylsilyl)oxy)-6-(((triisopropylsilyl)oxy)methyl)tetrahydro-2*H*-pyran-2-yl)-13-methyl-7,8,9,11,12,13,14,15,16,17-decahydro-6*H*-cyclopenta[*a*]phenanthren-17-yl

pivalate (17x-1). According to a literature precedent,[24] compound **17x** (95.2 mg, 0.10 mmol, 1.00 equiv) was dissolved in mixed solvent of EtOAc/MeOH (5:1, 12.0 mL), and then 10% Pd/C (150 mg) was added. The reaction was stirred under a hydrogen balloon for 24 h. The mixture was then filtered through celite and concentrated under vacuum. Column chromatography of the residue on silica gel (Petroleum ether:Ether, 1:0-20:1) afforded 1**7x-1** (82.2 mg, 86%) as a white foam: **^{1}H NMR** (500 MHz, $CDCl_3$) δ 7.24 (d, *J* = 8.1 Hz, 1H), 7.14 (d, *J* = 1.9 Hz, 1H), 7.06 (dd, *J* = 8.1, 1.9 Hz, 1H), 4.67 (dd, *J* = 9.2, 7.6 Hz, 1H), 4.45 (dd, *J* = 11.3, 2.3 Hz, 1H), 4.07 – 4.01 (m, 2H), 3.93 (dd, *J* = 10.7, 4.7 Hz, 1H), 3.84 (t, *J* = 7.3, 7.3 Hz, 1H), 3.48 – 3.45 (m, 1H), 2.88 – 2.85 (m, 2H), 2.34 – 2.18 (m, 4H), 1.93 – 1.85 (m, 2H), 1.80 – 1.70 (m, 2H), 1.54 – 1.45 (m, 3H), 1.44 – 1.29 (m, 4H), 1.22 (s, 9H), 1.13 – 1.06 (m, 63H), 0.85 (s, 3H); **^{13}C NMR** (126 MHz, $CDCl_3$) δ 178.7, 139.7, 139.2, 136.5, 126.8, 125.2, 123.2, 82.6, 82.4, 75.8, 75.6, 72.9, 64.0, 50.1, 44.4, 43.2, 42.1, 39.0, 38.5, 37.1, 29.7, 27.7, 27.4(2), 26.2, 23.5, 18.7, 18.6, 18.5(2), 18.2, 18.1, 13.9, 13.8, 12.3; **HRMS** (ESI) m/z calcd for $C_{56}H_{102}O_6Si_3Na$ [M + Na]$^+$ 977.6876, found 977.6874.

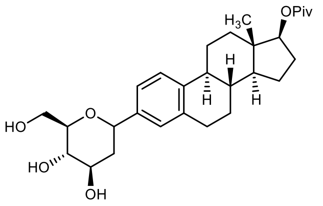

17x-2

(8*R*,9*S*,13*S*,14*S*,17*S*)-3-((4*R*,5*S*,6*R*)-4,5-Dihydroxy-6-(hydroxymethyl)tetrahydro-2*H*-pyran-2-yl)-13-methyl-7,8,9,11,12,13,14,15,16,17-decahydro-6*H*-cyclopenta[*a*]phenanthren-17-yl pivalate (17x-2). Compound **17x-1** (57.2 mg, 0.06 mmol, 1.00 equiv) was dissolved in THF (1.50 mL), and TBAF (1.00 M in THF, 0.24 mL, 4.00 equiv) was added. The reaction was stirred at rt for 2 h and then concentrated under vacuum. Column chromatography of the residue on SiO_2 (DCM:MeOH, 1:0-20:1) afforded 1**7x-2** (23.5 mg, 81%) as a white solid: **^{1}H NMR** (500 MHz, Methanol-*d*$_4$) δ 7.23 (d, *J* = 8.1 Hz, 1H), 7.13 – 7.10 (m, 2H), 4.65 (dd, *J* = 9.2, 7.5 Hz, 1H), 4.43 (dd, *J* = 11.4, 2.0 Hz, 1H), 3.90 (dd, *J* = 11.9, 2.3 Hz, 1H), 3.75 – 3.67 (m, 2H), 3.36 – 3.33 (m, 1H), 3.27 (t, *J* = 9.1, 9.1 Hz, 1H), 2.87 – 2.84 (m, 2H), 2.37 – 2.33 (m, 1H), 2.27 – 2.18 (m, 2H), 2.14 – 2.10 (m, 1H), 1.94 – 1.89 (m, 1H), 1.87 – 1.84 (m, 1H), 1.82 – 1.76 (m, 1H), 1.64 – 1.40 (m, 6H), 1.39 – 1.30 (m, 2H), 1.21 (s, 9H), 0.88 (s, 3H); **^{13}C NMR** (126 MHz, Methanol-*d*$_4$) δ 180.2, 140.5, 140.3, 137.5, 127.6, 126.2, 124.5, 83.9, 82.2, 78.7, 74.2, 73.4, 63.3, 51.1, 45.5, 44.3, 42.8, 39.9, 38.2, 30.6, 28.6, 28.4, 27.6, 27.2, 24.2, 12.6; **HRMS** (ESI) m/z calcd for $C_{29}H_{42}O_6Na$ [M + Na]$^+$ 509.2874, found 509.2878.

Diels-Alder cycloaddition with dieno-glycoside

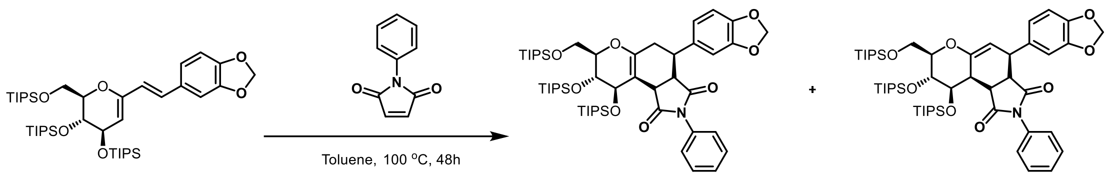

To a solution of compound **15r** (76.0 mg, 0.1 mmol, 1.00 equiv) in toluene (1.00 ml) was treated with *N*-Phenylmaleimide (34.6 mg, 0.20 mmol, 2.00 equiv), and the reaction mixture was heated at 100 °C for 48 h. After completion of the reaction, the reaction mixture concentrated under reduced pressure and purified by column chromatography on SiO_2 (Petroleum ether: EtOAc, 1:0 – 10:1) to afford **15r-1** (20.5 mg, 29%) as a yellow oil and **15r-2** (27.1 mg, 22%) as a yellow oil.

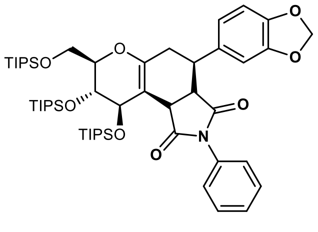

15r-1

(3a*S*,4*R*,7*R*,8*R*,9*R*,9b*S*)-4-(Benzo[*d*][1,3]dioxol-5-yl)-2-phenyl-8,9-bis((triisopropylsilyl)oxy)-7-(((triisopropylsilyl)oxy)methyl)-3a,5,7,8,9,9b-hexahydropyrano[3,2-*e*]isoindole-1,3(2*H*,4*H*)-dione (15r-1). ^{1}**H NMR** (500 MHz, Acetone-d_6) δ 7.50 – 7.46 (m, 2H), 7.42 – 7.38 (m, 1H), 7.31 – 7.29 (m, 2H), 6.87 (d, *J* = 1.8 Hz, 1H), 6.80 (dd, *J* = 8.0, 1.8 Hz, 1H), 6.74 (d, *J* = 8.0 Hz, 1H), 5.97 (s, 2H), 4.31 – 4.24 (m, 3H), 4.15 (t, *J* = 1.8, 1.8 Hz, 1H), 3.89 – 3.83 (m, 2H), 3.72 – 3.67 (m, 2H), 2.55 – 2.50 (m, 1H), 2.43 – 2.39 (m, 1H), 1.41 – 1.35 (m, 3H), 1.16 – 1.04 (m, 60H); ^{13}**C NMR** (126 MHz, Acetone-d_6) δ 177.4, 176.1, 149.6, 148.7, 147.1, 137.6, 134.0, 129.5, 128.9, 127.6, 121.2, 108.8(2), 101.9, 100.1, 82.0, 71.9, 71.7, 62.8, 47.0, 46.4, 38.8, 33.4, 19.3, 19.2, 18.5(2), 18.4, 14.4, 13.3, 12.8; **HRMS** (ESI) m/z calcd for $C_{52}H_{83}NO_8Si_3Na$ [M + Na]$^+$ 956.5324, found 956.5312.

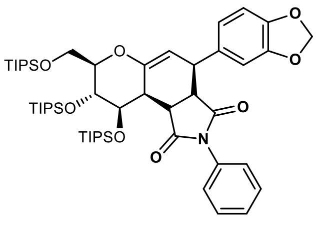

15r-2

(3a*S*,4*R*,7*R*,8*R*,9*R*,9a*S*,9b*R*)-4-(Benzo[*d*][1,3]dioxol-5-yl)-2-phenyl-8,9-bis((triisopropylsilyl)oxy)-7-(((triisopropylsilyl)oxy)methyl)-3a,7,8,9,9a,9b-hexahydropyrano[3,2-*e*]isoindole-1,3(2*H*,4*H*)-dione (15r-2). ^{1}**H NMR** (500 MHz, Acetone-d_6) δ 7.50 – 7.46 (m, 2H), 7.41 – 7.38 (m, 1H), 7.33 – 7.31 (m, 2H), 6.84 – 6.78 (m, 3H), 5.97 (s, 2H), 5.18 (t, *J* = 2.7, 2.7 Hz, 1H), 5.07 (d, *J* = 3.7 Hz, 1H), 4.38

(d, J = 3.7 Hz, 1H), 4.25 (t, J = 6.3, 6.3 Hz, 1H), 4.12 (dd, J = 10.2, 6.1 Hz, 1H), 4.05 (dd, J = 10.2, 6.5 Hz, 1H), 3.81 (dd, J = 10.6, 9.6 Hz, 1H), 3.72 – 3.69 (m, 1H), 3.27 (dd, J = 9.7, 8.4 Hz, 1H), 1.25 – 1.04 (m, 63H); **^{13}C NMR** (126 MHz, Acetone-d_6) δ 178.0, 177.3, 152.7, 148.6, 147.2, 140.4, 133.7, 129.5, 128.9, 127.8, 122.1, 109.3, 109.1, 108.8, 101.9, 86.3, 72.1, 70.9, 67.0, 48.8, 44.6, 41.5, 41.0, 18.8, 18.7(2), 18.6, 18.4, 18.2, 13.2(2), 12.8; **HRMS** (ESI) m/z calcd for $C_{52}H_{83}NO_8Si_3Na$ [M + Na]$^+$ 956.5324, found 956.5327.

Total synthesis of Tofogliflozin and its derivatives

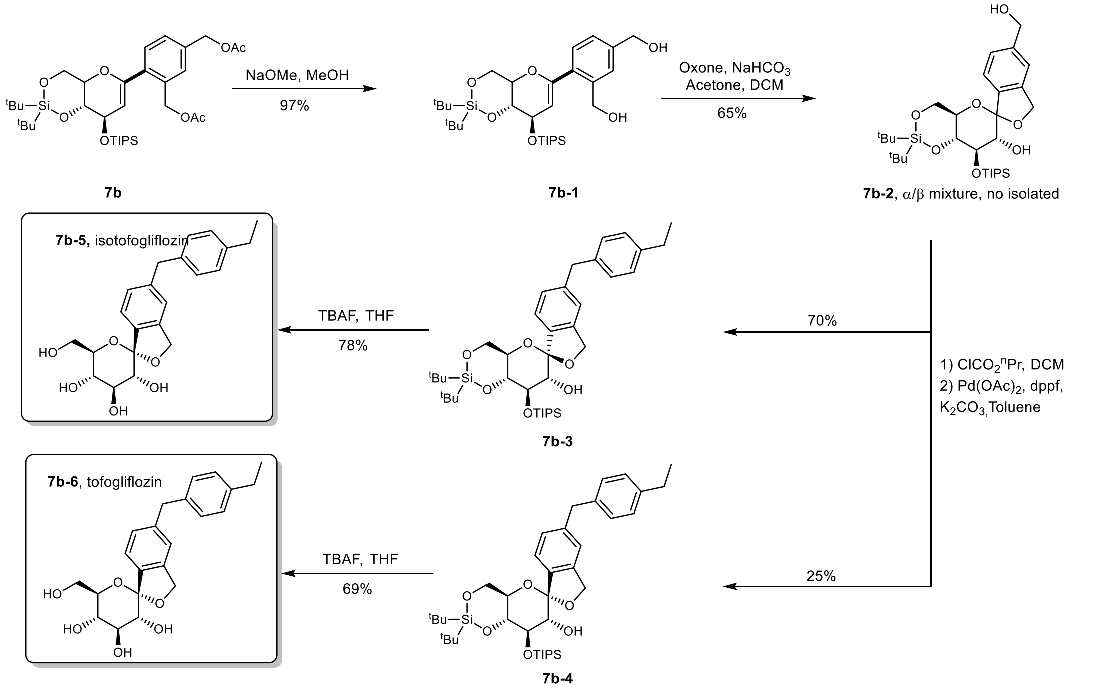

To a solution of compound **7b** (1.25 g, 1.80 mmol, 1.00 equiv) in anhydrous MeOH (5.00 ml) at rt was treated with NaOMe (4.86 mg, 0.09 mmol, 0.05 equiv). The reaction mixture was stirred at 2 h, concentrated and purified by column chromatography on SiO_2 (Petroleum ether:EtOAc, 1:0–1.5:1) to afford **7b-1** (1.05 g, 97%) as a white foam.

To a solution of compound **7b-1** (289 mg, 0.50 mmol, 1.00 equiv) in a cooled (0 °C), vigorously stirring biphasic solution of DCM (10.0 mL), saturated $NaHCO_3$ (30.0 mL), and acetone (6.00 mL), a solution of Oxone® (1.23 g, 2.00 mmol, 4.00 equiv) in H_2O (5.00 mL) was added dropwise over 10 min. The reaction mixture was stirred at that temperature for 1 hour, then gradually warmed to room temperature and stirred overnight. The reaction mixture was extracted with DCM, and the combined organic layers were washed with a saturated solution of NaCl (50.0 mL), dried over Na_2SO_4, and concentrated under reduced pressure. Column chromatography of the residue on SiO_2 (Petroleum ether:EtOAc, 1:0-3:1) afforded α/β mixture **7b-2** (193 mg, 65%) as a white foam.

To a solution of **7b-2** (495 mg, 0.80 mmol, 1.00 equiv) and DMAP (244 mg, 2.00 mmol, 2.50 equiv) in anhydrous DCM (3.00 ml), propyl chloroformate (232 mg, 1.90 mol, 2.40 equiv) was added at -10 °C. The reaction mixture was stirred for 30 min at 0 °C and then for 3 h at rt. After completion, the reaction mixture concentrated under reduced

pressure and column chromatography of the residue on SiO_2 (Petroleum ether:EtOAc, 1:0-10:1) afforded the acylation product (540 mg, 94%) as a white solid.

In a round flask, the above acylation product (530 mg, 0.75 mmol, 1.00 equiv), potassium carbonate (124 mg, 0.09 mmol, 1.20 equiv), 4-ethylphenylboronic acid (170.0 mg, 1.13 mmol, 1.50 equiv), $Pd(OAc)_2$ (16.8 mg, 0.075 mmol, 0.10 equiv), and DPPF (49.9 mg, 0.09 mmol, 0.12 equiv) were combined. DME (5.00 mL) was added, and the reaction mixture was heated at 85 °C for 8 h. After completion, the reaction mixture was concentrated under reduced pressure, and purified by column chromatography on SiO_2 (Petroleum ether:EtOAc, 1:0-10:1) to afforded **7b-3** (354 mg, 70%) as a white foam and **7b-4** (128 mg, 25%) as a white foam.

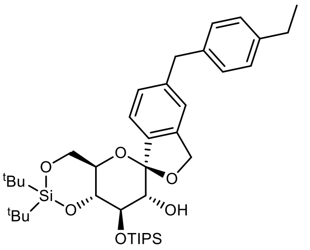

7b-3

(1*R*,4a'*R*,7'*R*,8'*R*,8a'*R*)-2',2'-Di-*tert*-butyl-5-(4-ethylbenzyl)-8'-((triisopropylsilyl)oxy)-4a',7',8',8a'-tetrahydro-3*H*,4'*H*-spiro[isobenzofuran-1,6'-pyrano[3,2-*d*][1,3,2]dioxasilin]-7'-ol (7b-3). ^{1}**H NMR** (400 MHz, $CDCl_3$) δ 7.35 (s, 1H), 7.30 – 7.23 (m, 2H), 7.14 – 7.10 (m, 4H), 5.24 (d, *J* = 12.6 Hz, 1H), 5.04 (d, *J* = 12.6 Hz, 1H), 4.36 (dd, *J* = 9.5, 8.5 Hz, 1H), 3.99 – 3.85 (m, 6H), 3.76 – 3.70 (m, 1H), 2.63 (q, *J* = 7.6, 7.6, 7.6 Hz, 2H), 2.18 (d, *J* = 3.6 Hz, 1H), 1.27 – 1.20 (m, 6H), 1.13 – 1.12 (m, 18H), 1.08 (s, 9H), 1.02 (s, 9H); ^{13}**C NMR** (101 MHz, $CDCl_3$) δ 142.3, 140.8, 139.7, 137.7, 136.6, 130.4, 128.8, 128.3, 124.0, 122.1, 111.4, 78.6, 76.4, 76.3, 72.2, 68.6, 67.1, 42.0, 28.6, 27.6, 27.1, 22.9, 20.1, 18.5(2), 15.7, 13.0; **HRMS** (ESI) m/z calcd for $C_{39}H_{62}O_6Si_2Na$ $[M + Na]^+$ 705.3977, found 705.3982.

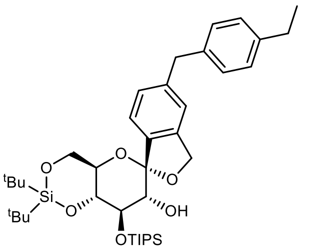

7b-4

(1*S*,4a'*R*,7'*R*,8'*R*,8a'*R*)-2',2'-di-*tert*-butyl-5-(4-ethylbenzyl)-8'-((triisopropylsilyl)oxy)-4a',7',8',8a'-tetrahydro-3*H*,4'*H*-spiro[isobenzofuran-1,6'-pyrano[3,2-*d*][1,3,2]dioxasilin]-7'-ol (7b-4). ^{1}**H NMR** (500 MHz, Acetone-d_6) δ 7.29 (s, 1H), 7.25 – 7.21 (m, 2H), 7.17 – 7.11 (m, 4H), 5.12 (s, 2H), 4.09 (t, *J* = 8.7, 8.7 Hz, 1H), 4.04 – 3.97 (m, 4H), 3.88 – 3.83 (m, 2H), 3.82 – 3.79 (m, 1H), 2.58 (q, *J* = 7.7, 7.6, 7.6 Hz, 2H), 2.07 (d, *J* = 2.3 Hz, 1H), 1.30 – 1.24 (m, 3H), 1.19 – 1.14 (m, 21H),

1.09 (s, 9H), 1.03 (s, 9H); **^{13}C NMR** (126 MHz, Acetone-d_6) δ 142.7, 142.1, 139.8, 139.4, 139.2, 130.8, 129.6, 128.7, 123.3, 121.6, 111.7, 79.6, 78.2, 78.1, 75.7, 75.5, 73.3, 69.5, 67.6, 41.8, 29.0, 28.0, 27.5, 23.3, 20.5, 19.0, 18.9, 16.1, 13.7; **HRMS** (ESI) m/z calcd for $C_{39}H_{62}O_6Si_2Na$ $[M + Na]^+$ 705.3977, found 705.3976.

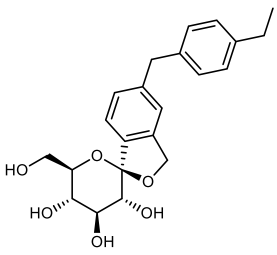

7b-5

(1*R*,3'*R*,4'*S*,5'*S*,6'*R*)-5-(4-Ethylbenzyl)-6'-(hydroxymethyl)-3',4',5',6'-tetrahydro-3*H*-spiro[isobenzofuran-1,2'-pyran]-3',4',5'-triol (7b-5). To a solution of compound **7b-3** (136 mg, 0.20 mmol, 1.00 equiv) in THF (8.00 mL) was added a solution of TBAF in THF (1.00 M, 0.80 mmol, 0.80 mL, 4.00 equiv) at 0 °C, and this reaction mixture was stirred at 45 °C for 5 h. The resulting mixture was concentrated under reduced pressure and purified by column chromatography on SiO_2 (DCM/MeOH, 1:0-10:1) to afford **7b-5** included some TBAF. Subsequent conventional formylation, column chromatography purification, and deformylation using catalytic amount of sodium methoxide afforded pure **7b-5** (60.2 mg, 78%) as a white foam: **^{1}H NMR** (500 MHz, Methanol-d_4) δ 7.49 (s, 1H), 7.24 – 7.17 (m, 2H), 7.13 – 7.08 (m, 4H), 5.17 (d, J = 12.4 Hz, 1H), 5.01 (d, J = 12.4 Hz, 1H), 4.09 (t, J = 9.5, 9.5 Hz, 1H), 3.98 (s, 2H), 3.76 – 3.70 (m, 2H), 3.66 – 3.59 (m, 2H), 3.52 (t, J = 9.3, 9.3 Hz, 1H), 2.58 (q, J = 7.6, 7.6, 7.6 Hz, 2H), 1.19 (t, J = 7.6, 7.6 Hz, 3H); **^{13}C NMR** (126 MHz, Methanol-d_4) δ 143.2, 141.7, 141.1, 139.7, 138.1, 131.0, 129.8, 128.9, 125.4, 122.6, 112.7, 76.6, 75.9, 75.5, 72.7, 71.9, 62.9, 42.2, 29.4, 16.3; Characterization data matched the literature report [25].

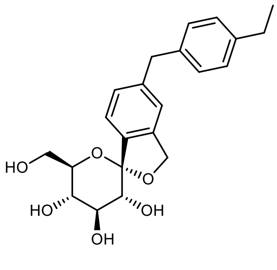

7b-6

(1*S*,3'*R*,4'*S*,5'*S*,6'*R*)-5-(4-ethylbenzyl)-6'-(hydroxymethyl)-3',4',5',6'-tetrahydro-3*H*-spiro[isobenzofuran-1,2'-pyran]-3',4',5'-triol (7b-6). To a solution of compound **7b-4** (102 mg, 0.15 mmol, 1.00 equiv) in THF (6.00 mL) was added a solution of TBAF in THF (1.00 M, 0.60 mmol, 0.60 mL, 4.00 equiv) at 0 °C, and this reaction mixture was stirred at 45 °C for 5 h. The resulting mixture was concentrated under reduced pressure and purified by column chromatography on SiO_2 (DCM/MeOH 1:0–10:1) to afford **7b-6** included some TBAF. Subsequent conventional formylation, column chromatography purification, and deformylation using catalytic amount of sodium

methoxide afforded pure **7b-6** (39.8 mg, 69%) as a white solid: **^{1}H NMR** (500 MHz, Methanol-d_4) δ 7.23 – 7.18 (m, 3H), 7.13 – 7.09 (m, 4H), 5.18 – 5.03 (m, 2H), 3.96 (s, 2H), 3.84 – 3.76 (m, 4H), 3.66 (dd, J = 12.0, 5.7 Hz, 1H), 3.50 – 3.43 (m, 1H), 2.59 (q, J = 7.6, 7.6, 7.6 Hz, 2H), 1.20 (t, J = 7.6, 7.6 Hz, 3H); **^{13}C NMR** (126 MHz, Methanol-d_4) δ 143.2, 142.6, 140.2, 139.8, 139.7, 131.1, 129.9, 128.9, 123.6, 121.8, 111.6, 76.4, 76.2, 74.9, 73.4, 71.8, 62.8, 42.2, 29.4, 16.3; Characterization data matched the literature report[26].

2.7 Copies of NMR spectra

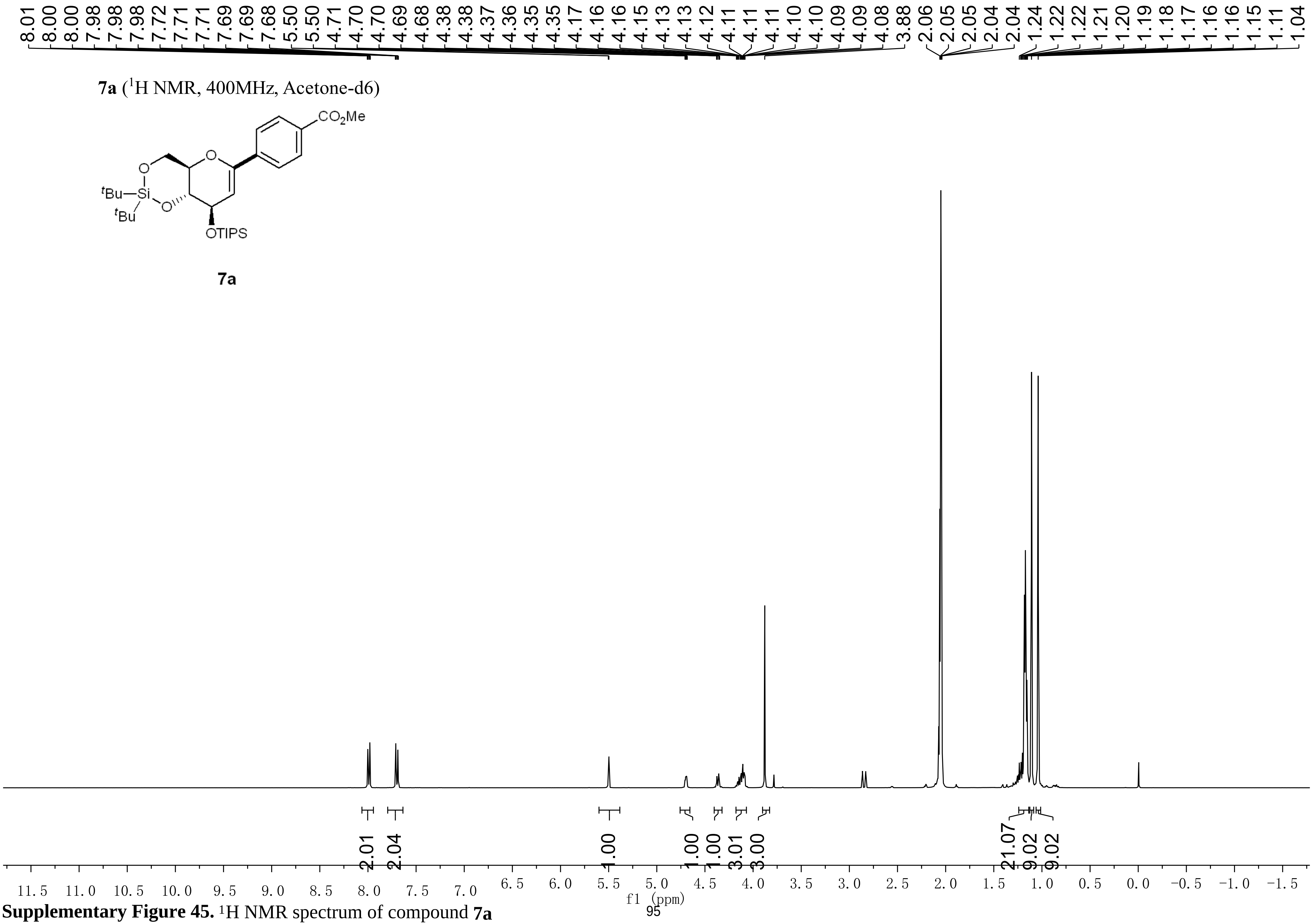

Supplementary Figure 45. ^{1}H NMR spectrum of compound **7a**

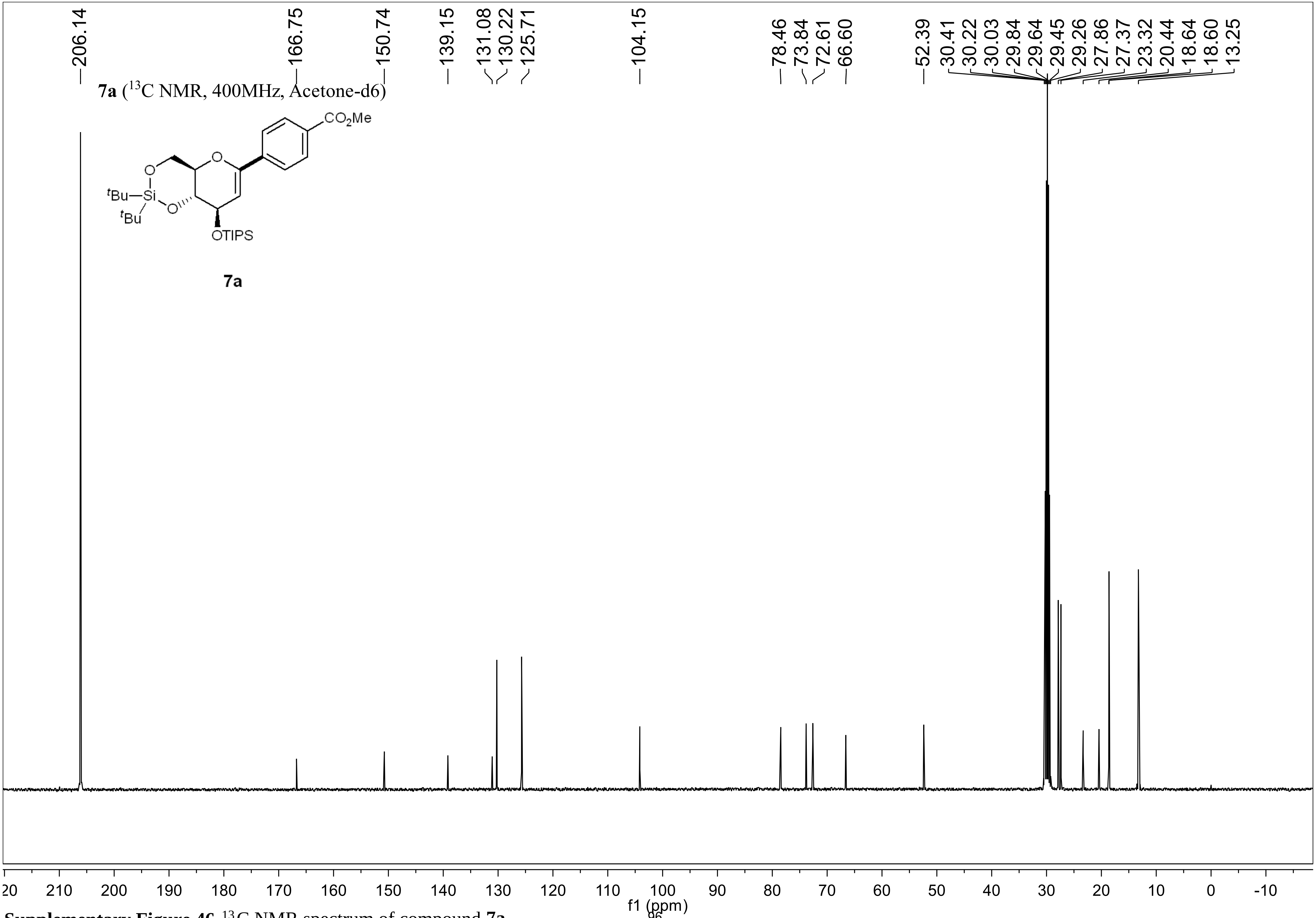

Supplementary Figure 46. ^{13}C NMR spectrum of compound **7a**

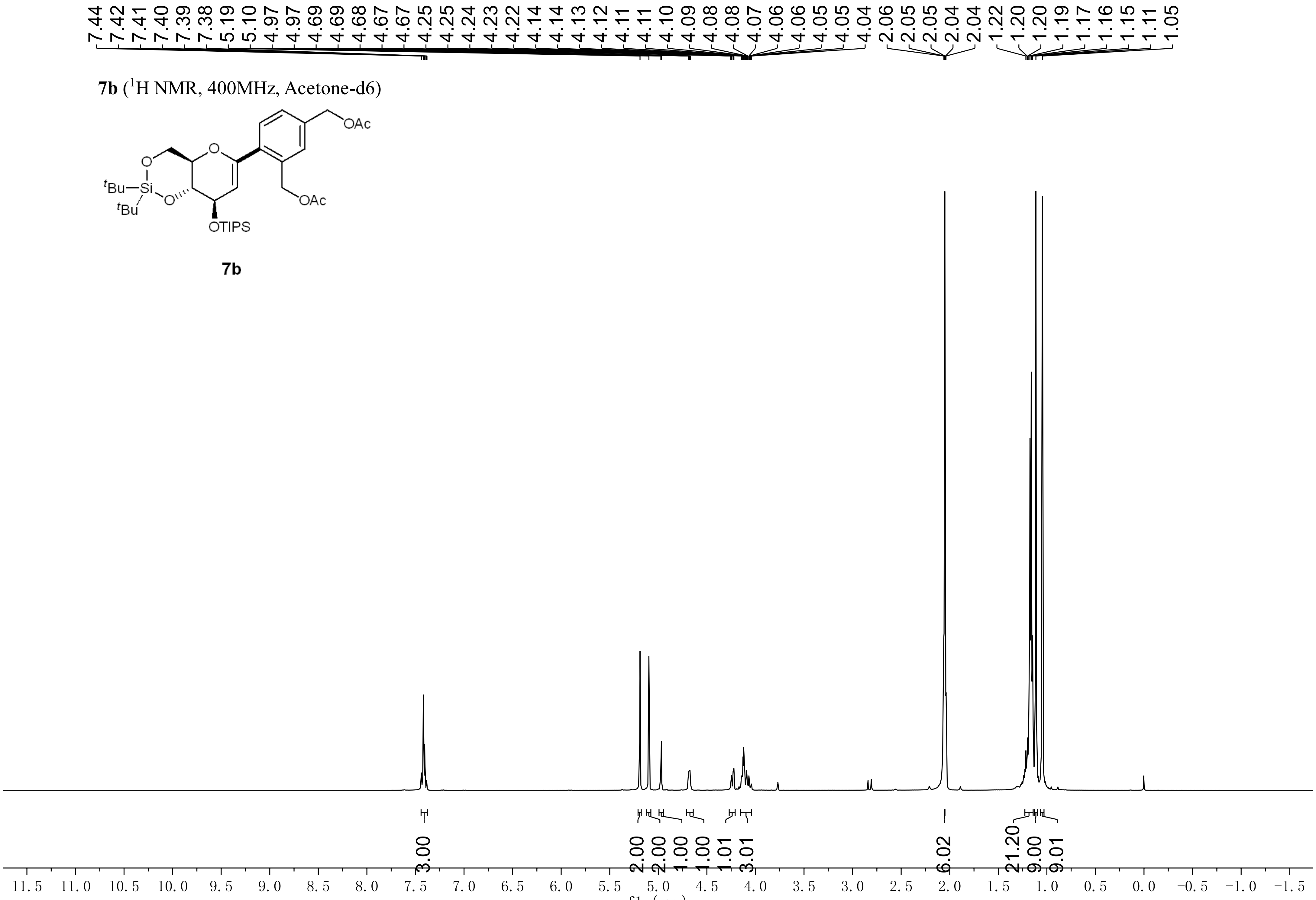

7b

Supplementary Figure 47. ^{1}H NMR spectrum of compound **7b**

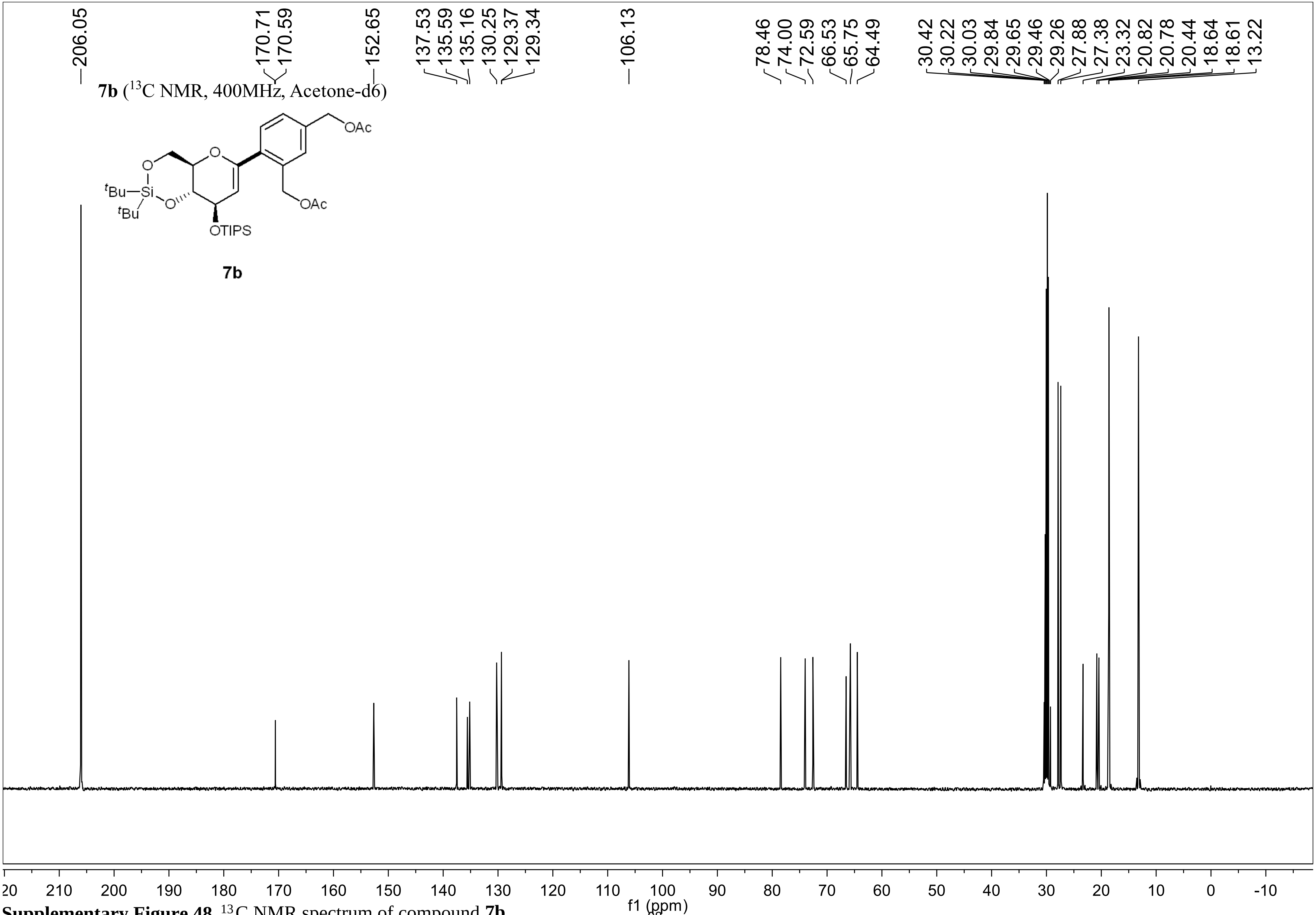

Supplementary Figure 48. ^{13}C NMR spectrum of compound **7b**

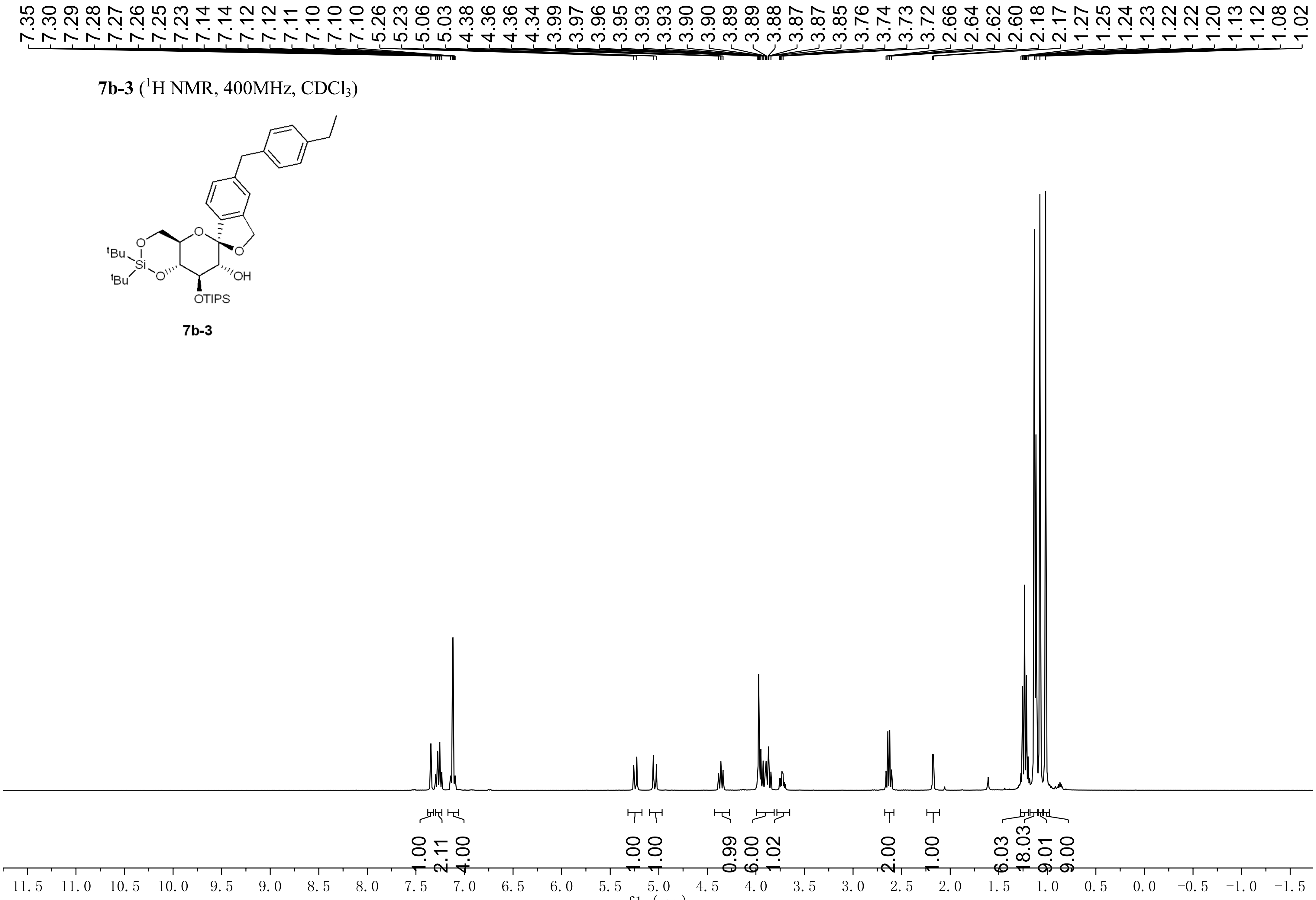

Supplementary Figure 49. ^{1}H NMR spectrum of compound **7b-3**

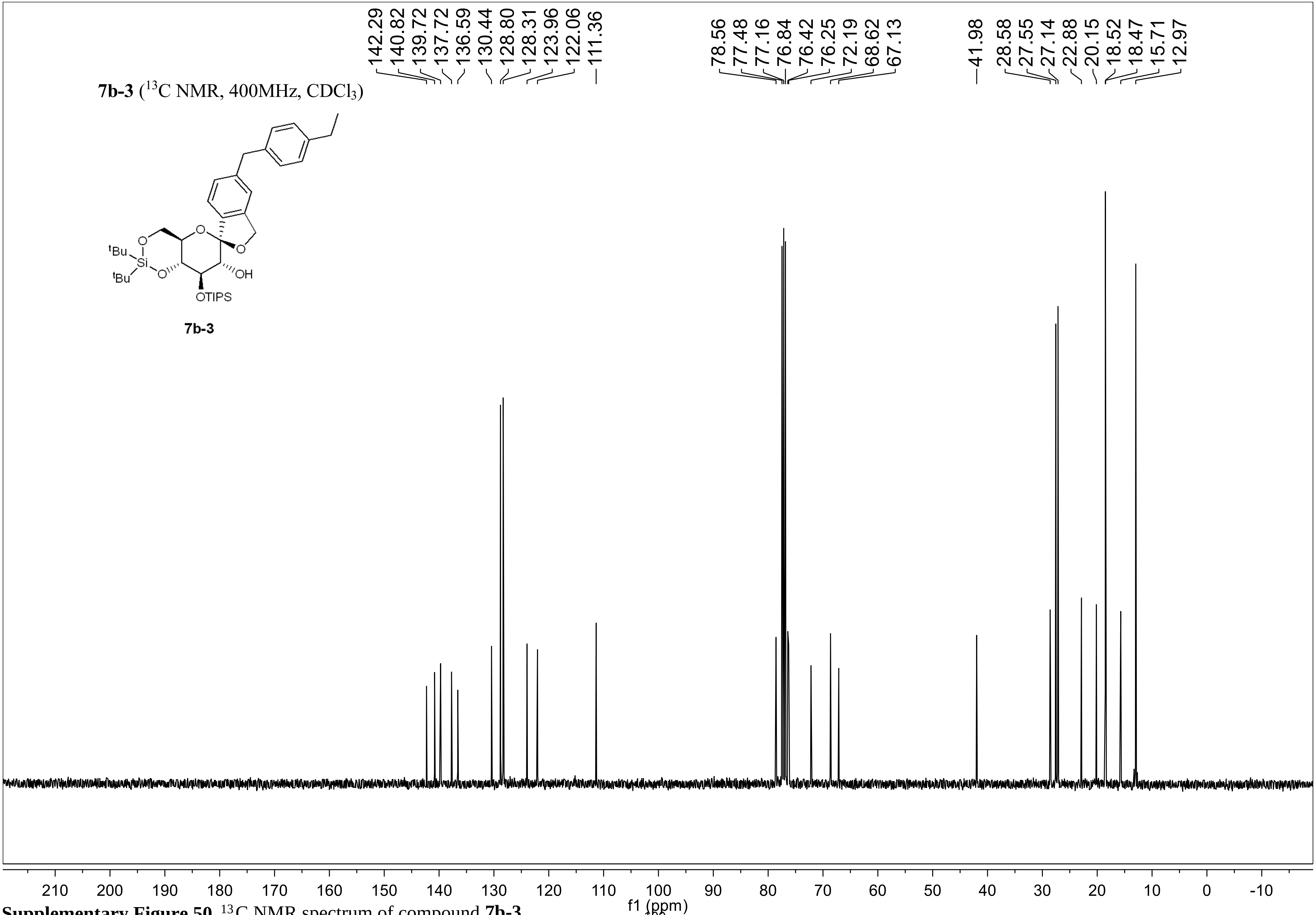

Supplementary Figure 50. ^{13}C NMR spectrum of compound **7b-3**

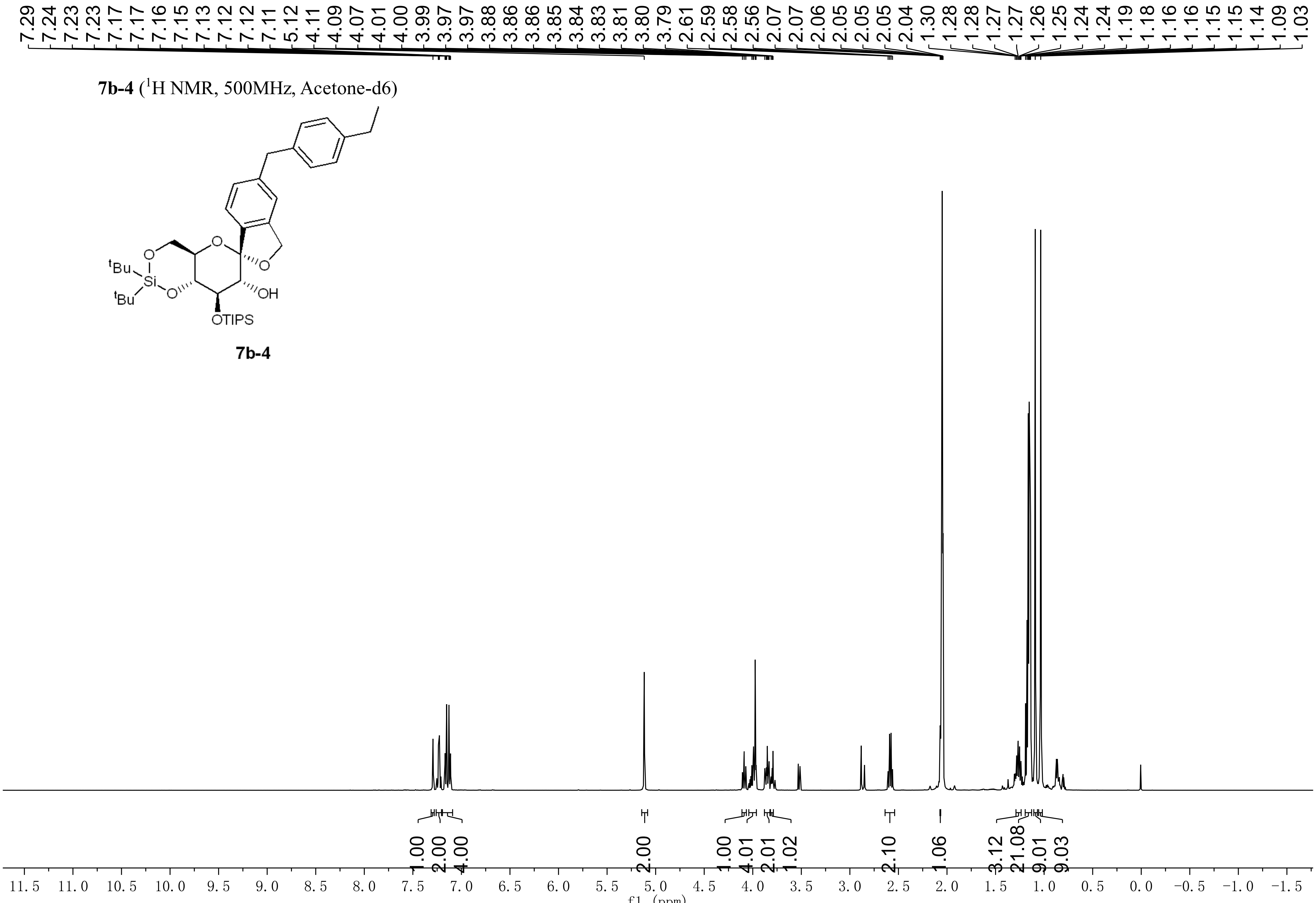

Supplementary Figure 51. ^{1}H NMR spectrum of compound **7b-4**

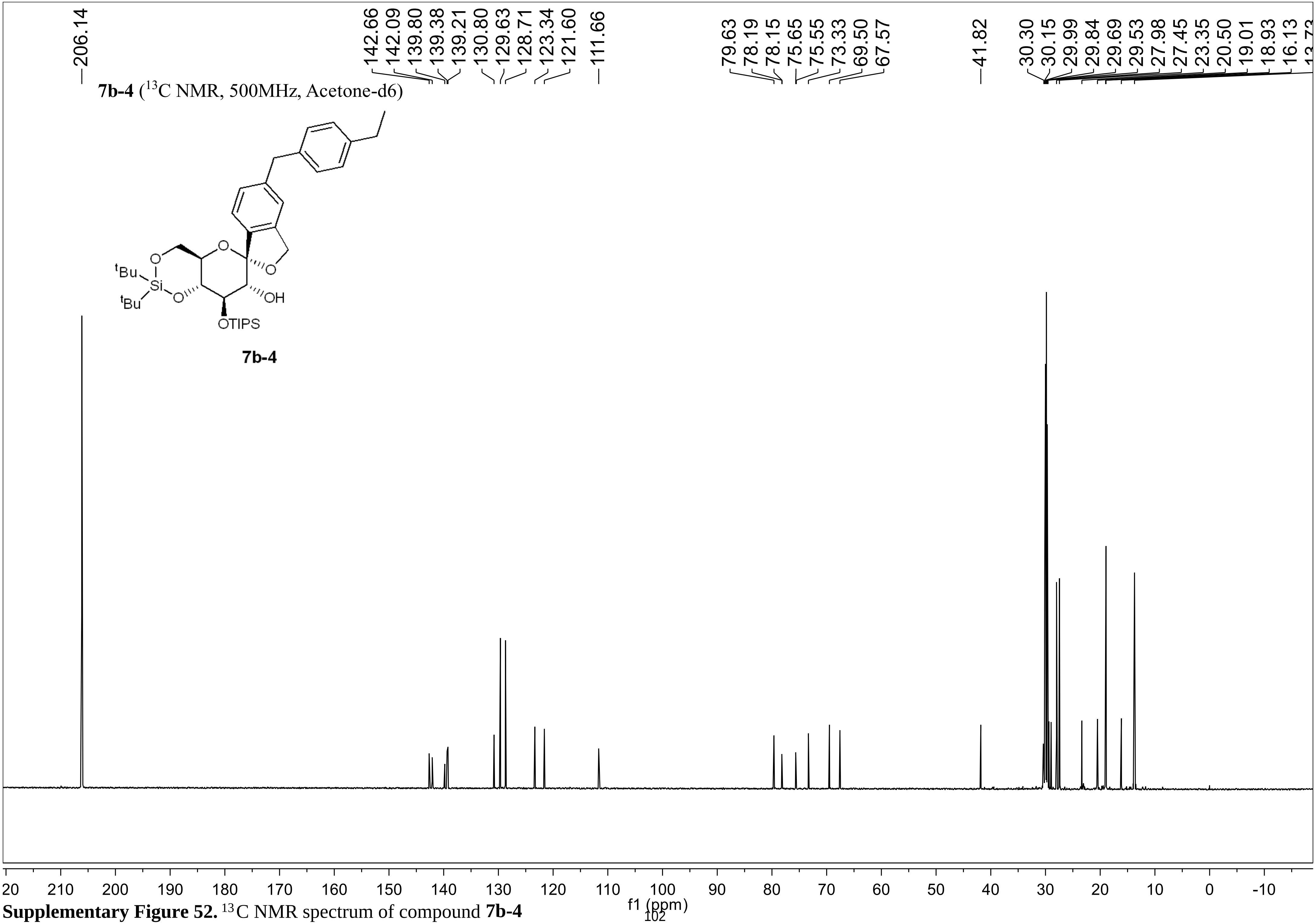

Supplementary Figure 52. ^{13}C NMR spectrum of compound **7b-4**

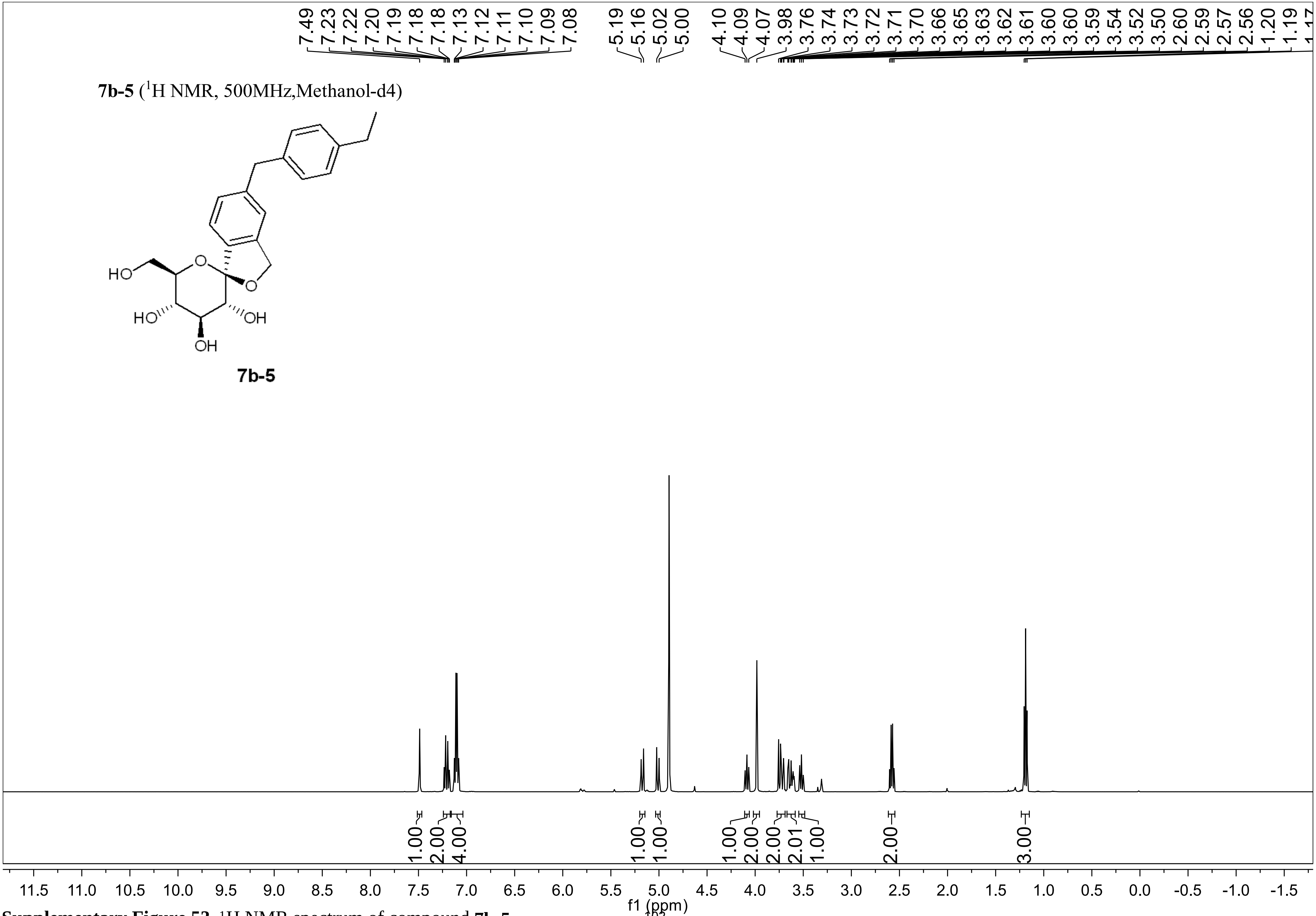

Supplementary Figure 53. ^{1}H NMR spectrum of compound **7b-5**

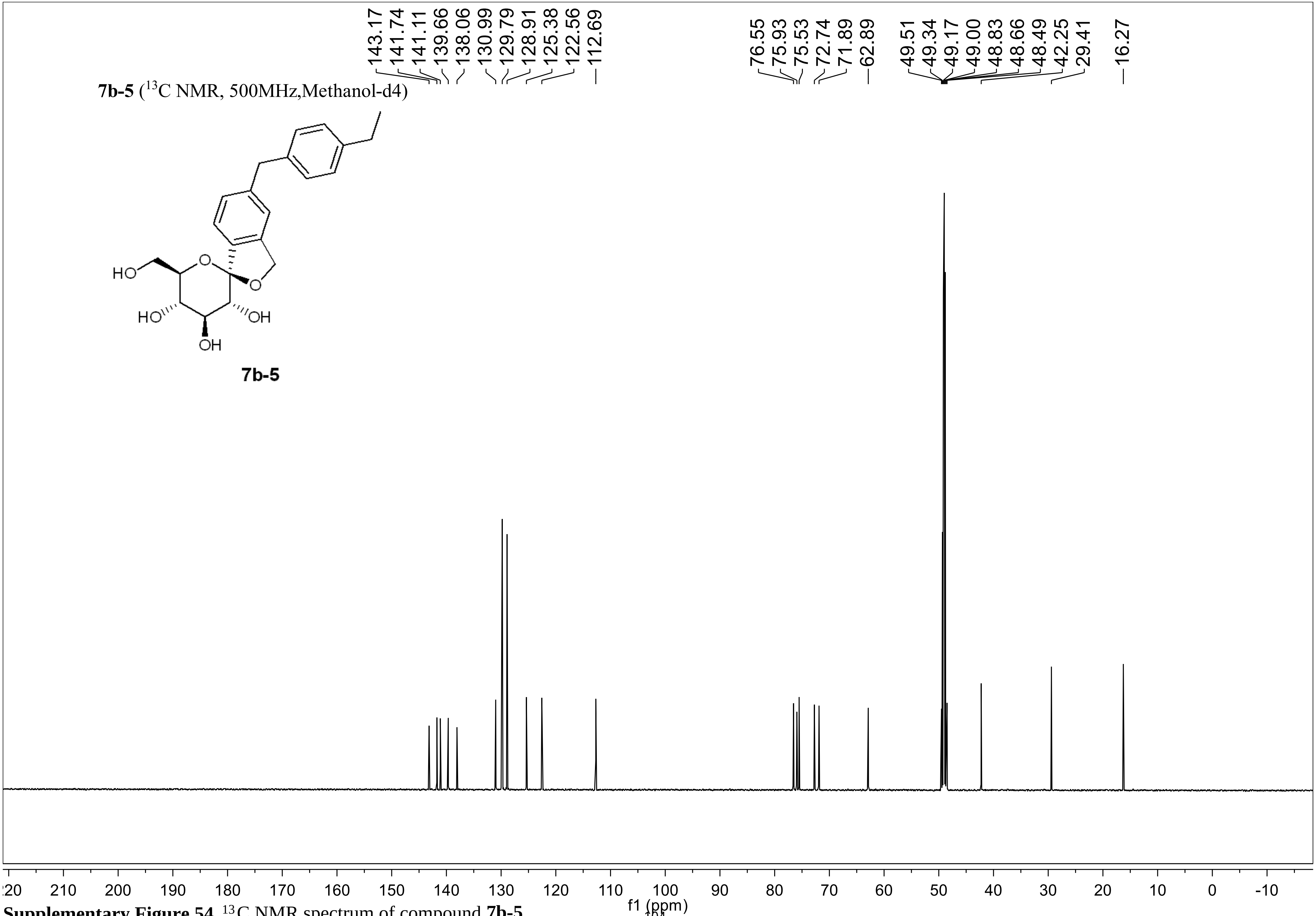

Supplementary Figure 54. ^{13}C NMR spectrum of compound **7b-5**

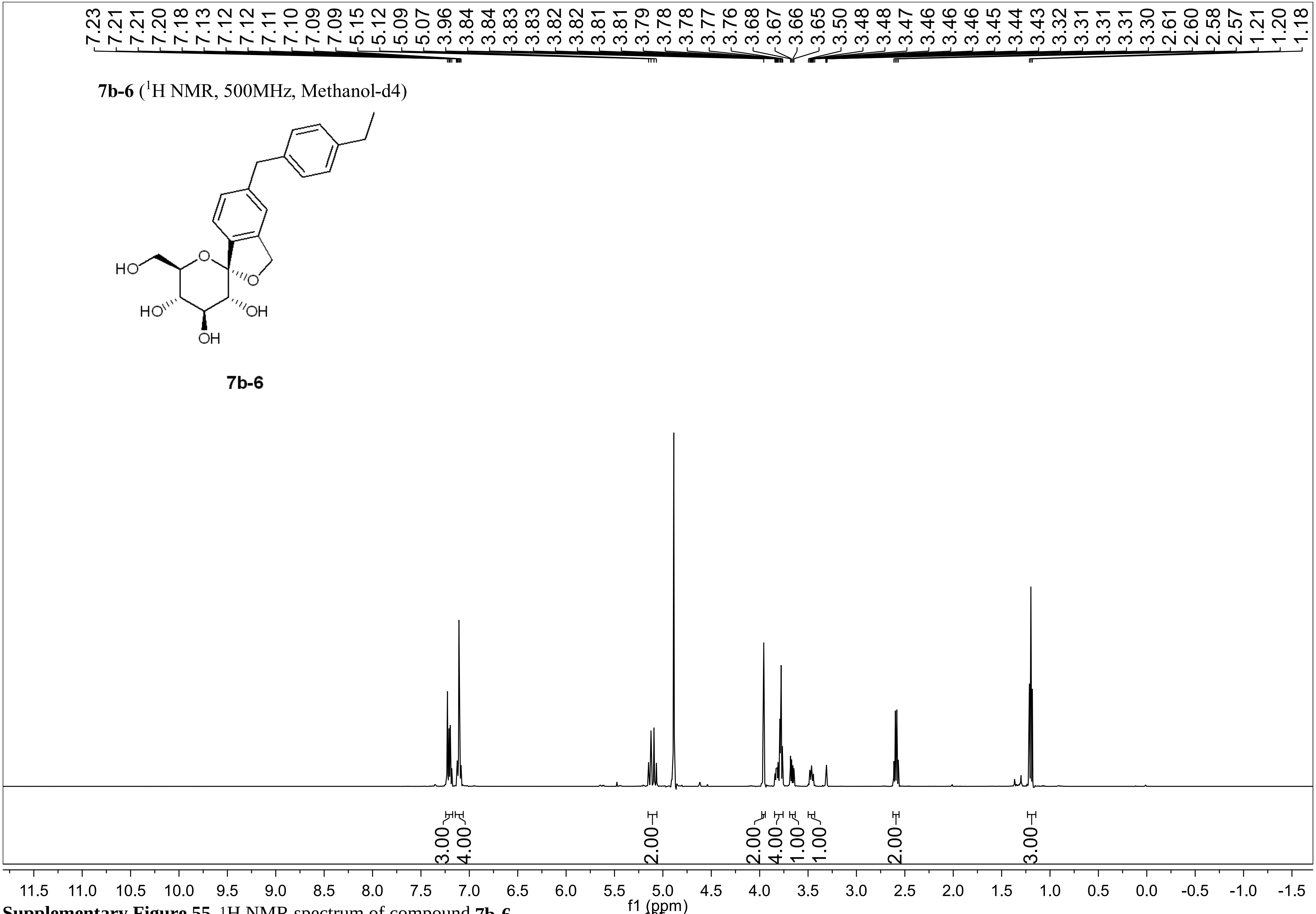

Supplementary Figure 55. ^{1}H NMR spectrum of compound **7b-6**

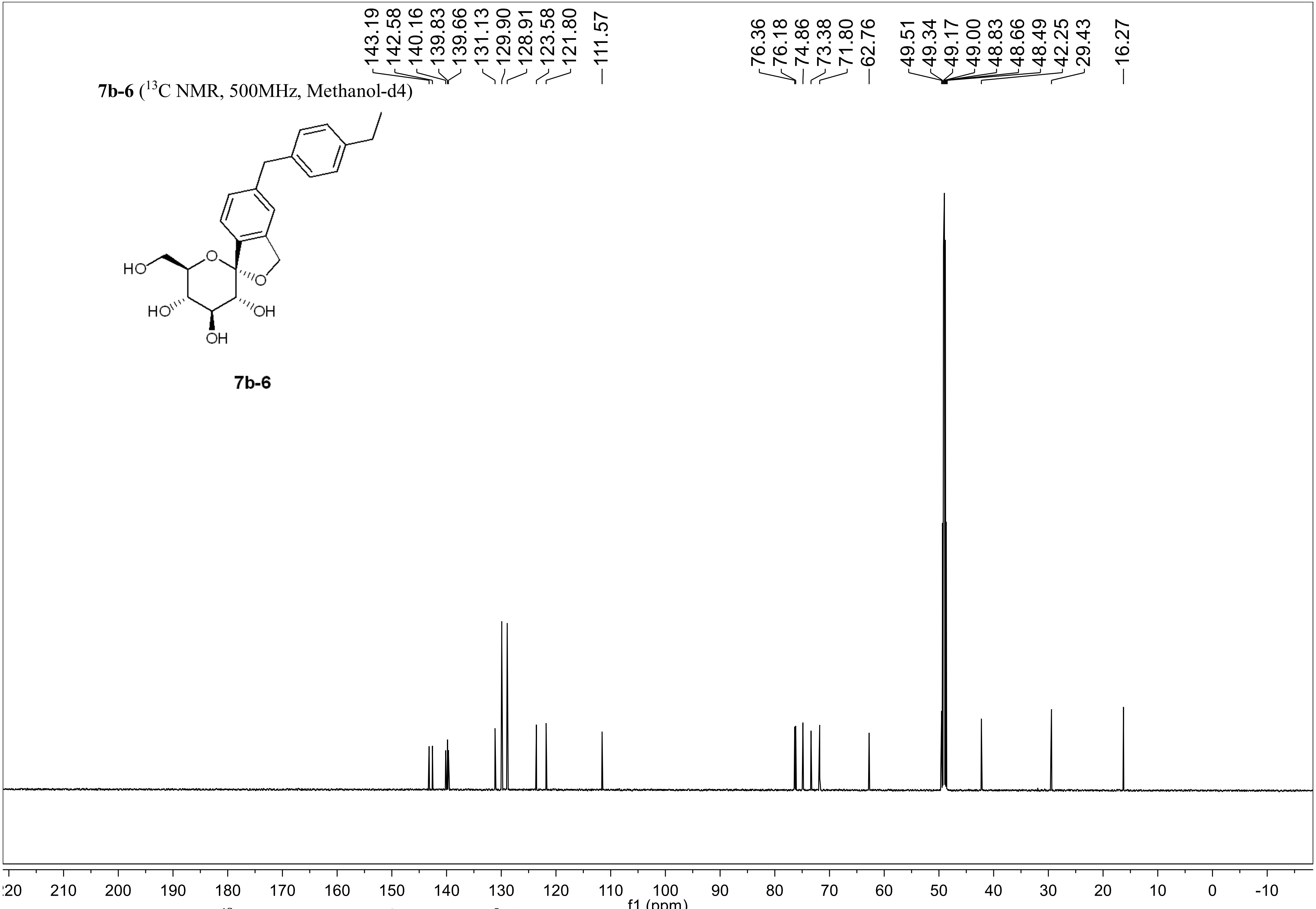

Supplementary Figure 56. ^{13}C NMR spectrum of compound **7b-6**

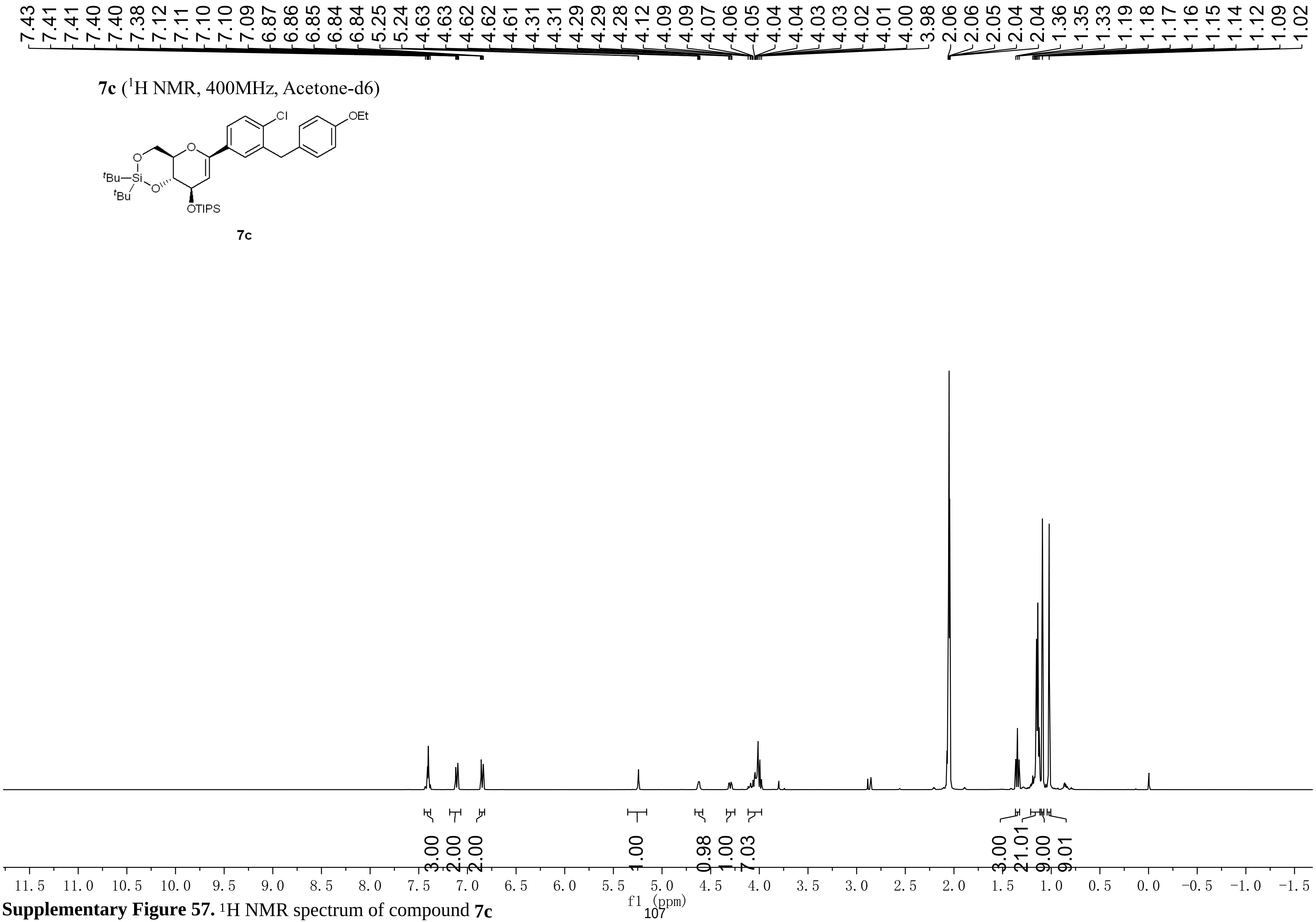

Supplementary Figure 57. ^{1}H NMR spectrum of compound **7c**

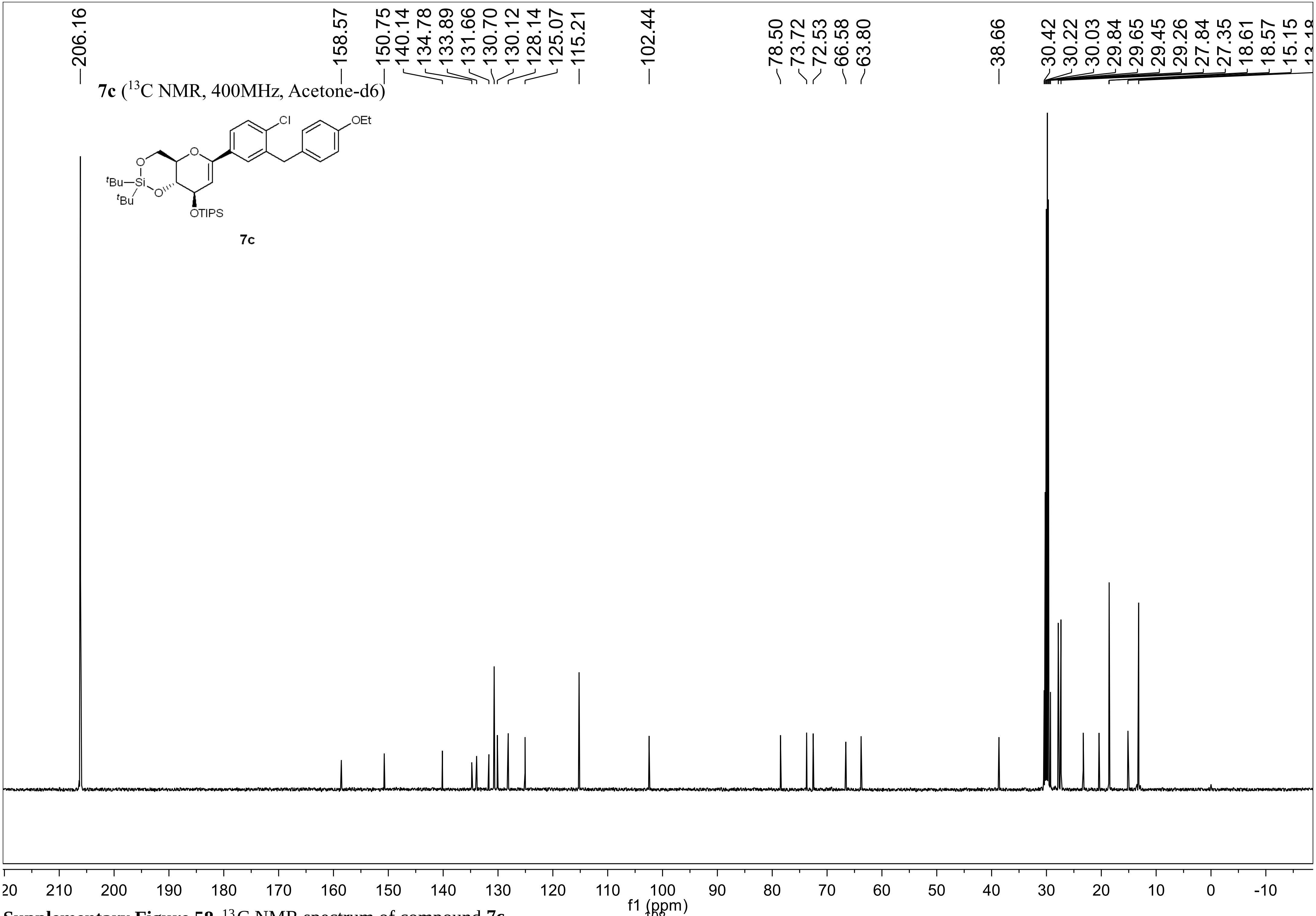

Supplementary Figure 58. ^{13}C NMR spectrum of compound **7c**

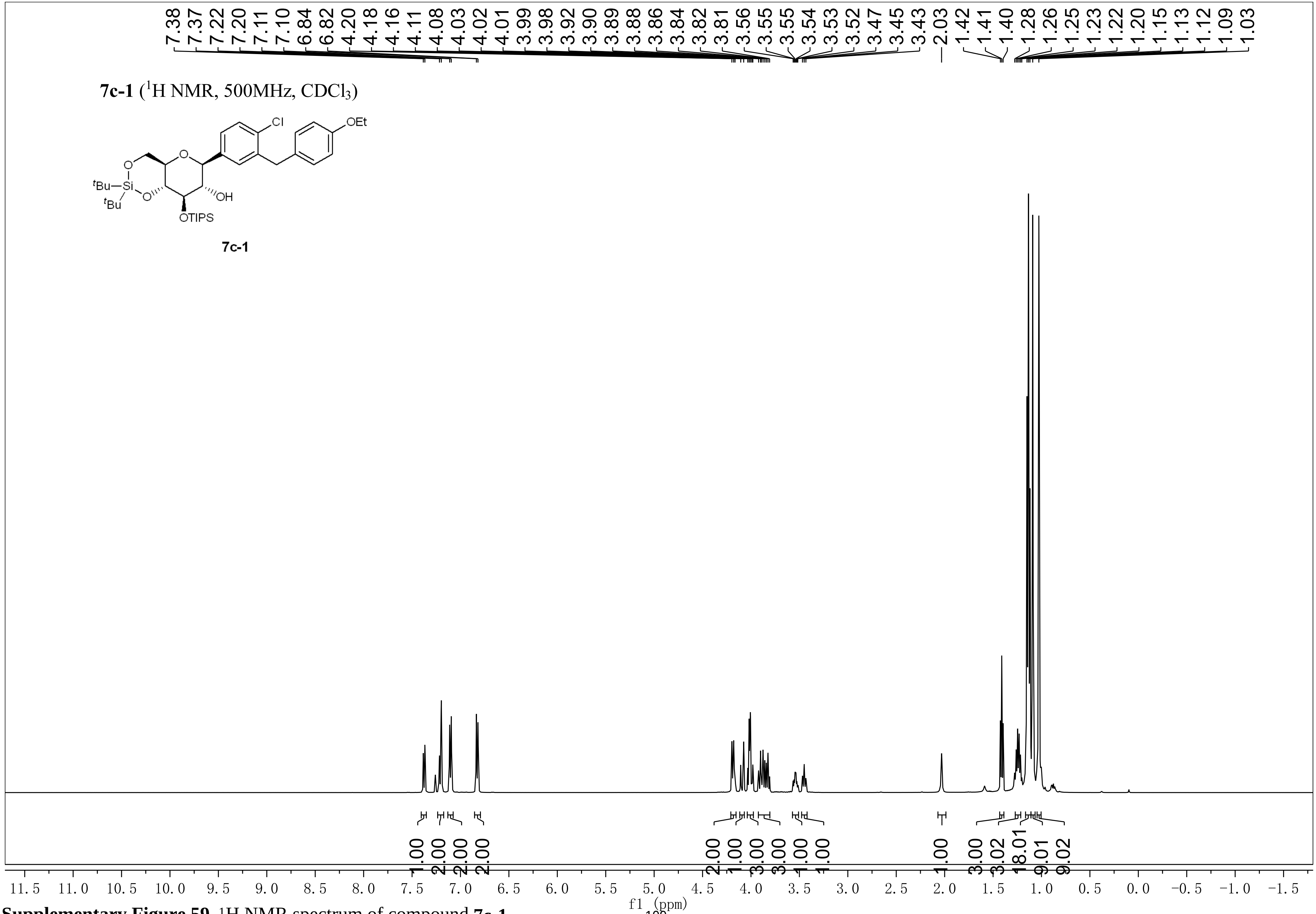

Supplementary Figure 59. ^{1}H NMR spectrum of compound **7c-1**

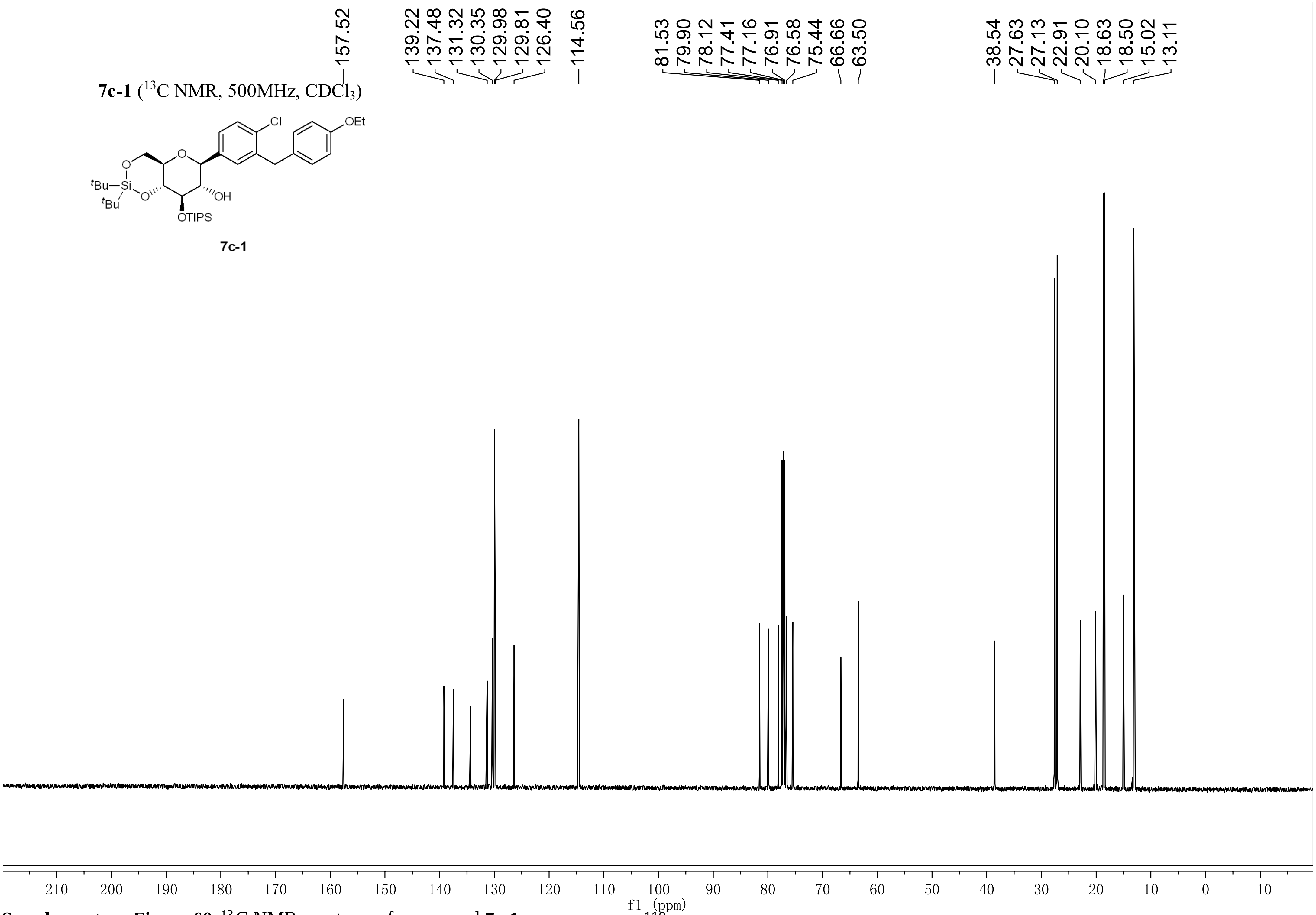

Supplementary Figure 60. ^{13}C NMR spectrum of compound **7c-1**

7c-1 (H-H COSY, 500MHz, $CDCl_3$)

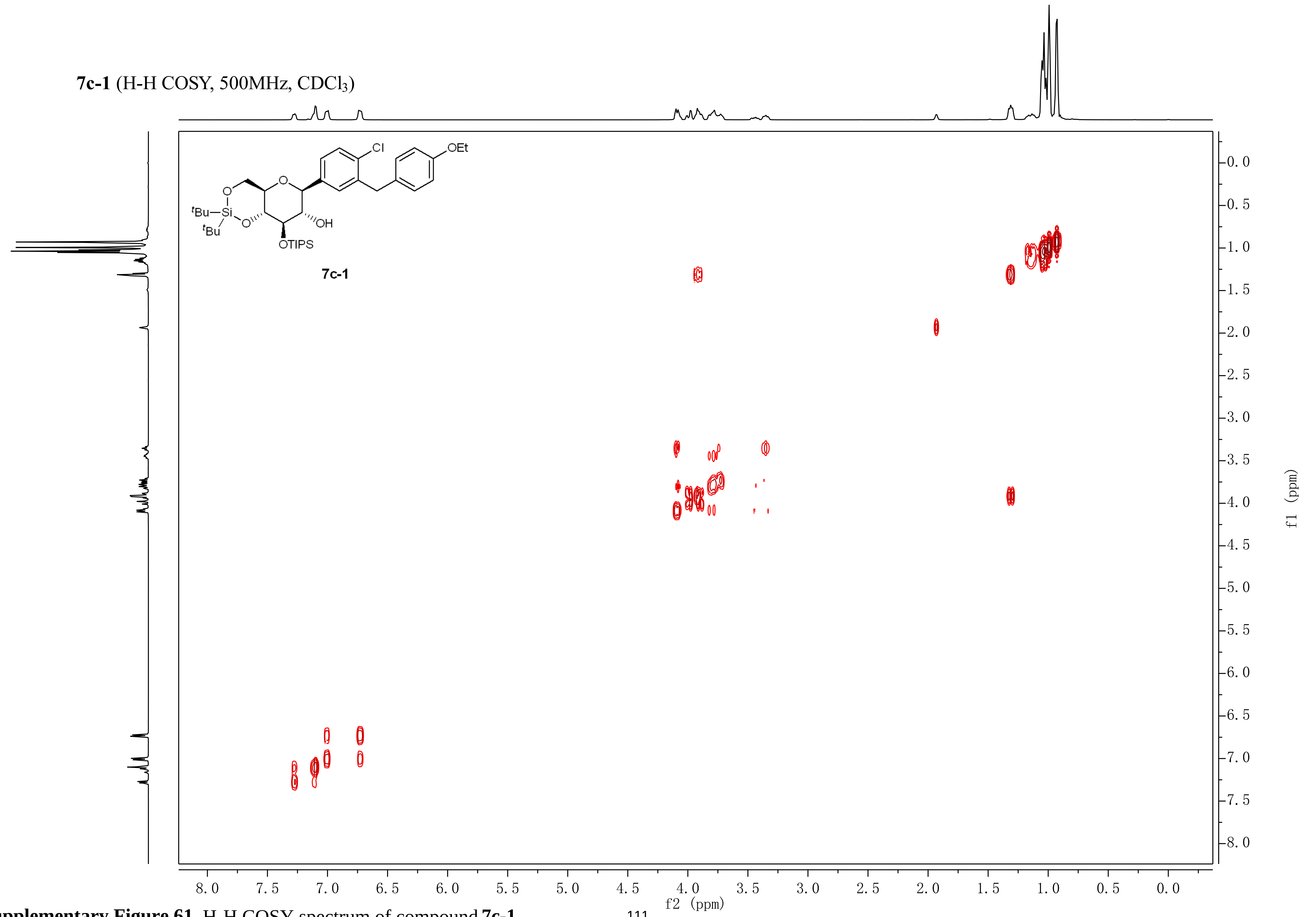

Supplementary Figure 61. H-H COSY spectrum of compound **7c-1**

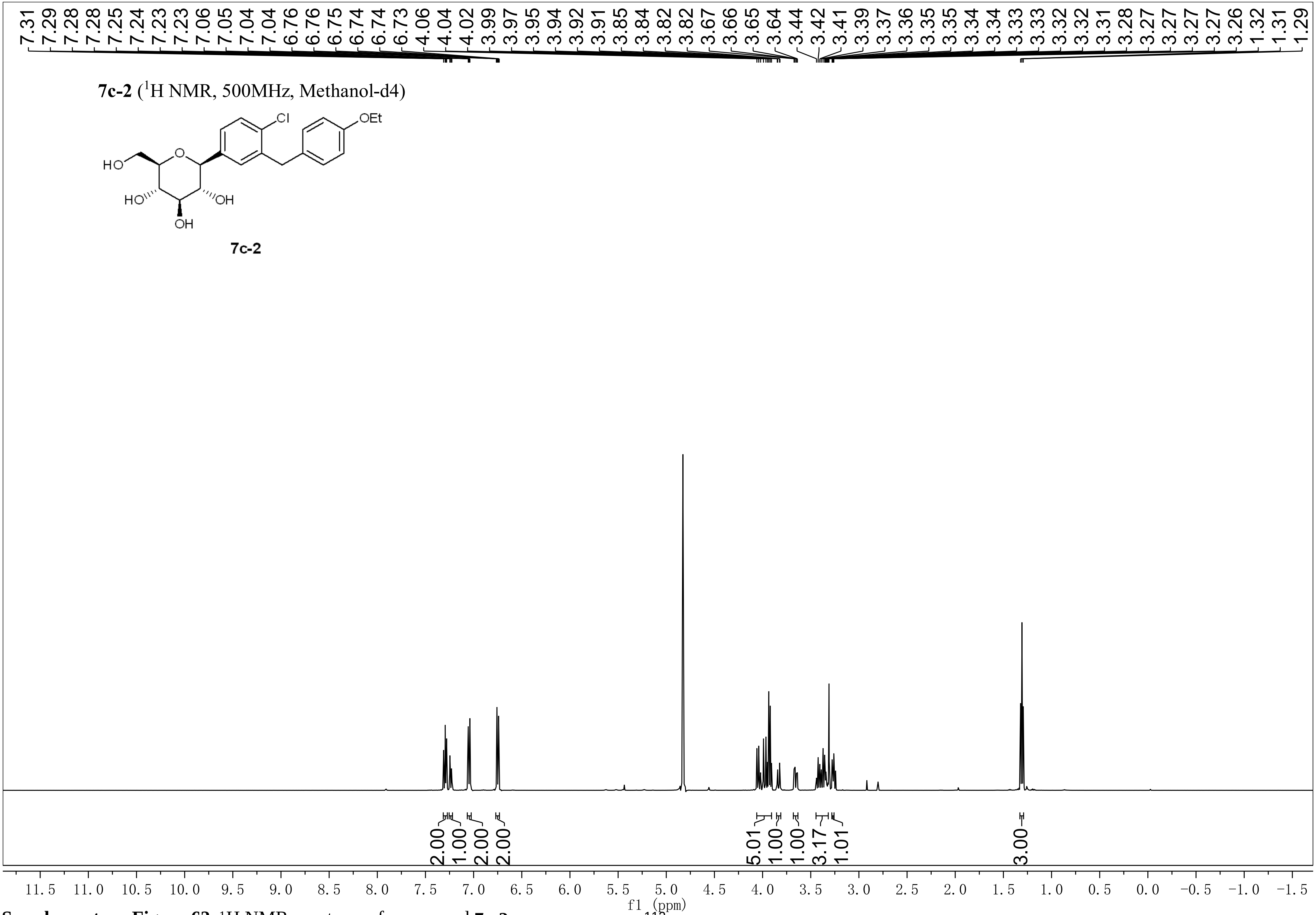

Supplementary Figure 62. ^{1}H NMR spectrum of compound **7c-2**

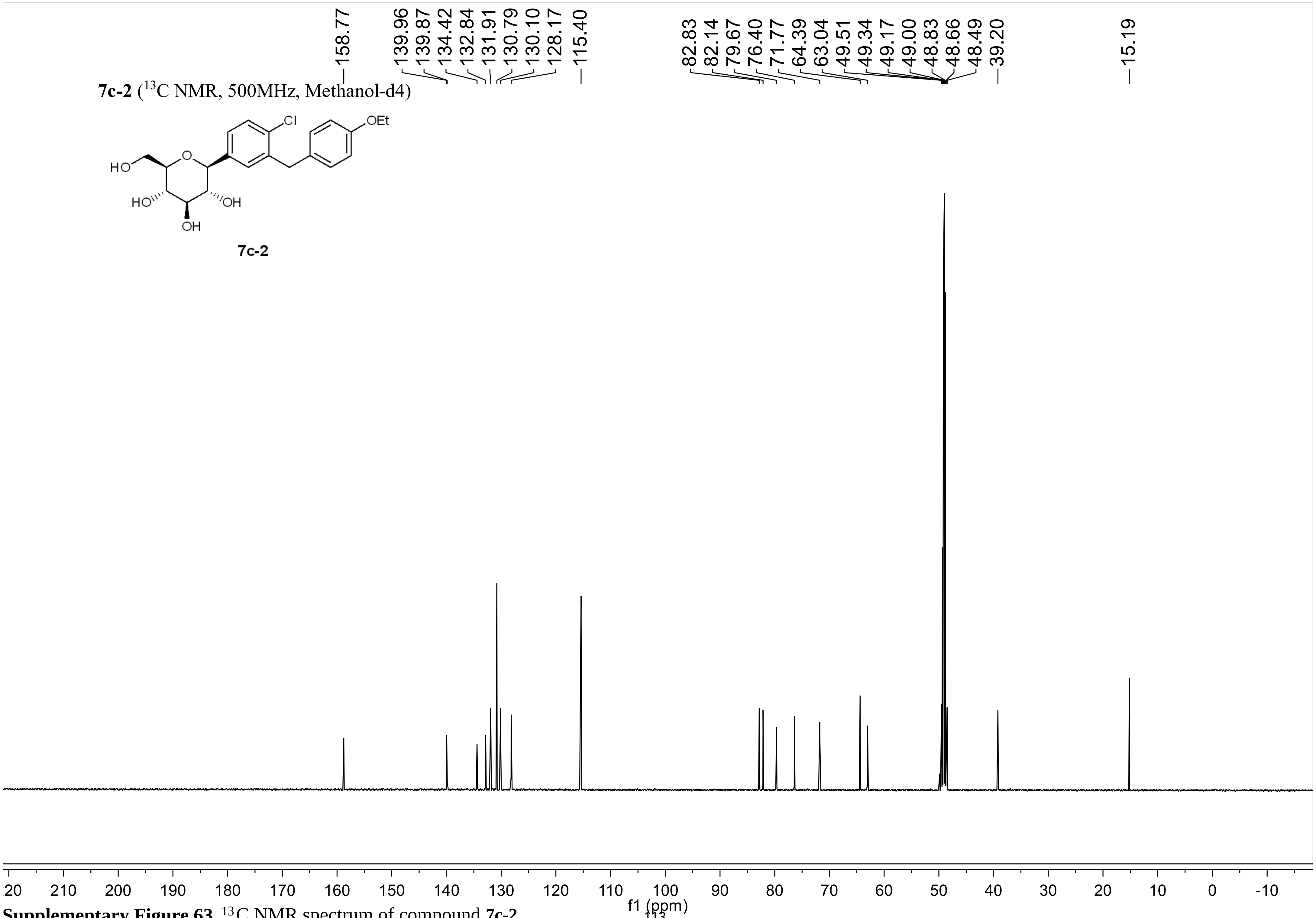

Supplementary Figure 63. ^{13}C NMR spectrum of compound **7c-2**

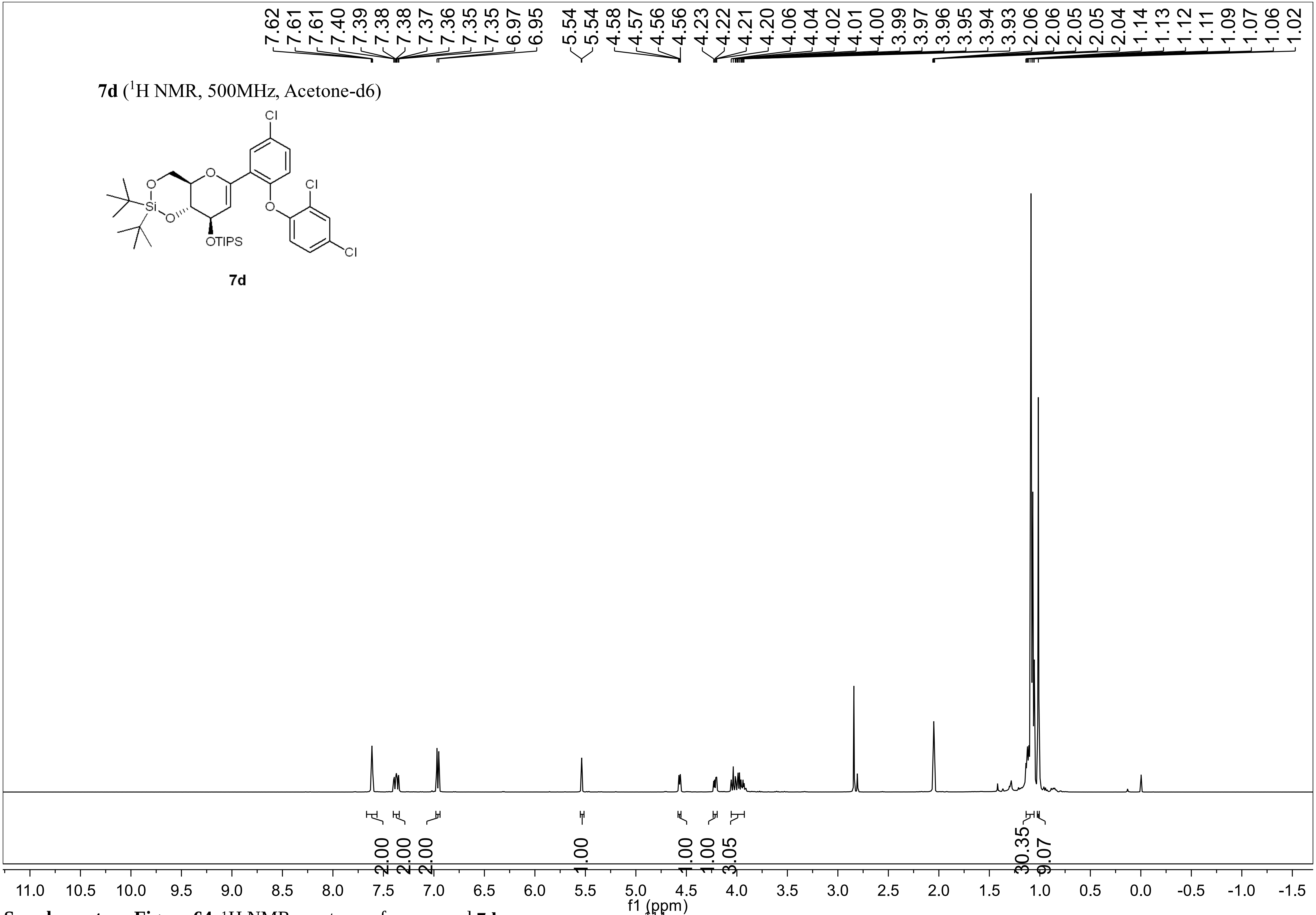

Supplementary Figure 64. ^{1}H NMR spectrum of compound **7d**

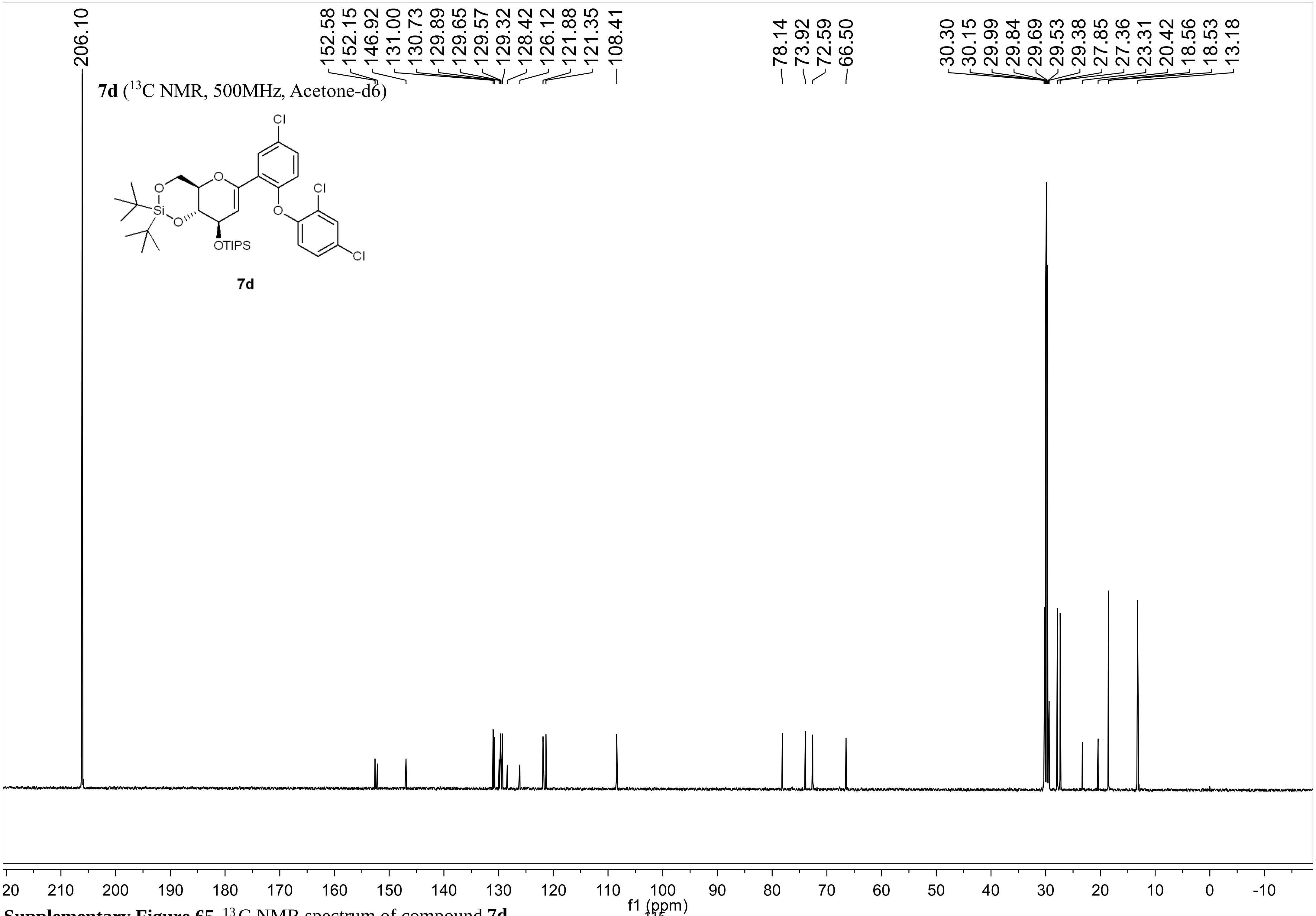

Supplementary Figure 65. ^{13}C NMR spectrum of compound **7d**

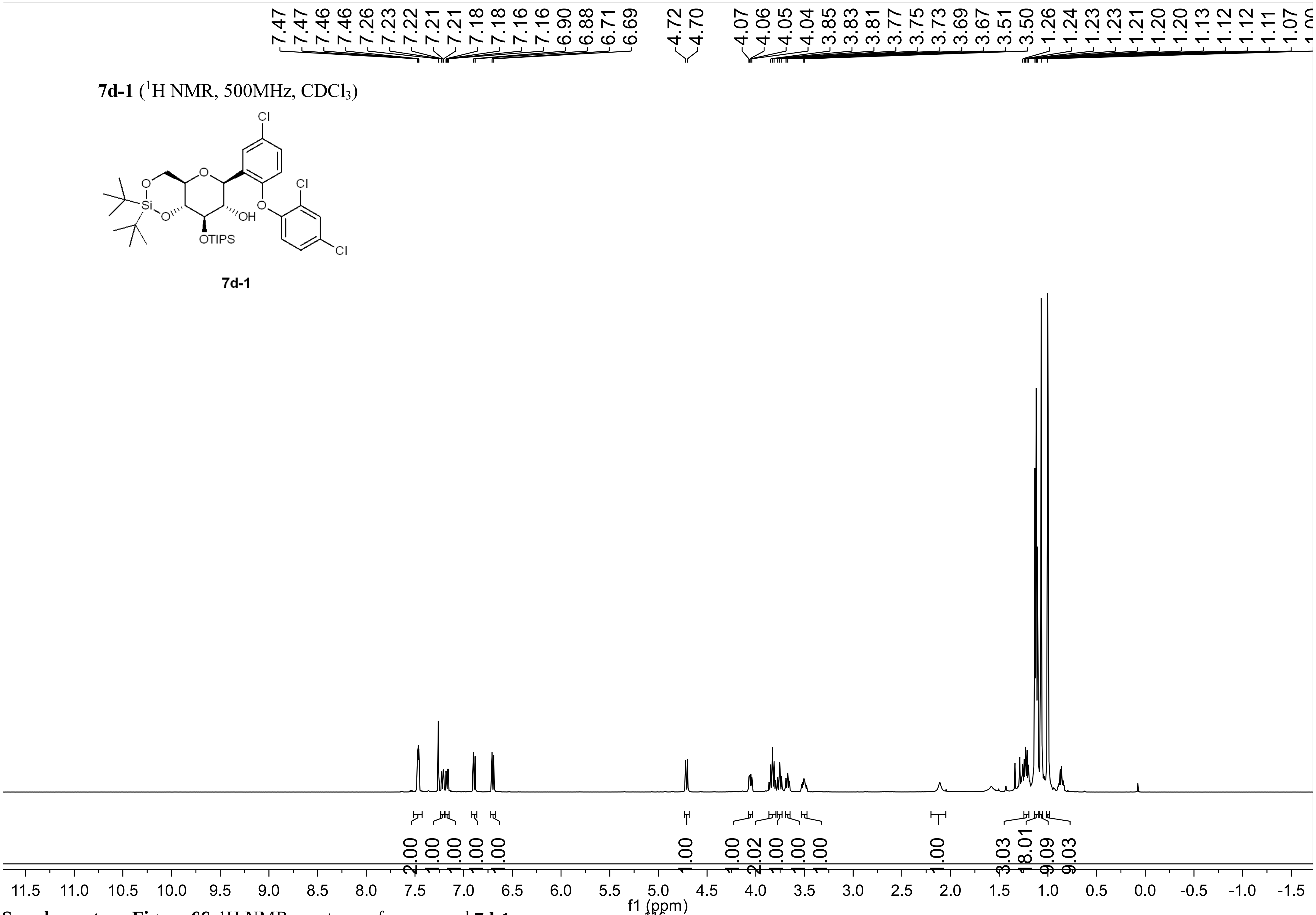

Supplementary Figure 66. ^{1}H NMR spectrum of compound **7d-1**

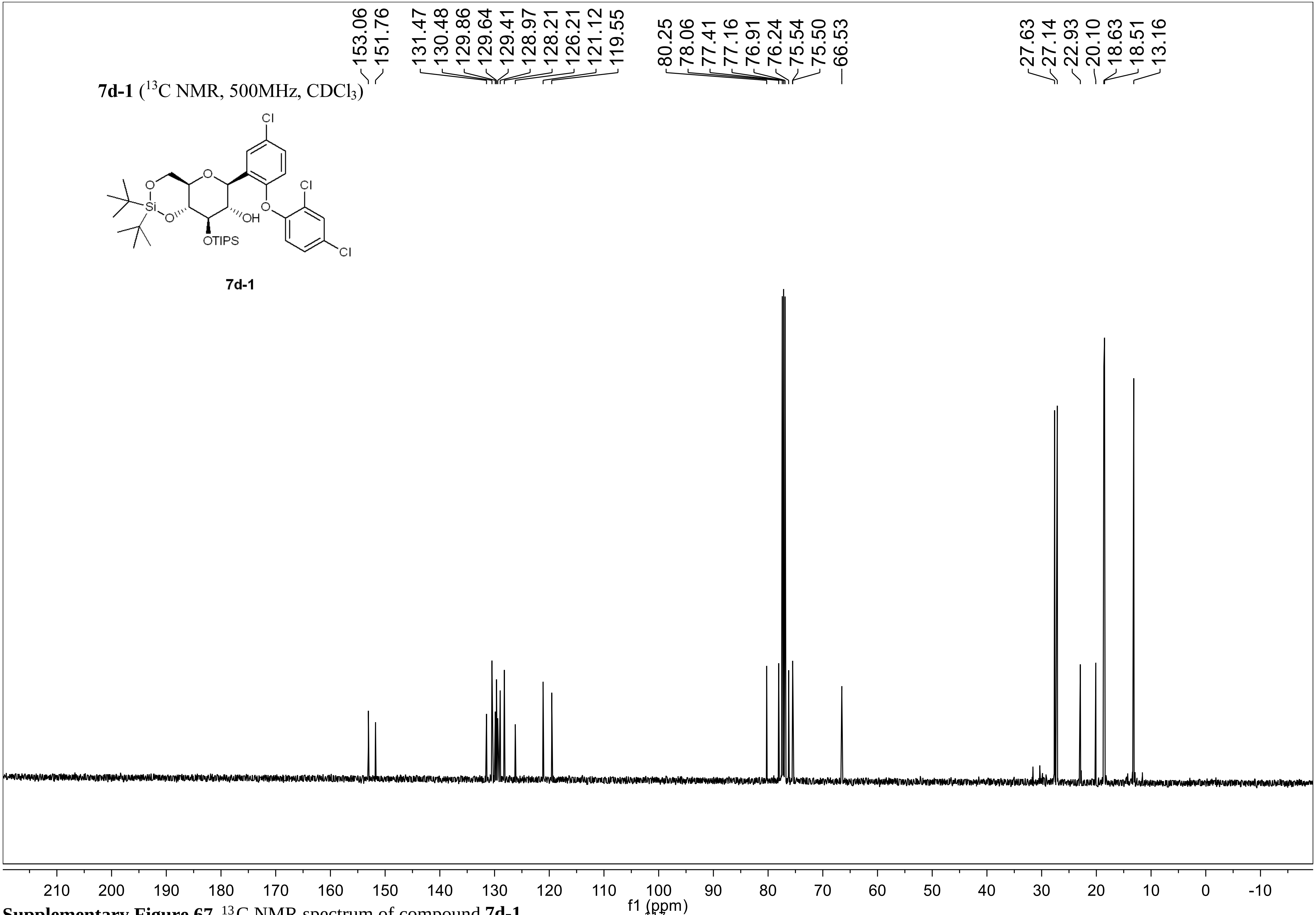

Supplementary Figure 67. ^{13}C NMR spectrum of compound **7d-1**

7d-1 (H-H COSY , 500MHz, $CDCl_3$)

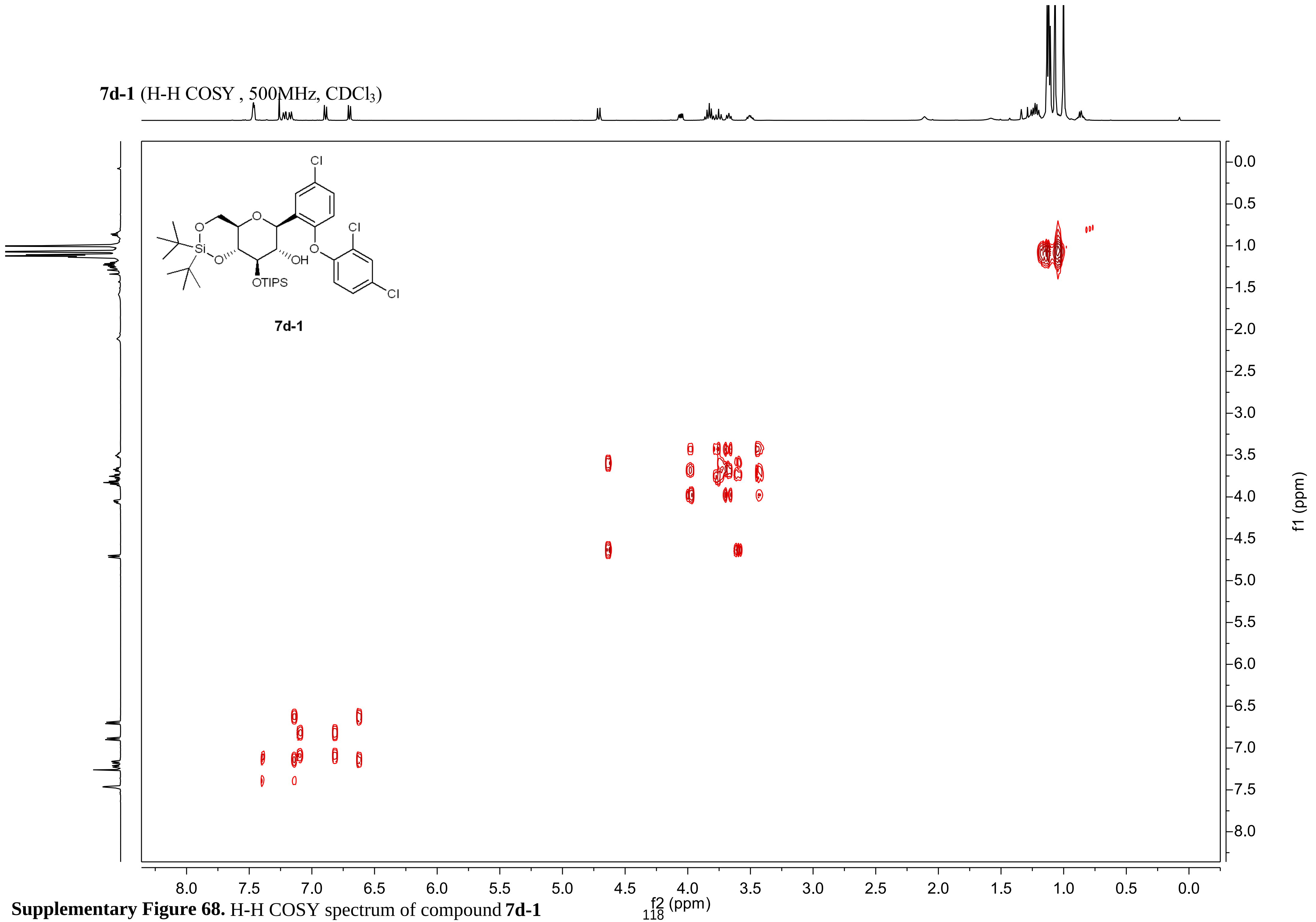

Supplementary Figure 68. H-H COSY spectrum of compound **7d-1**

7d-1 (coulped HSQC, 500MHz, $CDCl_3$)

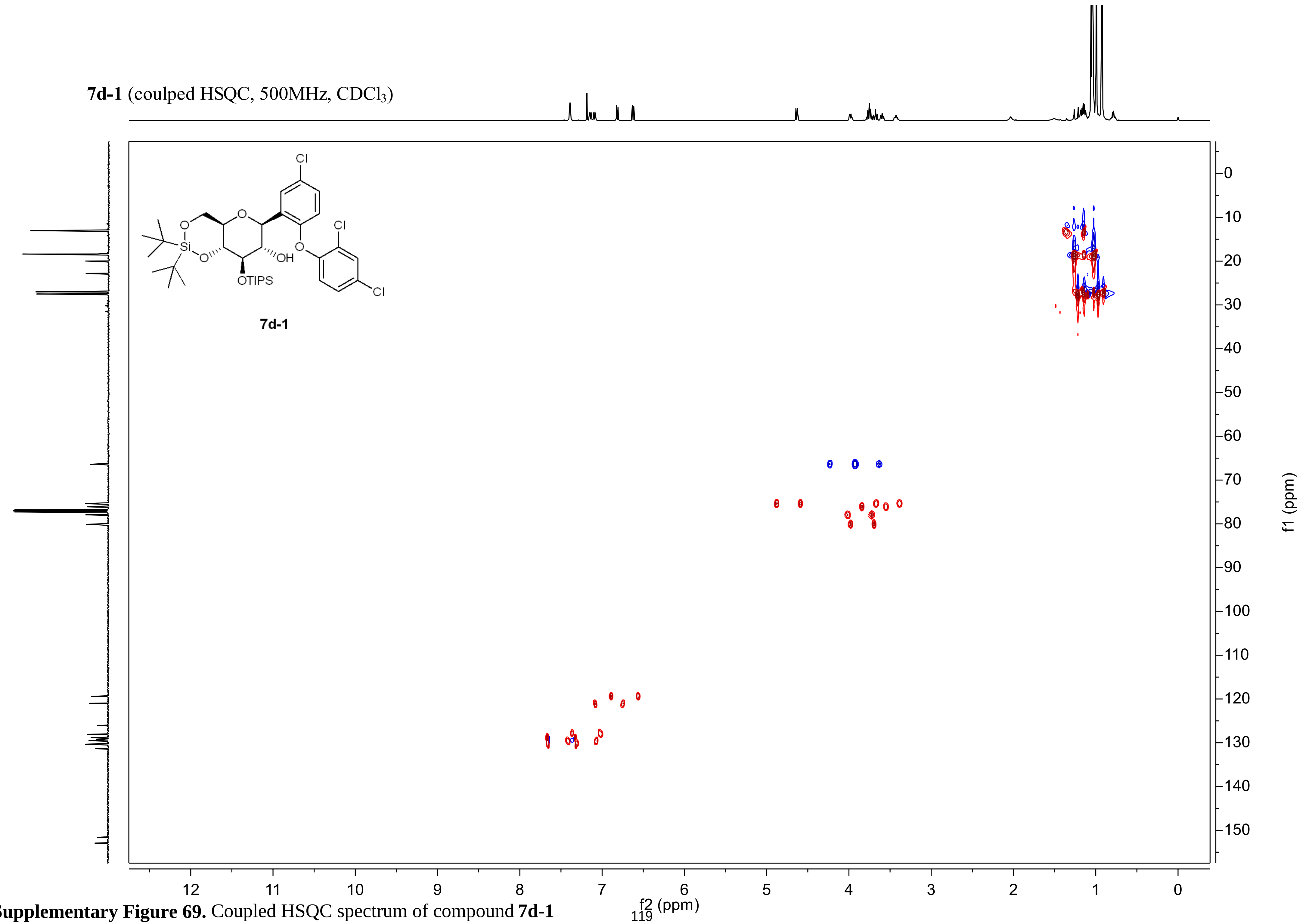

Supplementary Figure 69. Coupled HSQC spectrum of compound **7d-1**

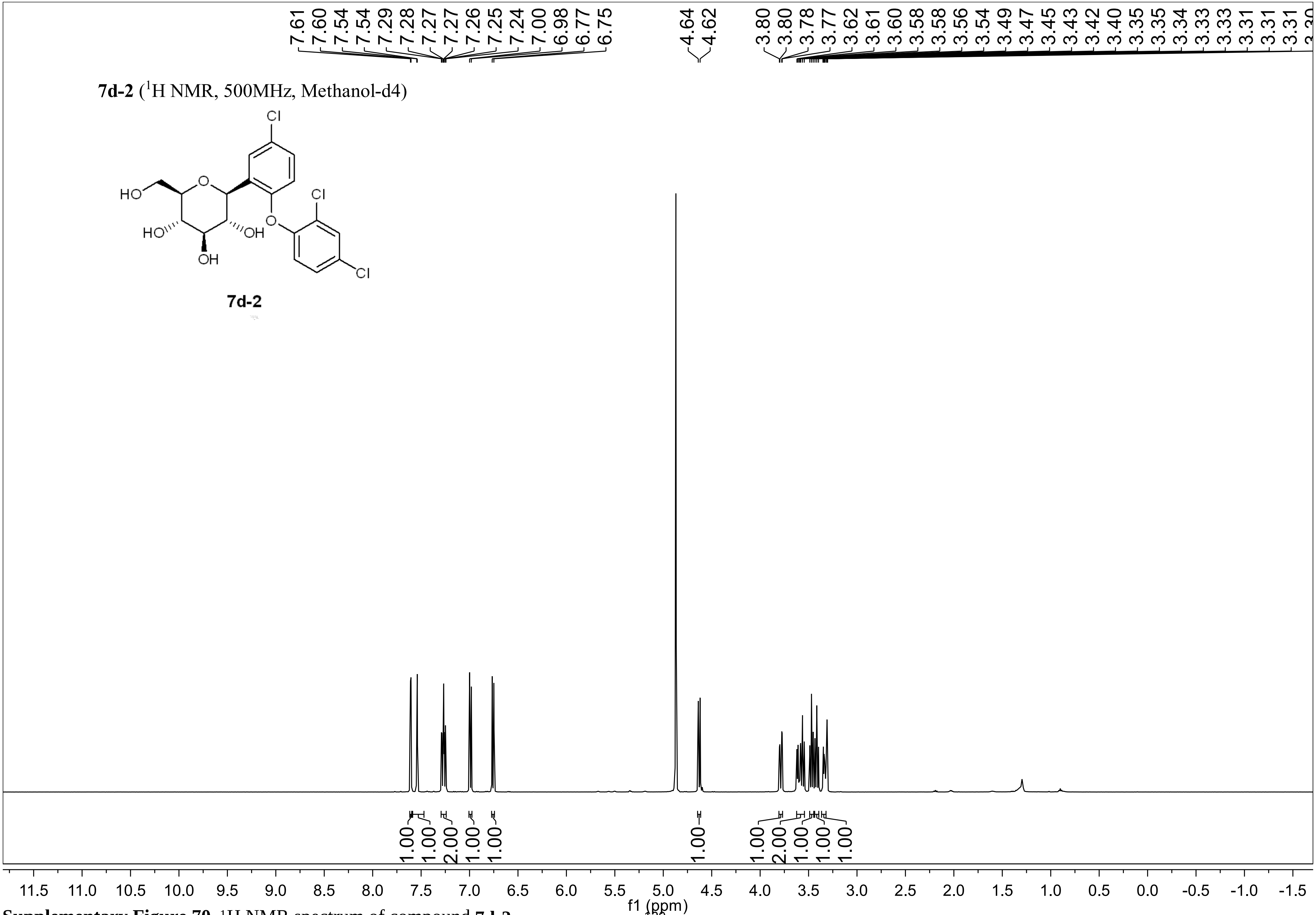

Supplementary Figure 70. ^{1}H NMR spectrum of compound **7d-2**

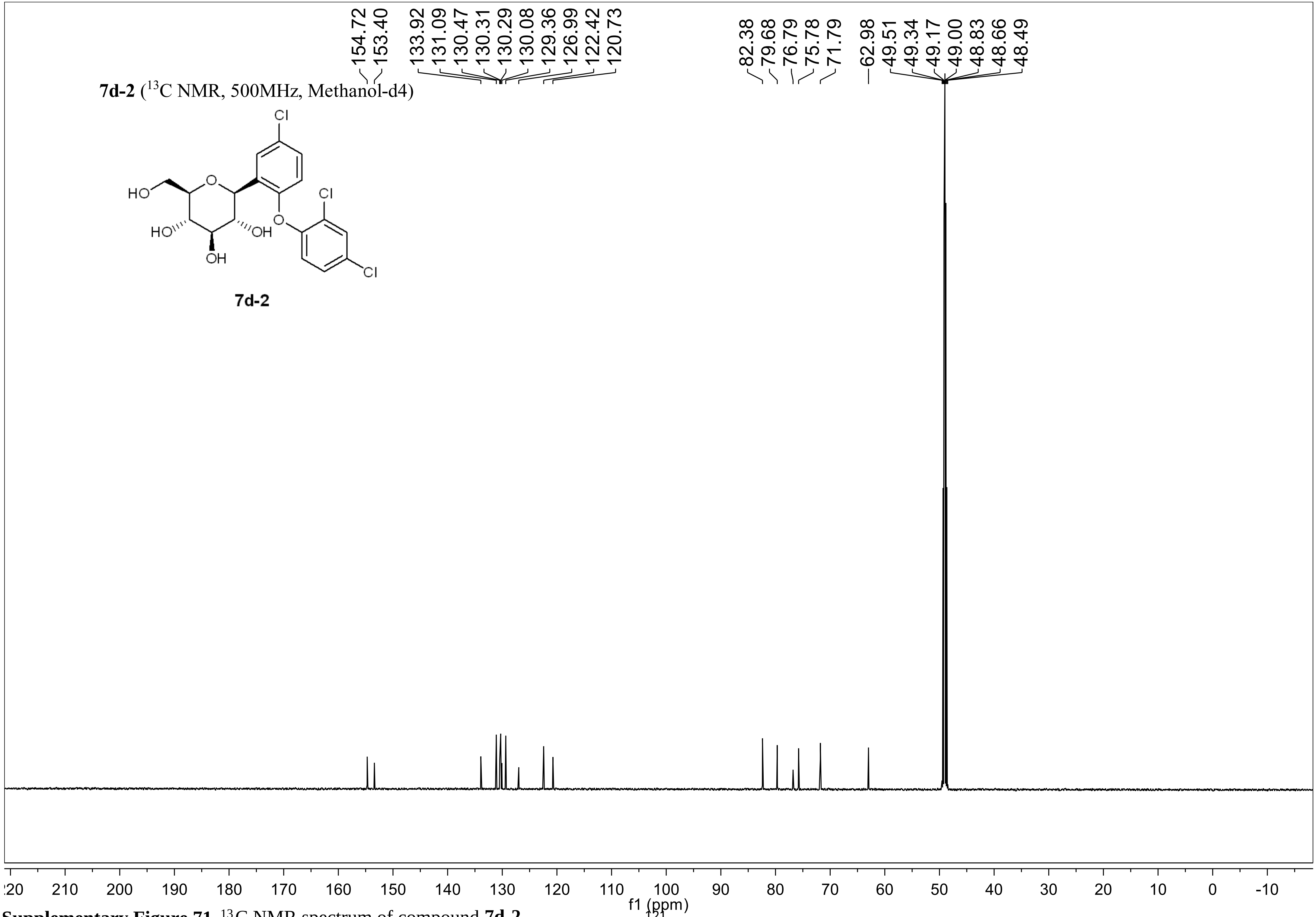

Supplementary Figure 71. ^{13}C NMR spectrum of compound **7d-2**

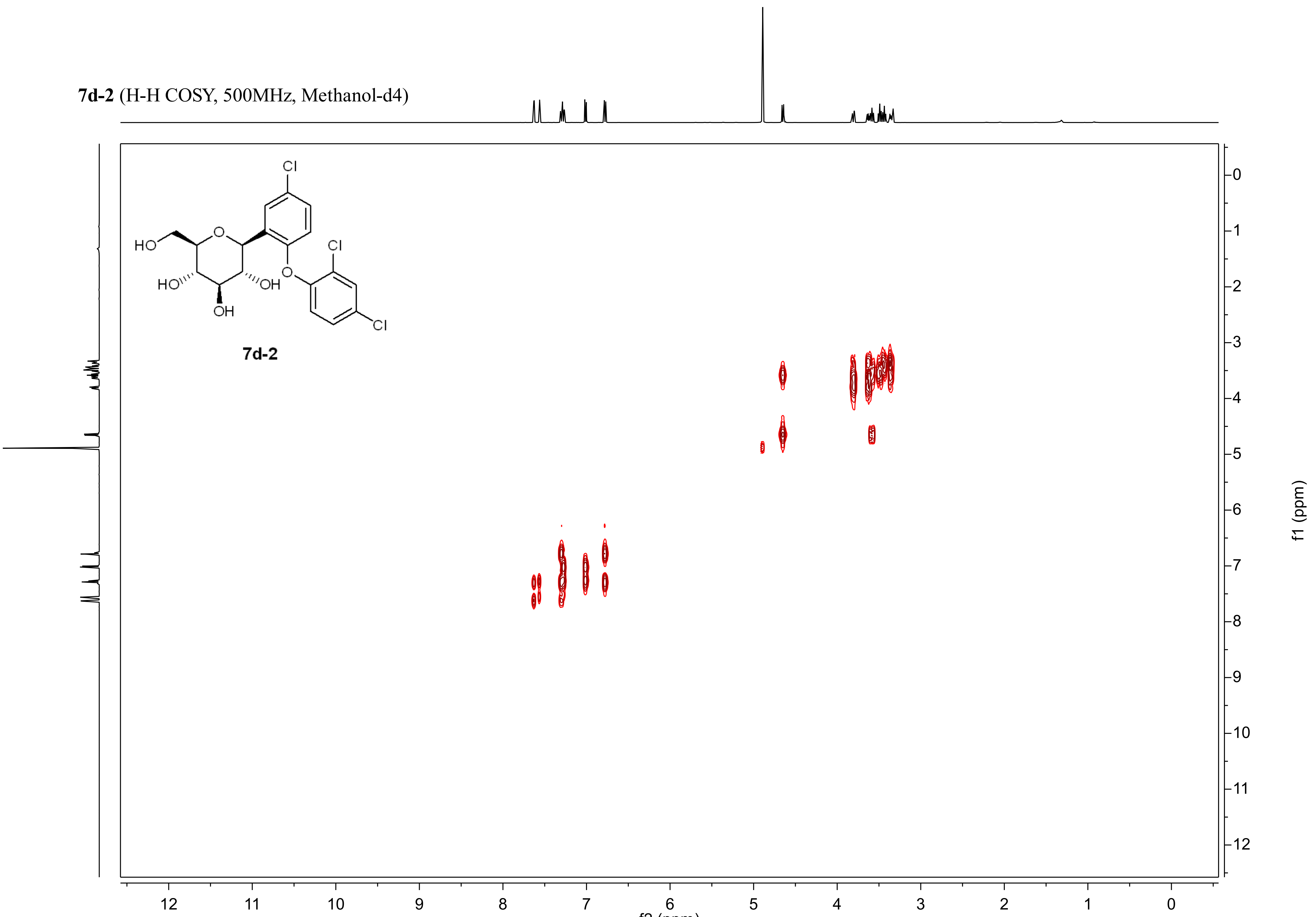

Supplementary Figure 72. H-H COSY spectrum of compound **7d-2**

7d-2 (coulped HSQC, 500MHz, Methanol-d4)

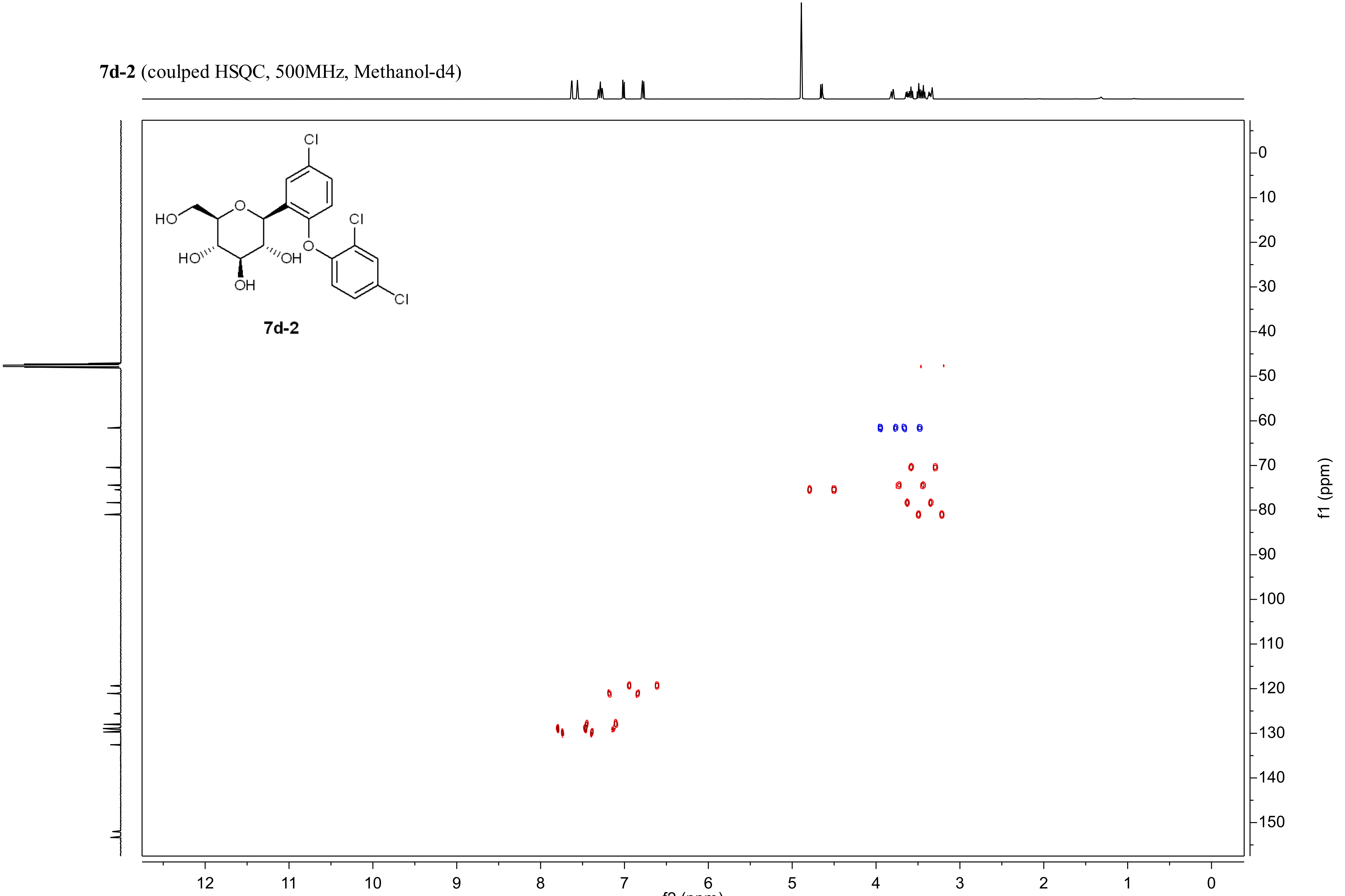

Supplementary Figure 73. Coupled HSQC spectrum of compound **7d-2**

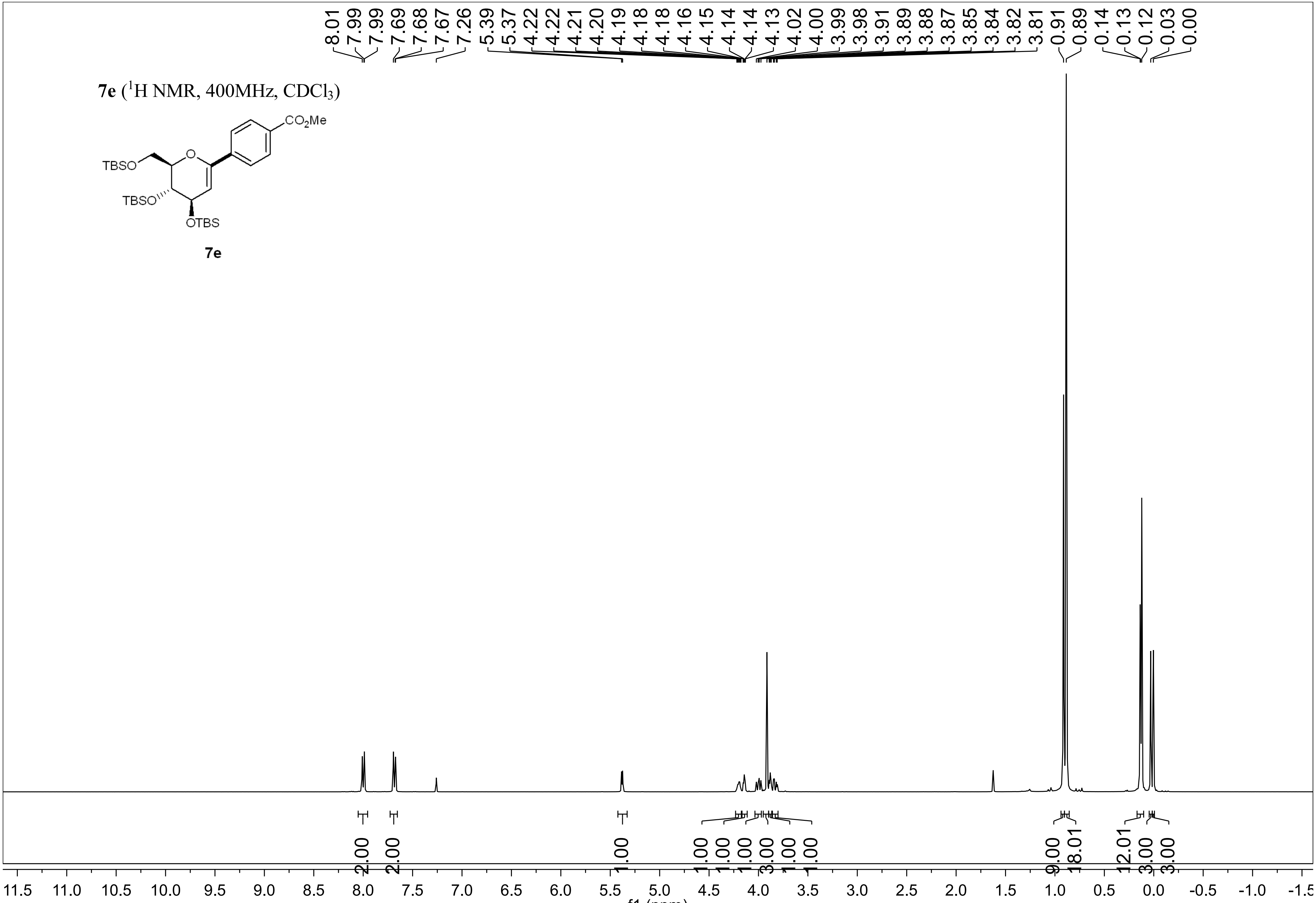

Supplementary Figure 74. ^{1}H NMR spectrum of compound **7e**

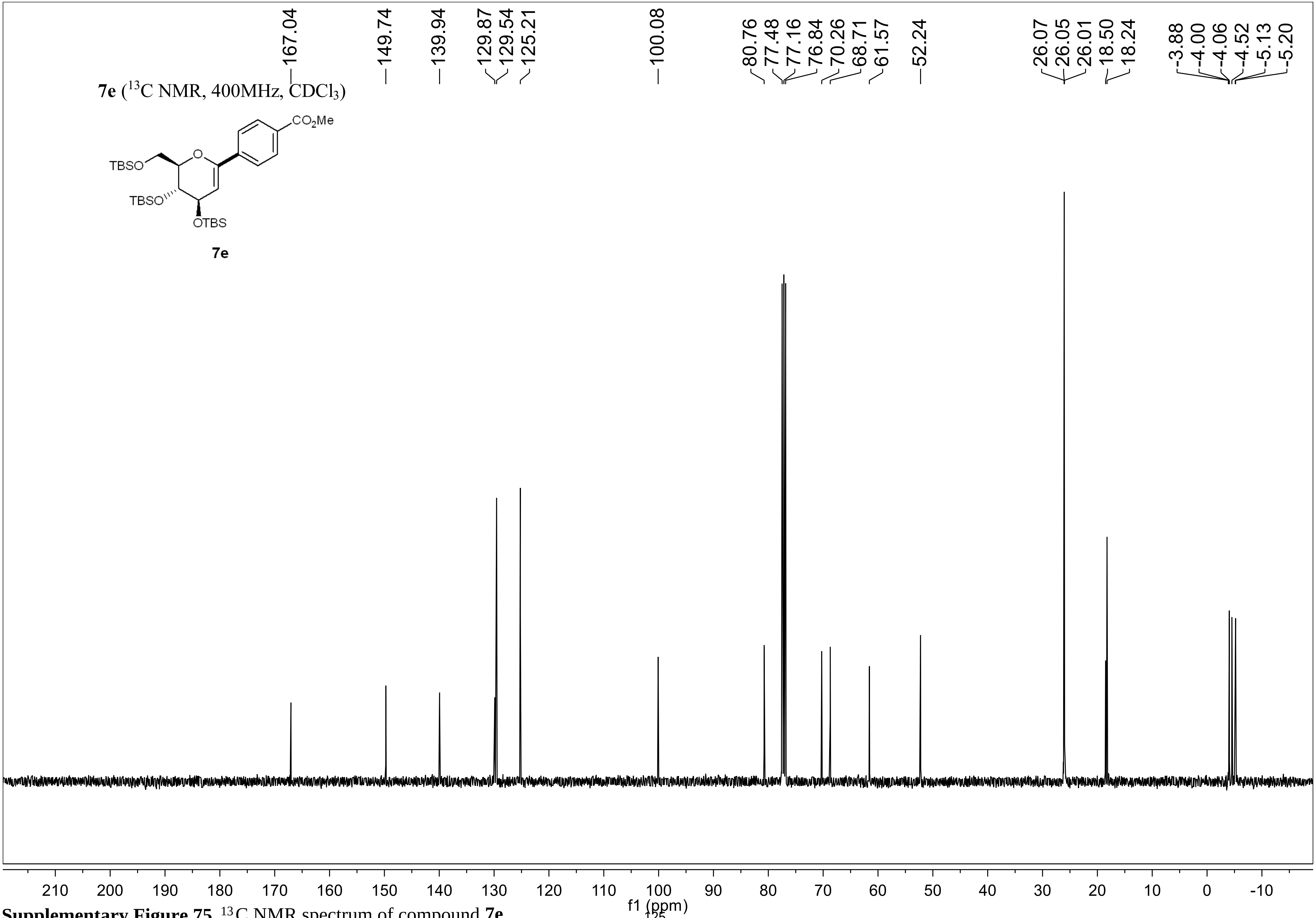

Supplementary Figure 75. ^{13}C NMR spectrum of compound **7e**

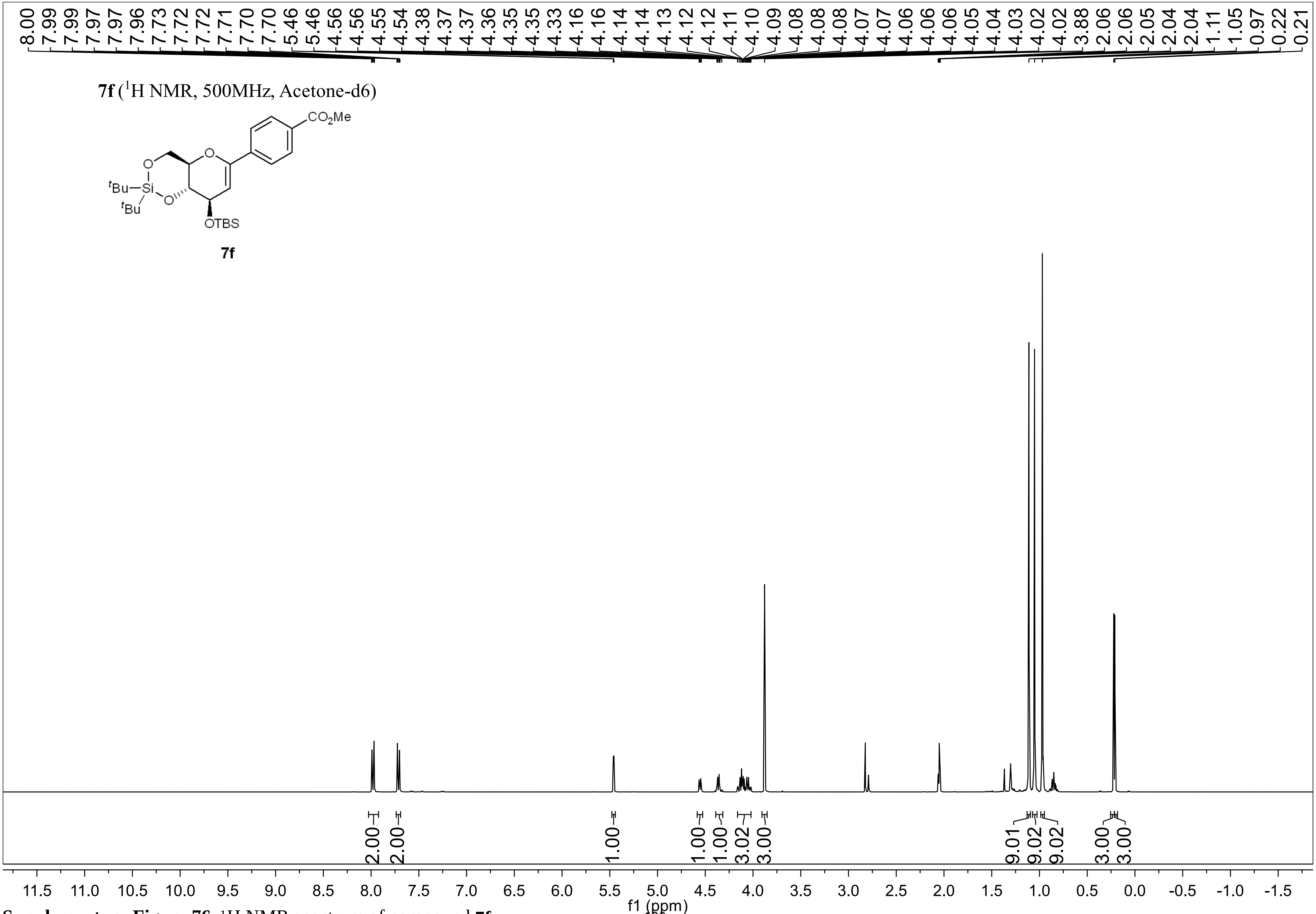

Supplementary Figure 76. ^{1}H NMR spectrum of compound **7f**

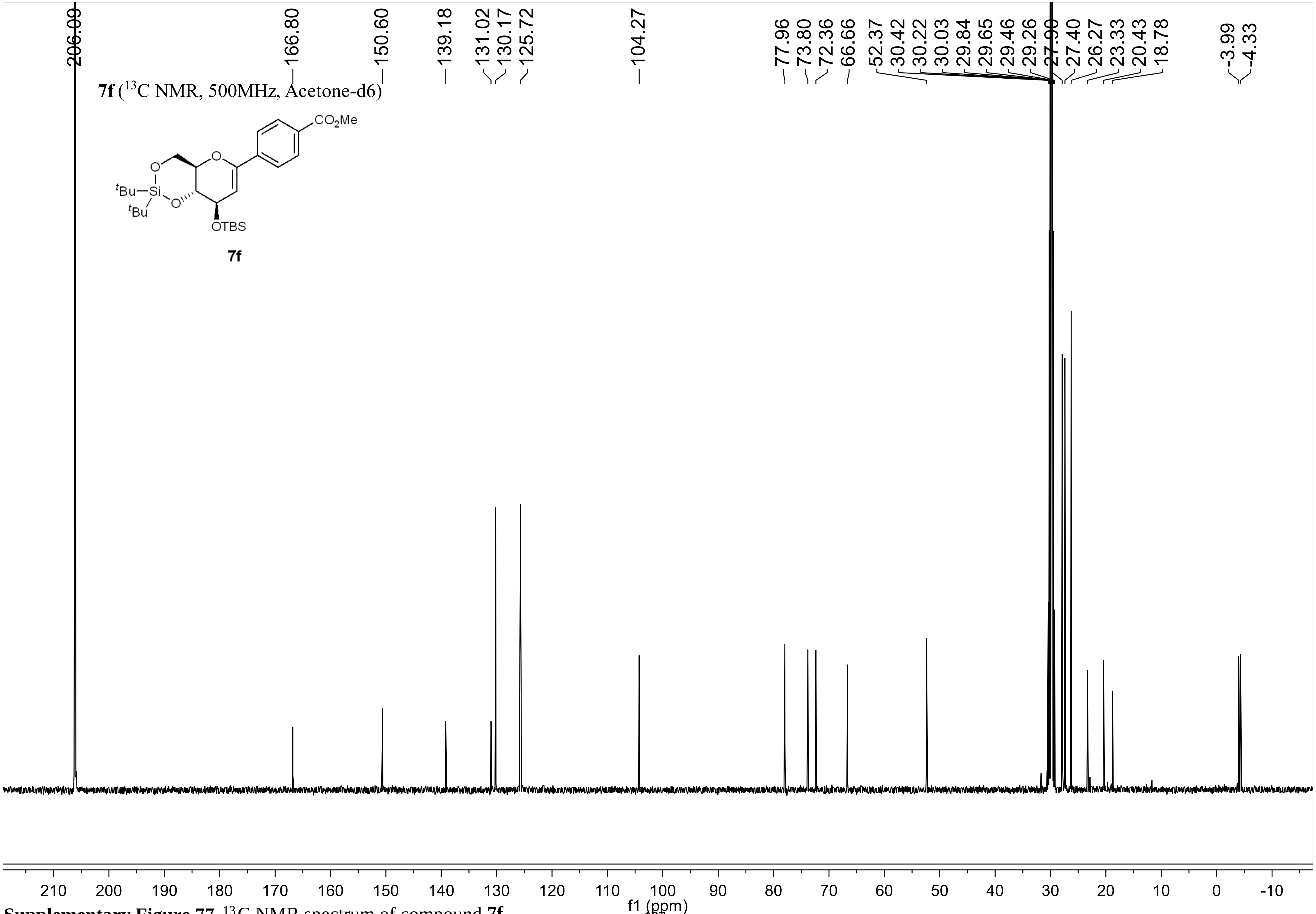

Supplementary Figure 77. ^{13}C NMR spectrum of compound **7f**

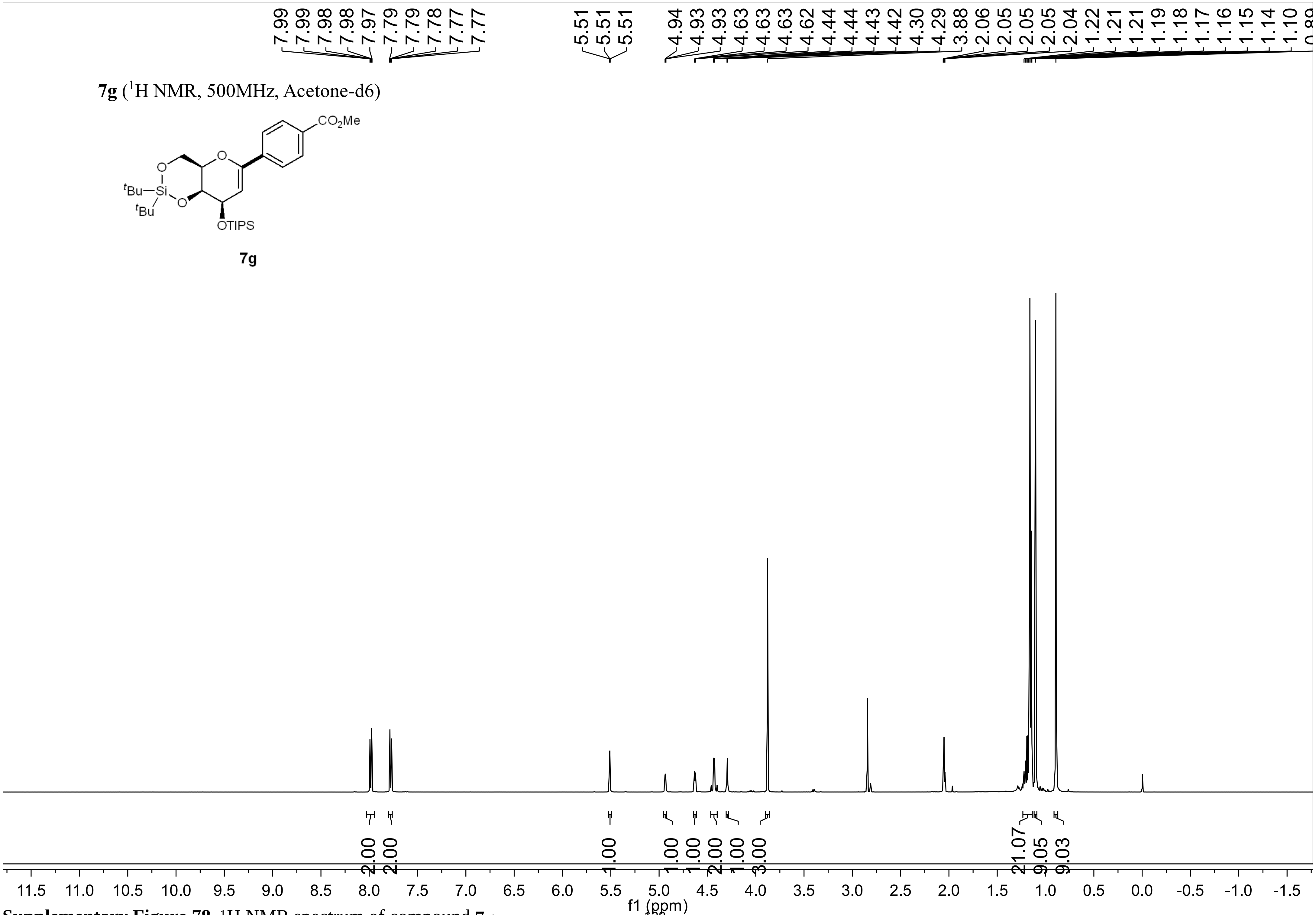

Supplementary Figure 78. ^{1}H NMR spectrum of compound **7g**

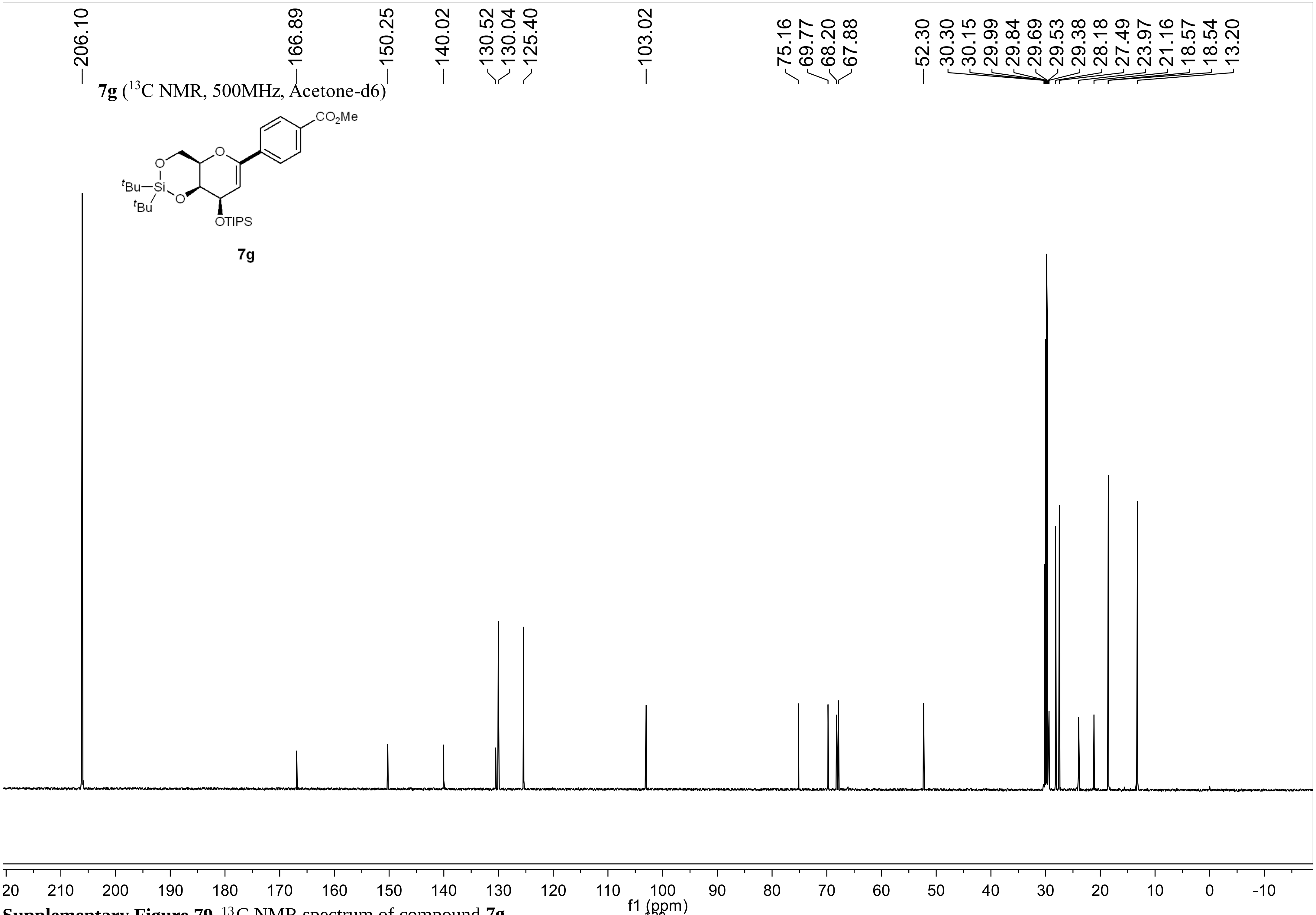

Supplementary Figure 79. ^{13}C NMR spectrum of compound **7g**

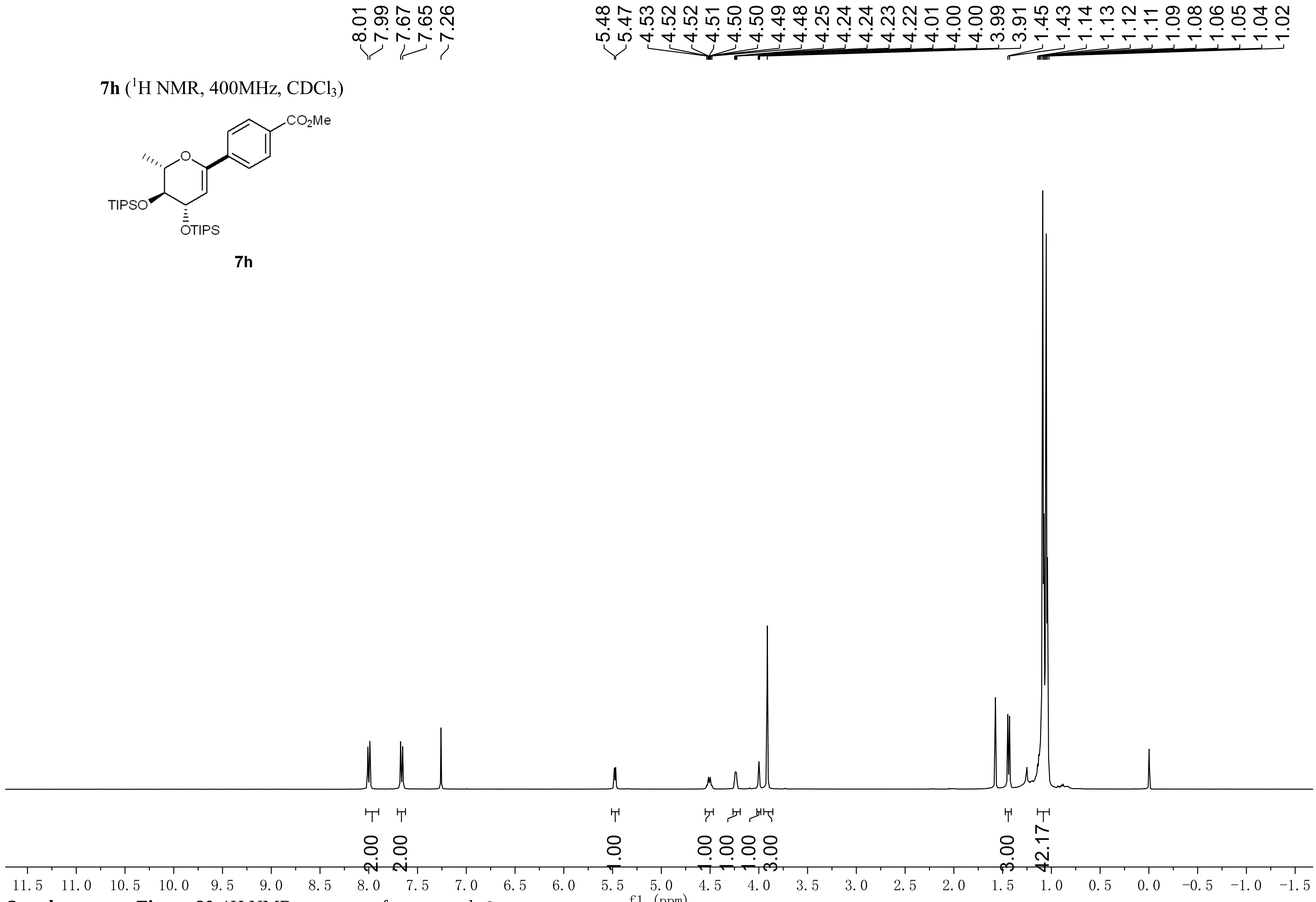

Supplementary Figure 80. ^{1}H NMR spectrum of compound **7h**

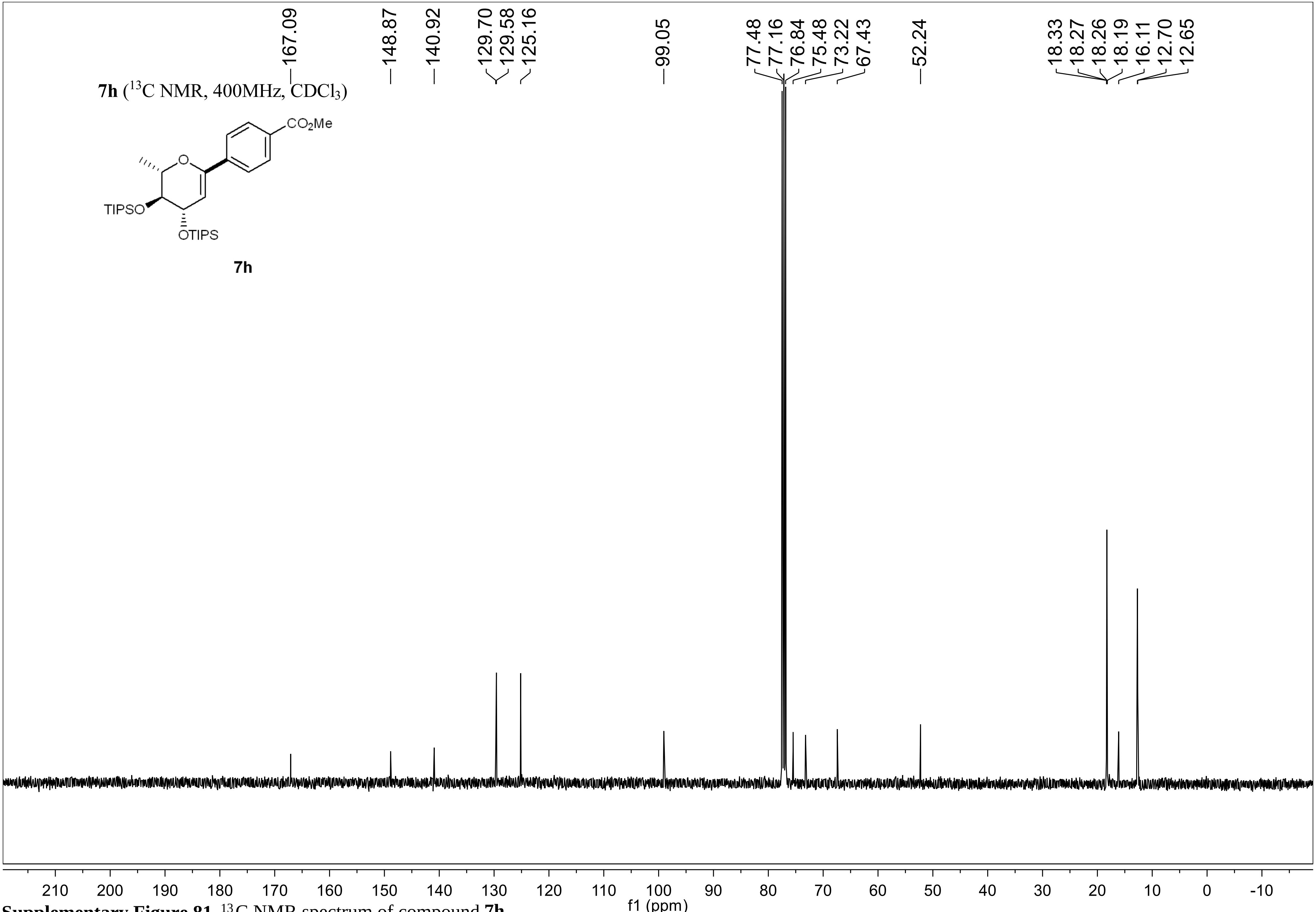

Supplementary Figure 81. ^{13}C NMR spectrum of compound **7h**

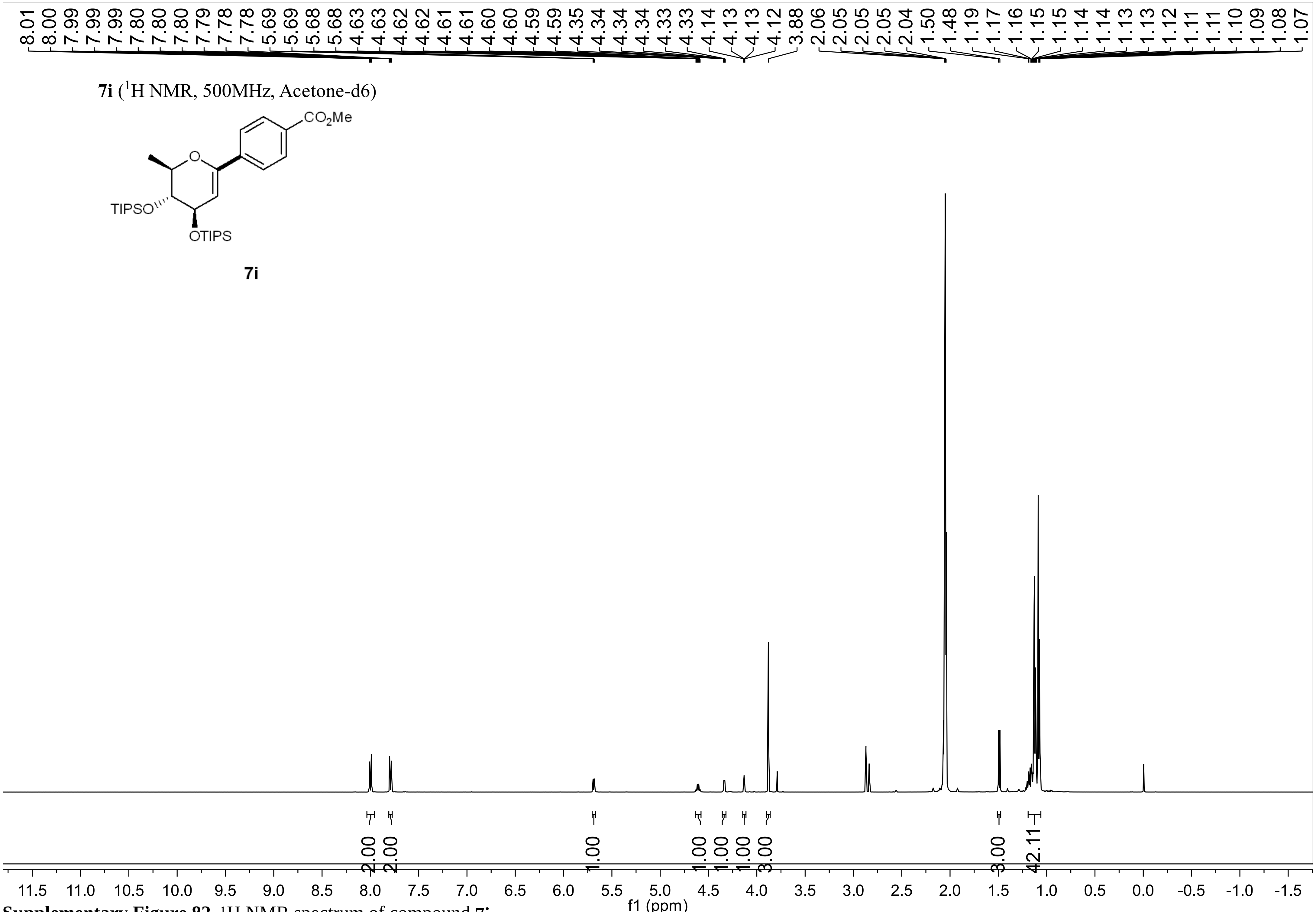

Supplementary Figure 82. ^{1}H NMR spectrum of compound **7i**

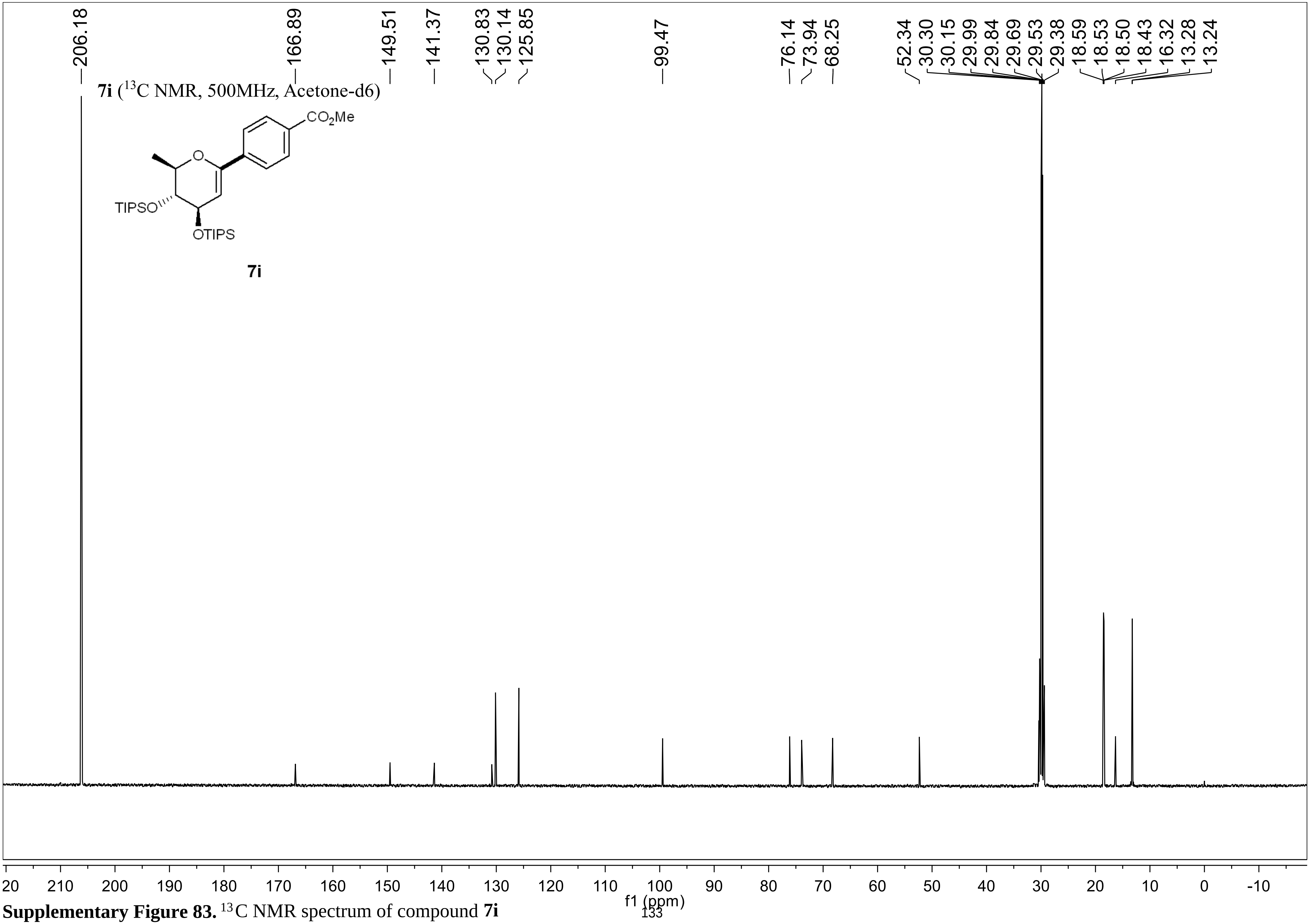

Supplementary Figure 83. ^{13}C NMR spectrum of compound **7i**

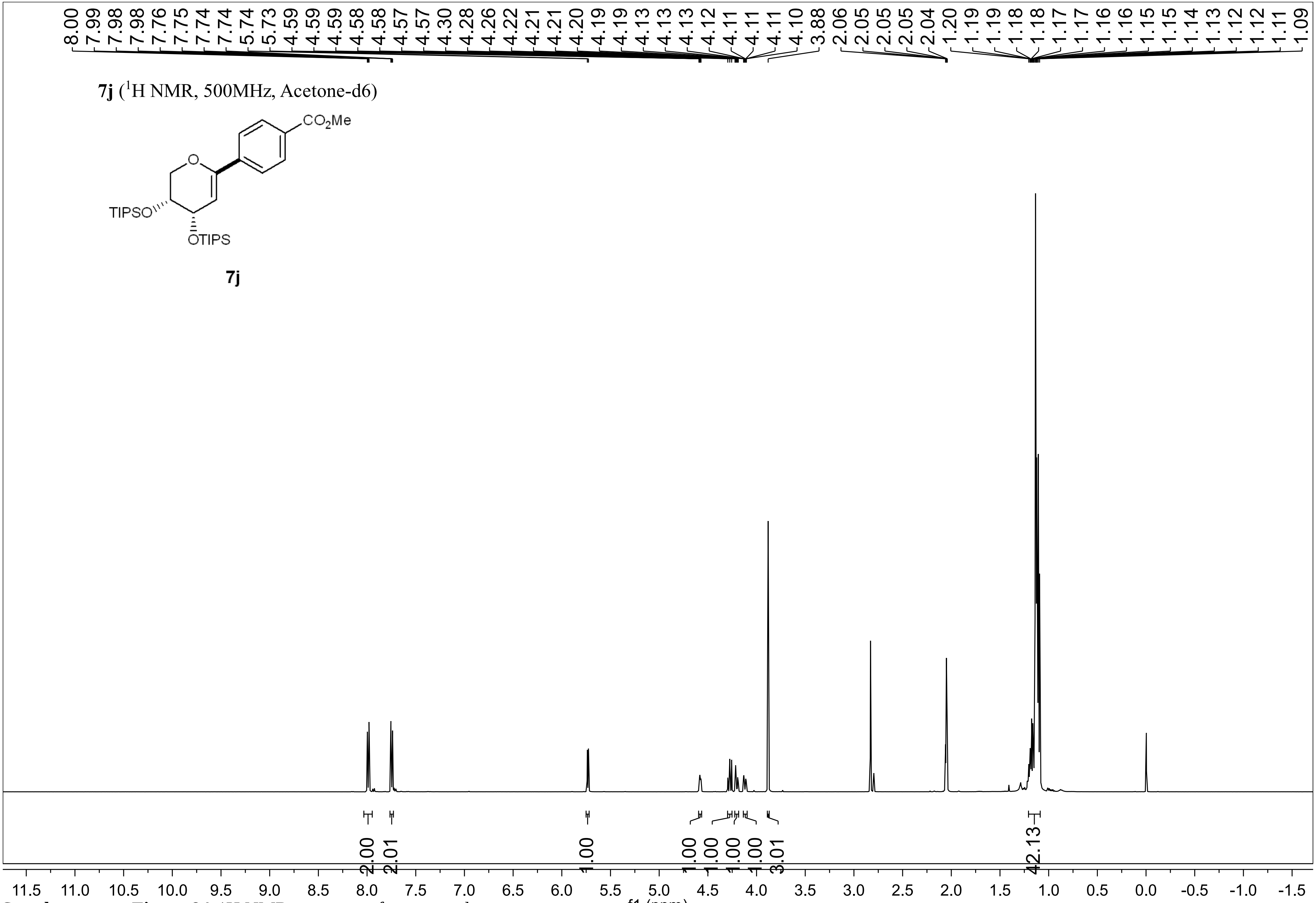

Supplementary Figure 84. ^{1}H NMR spectrum of compound **7j**

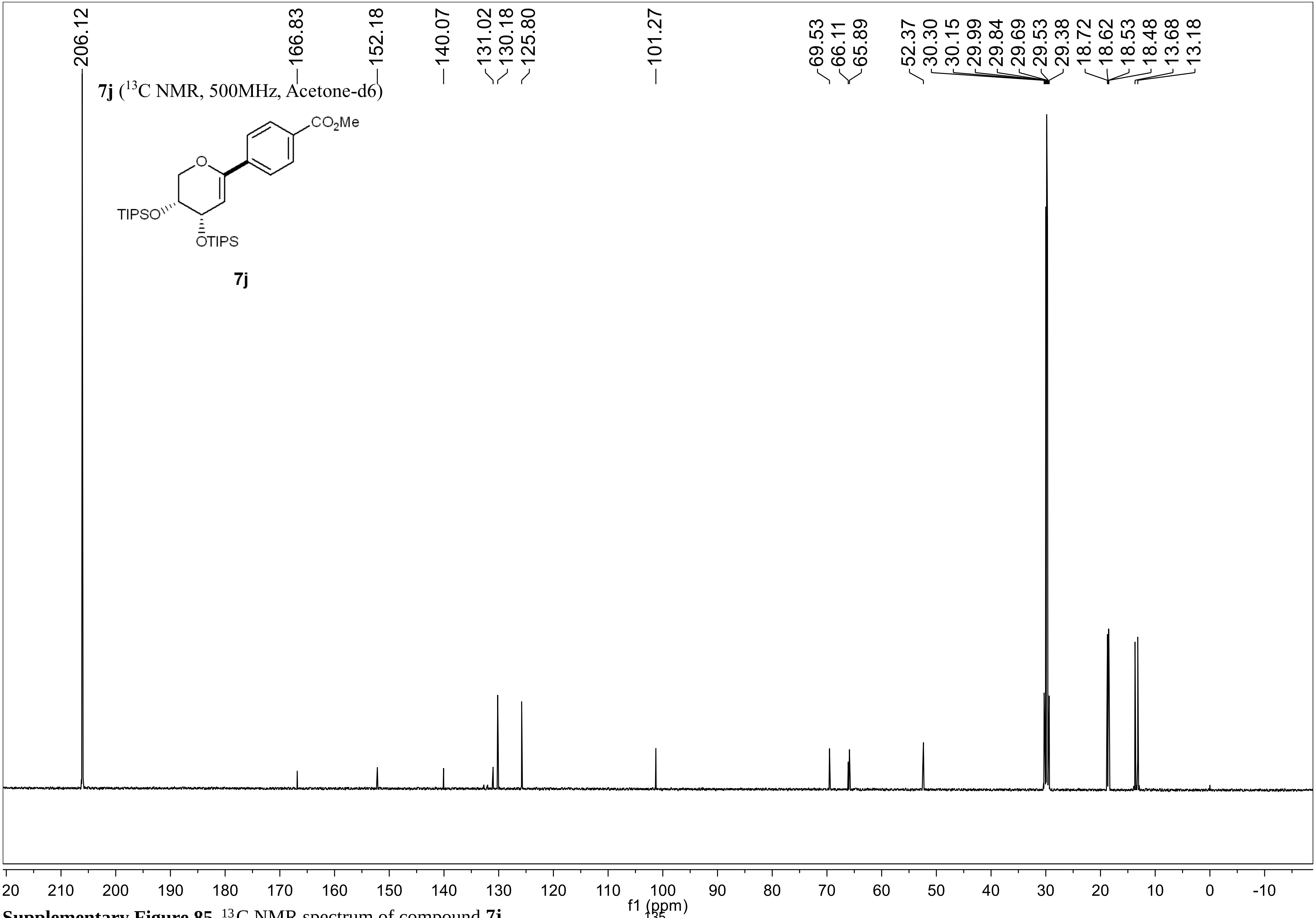

Supplementary Figure 85. ^{13}C NMR spectrum of compound **7j**

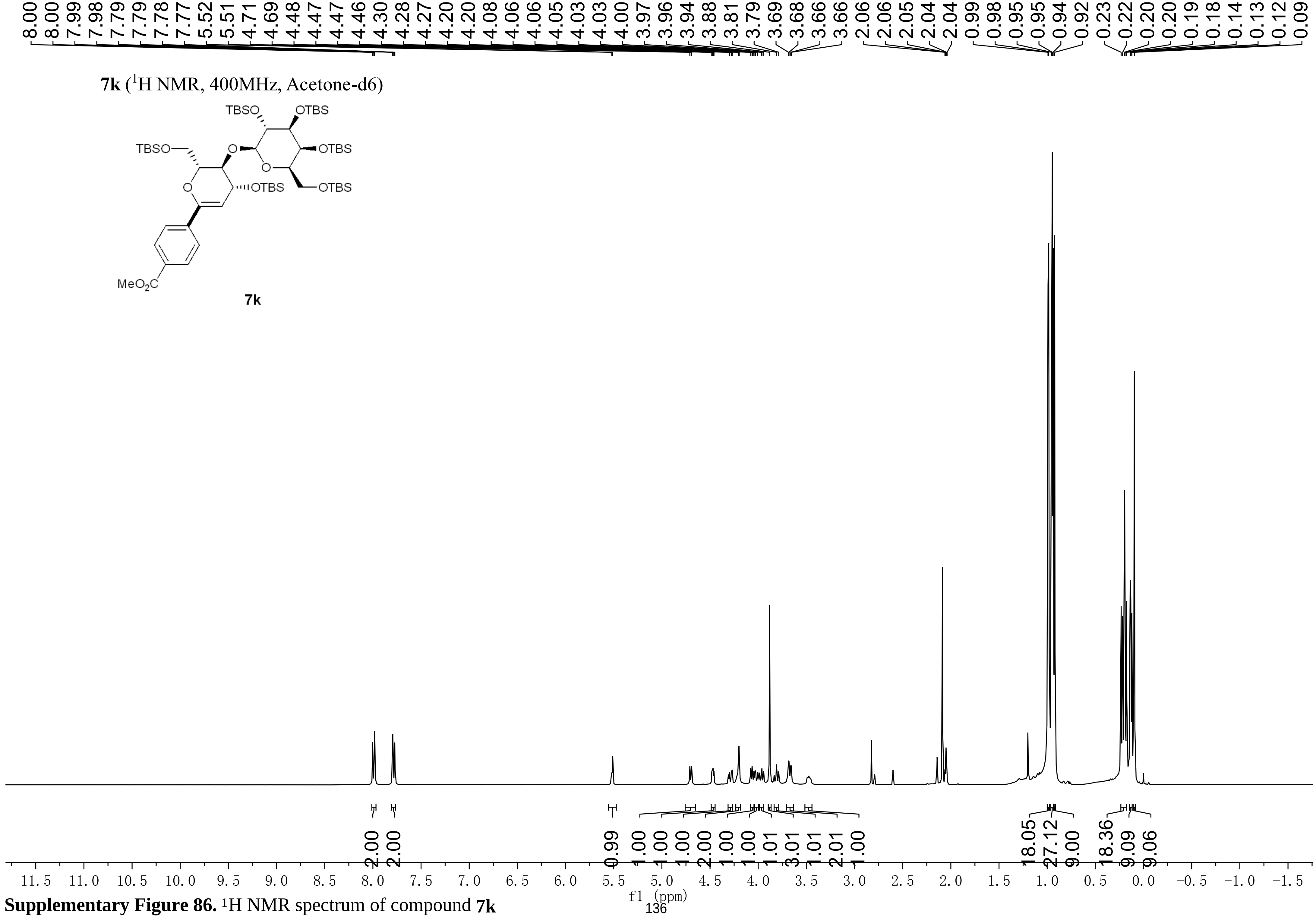

Supplementary Figure 86. ^{1}H NMR spectrum of compound **7k**

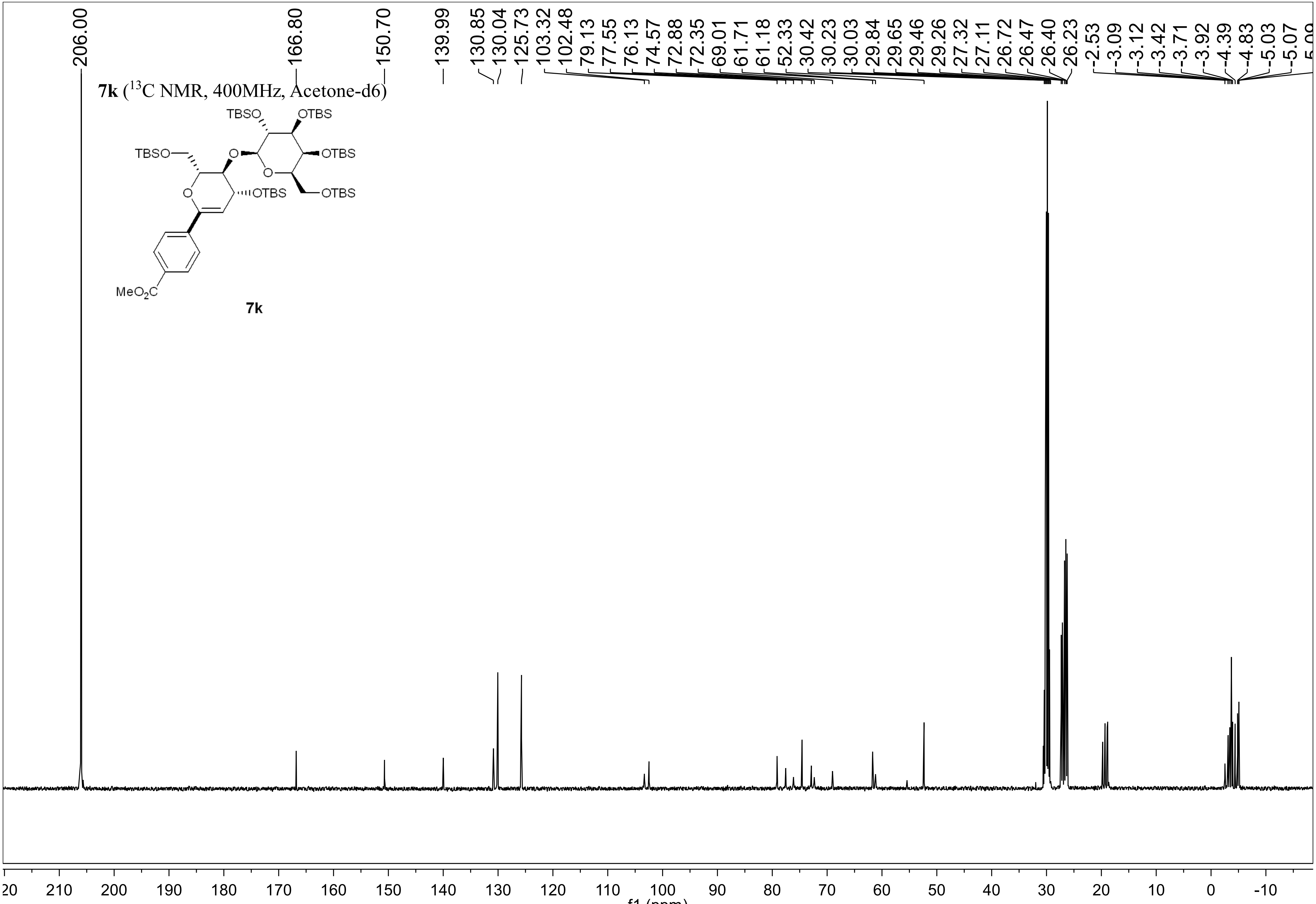

Supplementary Figure 87. ^{13}C NMR spectrum of compound **7k**

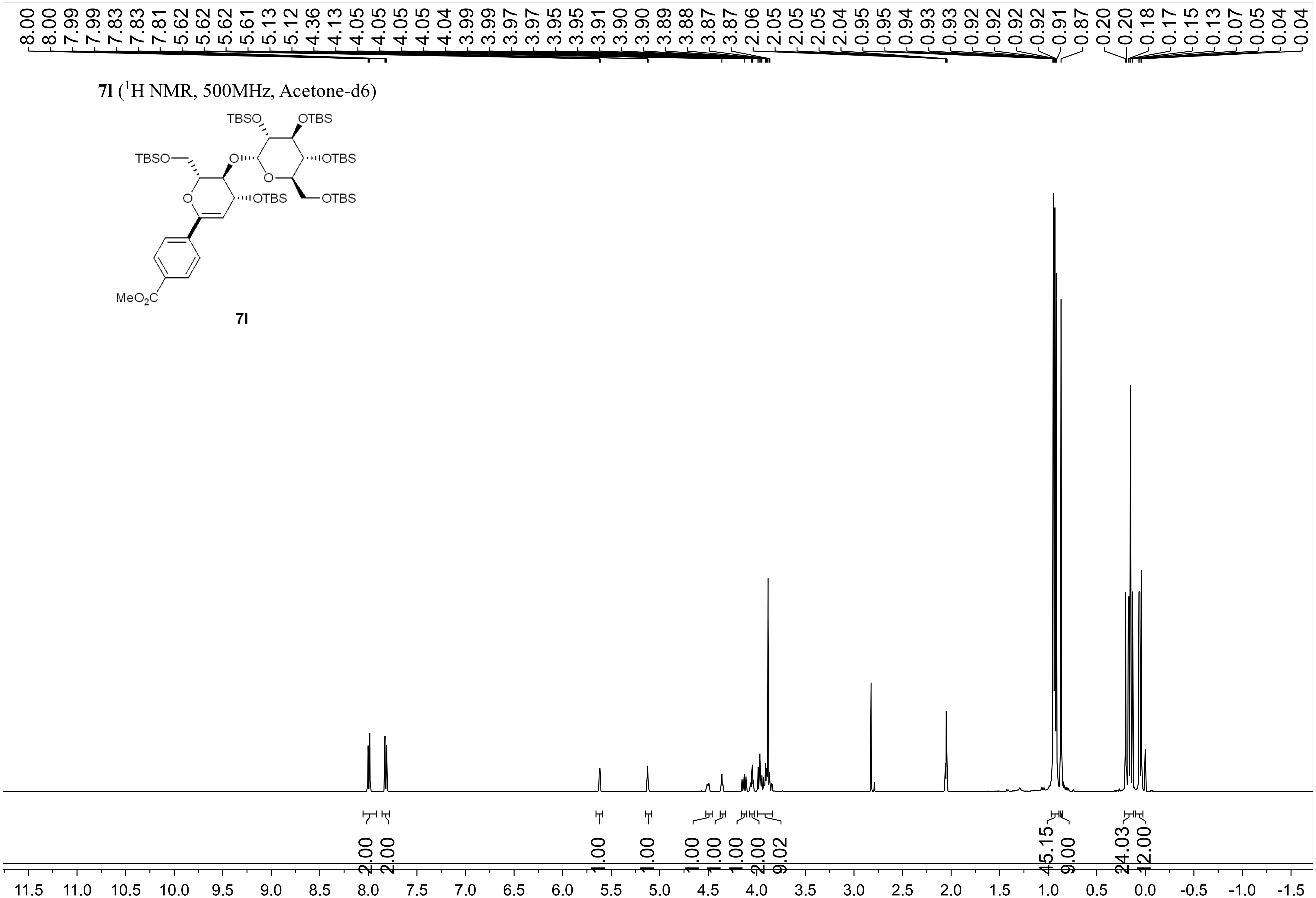

Supplementary Figure 88. ^{1}H NMR spectrum of compound **7l**

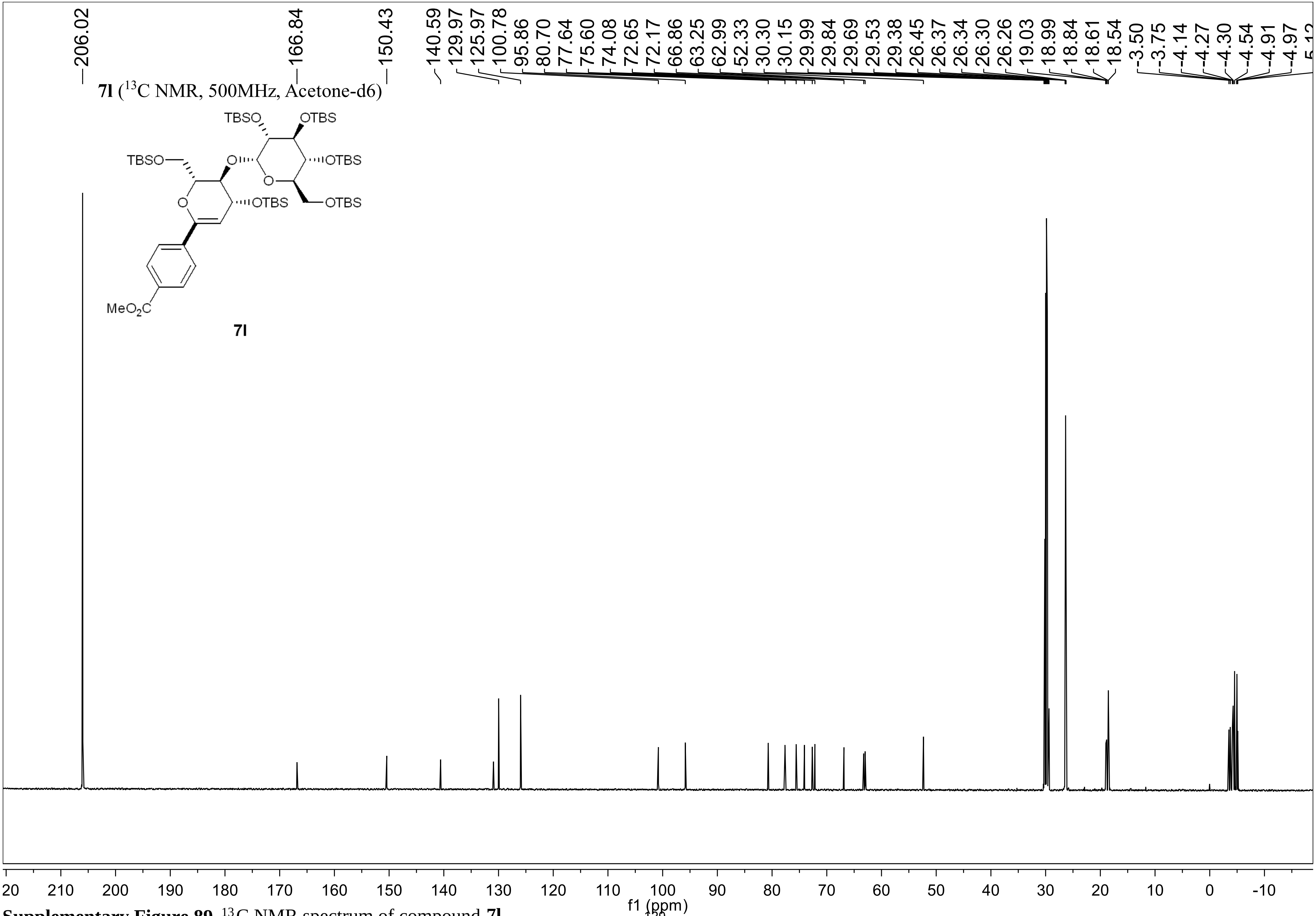

Supplementary Figure 89. ^{13}C NMR spectrum of compound **7l**

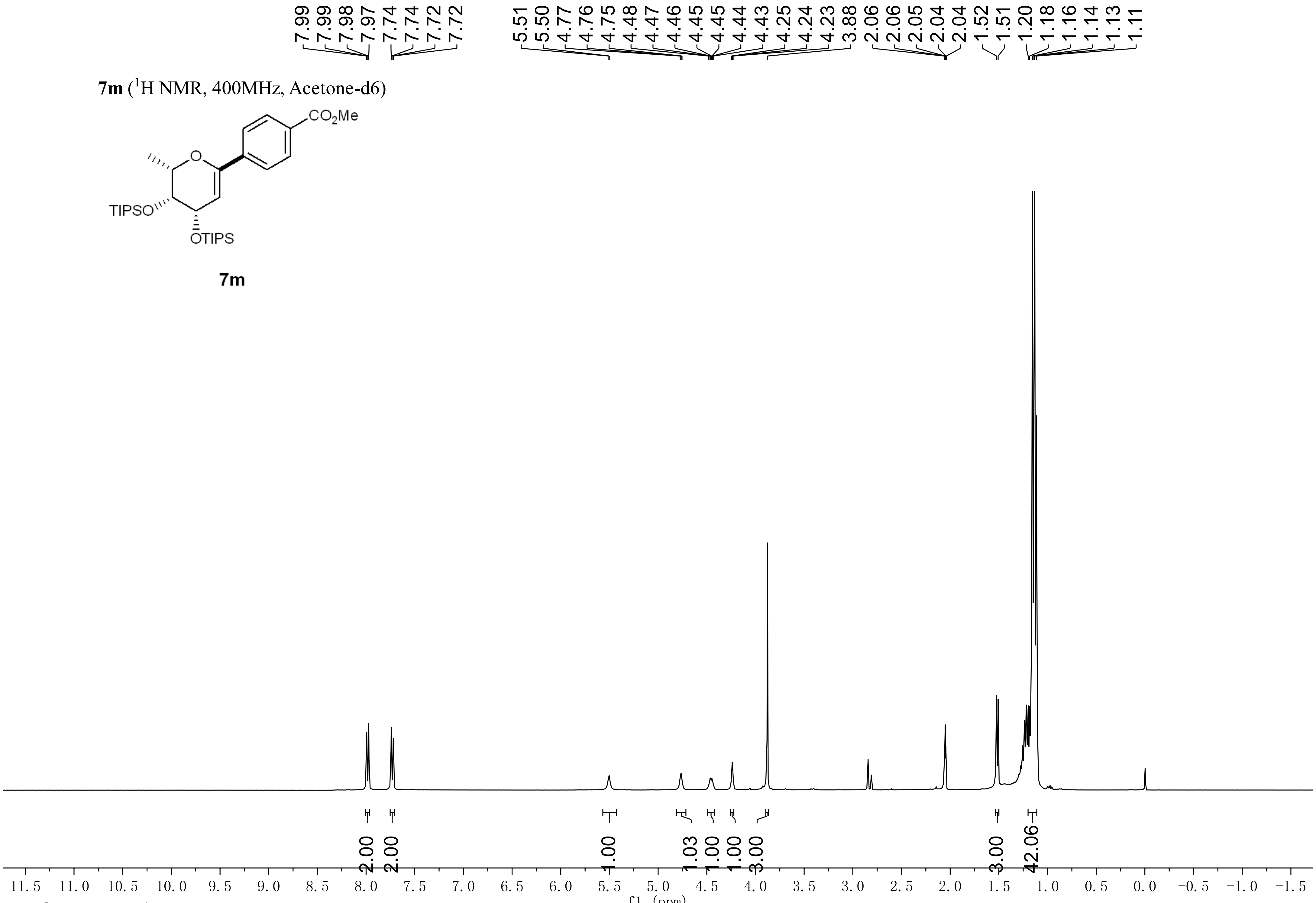

Supplementary Figure 90. ^{1}H NMR spectrum of compound **7m**

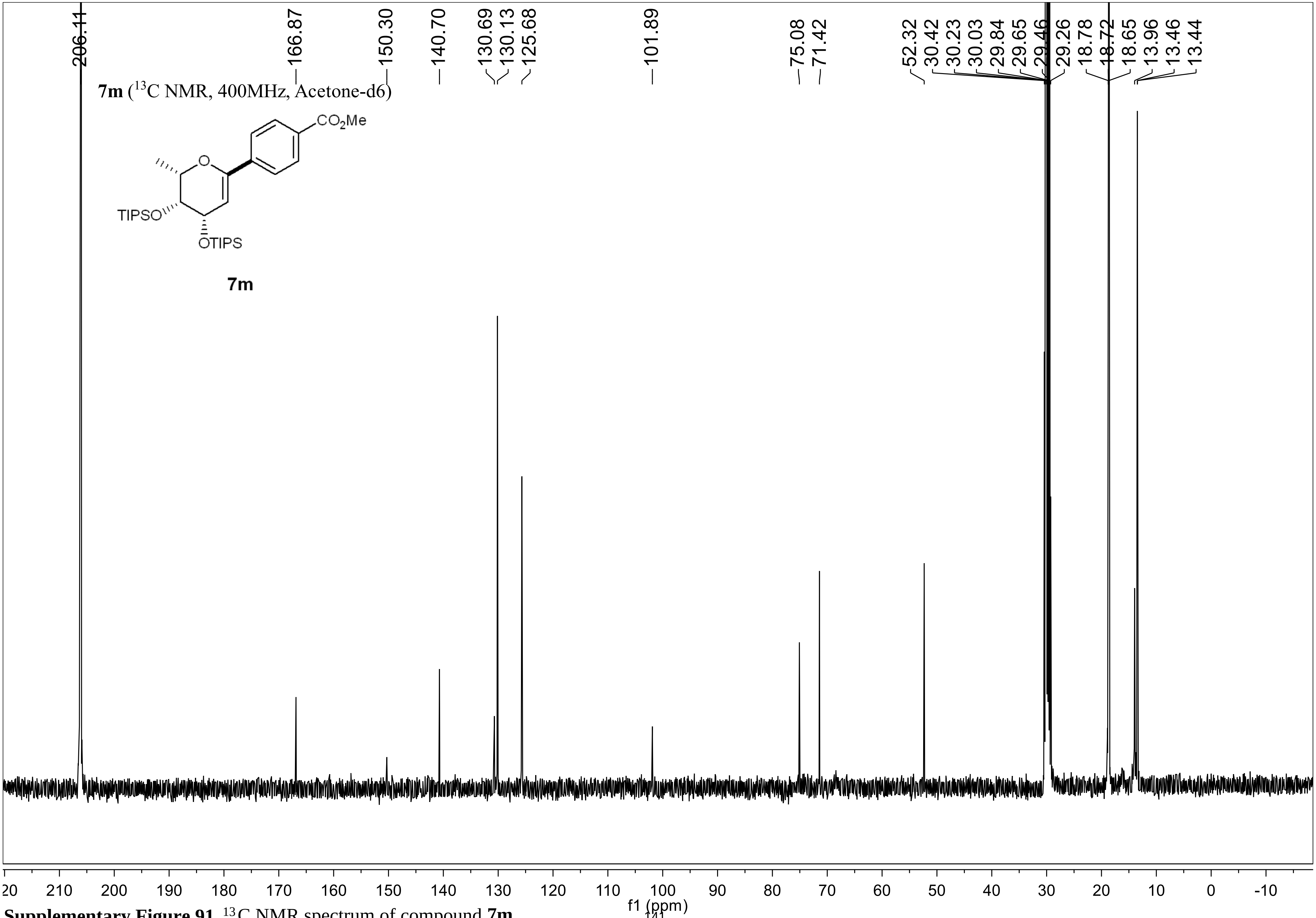

Supplementary Figure 91. ^{13}C NMR spectrum of compound **7m**

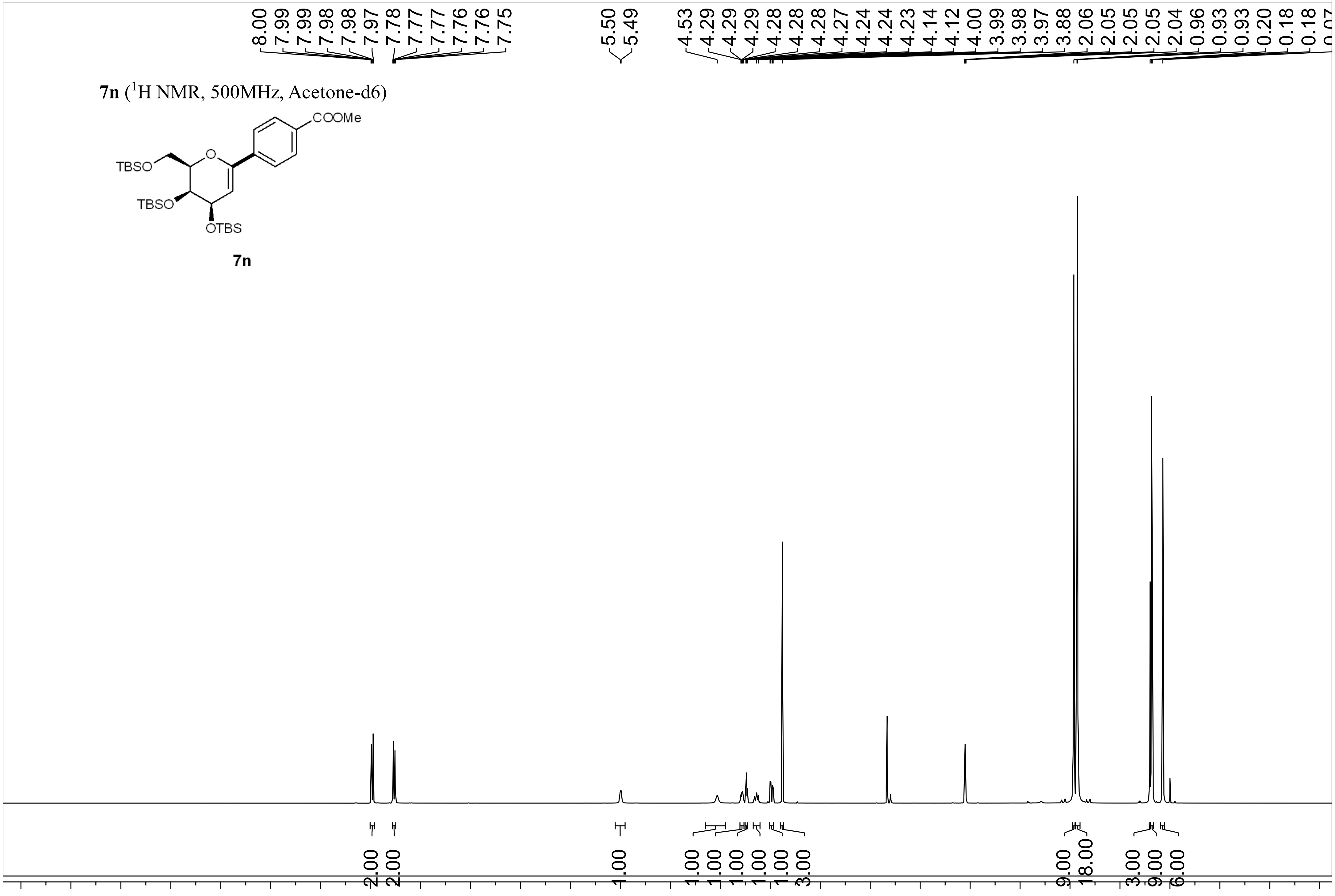

Supplementary Figure 92. ^{1}H NMR spectrum of compound **7n**

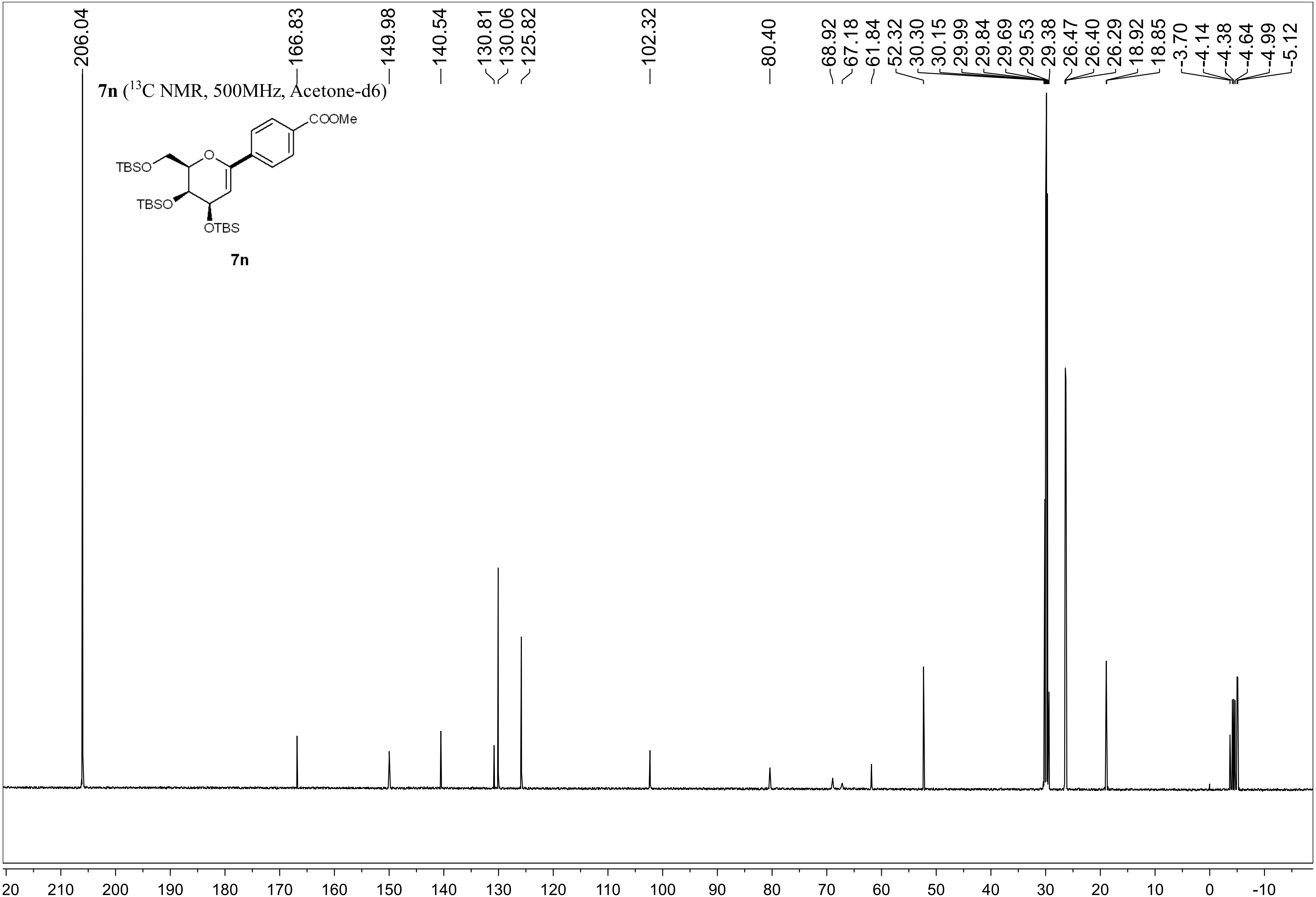

Supplementary Figure 93. ^{13}C NMR spectrum of compound **7n**

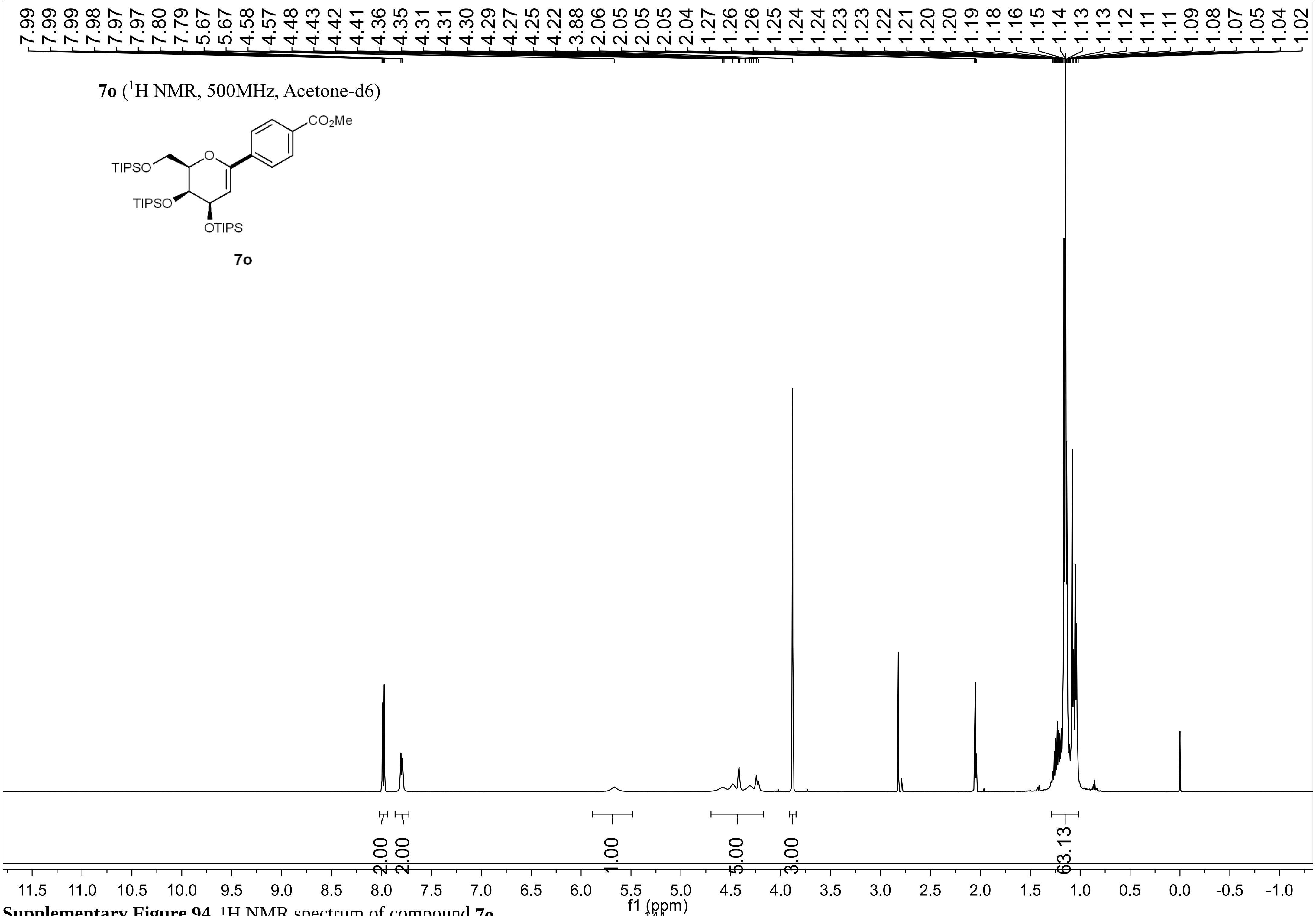

Supplementary Figure 94. ^{1}H NMR spectrum of compound **7o**

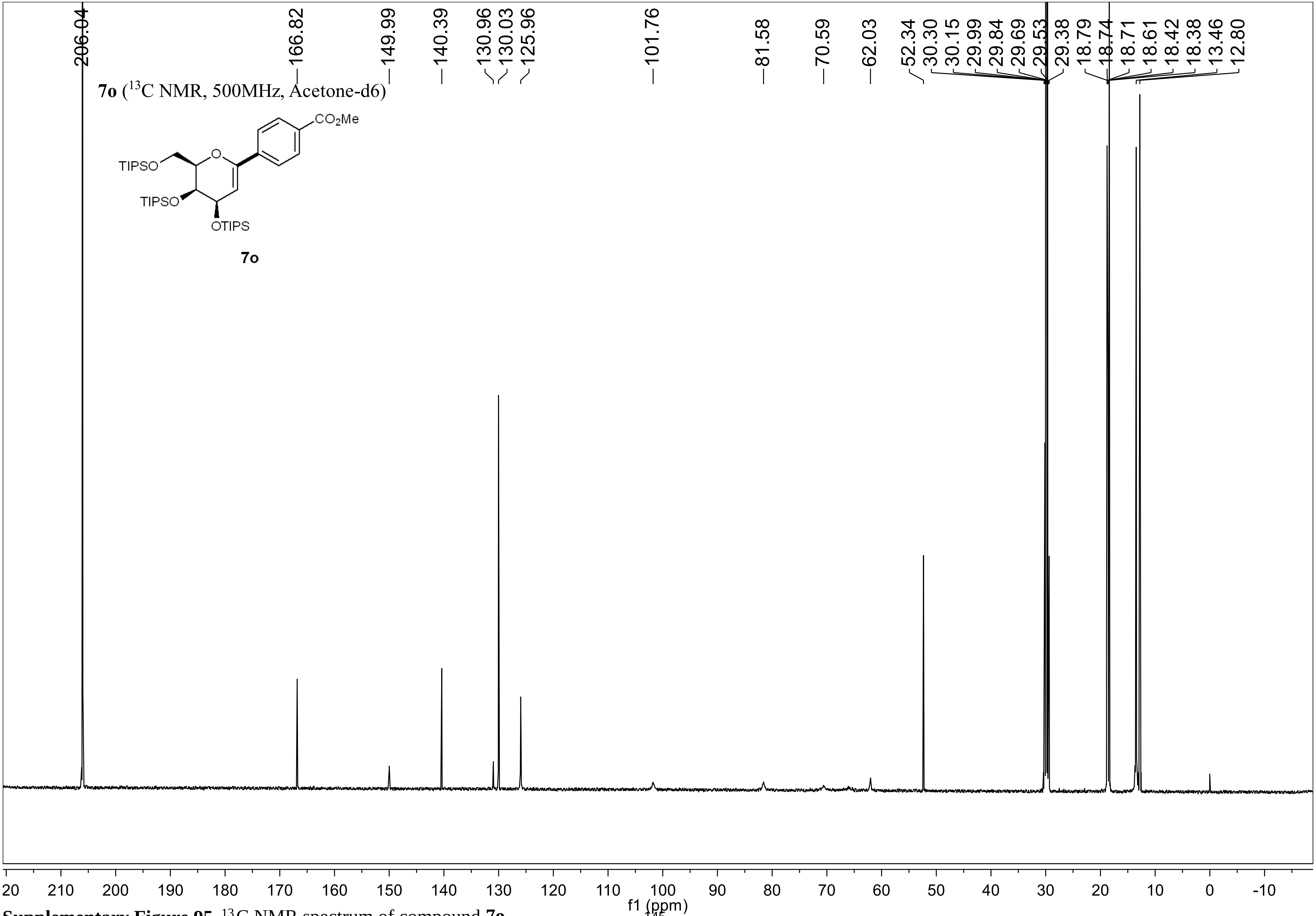

Supplementary Figure 95. ^{13}C NMR spectrum of compound **7o**

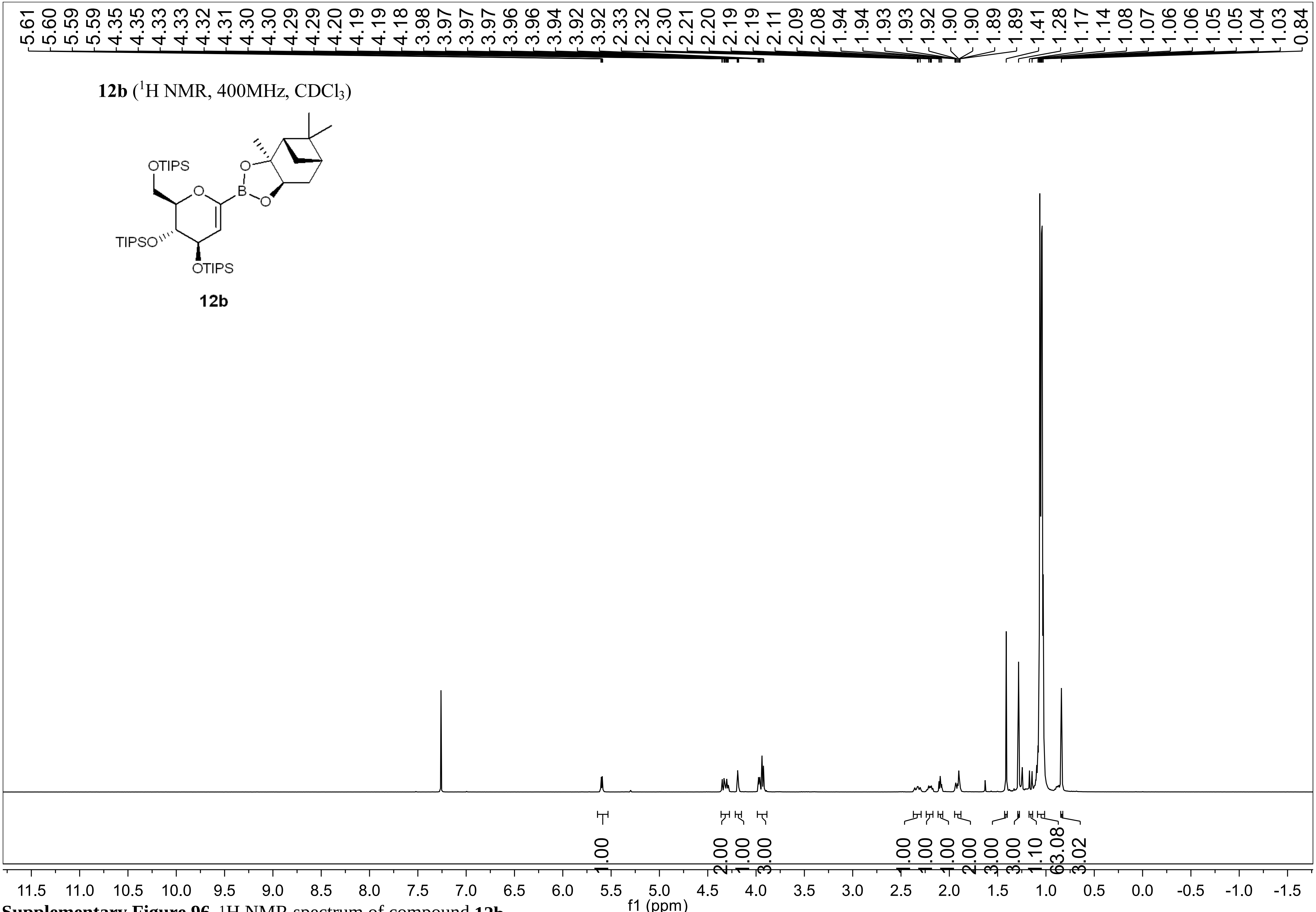

Supplementary Figure 96. ^{1}H NMR spectrum of compound **12b**

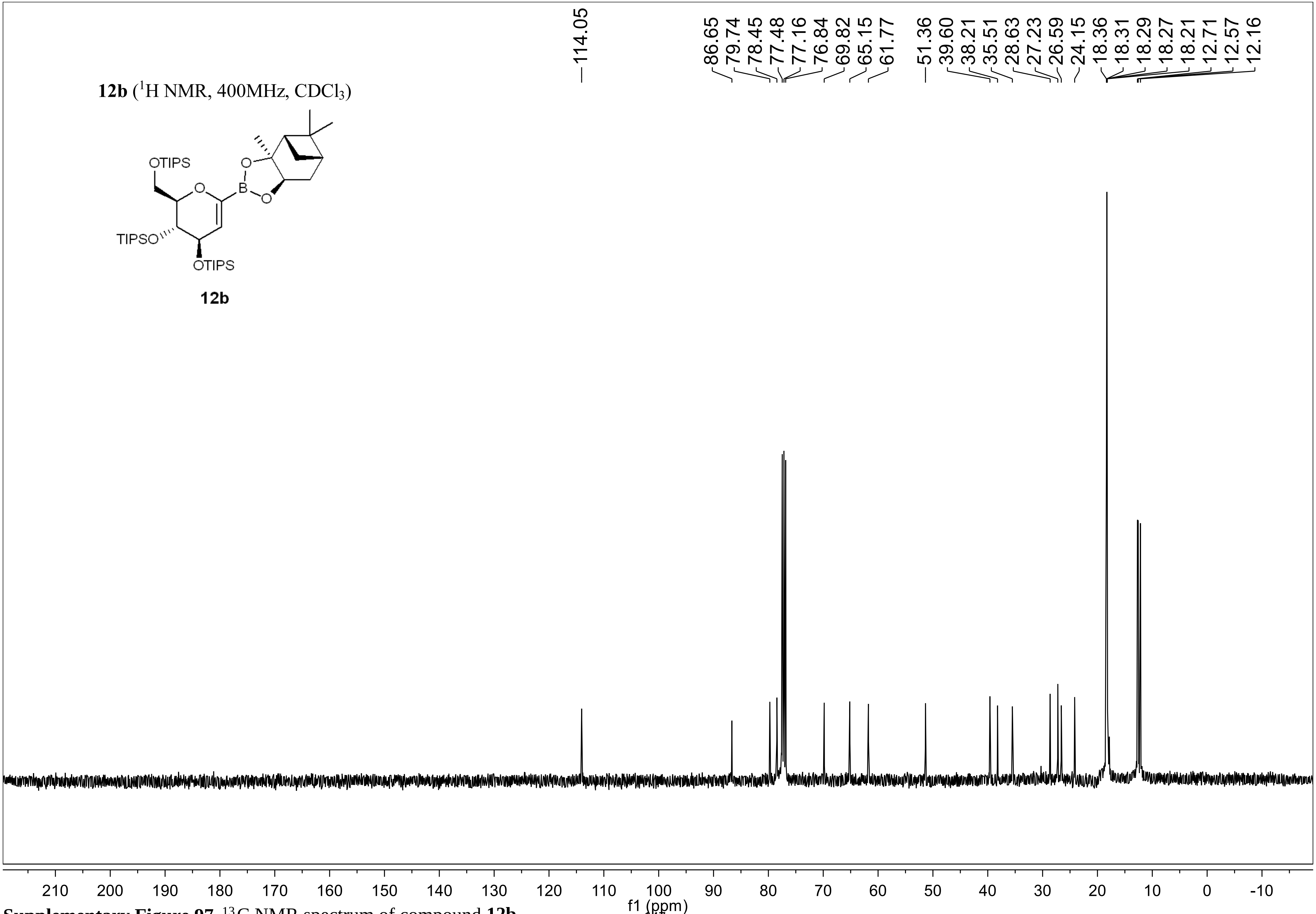

Supplementary Figure 97. ^{13}C NMR spectrum of compound **12b**

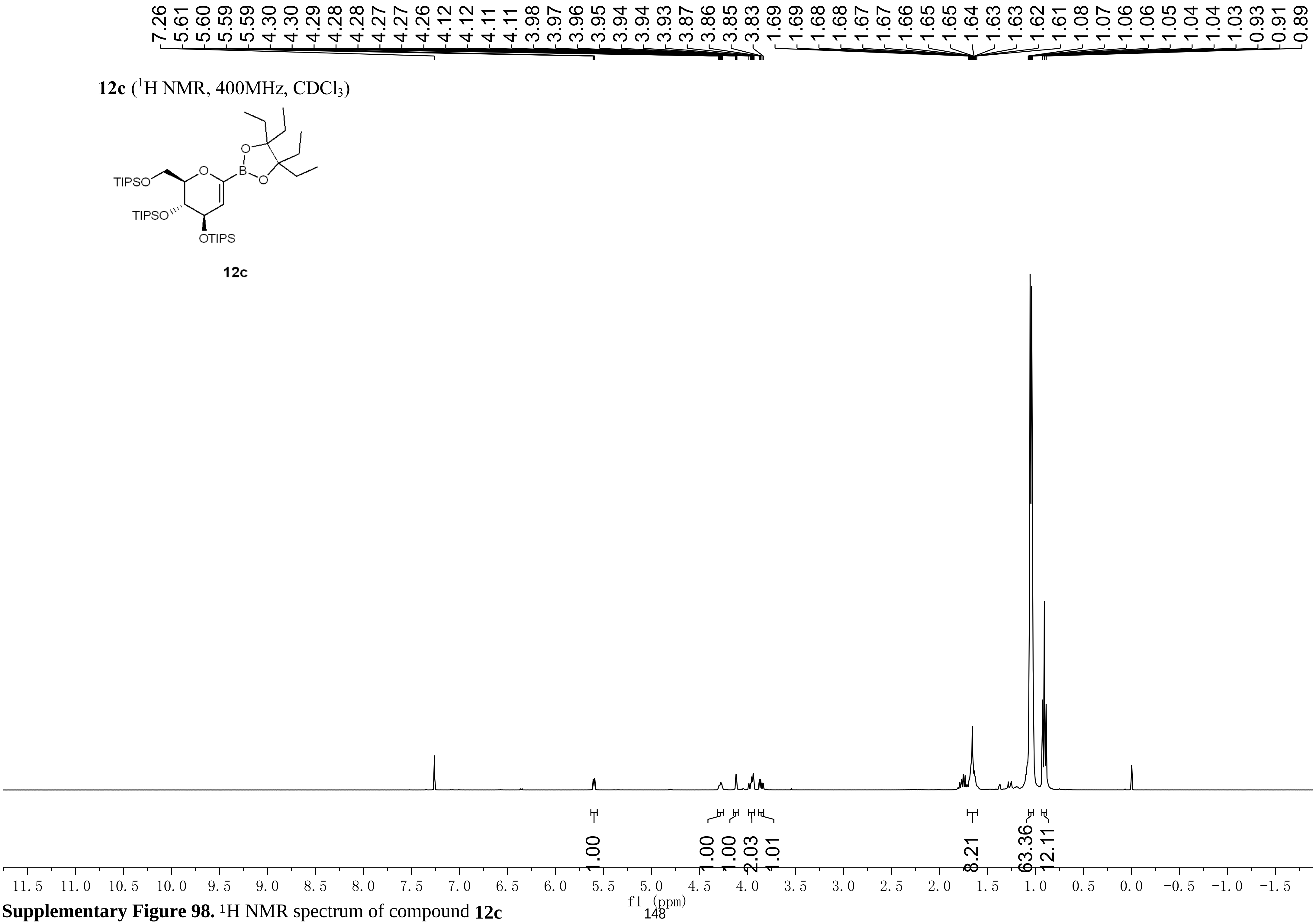

Supplementary Figure 98. ^{1}H NMR spectrum of compound **12c**

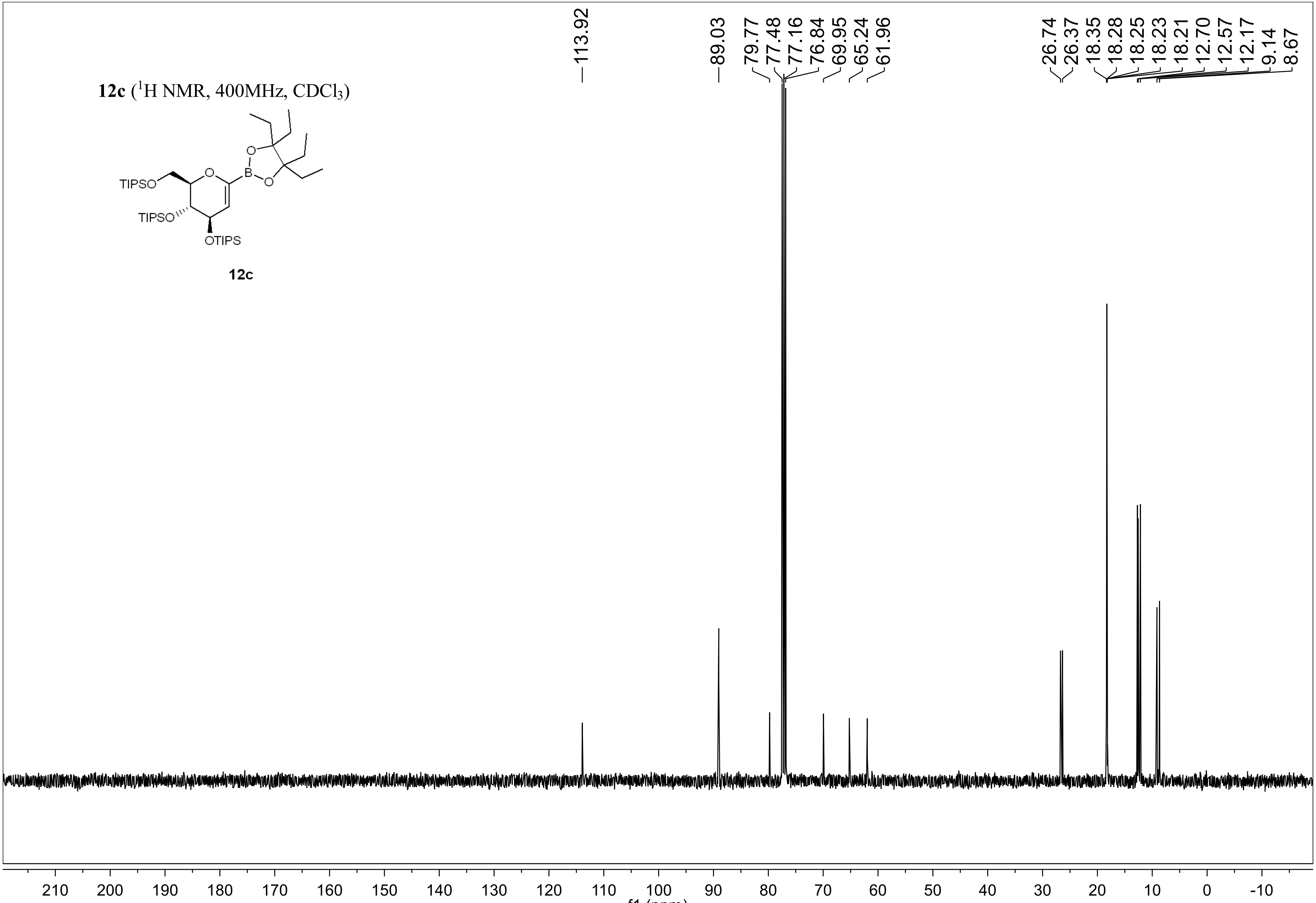

Supplementary Figure 99. ^{13}C NMR spectrum of compound **12c**

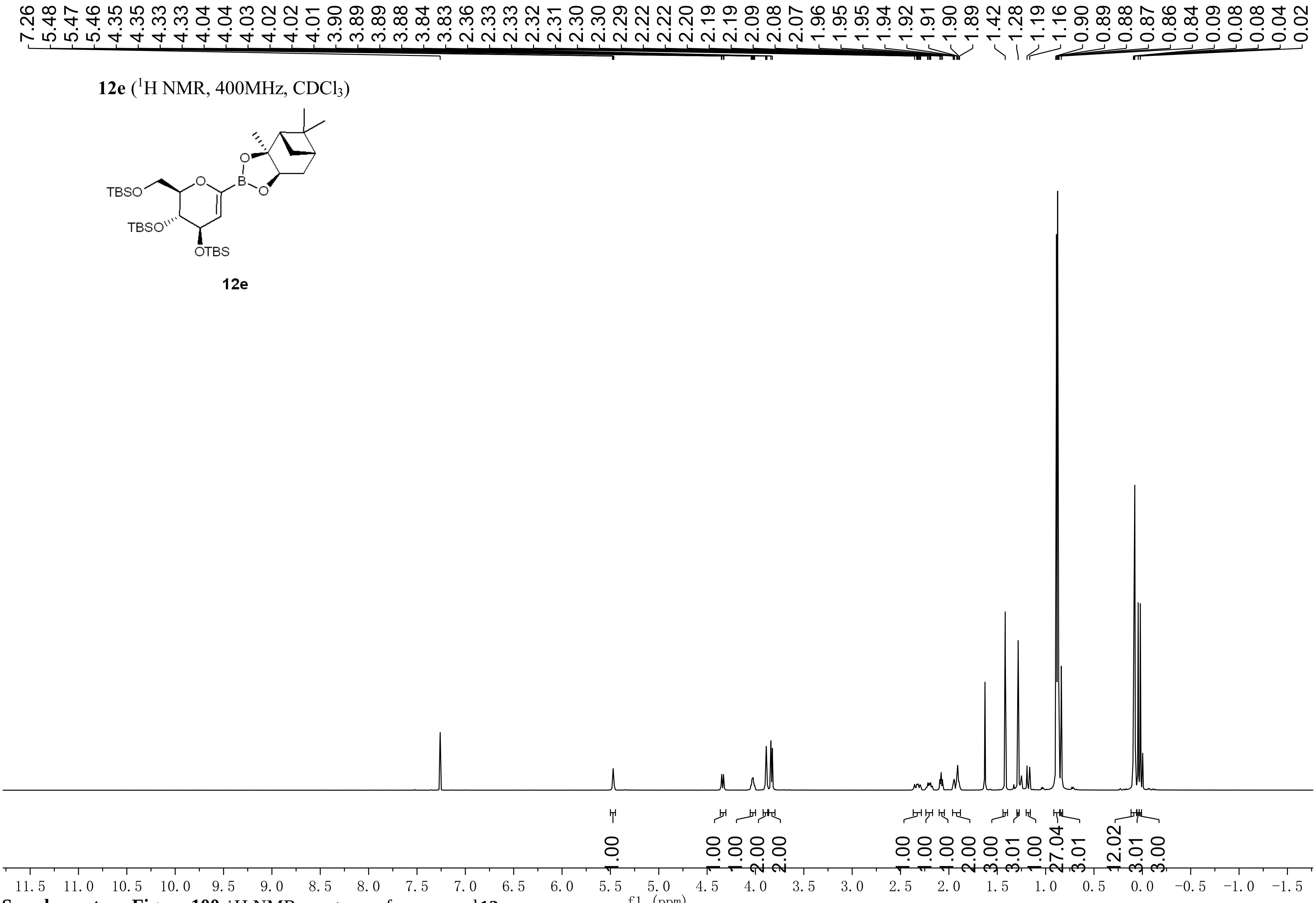

Supplementary Figure 100. ^{1}H NMR spectrum of compound **12e**

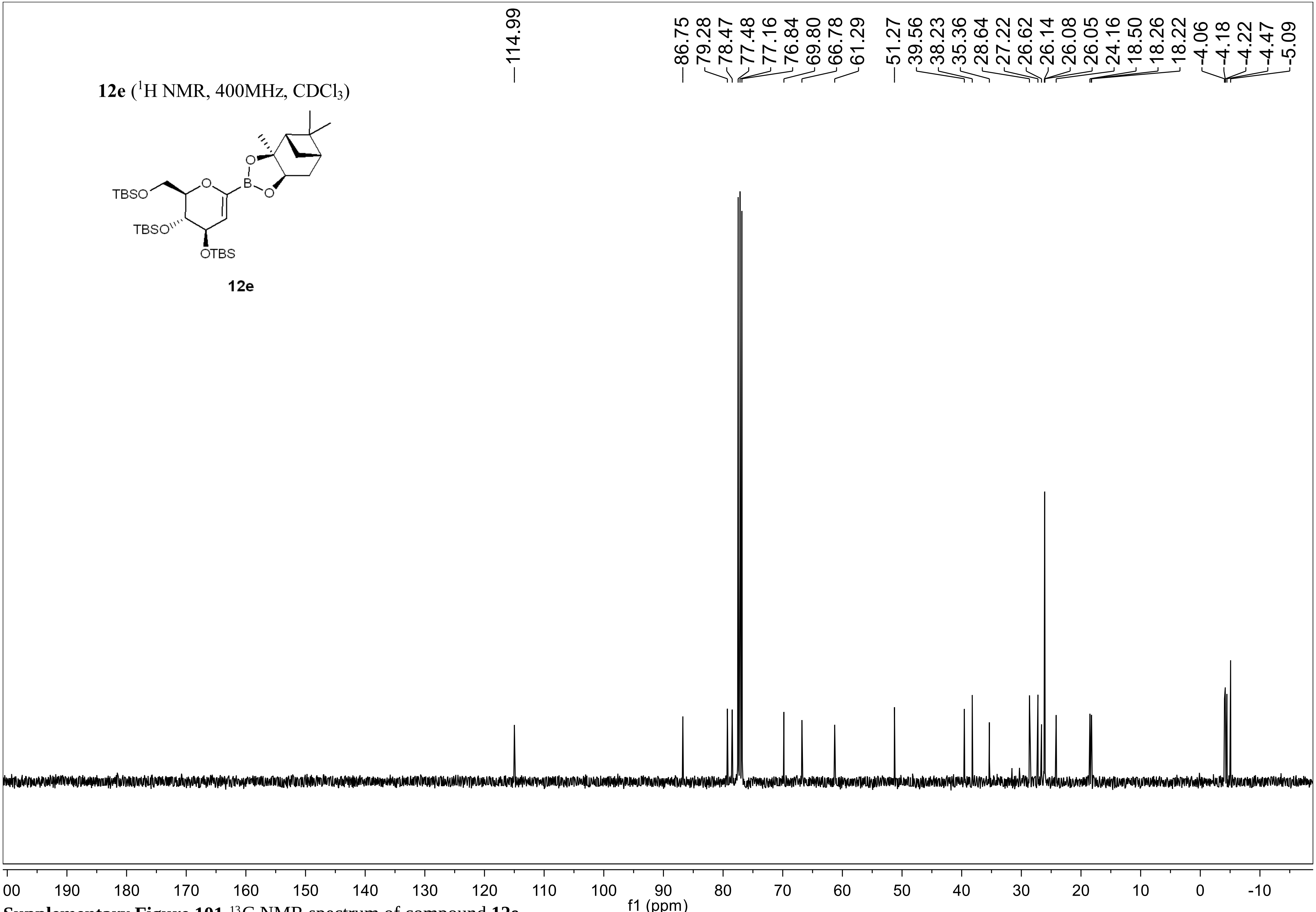

Supplementary Figure 101. ^{13}C NMR spectrum of compound **12e**

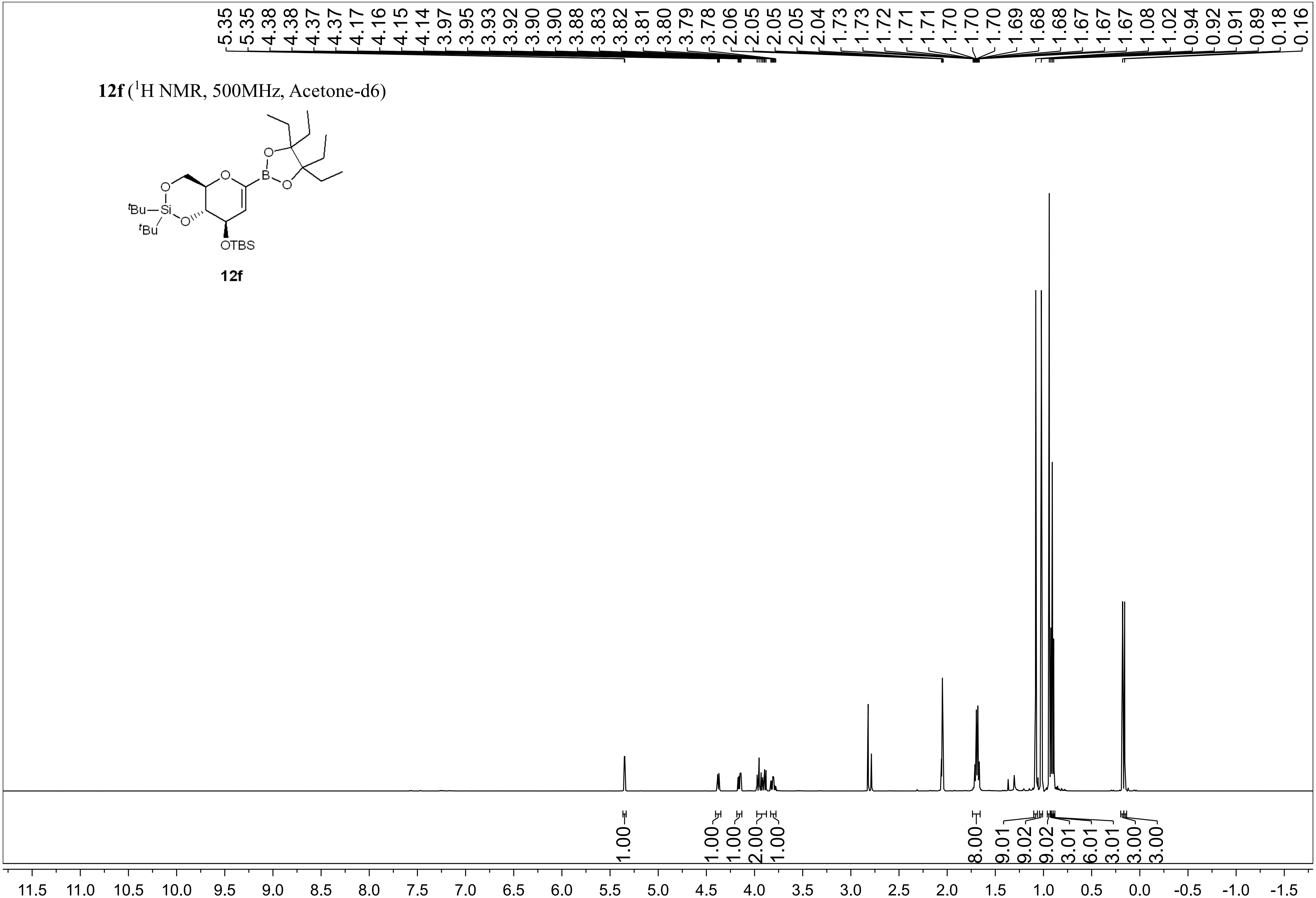

Supplementary Figure 102. ^{1}H NMR spectrum of compound **12f**

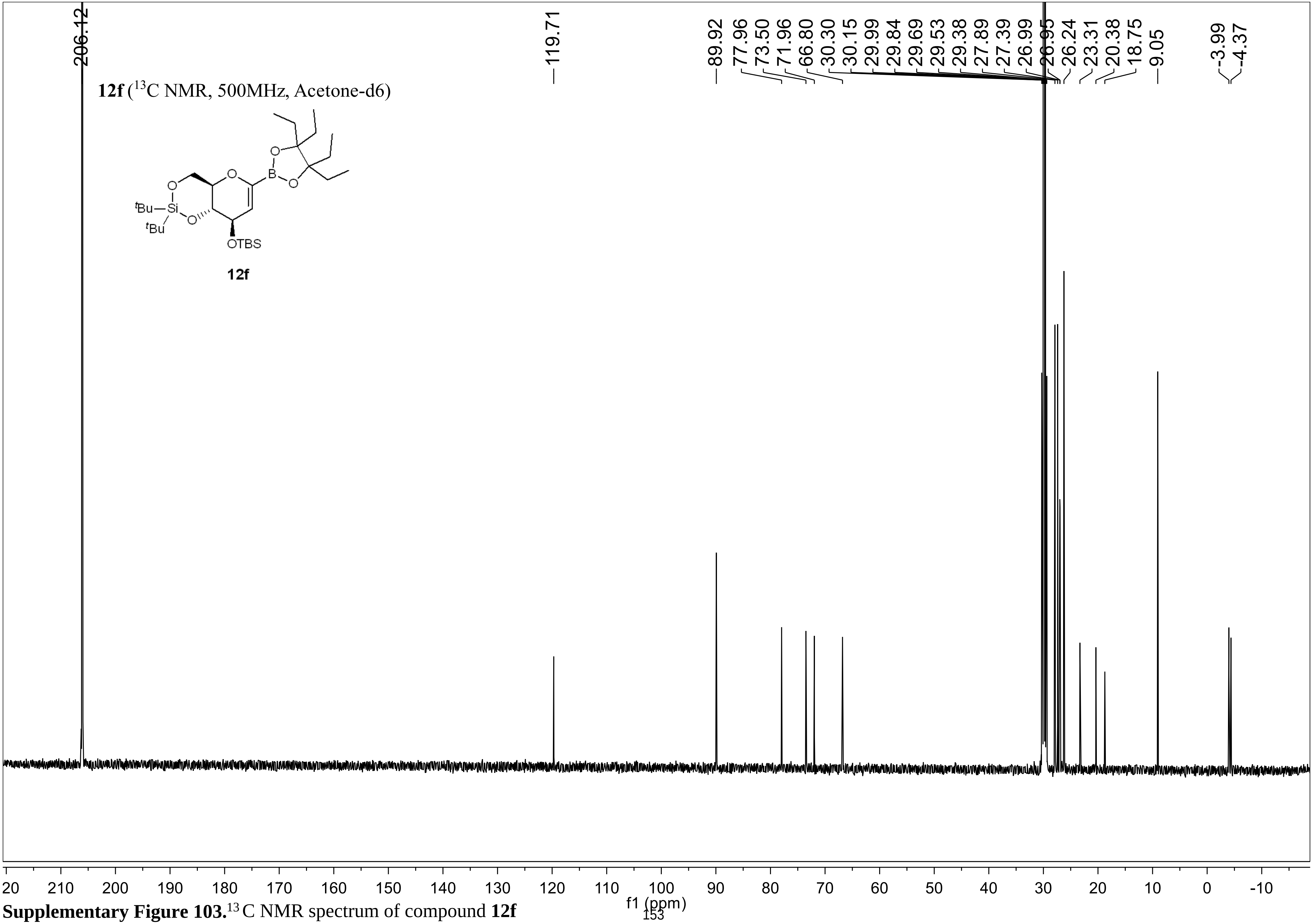

Supplementary Figure 103. ^{13}C NMR spectrum of compound **12f**

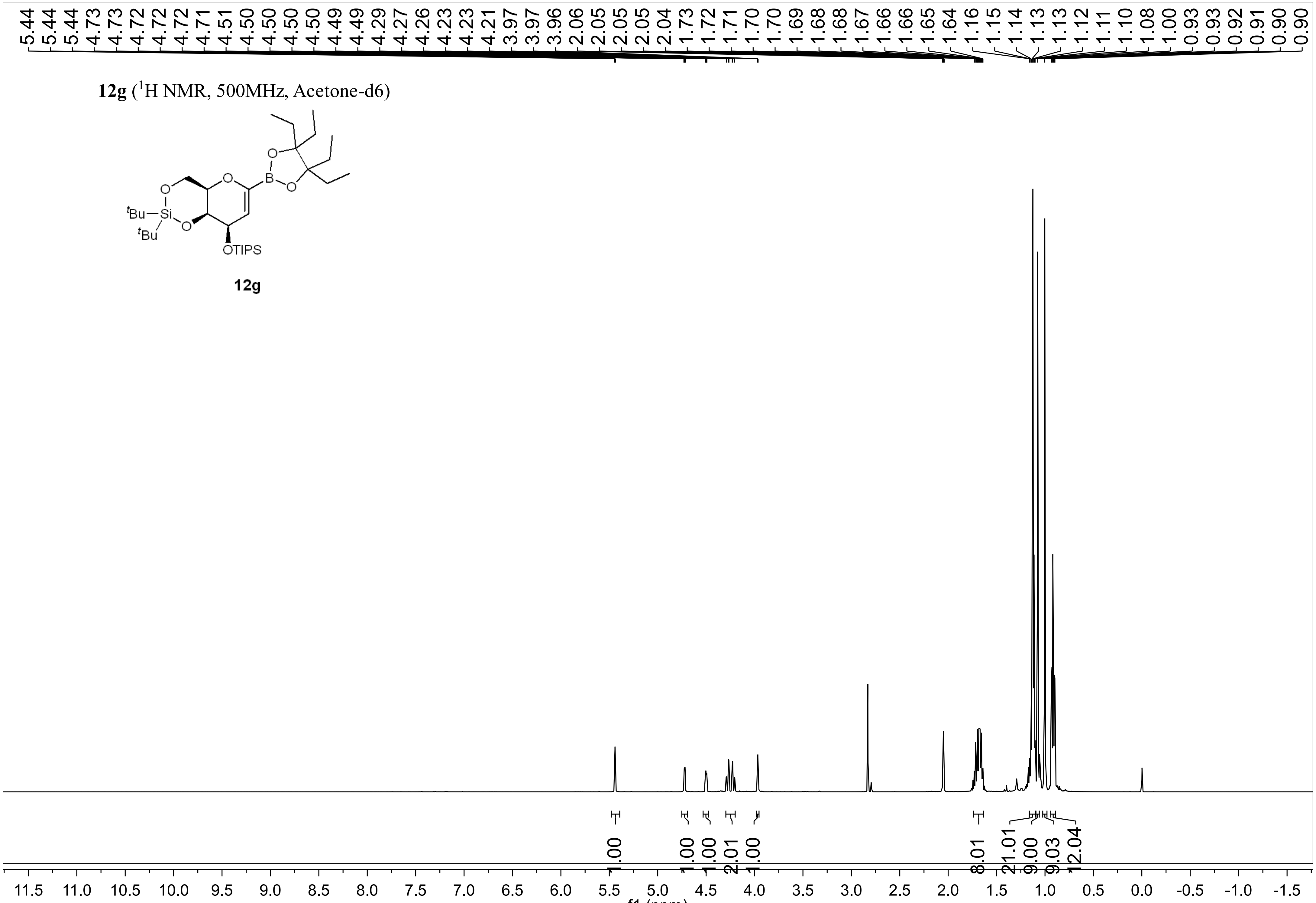

Supplementary Figure 104. ^{1}H NMR spectrum of compound **12g**

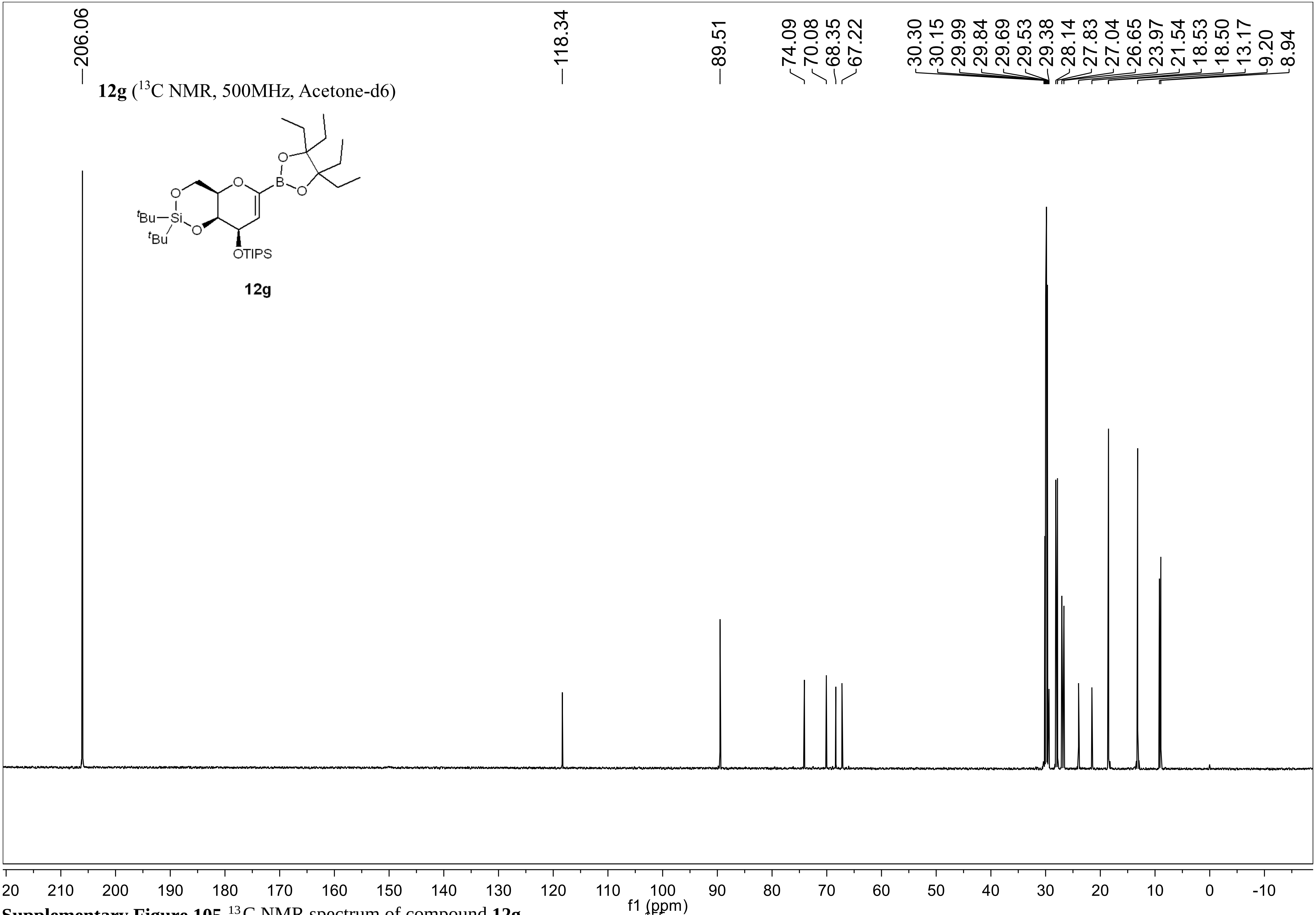

Supplementary Figure 105. ^{13}C NMR spectrum of compound **12g**

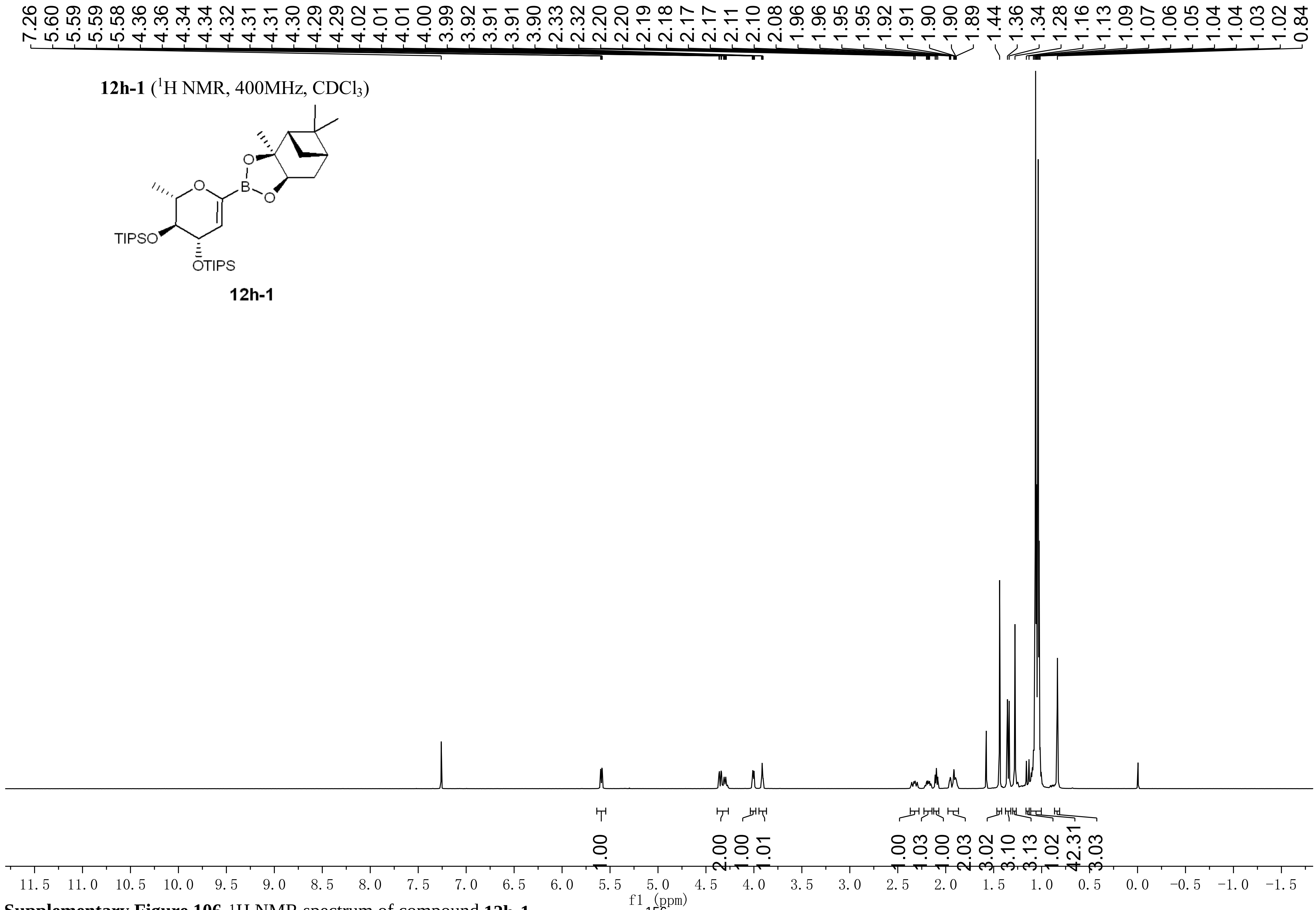

Supplementary Figure 106. ^{1}H NMR spectrum of compound **12h-1**

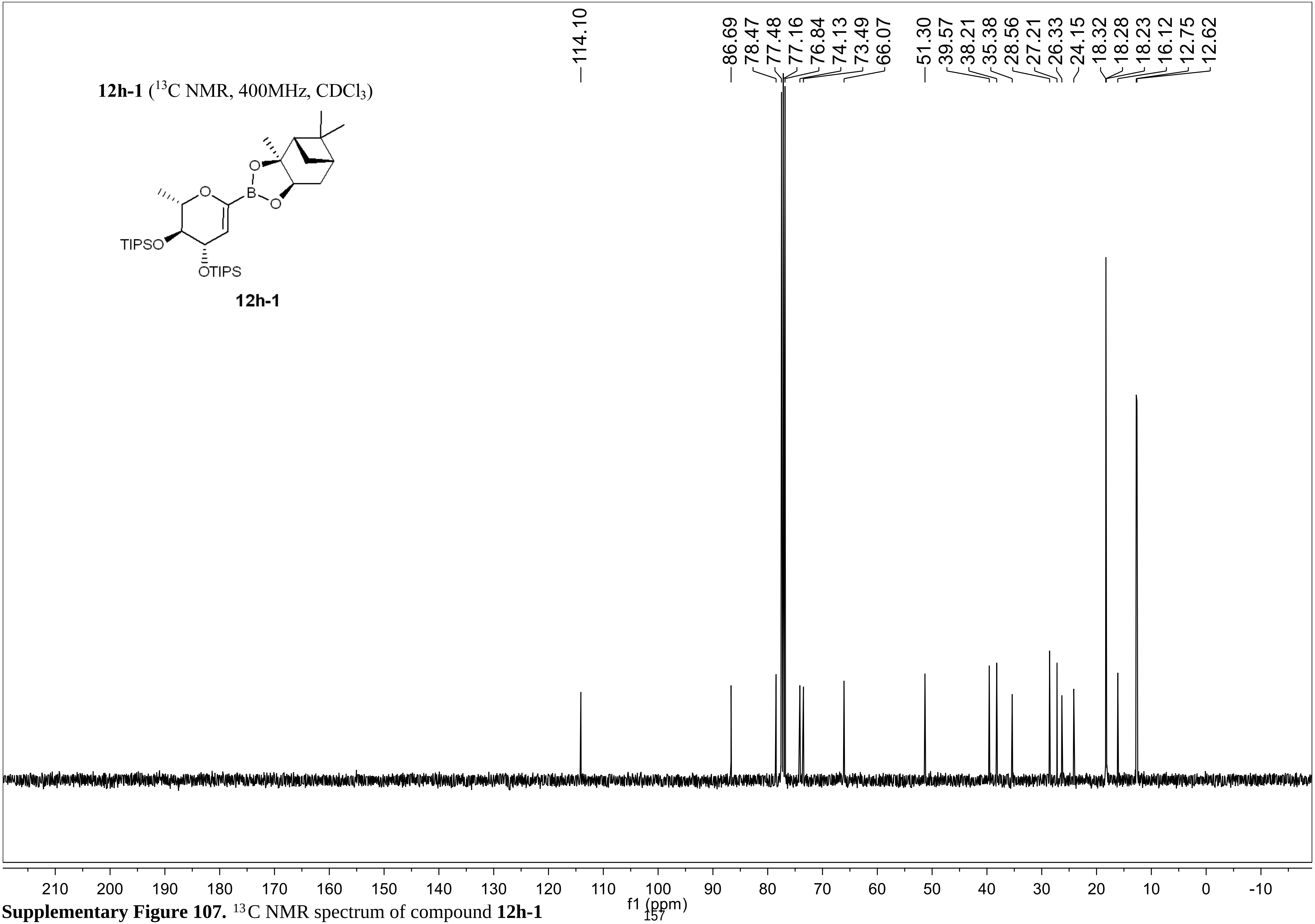

Supplementary Figure 107. ^{13}C NMR spectrum of compound **12h-1**

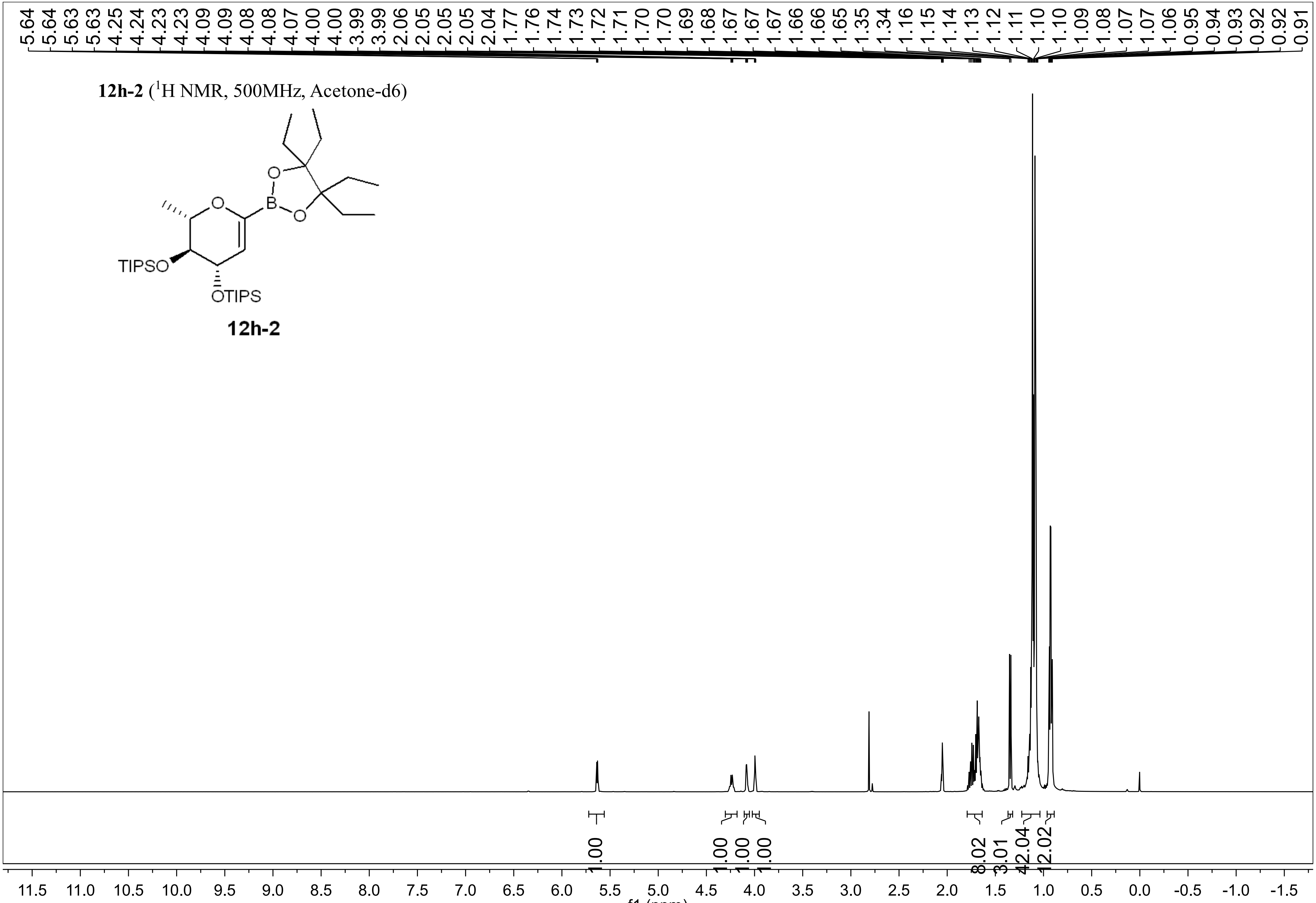

Supplementary Figure 108. ^{1}H NMR spectrum of compound **12h-2**

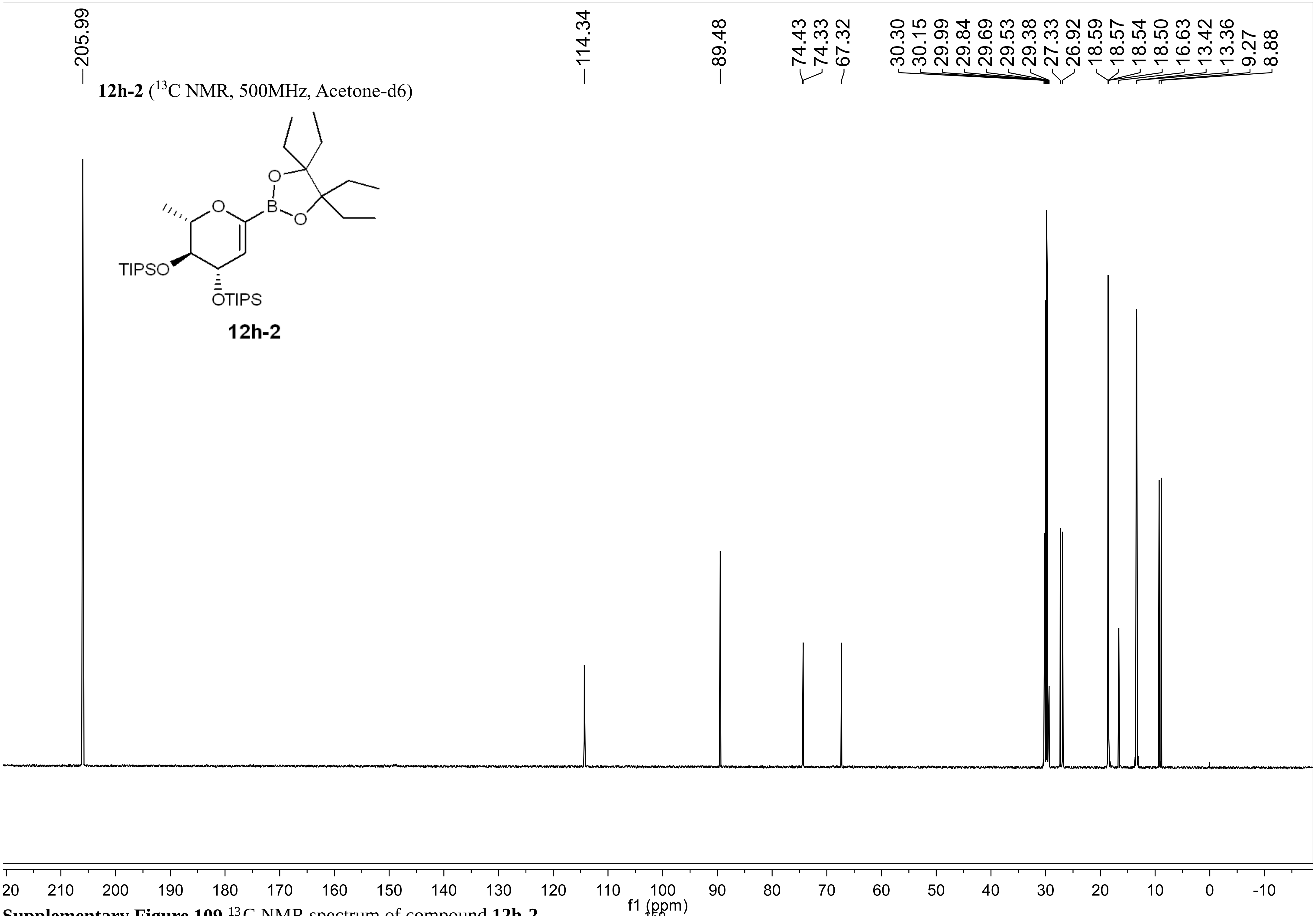

Supplementary Figure 109. ^{13}C NMR spectrum of compound **12h-2**

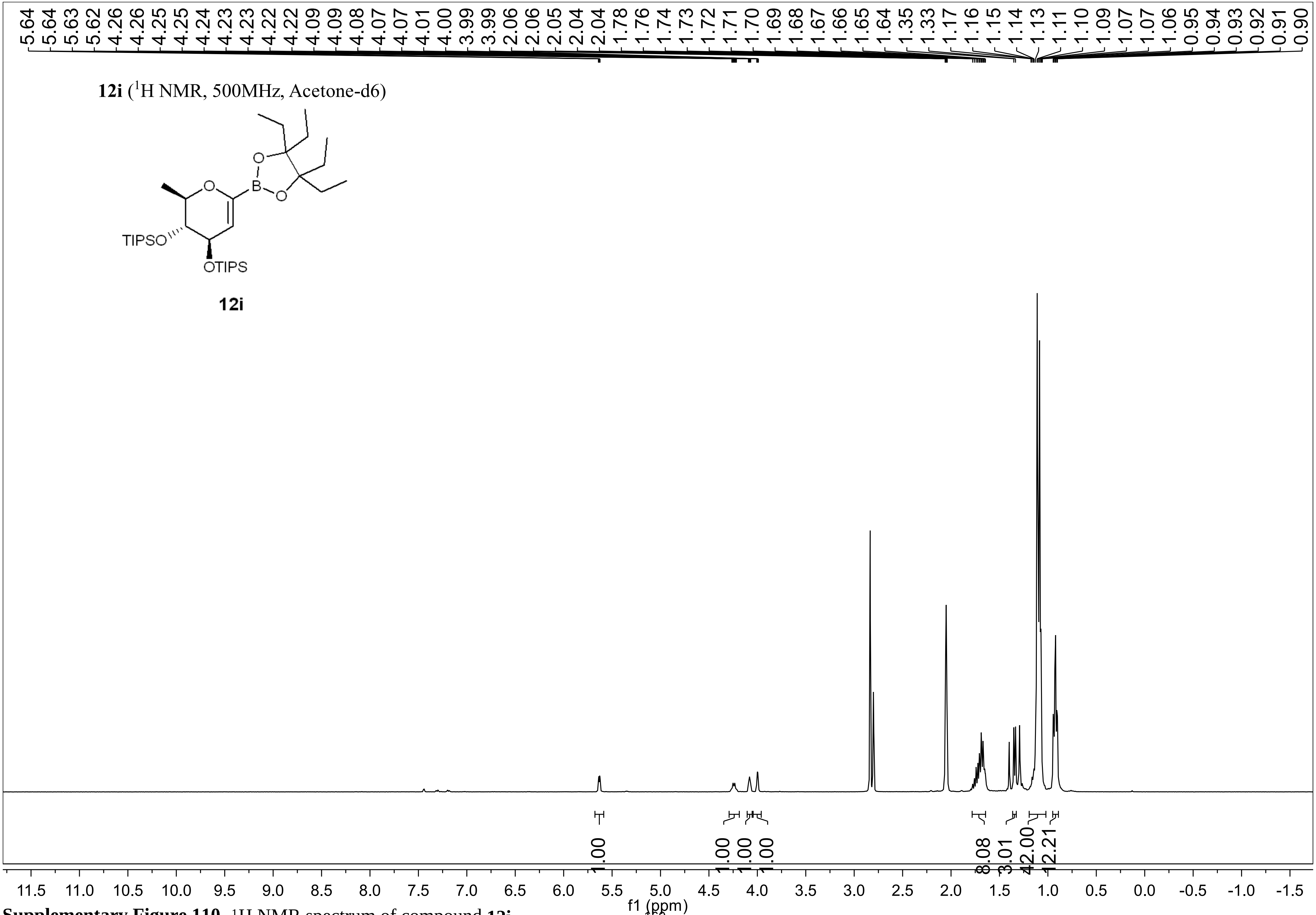

Supplementary Figure 110. ^{1}H NMR spectrum of compound **12i**

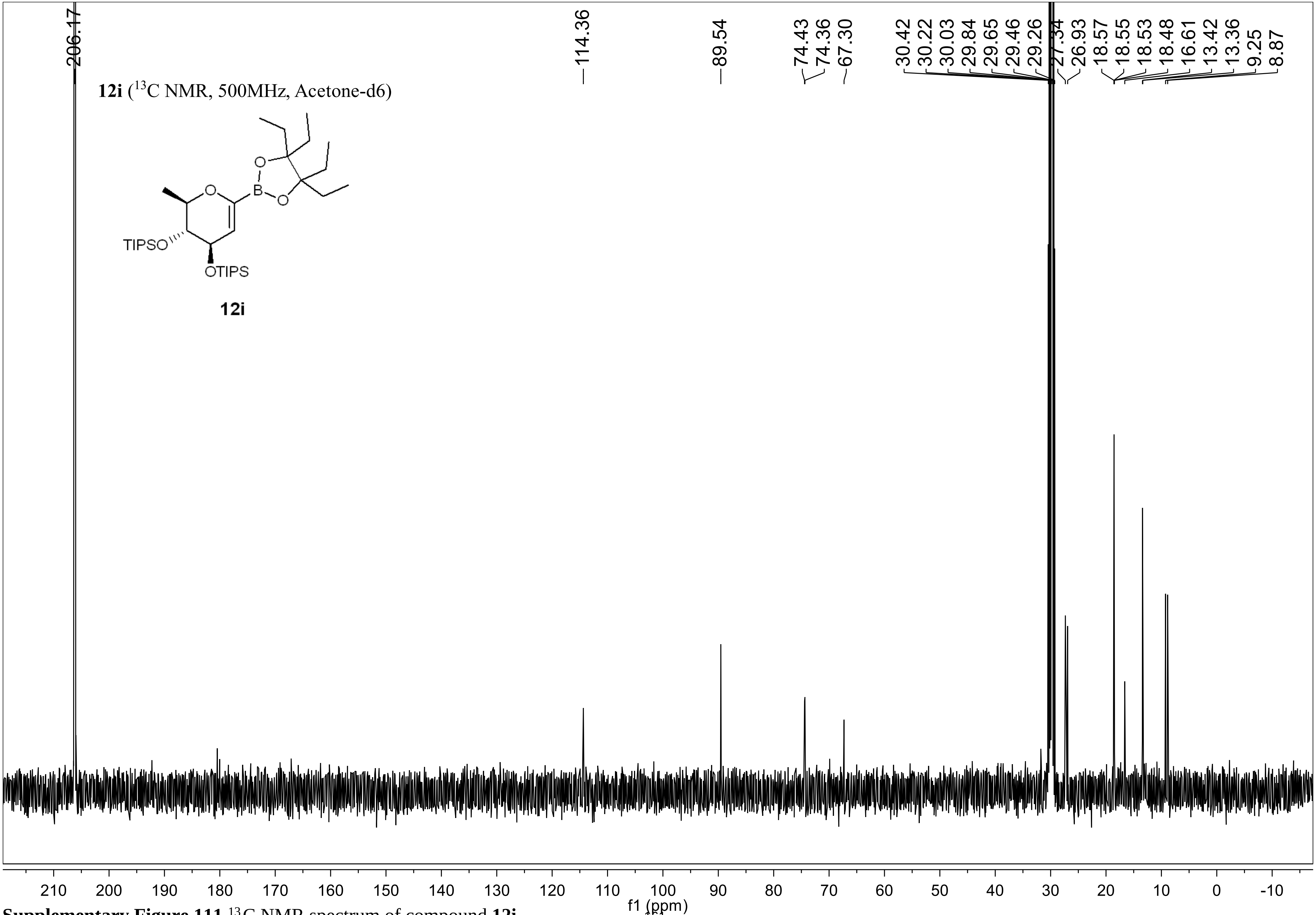

Supplementary Figure 111. ^{13}C NMR spectrum of compound **12i**

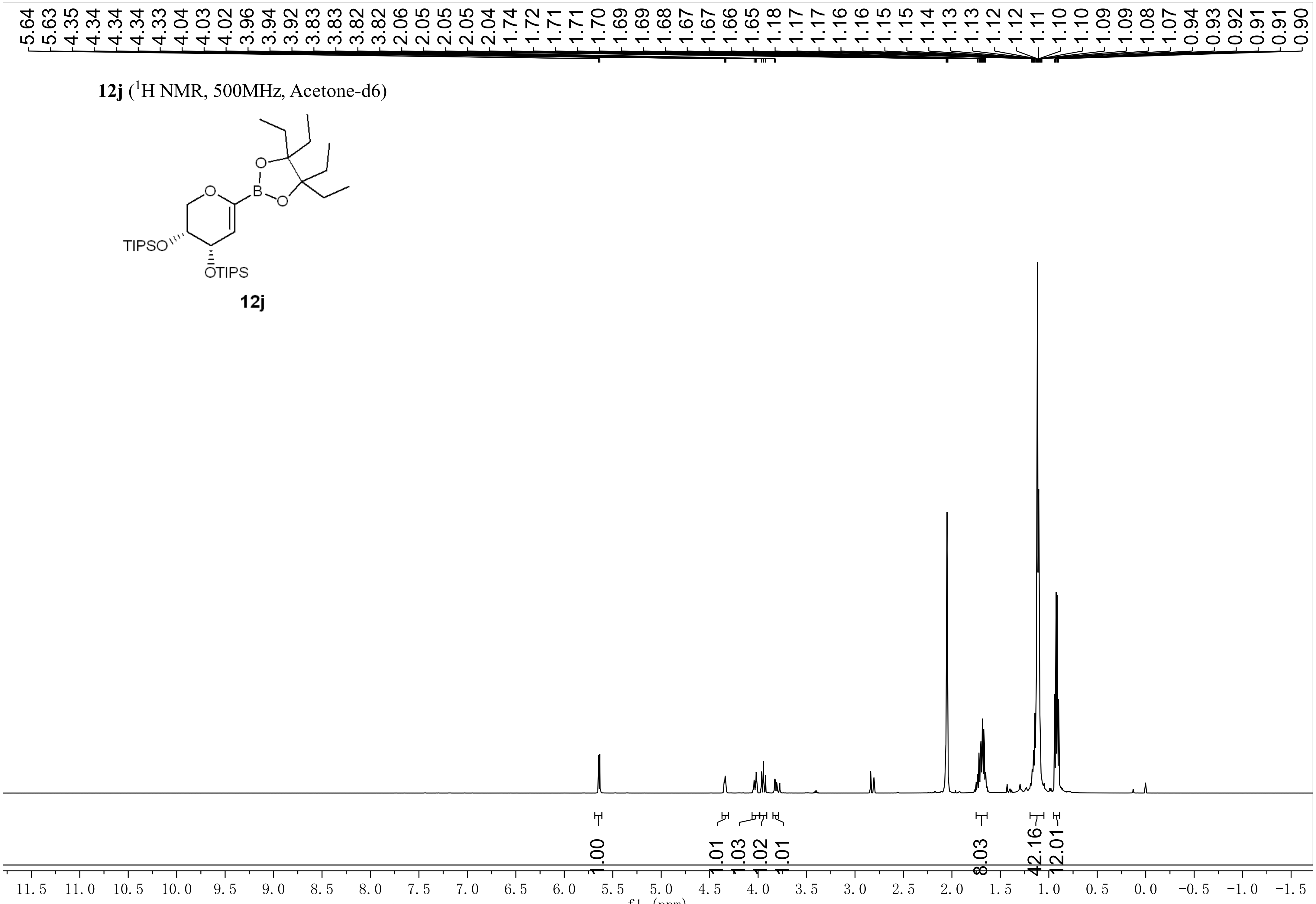

Supplementary Figure 112. ^{1}H NMR spectrum of compound **12j**

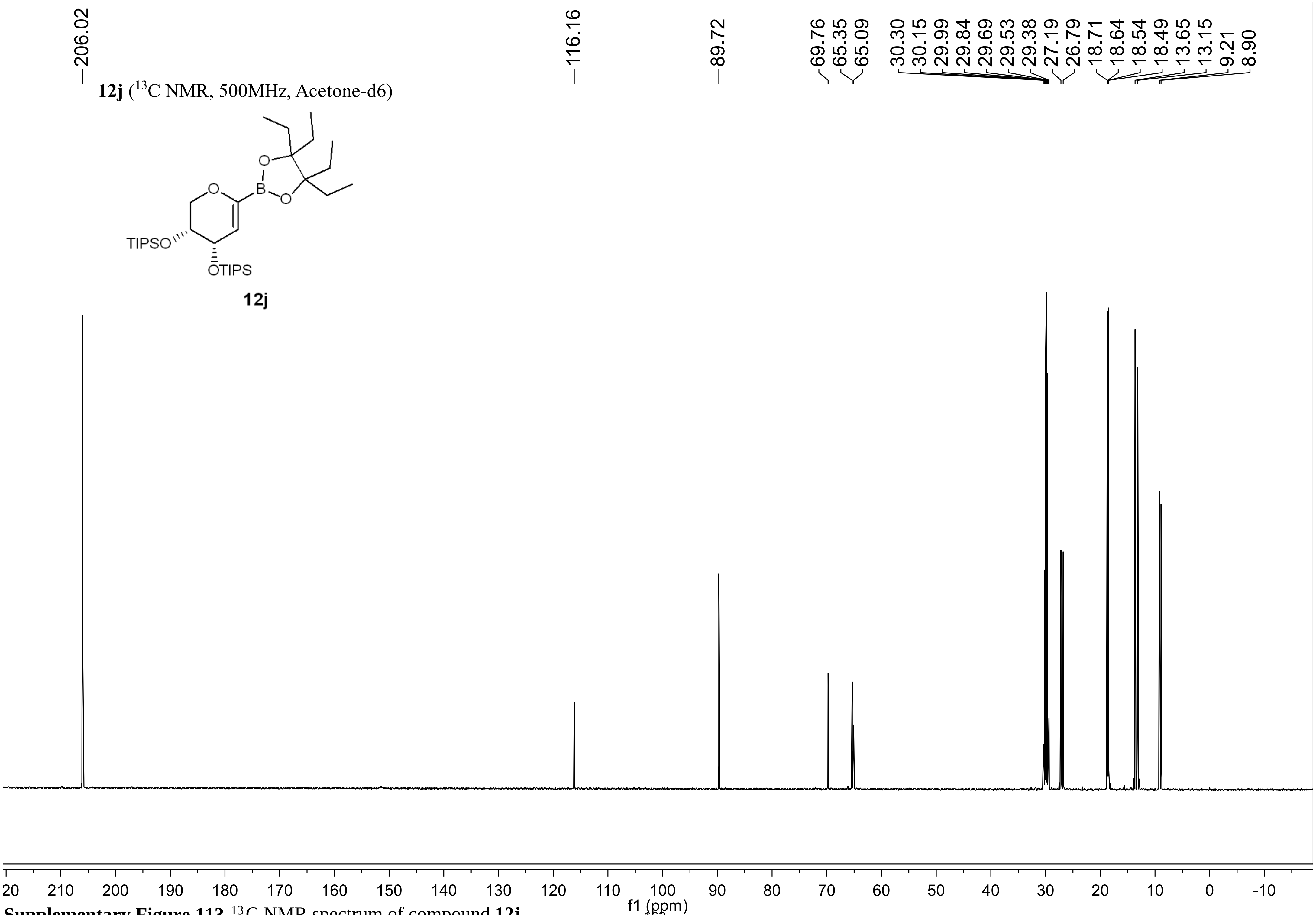

Supplementary Figure 113. ^{13}C NMR spectrum of compound **12j**

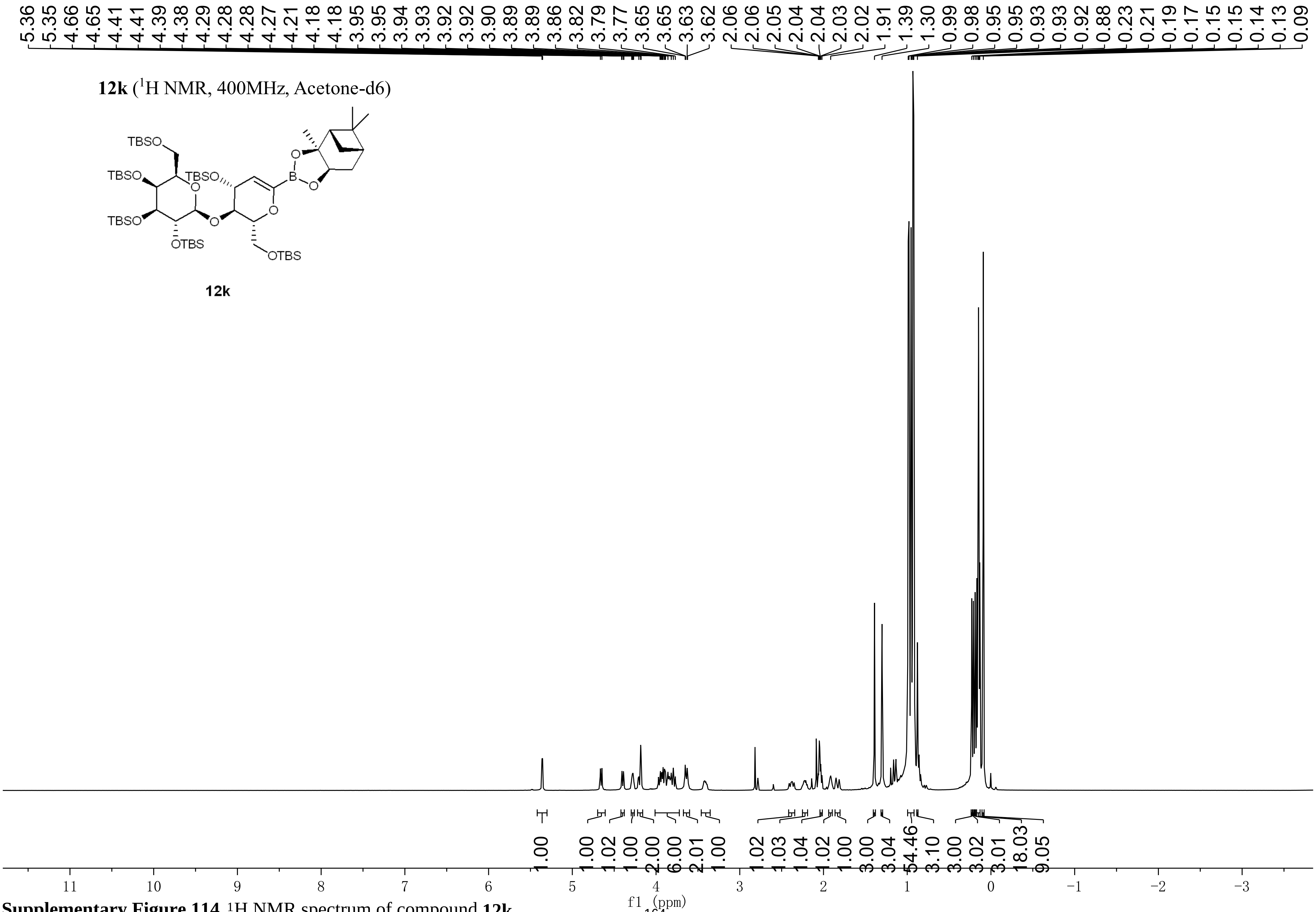

Supplementary Figure 114. ^{1}H NMR spectrum of compound **12k**

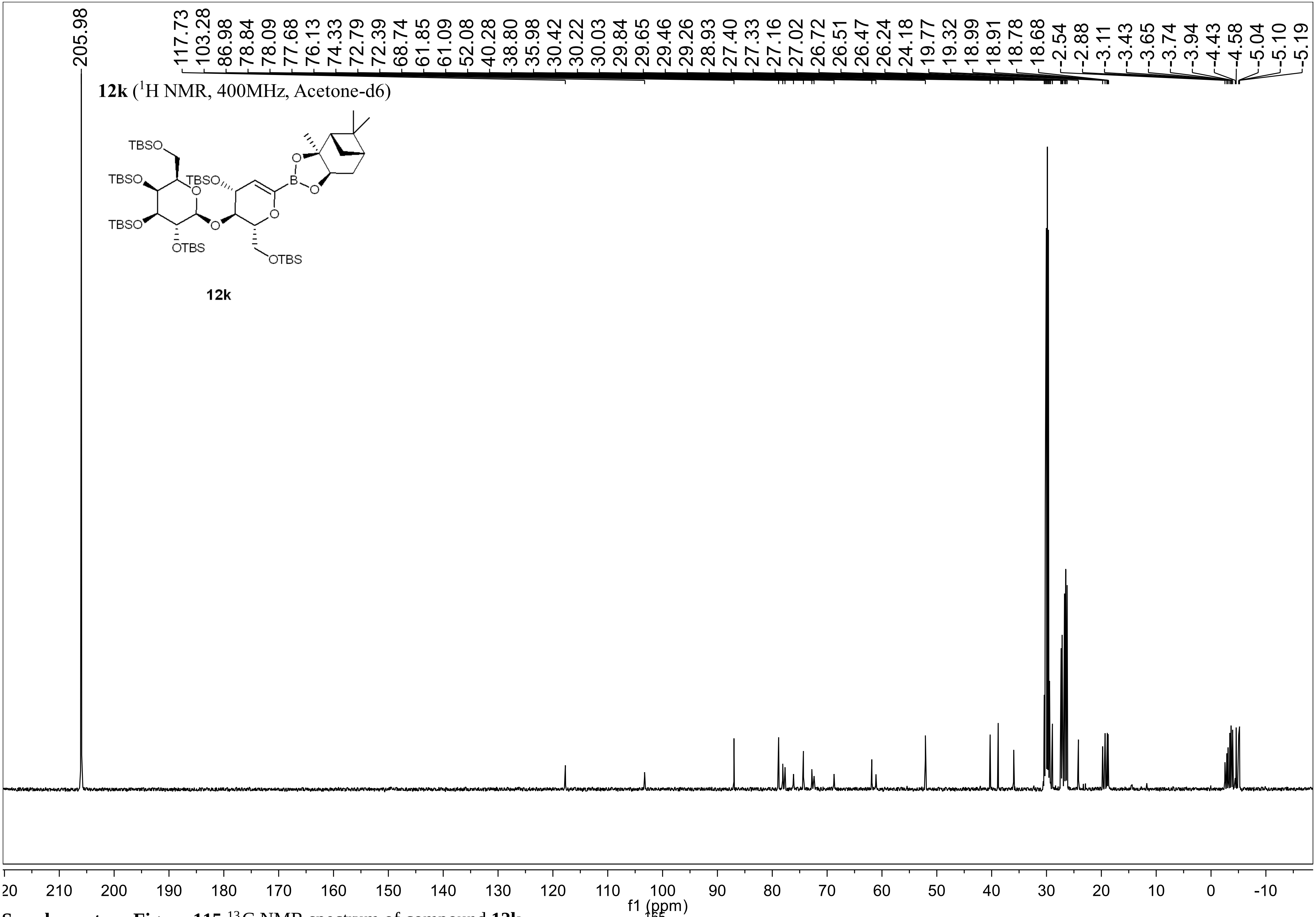

Supplementary Figure 115. ^{13}C NMR spectrum of compound **12k**

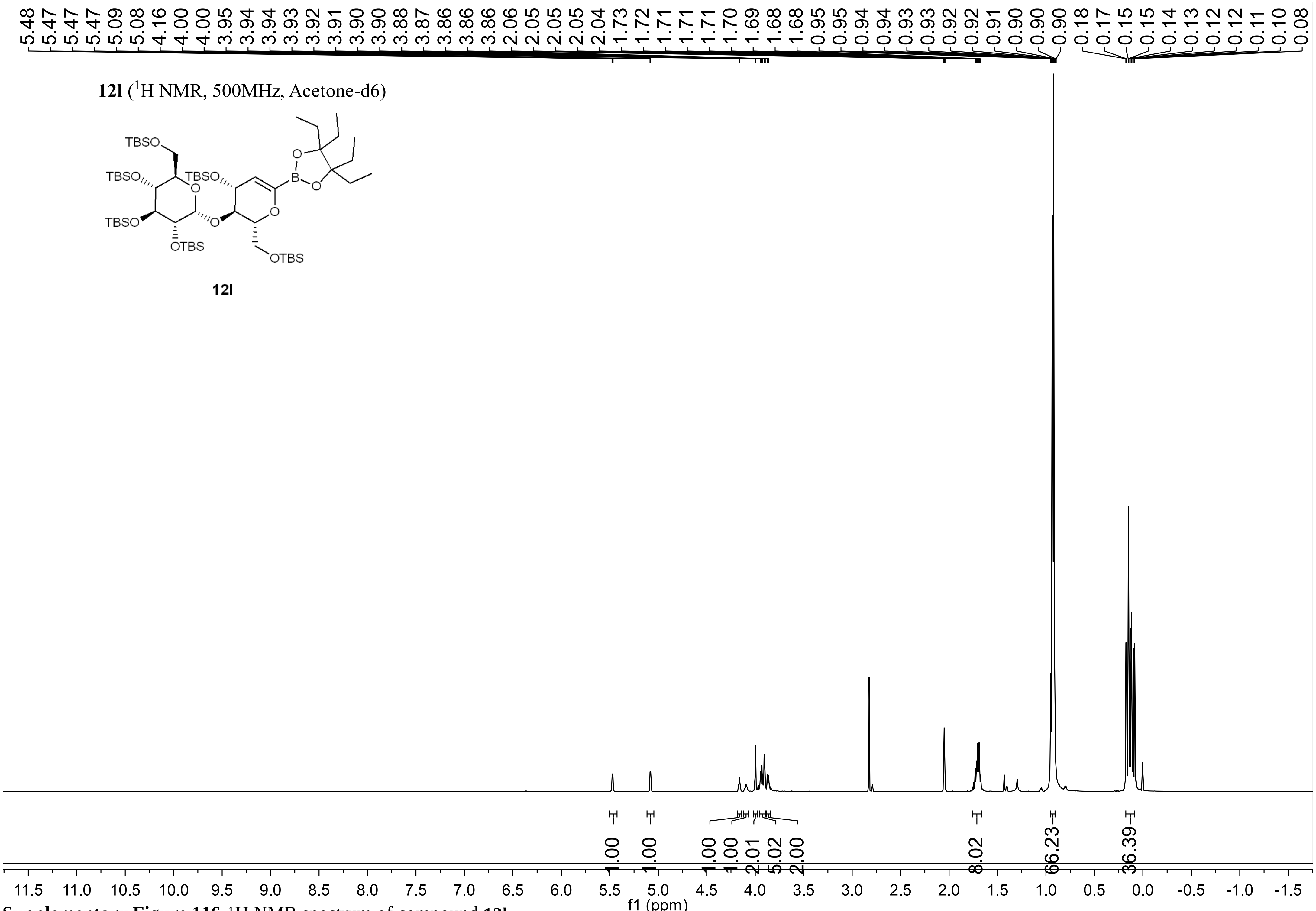

Supplementary Figure 116. ^{1}H NMR spectrum of compound **12l**

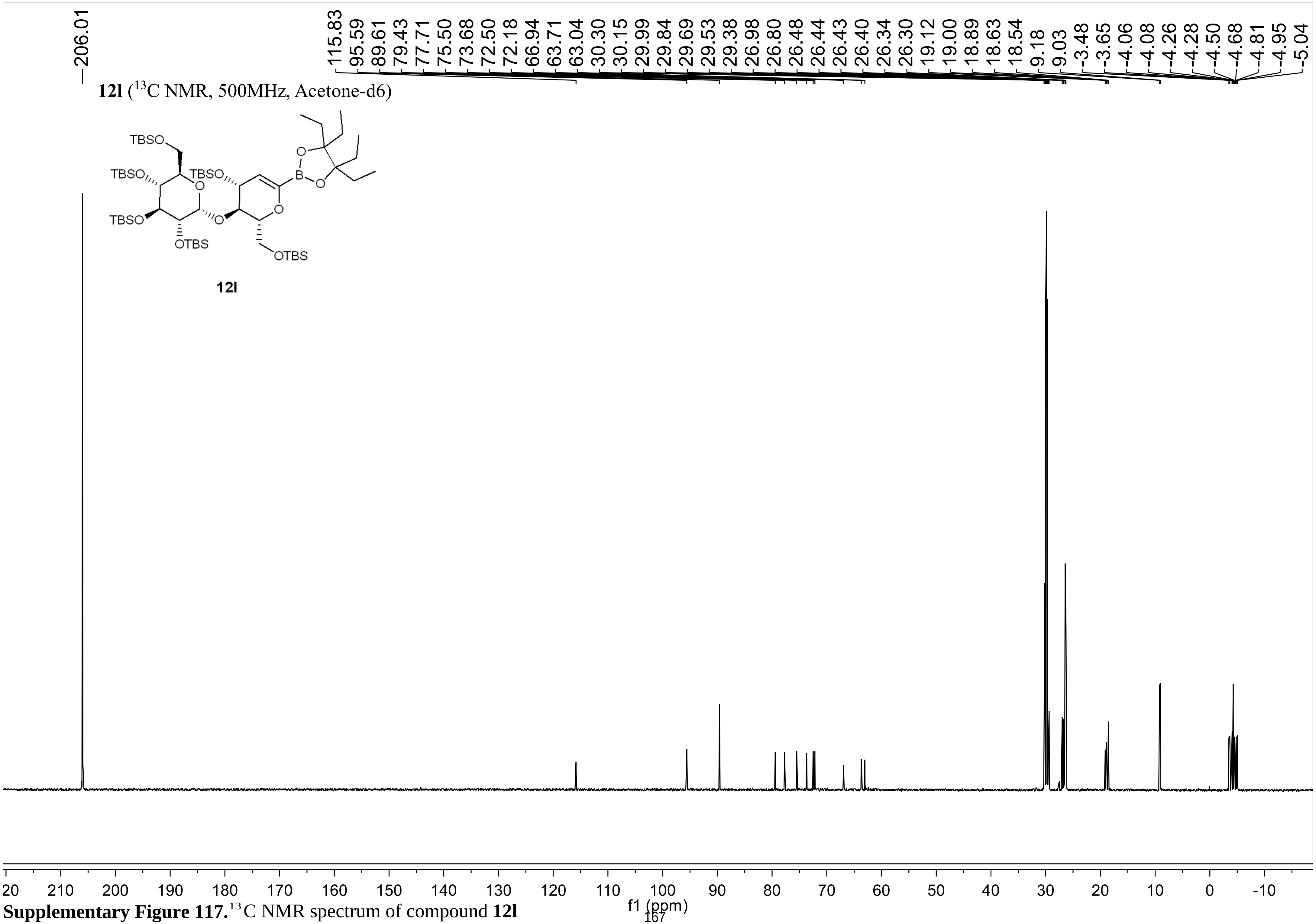

Supplementary Figure 117. ^{13}C NMR spectrum of compound **12l**

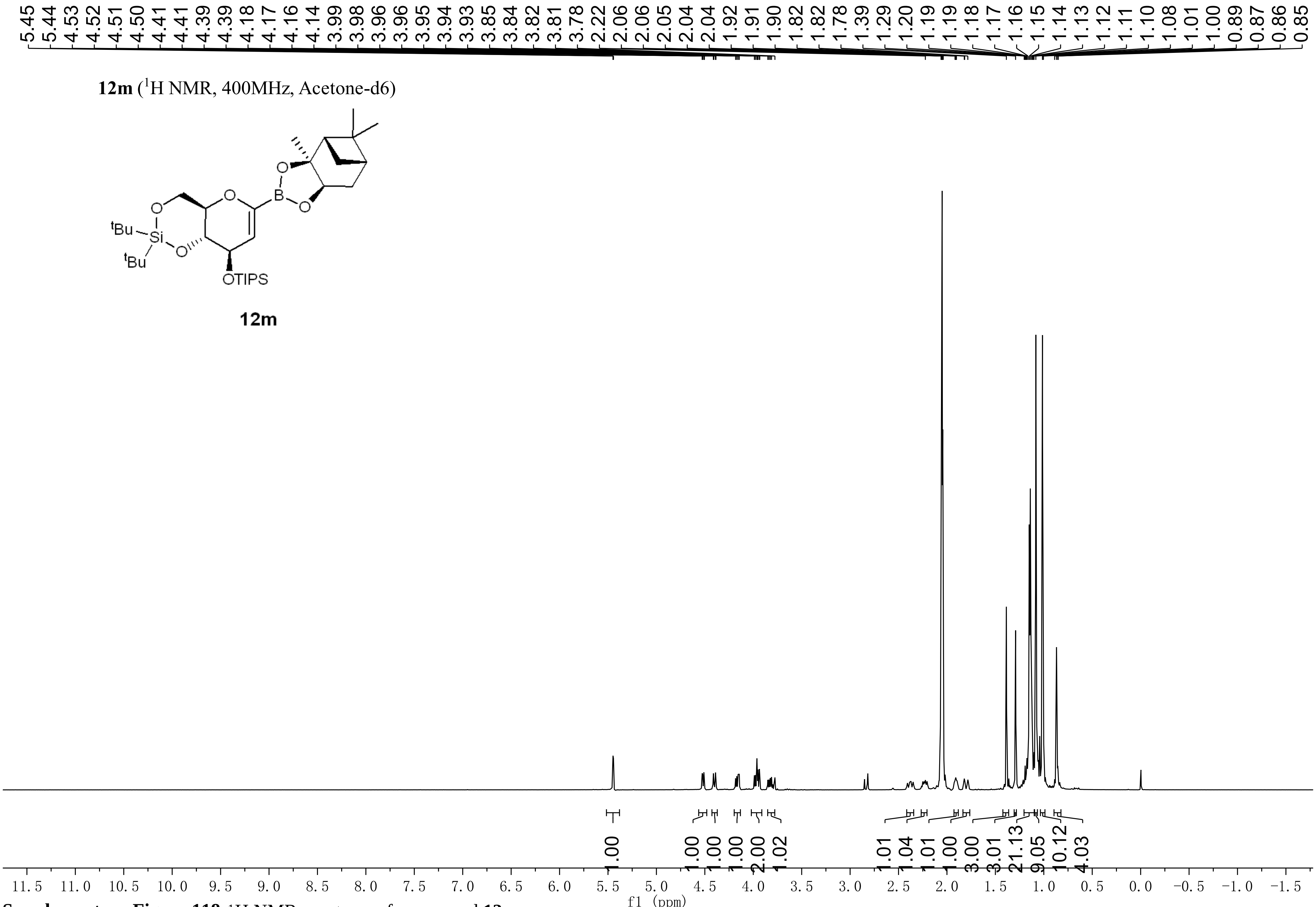

Supplementary Figure 118. ^{1}H NMR spectrum of compound **12m**

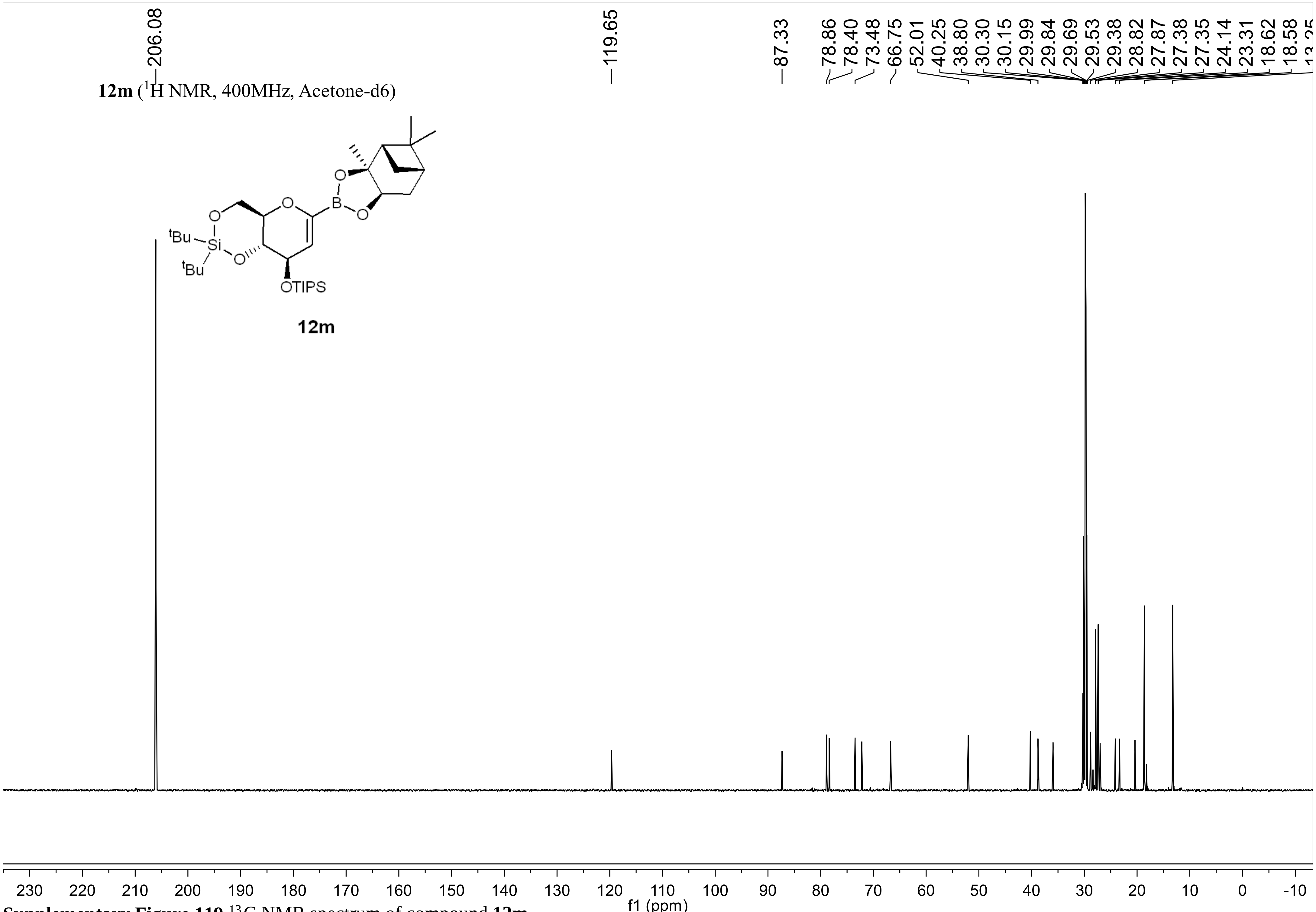

Supplementary Figure 119. ^{13}C NMR spectrum of compound **12m**

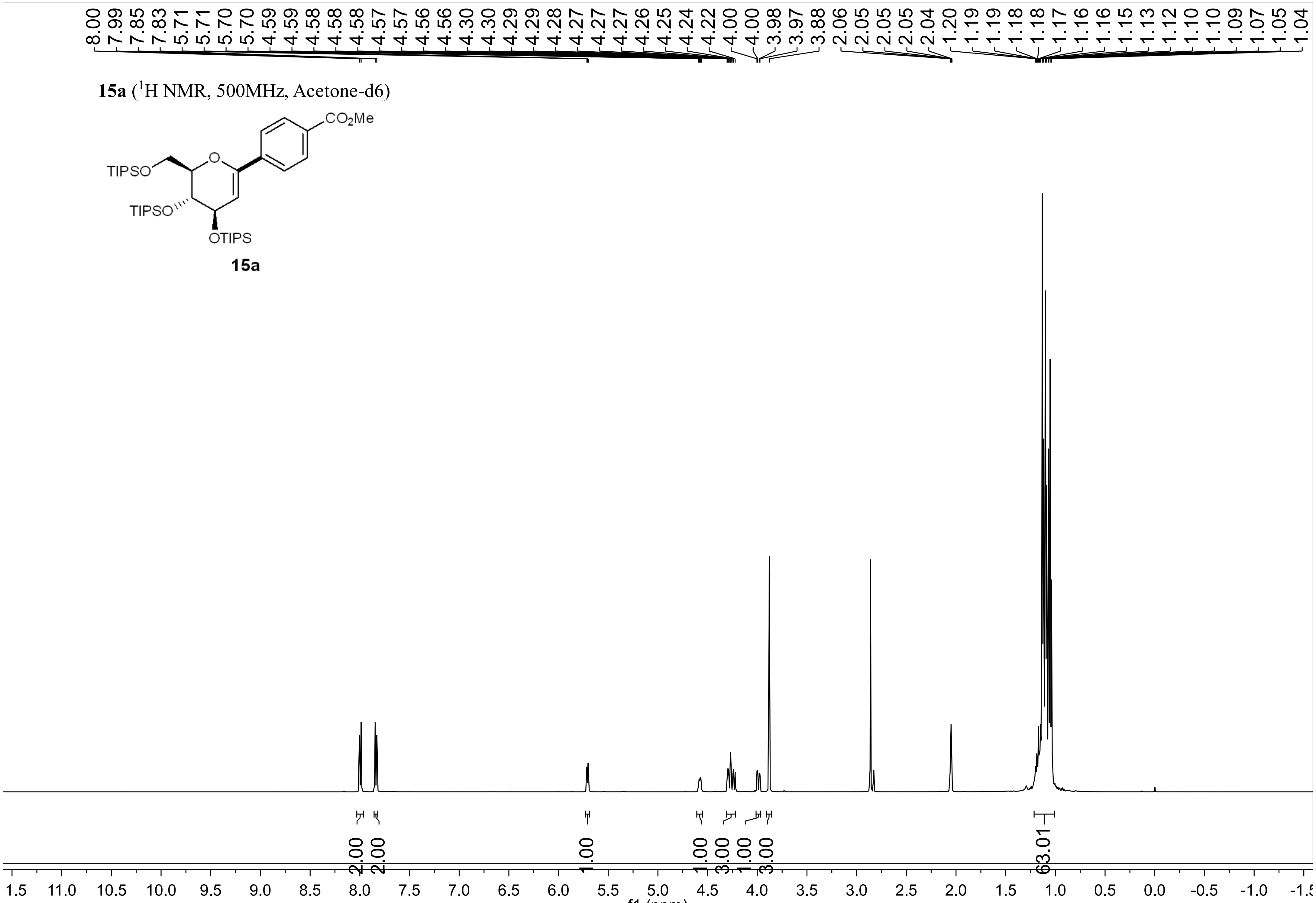

Supplementary Figure 120. ^{1}H NMR spectrum of compound **15a**

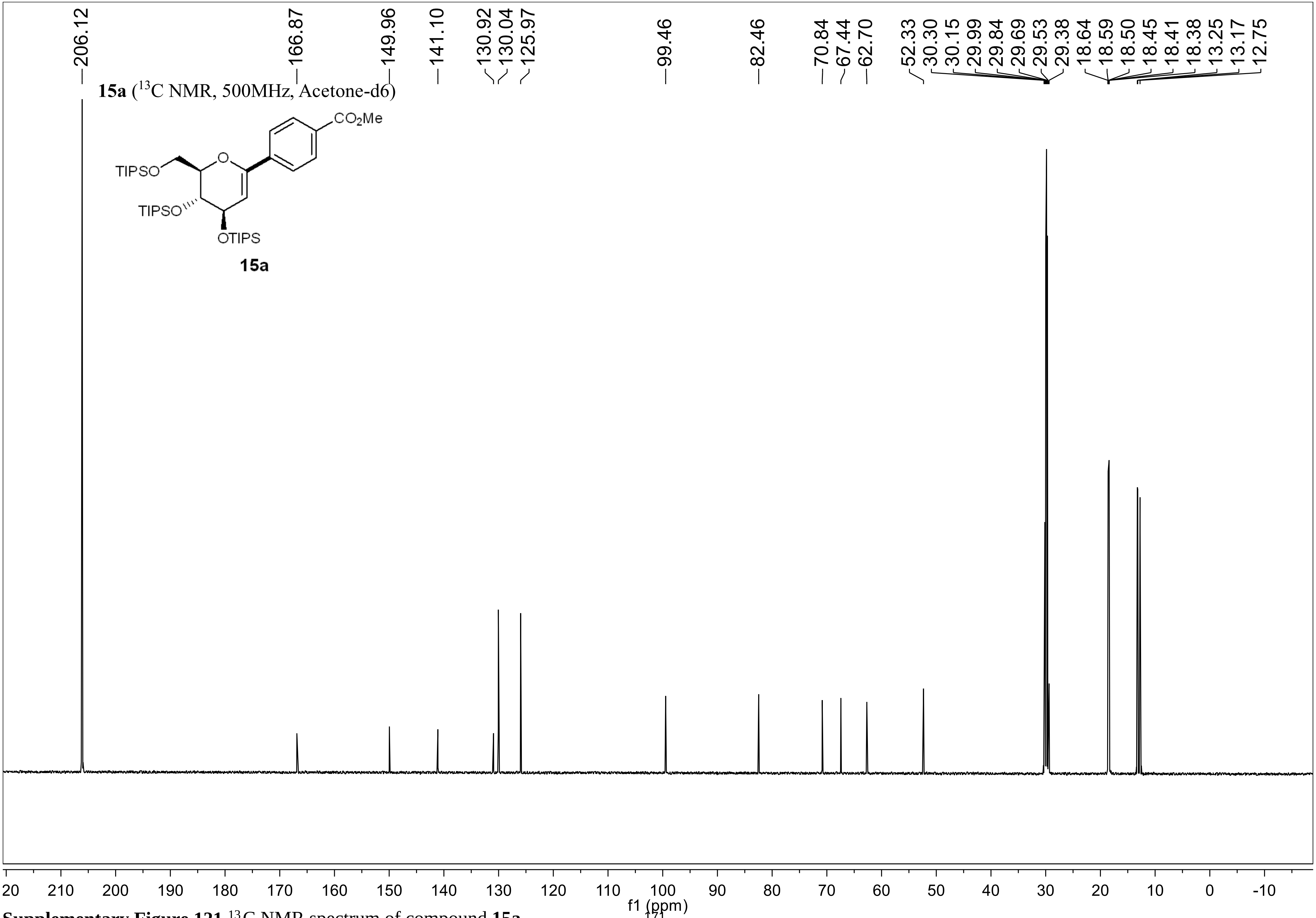

Supplementary Figure 121. ^{13}C NMR spectrum of compound **15a**

15b (^{1}H NMR, 400MHz, $CDCl_3$)

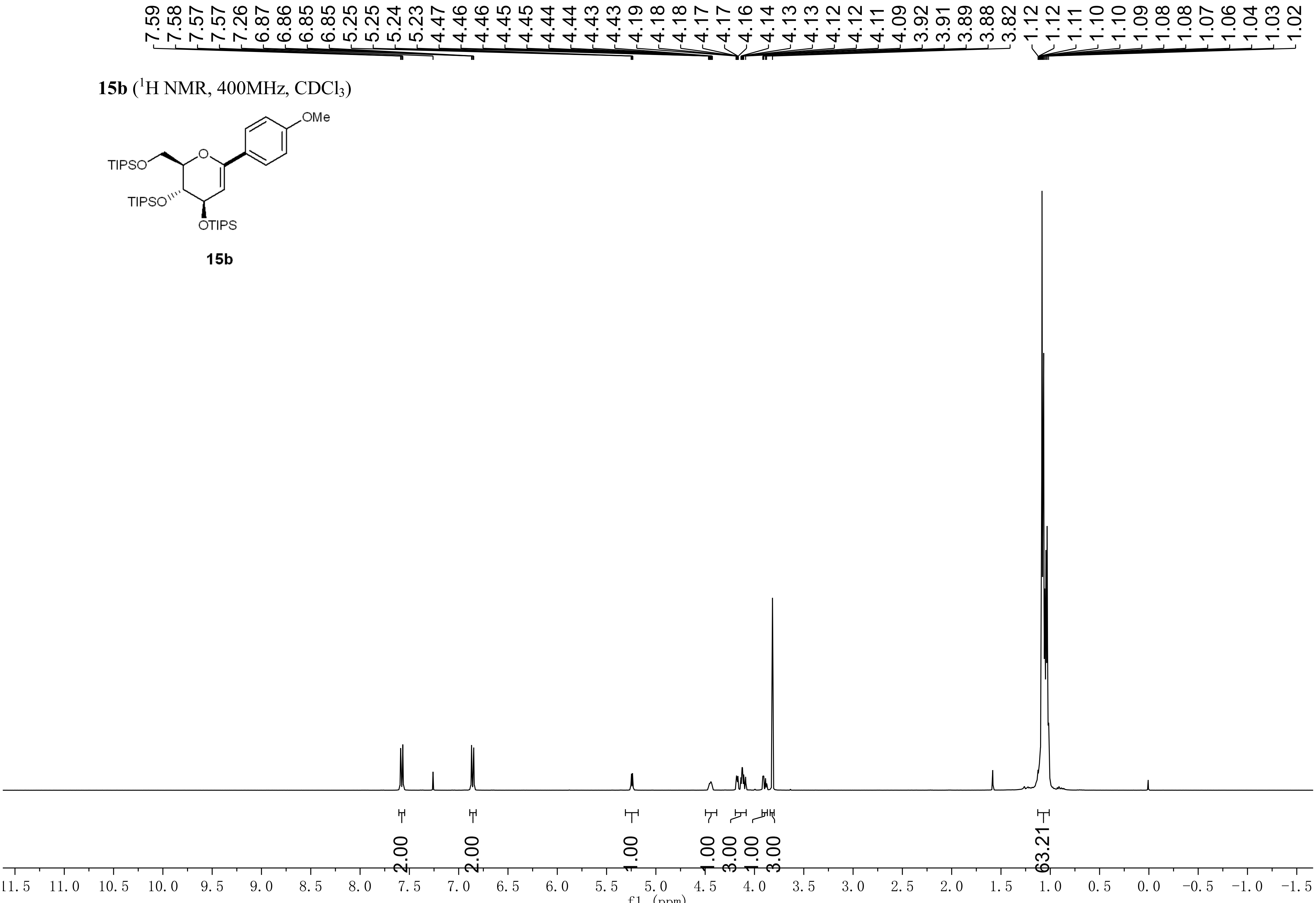

15b

Supplementary Figure 122. ^{1}H NMR spectrum of compound **15b**

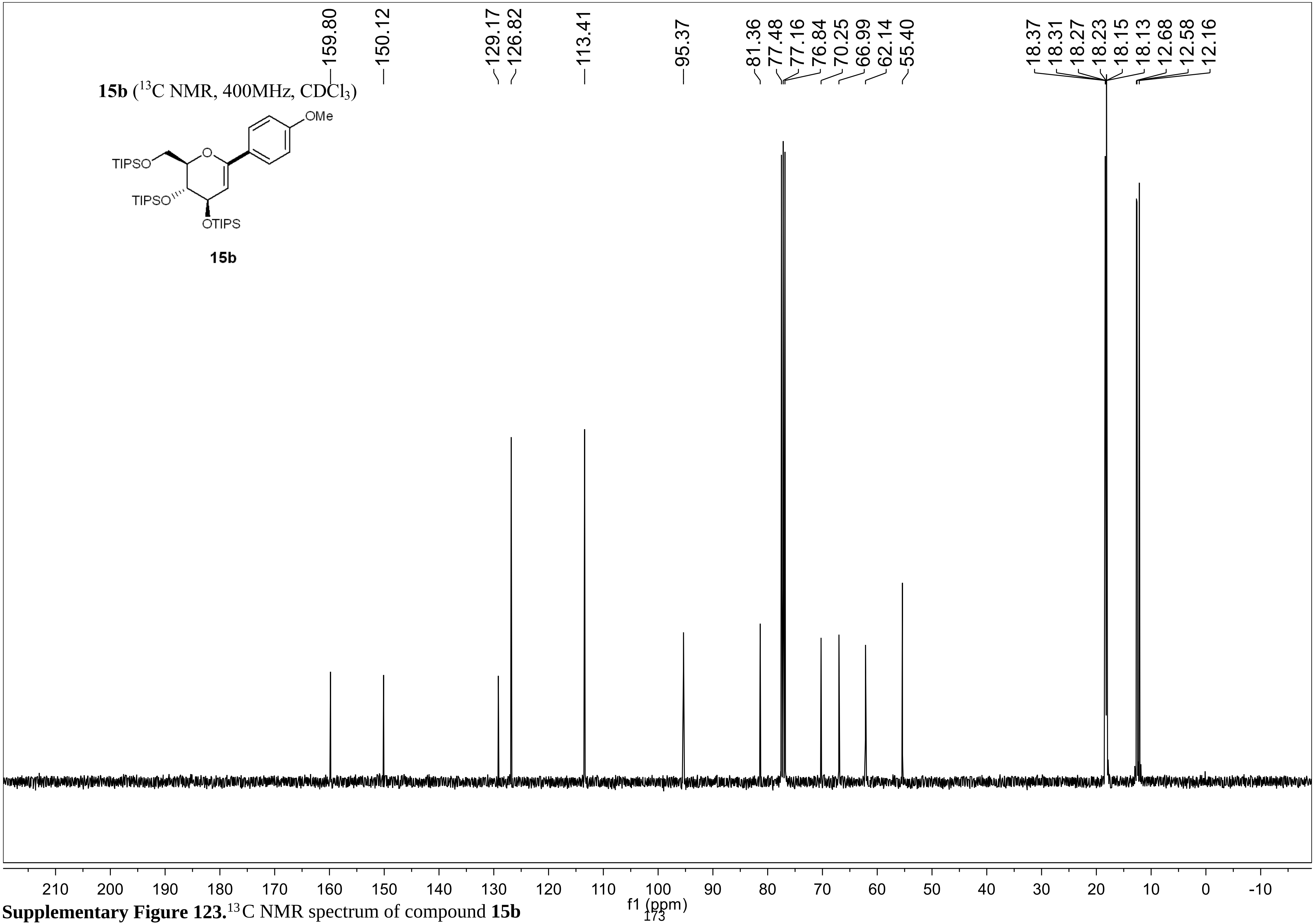

Supplementary Figure 123. ^{13}C NMR spectrum of compound **15b**

15c (^{1}H NMR, 400MHz, Acetone-d6)

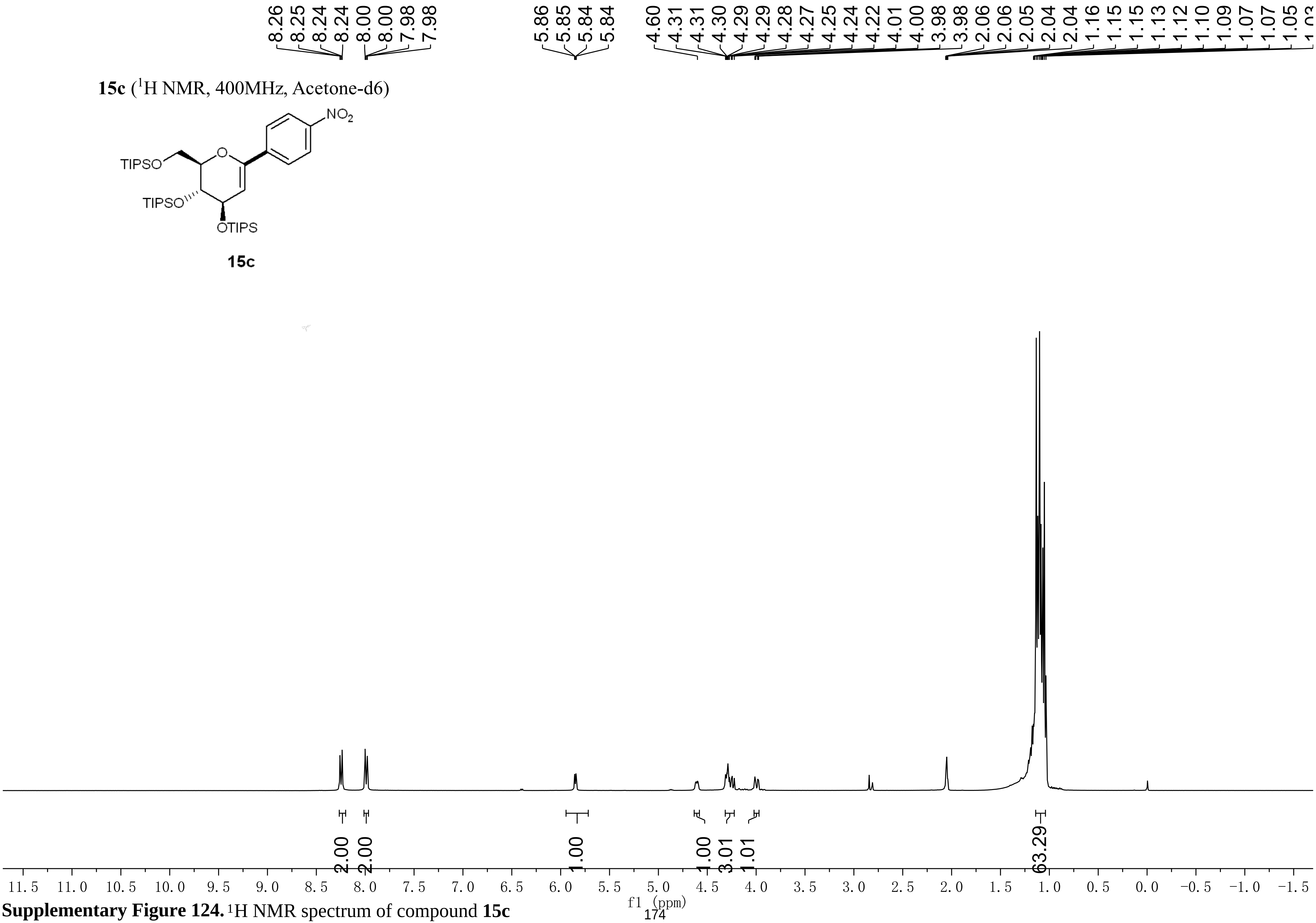

15c

Supplementary Figure 124. ^{1}H NMR spectrum of compound **15c**

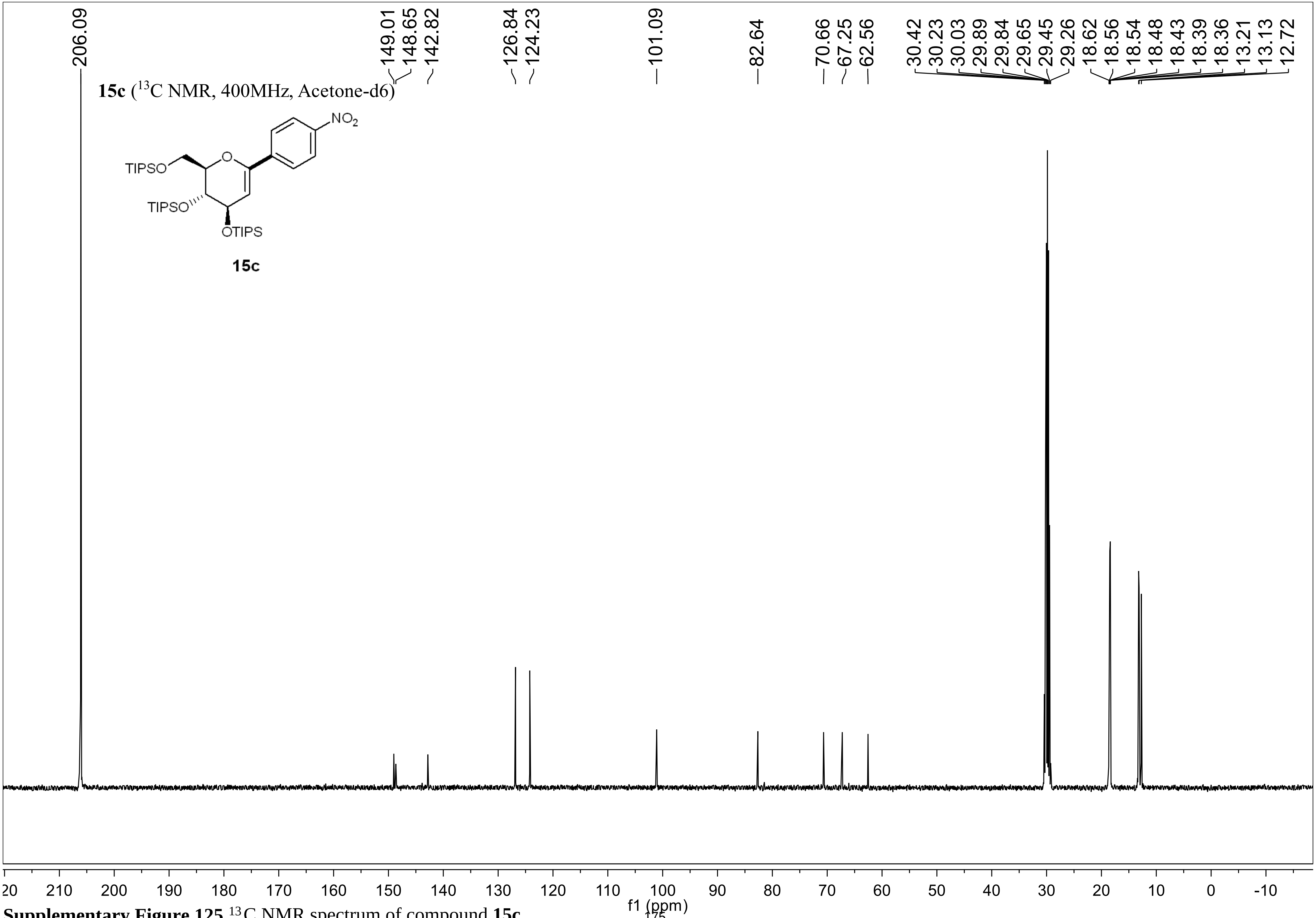

Supplementary Figure 125. ^{13}C NMR spectrum of compound **15c**

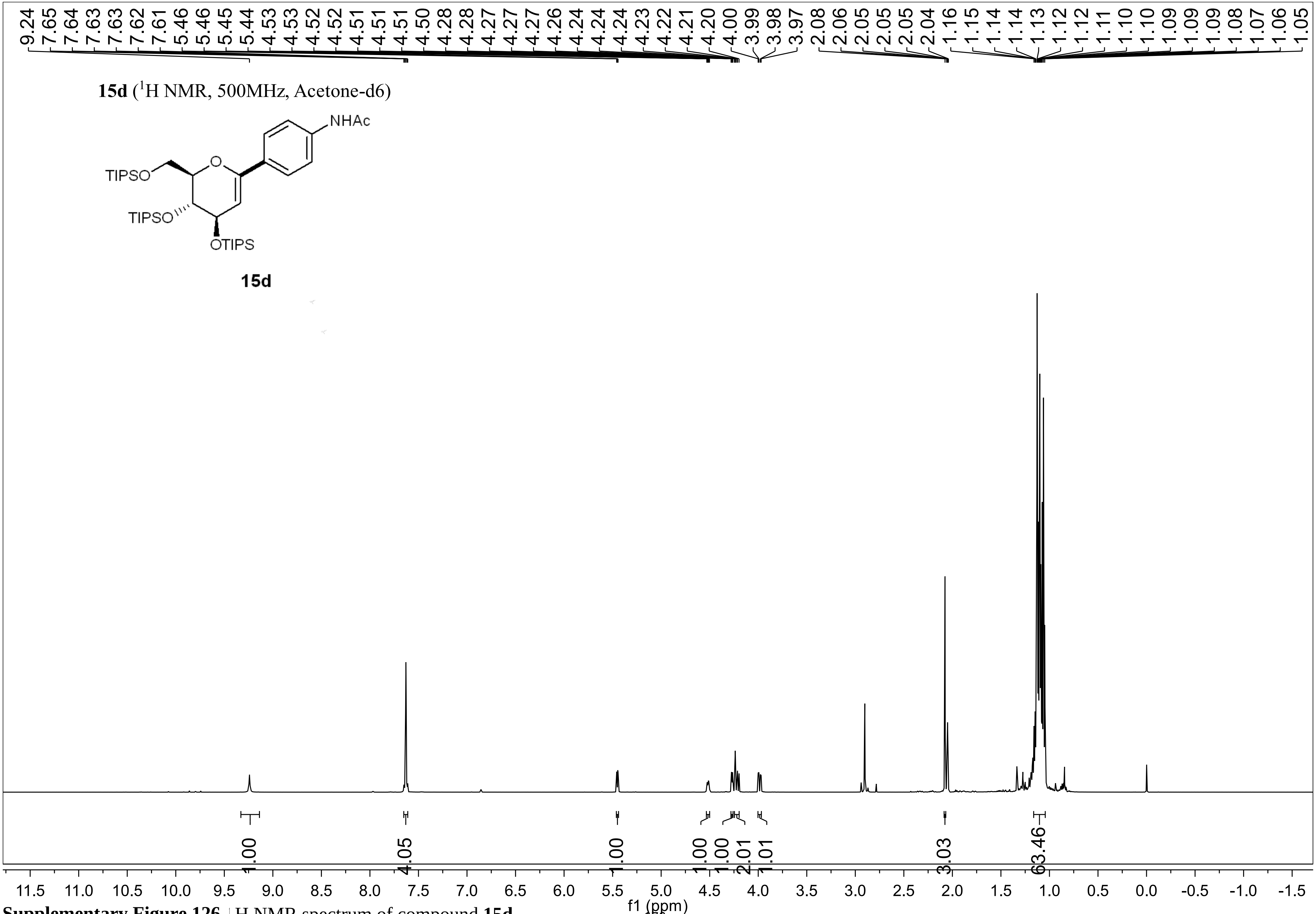

Supplementary Figure 126. ^{1}H NMR spectrum of compound **15d**

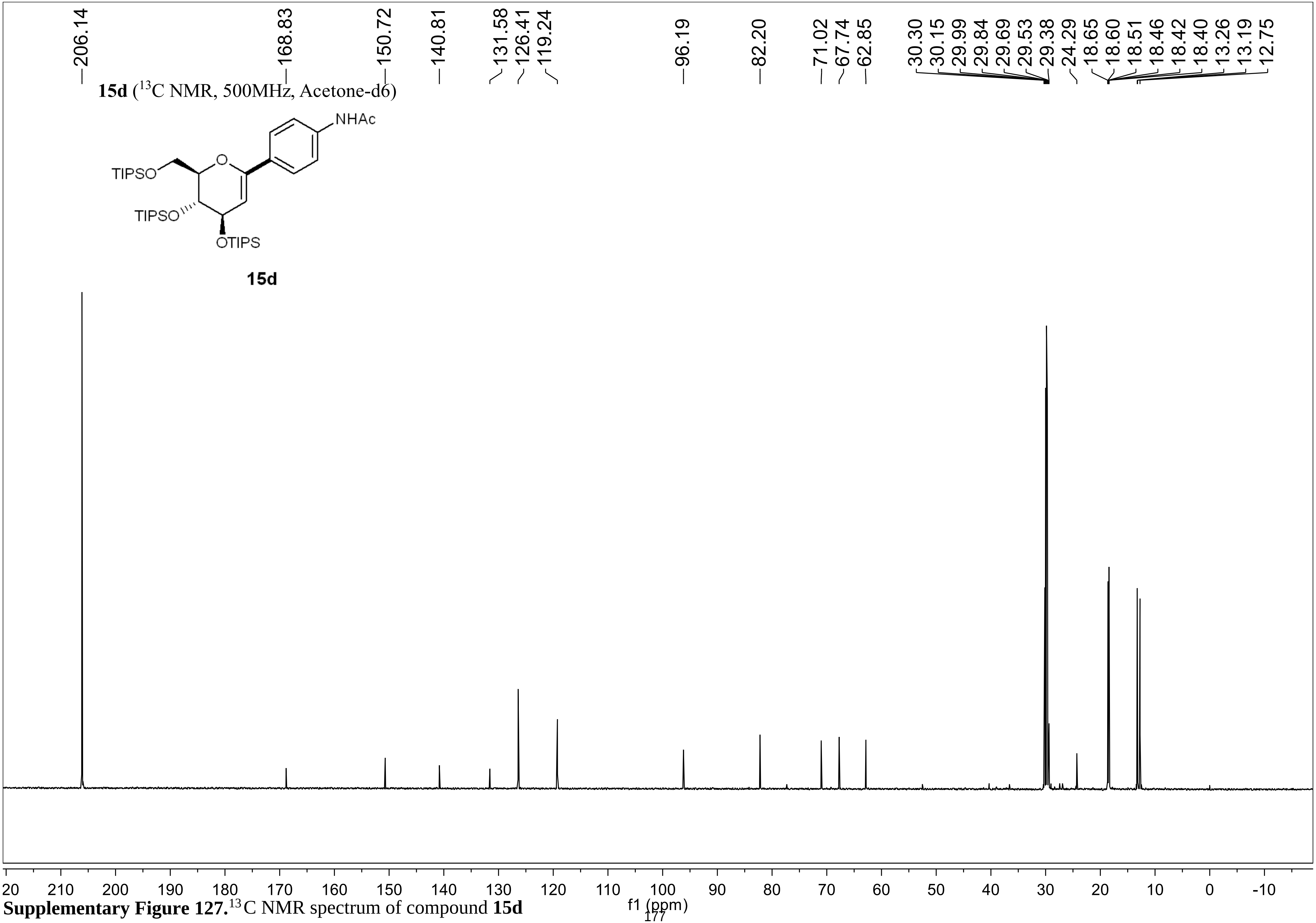

Supplementary Figure 127. ^{13}C NMR spectrum of compound **15d**

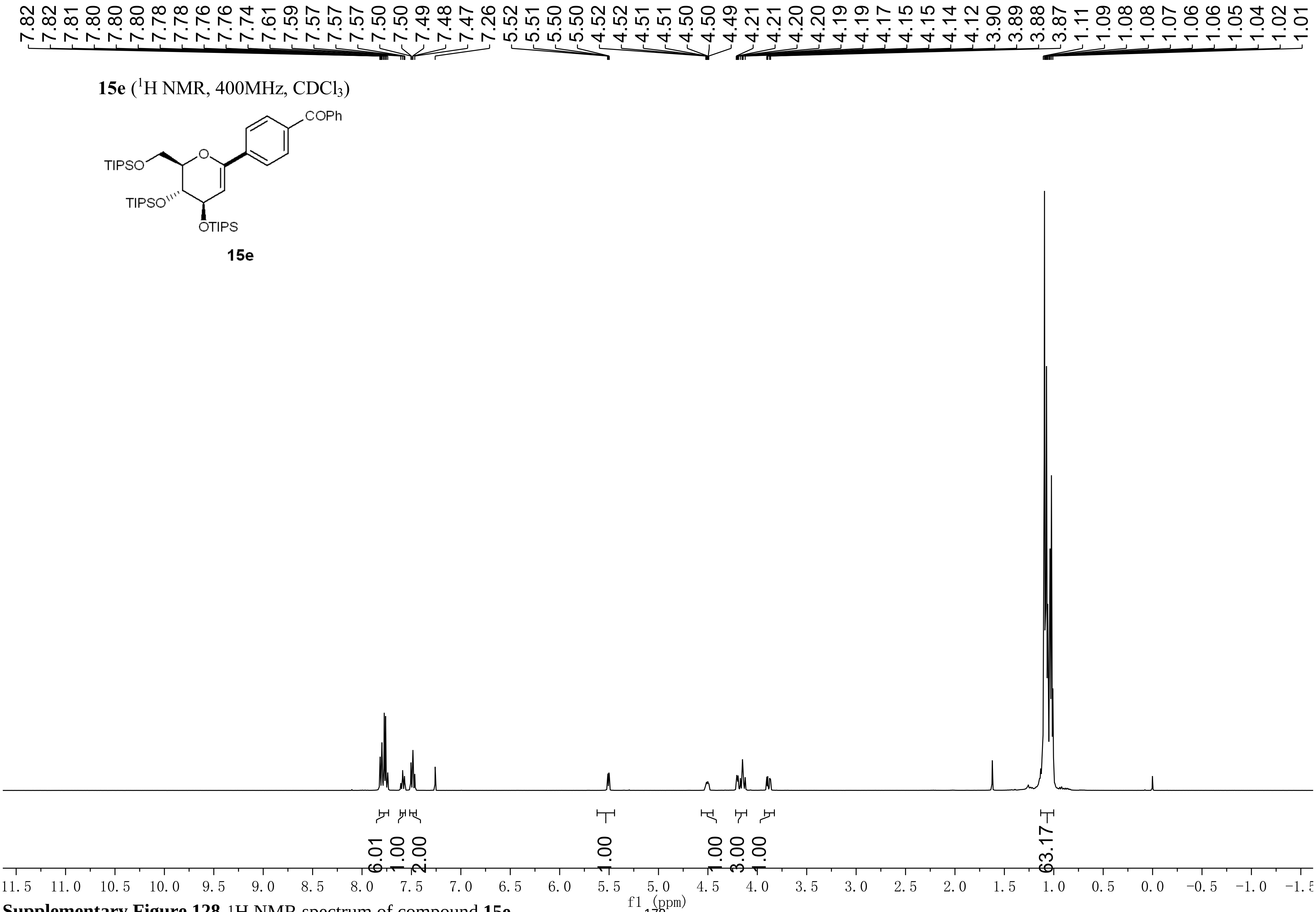

Supplementary Figure 128. ^{1}H NMR spectrum of compound **15e**

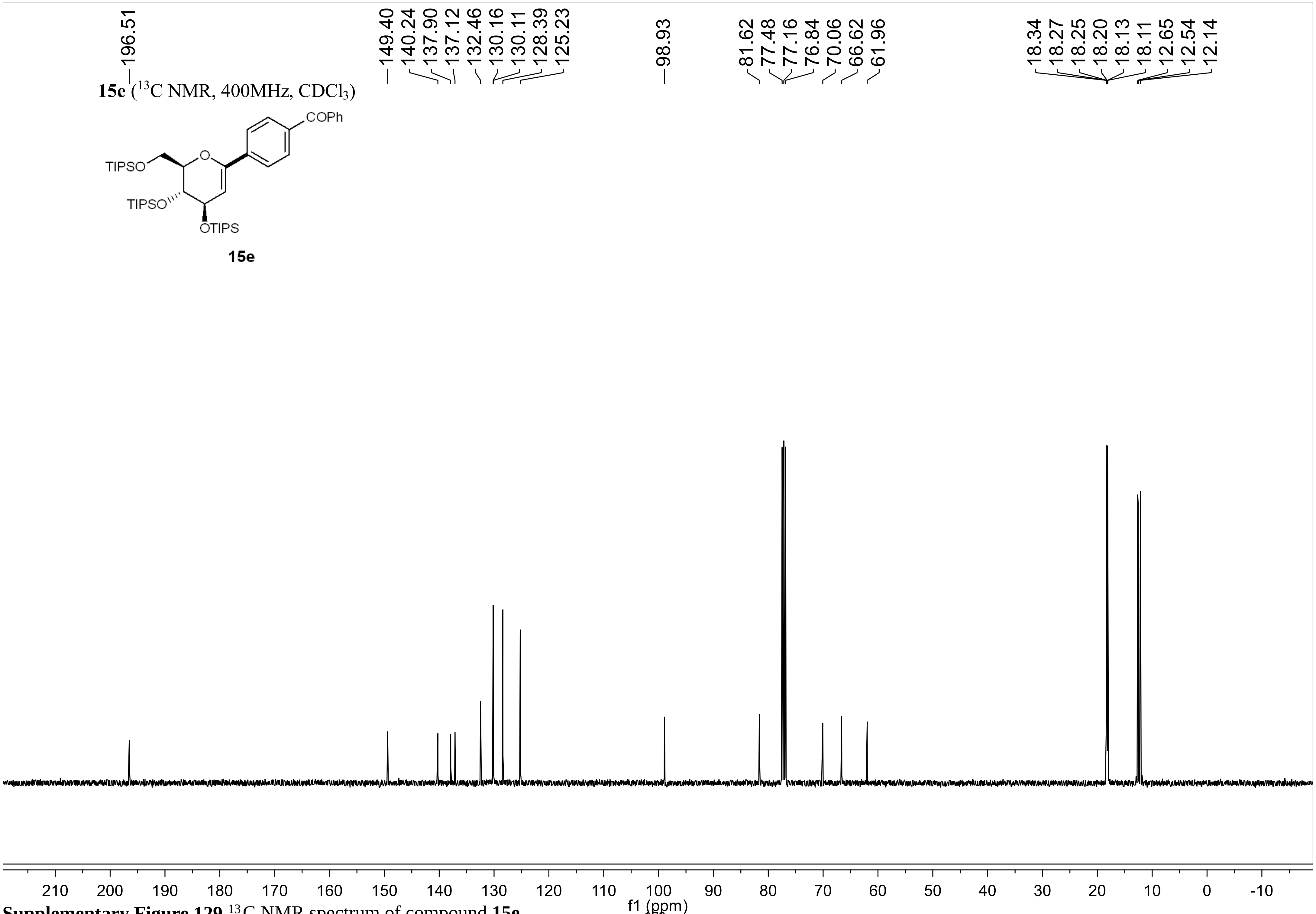

Supplementary Figure 129. ^{13}C NMR spectrum of compound **15e**

15f (^{1}H NMR, 400MHz, $CDCl_3$)

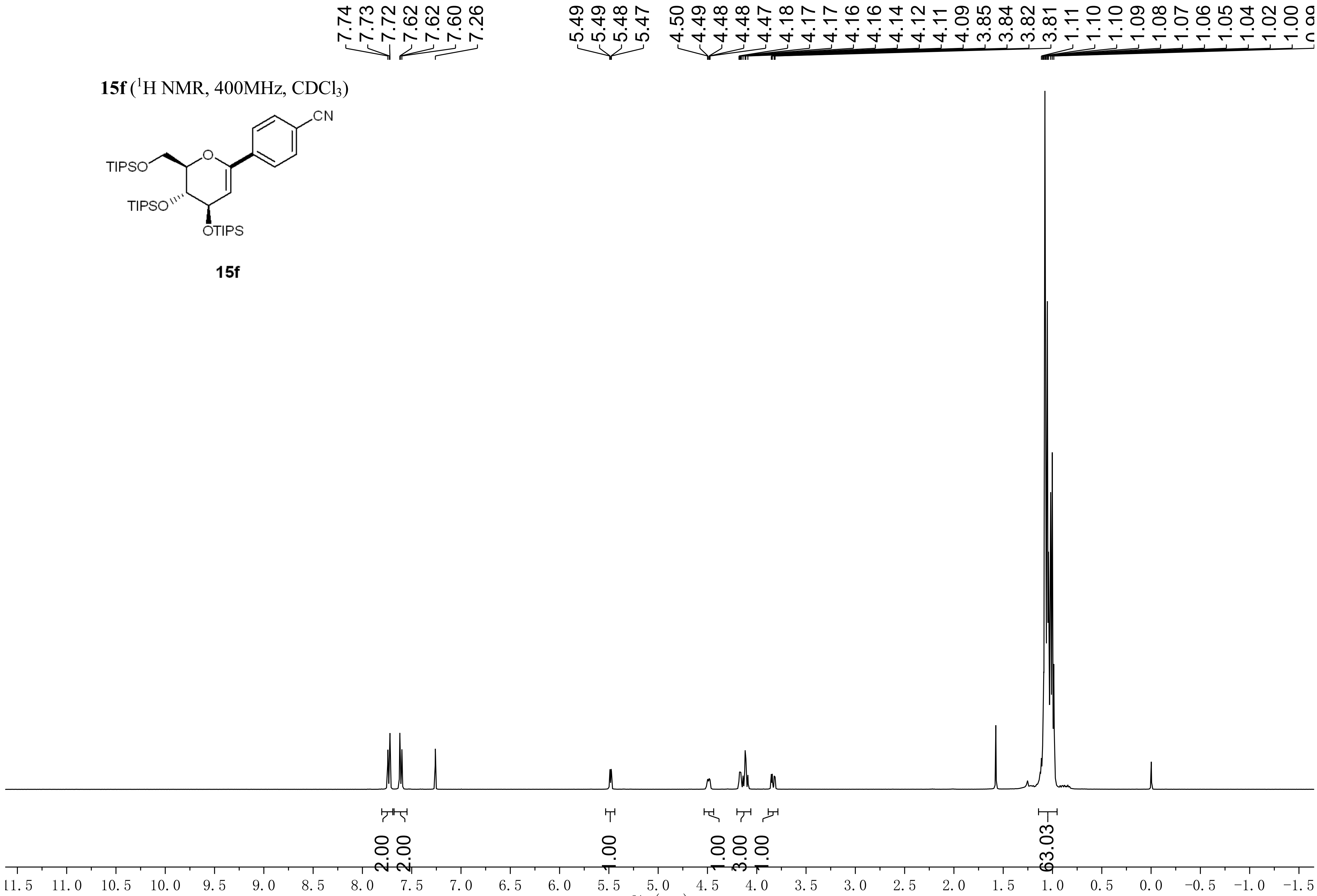

Supplementary Figure 130. ^{1}H NMR spectrum of compound **15f**

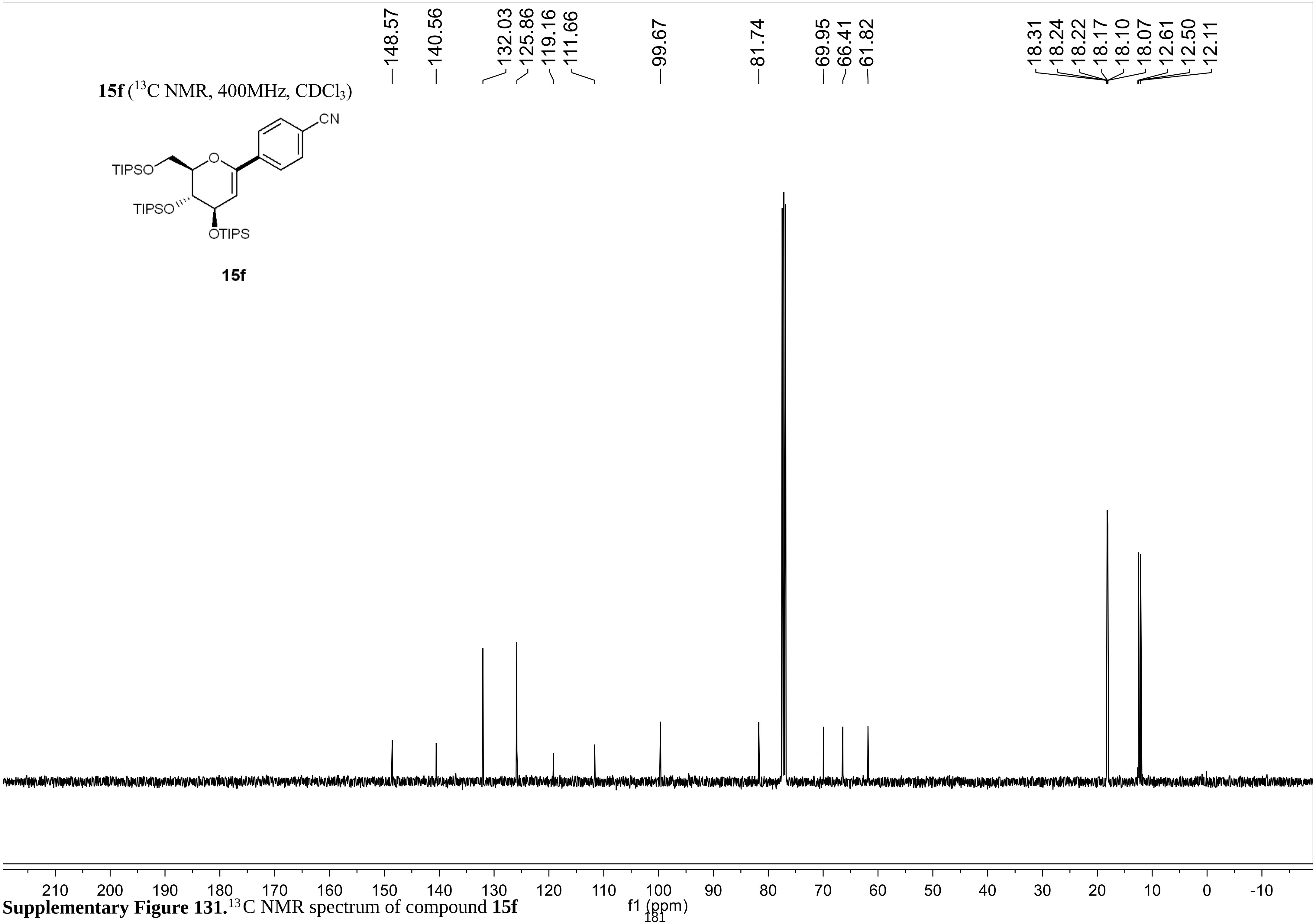

Supplementary Figure 131. ^{13}C NMR spectrum of compound **15f**

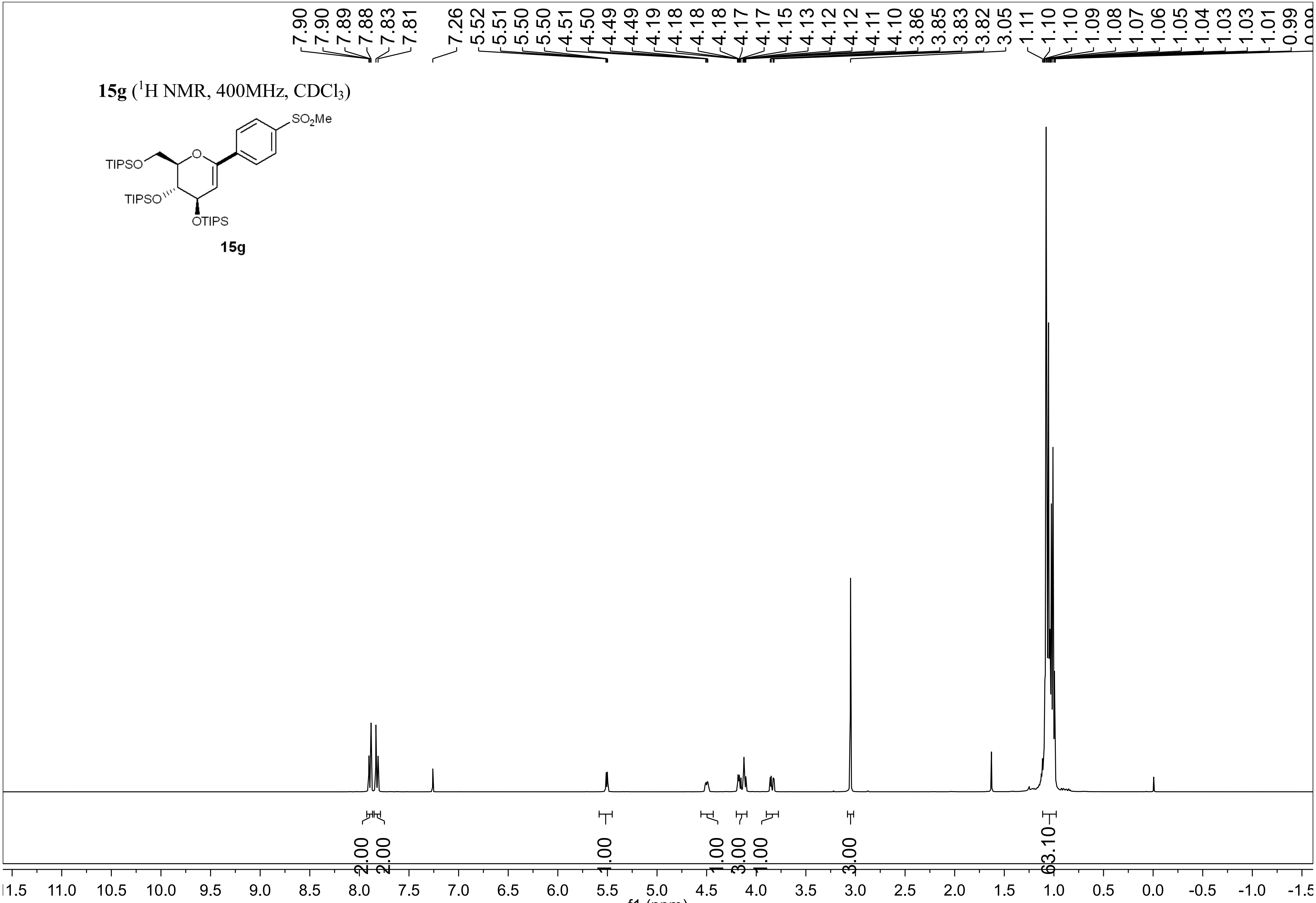

Supplementary Figure 132. ^{1}H NMR spectrum of compound **15g**

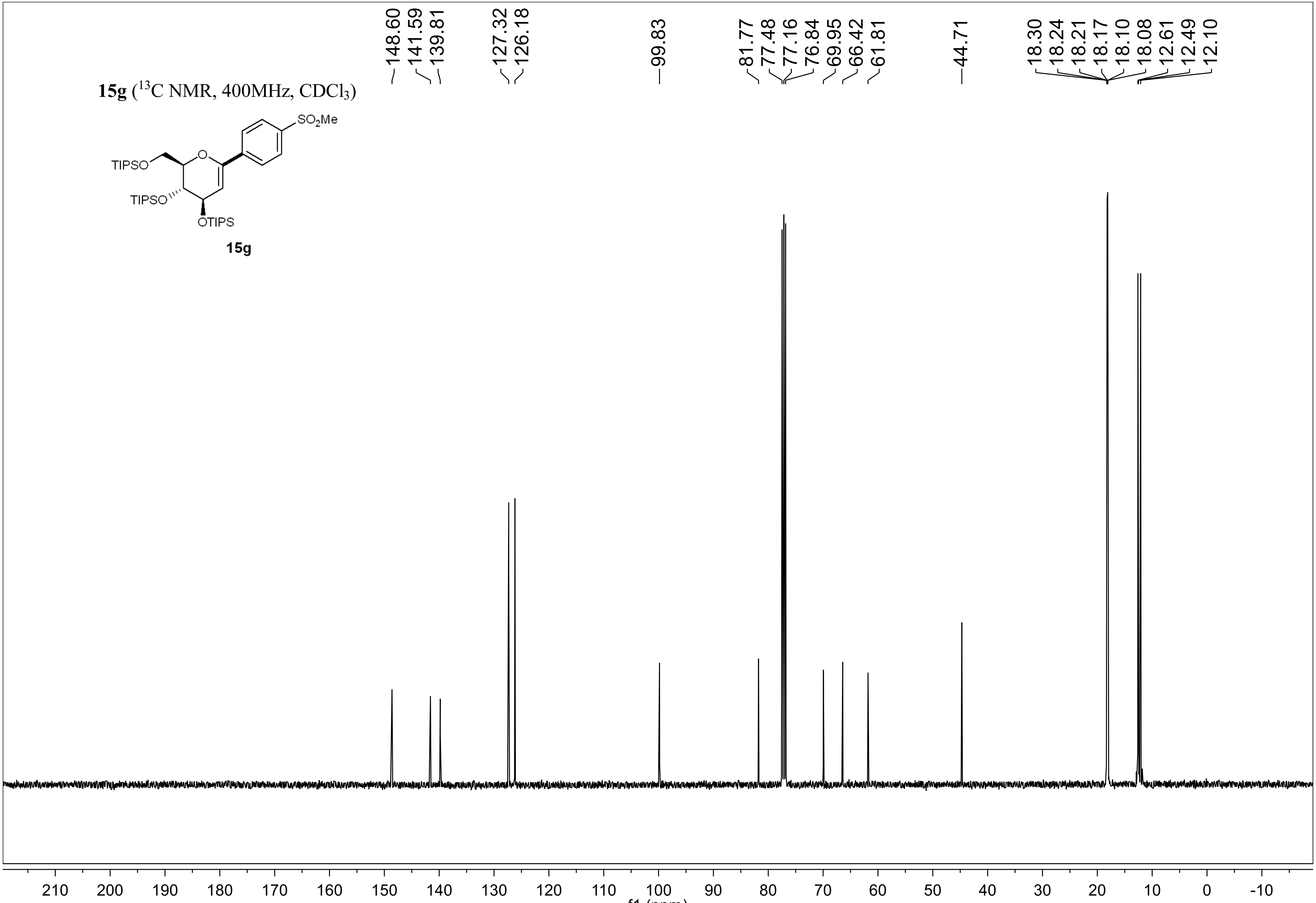

Supplementary Figure 133. ^{13}C NMR spectrum of compound **15g**

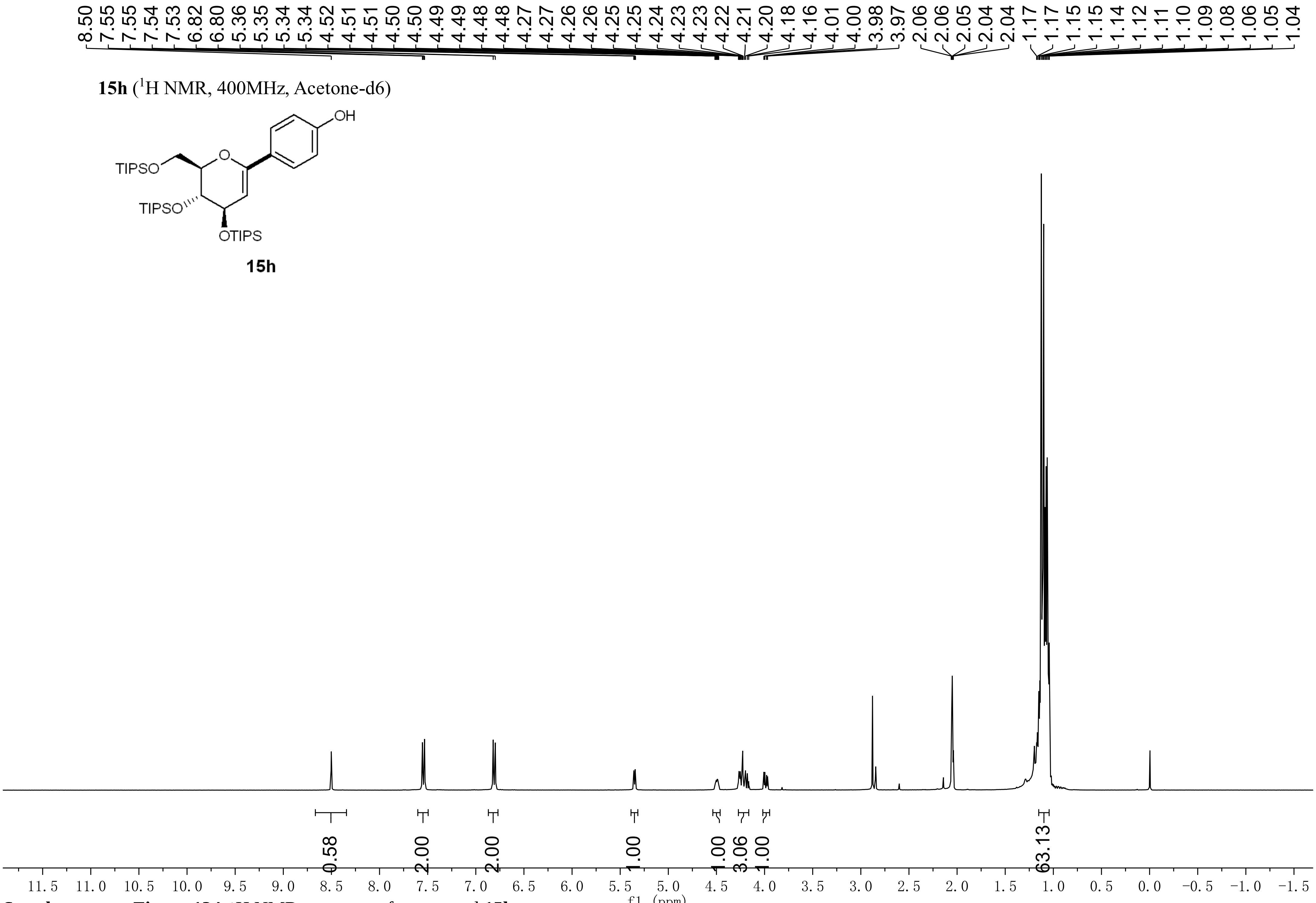

Supplementary Figure 134. 1H NMR spectrum of compound **15h**

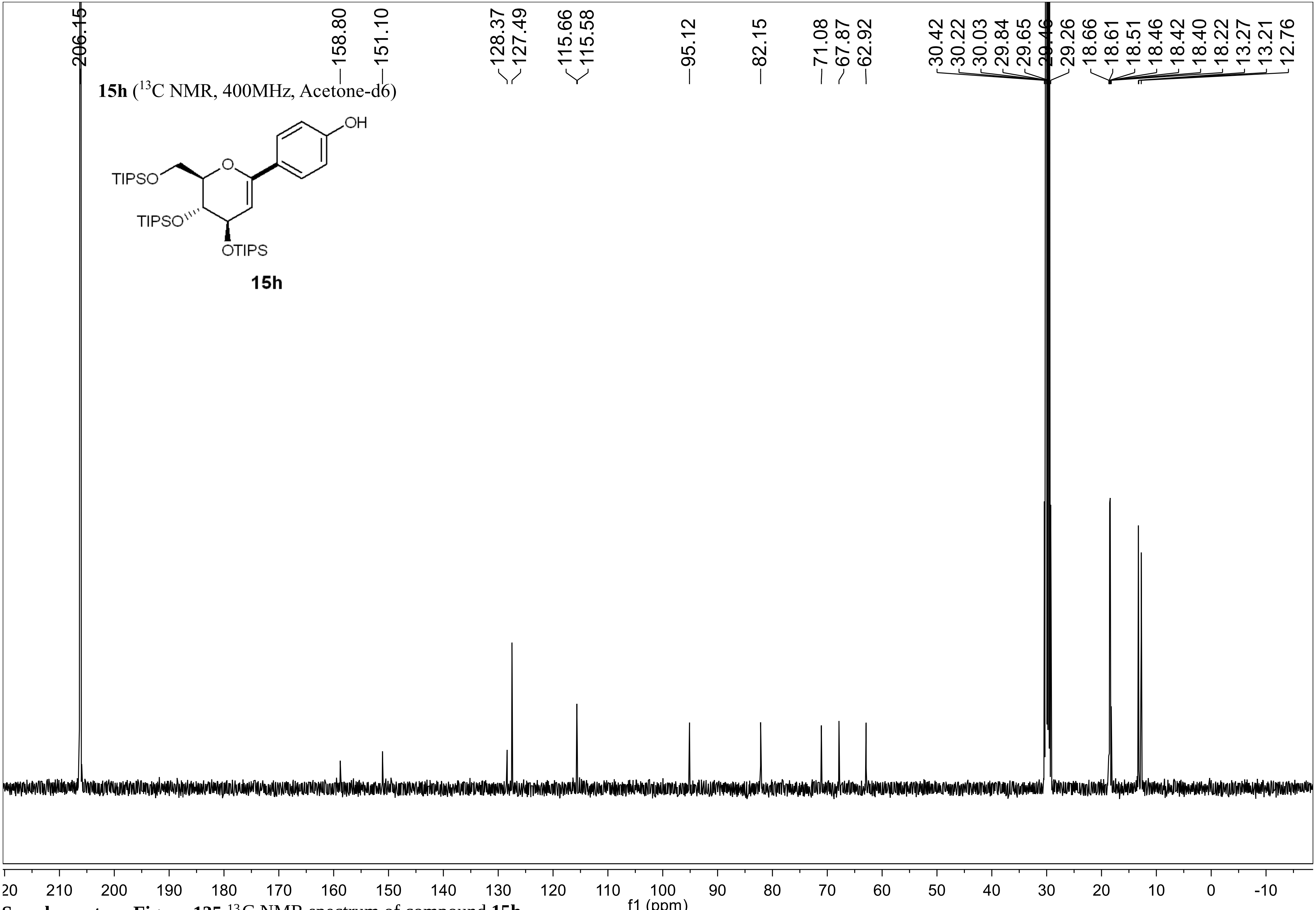

Supplementary Figure 135. ^{13}C NMR spectrum of compound **15h**

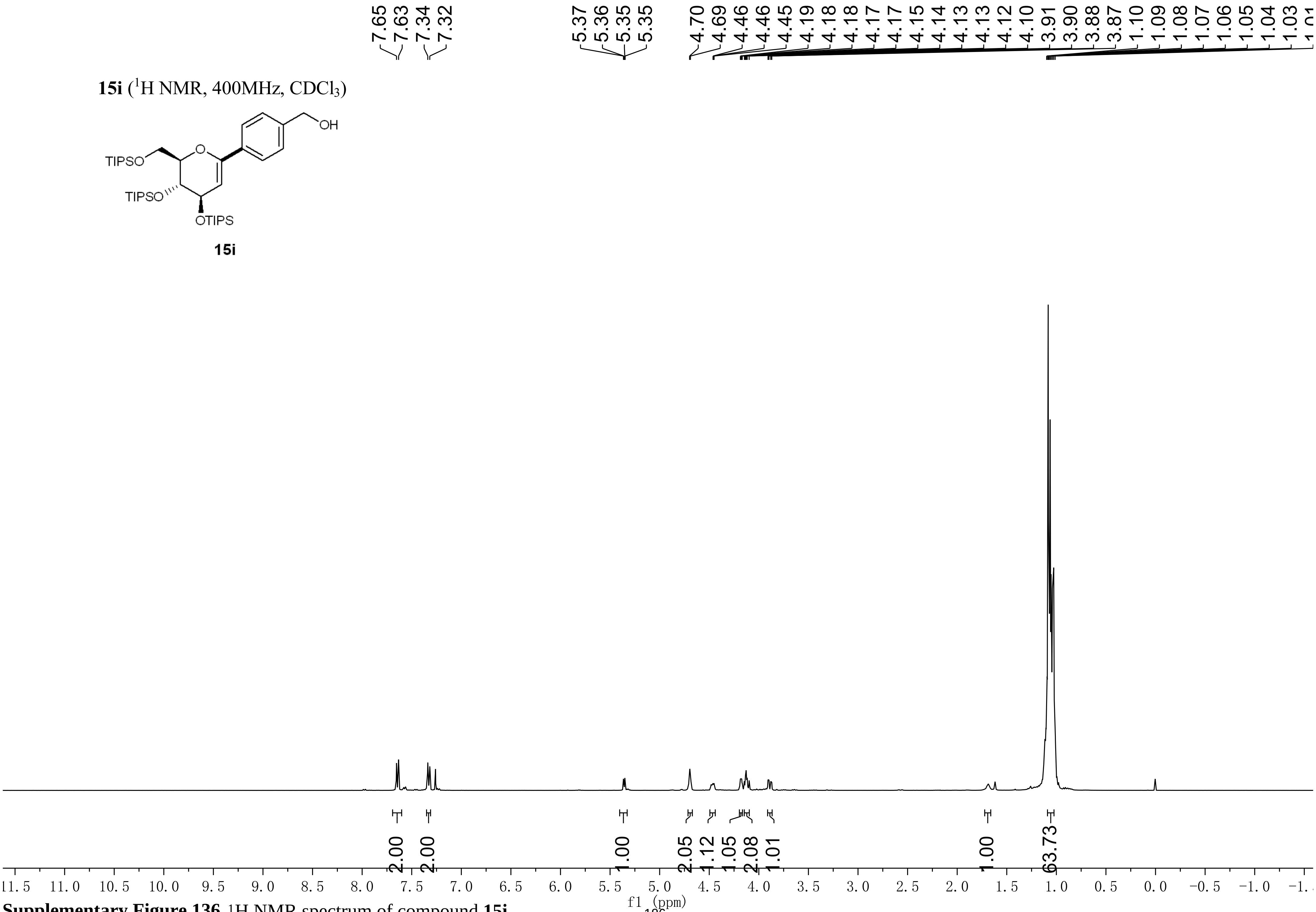

Supplementary Figure 136. ^{1}H NMR spectrum of compound **15i**

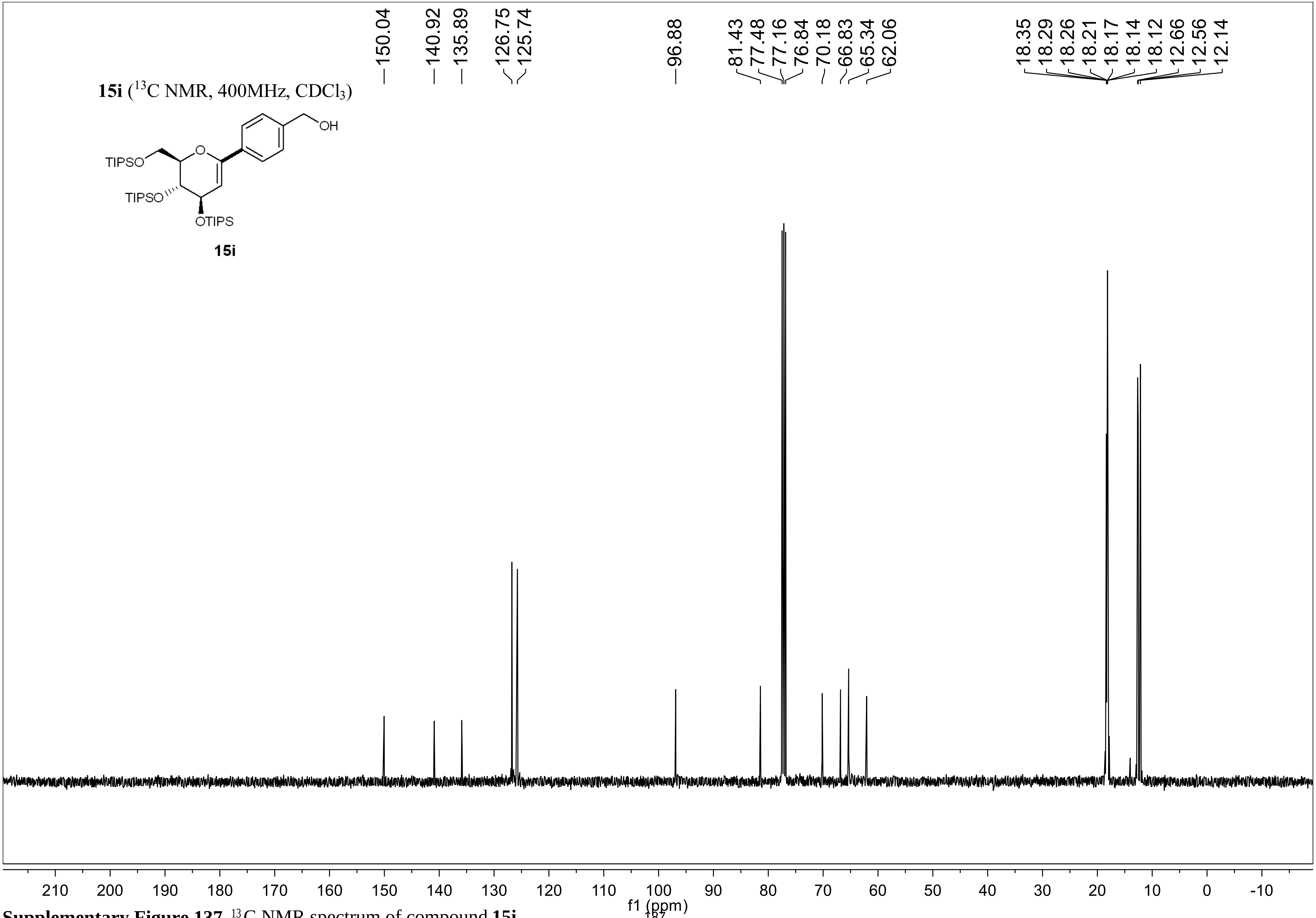

Supplementary Figure 137. ^{13}C NMR spectrum of compound **15i**

15j (^{1}H NMR, 400MHz, $CDCl_3$)

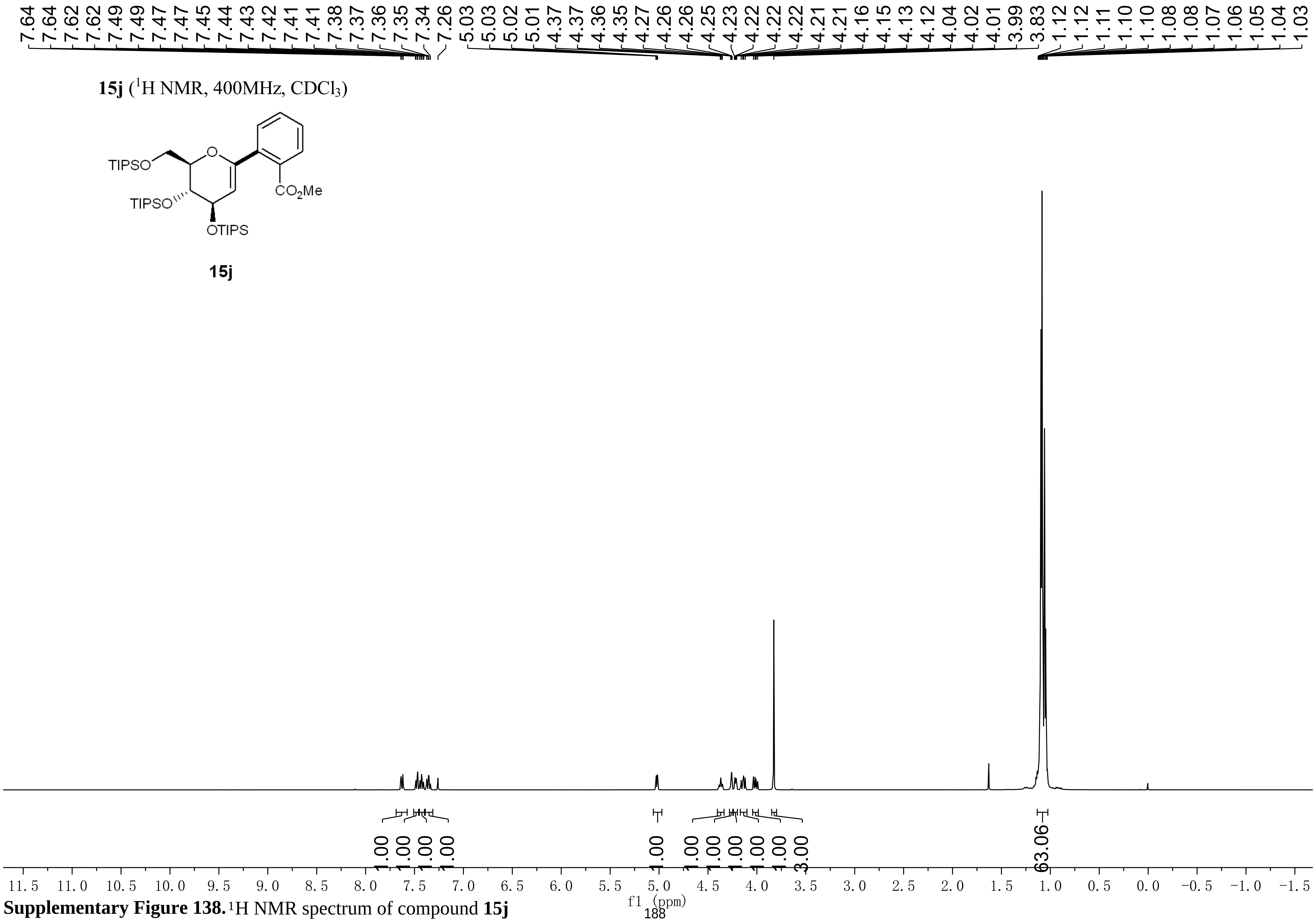

Supplementary Figure 138. ^{1}H NMR spectrum of compound **15j**

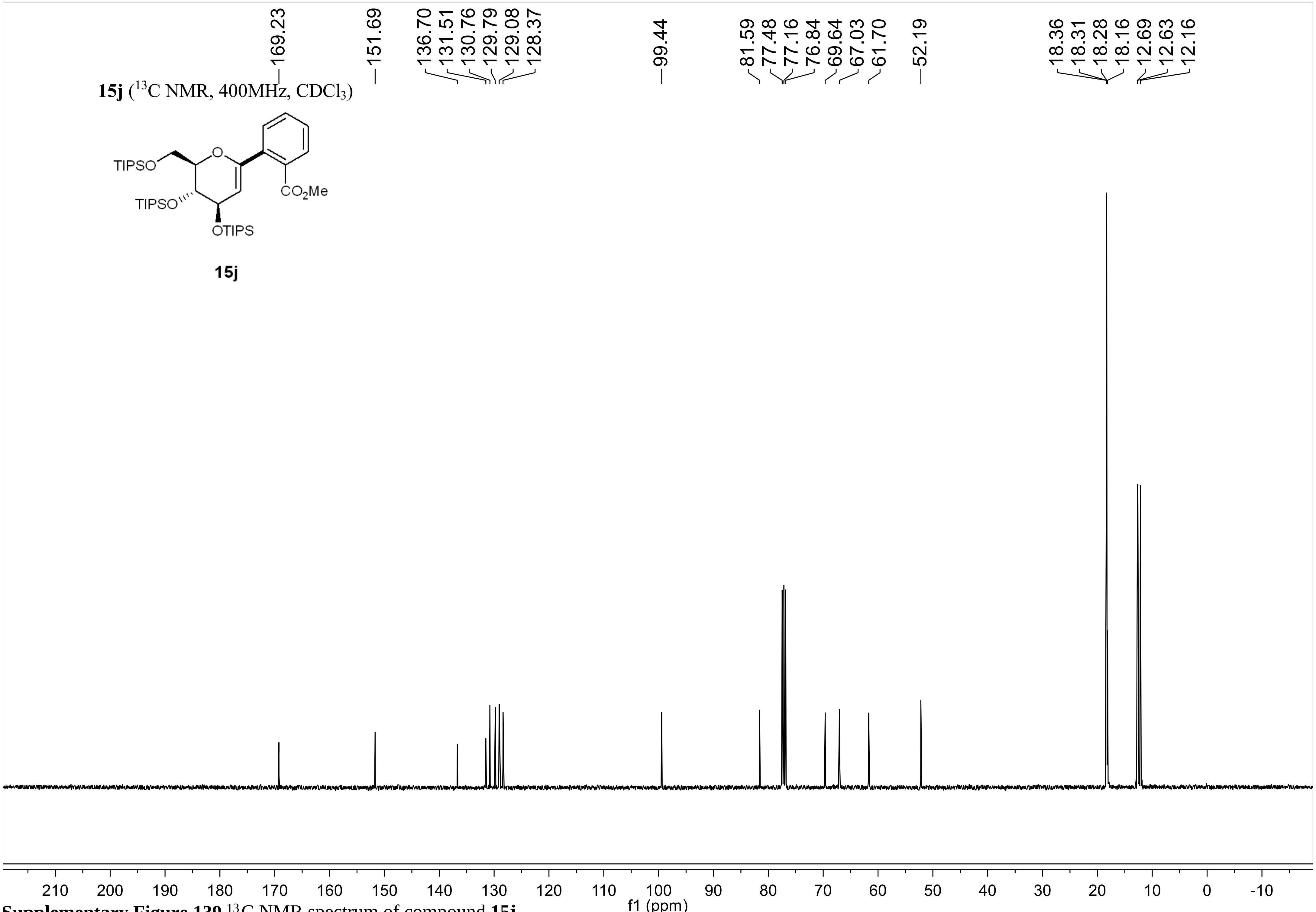

Supplementary Figure 139. ^{13}C NMR spectrum of compound **15j**

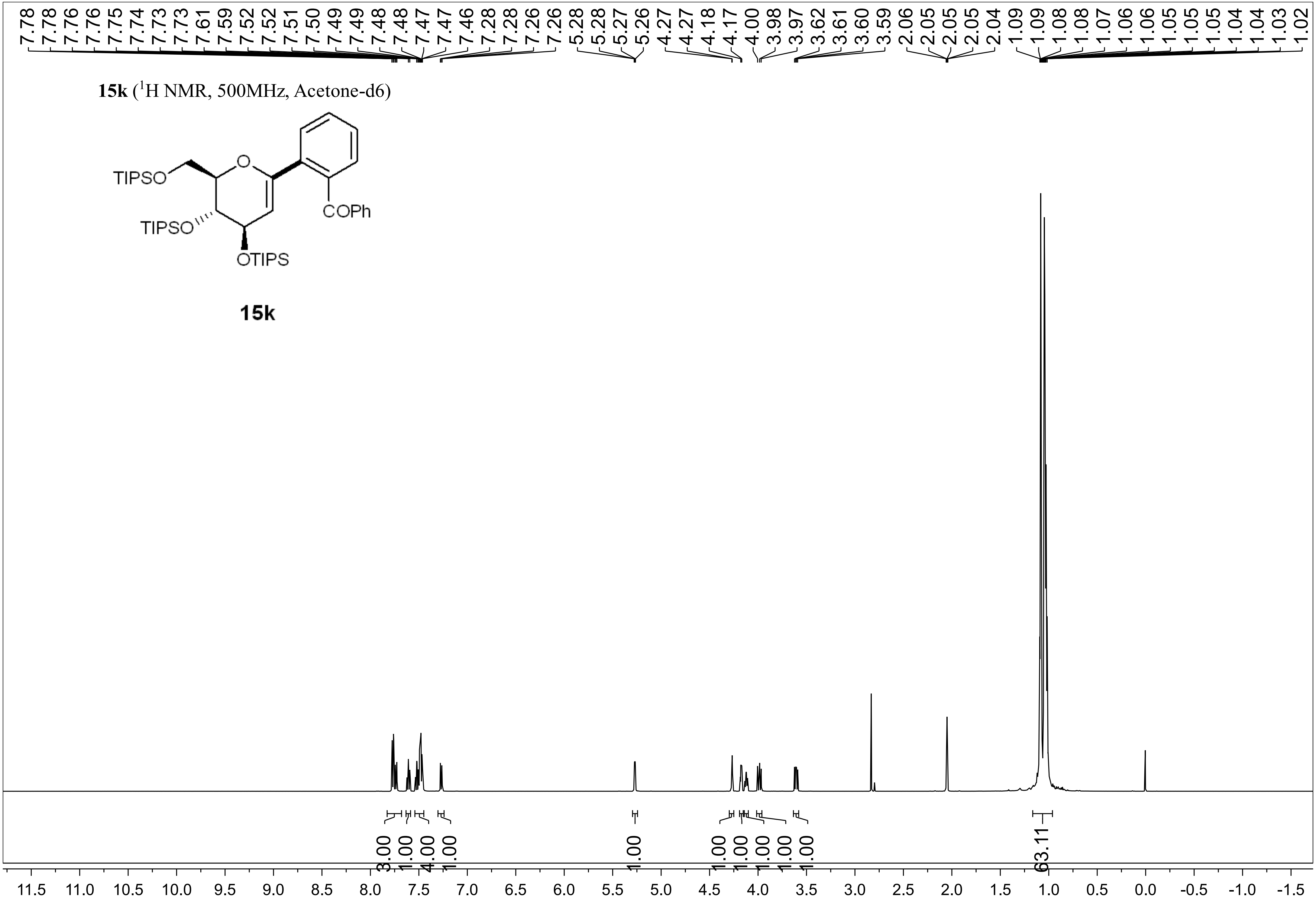

Supplementary Figure 140. ^{1}H NMR spectrum of compound **15k**

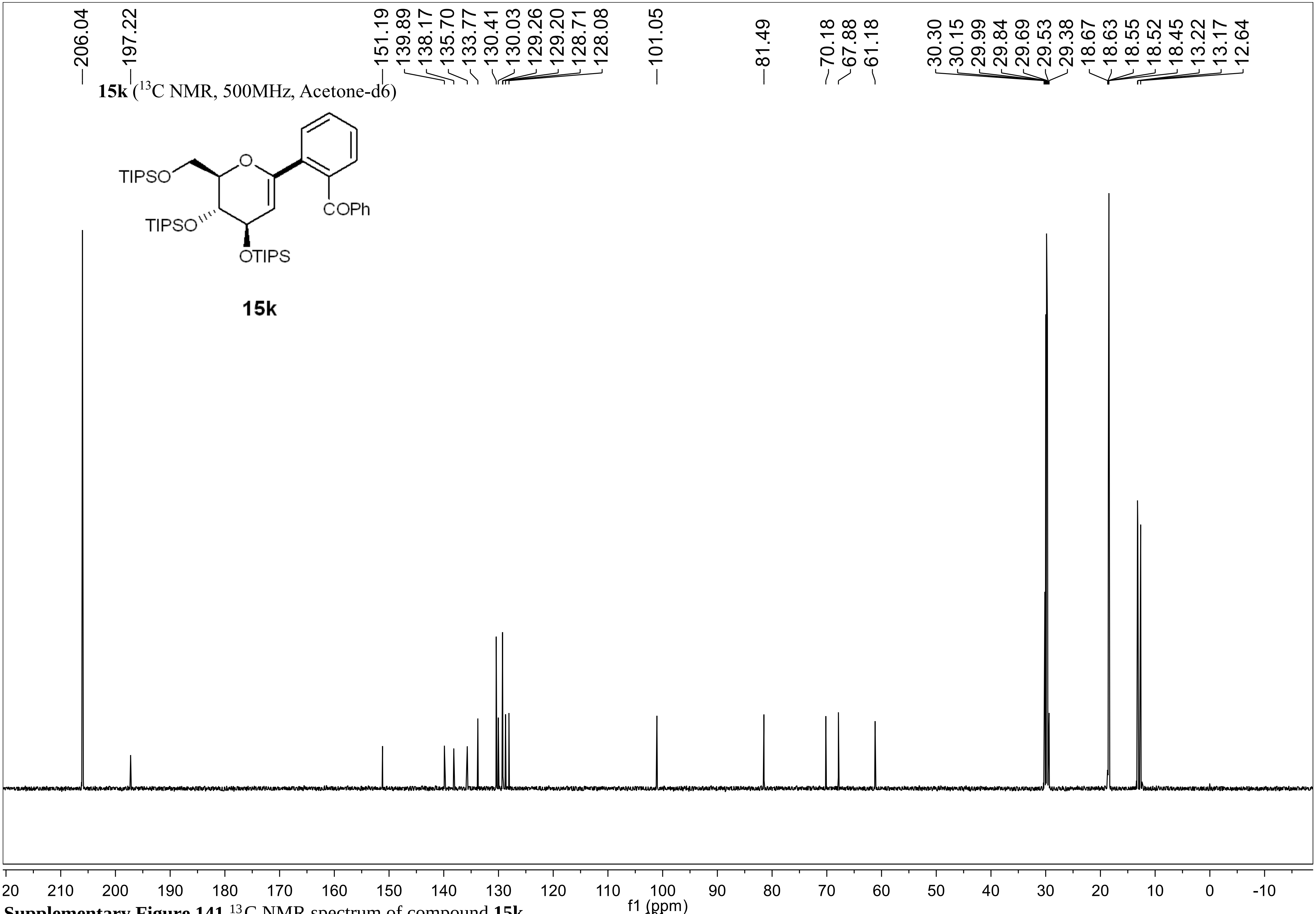

Supplementary Figure 141. ^{13}C NMR spectrum of compound **15k**

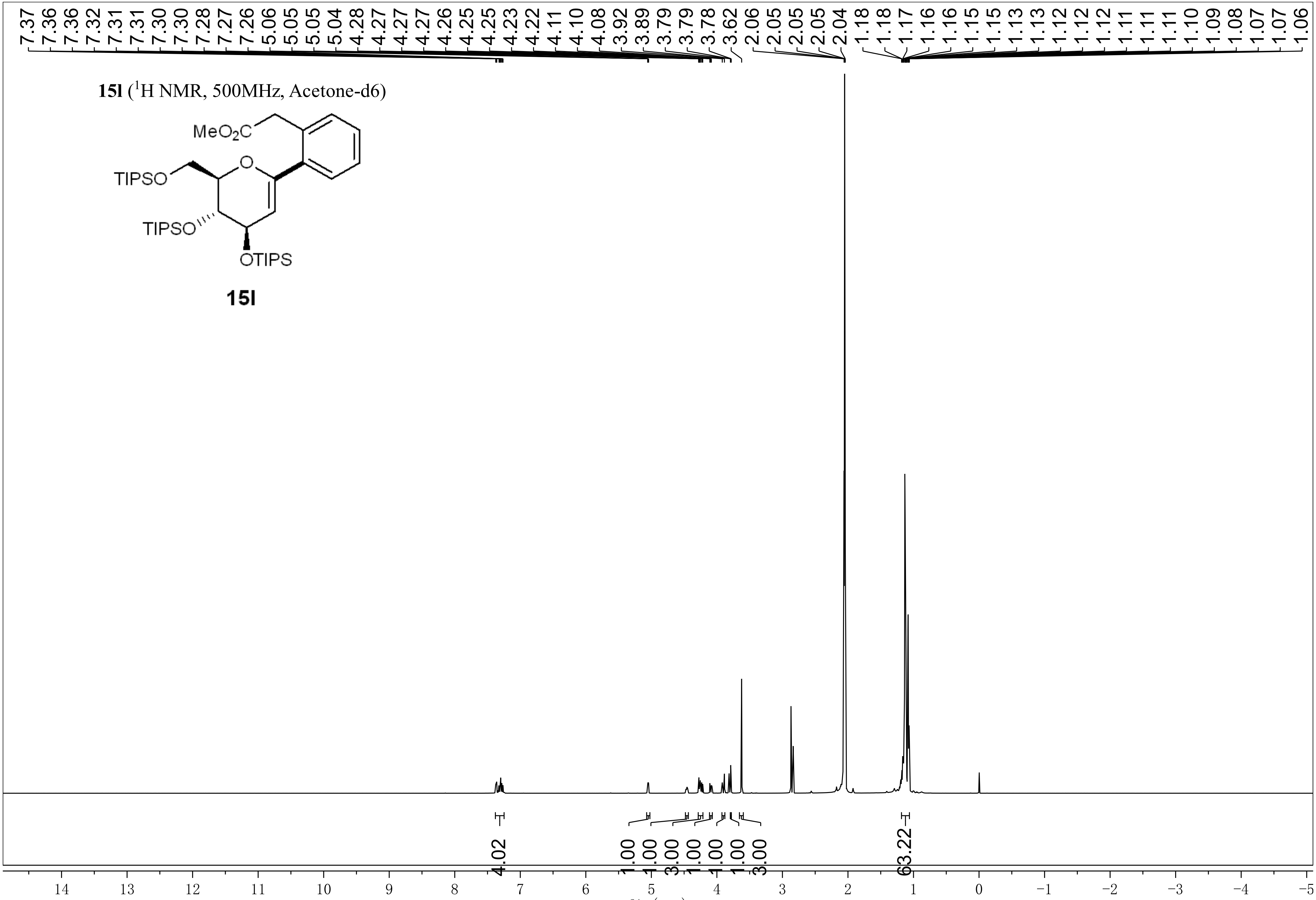

Supplementary Figure 142. ^{1}H NMR spectrum of compound **15l**

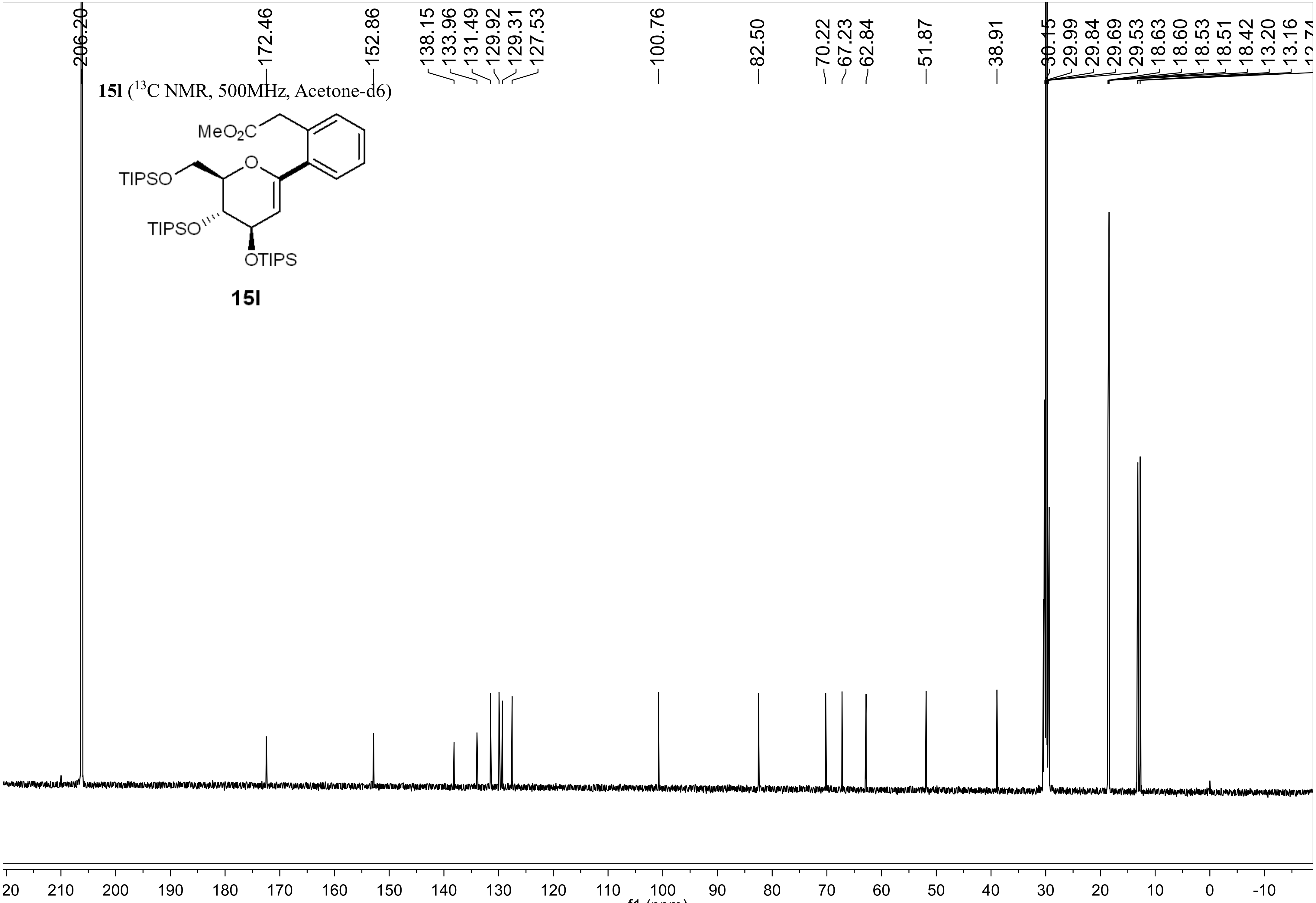

Supplementary Figure 143. ^{13}C NMR spectrum of compound **15l**

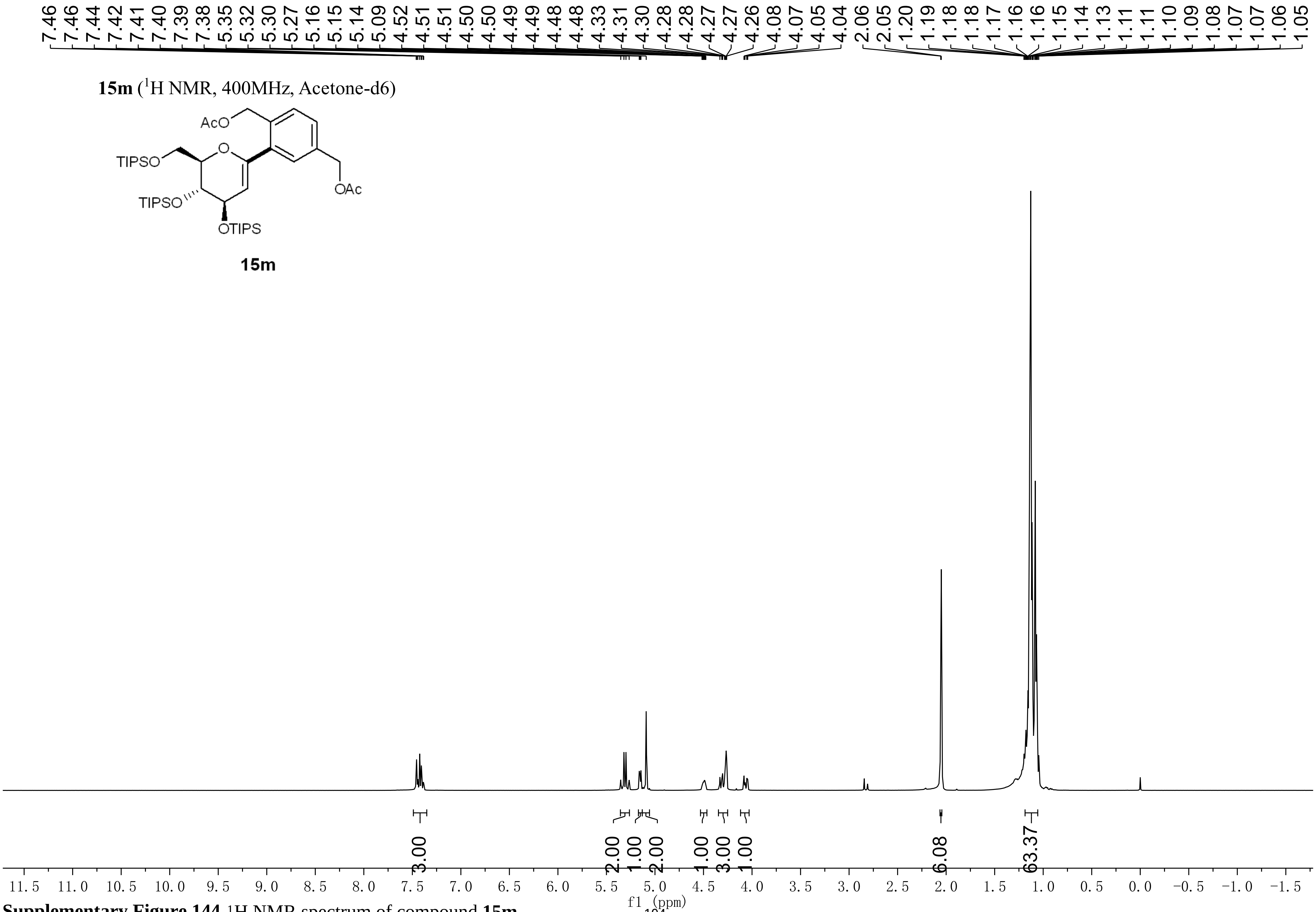

Supplementary Figure 144. 1H NMR spectrum of compound **15m**

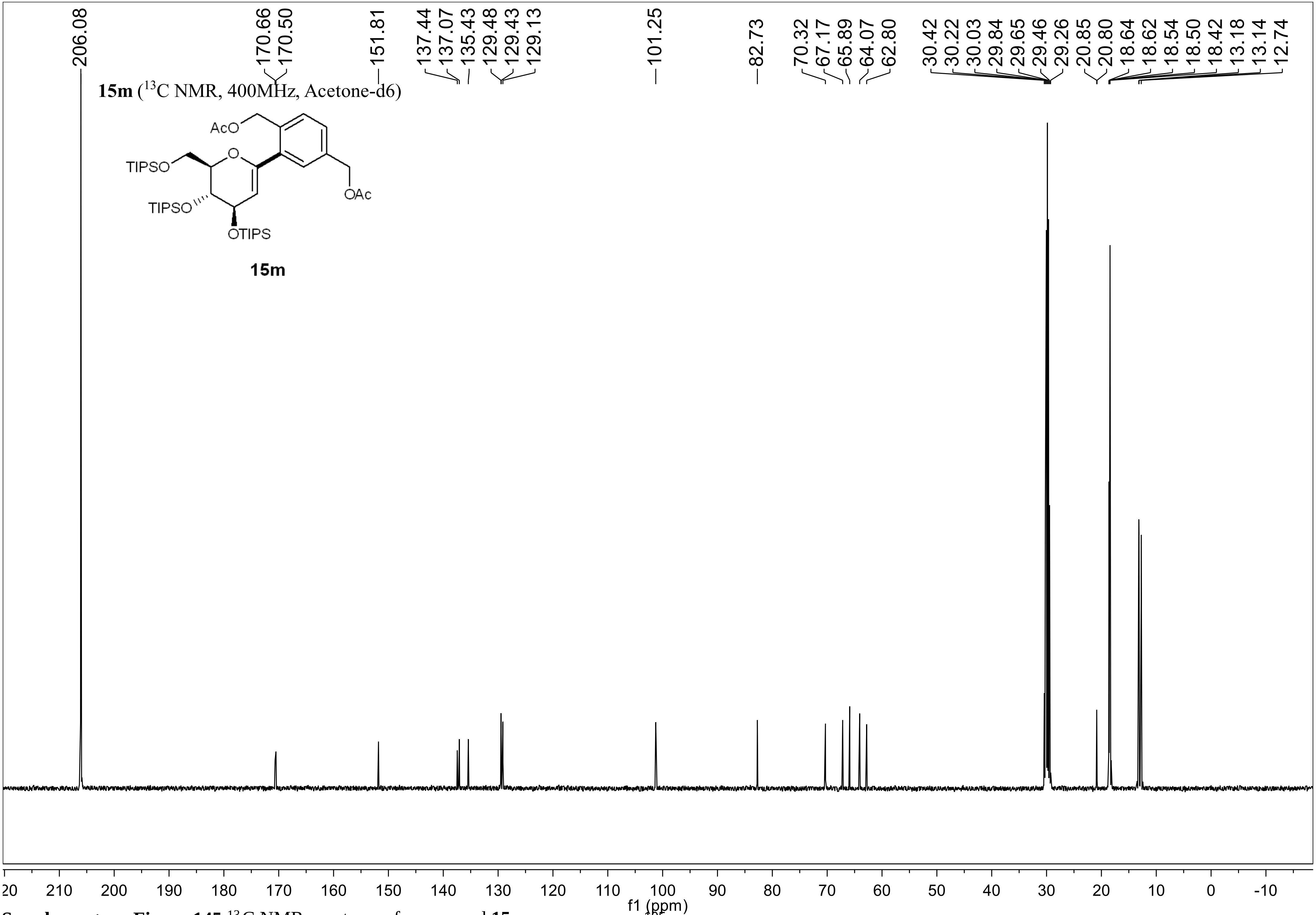

Supplementary Figure 145. ^{13}C NMR spectrum of compound **15m**

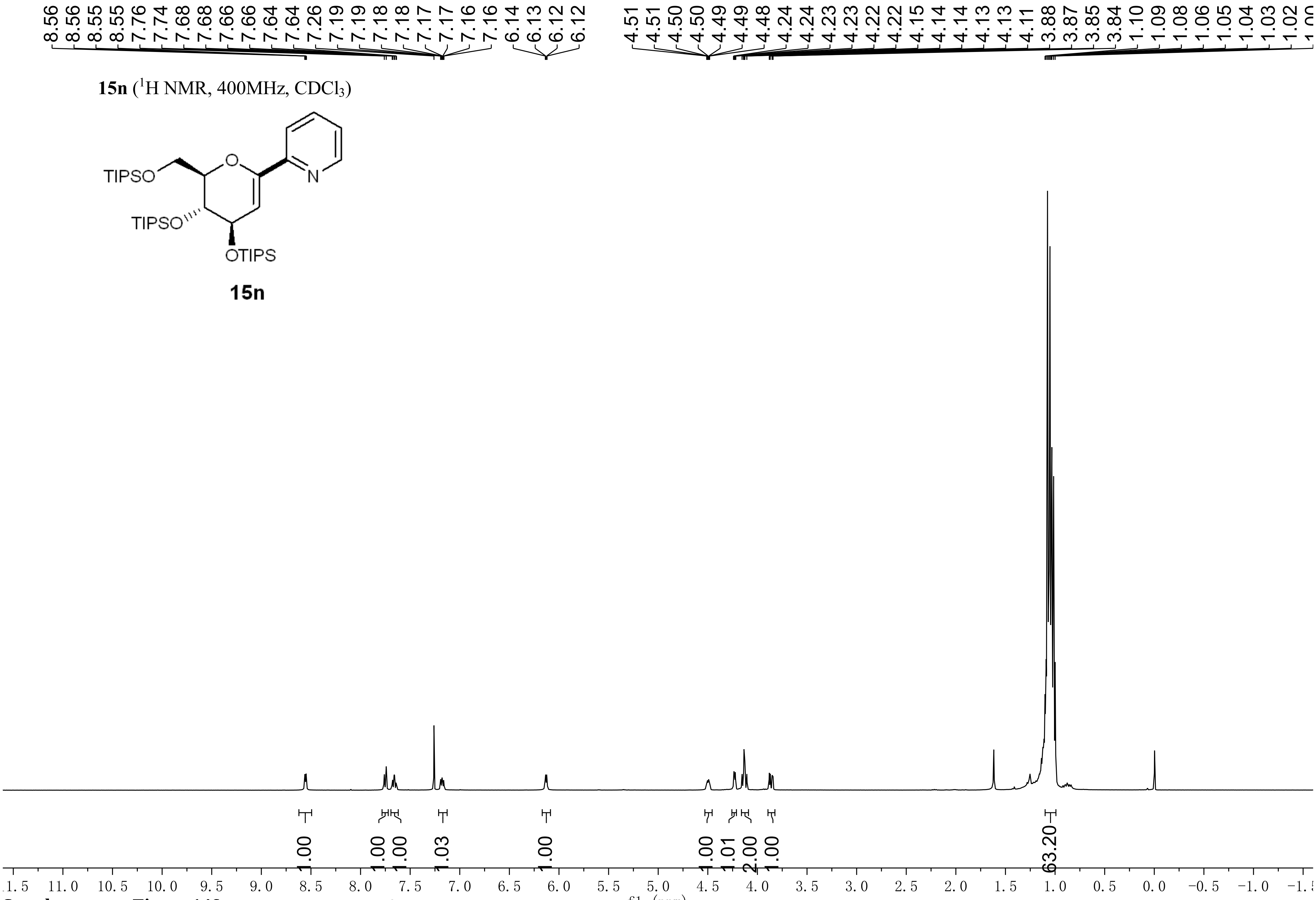

Supplementary Figure 146. ^{1}H NMR spectrum of compound **15n**

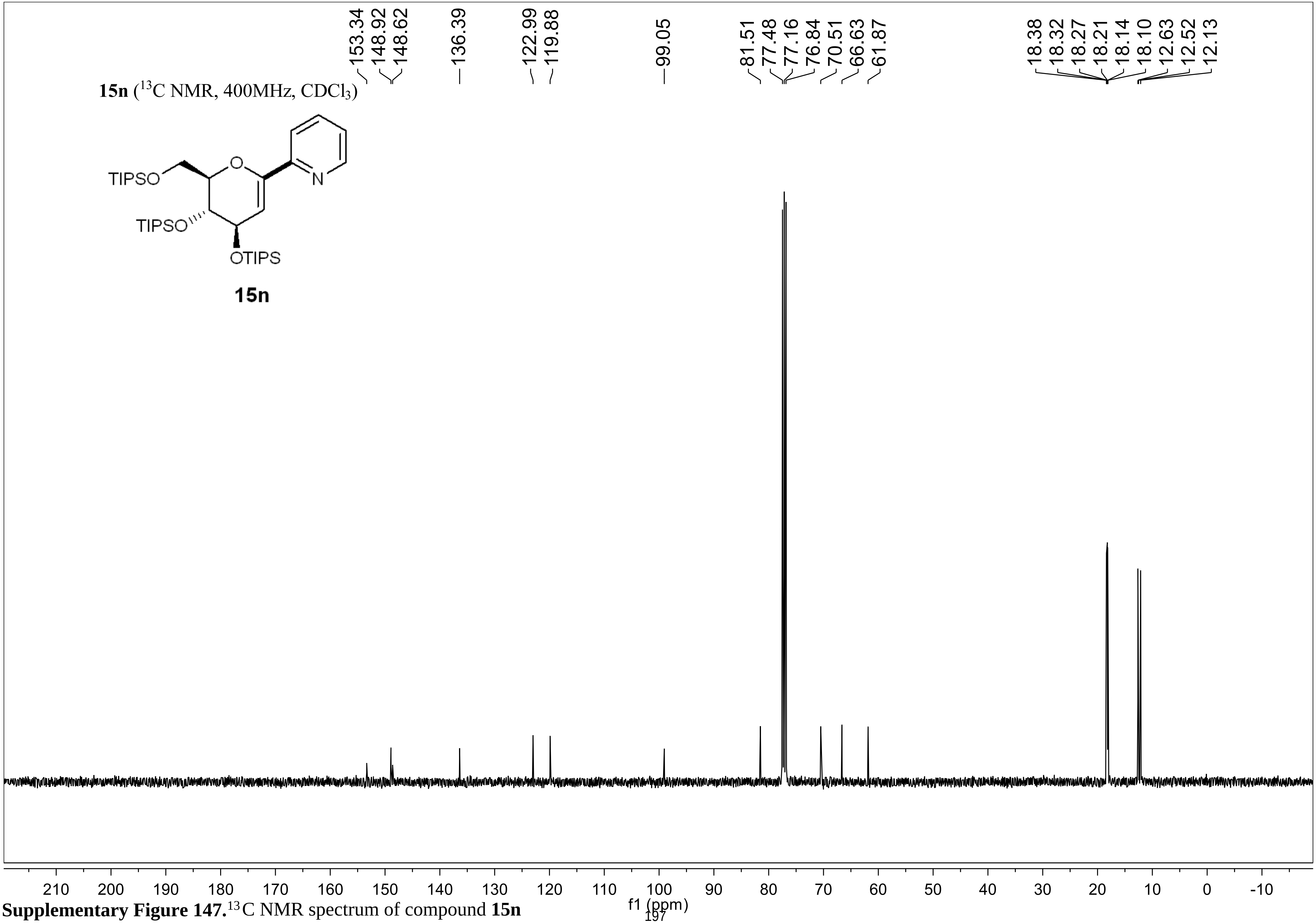

Supplementary Figure 147. ^{13}C NMR spectrum of compound **15n**

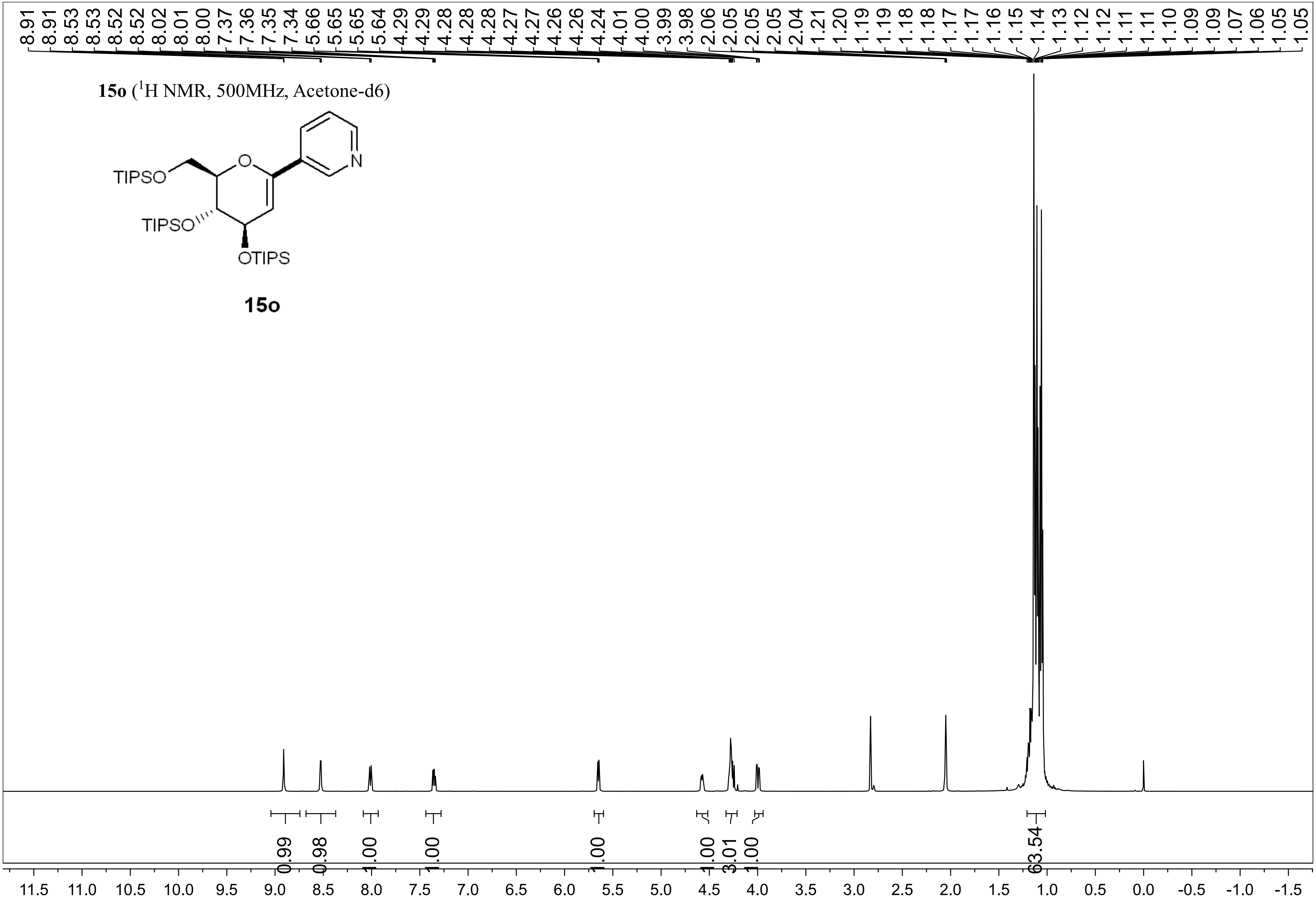

Supplementary Figure 148. ^{1}H NMR spectrum of compound **15o**

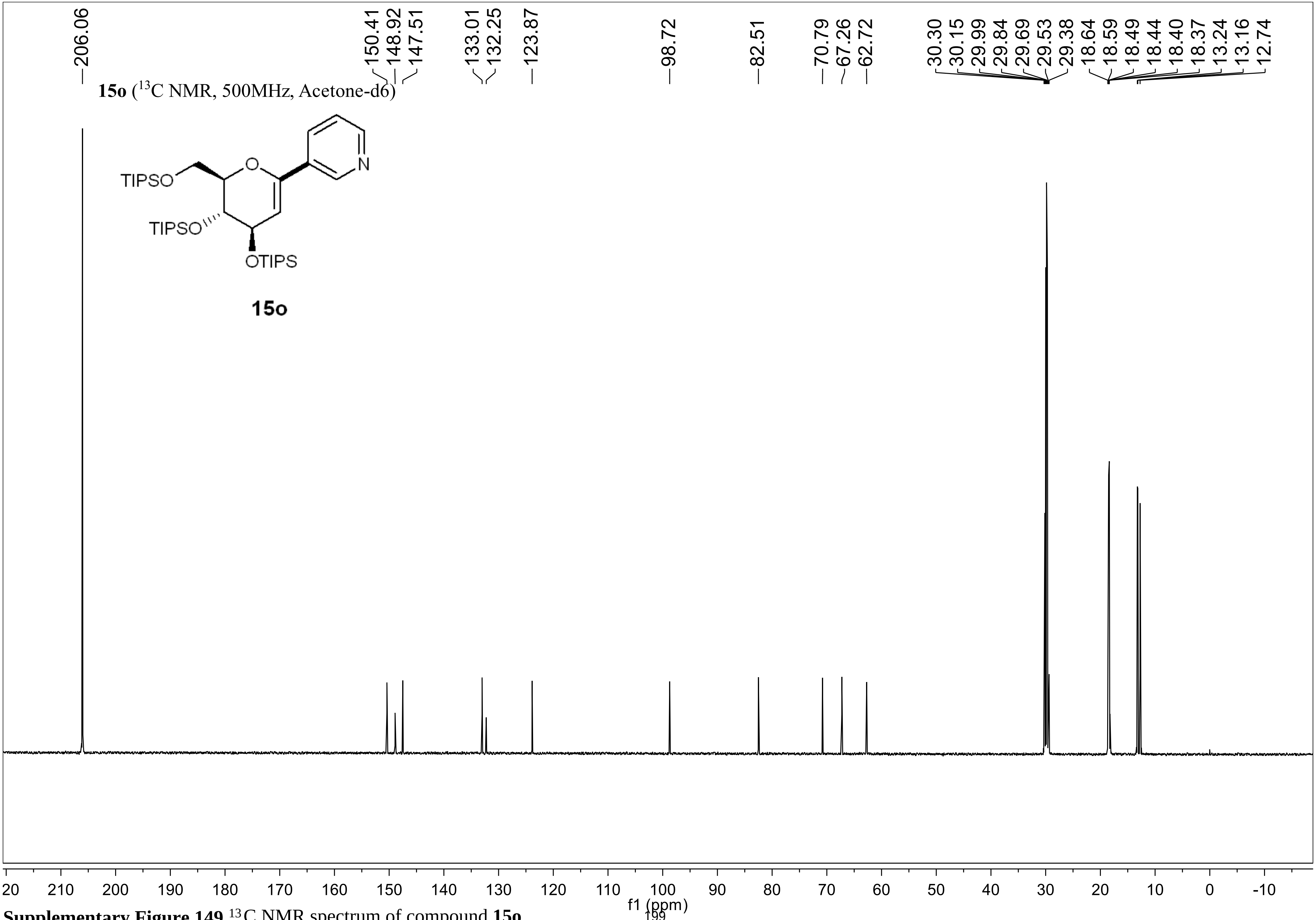

Supplementary Figure 149. ^{13}C NMR spectrum of compound **15o**

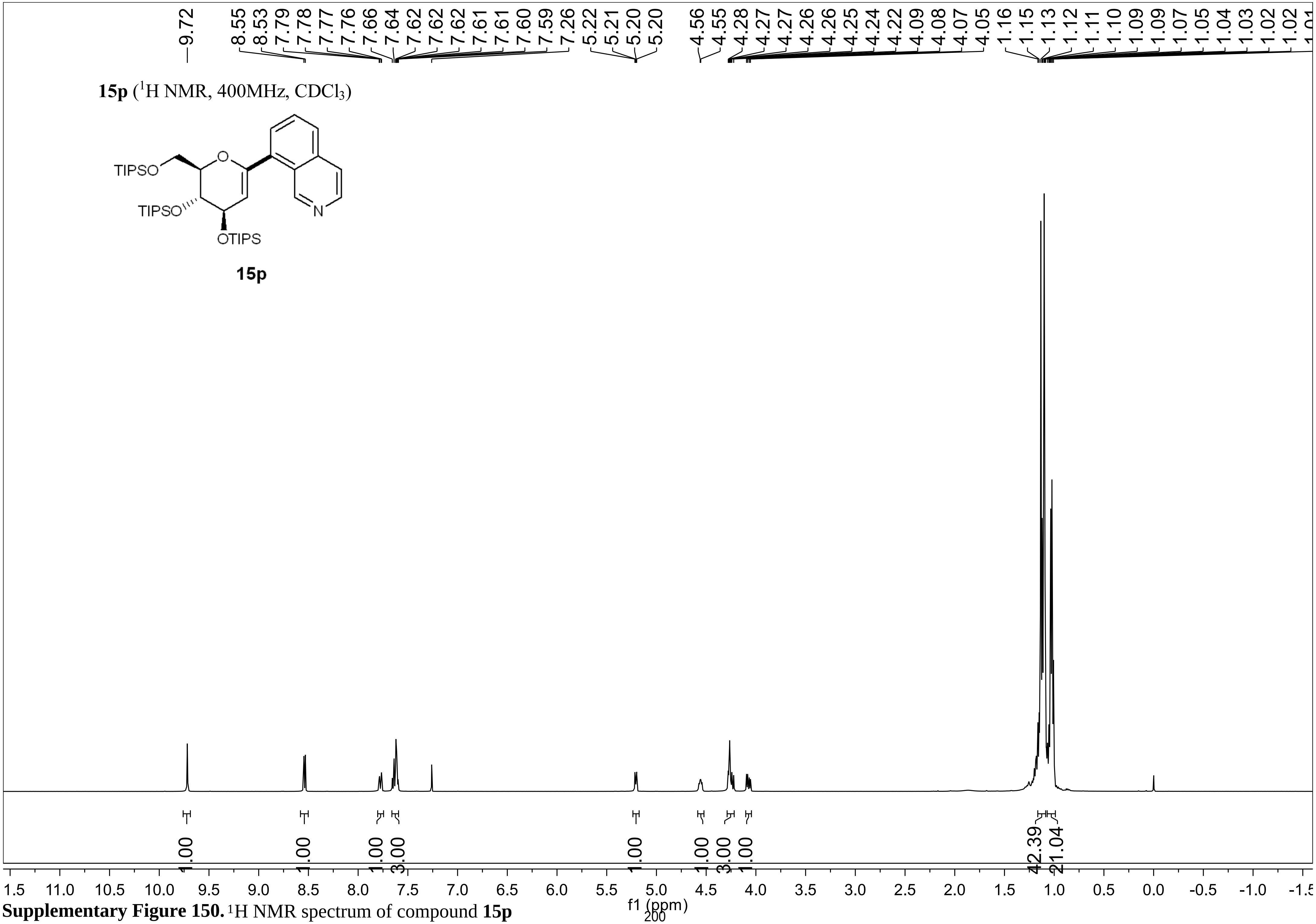

Supplementary Figure 150. ^{1}H NMR spectrum of compound **15p**

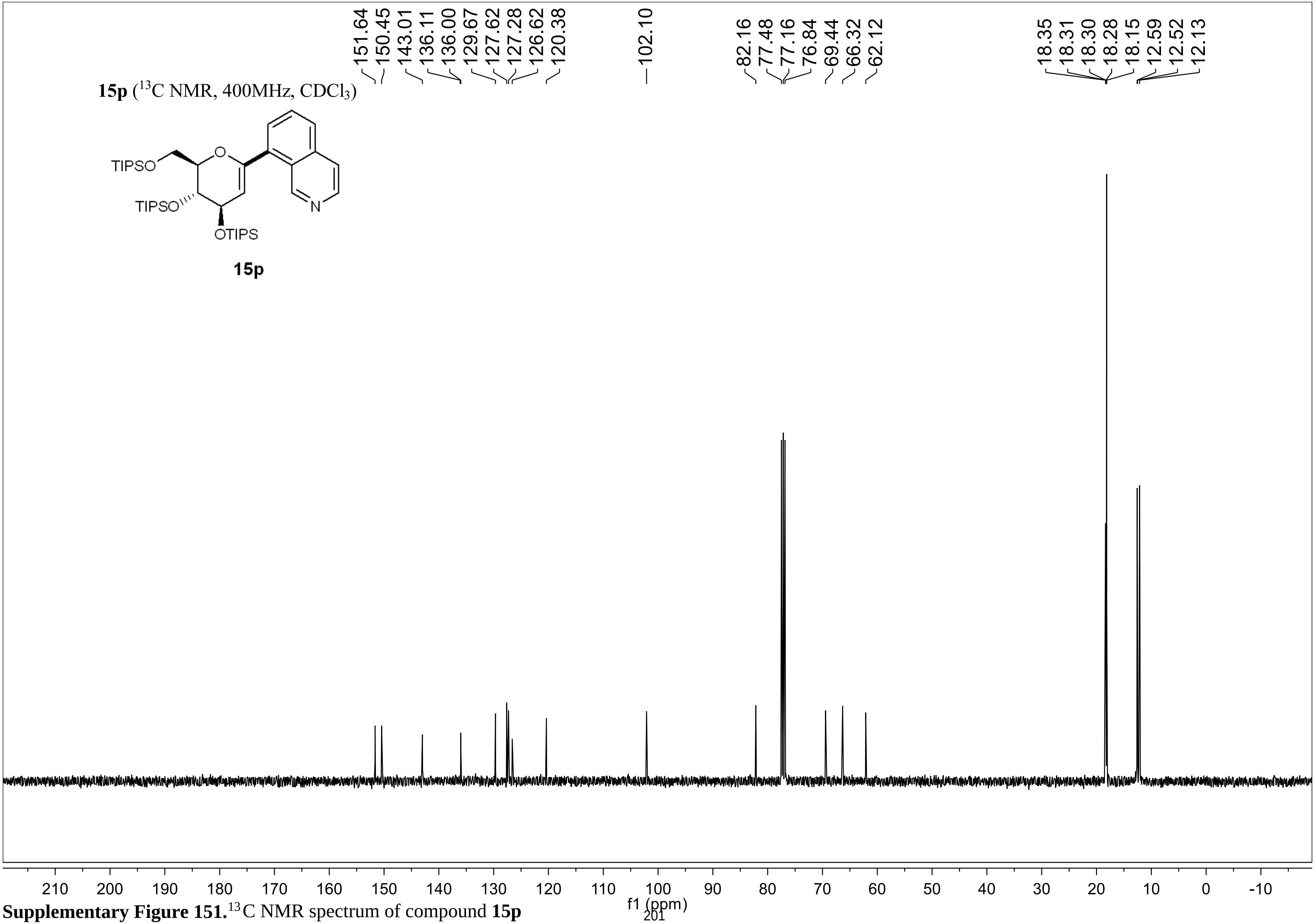

Supplementary Figure 151. ^{13}C NMR spectrum of compound **15p**

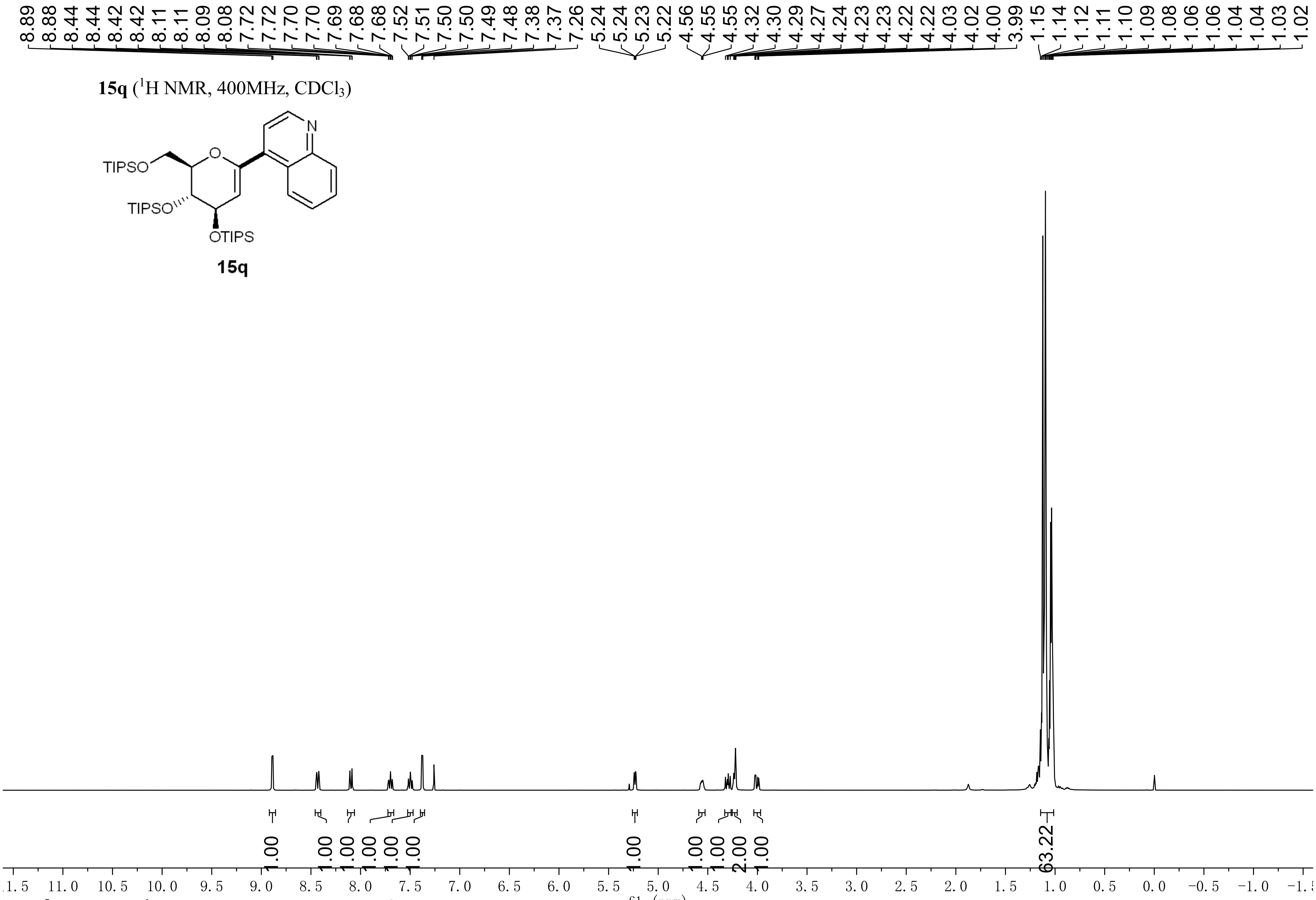

Supplementary Figure 152. ^{1}H NMR spectrum of compound **15q**

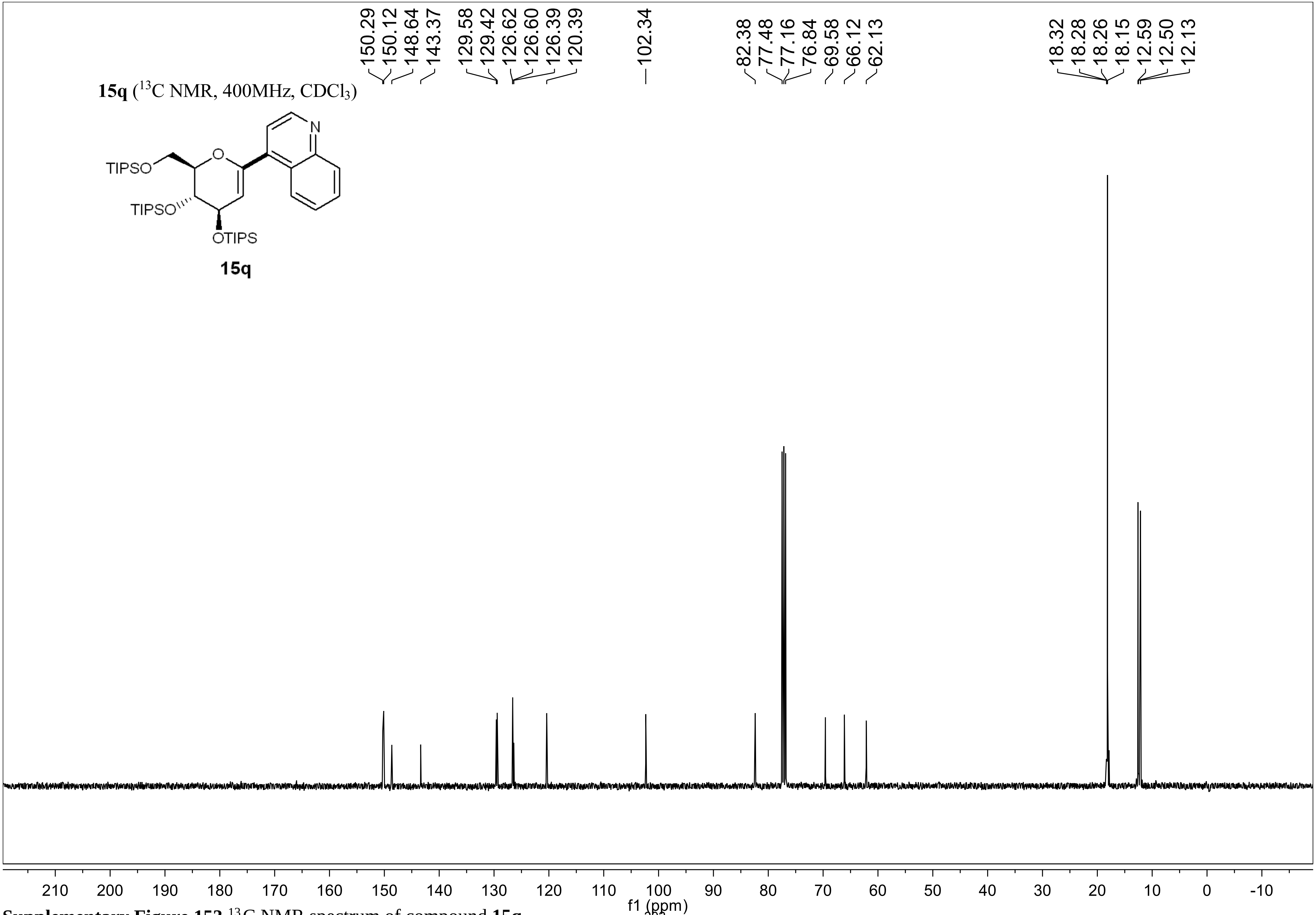

Supplementary Figure 153. ^{13}C NMR spectrum of compound **15q**

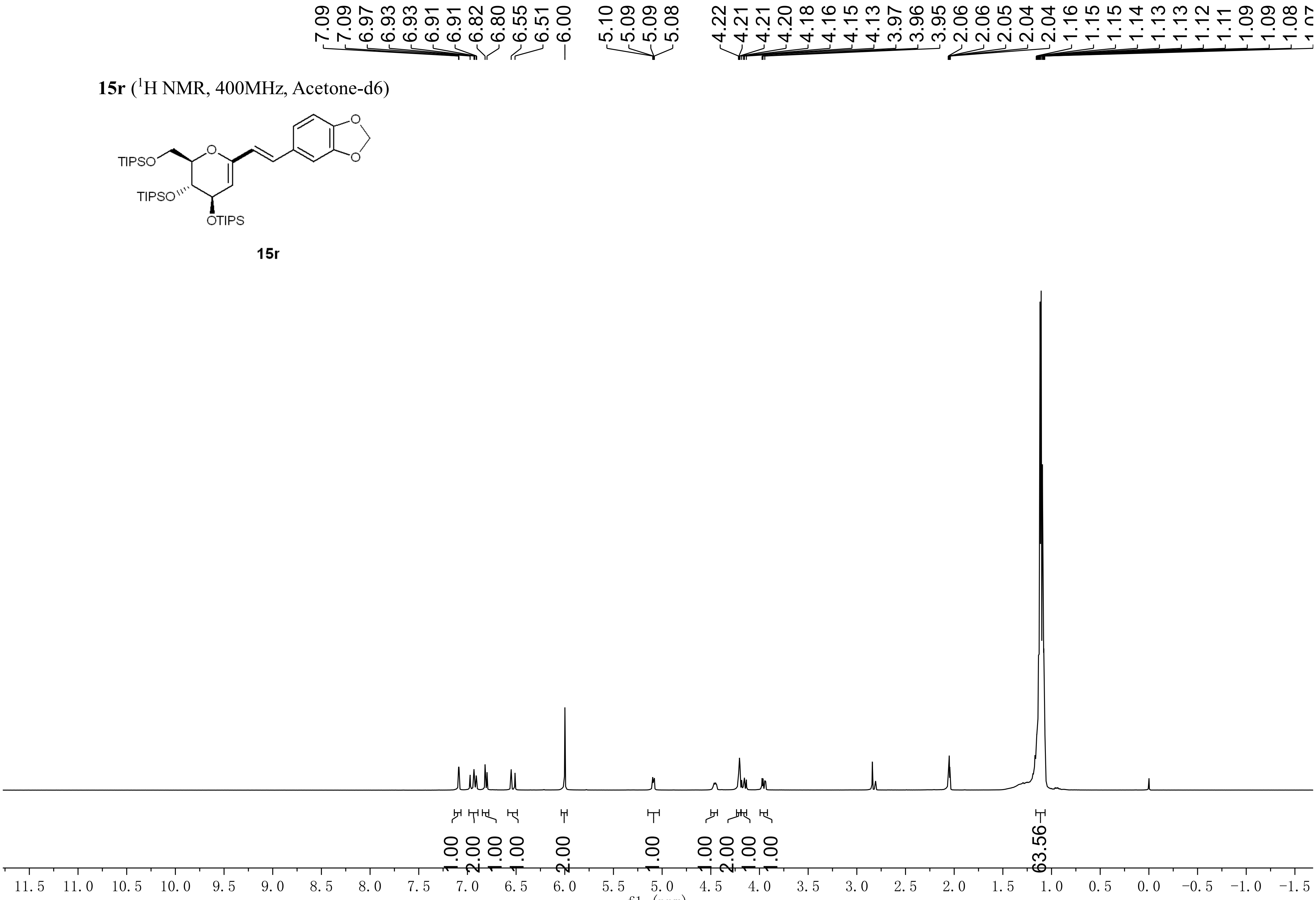

Supplementary Figure 154. ^{1}H NMR spectrum of compound **15r**

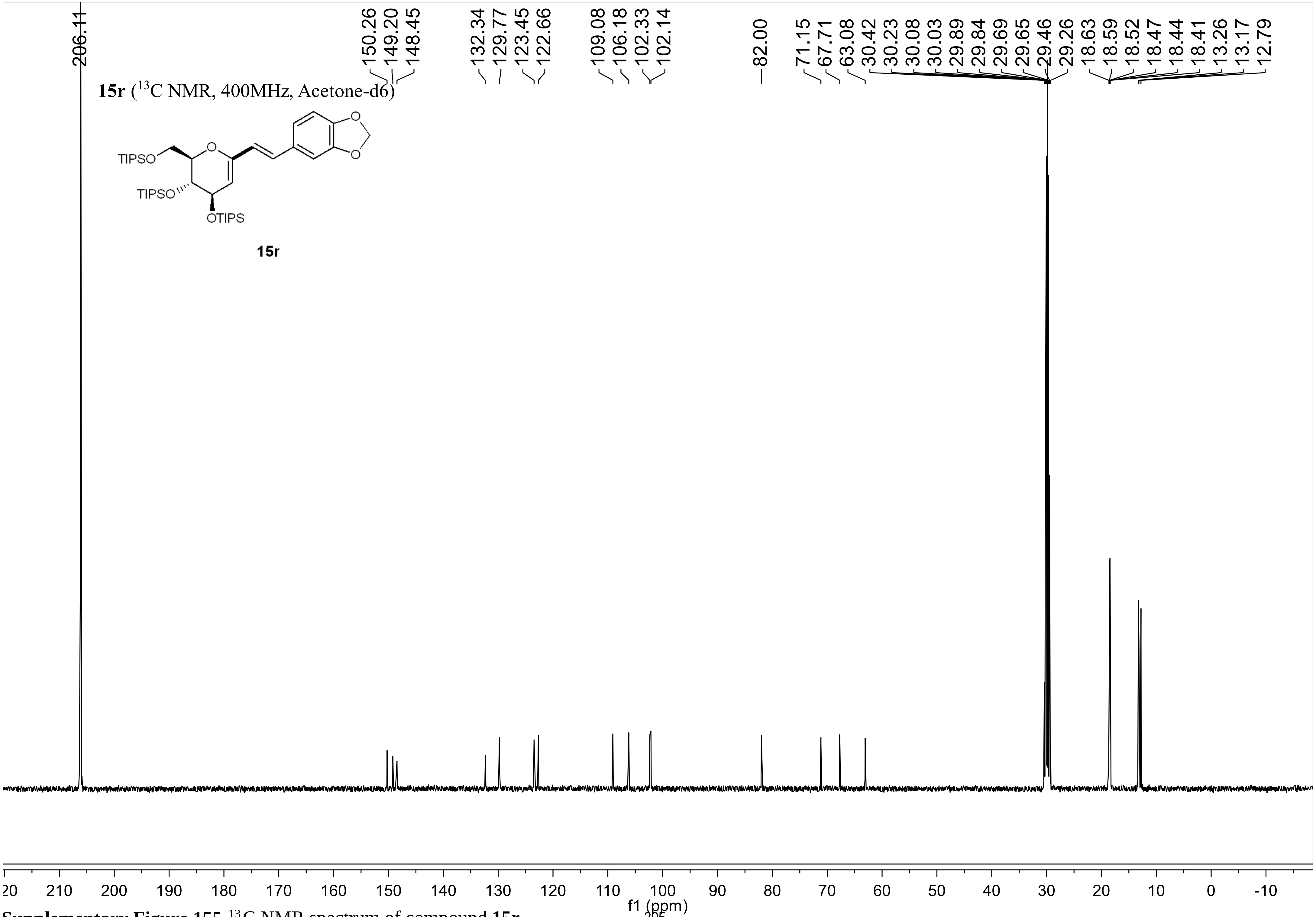

Supplementary Figure 155. ^{13}C NMR spectrum of compound **15r**

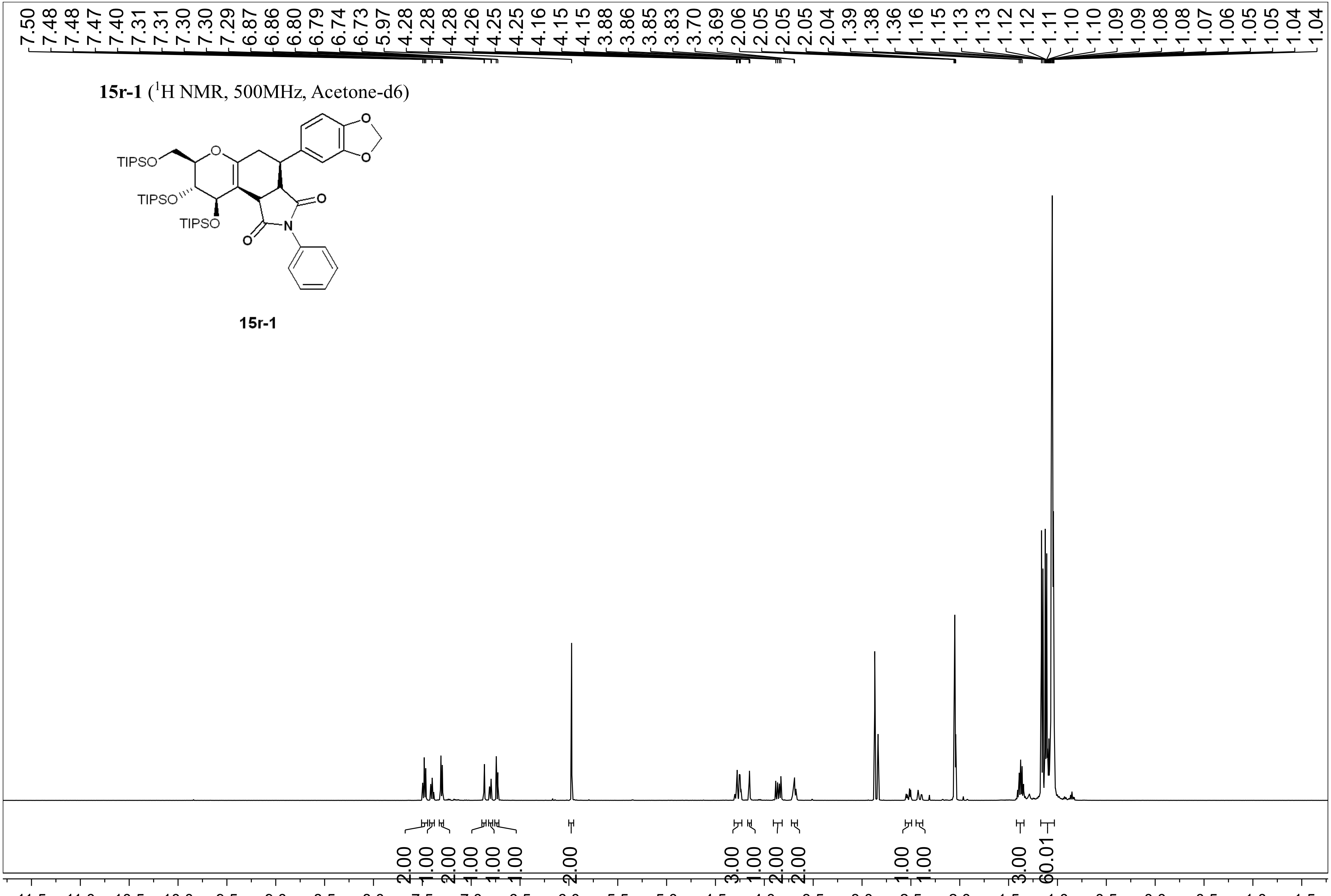

Supplementary Figure 156. ^{1}H NMR spectrum of compound **15r-1**

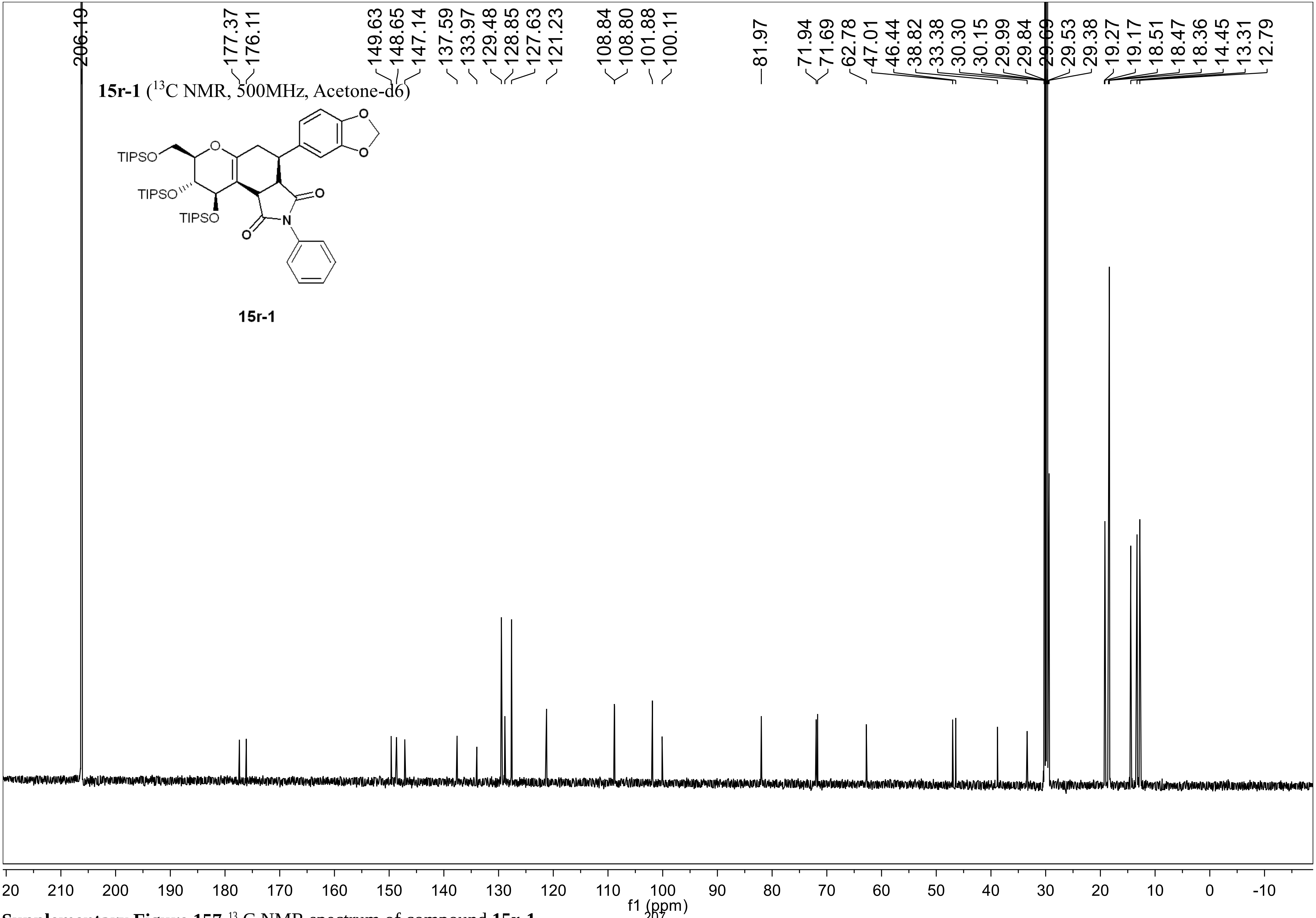

Supplementary Figure 157. ^{13} C NMR spectrum of compound **15r-1**

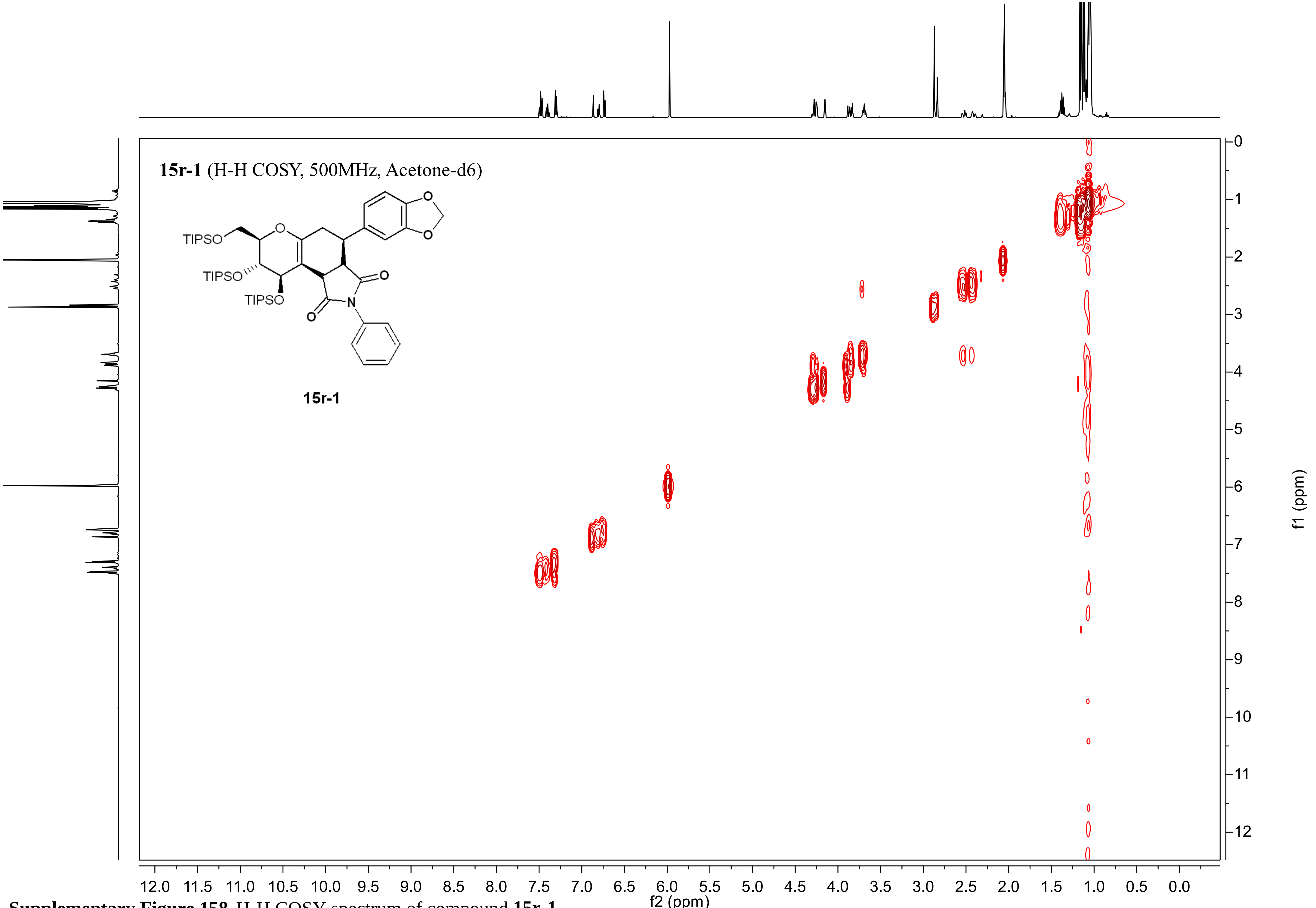

Supplementary Figure 158. H-H COSY spectrum of compound **15r-1**

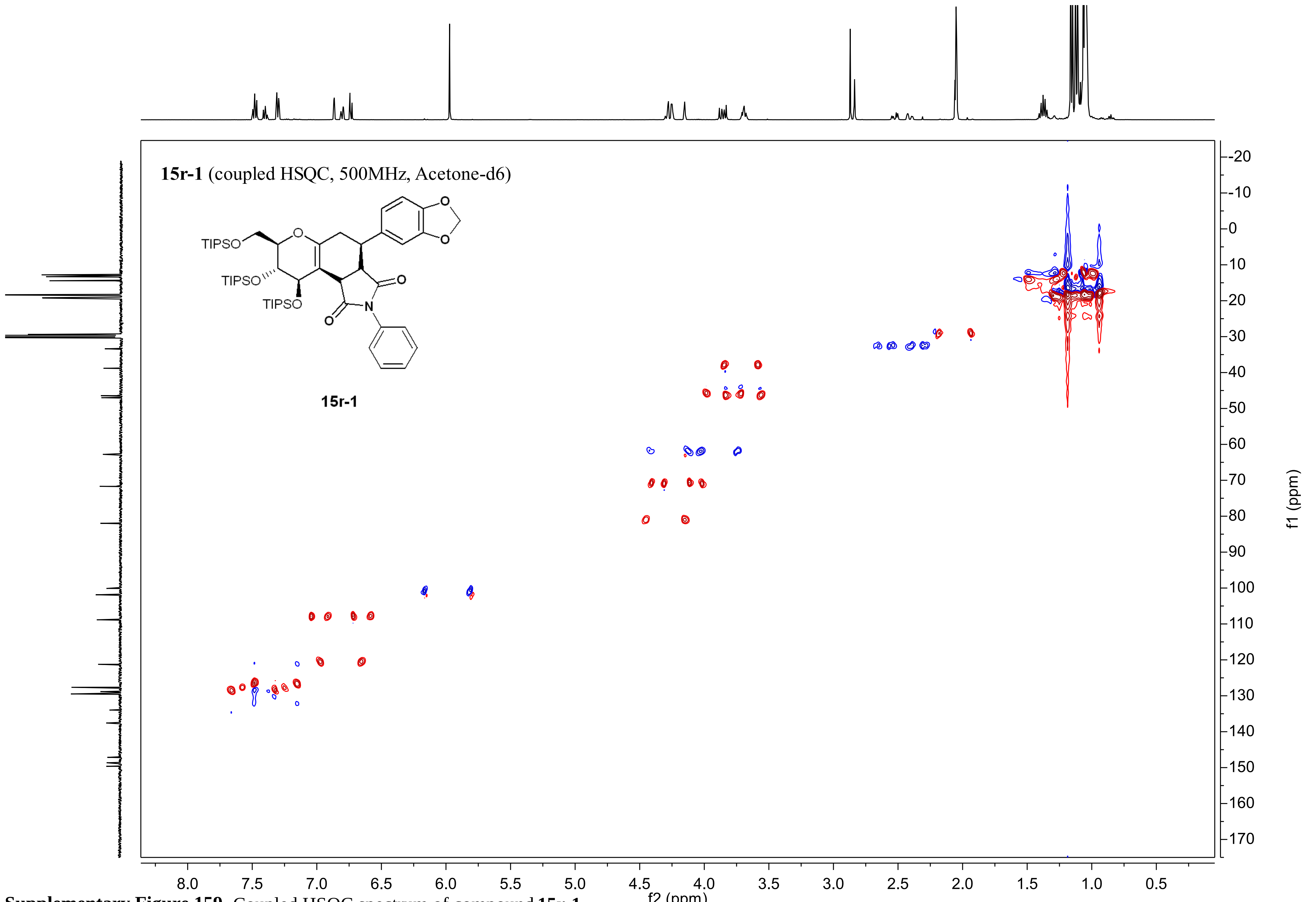

Supplementary Figure 159. Coupled HSQC spectrum of compound **15r-1**

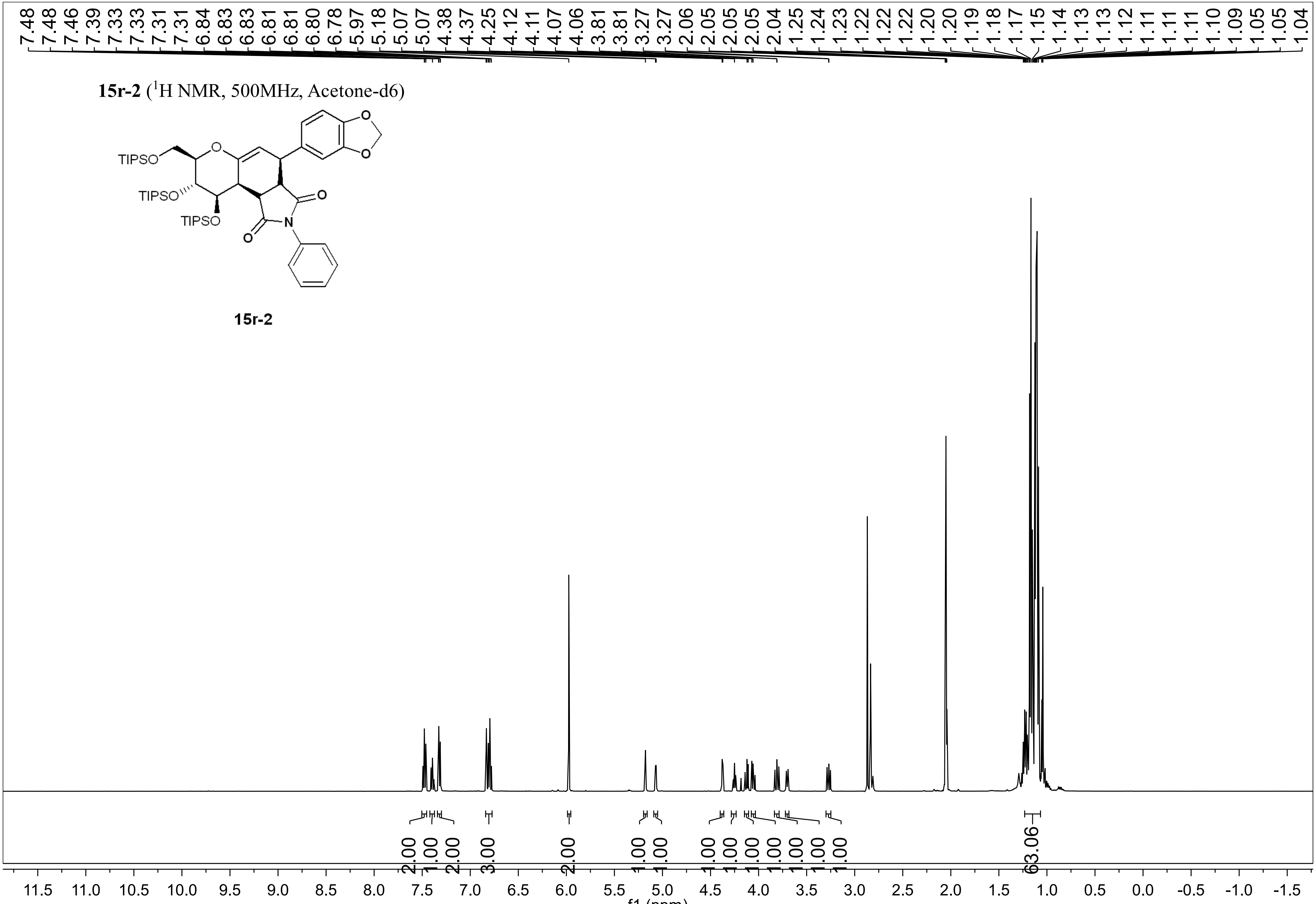

Supplementary Figure 160. ^{1}H NMR spectrum of compound **15r-2**

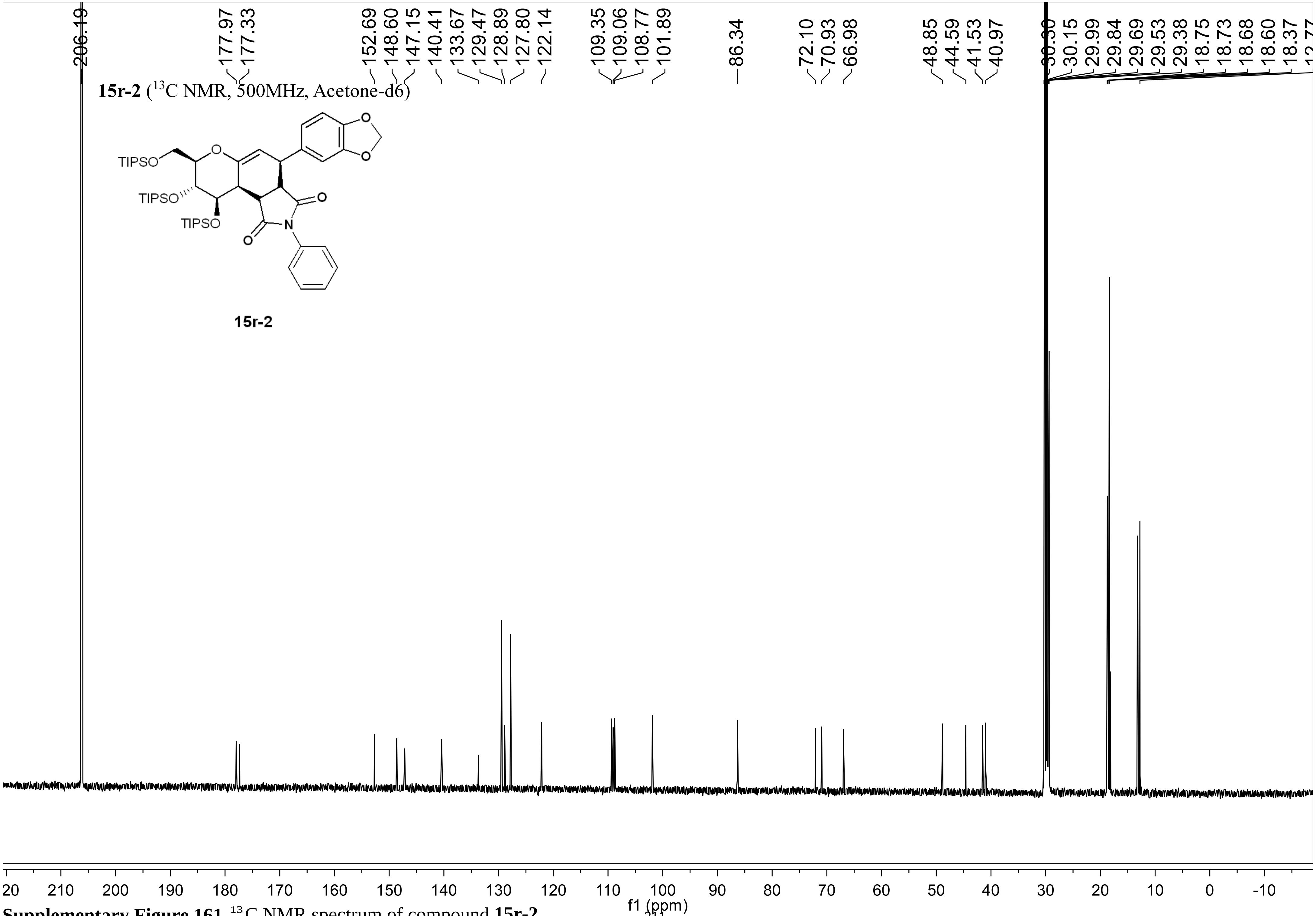

Supplementary Figure 161. ^{13}C NMR spectrum of compound **15r-2**

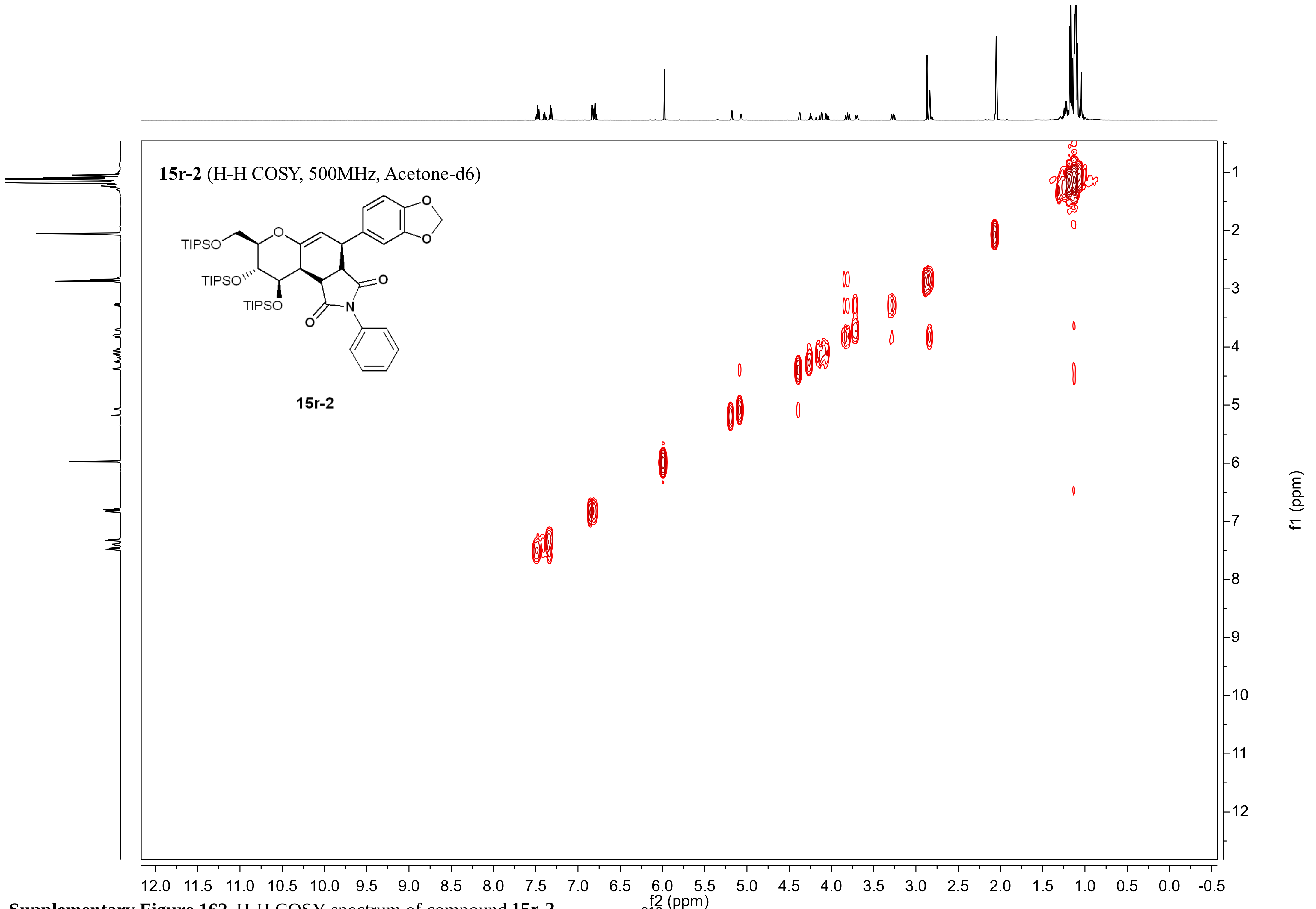

Supplementary Figure 162. H-H COSY spectrum of compound **15r-2**

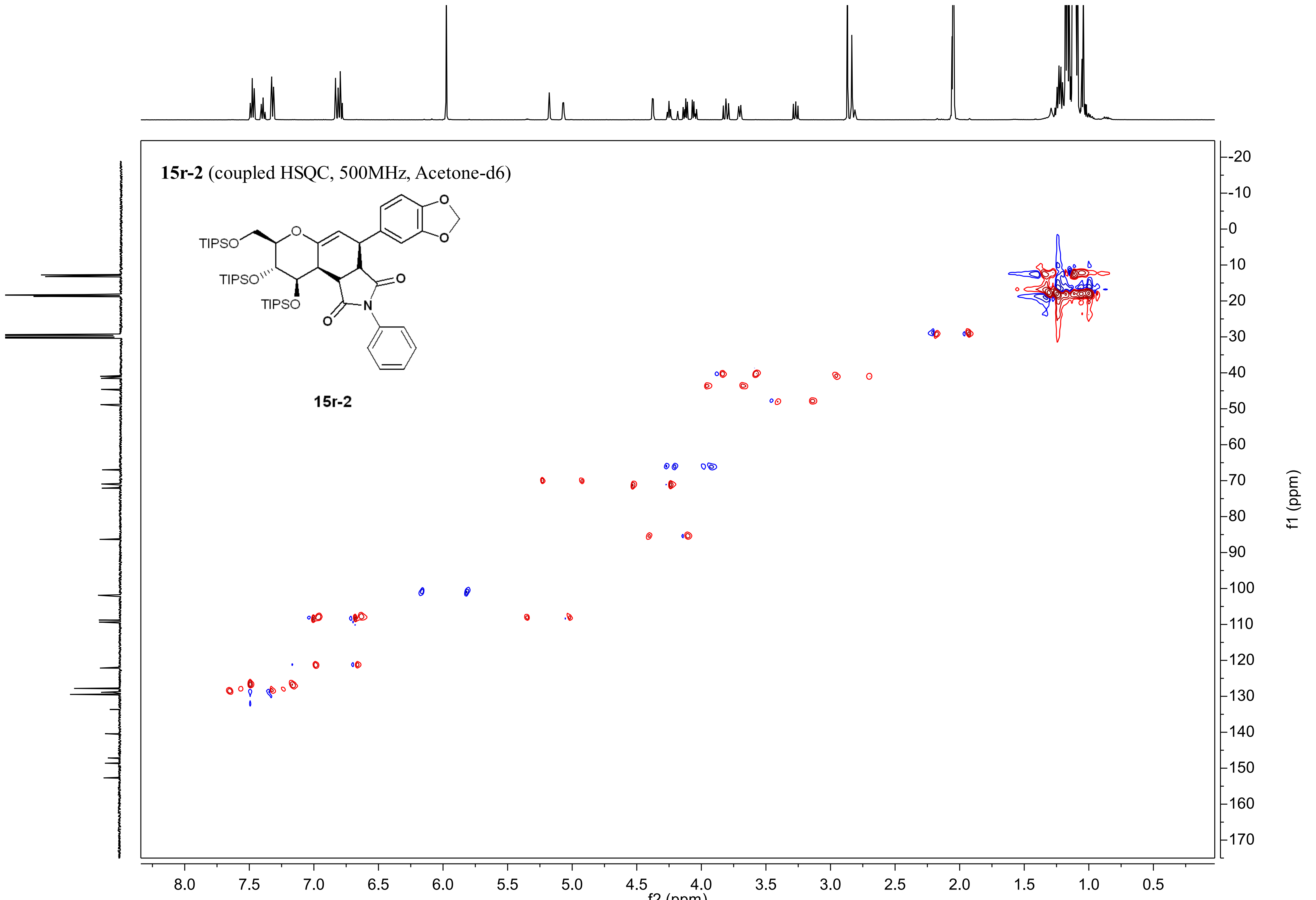

Supplementary Figure 163. Coupled HSQC spectrum of compound **15r-2**

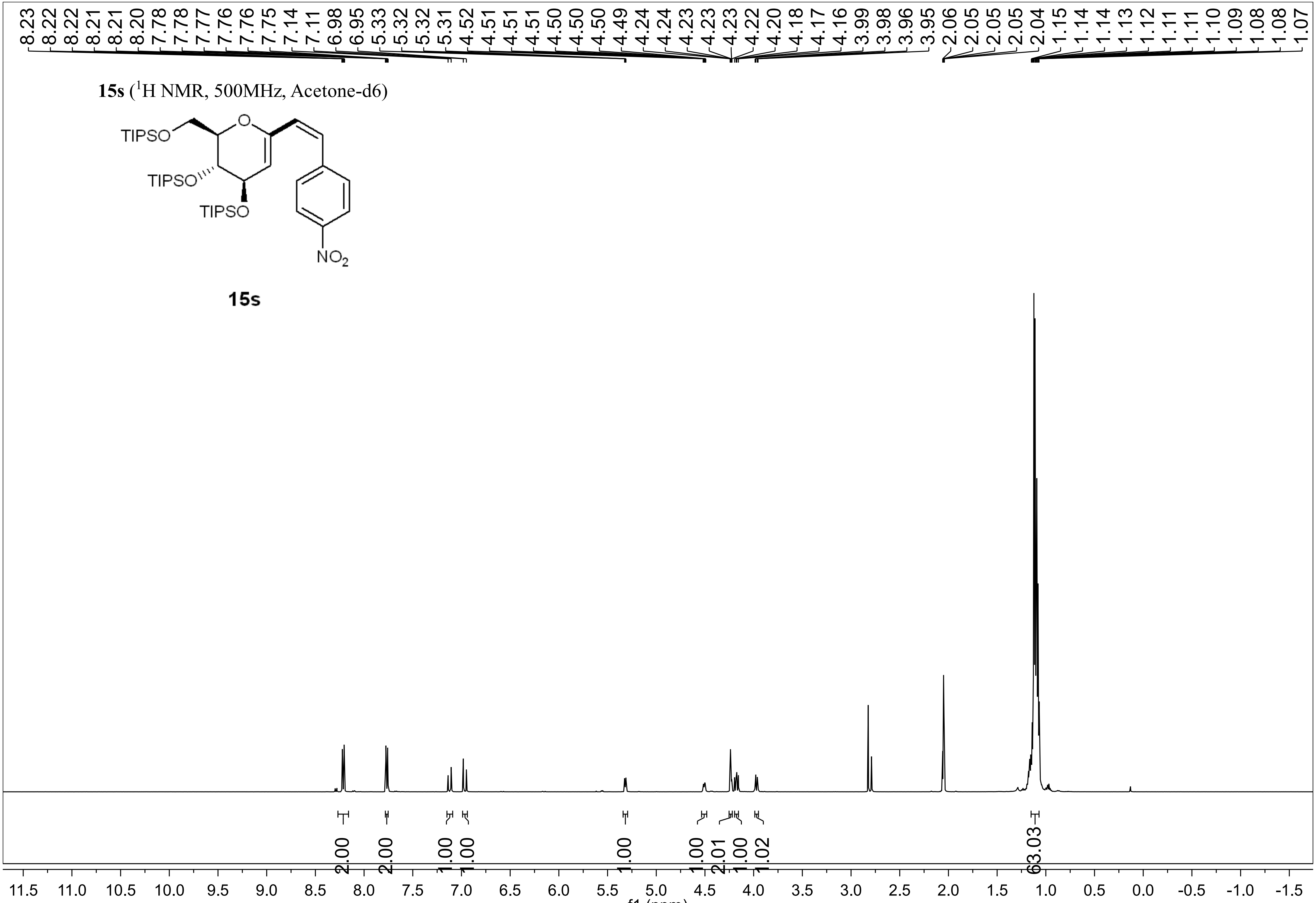

Supplementary Figure 164. ^{1}H NMR spectrum of compound **15s**

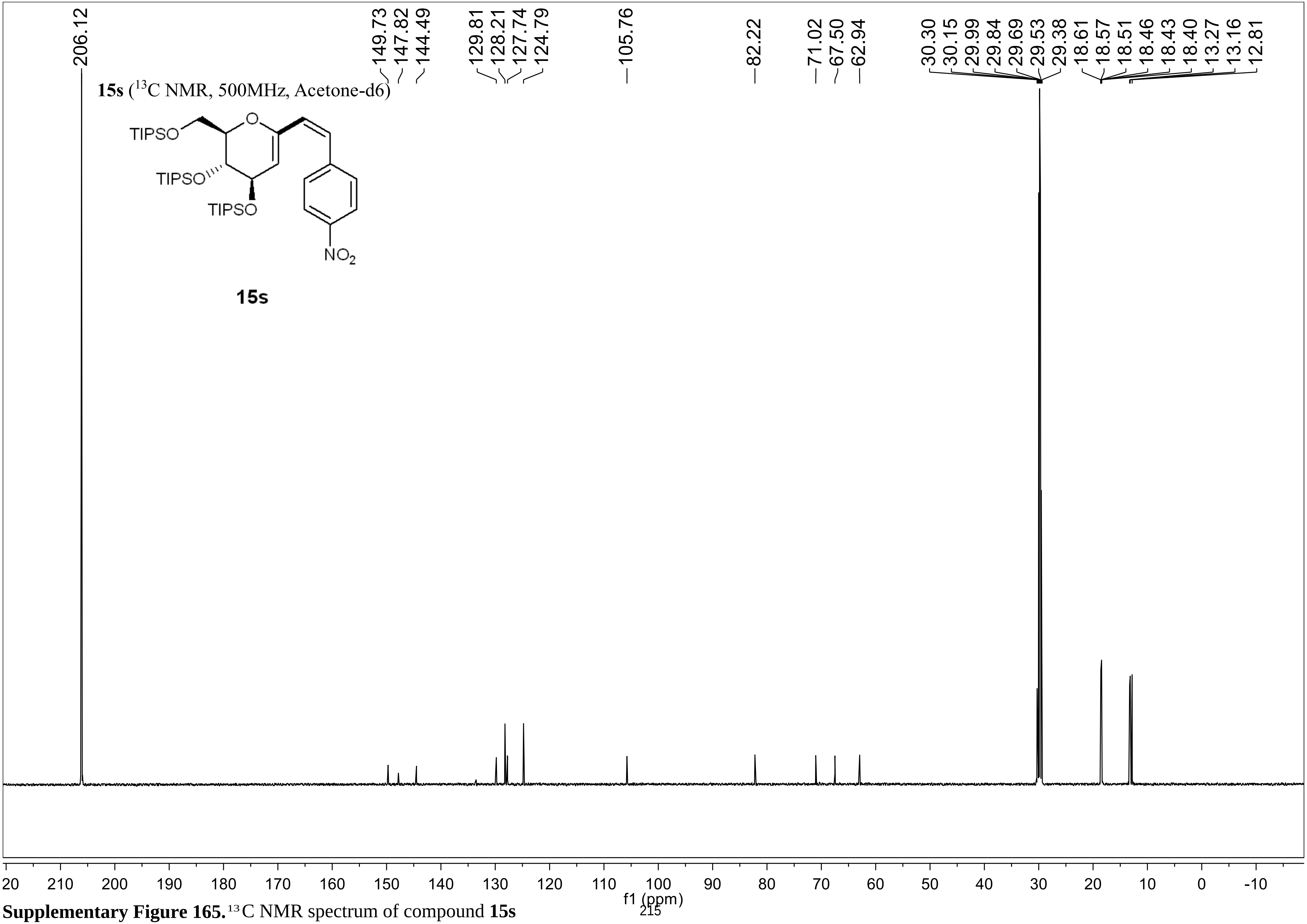

Supplementary Figure 165. ^{13}C NMR spectrum of compound **15s**

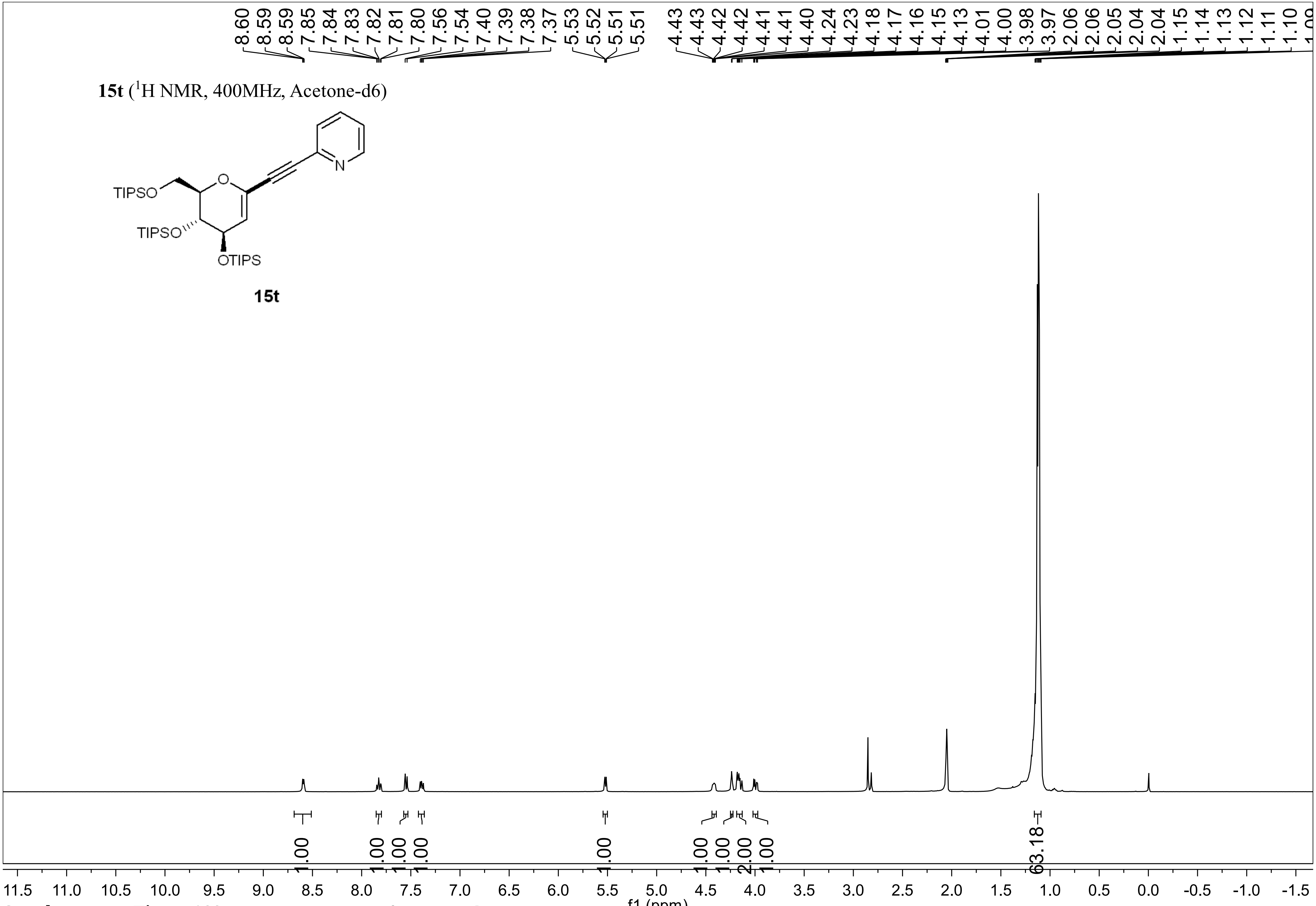

Supplementary Figure 166. ^{1}H NMR spectrum of compound **15t**

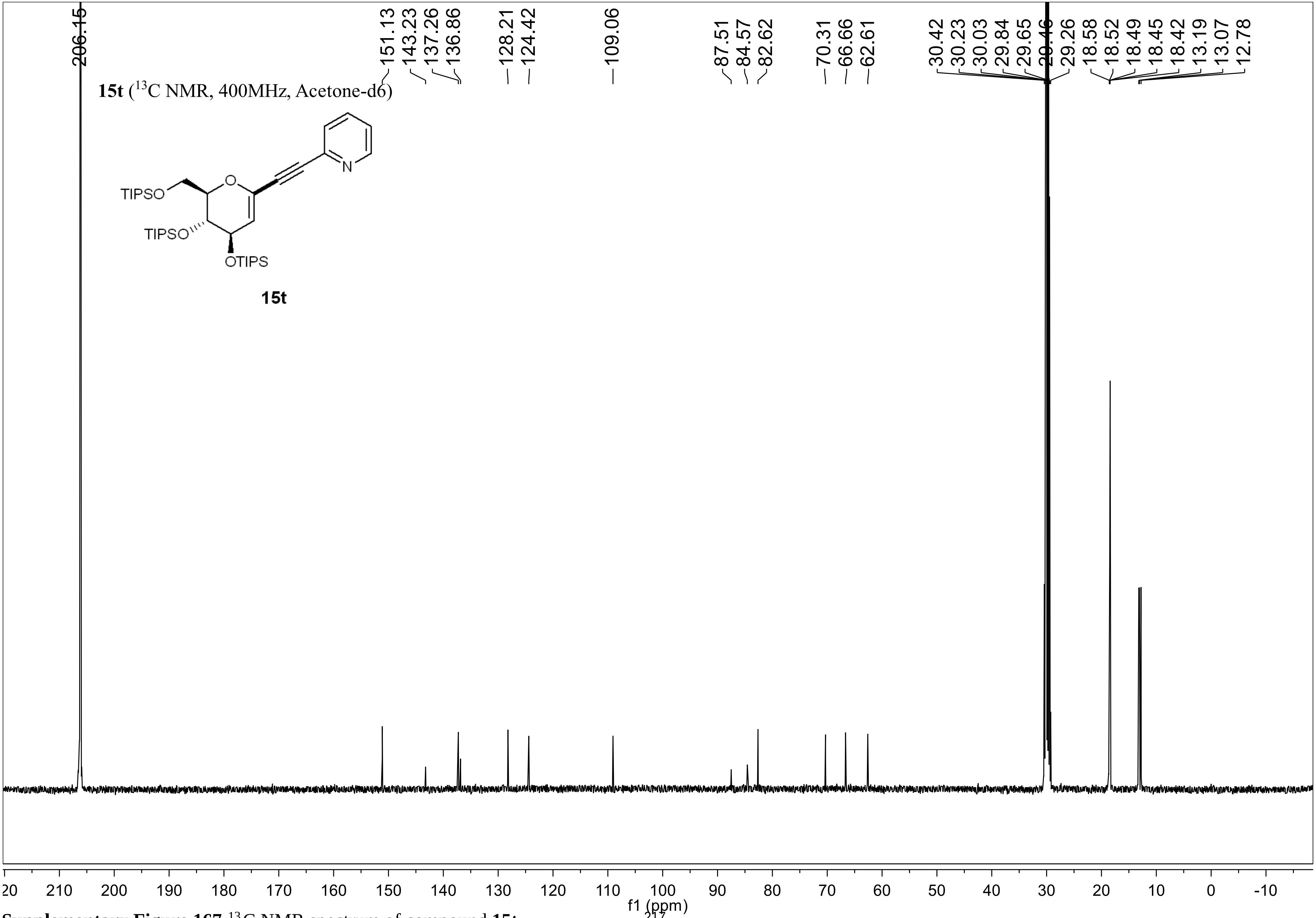

Supplementary Figure 167. ^{13}C NMR spectrum of compound **15t**

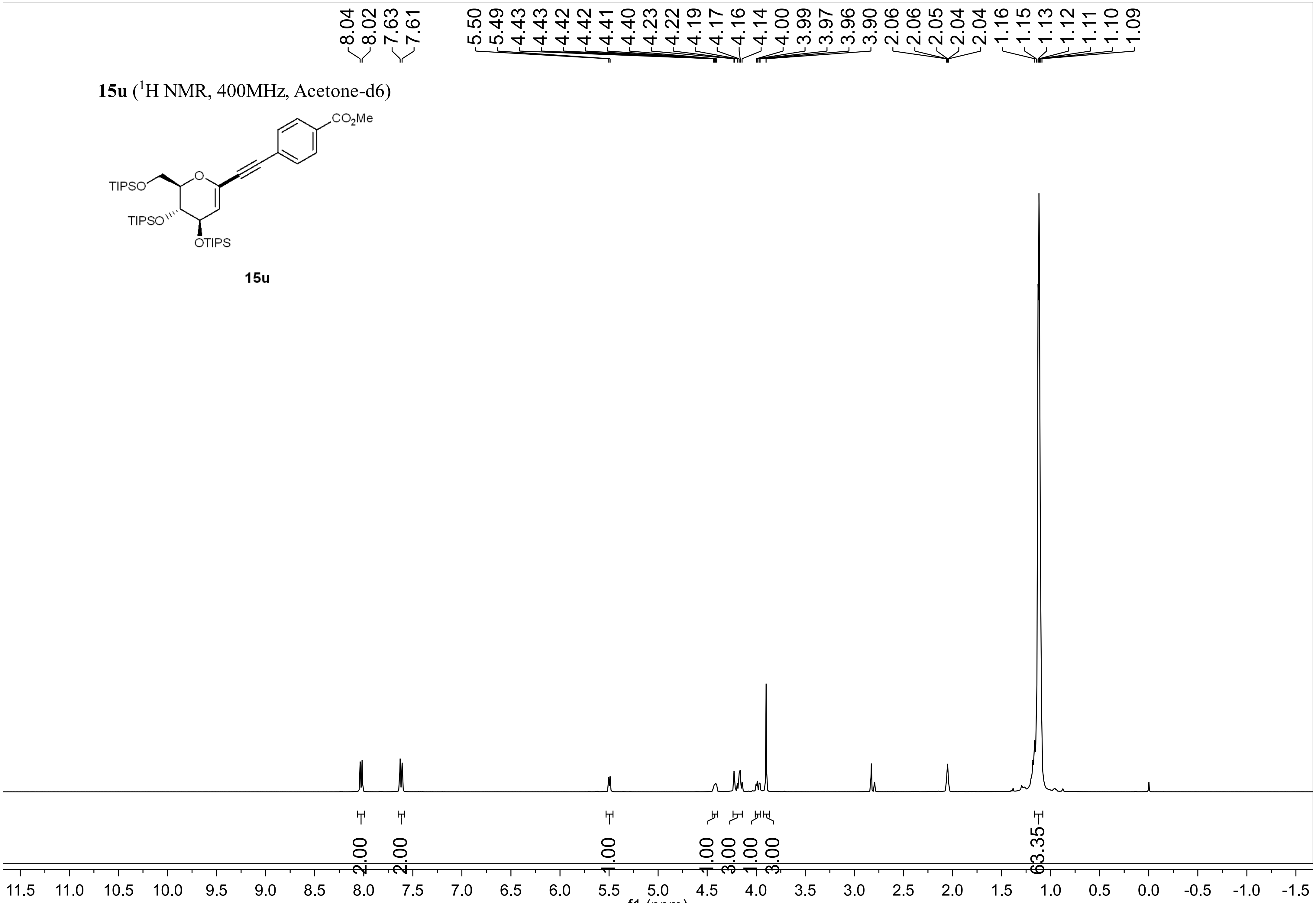

Supplementary Figure 168. ^{1}H NMR spectrum of compound **15u**

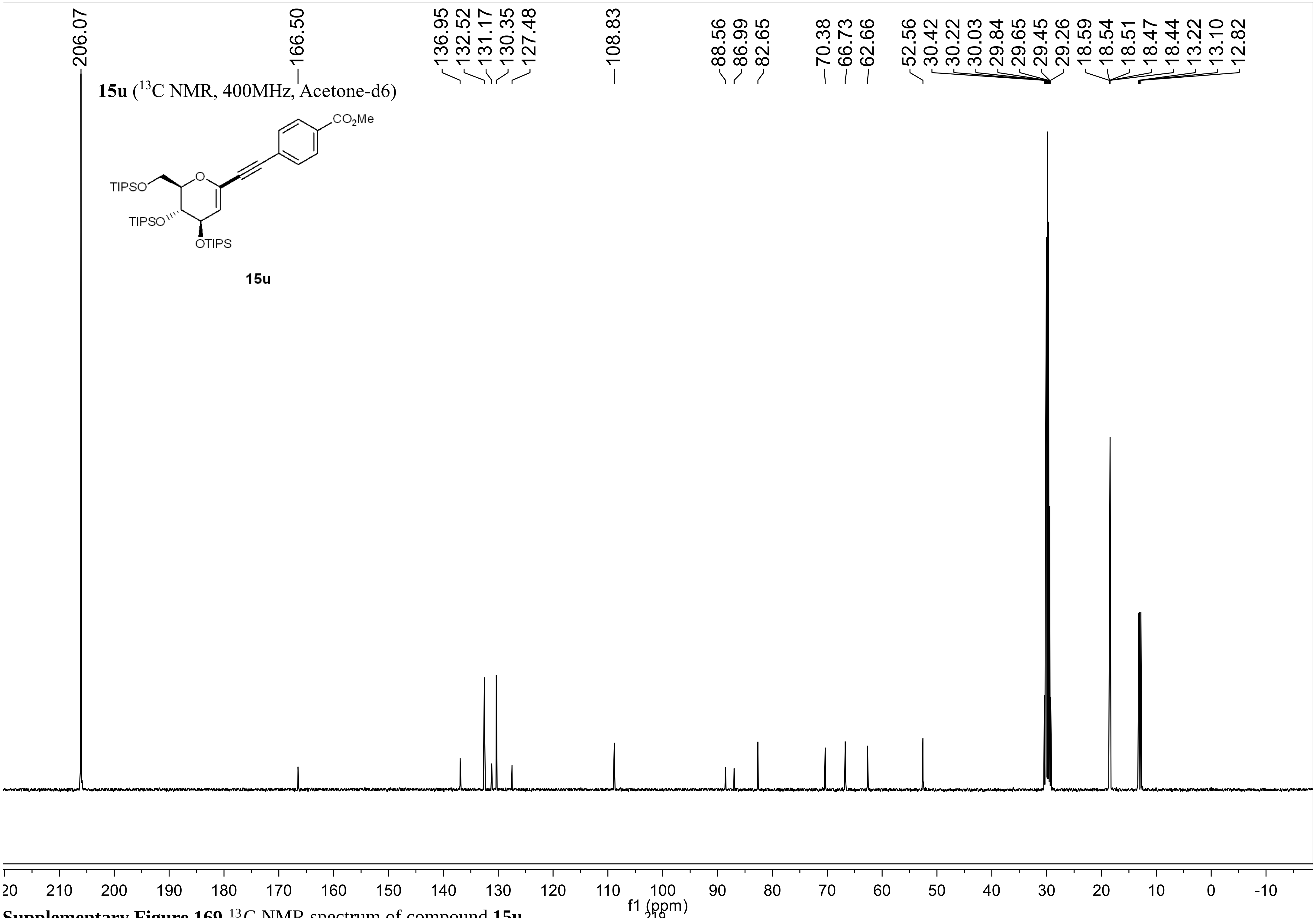

Supplementary Figure 169. ^{13}C NMR spectrum of compound **15u**

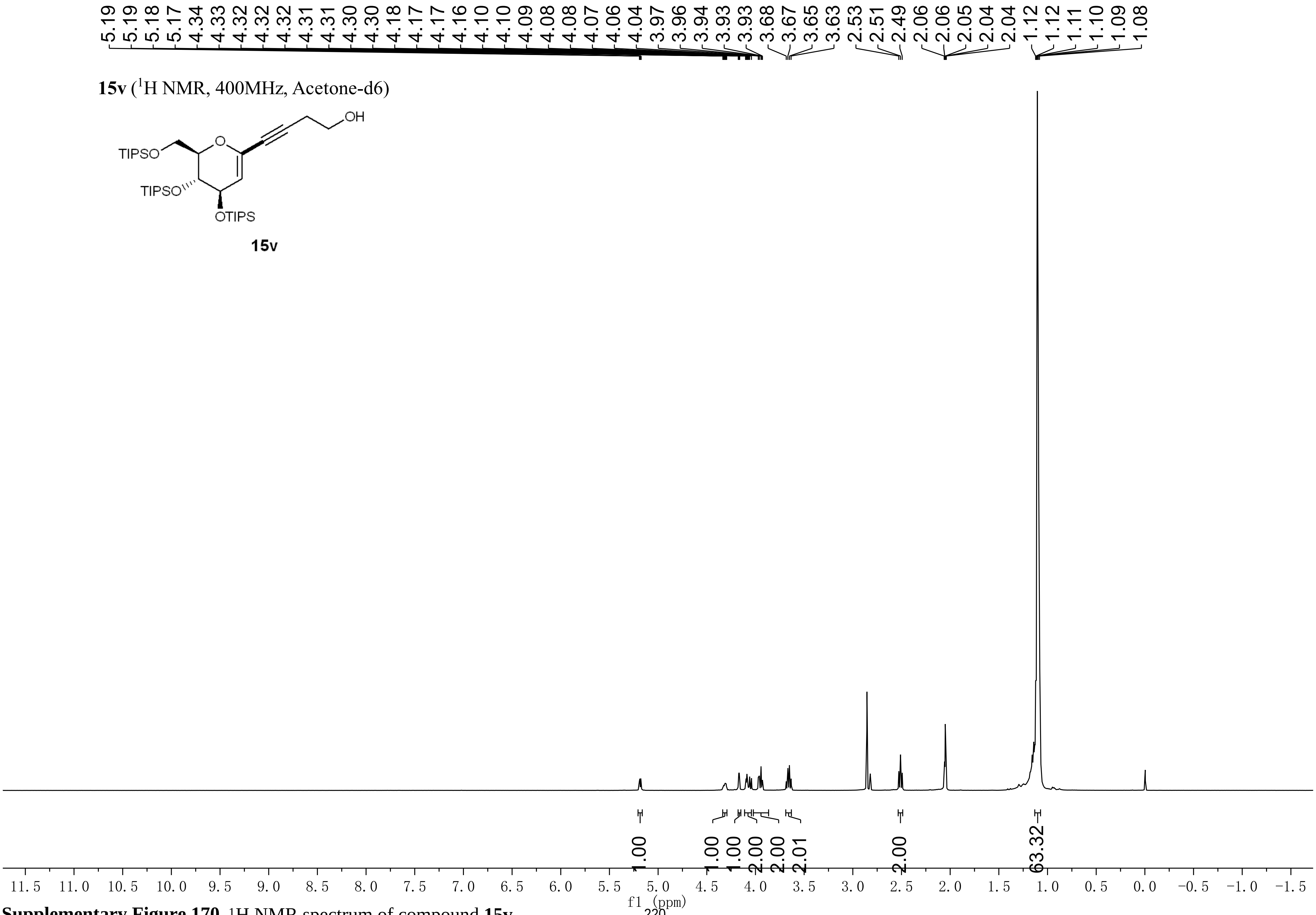

Supplementary Figure 170. ^{1}H NMR spectrum of compound **15v**

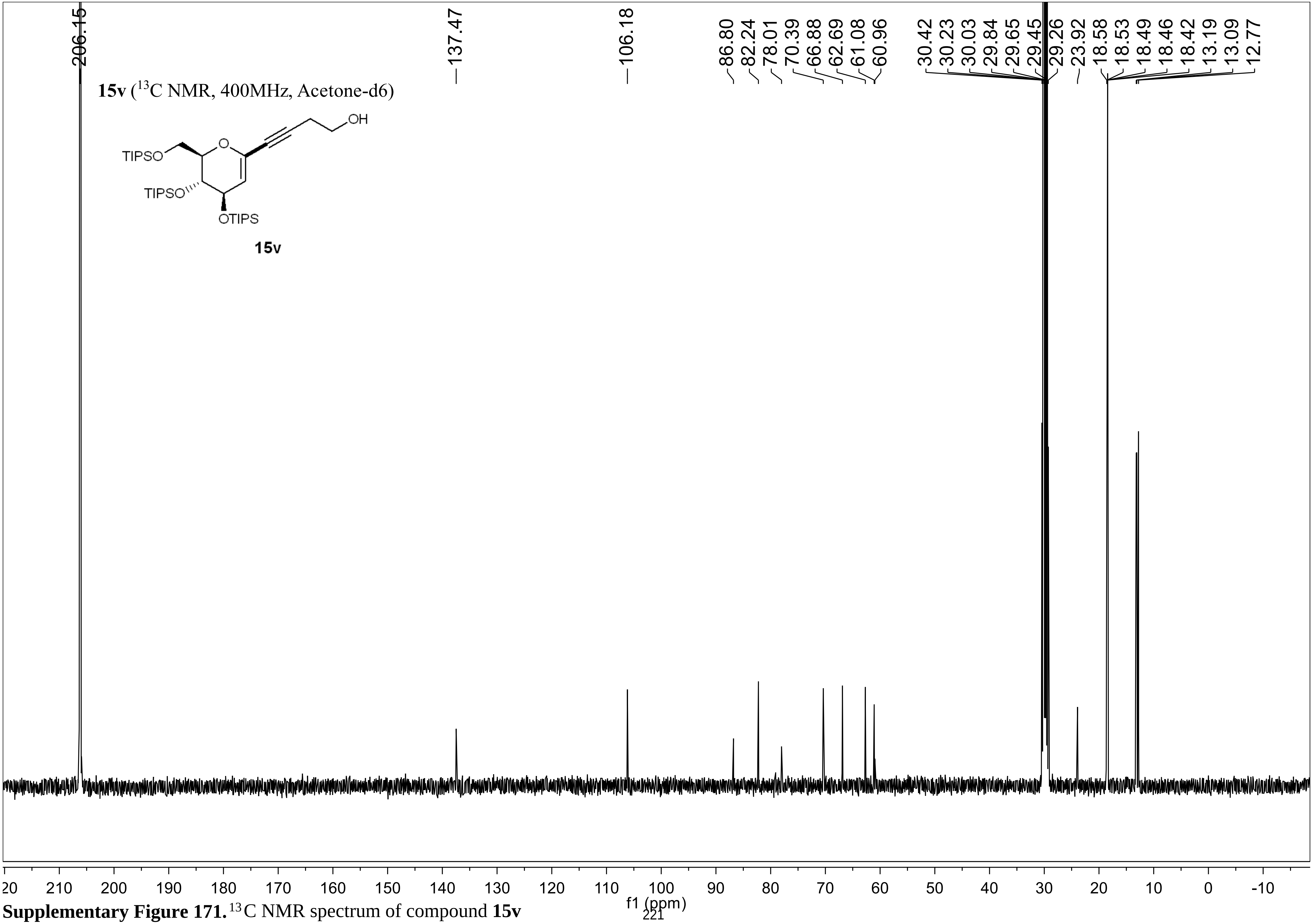

Supplementary Figure 171. ^{13}C NMR spectrum of compound **15v**

15w (^{1}H NMR, 400MHz, Acetone-d6)

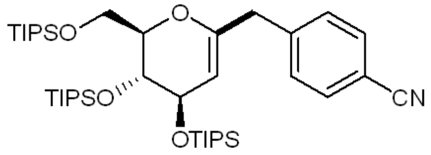

15w

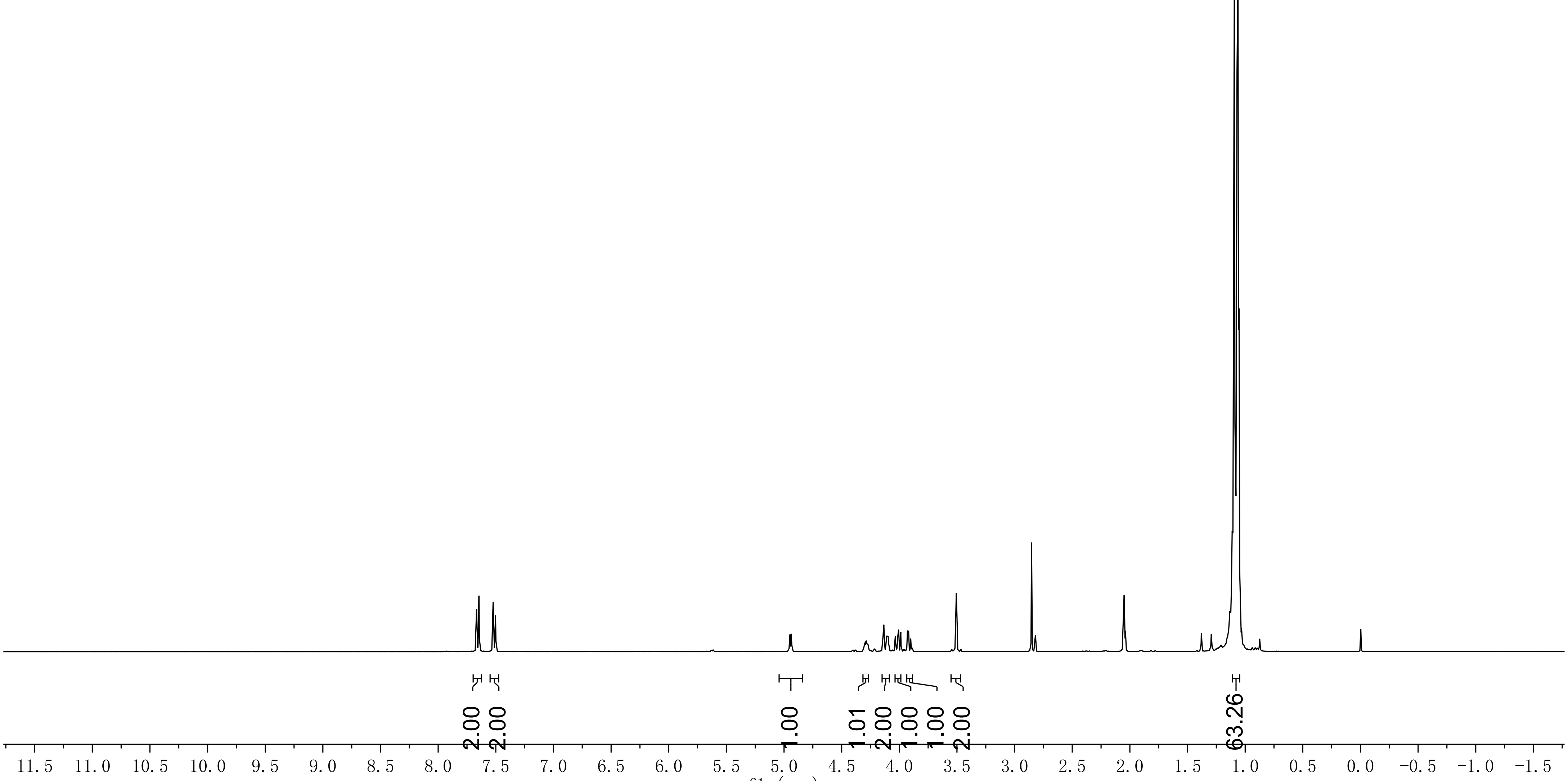

Supplementary Figure 172. ^{1}H NMR spectrum of compound **15w**

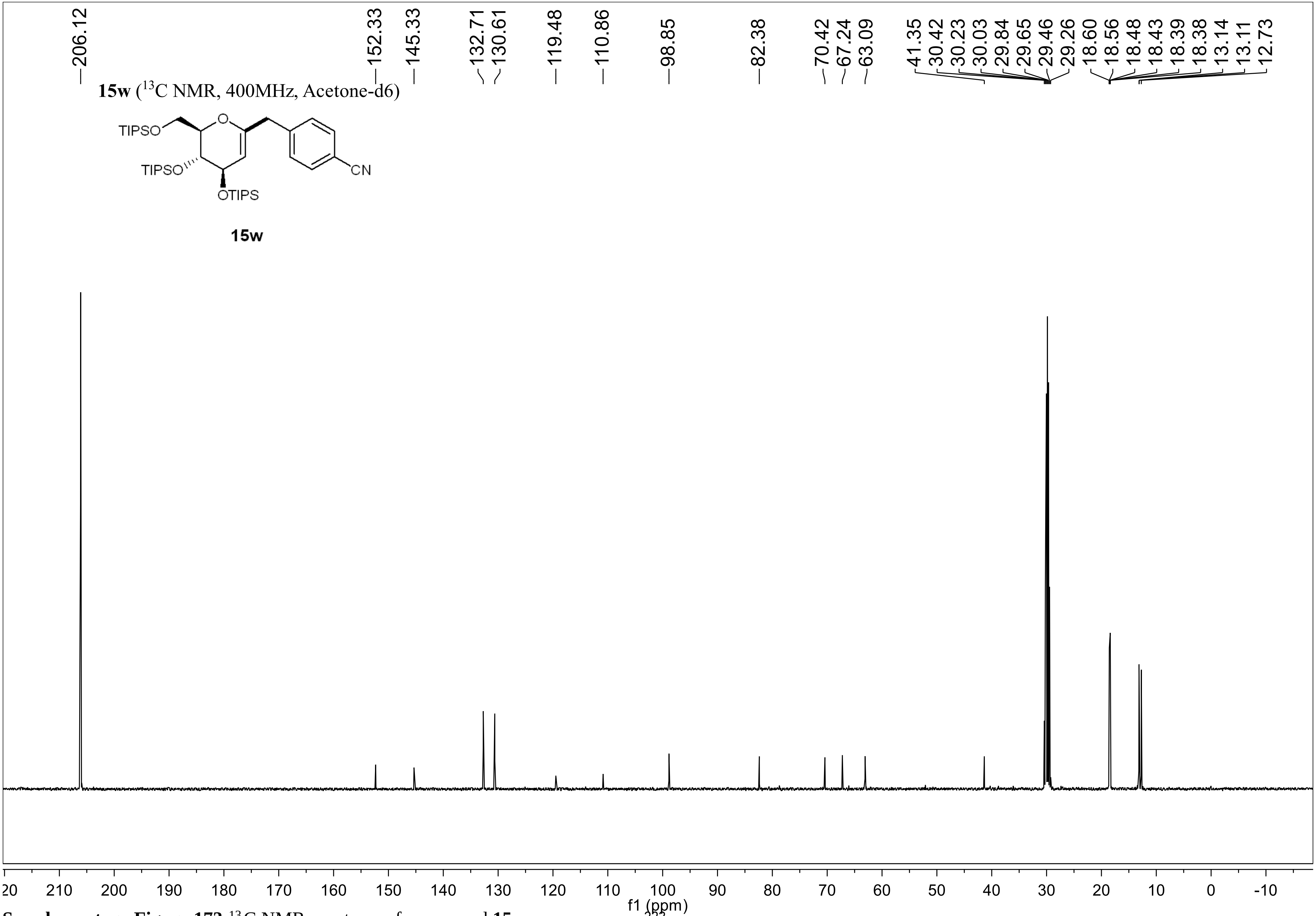

Supplementary Figure 173. ^{13}C NMR spectrum of compound **15w**

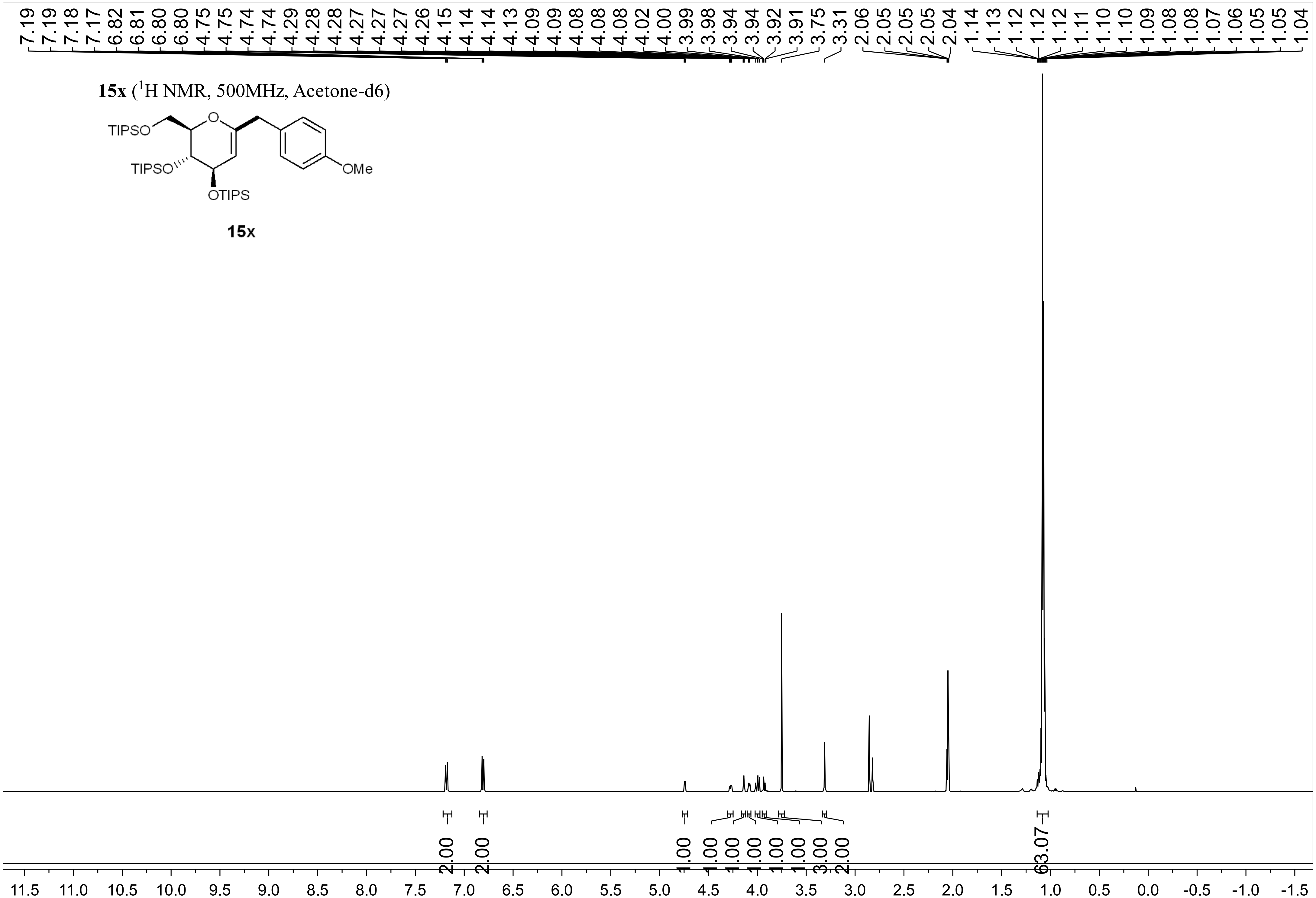

Supplementary Figure 174. ^{1}H NMR spectrum of compound **15x**

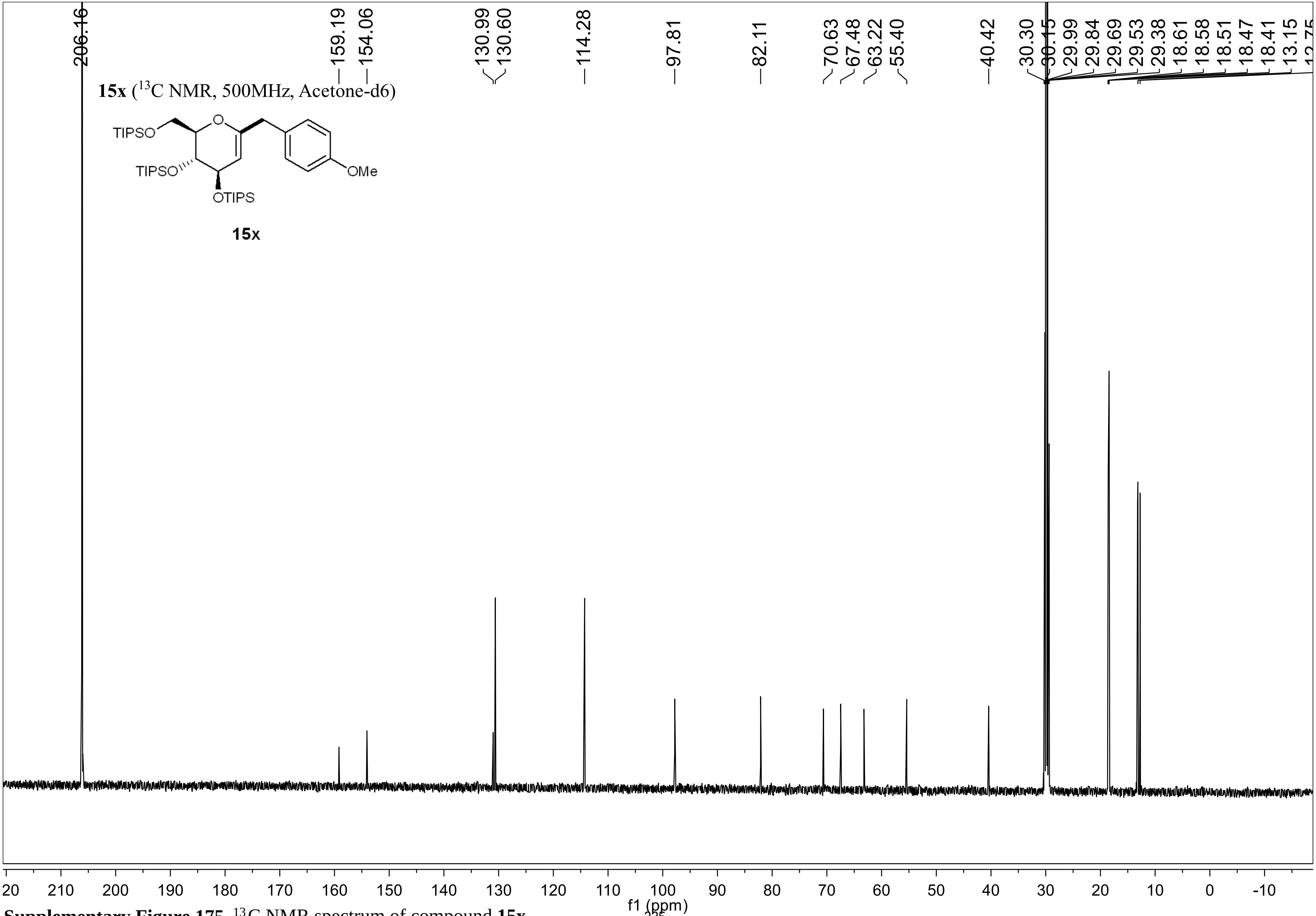

Supplementary Figure 175. ^{13}C NMR spectrum of compound **15x**

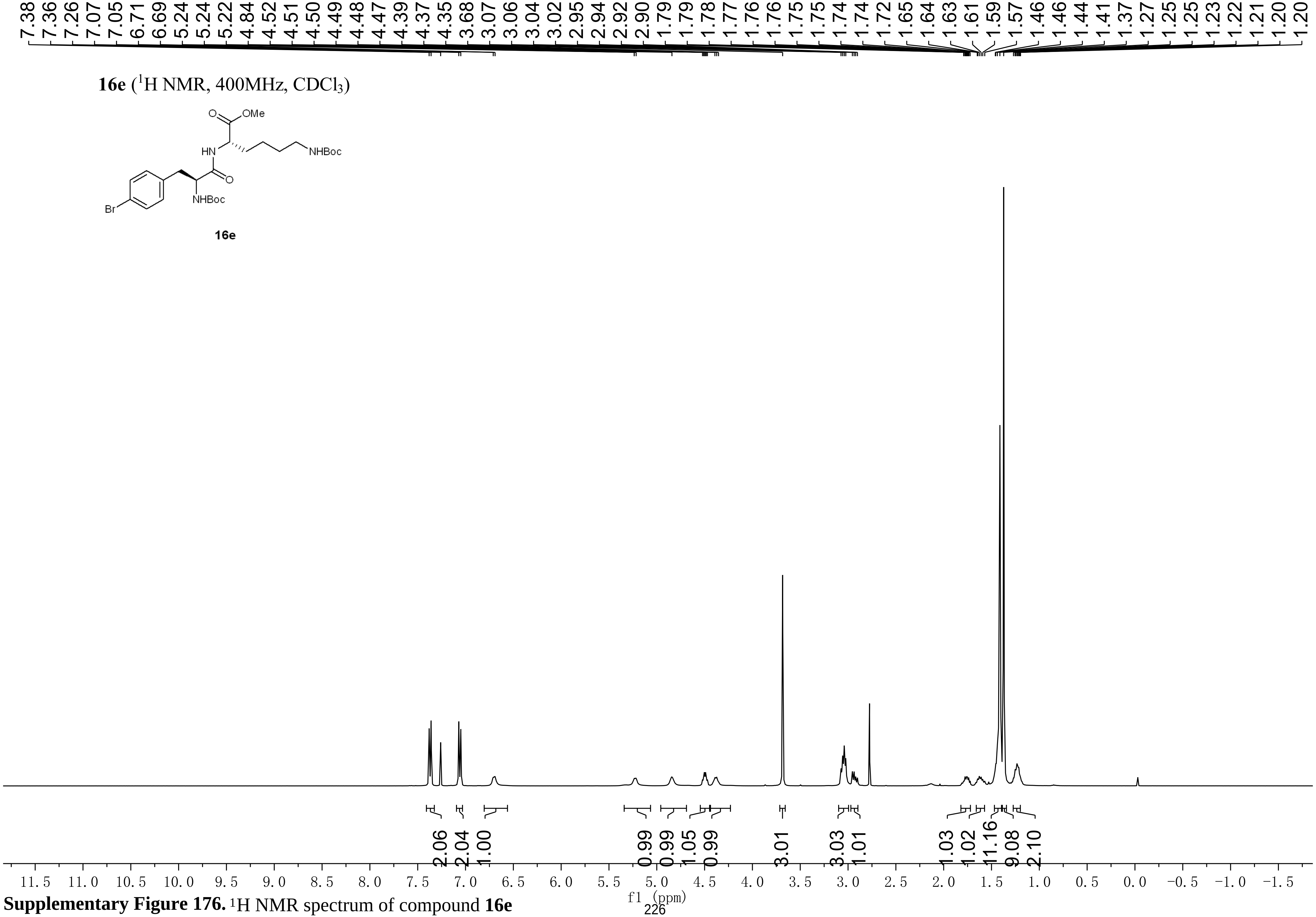

Supplementary Figure 176. ^{1}H NMR spectrum of compound **16e**

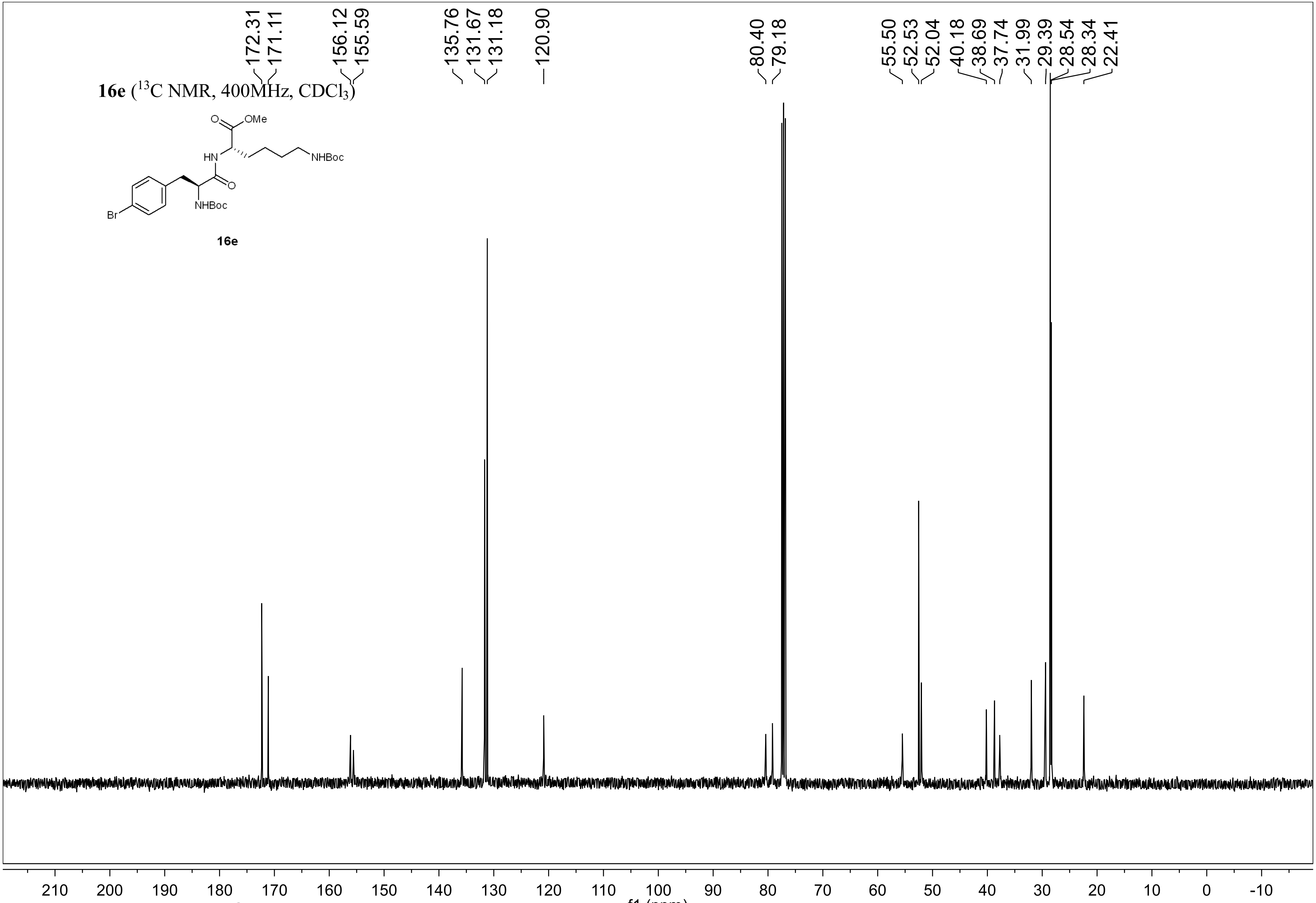

Supplementary Figure 177. ^{13}C NMR spectrum of compound **16e**

16f (^{1}H NMR, 400MHz, $CDCl_3$)

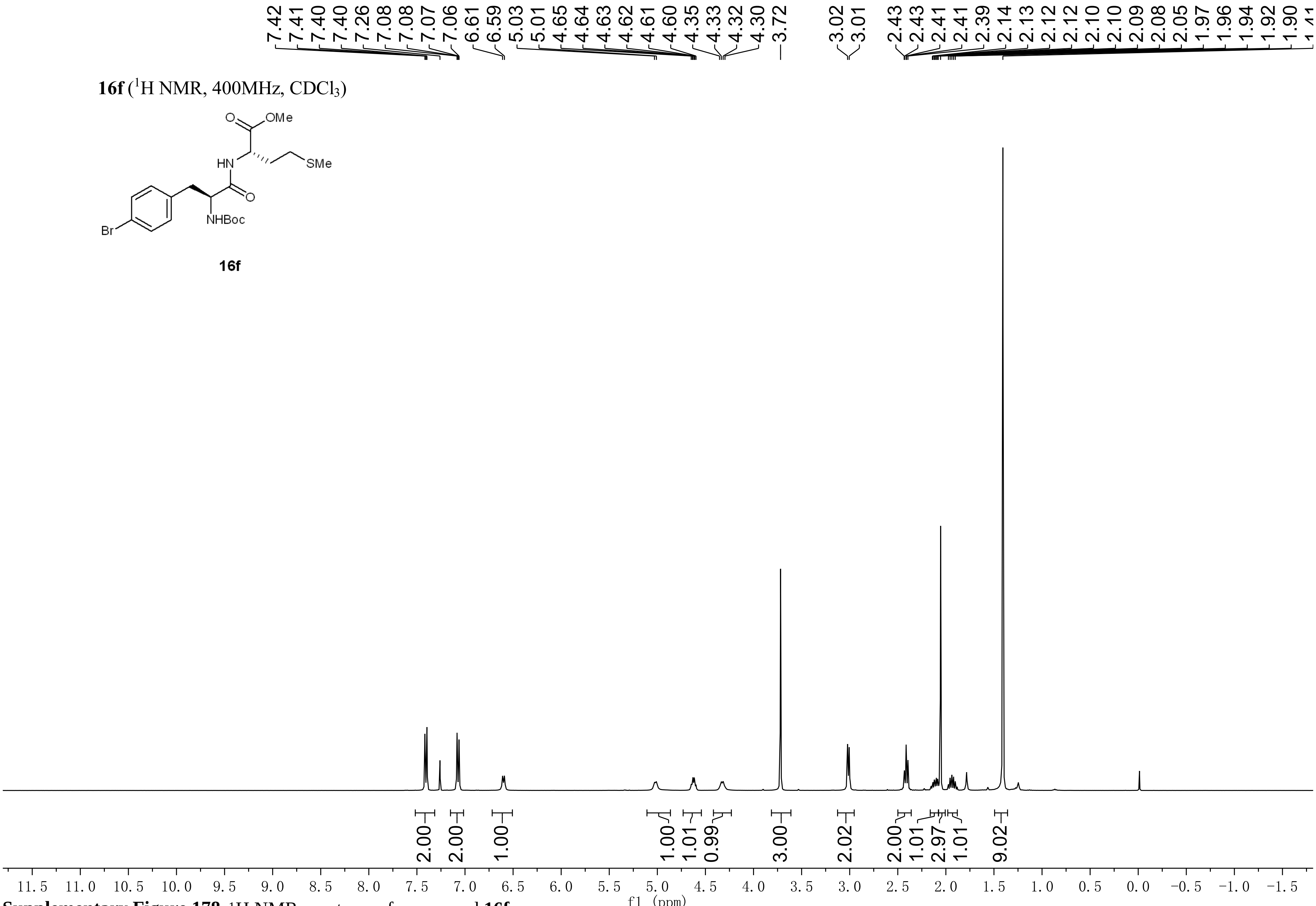

Supplementary Figure 178. ^{1}H NMR spectrum of compound **16f**

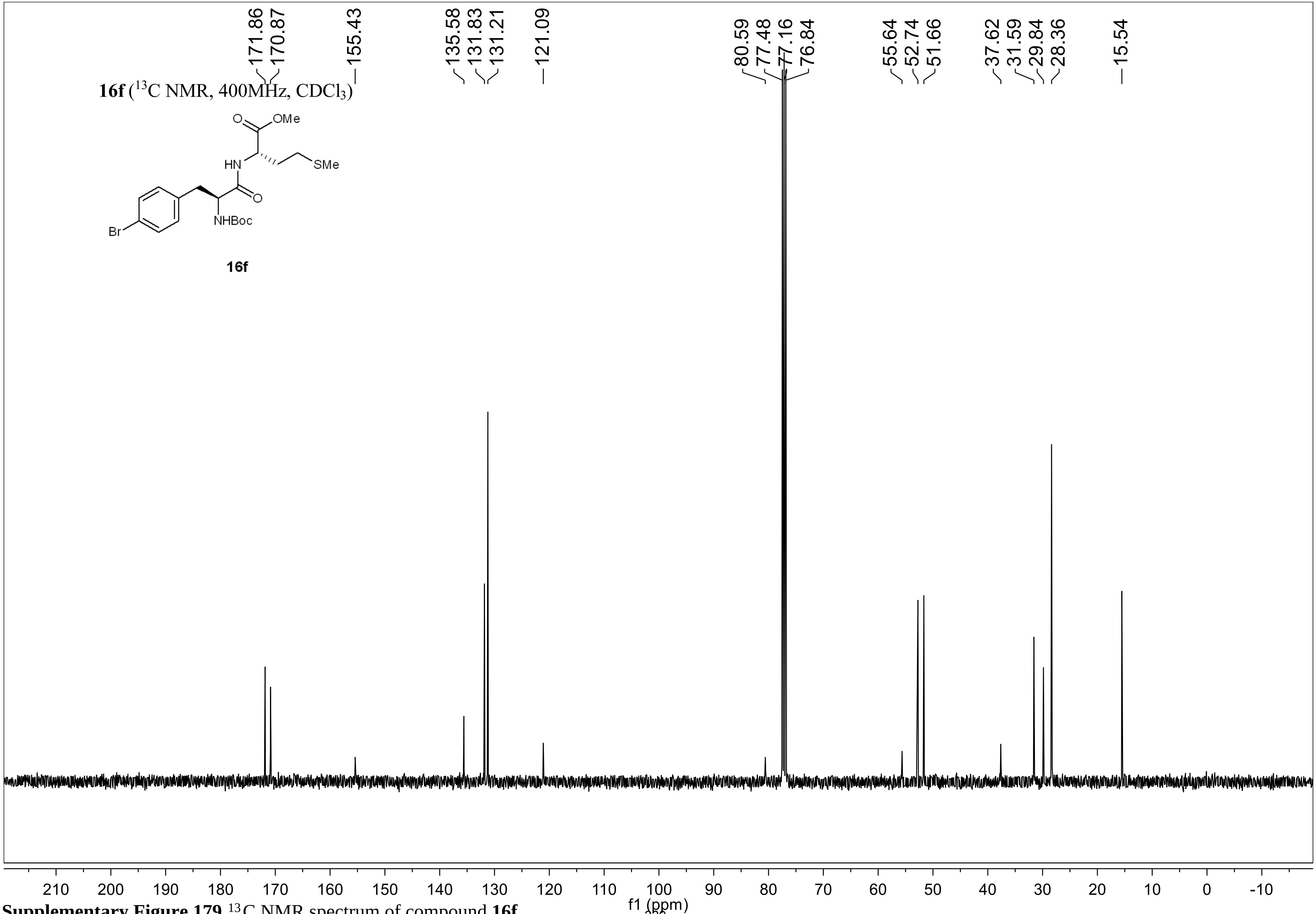

Supplementary Figure 179. ^{13}C NMR spectrum of compound **16f**

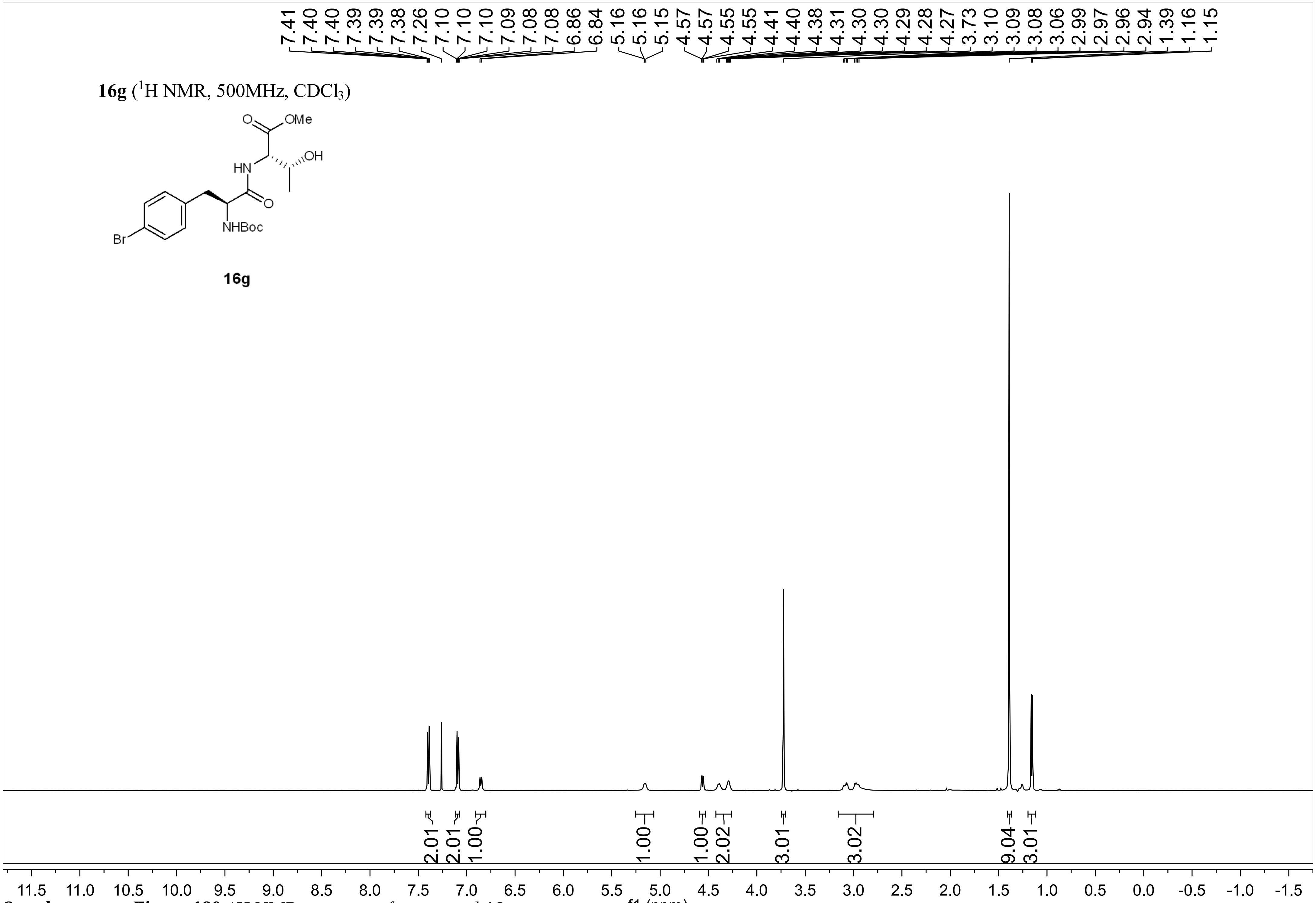

Supplementary Figure 180. ^{1}H NMR spectrum of compound **16g**

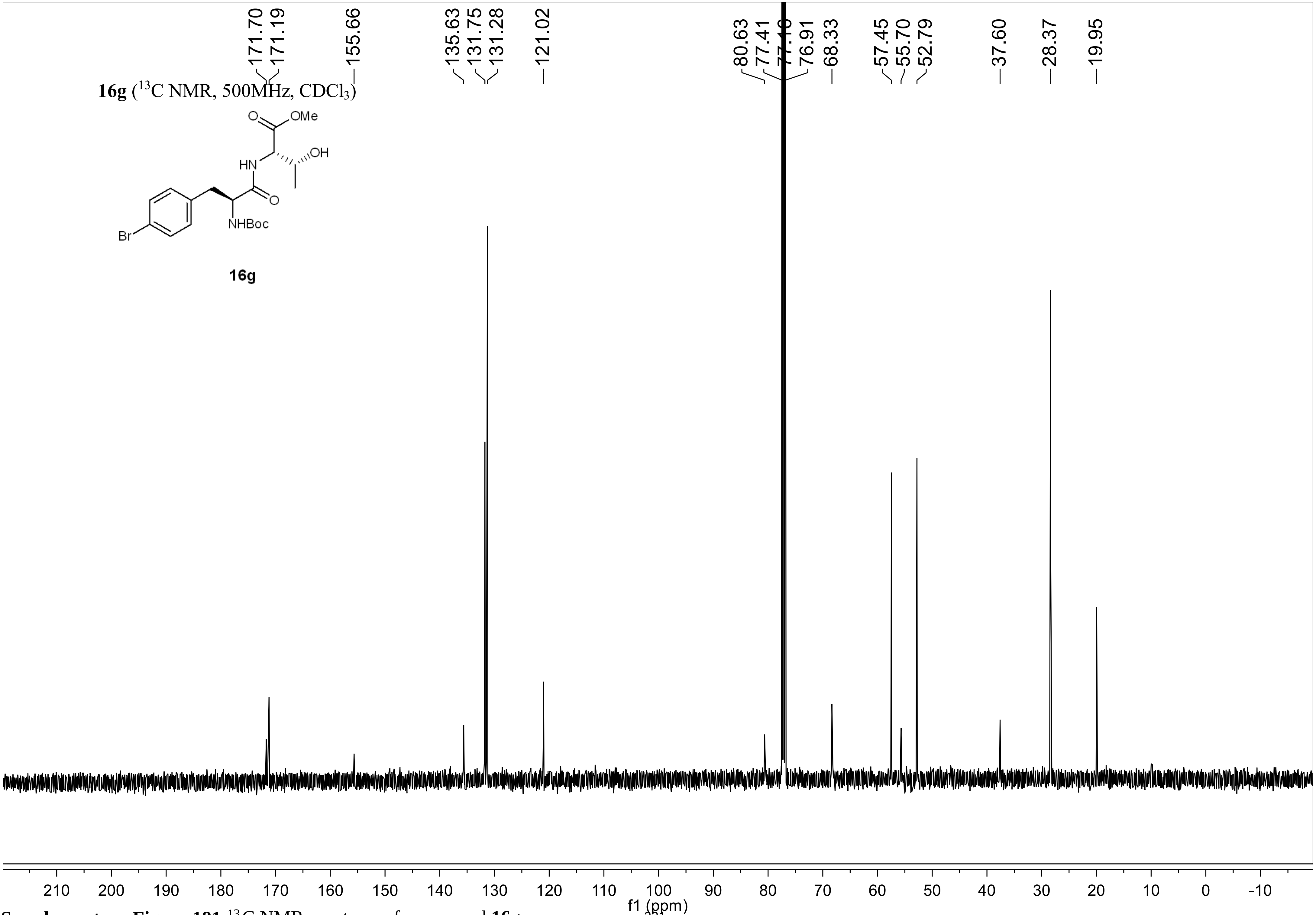

Supplementary Figure 181. ^{13}C NMR spectrum of compound **16g**

16h (^{1}H NMR, 400MHz, $CDCl_3$)

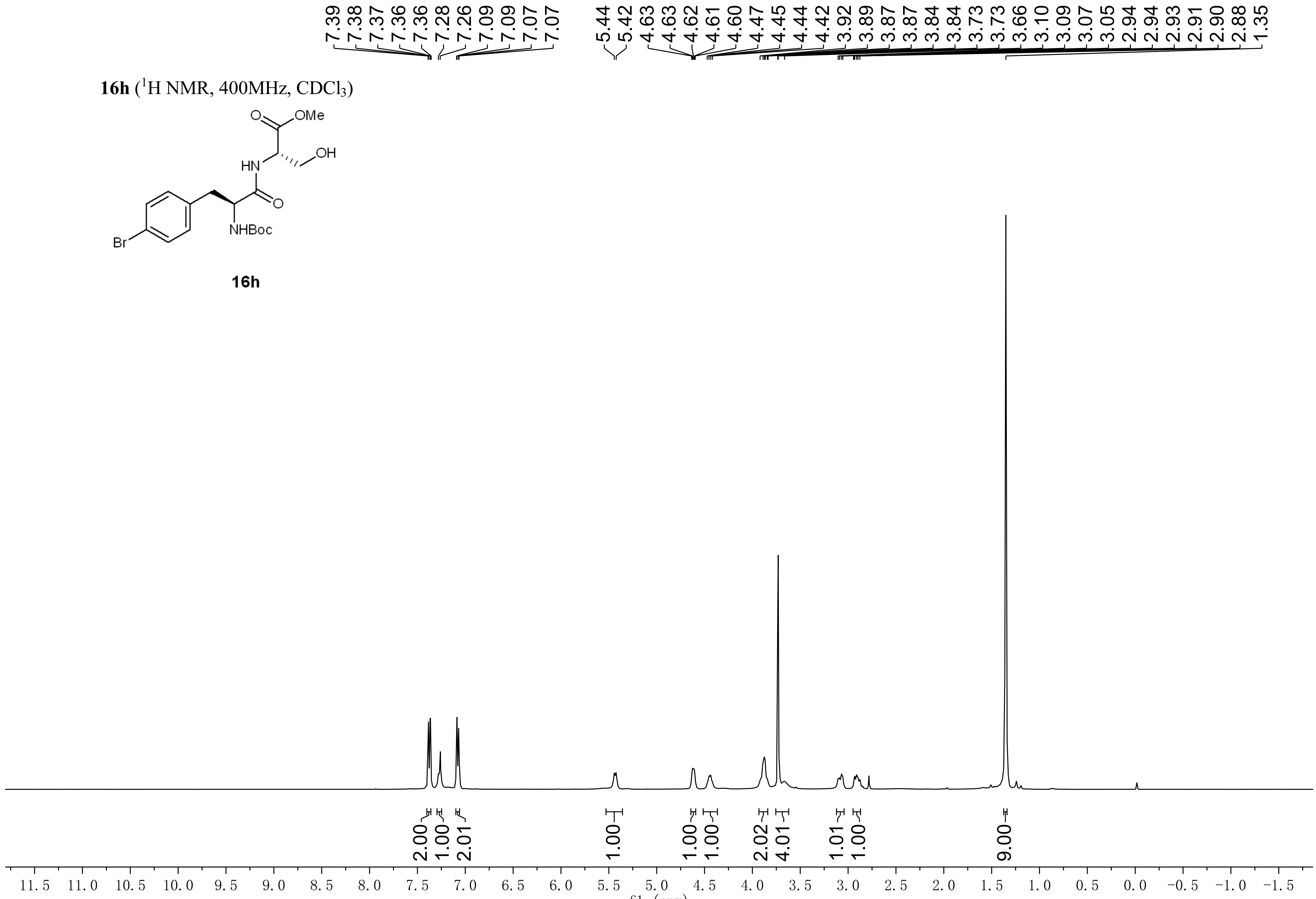

Supplementary Figure 182. ^{1}H NMR spectrum of compound **16h**

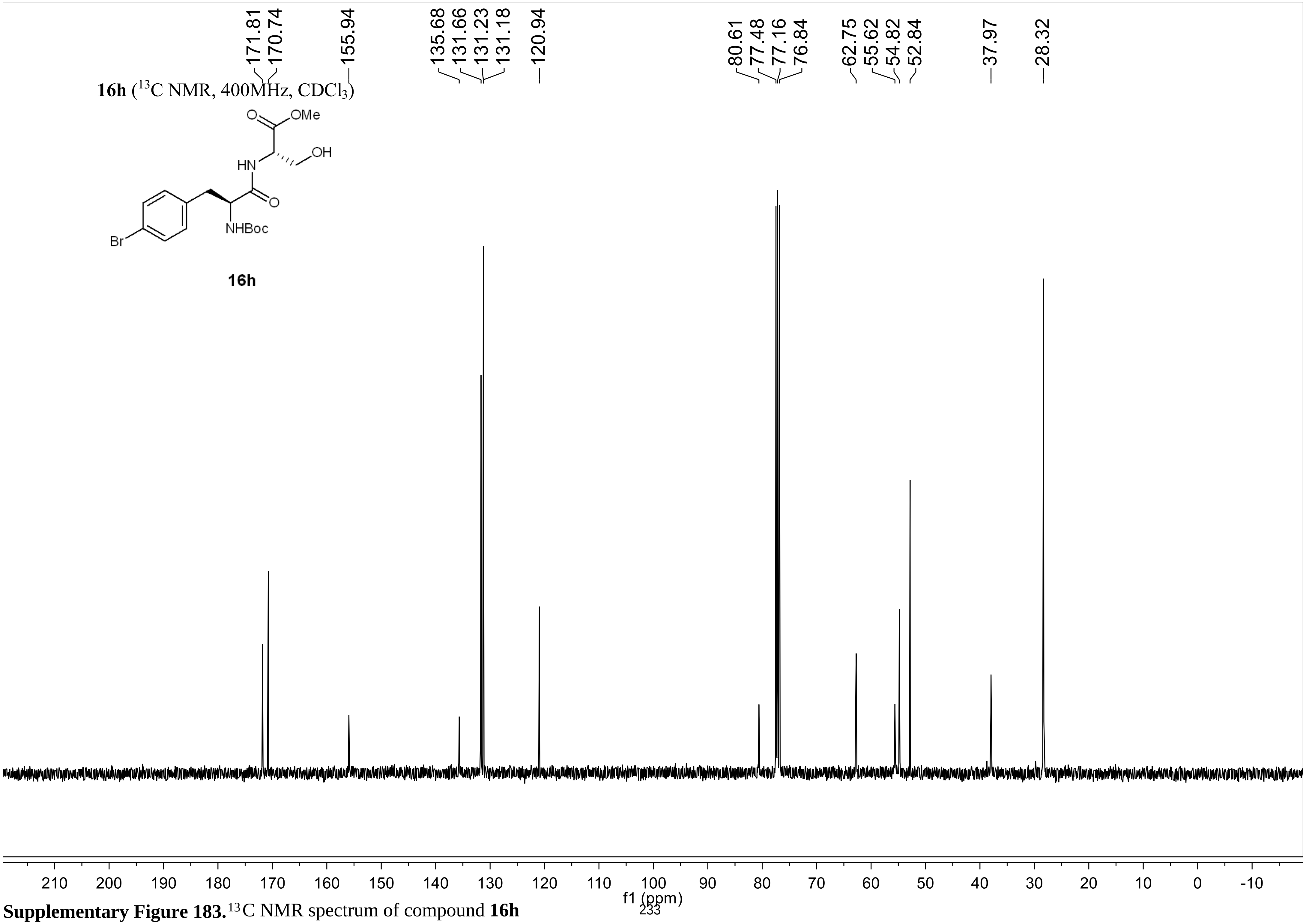

Supplementary Figure 183. ^{13}C NMR spectrum of compound **16h**

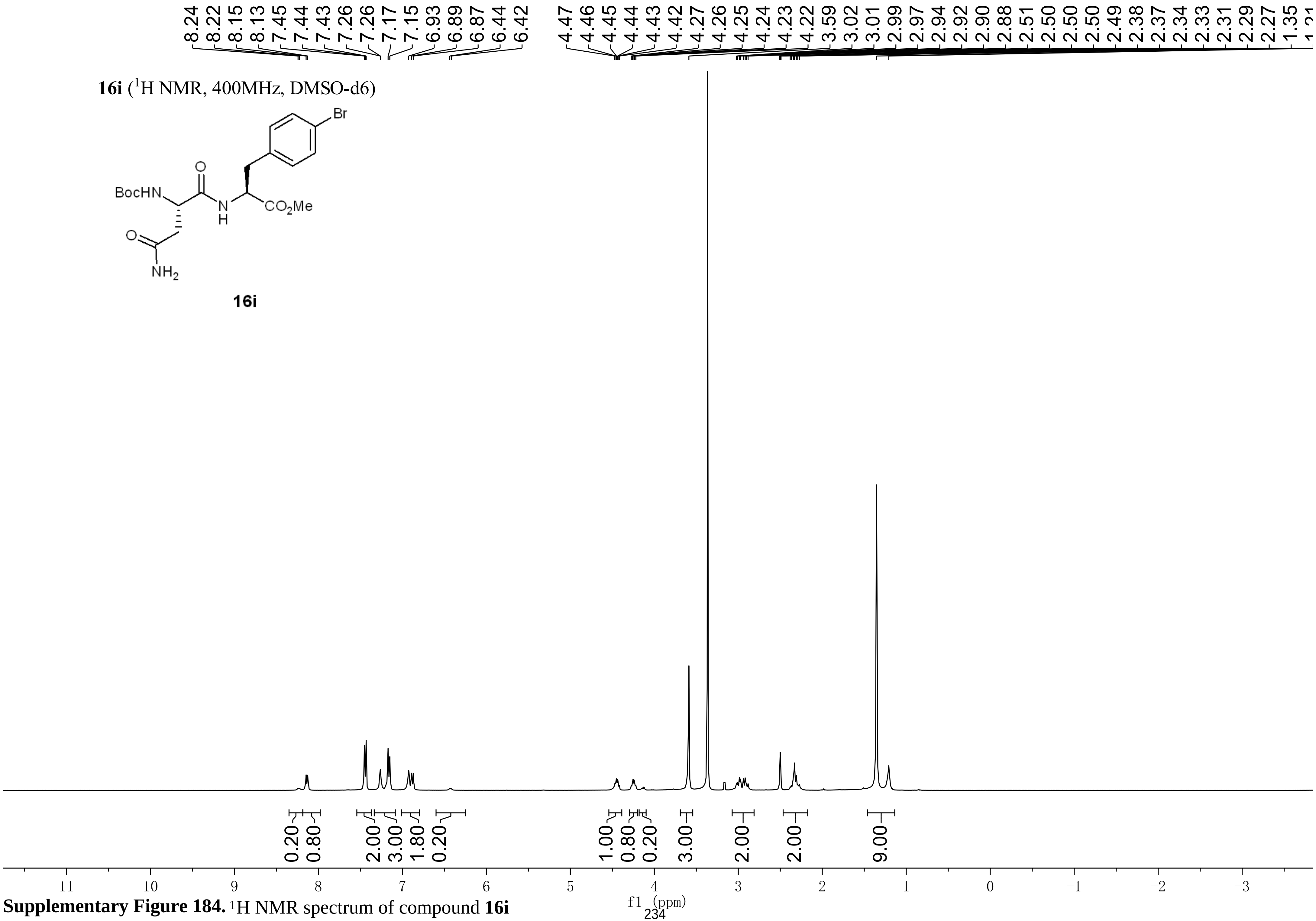

Supplementary Figure 184. ^{1}H NMR spectrum of compound **16i**

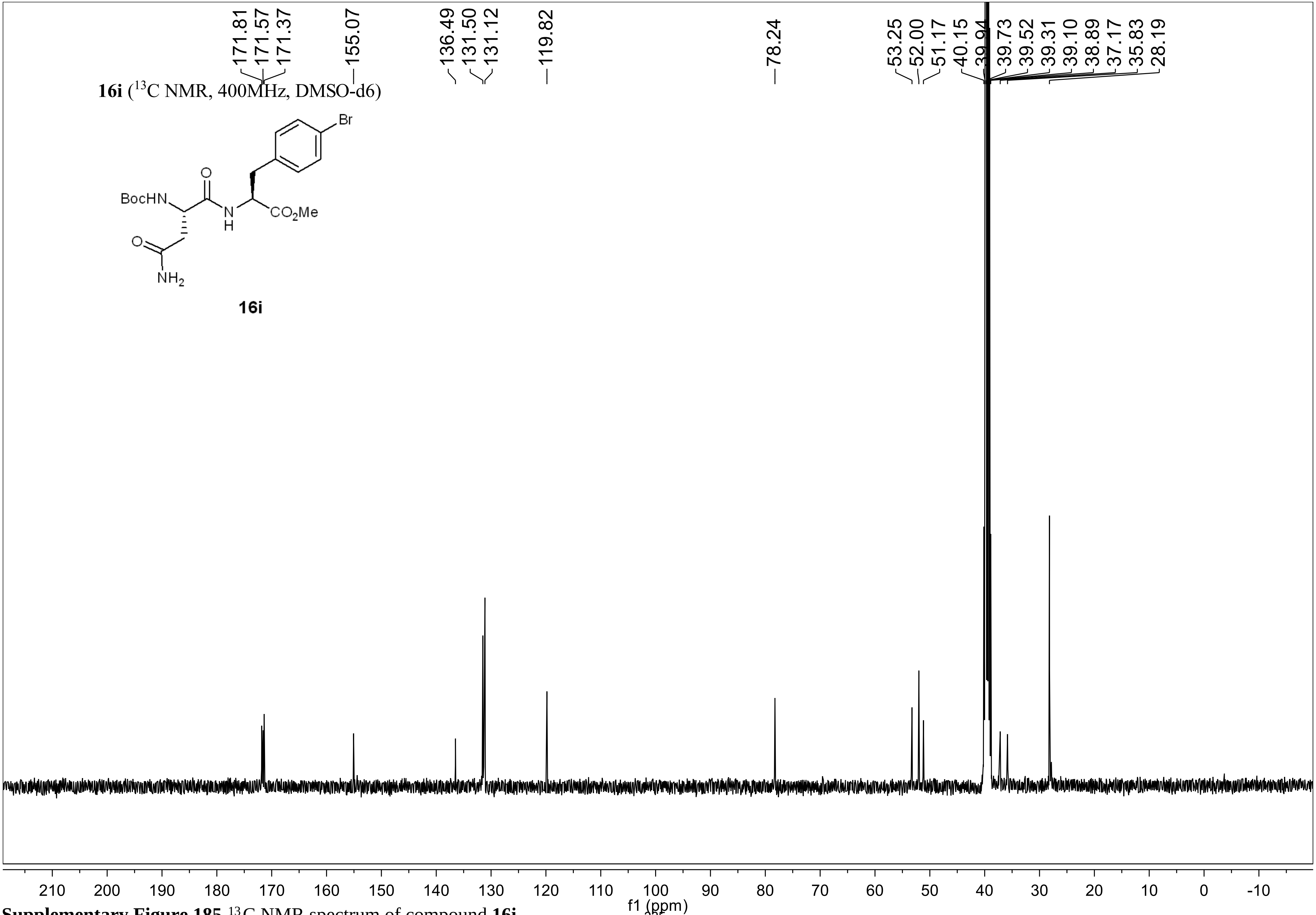

Supplementary Figure 185. ^{13}C NMR spectrum of compound **16i**

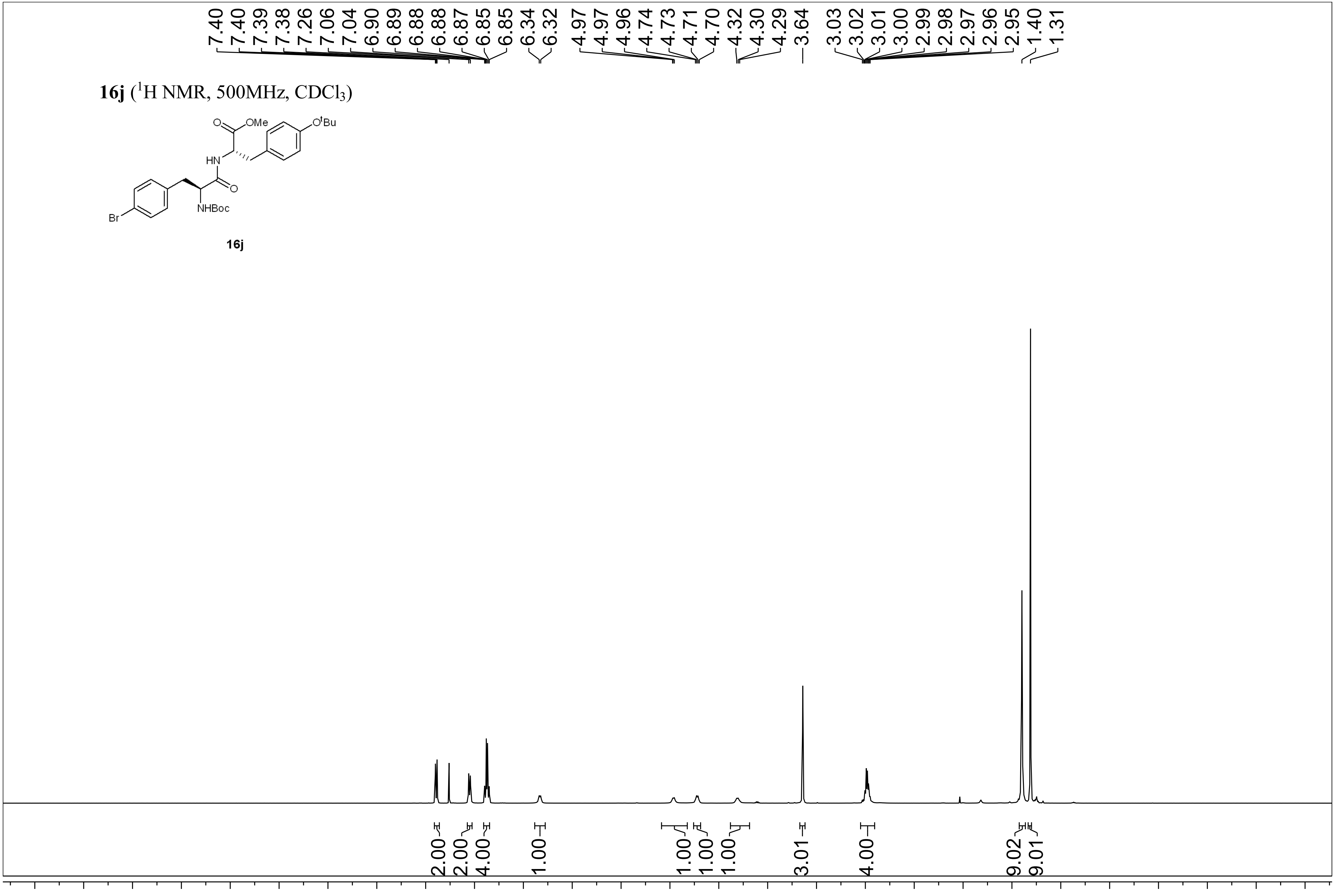

Supplementary Figure 186. ^{1}H NMR spectrum of compound **16j**

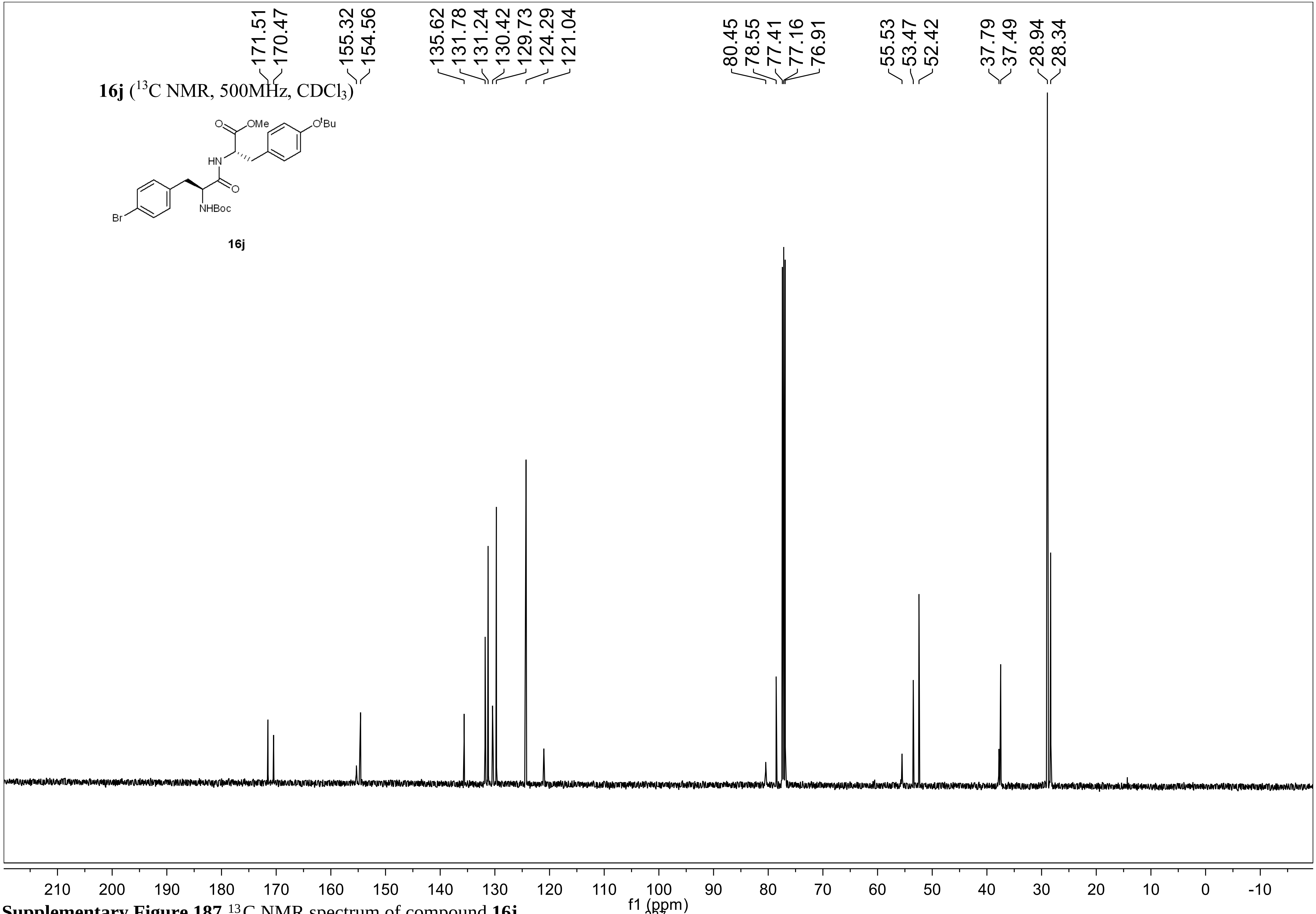

Supplementary Figure 187. ^{13}C NMR spectrum of compound **16j**

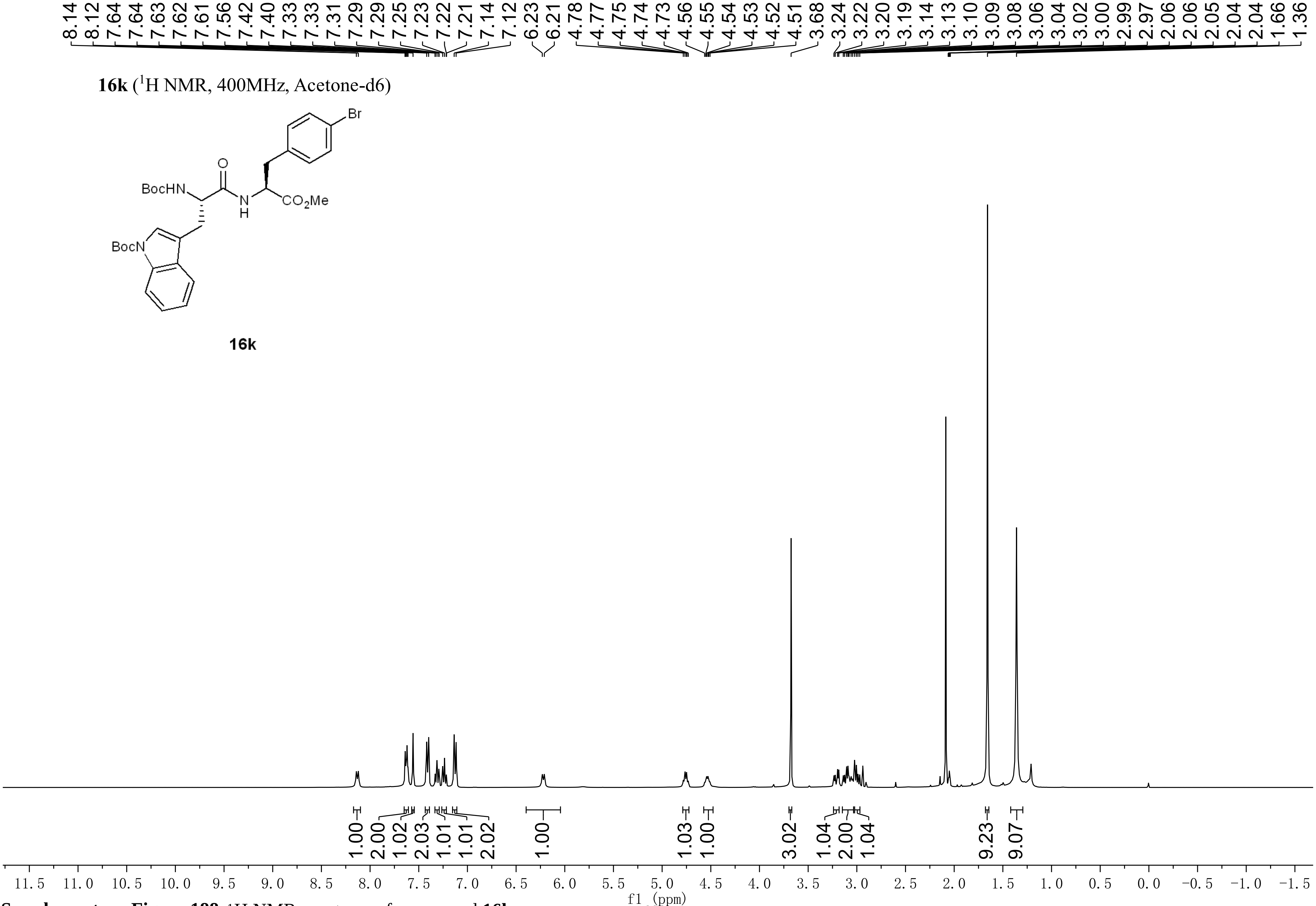

Supplementary Figure 188. ^{1}H NMR spectrum of compound **16k**

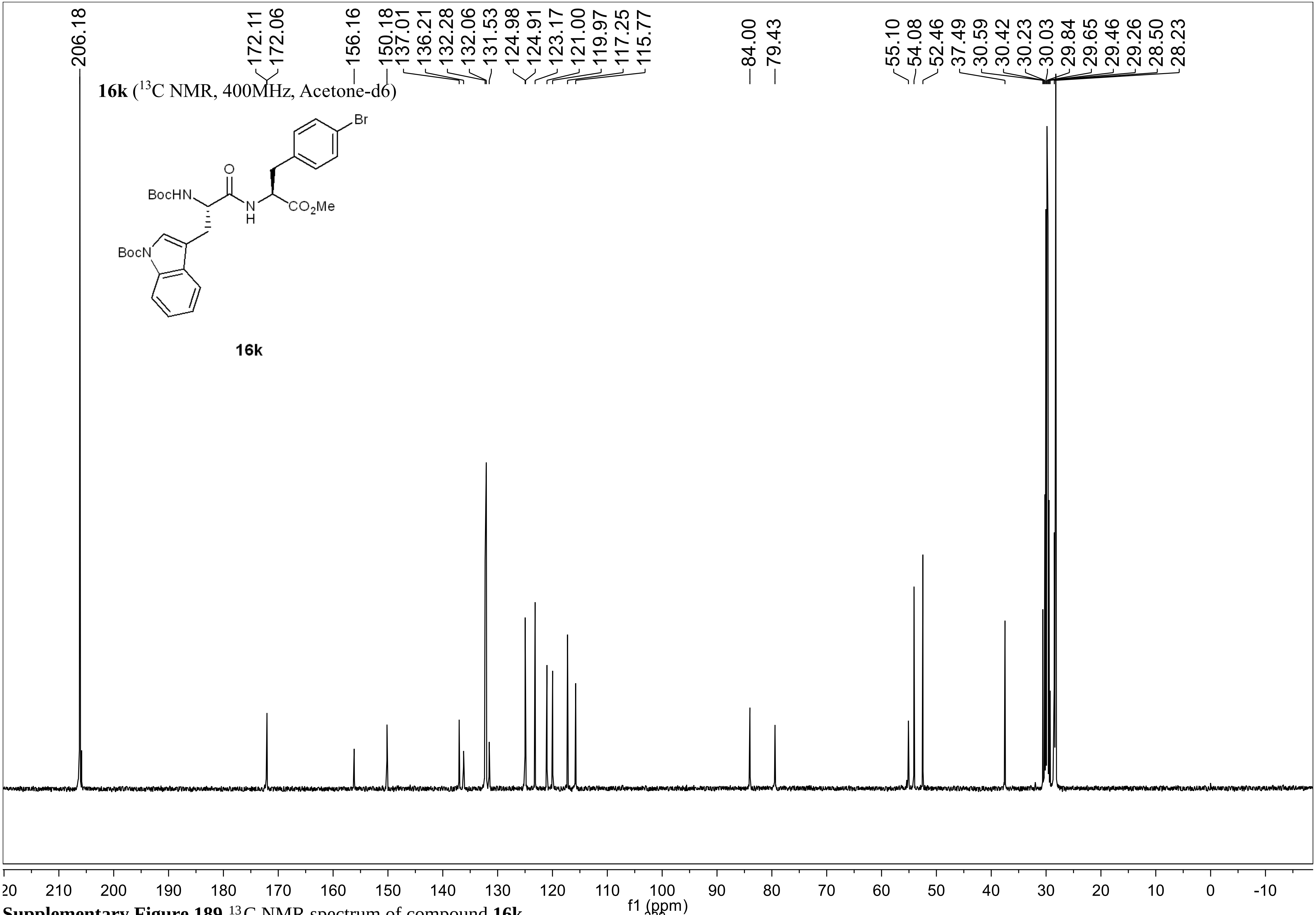

Supplementary Figure 189. ^{13}C NMR spectrum of compound **16k**

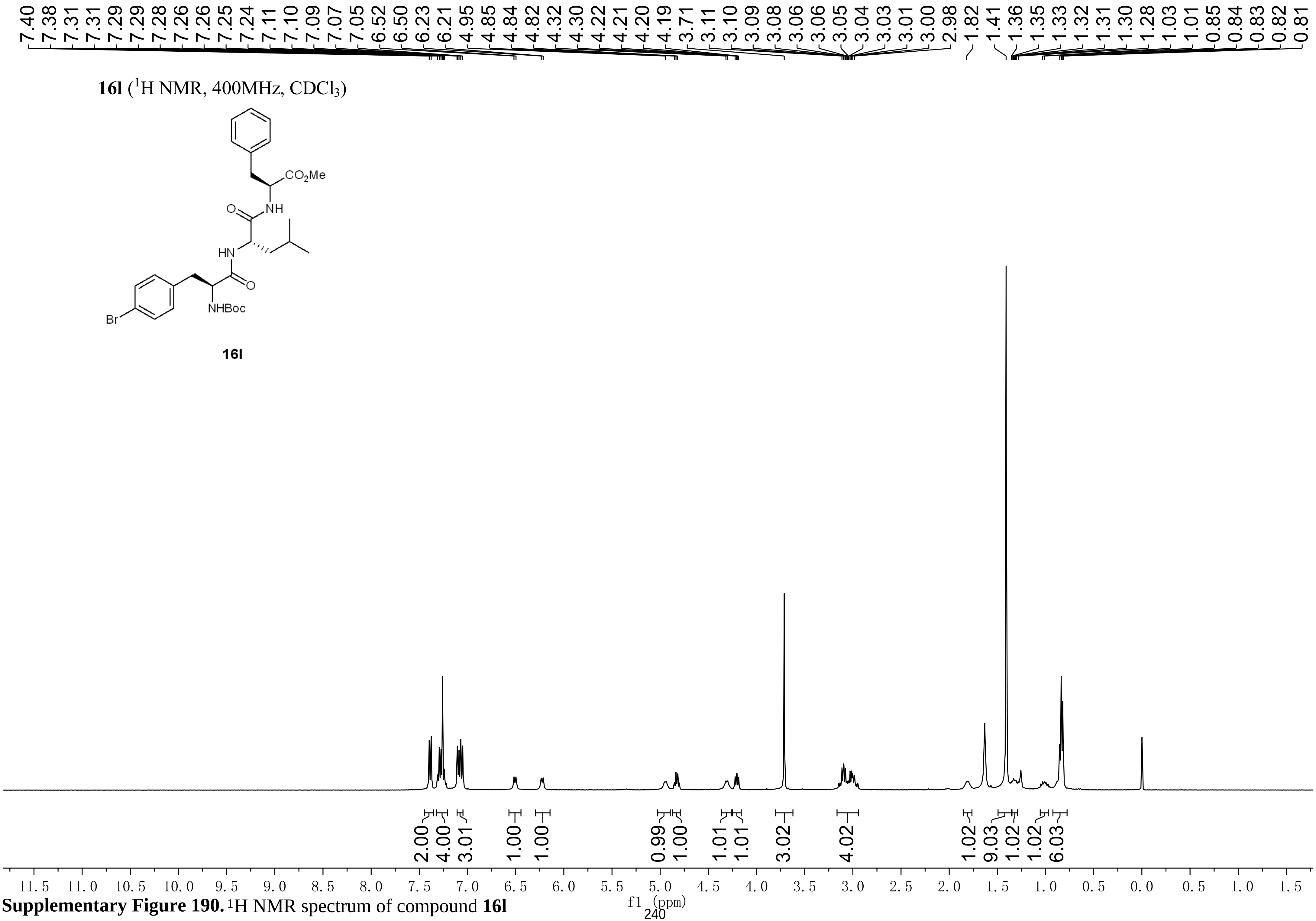

Supplementary Figure 190. ¹H NMR spectrum of compound **16l**

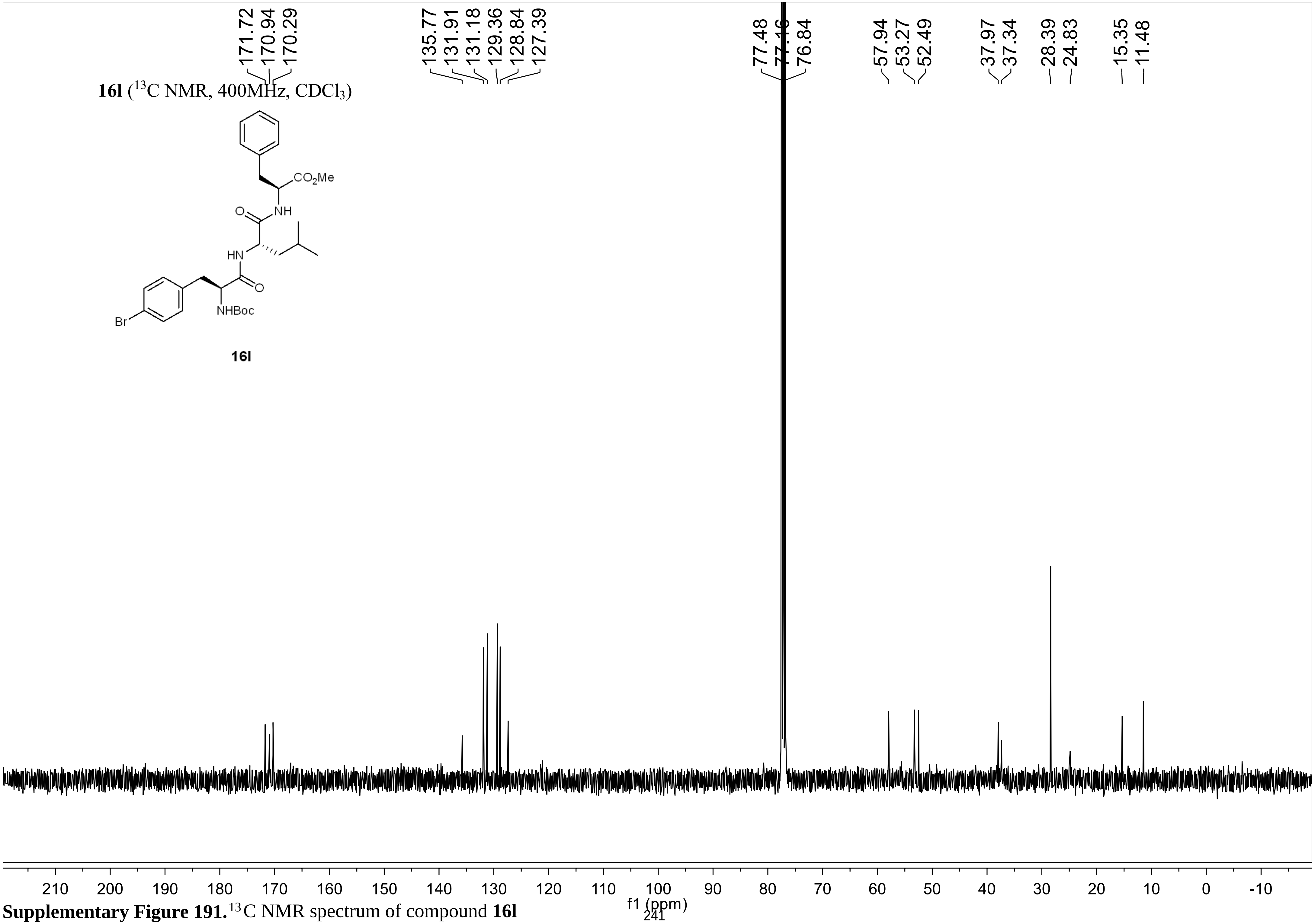

Supplementary Figure 191. ^{13}C NMR spectrum of compound **16l**

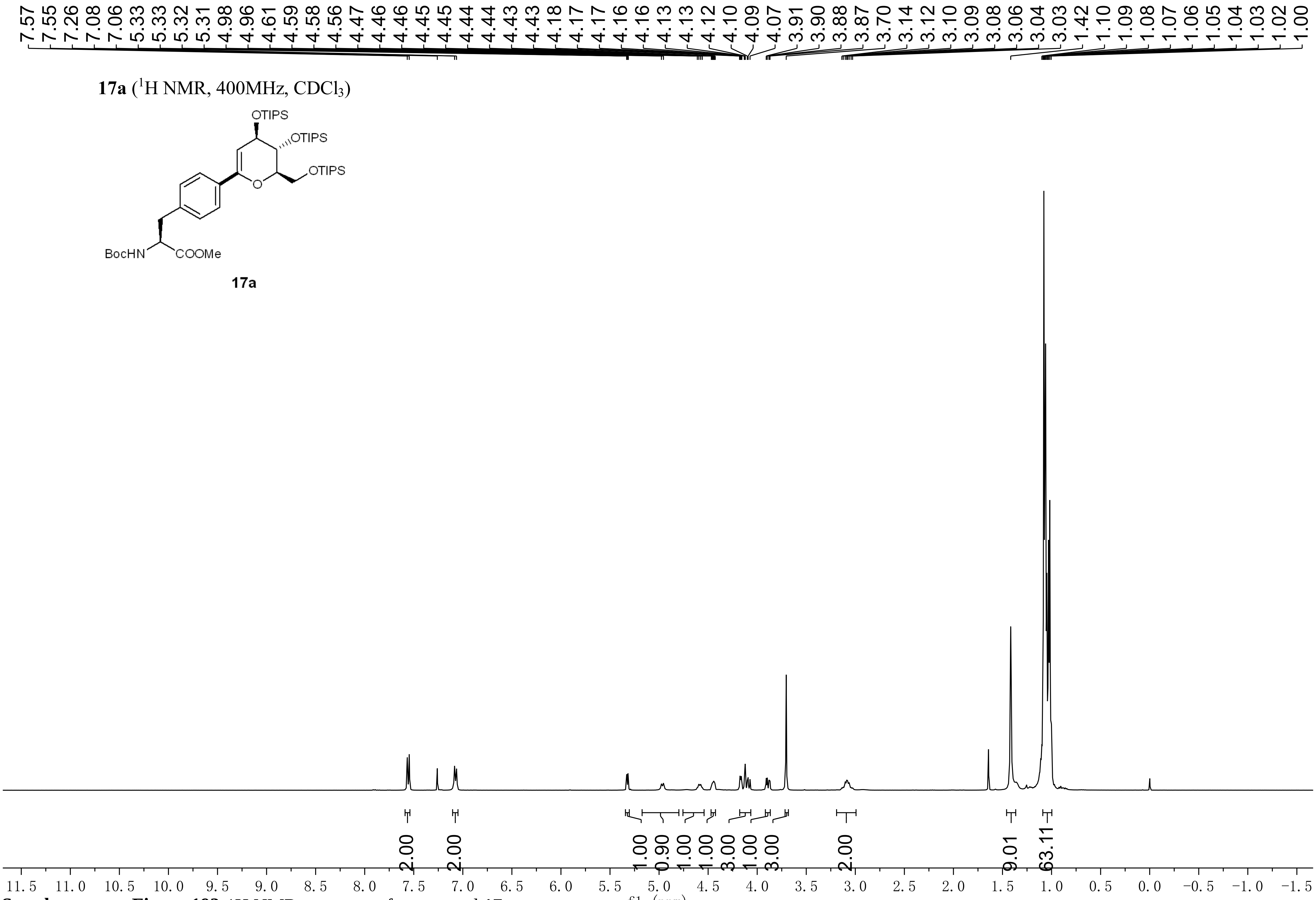

Supplementary Figure 192. ^{1}H NMR spectrum of compound **17a**

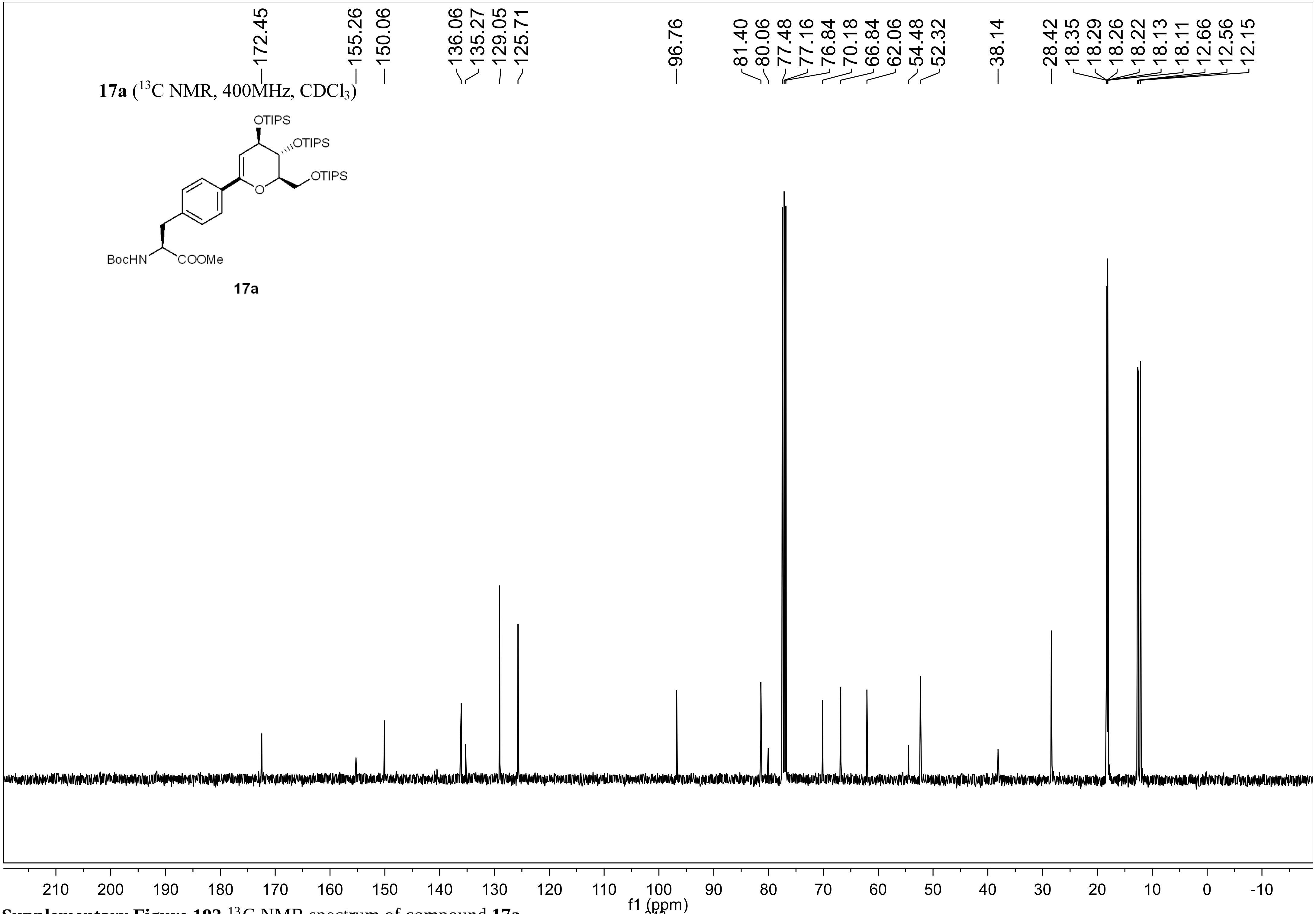

Supplementary Figure 193. ^{13}C NMR spectrum of compound **17a**

17b (^{1}H NMR, 400MHz, Acetone-d6)

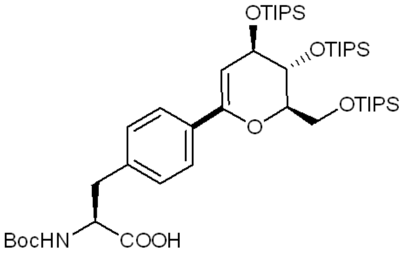

17b

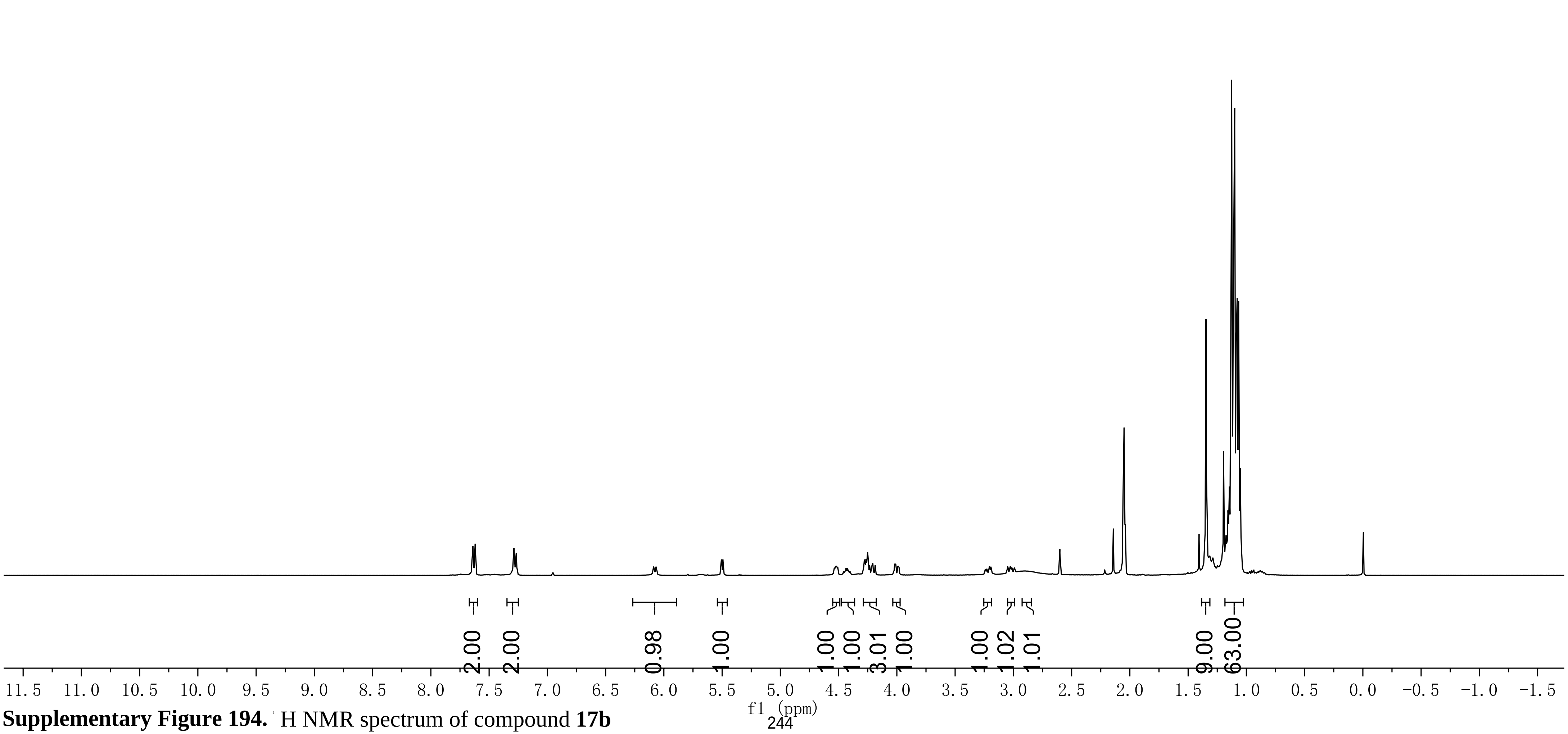

Supplementary Figure 194. ^{1}H NMR spectrum of compound **17b**

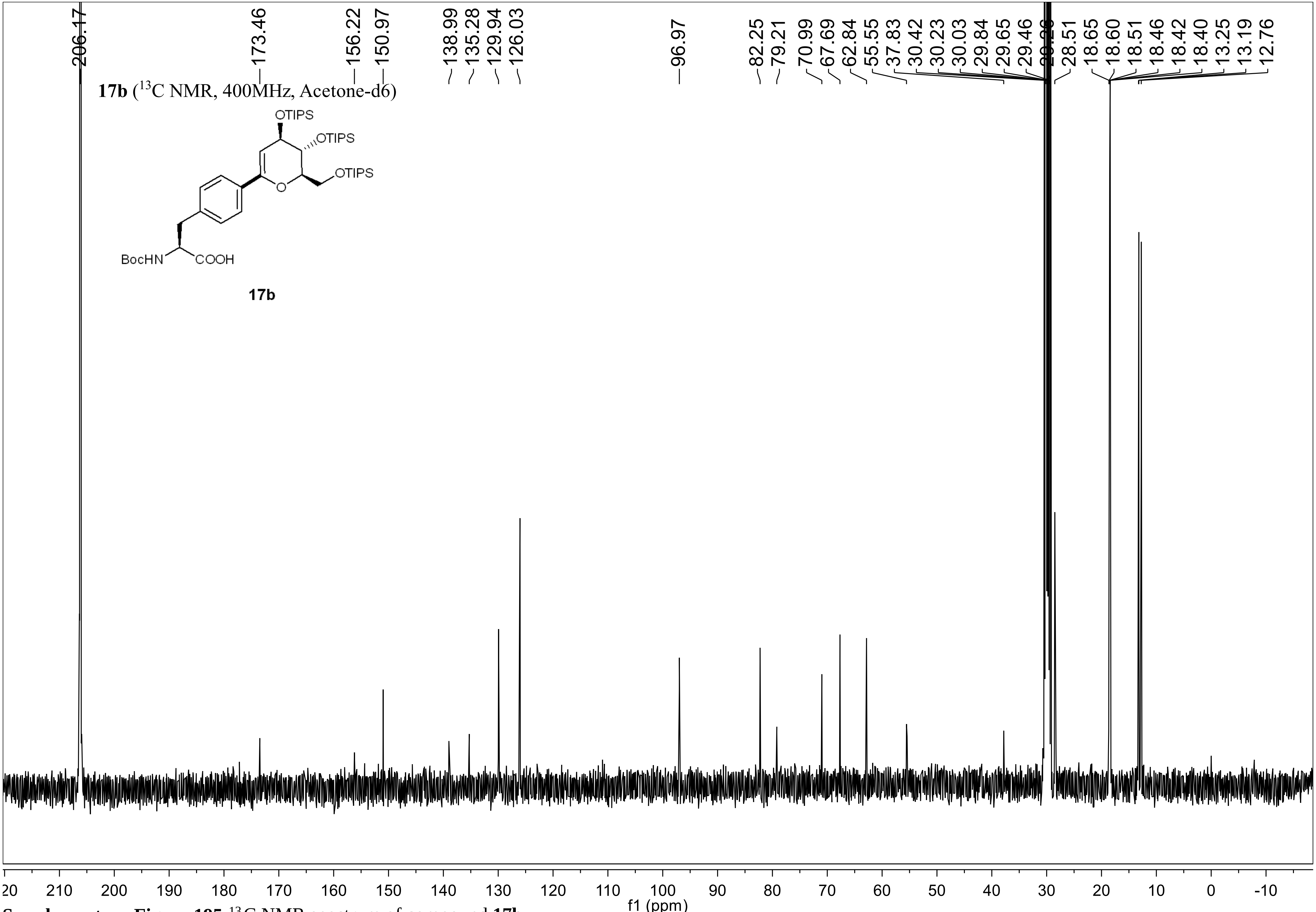

Supplementary Figure 195. ^{13}C NMR spectrum of compound **17b**

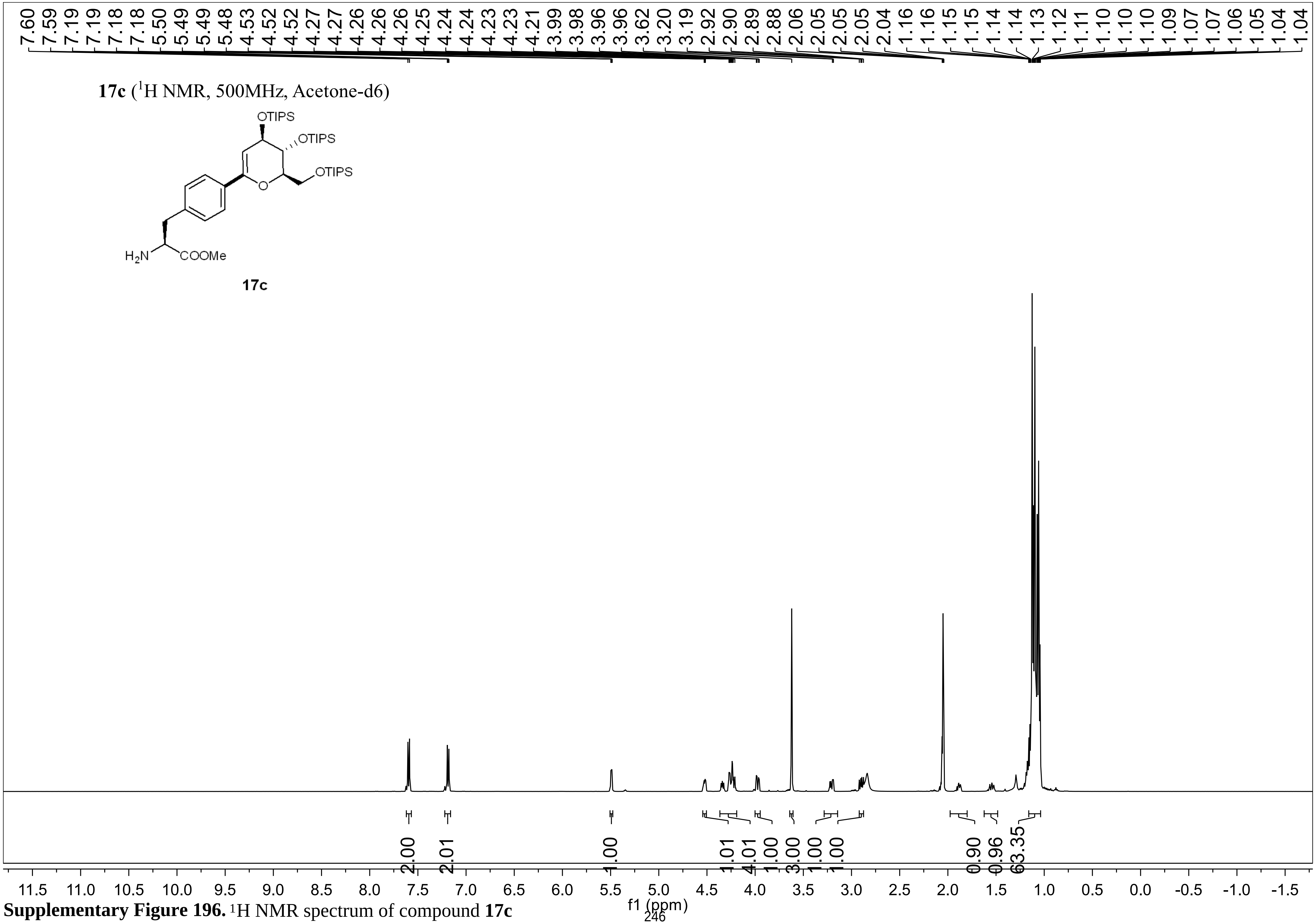

Supplementary Figure 196. ^{1}H NMR spectrum of compound **17c**

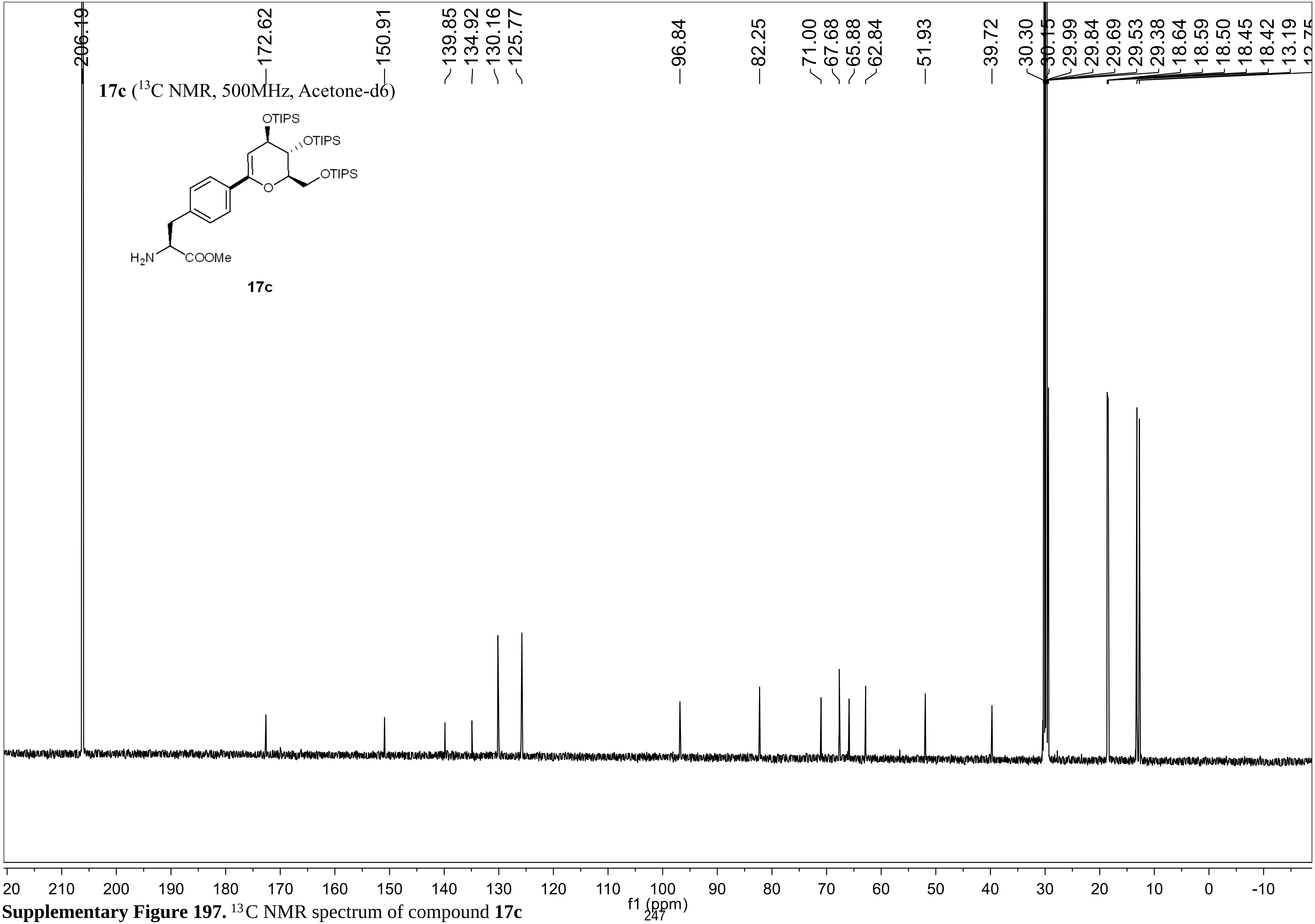

Supplementary Figure 197. ^{13}C NMR spectrum of compound **17c**

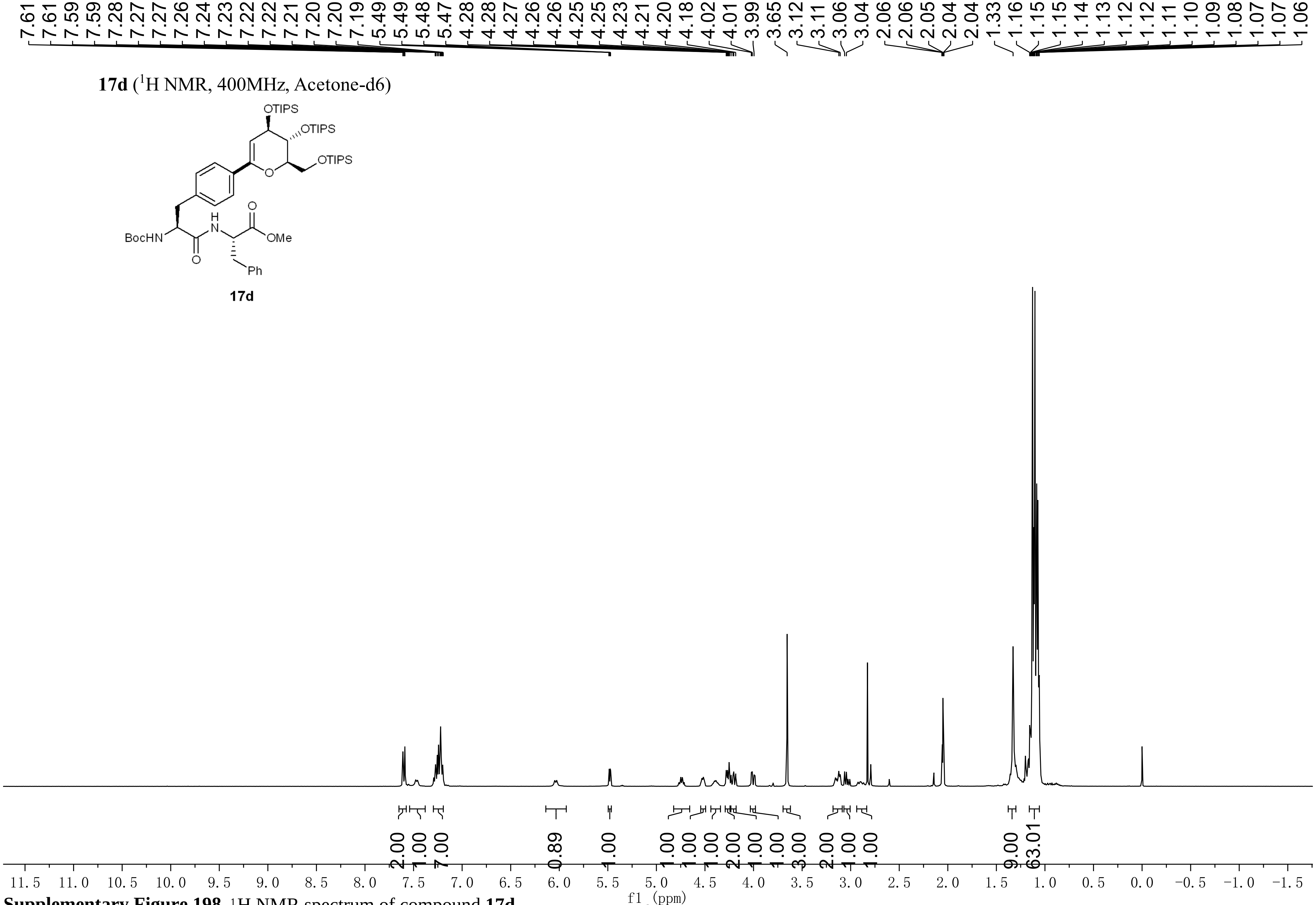

Supplementary Figure 198. ^{1}H NMR spectrum of compound **17d**

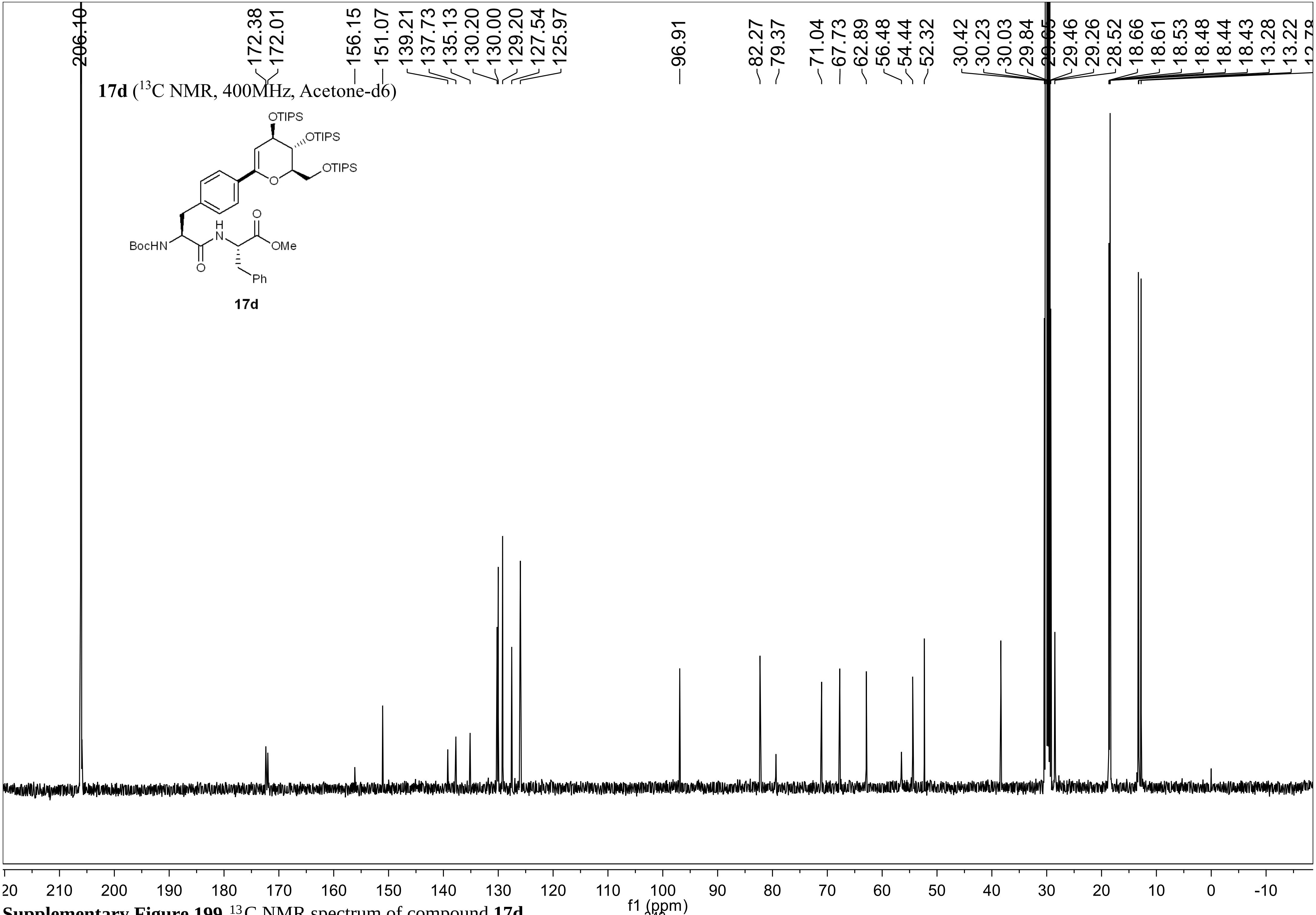

Supplementary Figure 199. ^{13}C NMR spectrum of compound **17d**

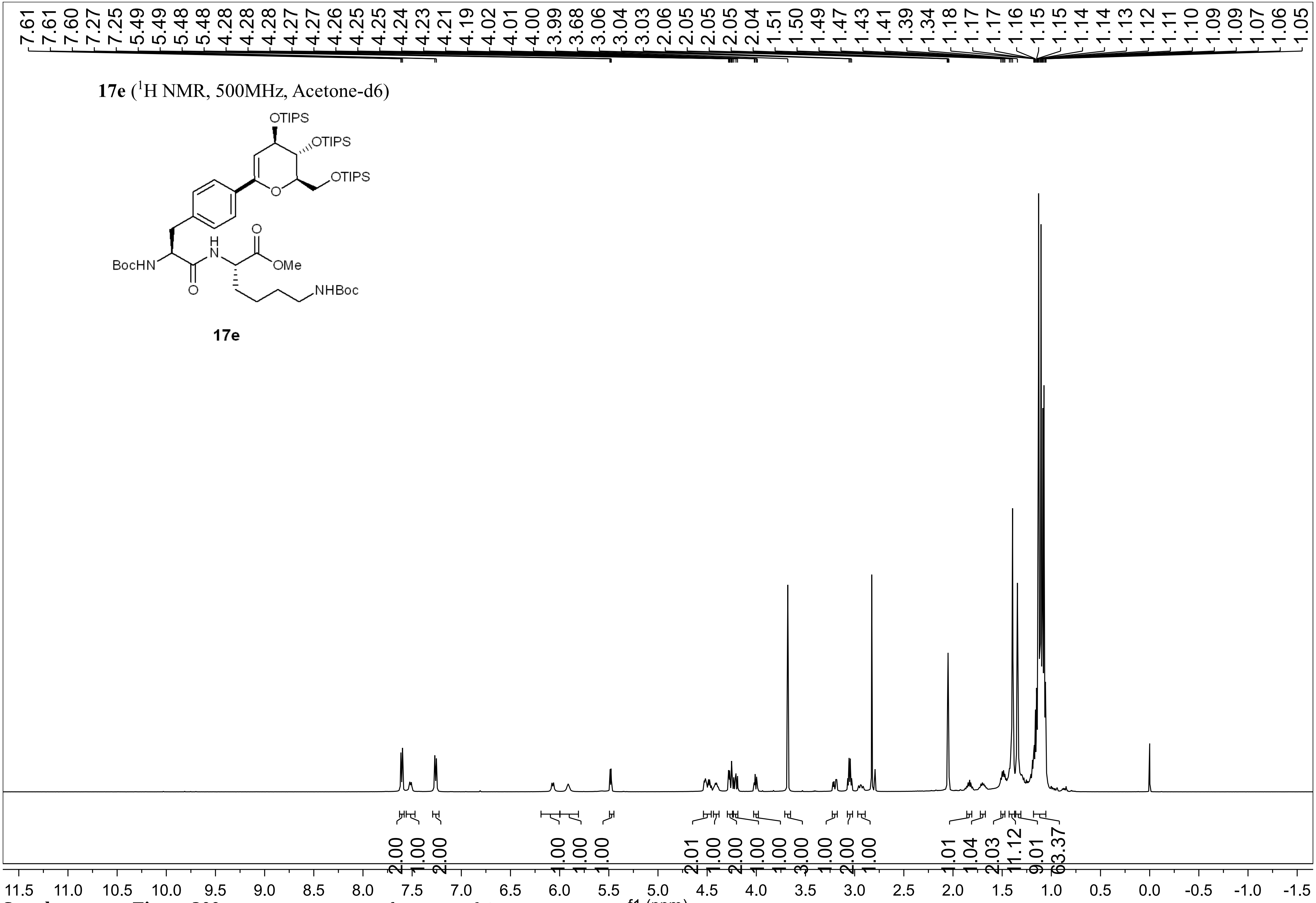

Supplementary Figure 200. ^{1}H NMR spectrum of compound **17e**

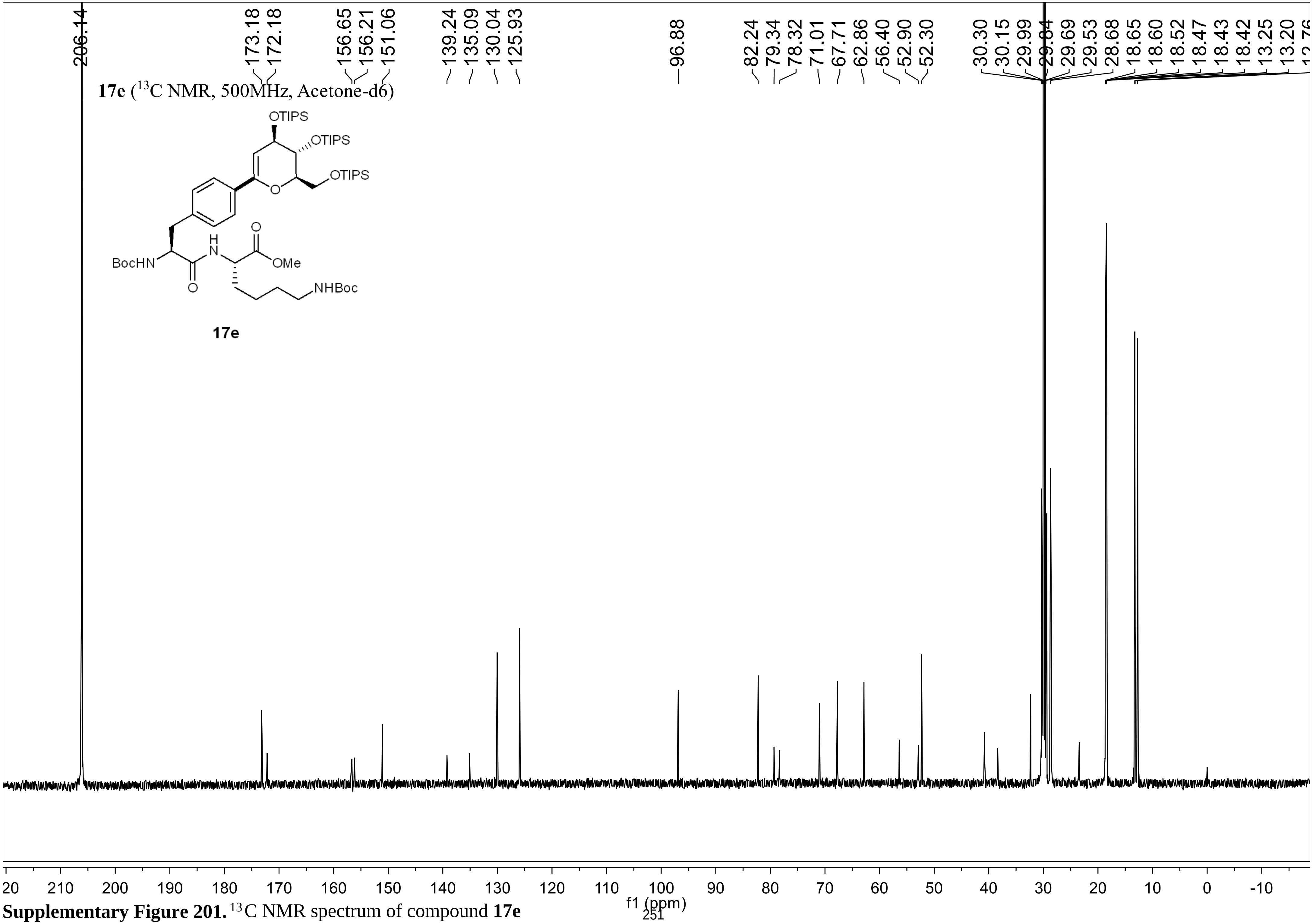

Supplementary Figure 201. ^{13}C NMR spectrum of compound **17e**

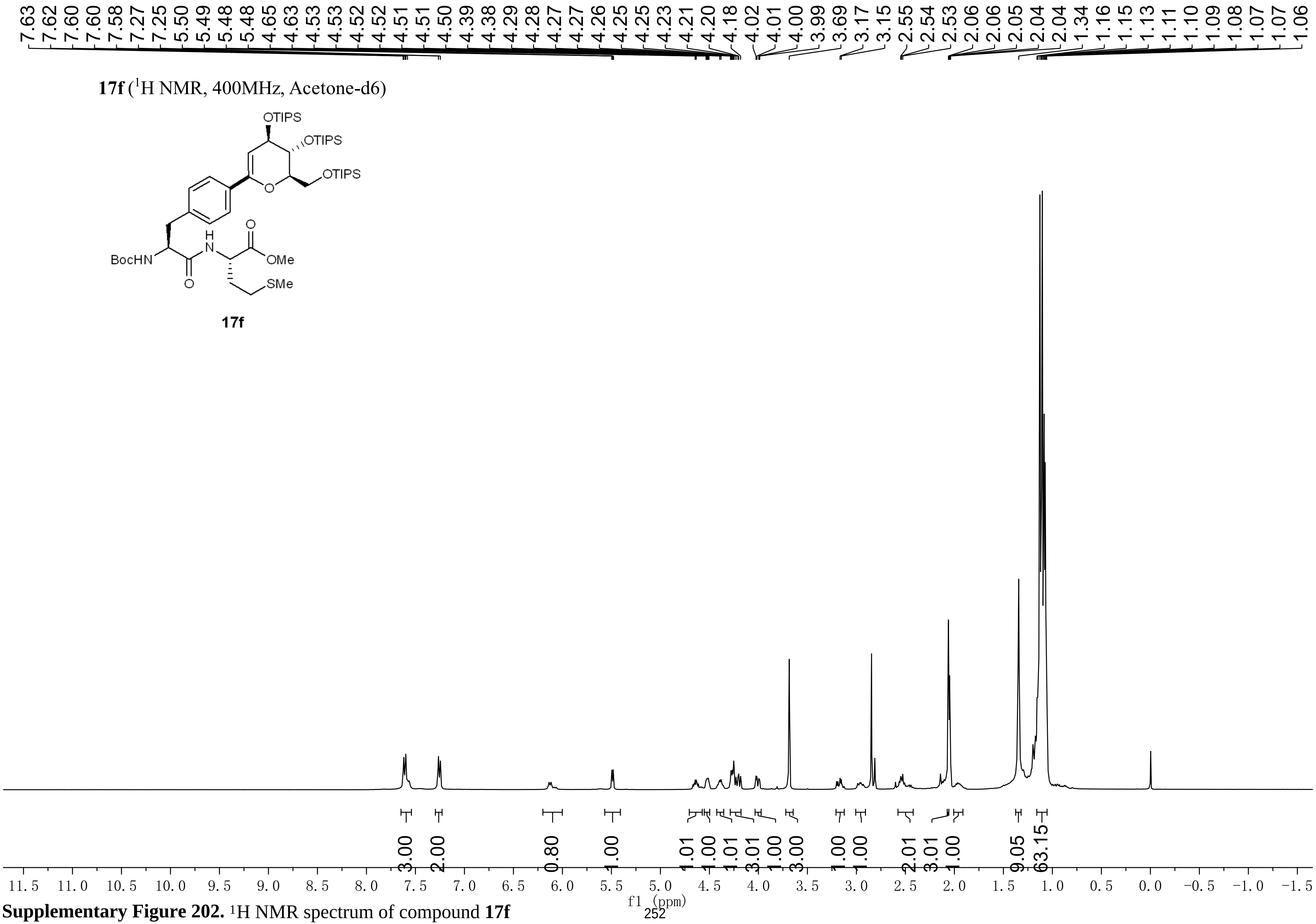

Supplementary Figure 202. ^{1}H NMR spectrum of compound **17f**

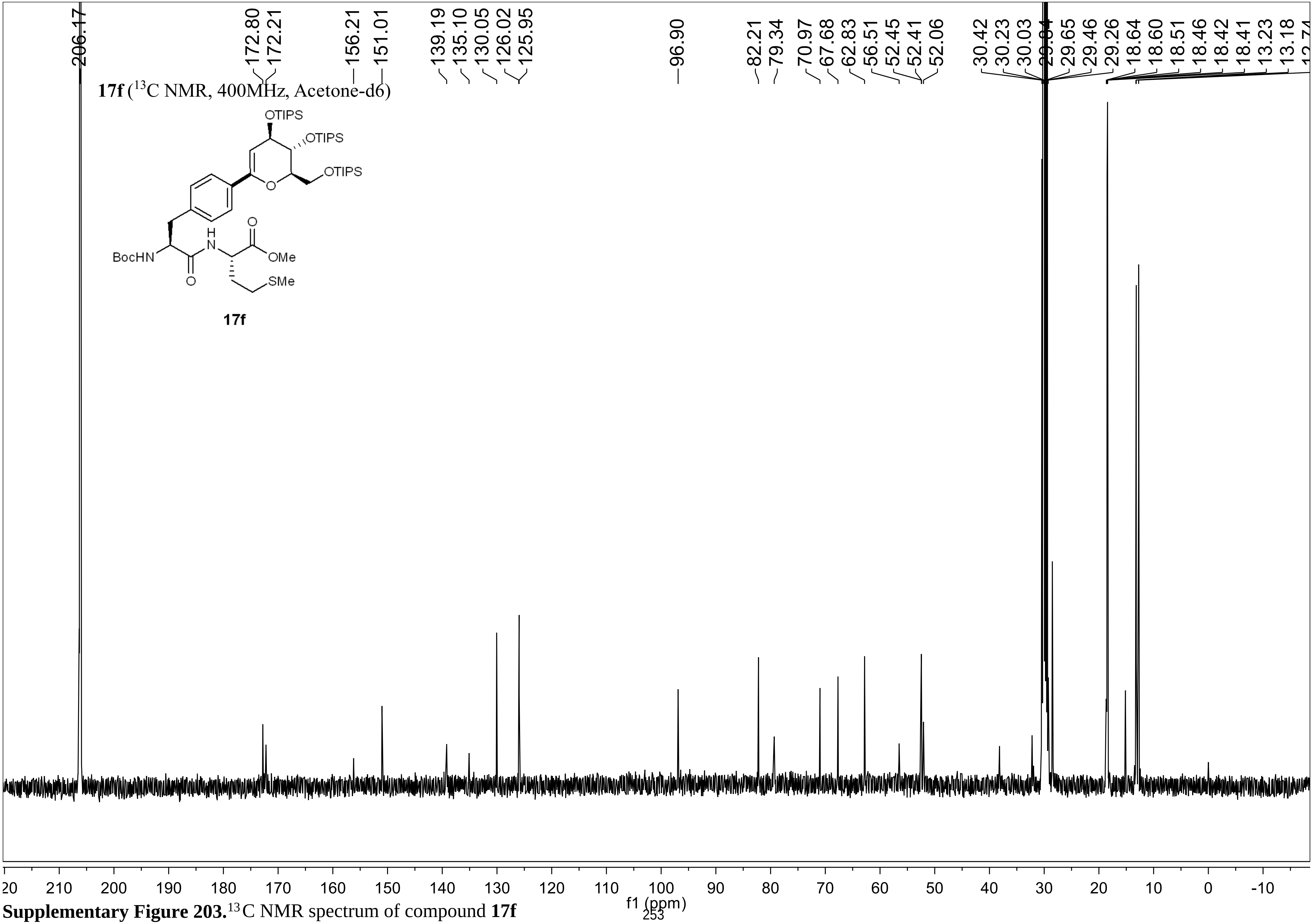

Supplementary Figure 203. ^{13}C NMR spectrum of compound **17f**

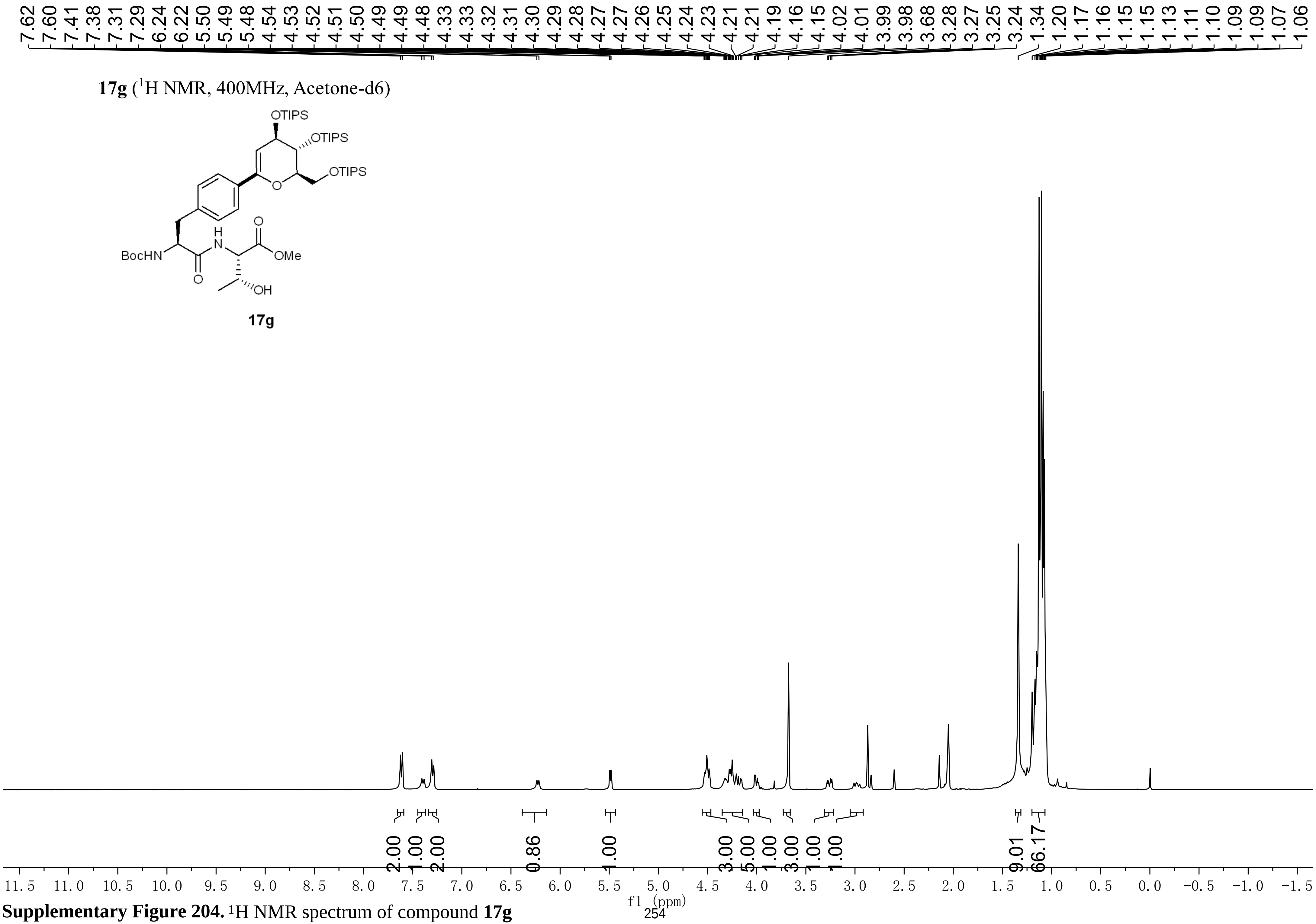

Supplementary Figure 204. ^{1}H NMR spectrum of compound **17g**

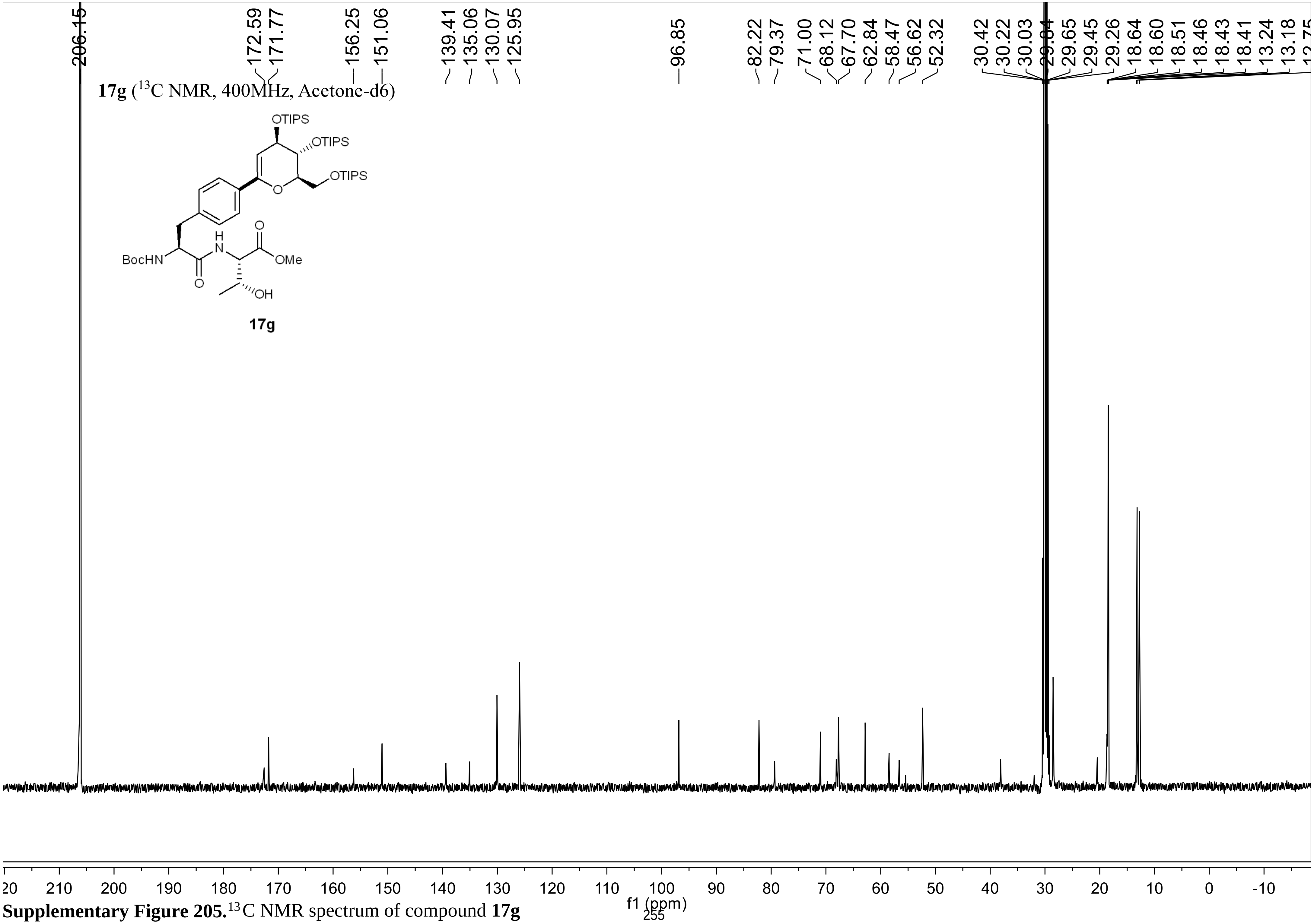

Supplementary Figure 205. ^{13}C NMR spectrum of compound **17g**

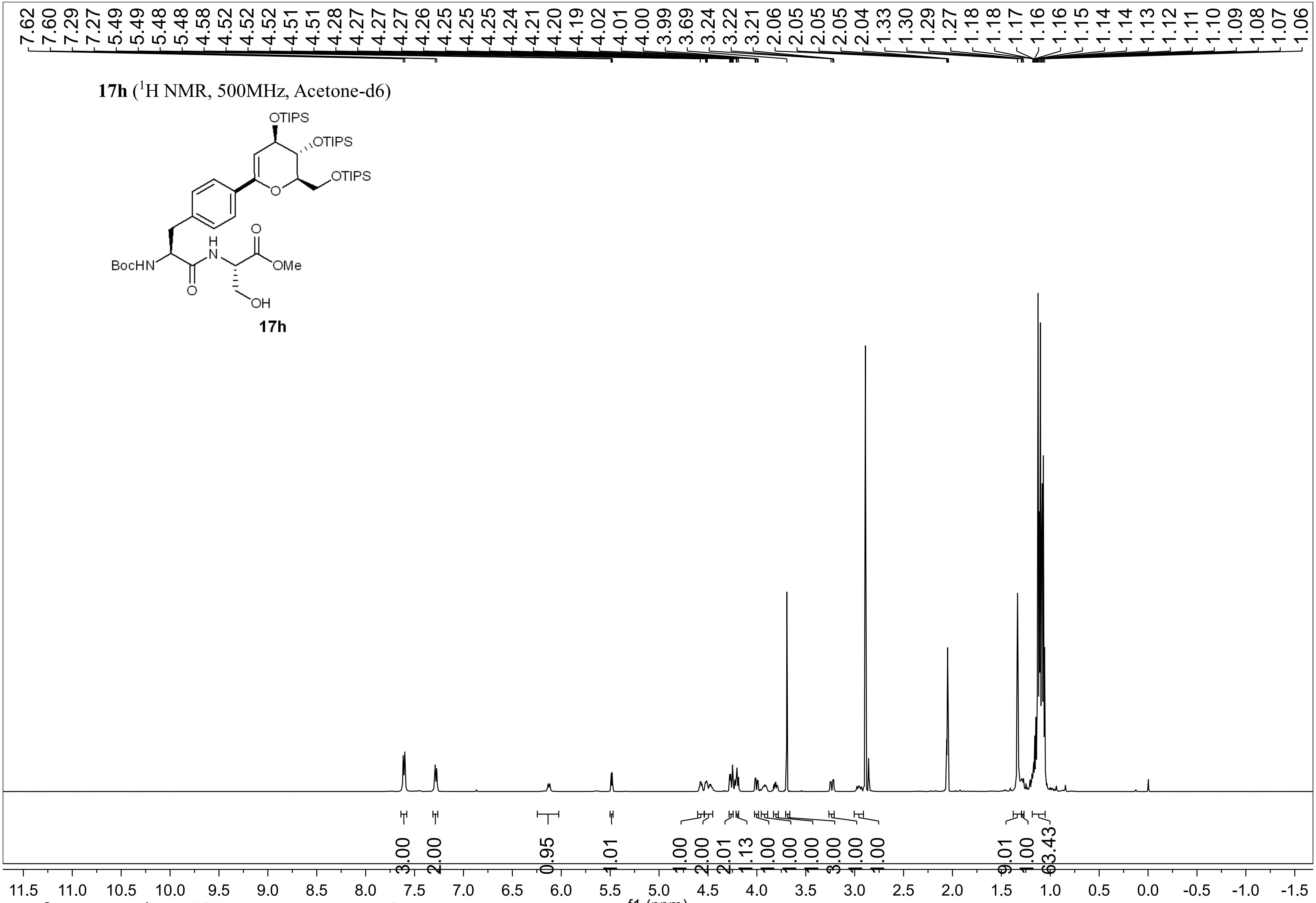

Supplementary Figure 206. ^{1}H NMR spectrum of compound **17h**

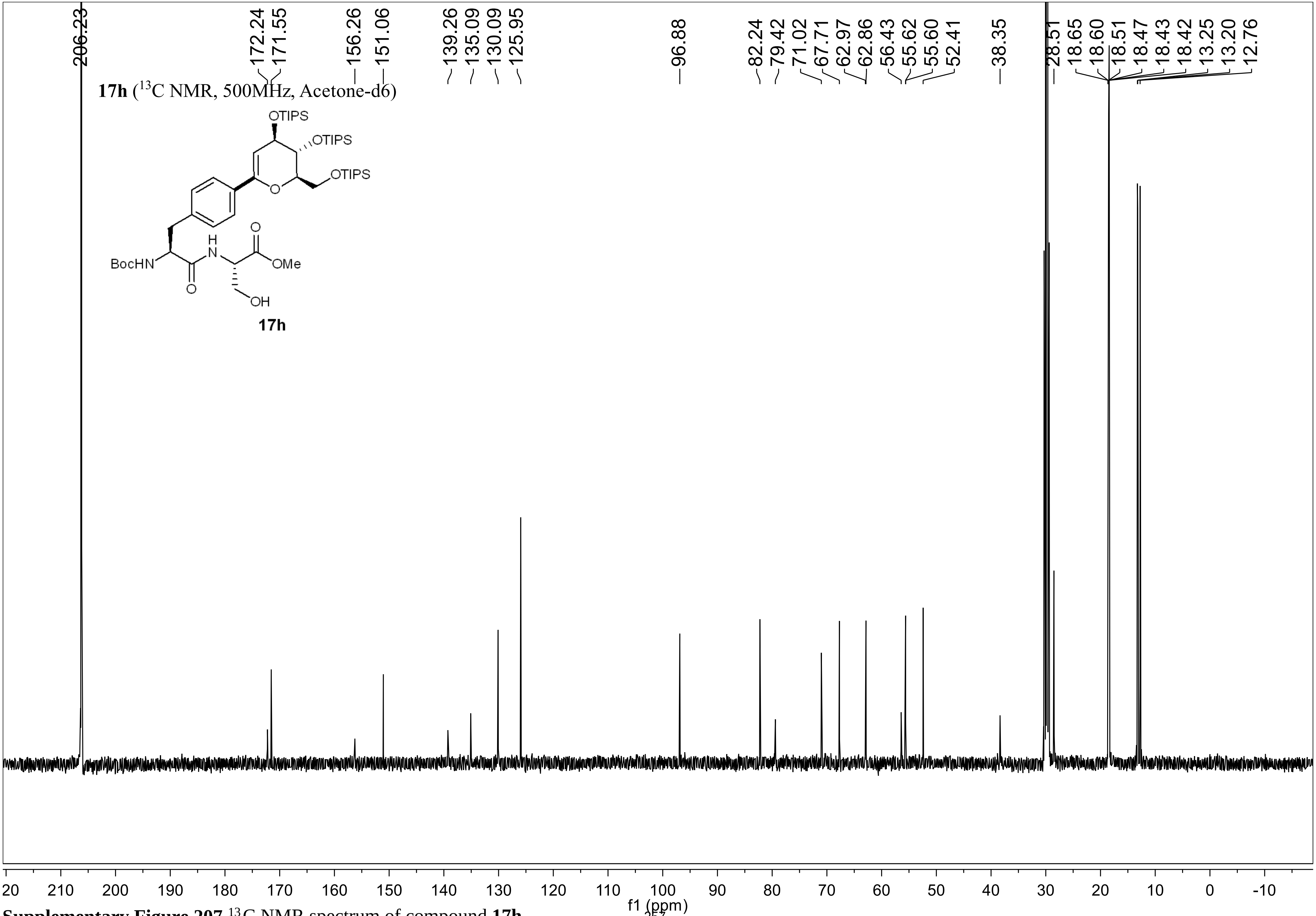

Supplementary Figure 207. ^{13}C NMR spectrum of compound **17h**

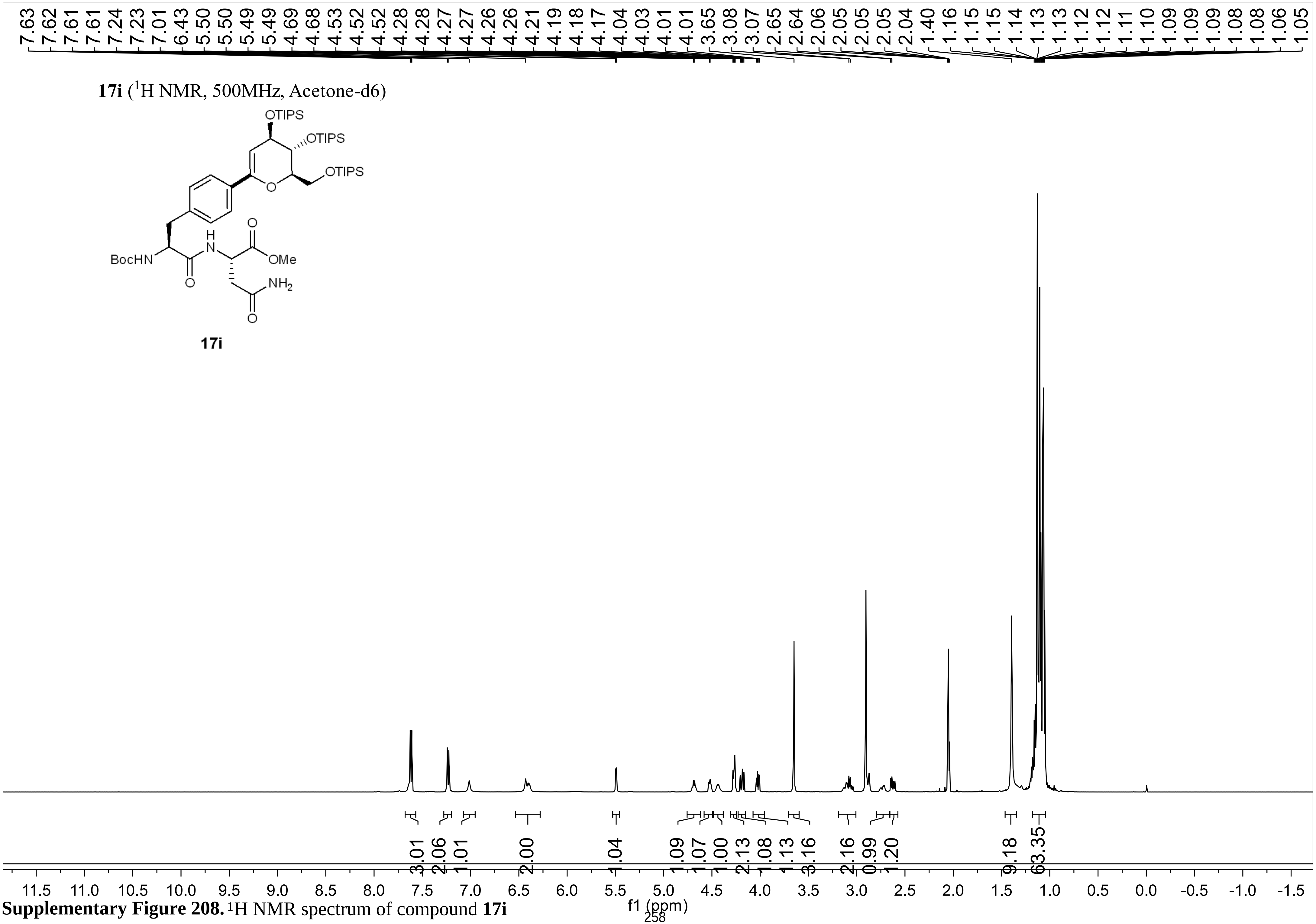

Supplementary Figure 208. ^{1}H NMR spectrum of compound **17i**

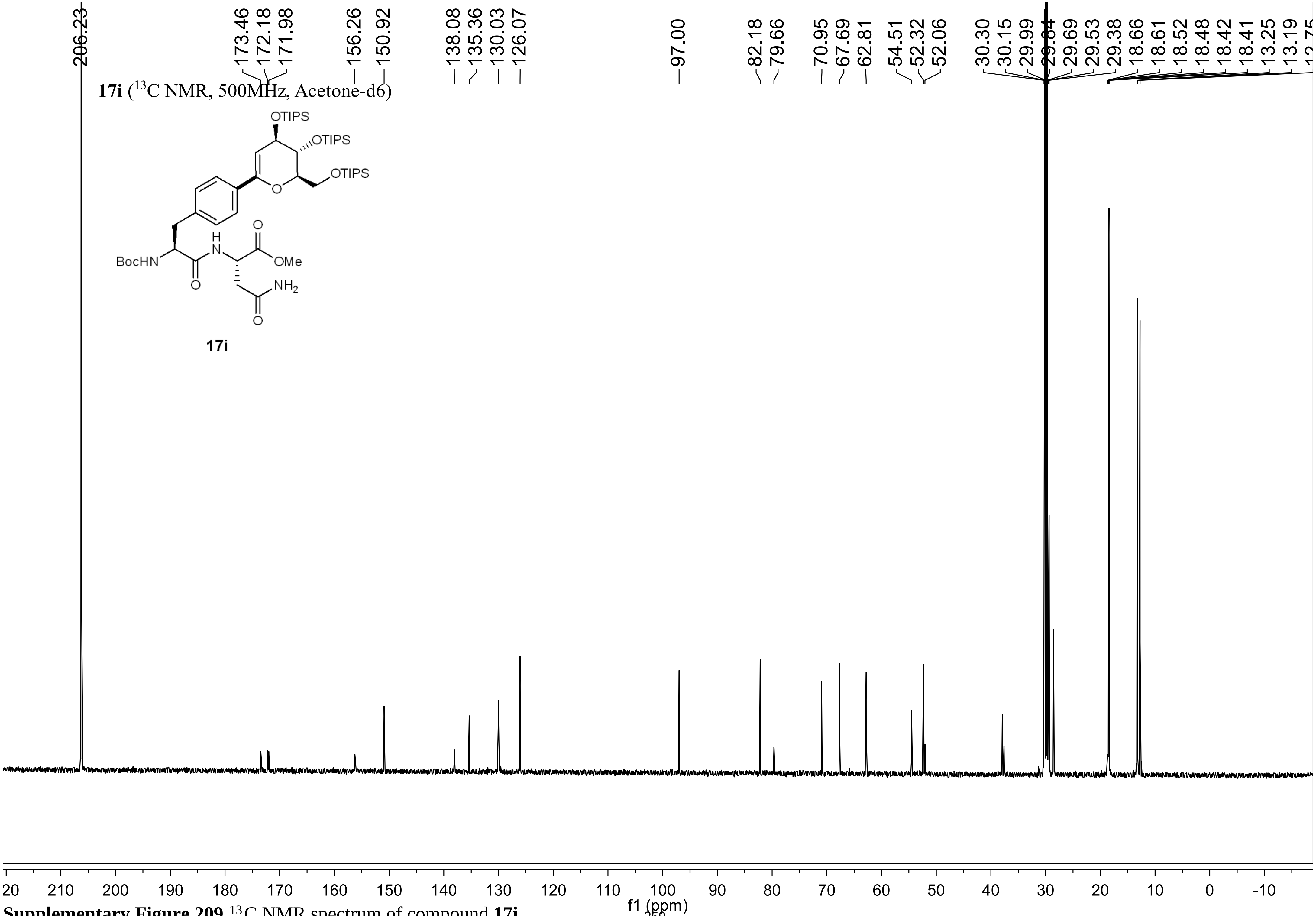

Supplementary Figure 209. ^{13}C NMR spectrum of compound **17i**

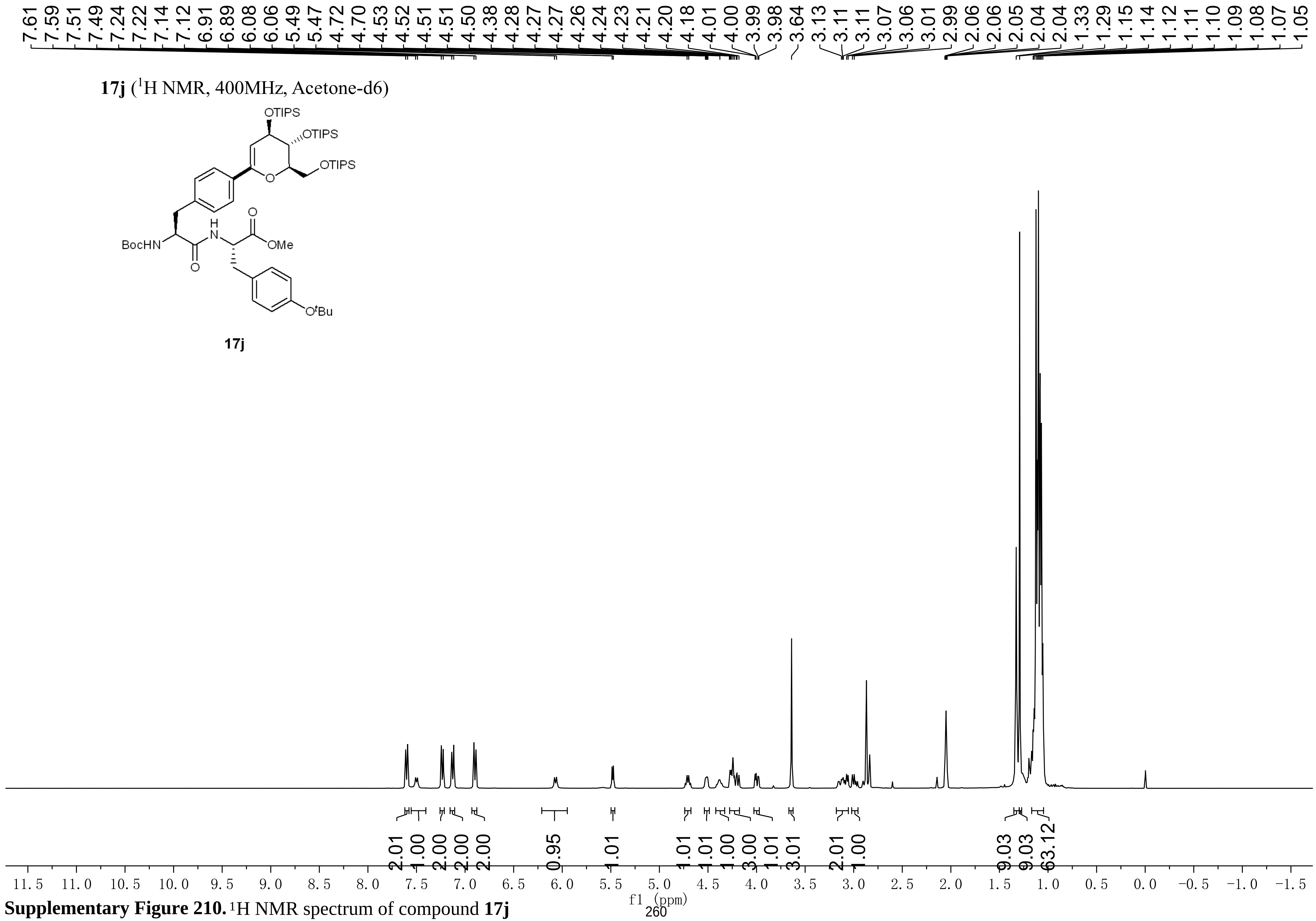

Supplementary Figure 210. ^{1}H NMR spectrum of compound **17j**

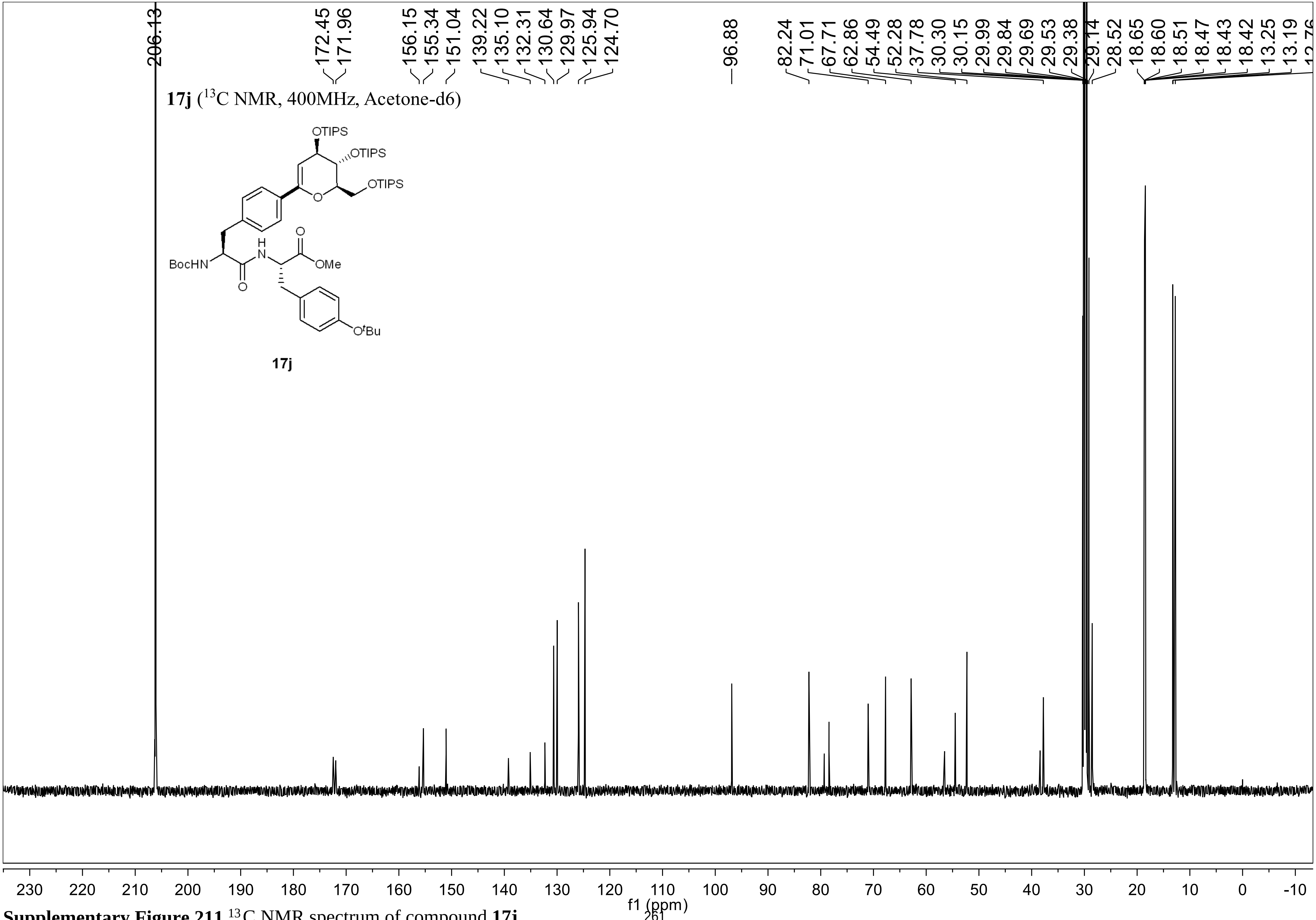

Supplementary Figure 211. ^{13}C NMR spectrum of compound **17j**

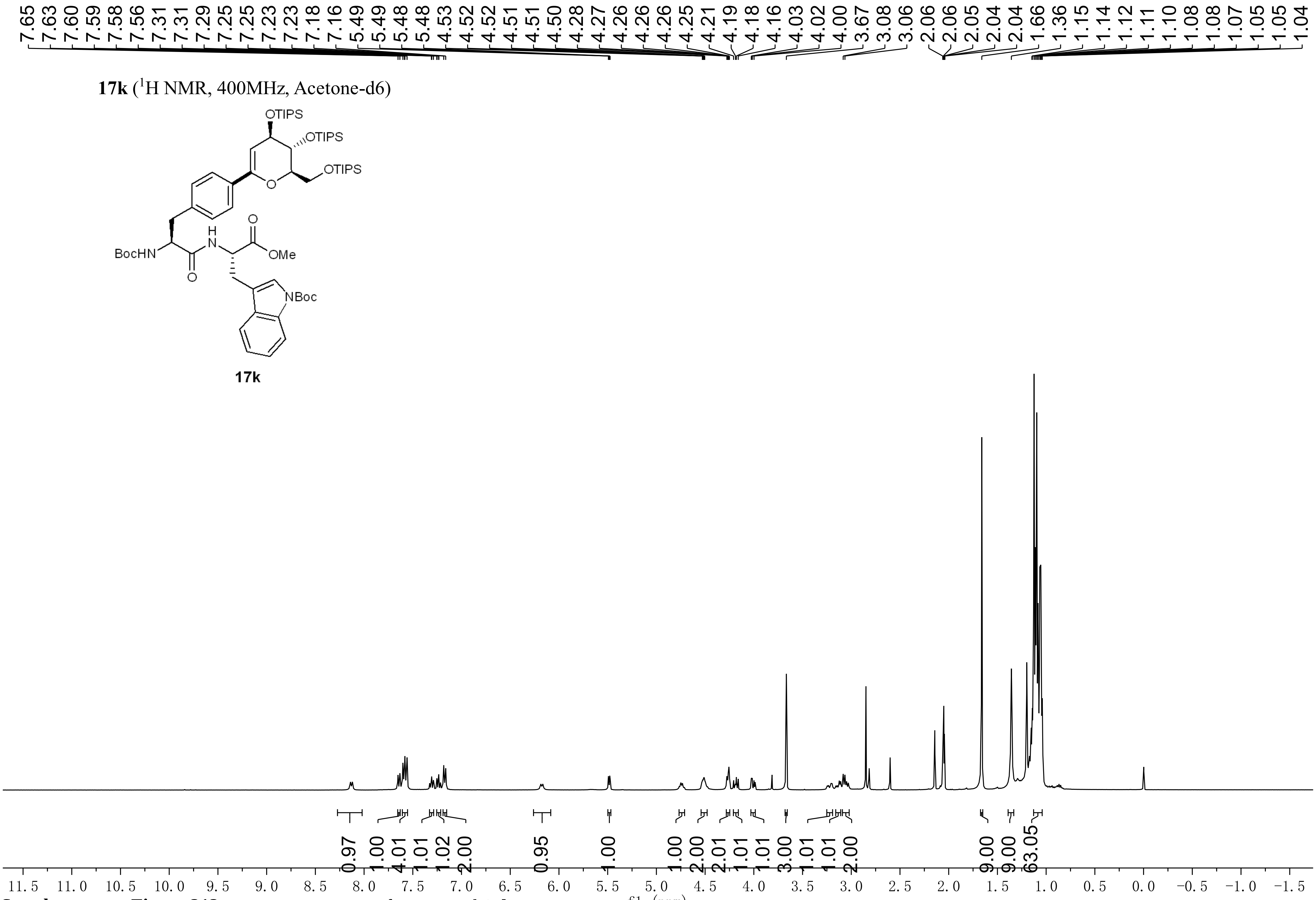

Supplementary Figure 212. ^{1}H NMR spectrum of compound **17k**

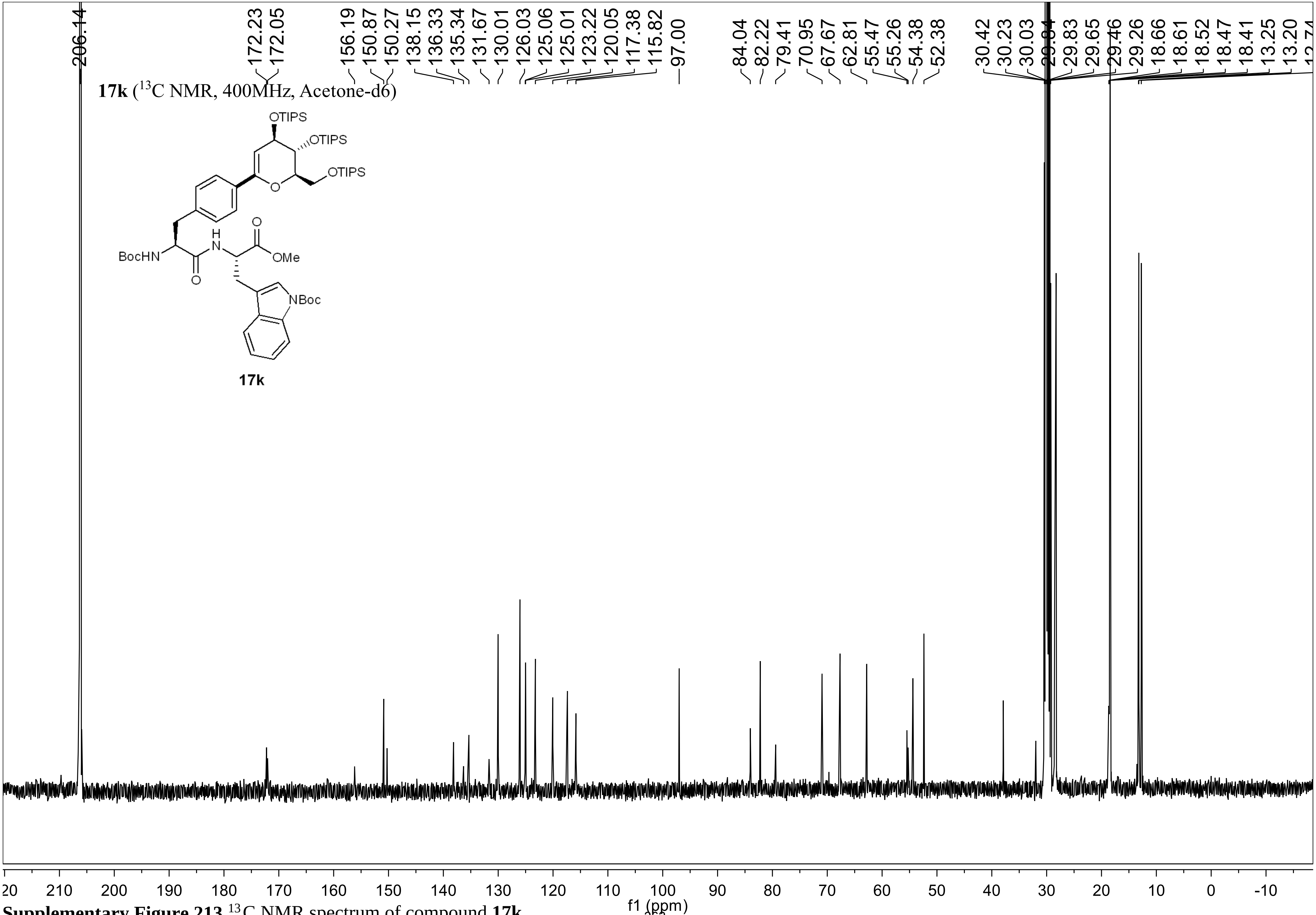

Supplementary Figure 213. ^{13}C NMR spectrum of compound **17k**

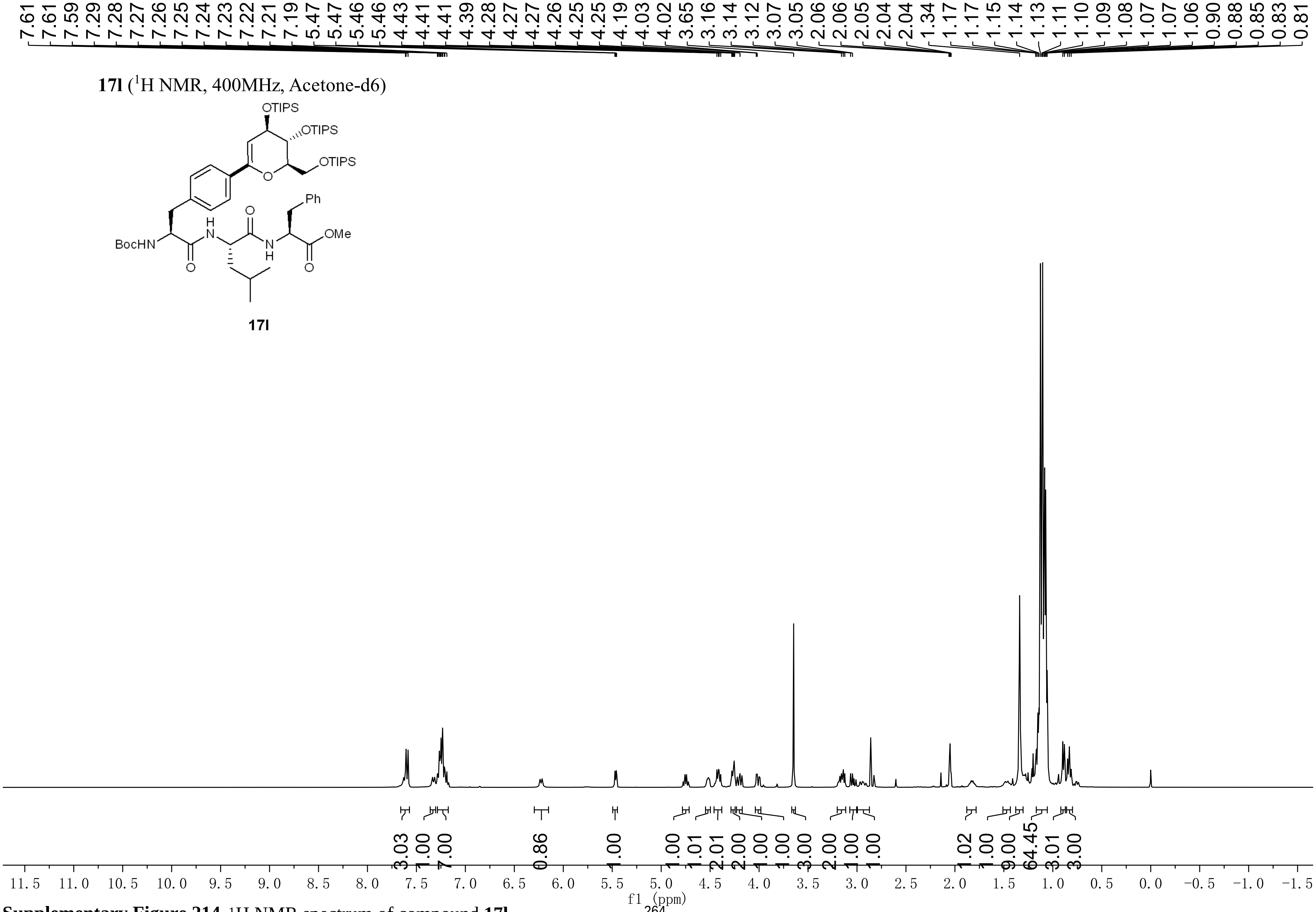

Supplementary Figure 214. ^{1}H NMR spectrum of compound **17l**

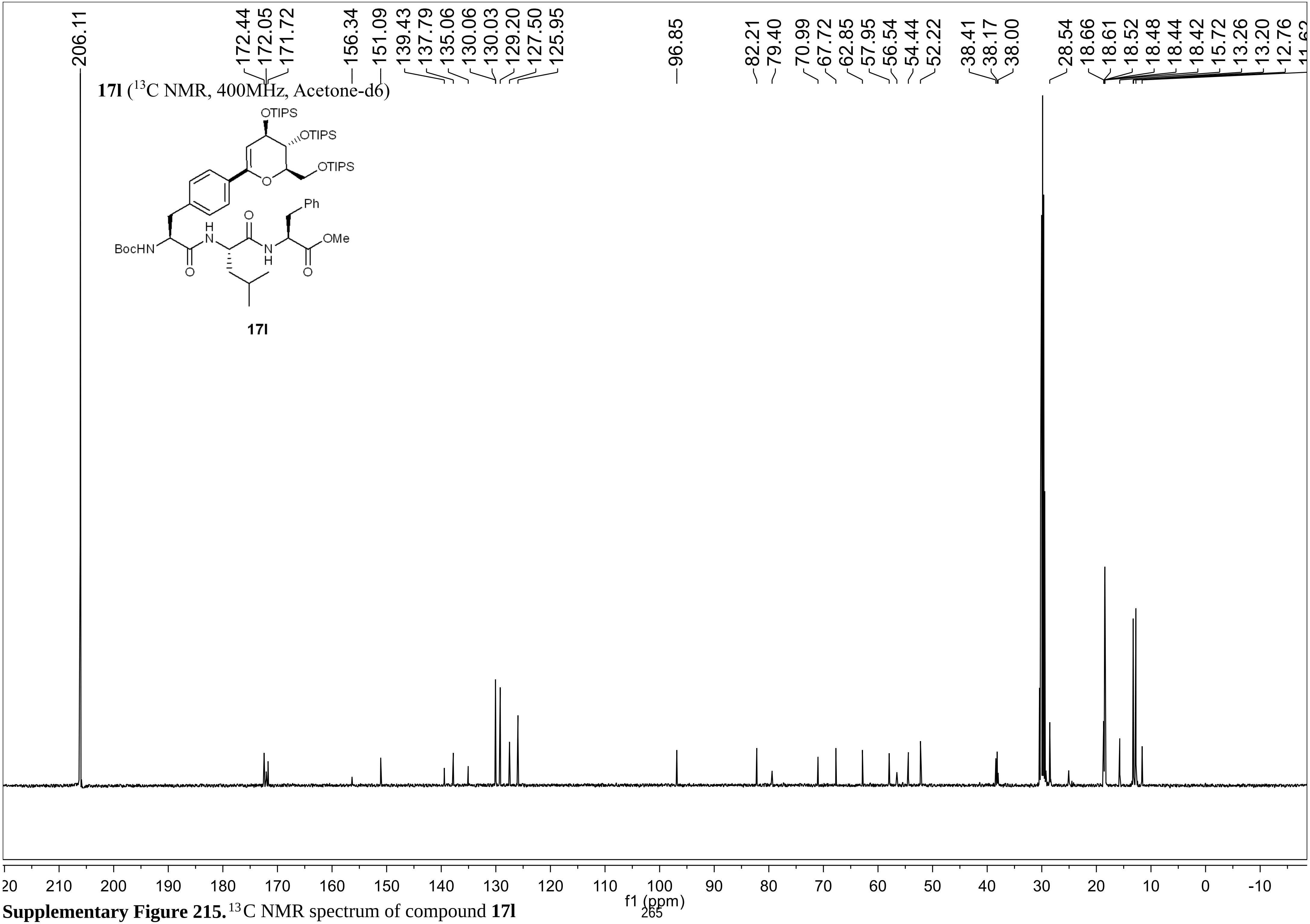

Supplementary Figure 215. ^{13}C NMR spectrum of compound **17l**

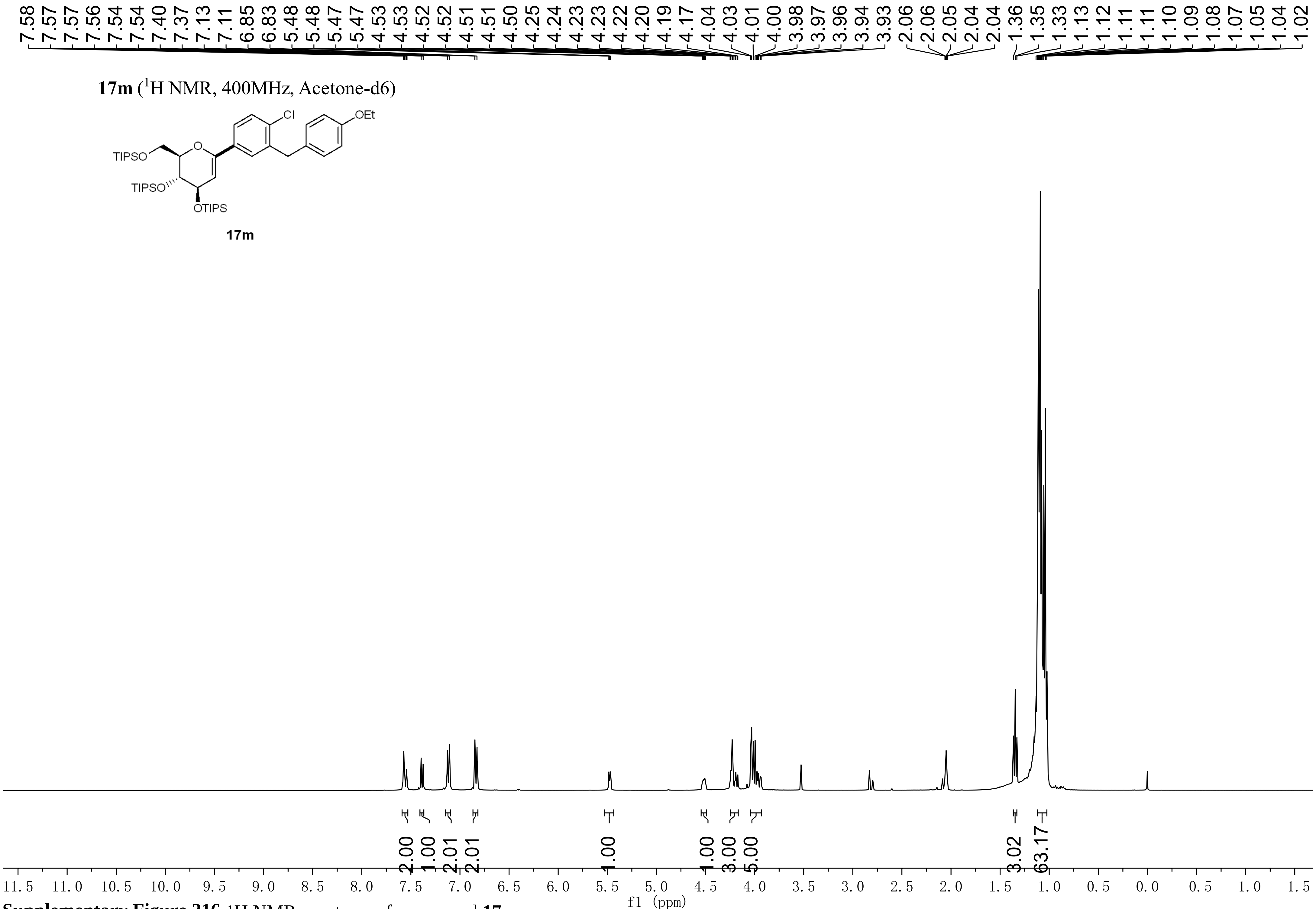

Supplementary Figure 216. ^{1}H NMR spectrum of compound **17m**

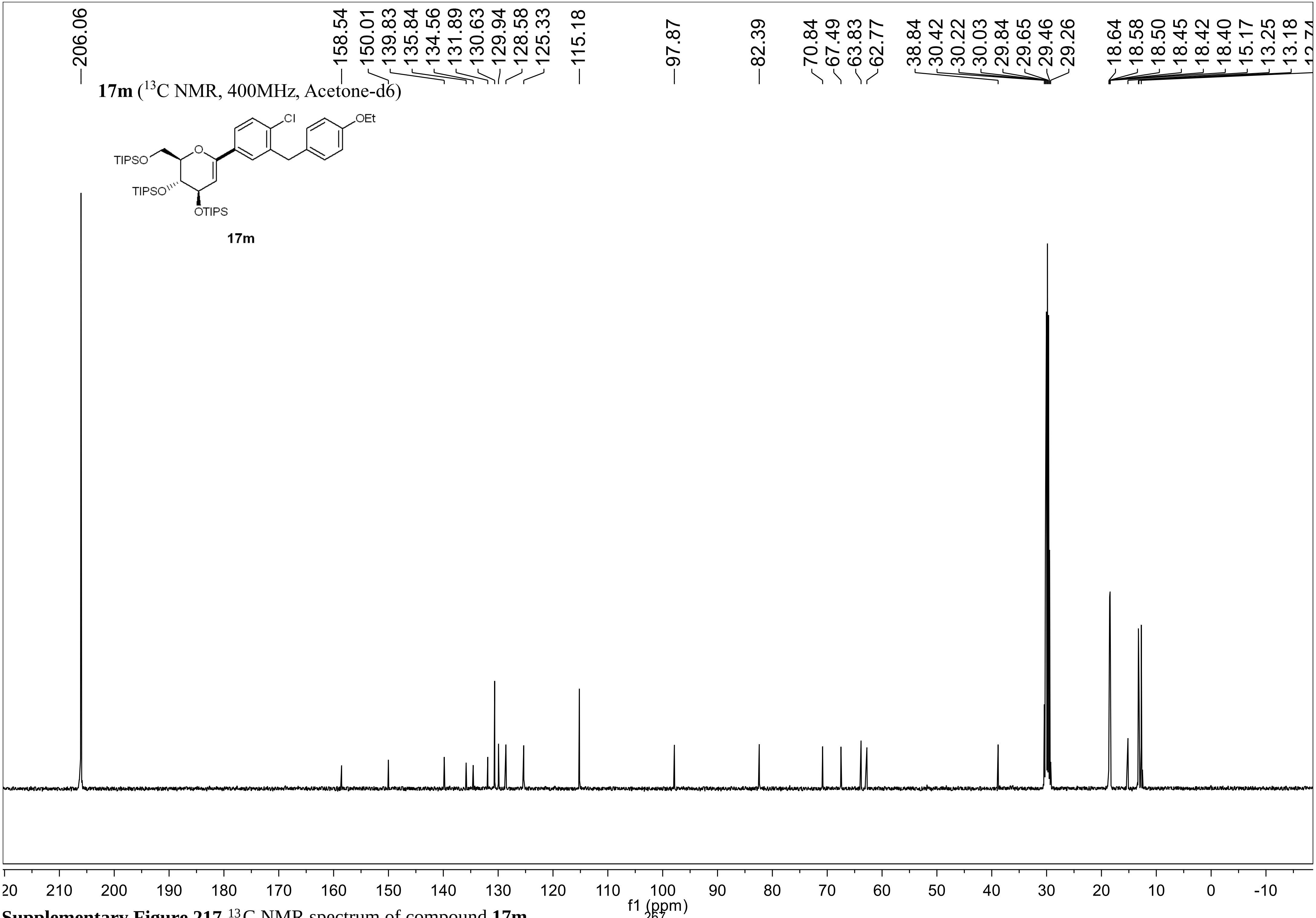

Supplementary Figure 217. ^{13}C NMR spectrum of compound **17m**

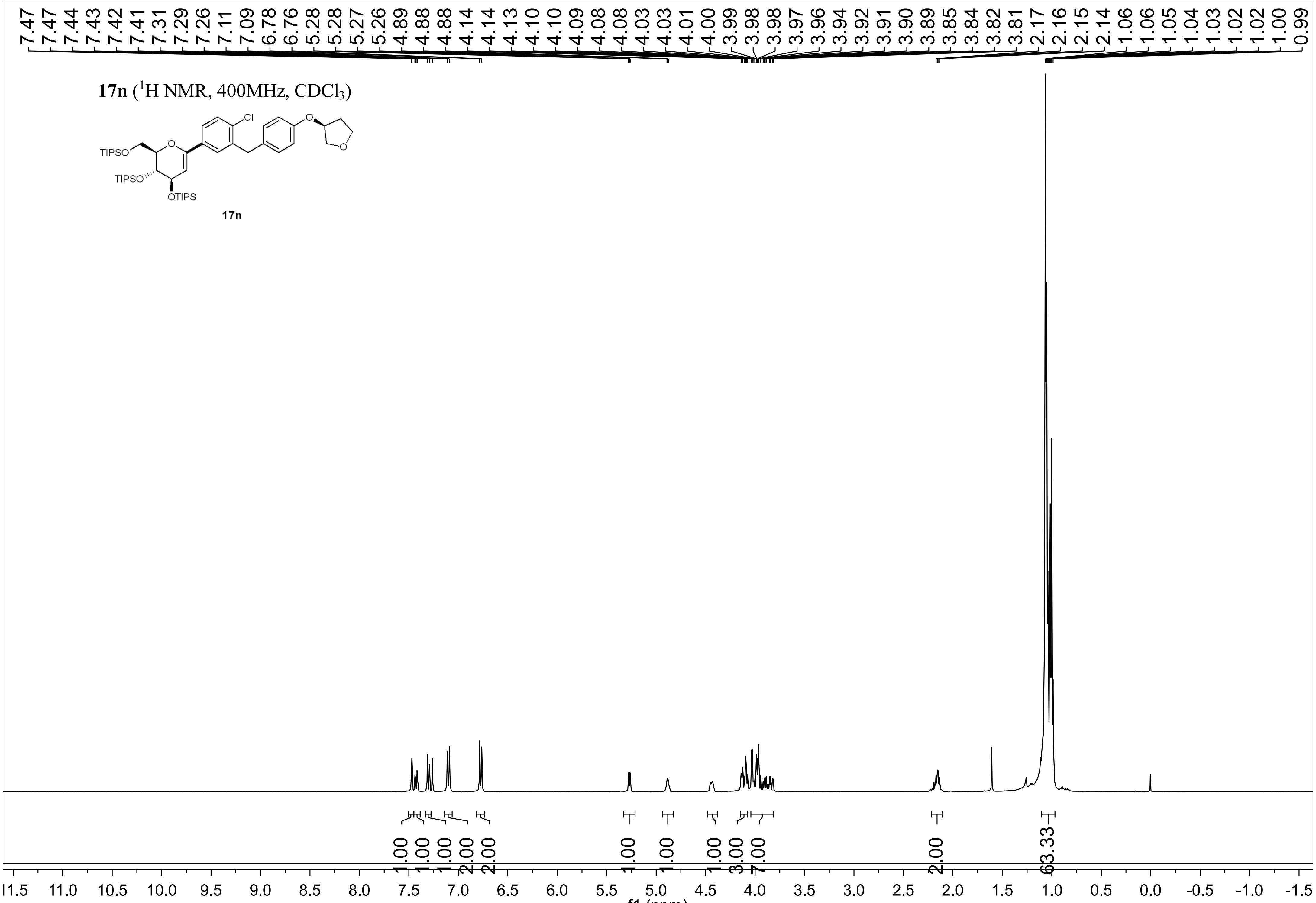

Supplementary Figure 218. ^{1}H NMR spectrum of compound **17n**

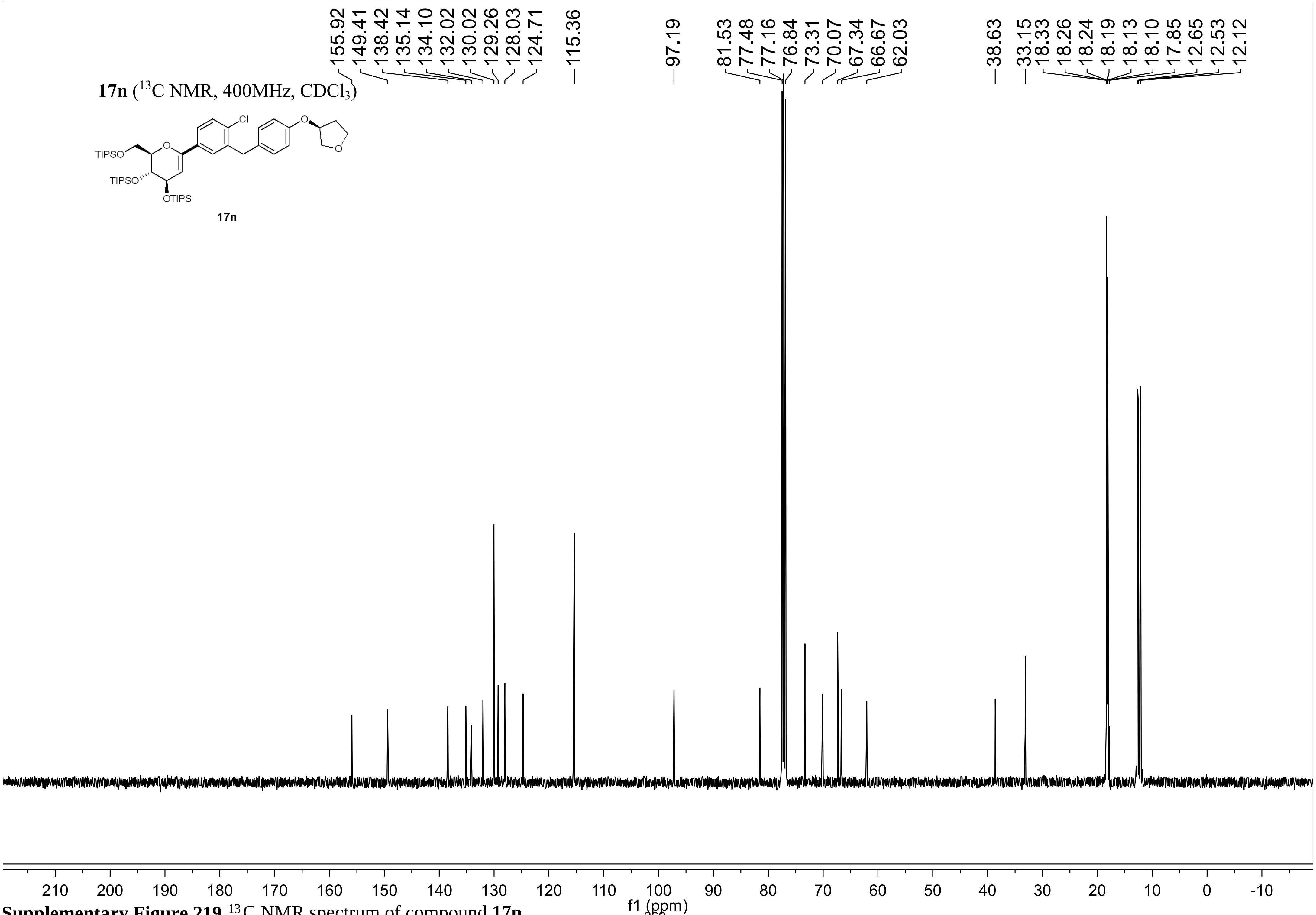

Supplementary Figure 219. ^{13}C NMR spectrum of compound **17n**

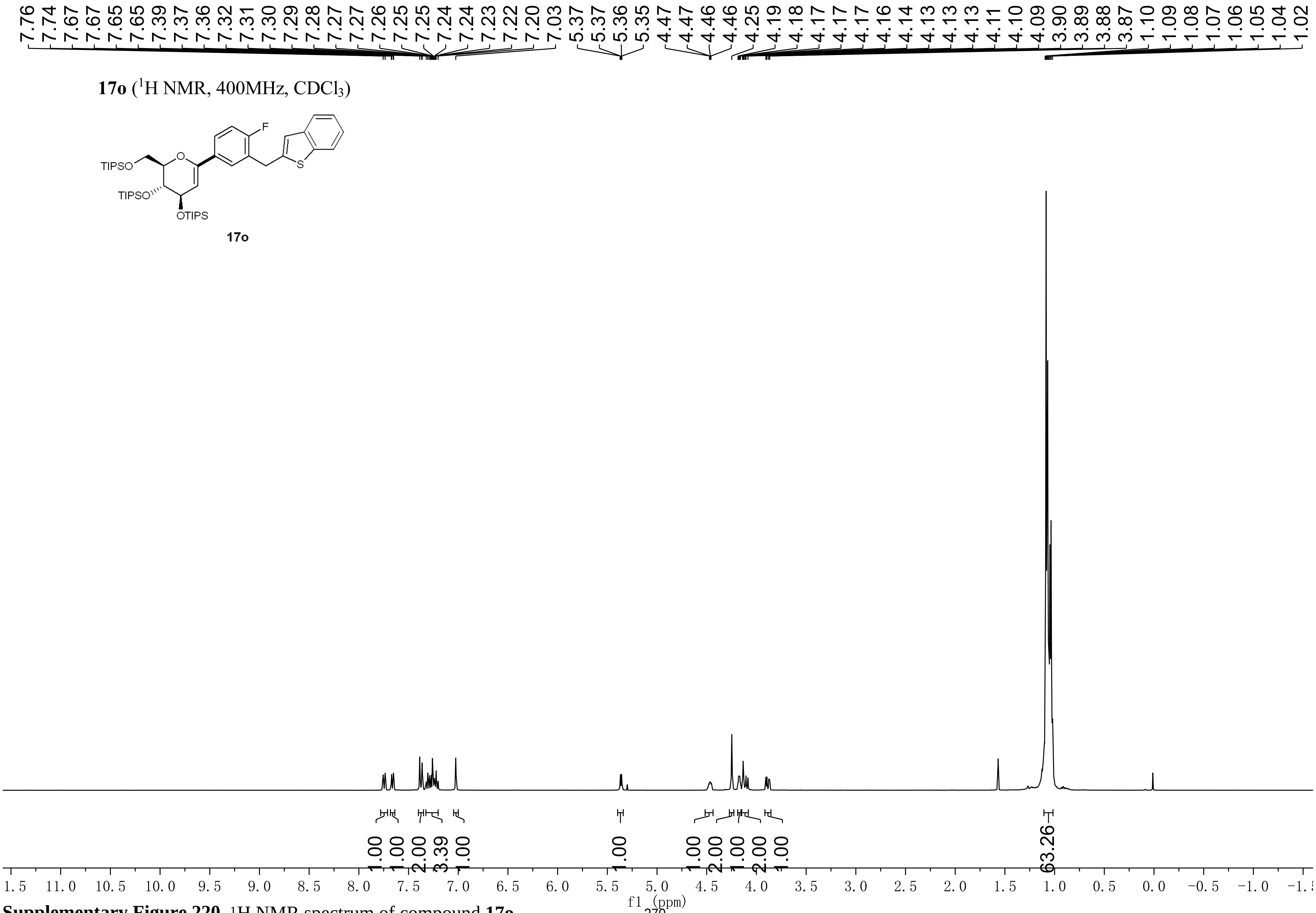

Supplementary Figure 220. ^{1}H NMR spectrum of compound **17o**

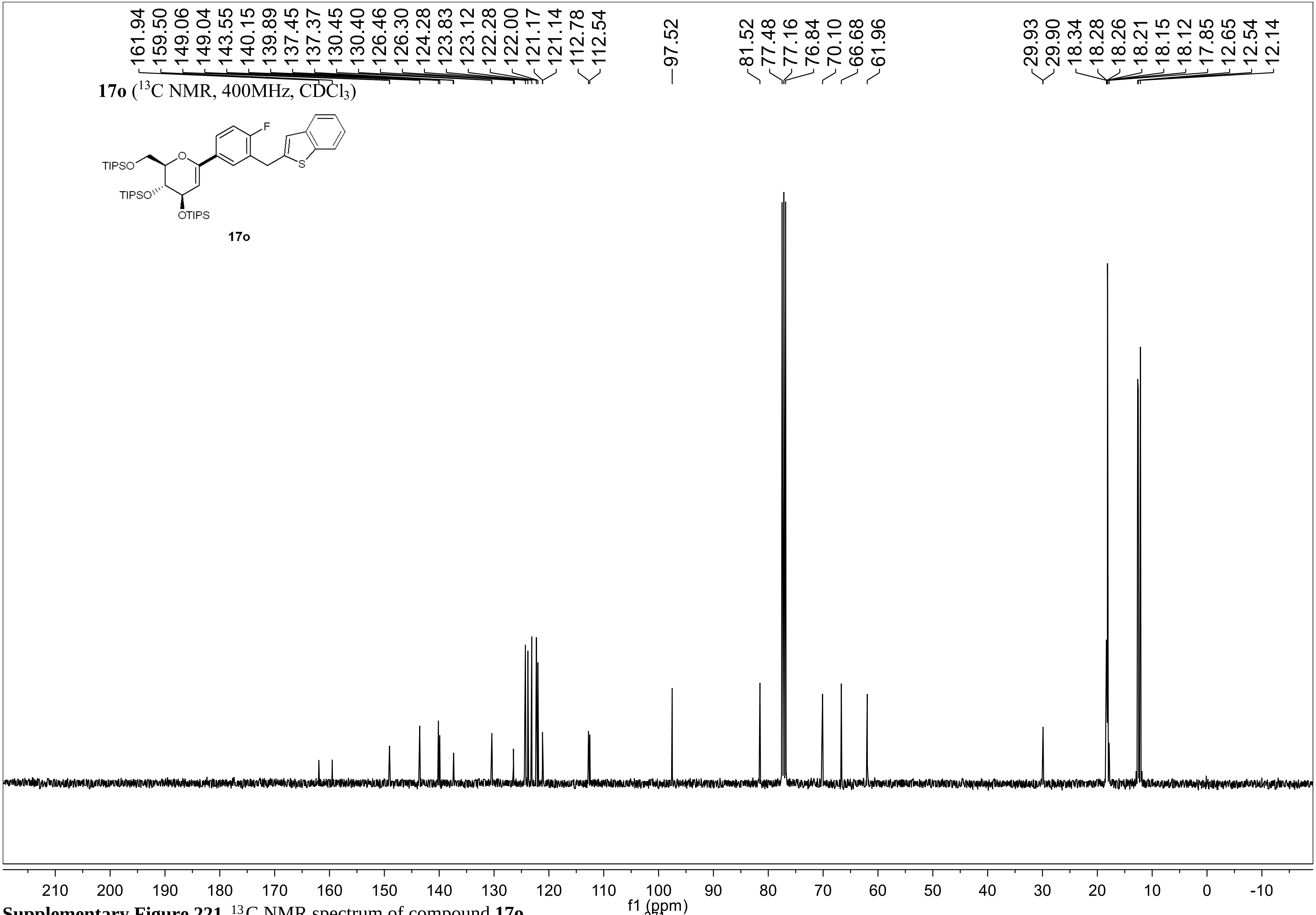

Supplementary Figure 221. ^{13}C NMR spectrum of compound **17o**

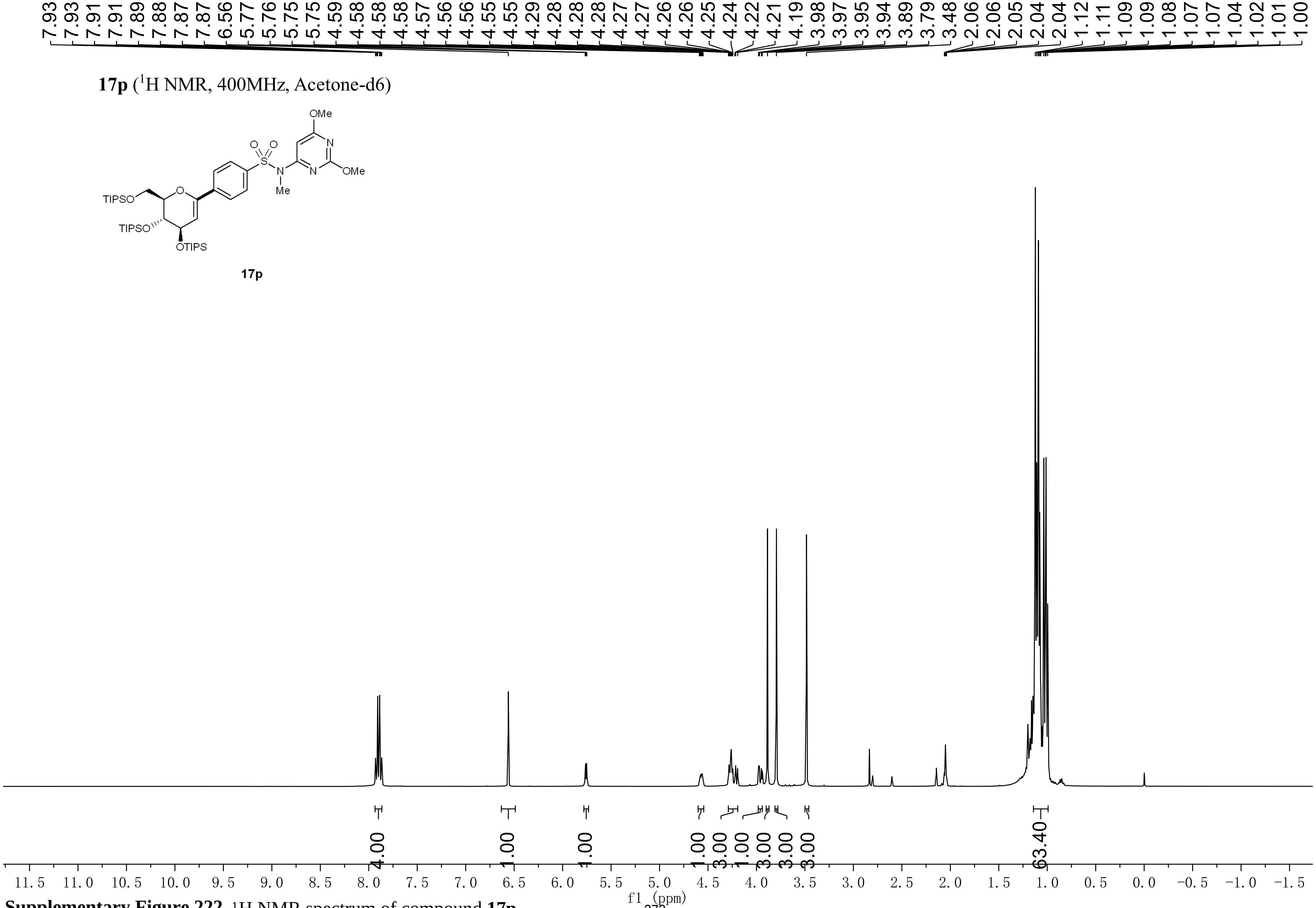

Supplementary Figure 222. ^{1}H NMR spectrum of compound **17p**

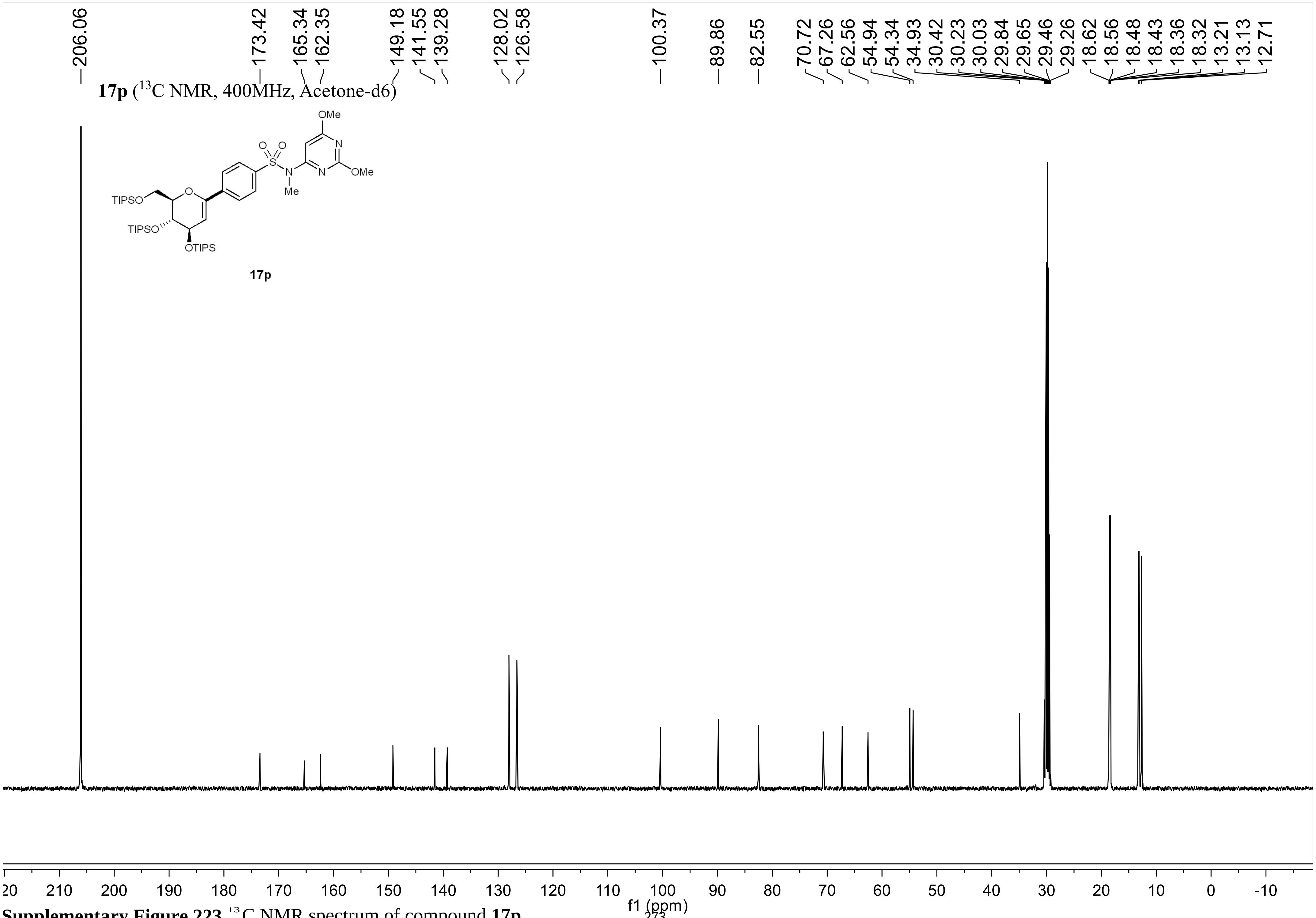

Supplementary Figure 223. ^{13}C NMR spectrum of compound **17p**

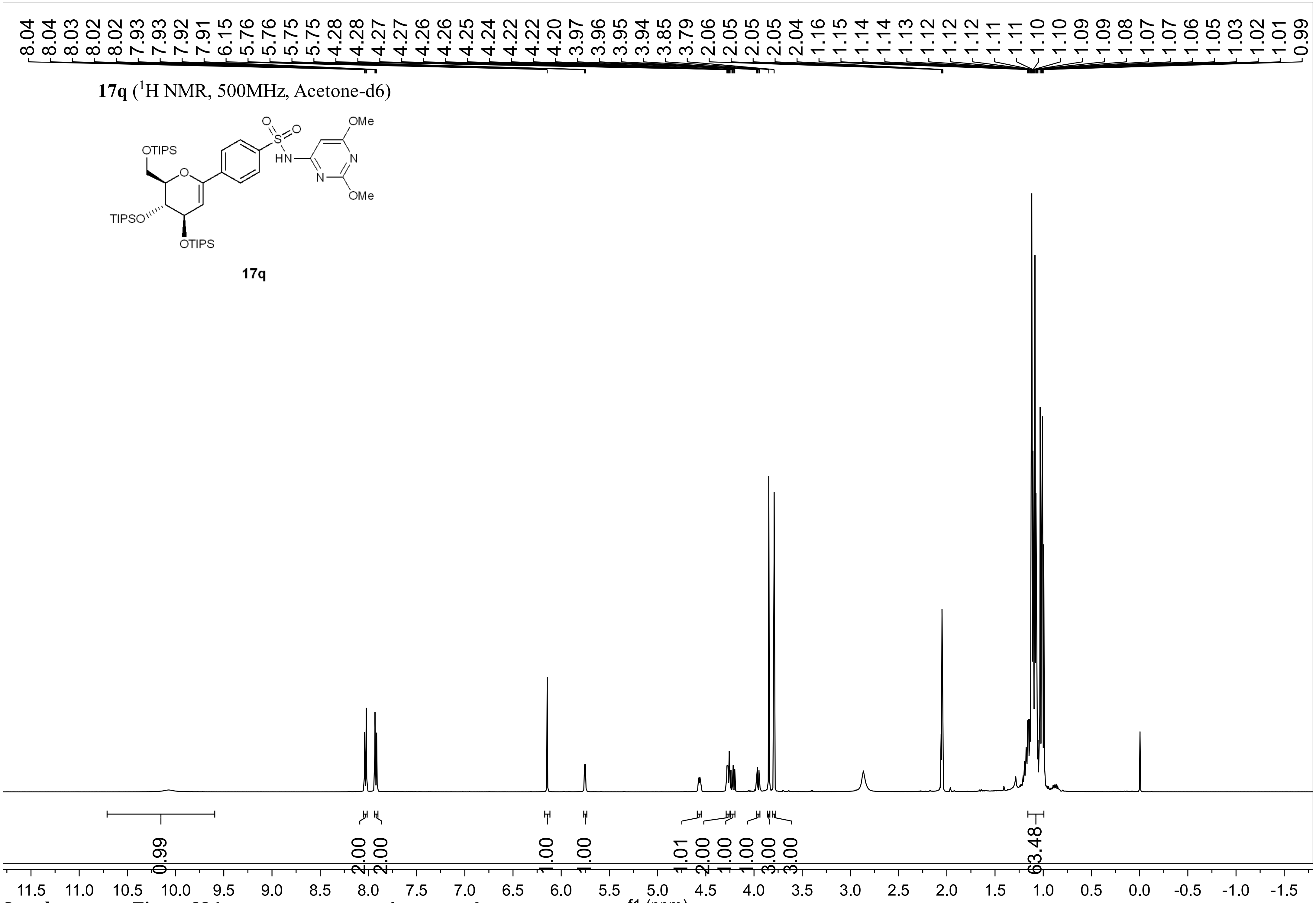

Supplementary Figure 224. ^{1}H NMR spectrum of compound **17q**

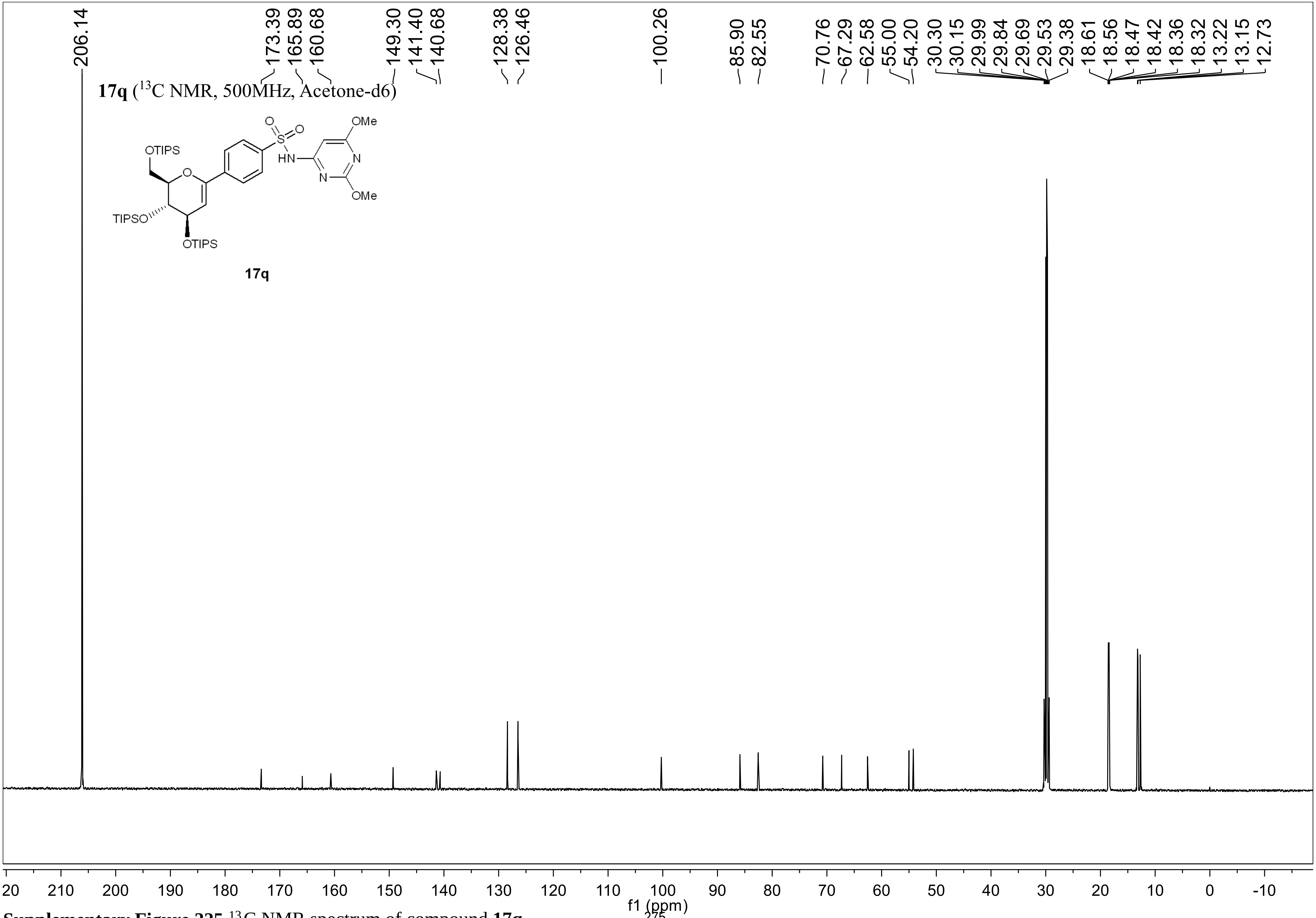

Supplementary Figure 225. ^{13}C NMR spectrum of compound **17q**

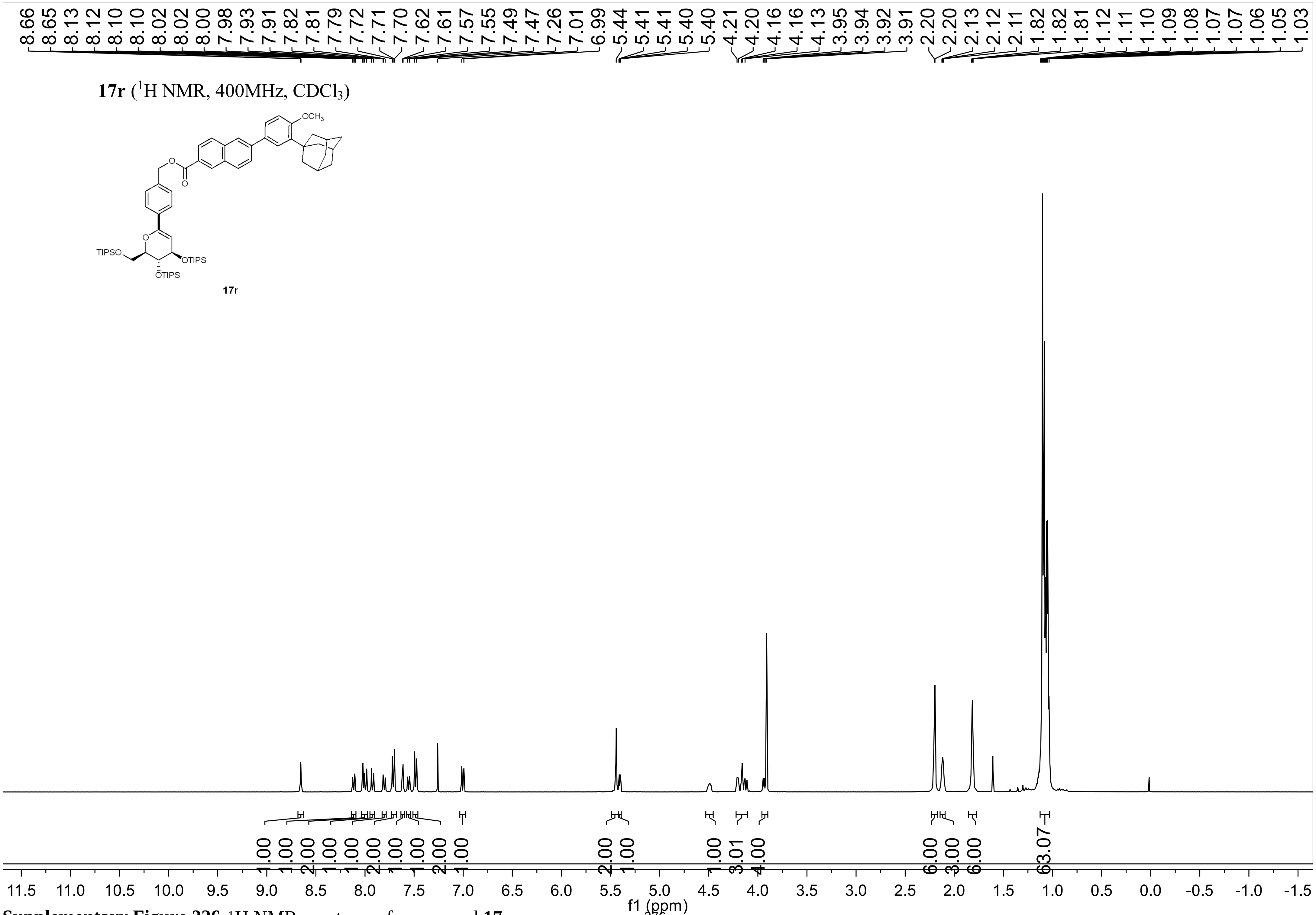

Supplementary Figure 226. 1H NMR spectrum of compound **17r**

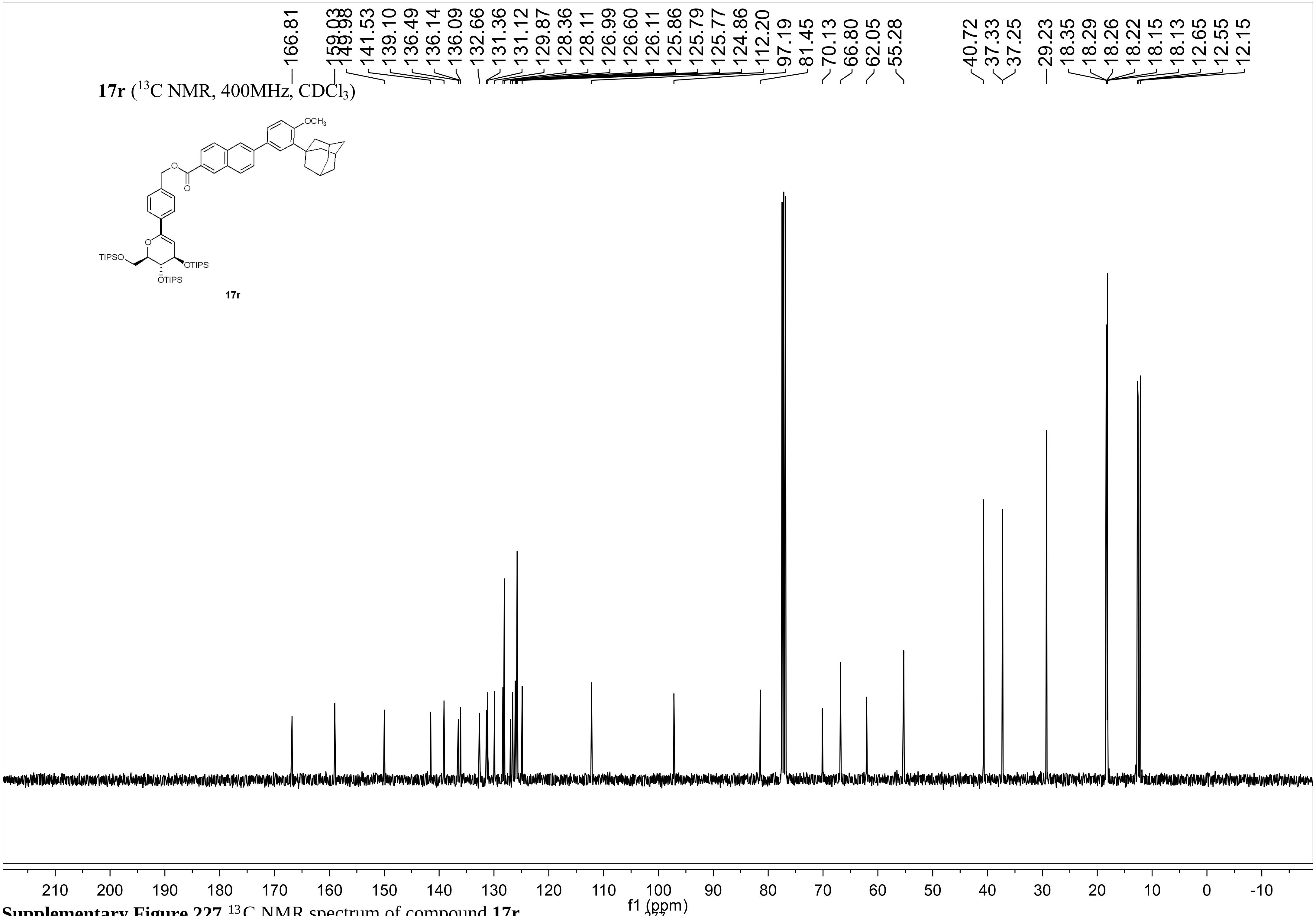

Supplementary Figure 227. ^{13}C NMR spectrum of compound **17r**

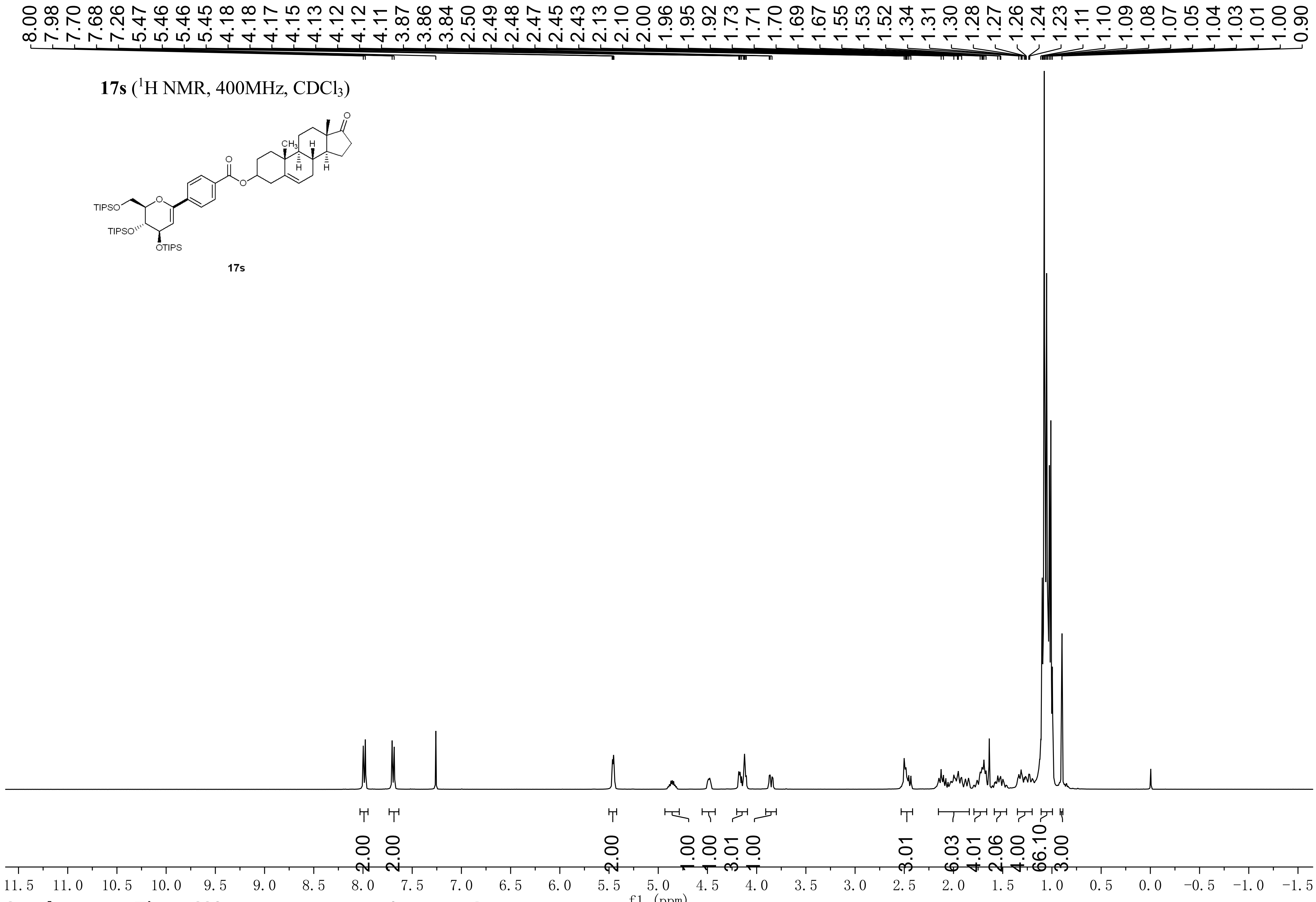

Supplementary Figure 228. ^1H NMR spectrum of compound **17s**

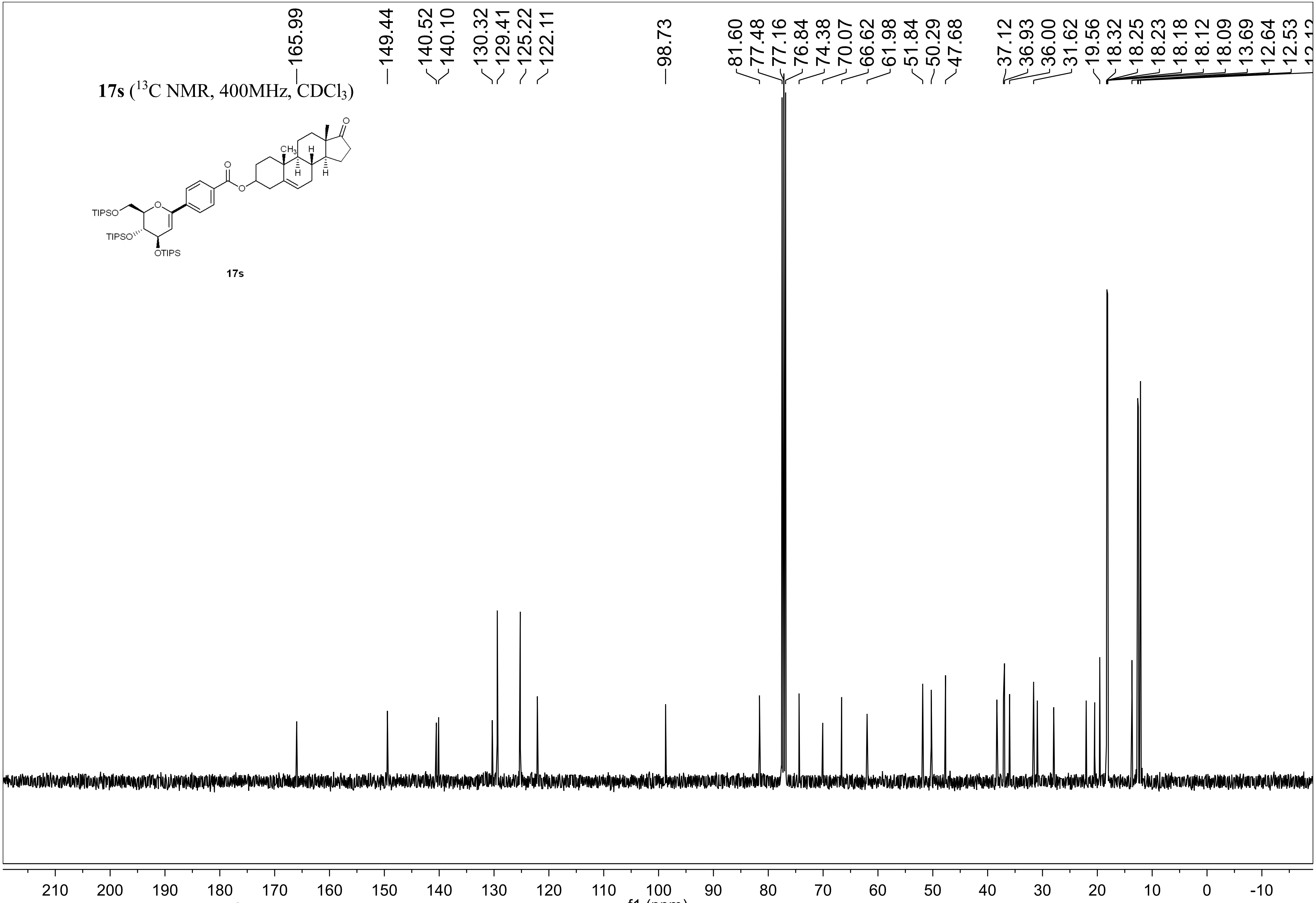

Supplementary Figure 229. ^{13}C NMR spectrum of compound **17s**

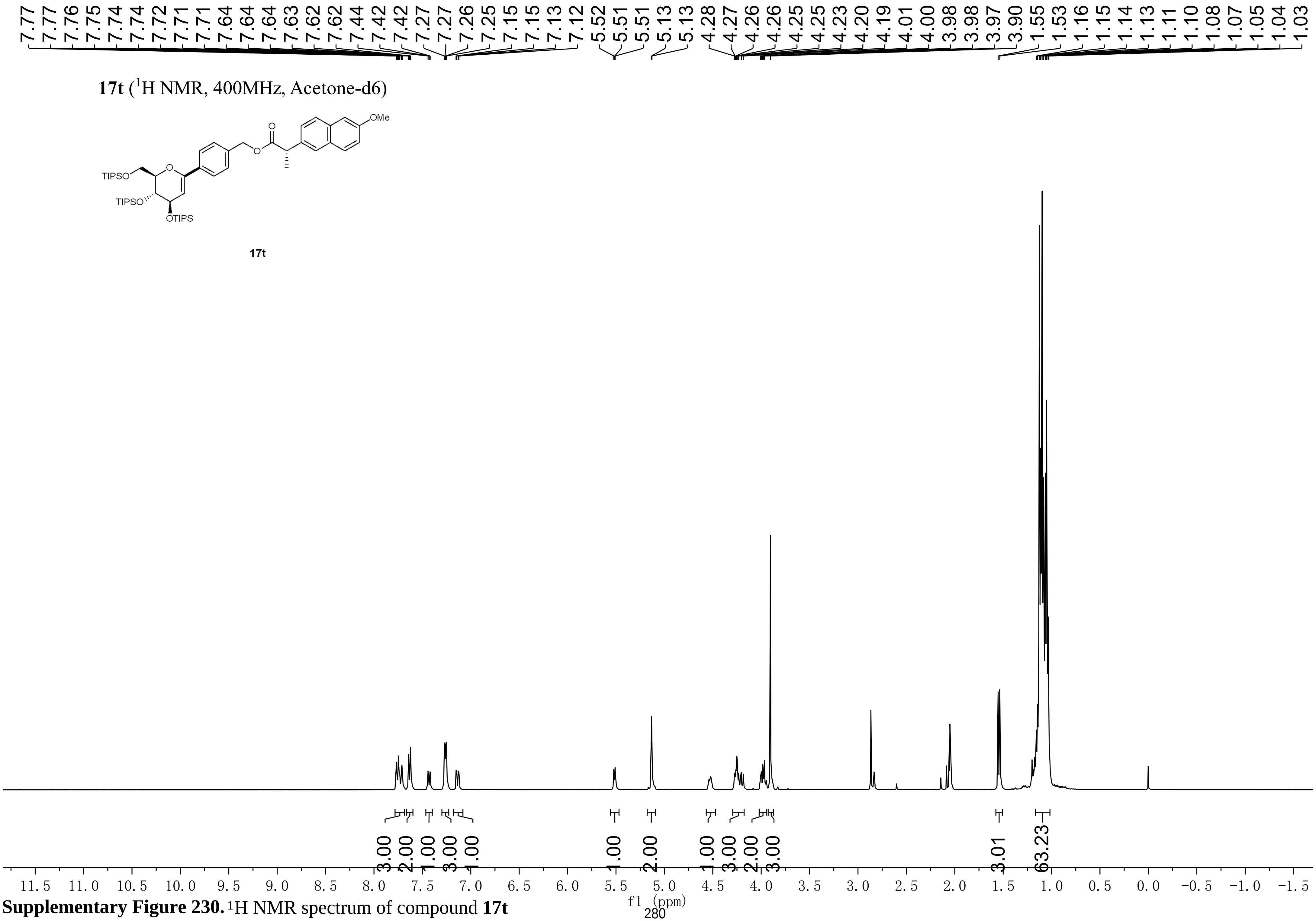

Supplementary Figure 230. ^{1}H NMR spectrum of compound **17t**

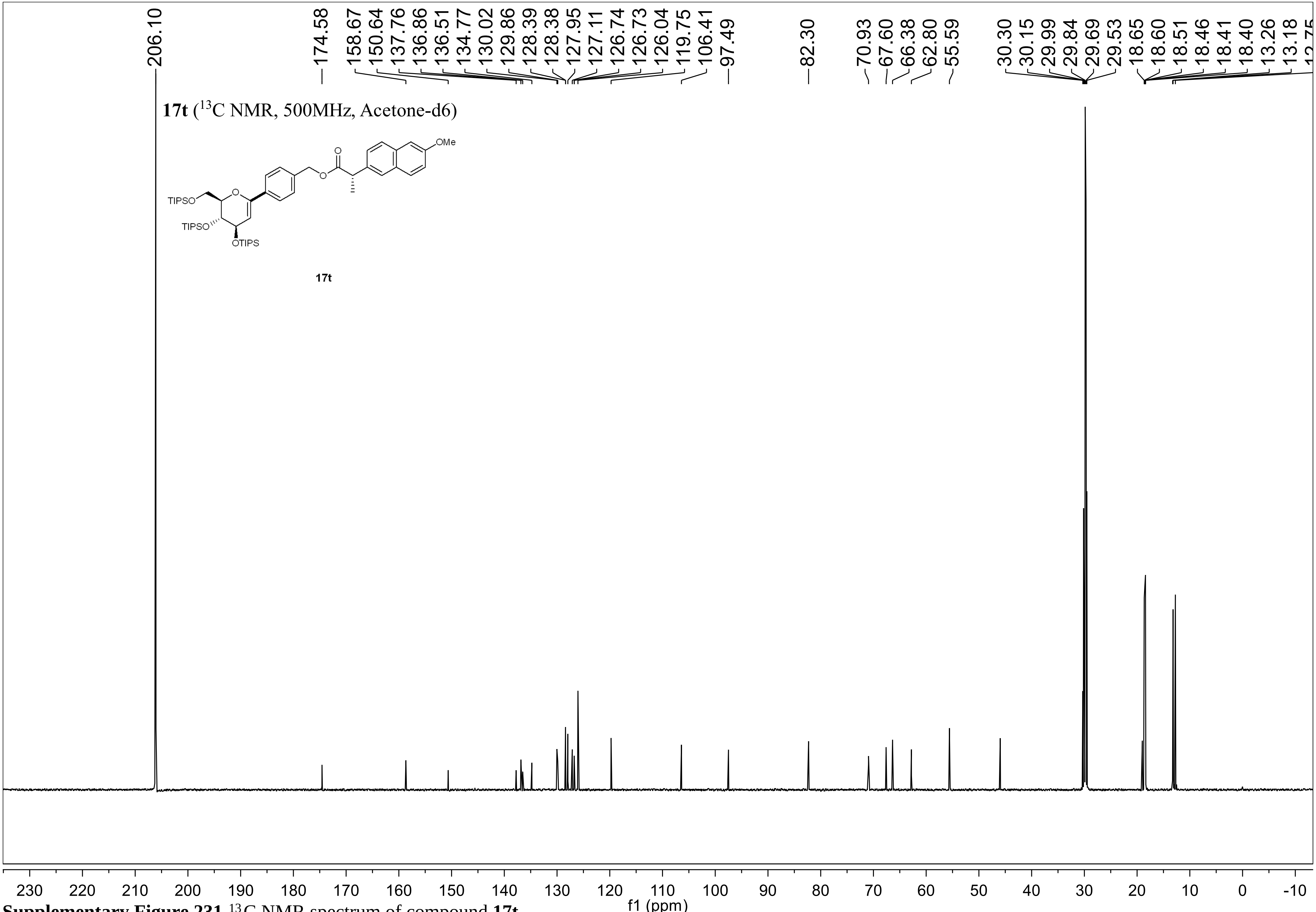

Supplementary Figure 231. ^{13}C NMR spectrum of compound **17t**

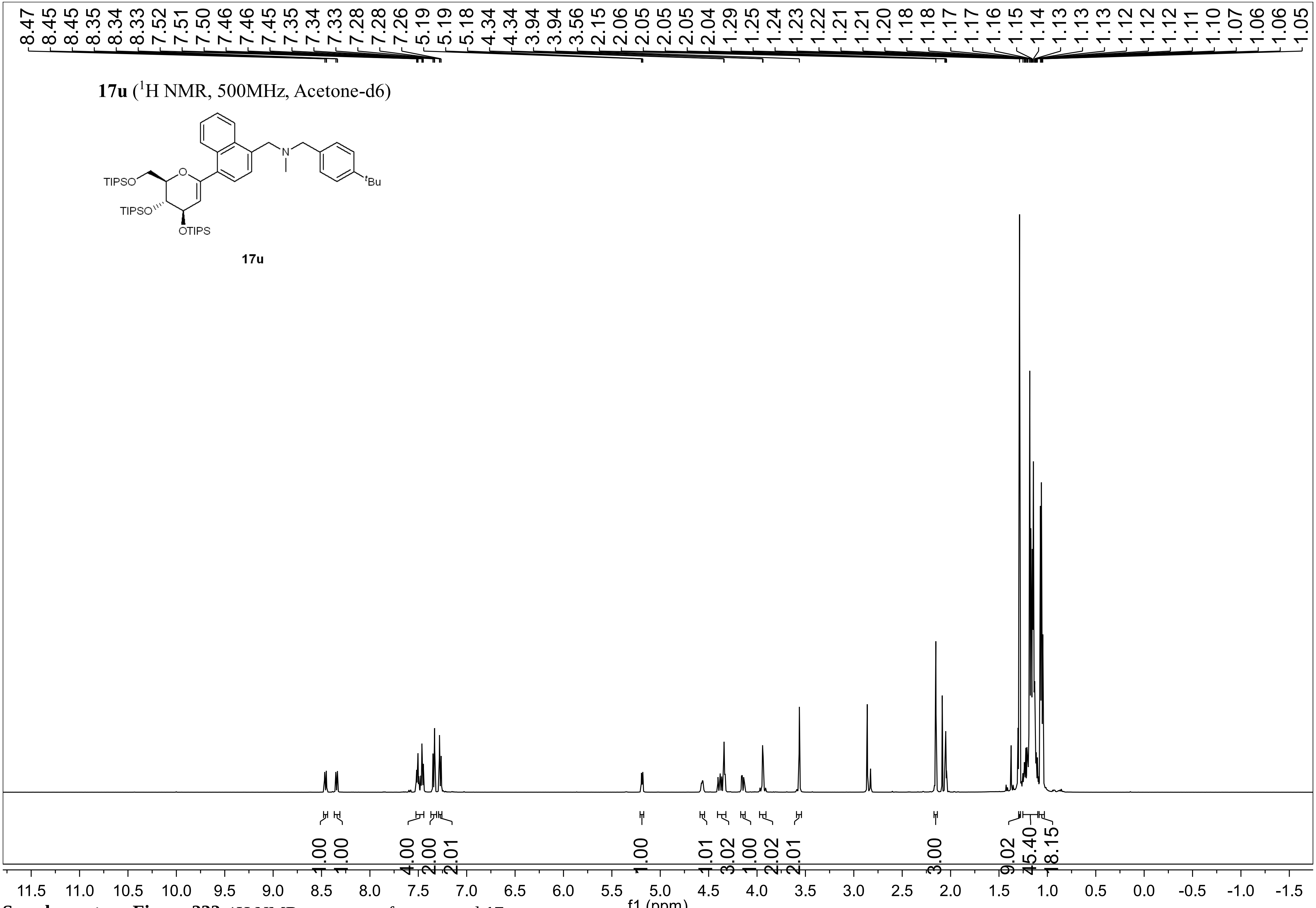

Supplementary Figure 232. ^{1}H NMR spectrum of compound **17u**

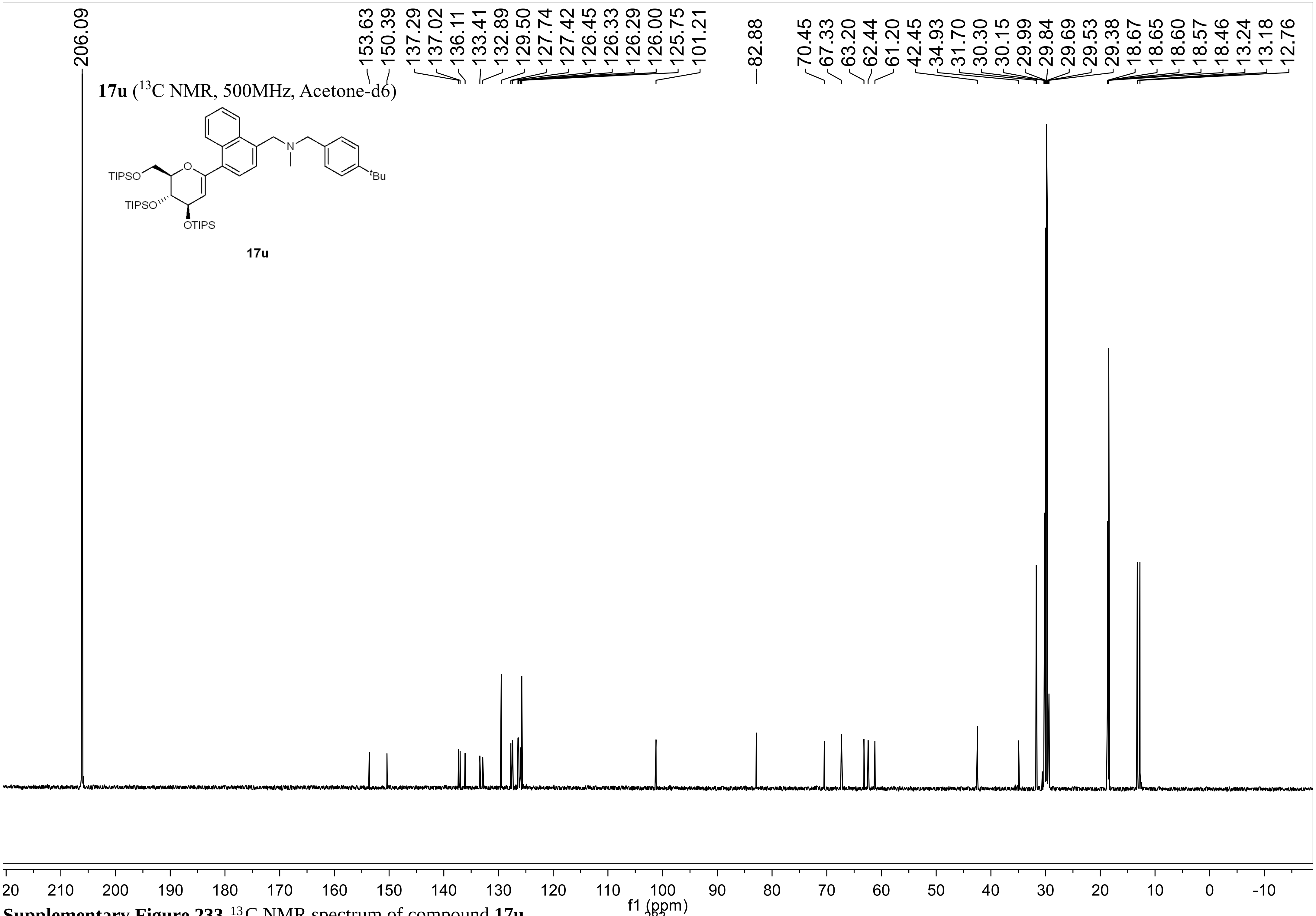

Supplementary Figure 233. ^{13}C NMR spectrum of compound **17u**

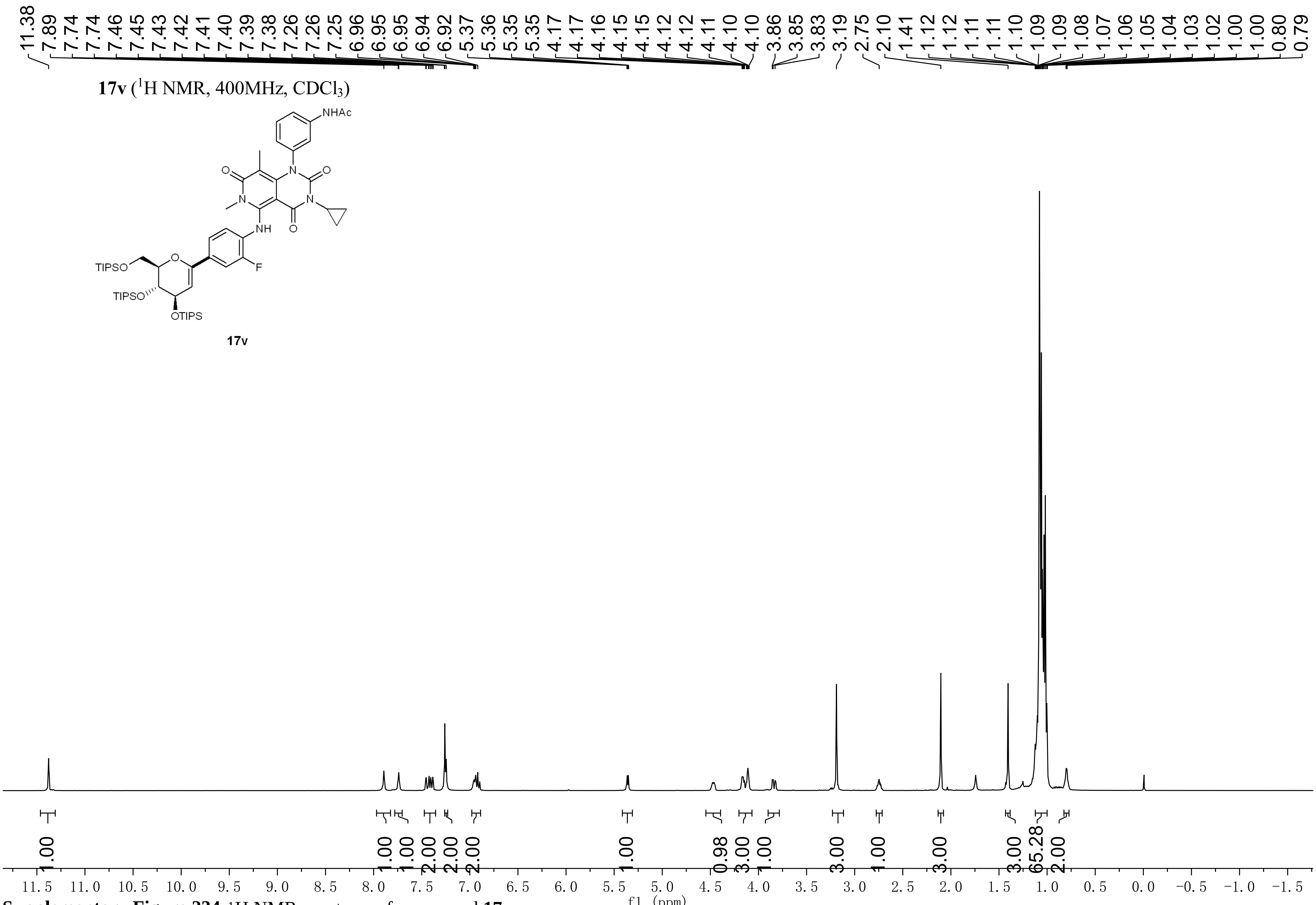

Supplementary Figure 234. 1H NMR spectrum of compound **17v**

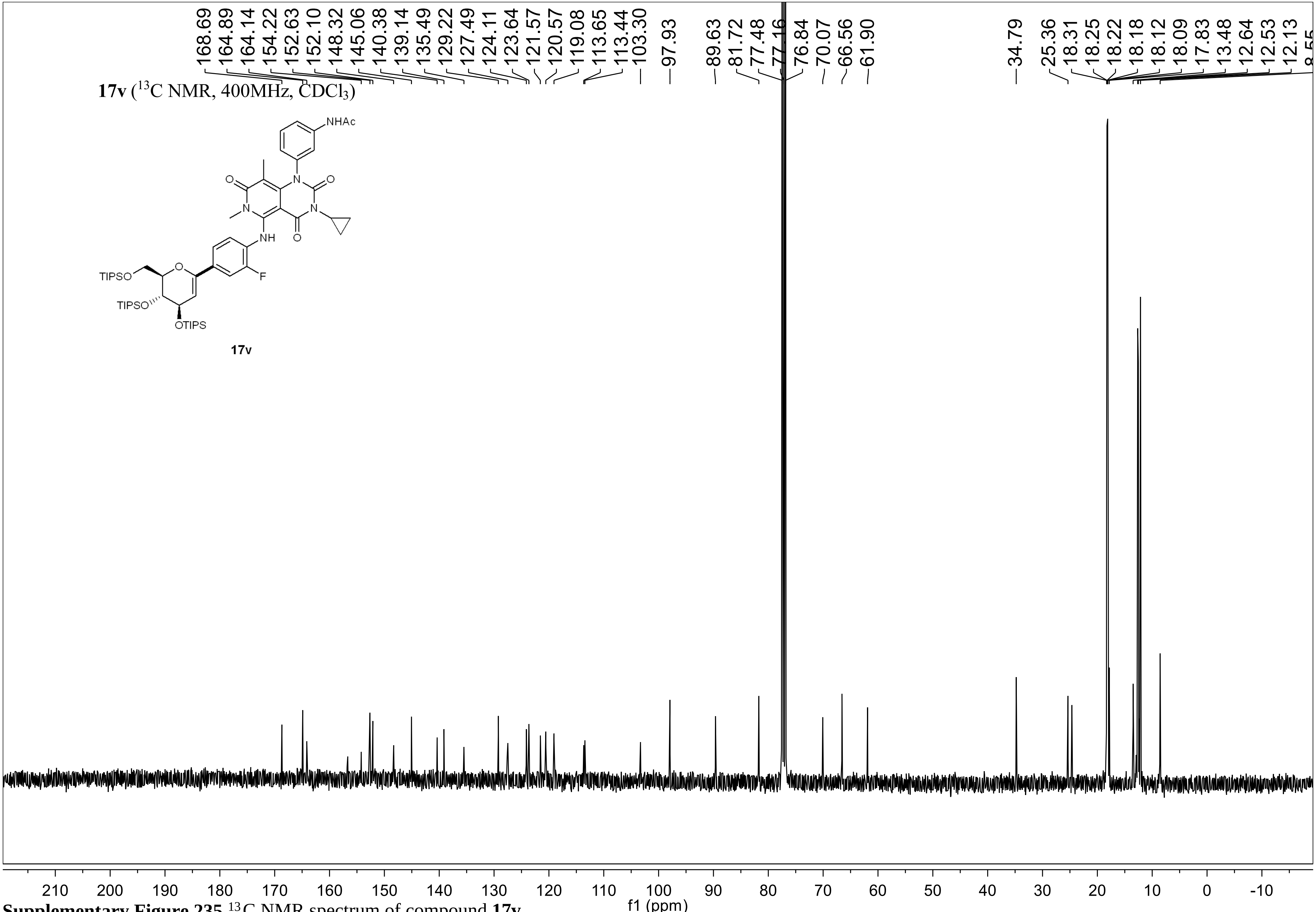

Supplementary Figure 235. ^{13}C NMR spectrum of compound **17v**

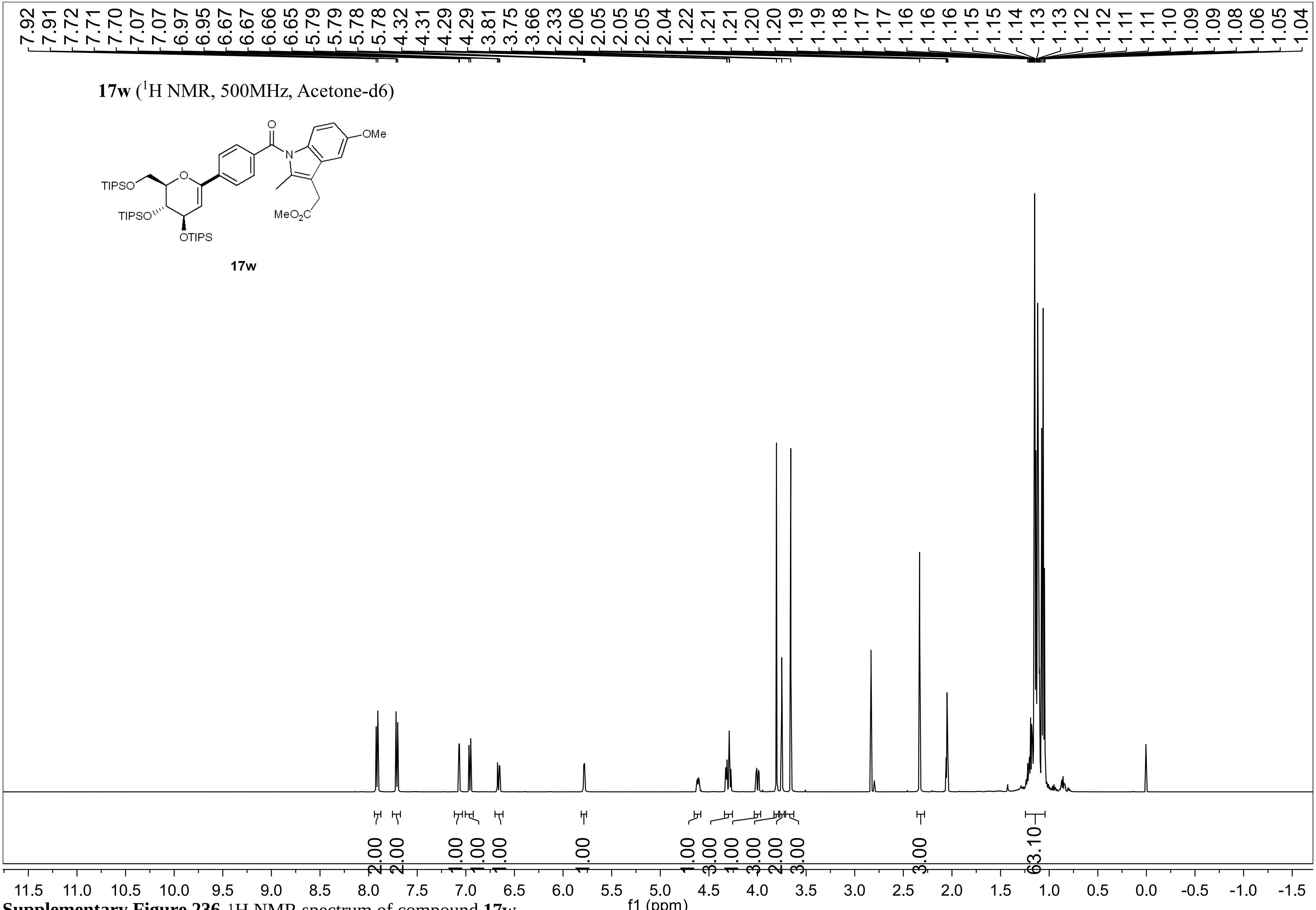

Supplementary Figure 236. ¹H NMR spectrum of compound **17w**

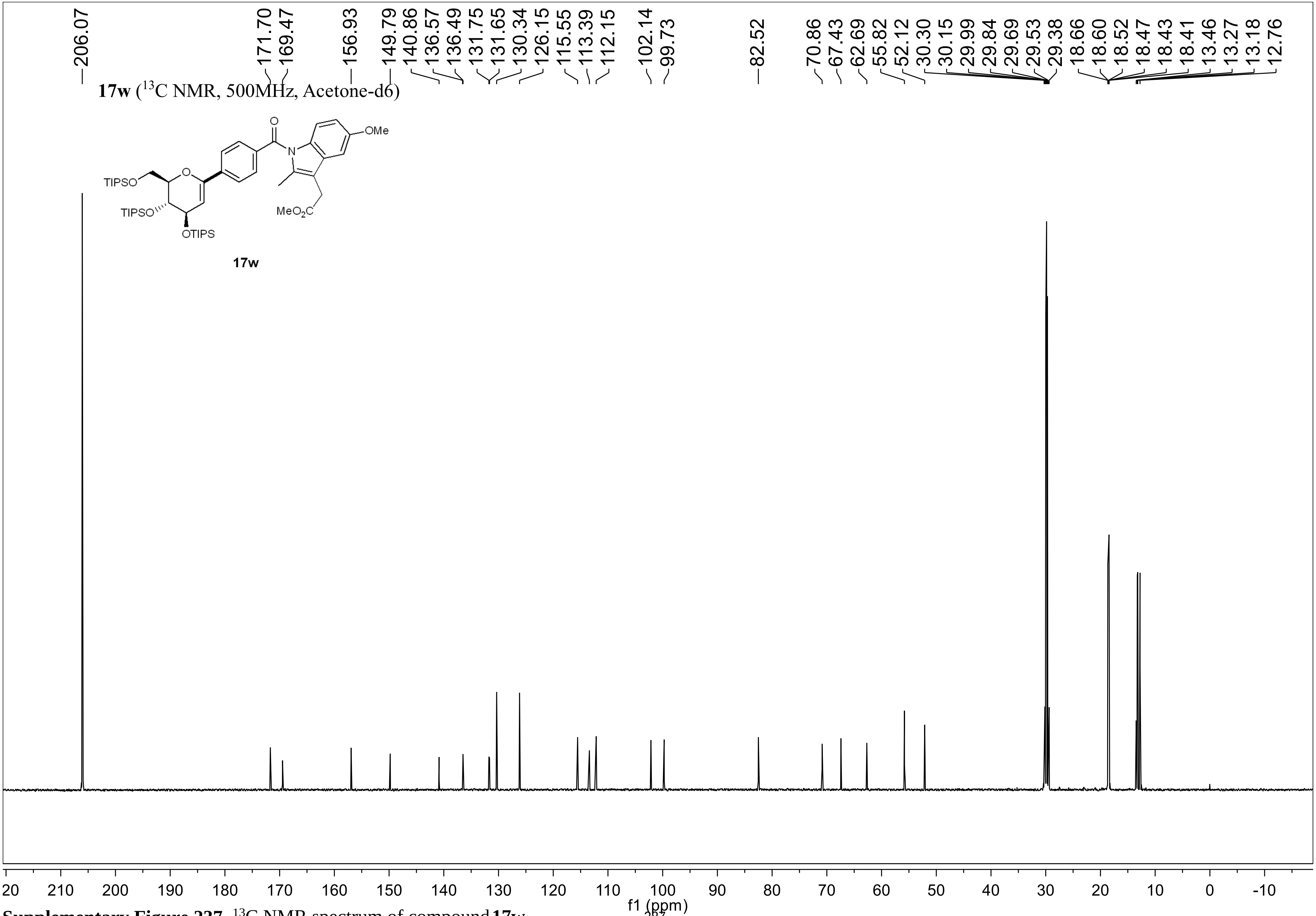

Supplementary Figure 237. ^{13}C NMR spectrum of compound **17w**

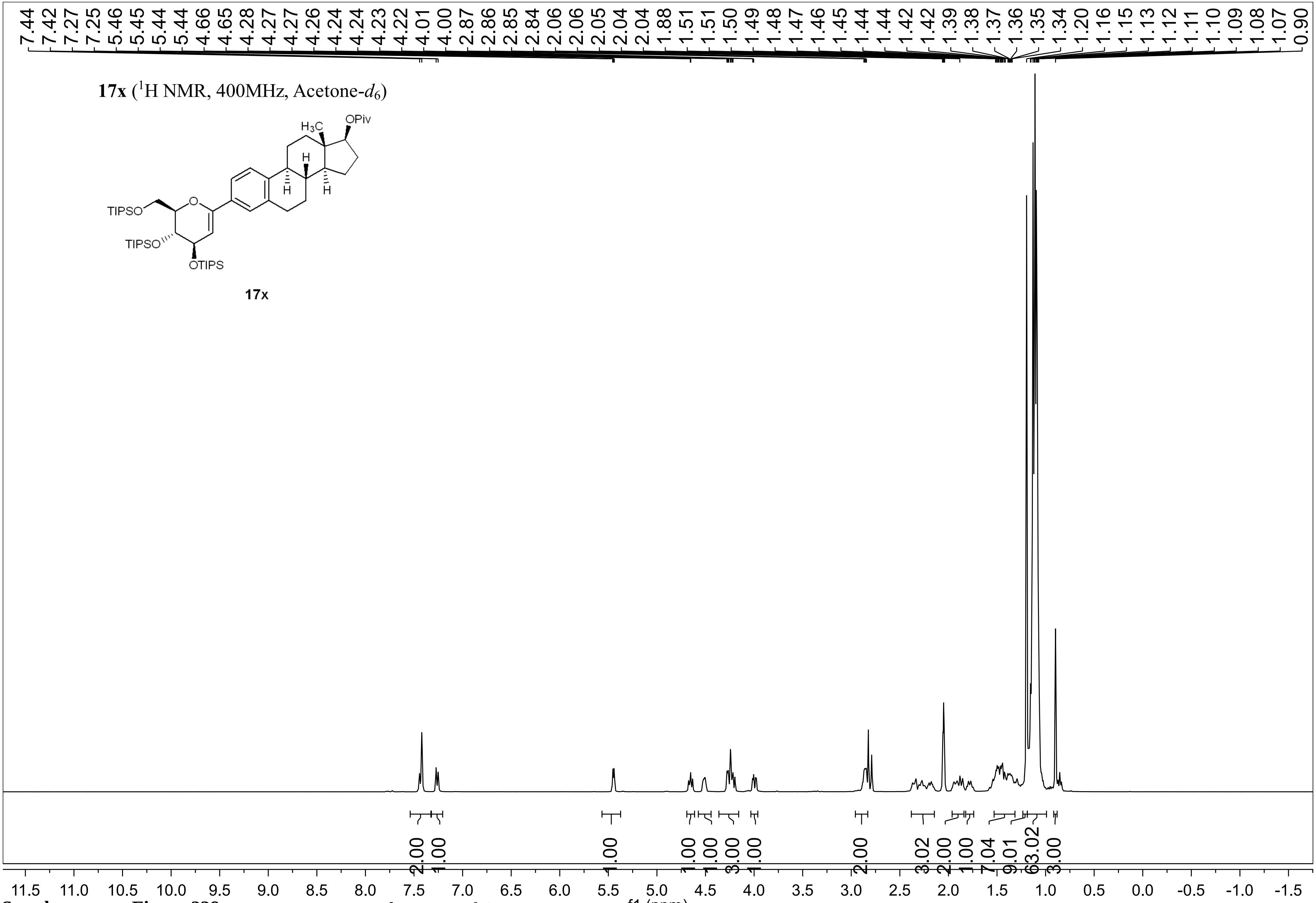

Supplementary Figure 238. ^{1}H NMR spectrum of compound **17x**

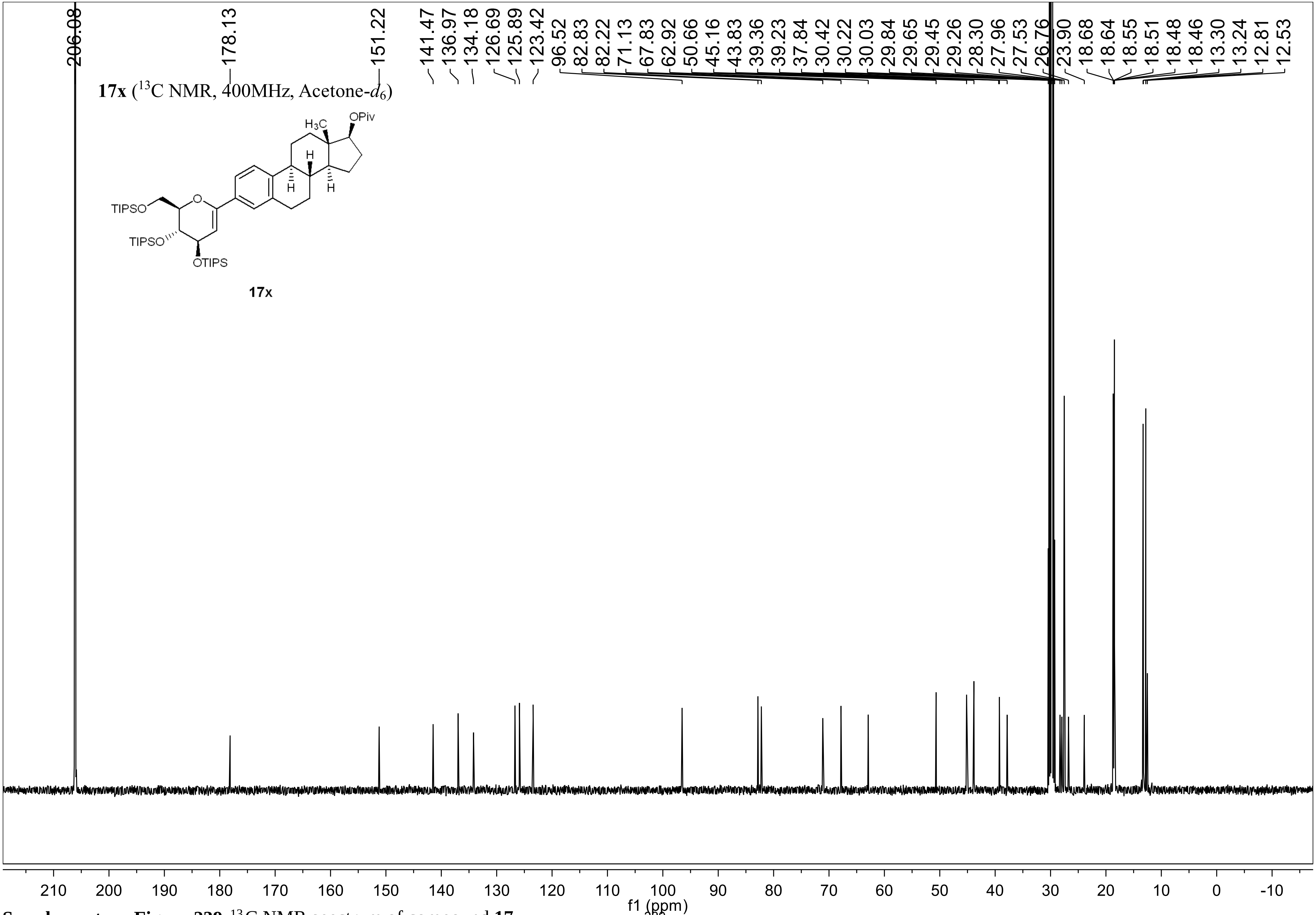

Supplementary Figure 239. ^{13}C NMR spectrum of compound **17x**

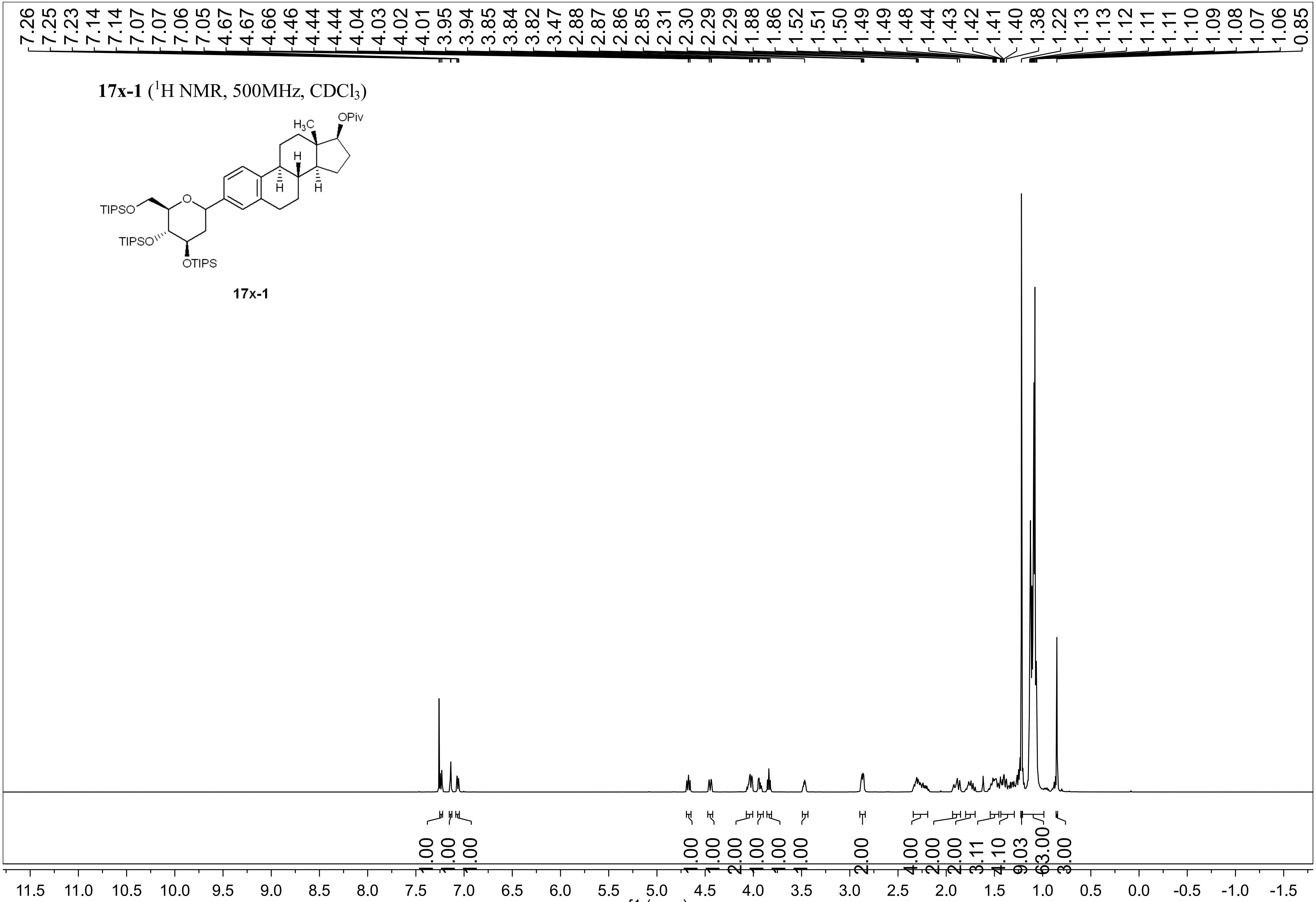

Supplementary Figure 240. ^{1}H NMR spectrum of compound **17x-1**

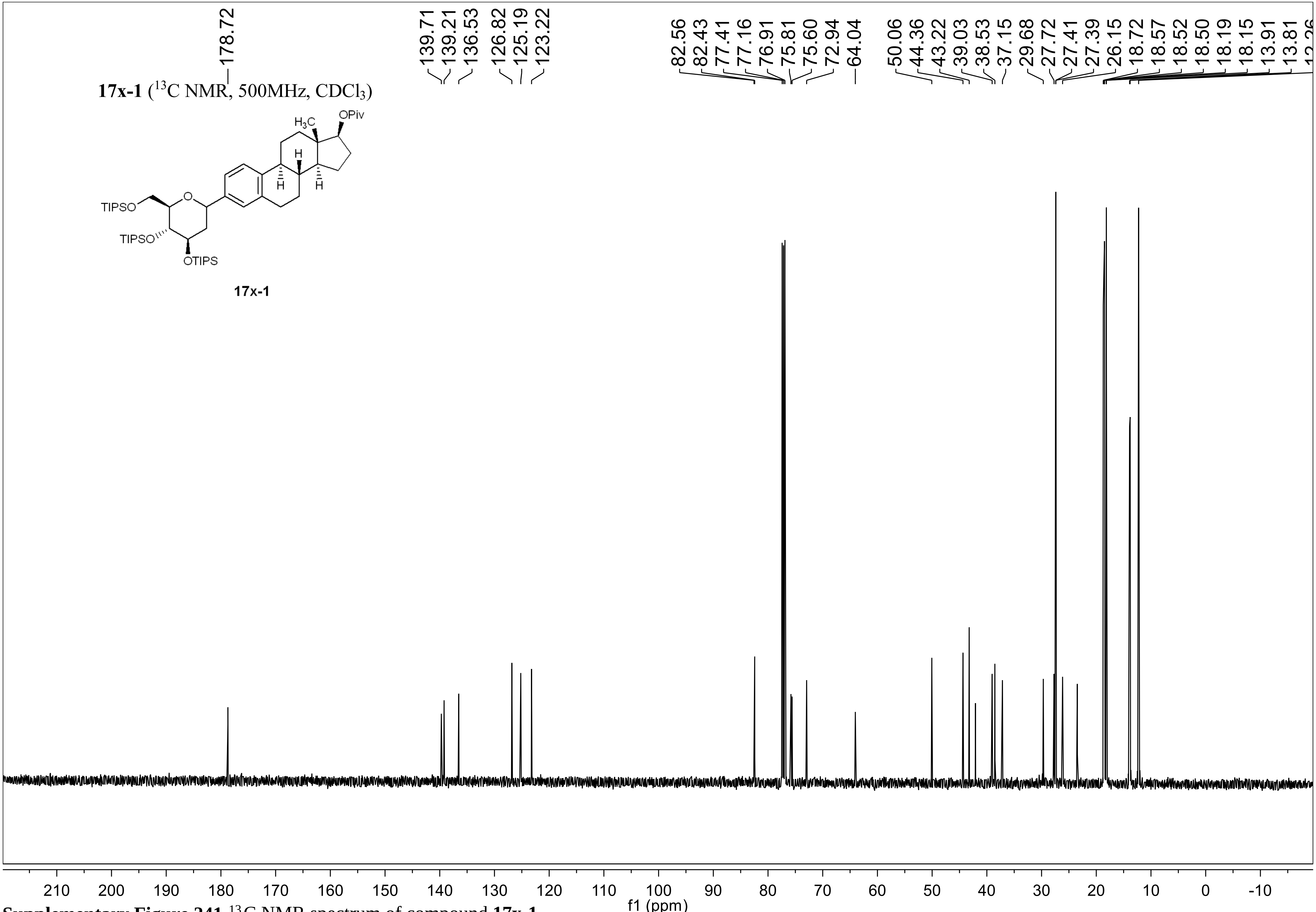

Supplementary Figure 241. ^{13}C NMR spectrum of compound **17x-1**

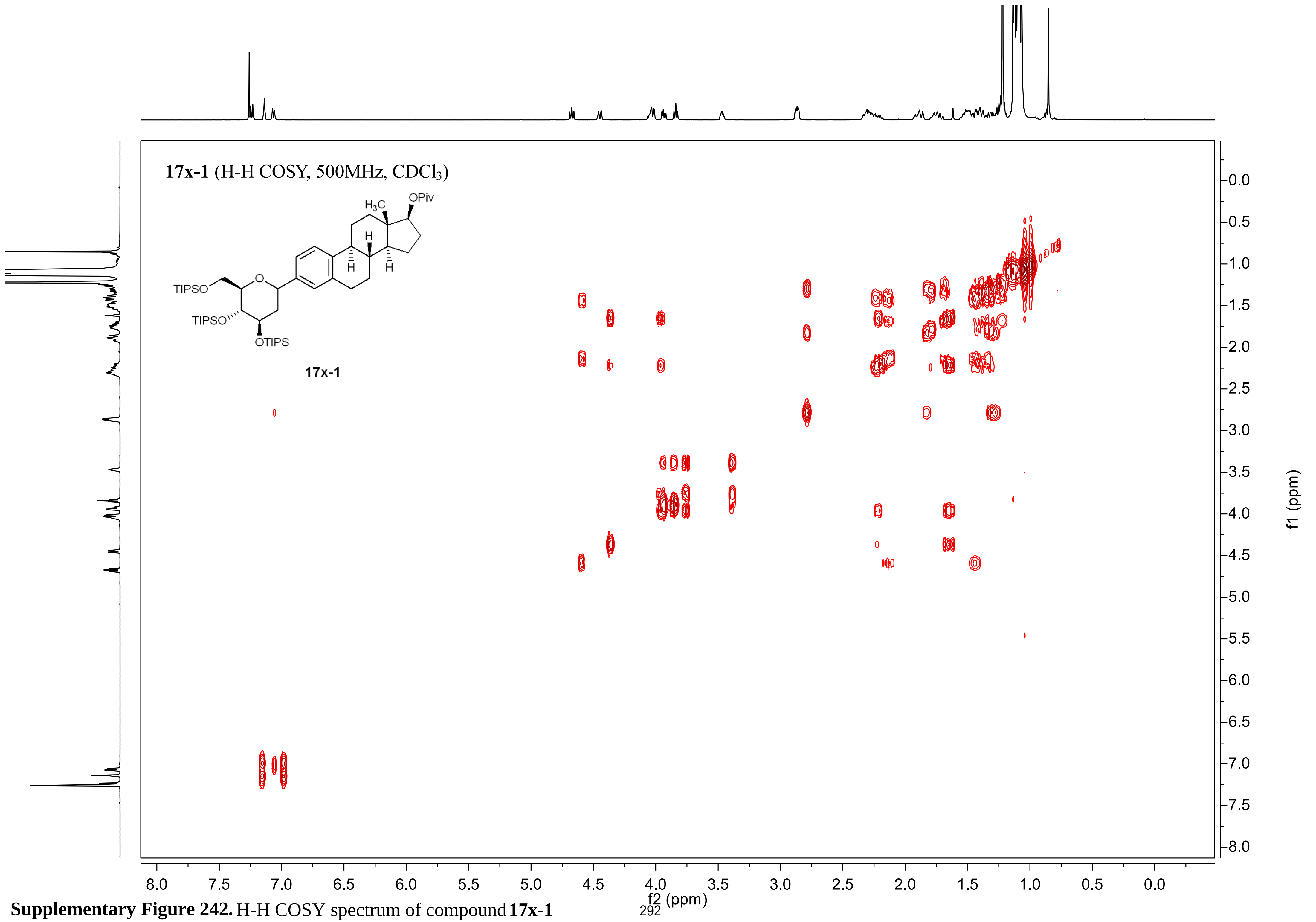

Supplementary Figure 242. H-H COSY spectrum of compound **17x-1**

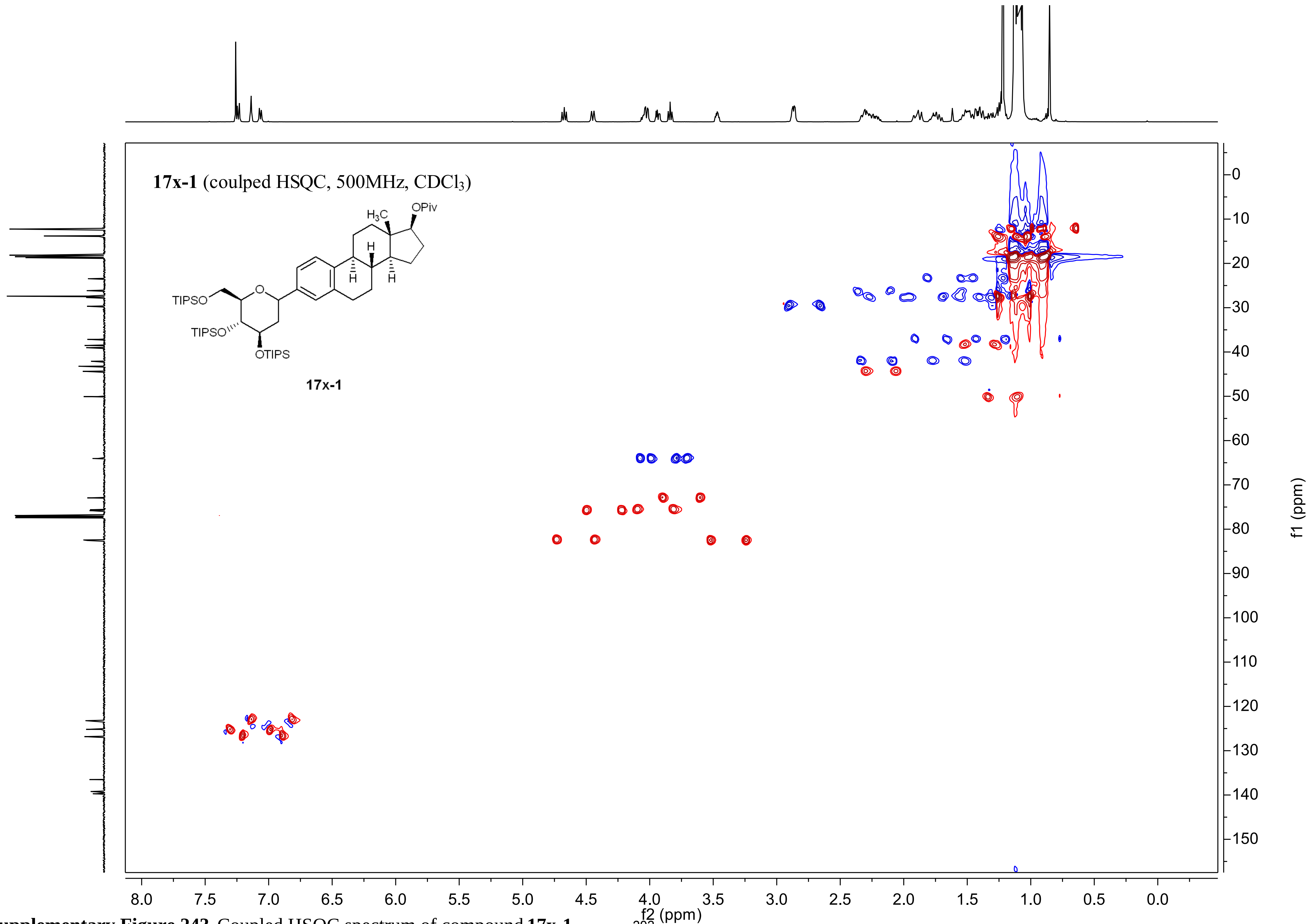

Supplementary Figure 243. Coupled HSQC spectrum of compound **17x-1**

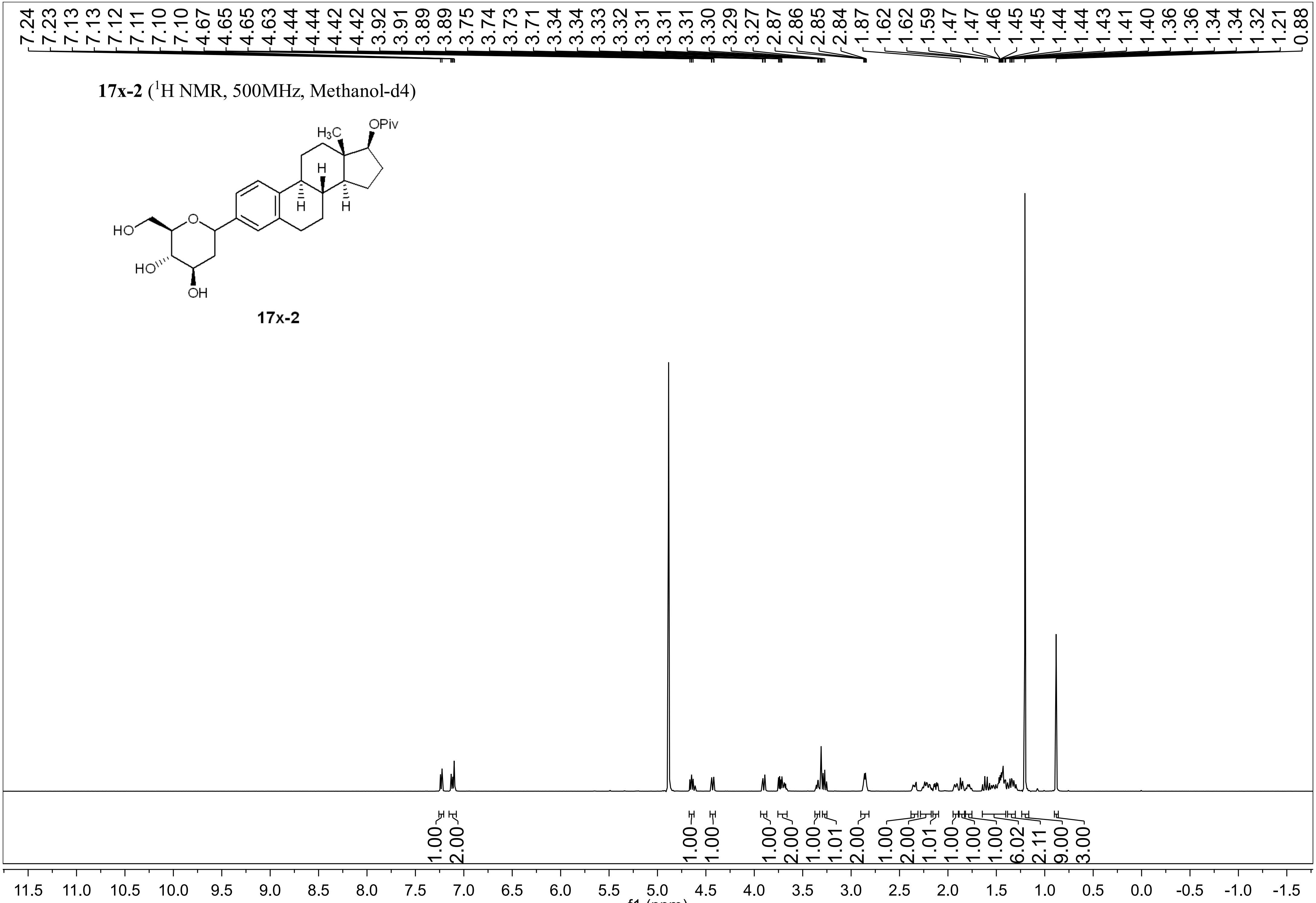

Supplementary Figure 244. ^{1}H NMR spectrum of compound **17x-2**

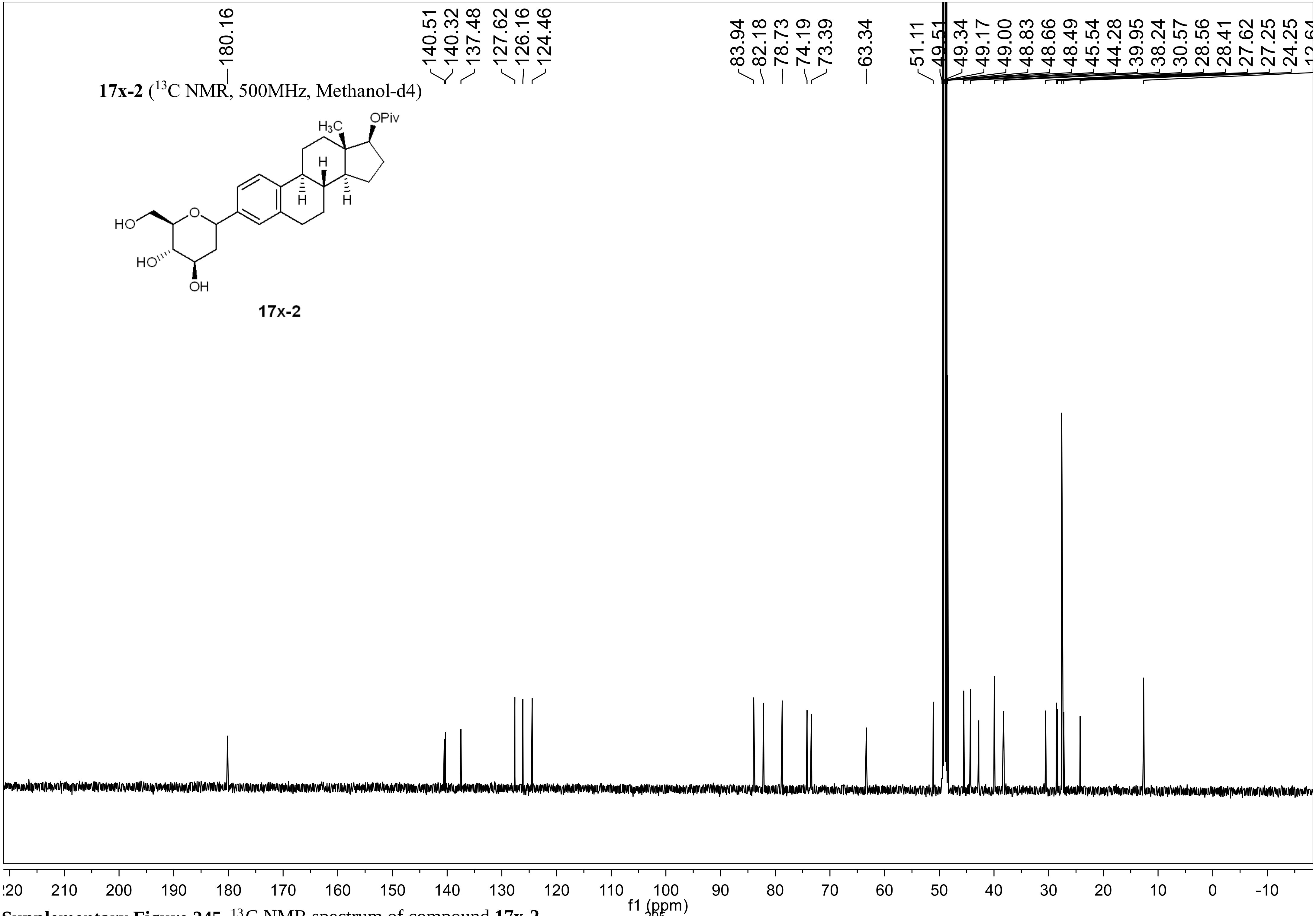

Supplementary Figure 245. ^{13}C NMR spectrum of compound **17x-2**

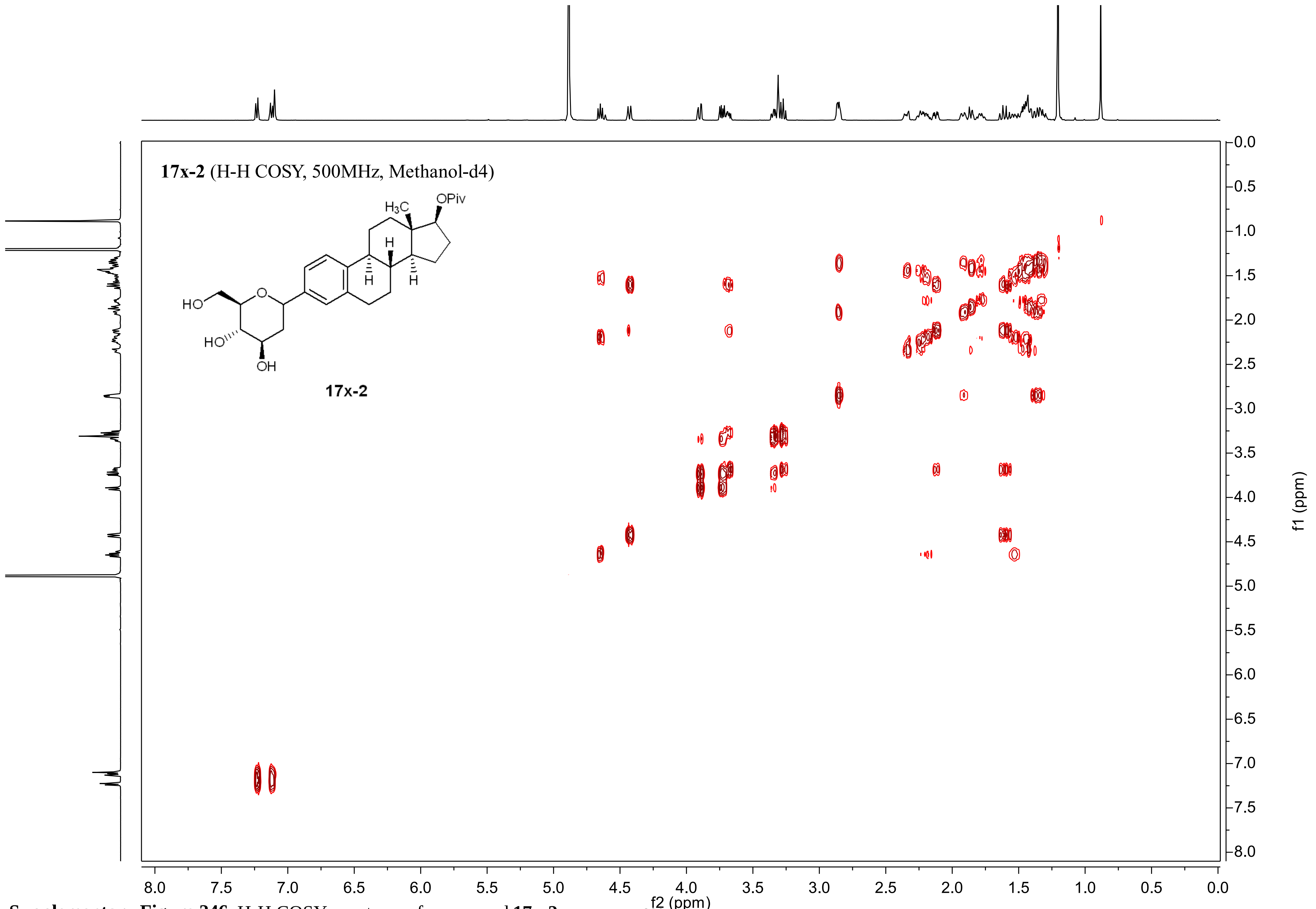

Supplementary Figure 246. H-H COSY spectrum of compound **17x-2**

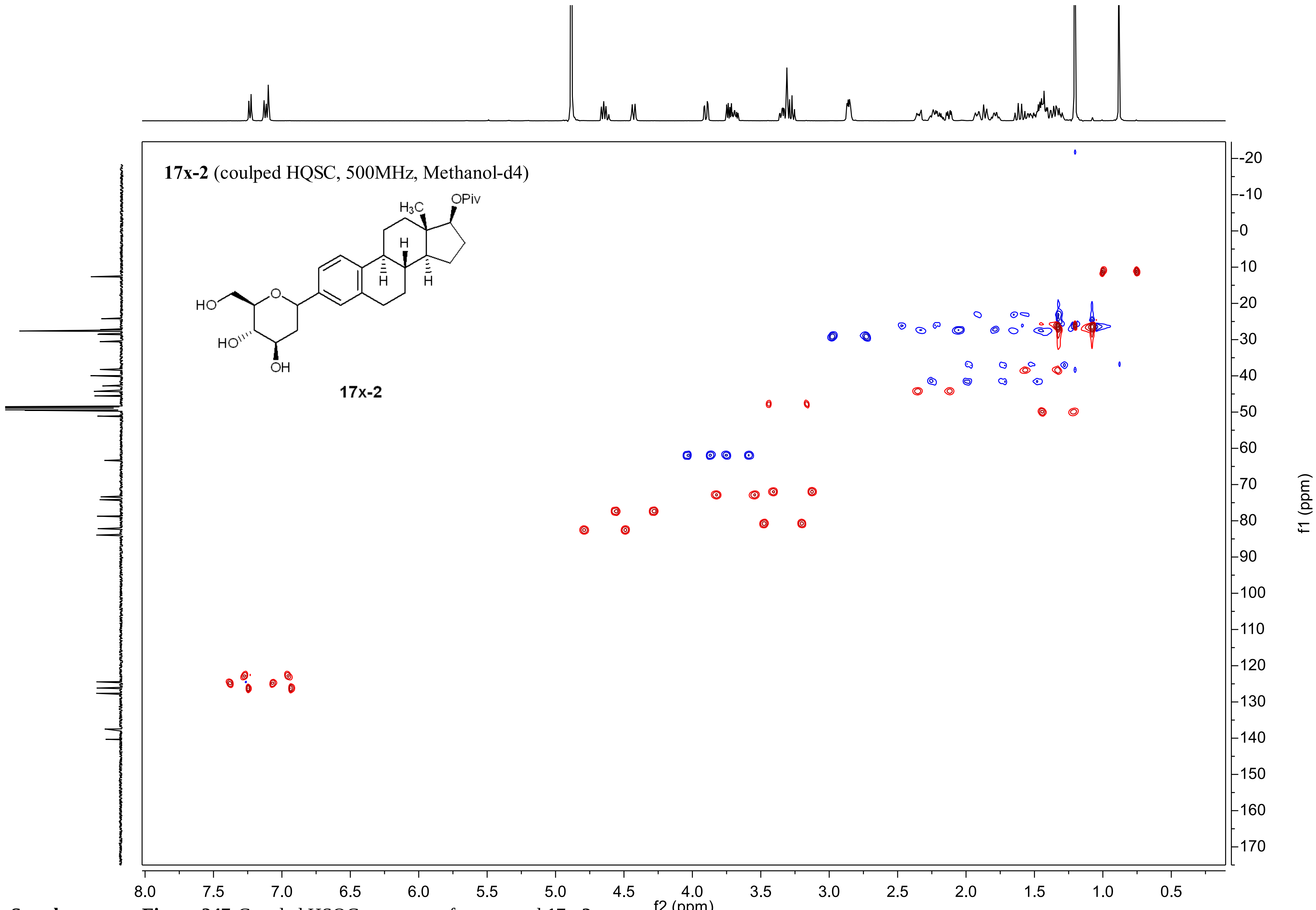

Supplementary Figure 247. Coupled HSQC spectrum of compound **17x-2**

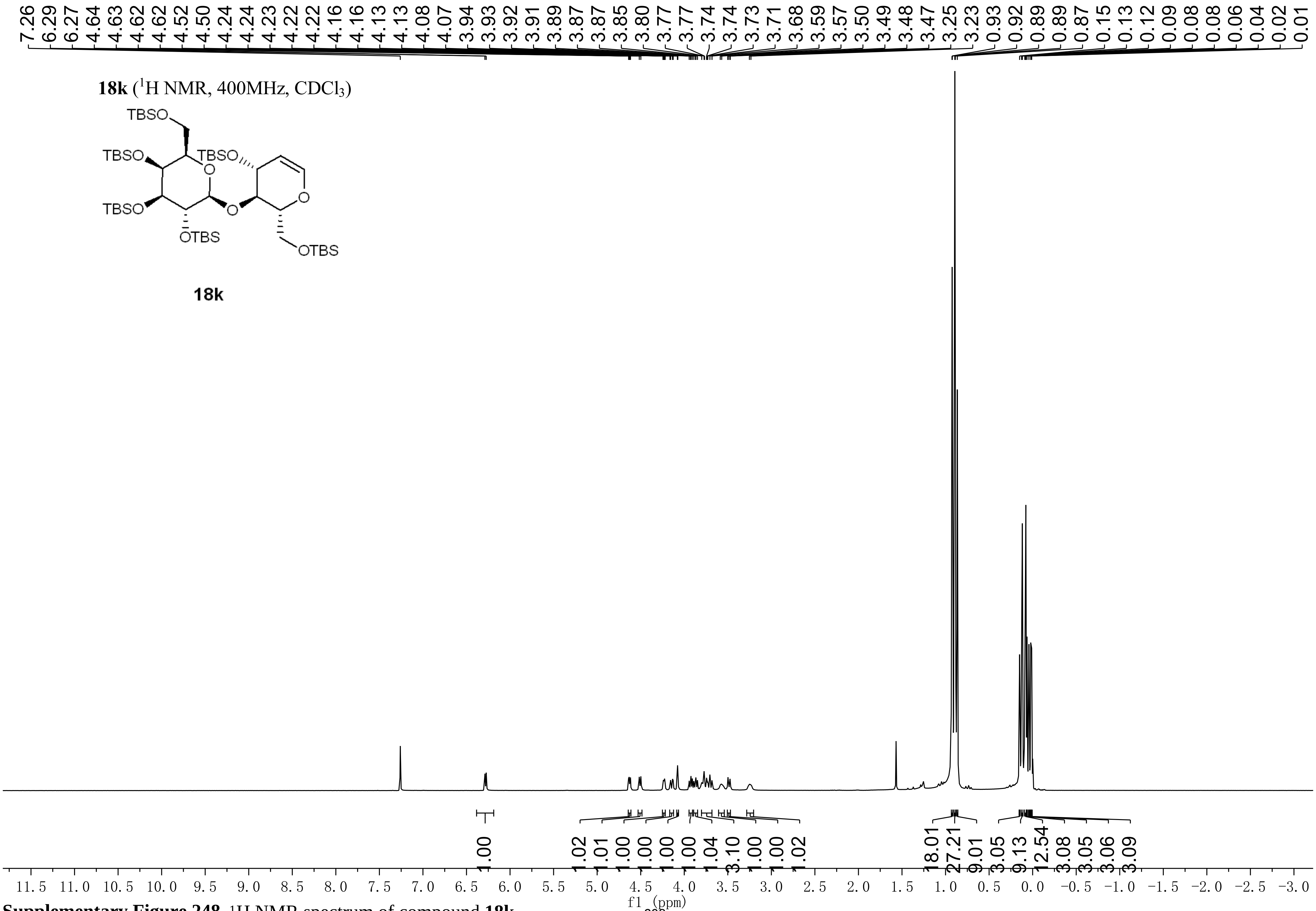

Supplementary Figure 248. ^{1}H NMR spectrum of compound **18k**

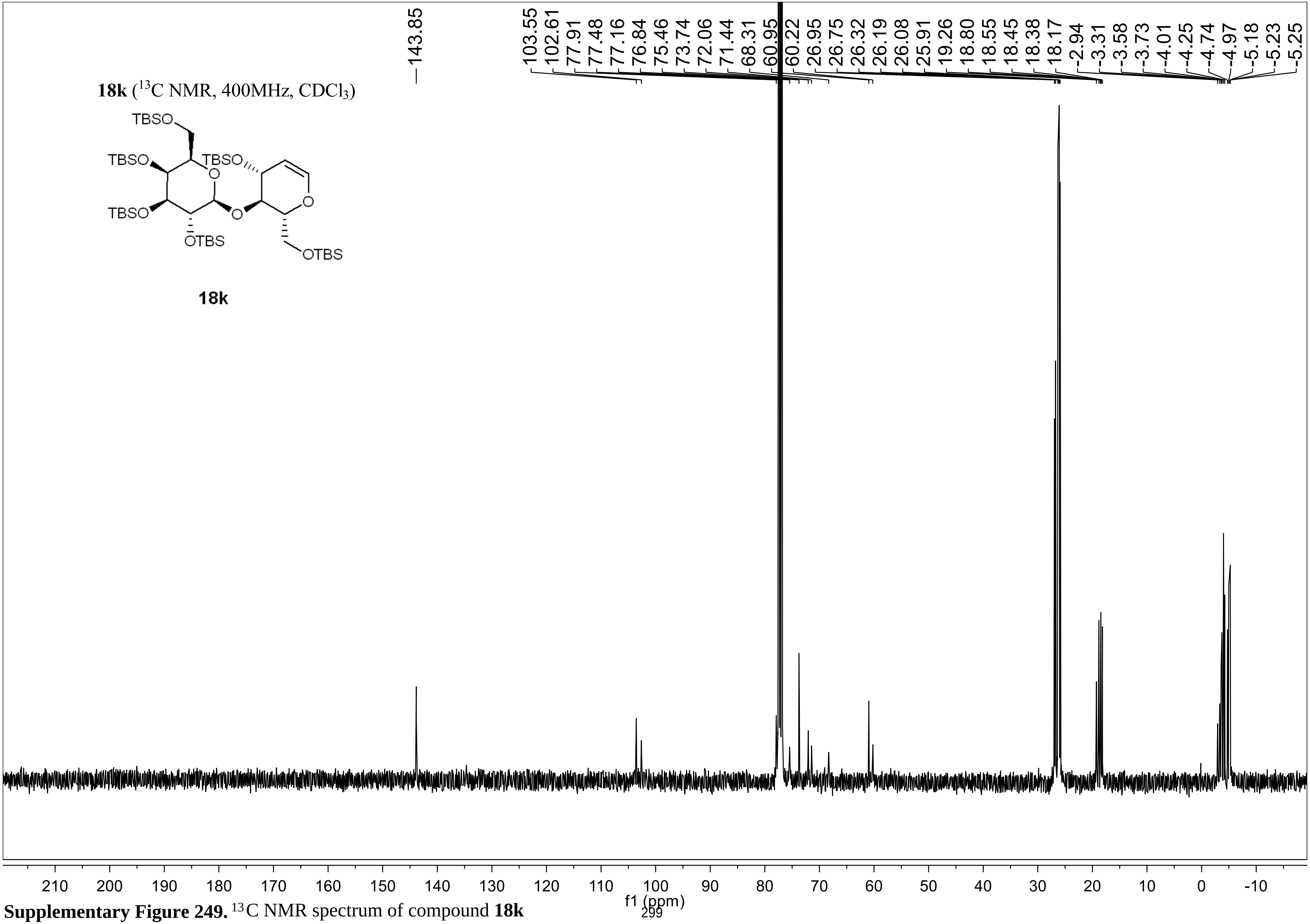

Supplementary Figure 249. ^{13}C NMR spectrum of compound **18k**

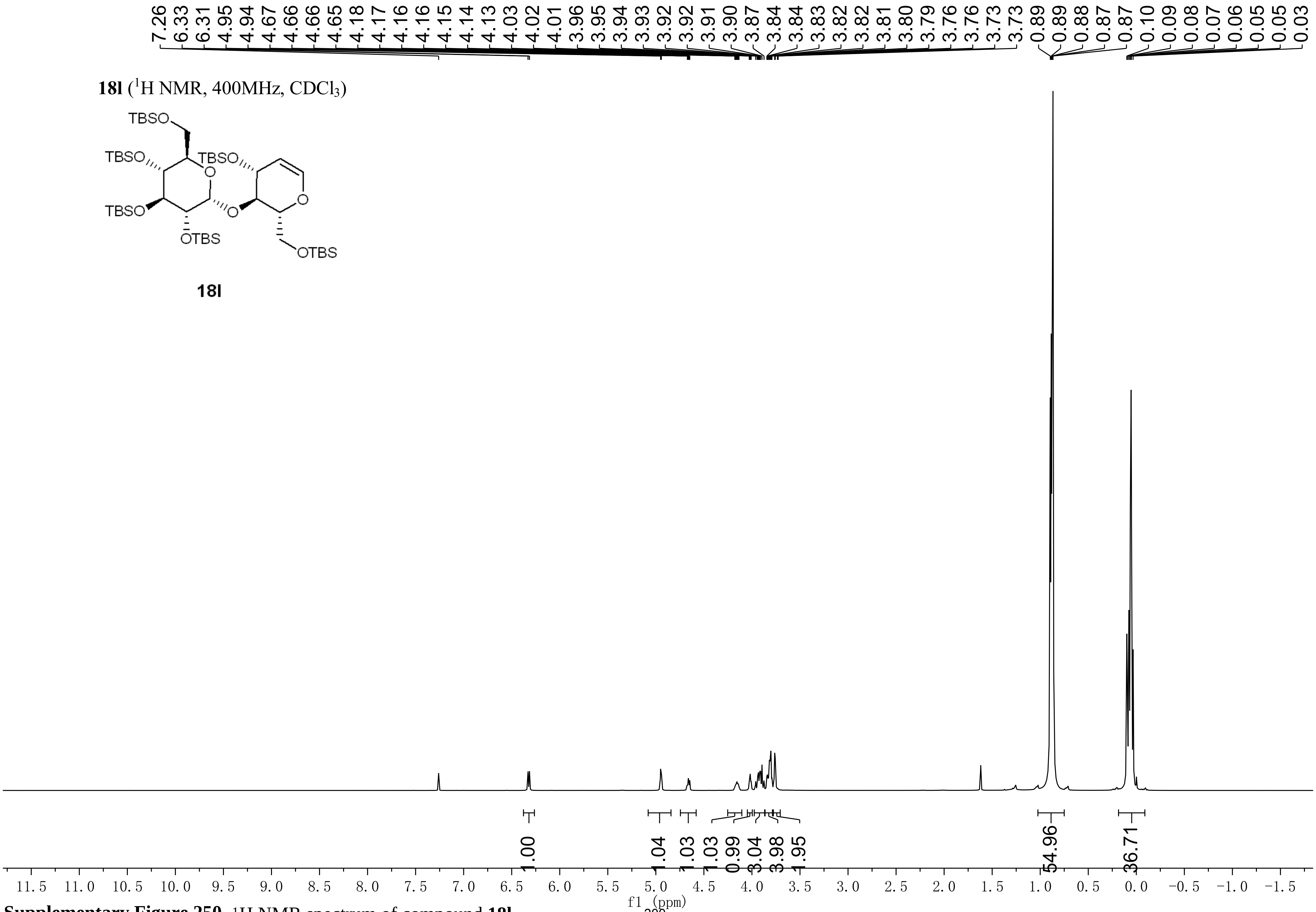

Supplementary Figure 250. ^{1}H NMR spectrum of compound **18l**

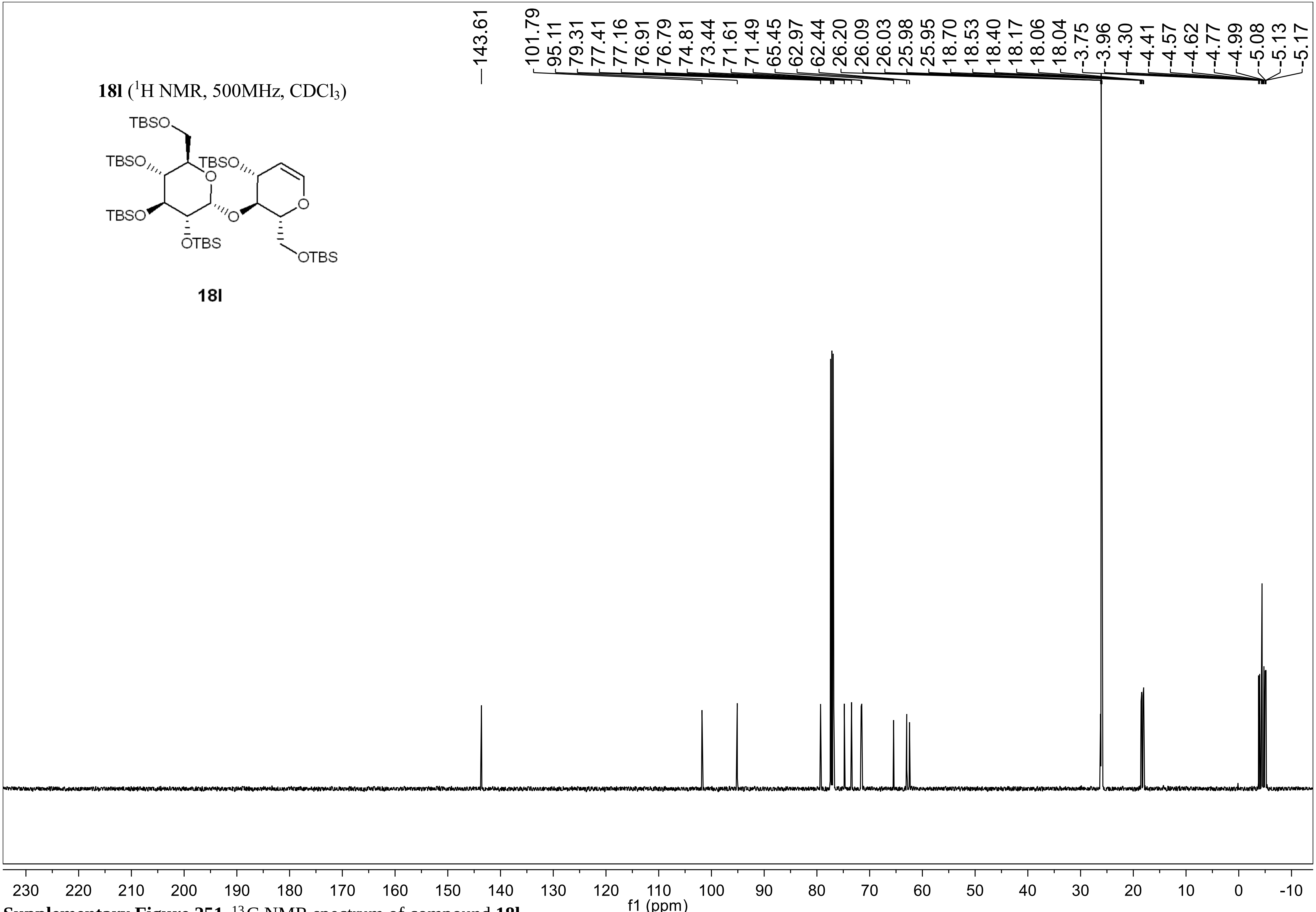

Supplementary Figure 251. ^{13}C NMR spectrum of compound **18l**

3. Supplementary References

1 Mendonca, S. & Laine, R. A. Synthesis of sterically crowded derivatives of anomeric pairs of D-glucose disaccharides. *Carbohydr. Res.* **340**, 2055-2059 (2005).

2 Kikuchi, T., Takagi, J., Isou, H., Ishiyama, T. & Miyaura, N. Vinylic C-H borylation of cyclic vinyl ethers with bis(pinacolato)diboron catalyzed by an iridium(I)-dtbpy complex. *Chem. Asian. J.* **3**, 2082-2090 (2008).

3 Zhao, J., Shao, H., Wu, X. & Shi, S. A rapid synthesis of pyranoid glycals promoted by β-cyclodextrin and ultrasound. *Chin. J. Chem.* **29**, 1434-1440 (2011).

4 Zhang, S., Niu, Y.-H. & Ye, X.-S. General approach to five-membered nitrogen heteroaryl *C*-glycosides using a palladium/copper cocatalyzed C–H functionalization strategy. *Org. Lett.* **19**, 3608-3611 (2017).

5 Takeda, D. *et al.* β-Glycosyl Trifluoroborates as precursors for direct α-*C*-glycosylation: Synthesis of 2-deoxy-α-*C*-glycosides. *Org. Lett.* **23**, 1940-1944 (2021).

6 Apsel, B. *et al.* General entries to *C*-aryl glycosides. formal synthesis of galtamycinone. *Tetrahedron Lett.* **44**, 1075-1077 (2003).

7 Verano, A. L. & Tan, D. S. Family-level stereoselective synthesis and biological evaluation of pyrrolomorpholine spiroketal natural product antioxidants. *Chem. Sci.* **8**, 3687-3693 (2017).

8 Sakamaki, S., Kawanishi, E., Nomura, S. & Ishikawa, T. Aryl-β-*C*-glucosidation using glucal boronate: Application to the synthesis of tri-*O*-methylnorbergenin. *Tetrahedron* **68**, 5744-5753 (2012).

9 Bol′shakov, O. I., Lebedyeva, I. O. & Katritzky, A. R. 17α-Ethynylestradiol peptide labeling by ‘click’ chemistry. *Synthesis* **44**, 2926-2932 (2012).

10 Dolganov, A. V. *et al.* Synthesis and electrochemical properties of 2,5-disubstituted derivatives of 1,4-bis(4,5-diphenylidimidazol-2-yl)benzene. *Russ. J. Gen. Chem.* **90**, 961-967 (2020).

11 He, L. Y. *et al.* Nucleophile-selective cross-coupling reactions with vinyl and alkynyl bromides on a dinucleophilic aromatic substrate. *Eur. J. Org. Chem.* **2015**, 2498-2502 (2015).

12 Tang, K. G., Kent, G. T., Erden, I. & Wu, W. cis-β-Bromostyrene derivatives from cinnamic acids via a tandem substitutive bromination-decarboxylation sequence. *Tetrahedron Lett.* **58**, 3894-3896 (2017).

13 Shi, W., Guan, Z., Cai, P. & Chen, H. Highly efficient and recyclable catalyst for the direct chlorination, bromination and iodination of terminal alkynes. *J. Catal.* **353**, 199-204 (2017).

14 Galler, D. J. & Parker, K. A. Five easy pieces. the total synthesis of phosphoiodyn A (and placotylene A). *Org. Lett.* **17**, 5544-5546 (2015).

15 Wang, G. *et al.* Design, synthesis and preliminary bioactivity studies of imidazolidine-2,4-dione derivatives as Bcl-2 inhibitors. *Bioorg. Med. Chem.* **23**, 7359-7365 (2015).

16 Zhou, Z. *et al*. Photoredox nickel-catalysed stille cross-coupling reactions. *Angew. Chem. Int. Ed.* **62**, e202314832 (2023).
17 Bacauanu, V. *et al*. Metallaphotoredox difluoromethylation of aryl bromides. *Angew. Chem. Int. Ed.* **57**, 12543-12548 (2018).
18 Zhu, F. *et al*. Umpolung Ala^B reagents for the synthesis of non-proteogenic amino acids, peptides and proteins. *Angew. Chem. Int. Ed.* **61**, e202207153 (2022).
19 Hostetler, E. D., Jonson, S. D.; Welch, M. J. & Katzenellenbogen, J. A. Synthesis of 2-[^{18}f]-fluoroestradiol, a potential diagnostic imaging agent for breast cancer: Strategies to achieve nucleophilic substitution of an electron-rich Aromatic ring with [^{18}F]F^-. *J. Org. Chem.* **64**, 178-185 (1999).
20 Chang, Y.-S., Jang, J.-S. & Delnzer, M. L. Photochemistry of irgasan-triflate: A simple conversion of an aromatic hydroxyl group to chlorine in the synthesis of polychlorinated diphenyl ethers and polychlorinated dibenzofurans. *Tetrahedron* **46**, 4161-4164 (1990).
21 Badir, S. O. *et al*. Multifunctional building blocks compatible with photoredox-mediated alkylation for DNA-encoded library synthesis. *Org. Lett.* **22**, 1046-1051 (2020).
22 Qi, J., Liu, S., Seydimemet, M., Wang, X. & Lu, X. A general set of DNA-compatible reactions for preparing DNA-tagged multisubstituted pyrroles. *Bioconjugate Chem.* **32**, 2290-2294 (2021).
23 Talode, J. *et al*. Syntheses of SGLT2 inhibitors by Ni- and Pd-catalyzed fukuyama coupling reactions. *J. Org. Chem.* **85**, 12382-12392 (2020).
24 Gong, L. *et al*. Ni-catalyzed suzuki–miyaura cross-coupling of α-oxo-vinylsulfones to prepare *C*-aryl glycals and acyclic vinyl ethers. *J. Am. Chem. Soc.* **141**, 7680-7686 (2019).
25 Murakata, M. *et al*. Synthesis of tofogliflozin as an SGLT2 inhibitor via construction of dihydroisobenzofuran by intramolecular [4 + 2] cycloaddition. *Org. Process Res. Dev.* **23**, 548-557 (2019).
26 Ohtake, Y. *et al*. Development of a scalable synthesis of tofogliflozin. *J. Org. Chem.* **81**, 2148-2153 (2016).
